# Precise borylation of targeted methyl group via an orderly chain-walking strategy

**DOI:** 10.1126/sciadv.aed6913

**Published:** 2026-05-01

**Authors:** Yinwei Bao, Chenke Hu, Lixuan Zheng, Yitao Wu, Feihe Huang, Zhan Lu

**Affiliations:** ^1^ Center of Chemistry for Frontier Technologies, State Key Laboratory of Soil Pollution Control and Safety, Department of Chemistry, Zhejiang University, Hangzhou 310058, China.; ^2^ ZJU-Hangzhou Global Scientific and Technological Innovation Center, Zhejiang University, Hangzhou 311215, China.; ^3^ State Key Laboratory of Coordination Chemistry, Nanjing University, Nanjing 210023, China.

## Abstract

C─H functionalization is an attractive approach in organic synthesis due to its directness and efficiency. Nevertheless, achieving precise differentiation among multiple methyl groups with highly similar chemical environments remains a notable challenge. Here, an orderly chain-walking borylation strategy was introduced via targeted hook and enduring slide that enables precise remote C(*sp^3^*)-H borylation and affords a series of “Y-shape” boron analogs through rational desymmetric ligand design. This method exhibits broad substrate scope, good functional group tolerance, and high atom economy and achieves a chain-walking distance of 32 carbons, and we hope it will promote cross-disciplinary innovation in areas including materials science and pharmaceuticals.

## Teaser

Chain-walking borylation targets indistinguishable methyl groups, creating “Y-shape” molecules with record-breaking reach.

## INTRODUCTION

C─H functionalization strategies have emerged as a powerful and highly attractive approach in organic synthesis, offering the ability to precisely modify carbon atoms at arbitrary positions (*1*). By cleaving the most ubiquitous C─H bonds, they enable targeted transformations along a molecular backbone with remarkable simplicity (*2*–*5*). However, achieving site-selective modification of one carbon atom without disturbing nearly identical sites remains an exceptionally daunting challenge (*6*).

**Fig. 1.**
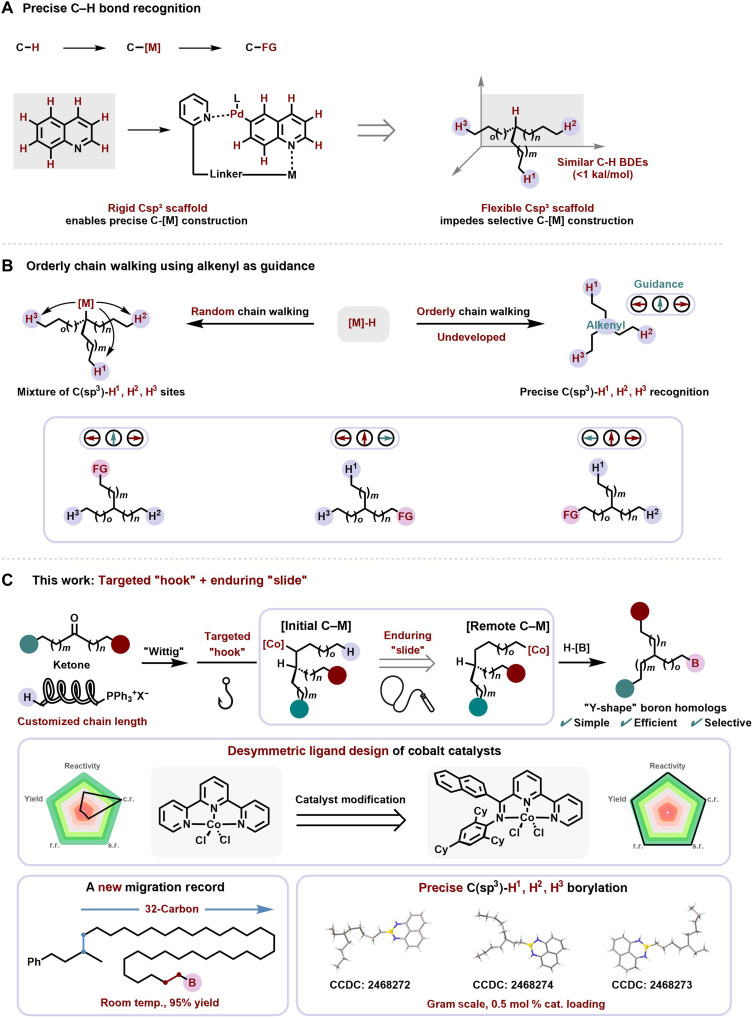
Precise functionalization of C(*sp^3^*)-H bonds via an orderly chain-walking strategy. (**A**) Precise C─H bond recognition. (**B**) Orderly chain walking using alkenyl as guidance. (**C**) This work: Targeted “hook” + enduring “slide.”

Advances in organometallic chemistry have made it possible to convert C─H bonds into carbon-metal intermediates (*7*), which can be readily transformed into diverse functional groups, thus enabling C─H functionalization (Fig. 1A). Through specific directing-group effects, rigid C*sp^2^* scaffolds enable precise recognition and functionalization of multiple remote C─H sites, providing a direct approach to targeting C─H bond identification (*1*, *8*–*12*). By contrast, flexible C*sp^3^* scaffolds, devoid of directing groups, present substantially greater challenges in achieving site-selective functionalization: Their higher C─H bond dissociation energies (BDEs) (*13*) and the presence of multiple similar primary C─H sites hinder selective carbon-metal bond formation (*14*, *15*), and even radical strategies under mild conditions cannot yet achieve precise control (Fig. 1A) (*16*, *17*).

To circumvent these limitations, a chain-walking approach was introduced which involves first installing a carbon-metal bond at the starting site, followed by metal hydride–mediated chain migration (*18*–*22*). Although it has been applied to the functionalization of specific sites, especially terminal positions, precise control is still quite difficult in the presence of multiple similar C─H sites (*23*–*25*). It was reasoned that precise recognition of closely related C─H sites could be achieved by controlling the insertion direction of a metal hydride species via differentiation between 1,2- and 2,1-insertion (Fig. 1B). Here, an orderly chain-walking borylation strategy (the hydrometalation of a trisubstituted double bond will inherently favor addition of transition metal to the less-substituted carbon due to the catalyst modification. This initial structural bias predetermines the migration direction, resulting in a single, predictable pathway) was proposed through the Wittig reaction (*26*) to generate defined alkenyl orientation, then a precise hook (*27*) directs the metal hydride to form the initial carbon-metal species, followed by a slide that generates remote carbon-metal intermediates, with terminal termination regenerating the active species (Fig. 1C) (*28*).

It was found that the resulting boron homologs, termed the “Y-shape” (*29*), which was used here descriptively for three alkyl chains attached to a single carbon, enabled versatile control over solubility, crystallinity, and molecular packing, thereby influencing device performance in organic photovoltaics, printed electronics, stretchable transistors, and carrier-free nanoassembly systems (*30*–*35*). Our group has long been dedicated to the design of unsymmetric tridentate ligands (*36*). By using a new class of desymmetric ligand with cobalt catalyst, 32 consecutive chain migrations at room temperature were achieved with high activity and selectivity. Moreover, by altering the orientation of the trisubstituted alkene through three simple Wittig reactions, gram-scale precise C(*sp^3^*)-H^1^, H^2^, and H^3^ borylation can be independently enabled at catalyst loadings as low as 0.5 mol %. This strategy exemplifies a powerful platform for C─H functionalization and is expected to inspire cross-disciplinary advances in materials science and beyond (Fig. 1C).

## RESULTS

### Reaction optimization

**Fig. 2.**
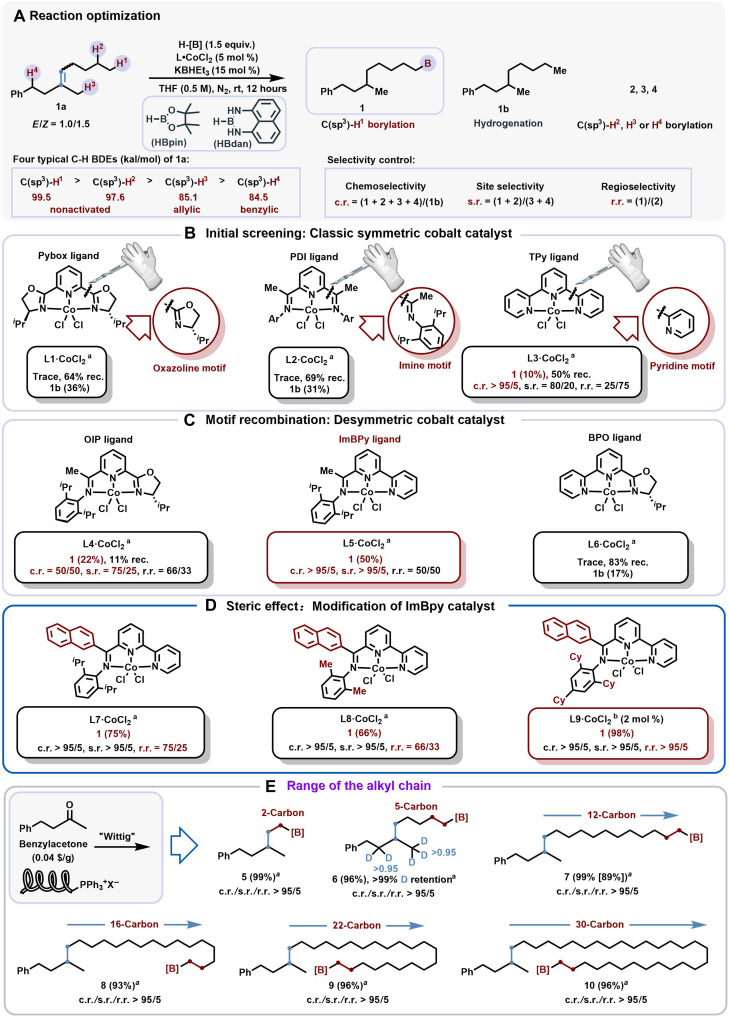
Reaction optimization. (**A**) Reaction optimization. rt, room temperature. (**B**) Initial screening: Classic symmetric cobalt catalyst. (**C**) Motif recombination: Desymmetric cobalt catalyst. (**D**) Steric effect: Modification of ImBpy catalyst. (**E**) Range of the alkyl chain. To assess the selectivity of the reaction, the alkyl borane compound was oxidized to the corresponding alcohol. Yields were determined by ^1^H NMR using phenyltrimethylsilane as an internal standard. ^a^Reaction was performed on 0.25 mmol scale in a glove box; ^b^HBdan instead of HBpin.

Using a commercially available and inexpensive benzylacetone, the nonactivated *E*/*Z*-mixed trisubstituted alkene **1a** (*E*/*Z* = 1.0/1.5) was efficiently prepared on gram scale via a one-step Wittig reaction in quantitative yield (see in fig. S4 for details). Four typical C─H BDEs were predicted on the basis of a machine learning approach (*37*). This straightforward route allowed us to initiate our study with **1a** in the presence of a cobalt precatalyst, HBpin (pinacolborane), and tetrahydrofuran (THF; Fig. 2A). Upon activation with KBHEt_3_ (1.0 M in THF) (*38*), in recognition of Chirik’s notable contributions (*25*, *39*) to this field, three classic symmetric ligands were initially screened (Fig. 2B). Regrettably, while the Pybox (pyridine bisoxazoline) **L1** and PDI (pyridine diamine) **L2** exhibited moderate reactivity, they failed to produce the desired Y-shape product **1**, yielding only the hydrogenation byproducts **1b**. Encouragingly, the introduction of a TPy [tris(pyridyl)] **L3** cobalt catalyst resulted in a 10% yield of product **1**, with the catalyst demonstrating excellent chemoselectivity ratio (>95/5), effectively suppressing the formation of **1b**. However, the s.r. (site selectivity ratio) and r.r. (regioselectivity ratio) were suboptimal, at 80/20 and 25/75, respectively.

Given the symmetrical nature of classic ligands, the three ligands were accordingly dissected into their constituent oxazoline, imine, and pyridine motifs (Fig. 2B). We hypothesized that by using a motif recombination approach, the distinct roles of each module could be exploited on a single ligand to enhance both reaction activity and further optimize the site and regioselectivity. This led to the design of three desymmetric cobalt catalysts (Fig. 2C). Our previous work on the OIP (oxazoline-imine-pyridine) ligand **L4** (*38*) showed improved reactivity, with product **1** reaching 22%, although the chemo, site, and regioselectivity remained unsatisfactory. The BPO [bis(pyridyl)oxazoline] ligand **L6** (*40*) yielded only 17% of **1b**. Excitingly, the ImBPy [imine-bis(pyridine)] ligand **L5** (*41*) resulted in a 50% yield of **1**, with both chemoselectivity ratio and s.r. > 95/5. This result validated the unique advantages of the desymmetric ligands and motivated further efforts to improve the regioselectivity.

Then, ImBPy ligand skeleton was used as a template for further ligand modification (Fig. 2D). Upon introducing the previously identified persistent carbon-centered radical effect (*38*) at the shoulder position of the imine side, the r.r. of the ligand **L7** was increased from 50/50 to 75/25. Through extensive ligand optimization (see in fig. S4 for details), the steric hindrance of substituents on the imine phenyl ring was identified as the key factor limiting further improvement of the r.r. Using a small steric hindrance substituent ligand **L8**, the r.r. decreased from 75/25 to 66/33. Last, when using the highly sterically hindered 2,4,6-tri(cyclohexyl) ligand (**L9**) with 2 mol % of bulky substituent and using HBdan (1,8-diaminonapthalene borane) as the boron source, all selectivity ratios exceeded >95/5, with an 98% yield of isolated product.

### Substrate scope

**Fig. 3.**
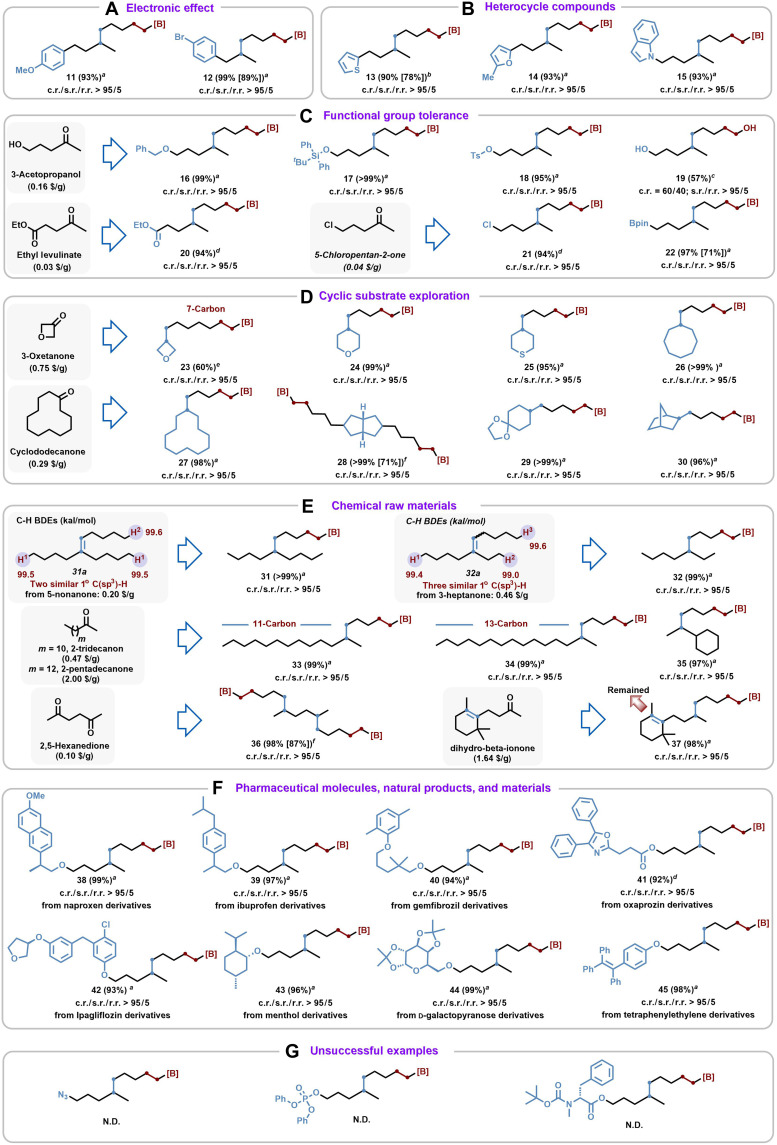
Substrate scope. (**A**) Electronic effect. (**B**) Heterocycle compounds. (**C**) Functional group tolerance. (**D**) Cyclic substrate exploration. (**E**) Chemical raw materials. (**F**) Pharmaceutical molecules, natural products, and Materials. (**G**) Unsuccessful examples. The ketones were commercially available. ^a^Standard conditions: **L9·**CoCl_2_ (2 mol %), HBdan (1.5 equiv.), alkene (0.25 mmol), THF (0.5 ml), and KBHEt_3_ (6 mol %), glove box, rt., 12 hours. ^b^**L9·**CoCl_2_ (4 mol %); ^c^oxidized to the corresponding alcohol; ^d^added the alkene last; ^e^30% of the ring-opened product; ^f^**L9·**CoCl_2_ (8 mol %). N.D., not detected.

After optimizing the reaction conditions, the substrate scope was extended. First, to assess the robustness of the orderly chain-walking process, mixed trisubstituted alkenes were synthesized (**1a** to **10a**) with carbon chain lengths ranging from **2** to **30** through a simple Wittig reaction. Under standard conditions, increasing the carbon chain length had no significant effect on either reaction activity or selectivity, demonstrating the superiority of the catalytic system (Fig. 2E). Notably, when using trisubstituted alkene **6a** bearing five deuterium atoms, >99% deuterium retention was observed in the corresponding product **6**. This result indicates a highly specific alkene insertion process, in sharp contrast to the extensive deuterium scrambling observed in our previous studies (*38*). Such selectivity highlights the unique advantages conferred by the desymmetric ligand design. The effect of electronic changes was then examined on the aryl group (Fig. 3A), and we found that there was no noticeable substituent effect on the reaction (**11** to **12**). Heterocycles were also well tolerated, with reactions involving thiophene (**13**), pyrrole (**14**), and *N*-indole (**15**) groups showing good selectivity (Fig. 3B).

Starting from low-cost and readily available ketones (see in fig. S3 for details)—including 5-hydroxy-2-pentanone (0.16 $/g), ethyl levulinate (0.03 $/g), and 5-chloropentan-2-one (0.04 $/g)—a diverse set of trisubstituted alkenes was synthesized (**16a** to **22a**) to assess the robustness and functional group tolerance of the molecular editing protocol (Fig. 3C). A wide range of functional groups—including benzyloxy (**16**), silyloxy (**17**), pseudo-halogen (OTs) (**18**), free hydroxyl (**19**), ester (**20**), halogen (**21**), and pinacol boronate esters (**22**)—were all well tolerated, consistently delivering the desired products in good yields.

Cyclic compounds, owing to their intrinsic ring strain and conformational rigidity, represent a unique class of substrates with broad interest in synthetic chemistry, the ability to retain intact ring systems during functionalization is important (*42*), especially for applications in complex molecule synthesis and material design. In this context, the applicability of orderly chain-walking borylation protocol was evaluated to a series of cyclic trisubstituted alkenes (Fig. 3D). No isomerization from exocyclic to thermodynamically favored endocyclic alkenes was observed (*43*), including strained four-membered rings (**23a**), heterocycles containing **O**, **S** atoms (**23a** to **25a**), medium-sized rings (**26a**), and macrocyclic rings (**27a**). Moreover, functionally rich and structurally complex motifs—such as bicyclic system (**28a**), spiro (**29a**), and bridged ring (**30a**)—were all successfully transformed.

The most widely used boron-containing Y-shape analogs to date feature purely alkyl side chains (Fig. 3E). To expand their structural diversity, trisubstituted alkenes were constructed bearing two (**31a**) and three (**32a**) C─H bonds with nearly identical BDEs from simple alkyl ketone precursors. Such closely matched BDEs (*37*) typically preclude site selectivity in conventional C─H activation, yet this strategy enabled precise selective borylation, affording **31** and **32** in 99% yield. The scalability and functional group tolerance were further demonstrated by using natural ketones to generate variable chain lengths (e.g., **33a** and **34a**) and secondary alkyl substitution (**35a**), all of which were converted smoothly. Notably, the industrial feedstock 2,5-hexanedione was used to synthesize a bis-alkene precursor (**36a**), which underwent efficient diborylation. Moreover, the complex substrate **37a**, derived from dihydro-β-ionone and bearing both trisubstituted and tetrasubstituted alkenes, exhibited excellent selectivity, retaining the more hindered tetrasubstituted alkene and furnishing the desired product **37** in 98% yield.

To further validate the practical application of this reaction (Fig. 3F), pharmaceutical derivatives such as naproxen (**38**), ibuprofen (**39**), gemfibrozil (**40**), oxaprozin (**41**), and ipagliflozin (**42**), as well as natural products menthol (**43**), *D*-galactopyranoseand (**44**), and luminescent material tetraphenylethylene (**45**), were introduced, all of which reacted smoothly under standard conditions. Unfortunately, the reaction failed to proceed efficiently when the substrate contained functional groups such as azide, phosphatidyl, and amide.

### Synthetic application

**Fig. 4.**
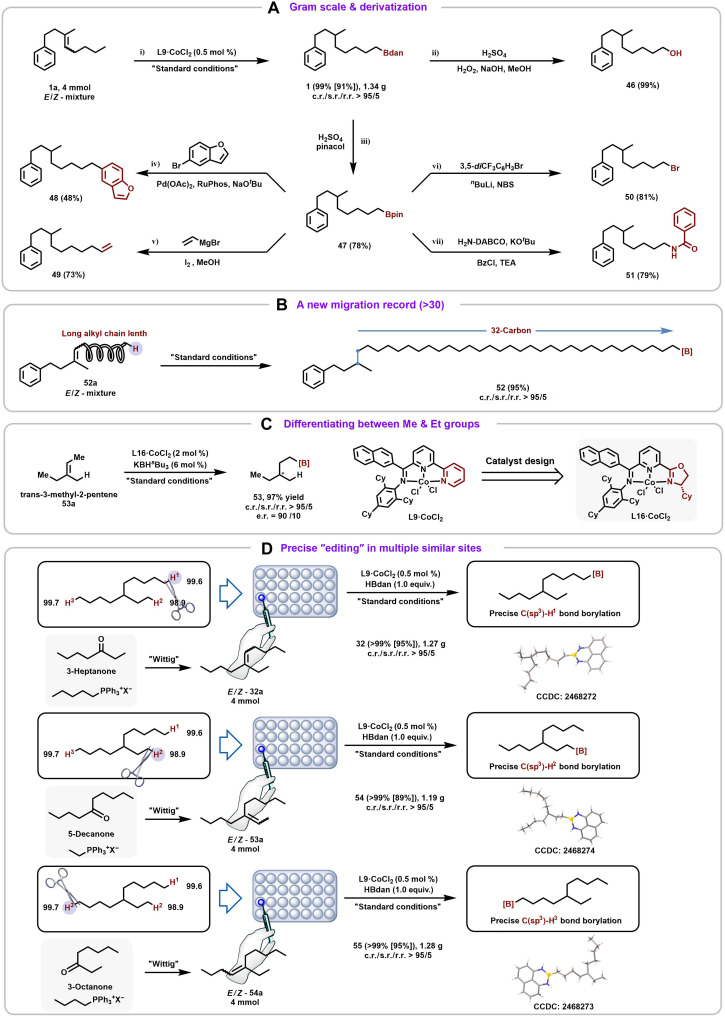
Synthetic application. (**A**) Gram scale and derivatization. (**B**) Migration distance of 32 carbons. (**C**) Differentiating between Me and Et groups. (**D**) Precise “editing” in multiple similar sites.

The representative Y-shape boron analog **1** was readily scaled up to gram quantities (1.35 g, 91% yield) using only 0.5 mol % catalyst, demonstrating the operational simplicity and scalability of this method. **1** could be smoothly transformed into alcohol **46**. The boron protecting group could also be selectively modified—e.g., replacing with a pinacol ester to give **47**. Leveraging the versatility of organoboron intermediates, **47** could be converted into diverse functionalities, including *sp^2^*-carbon heterocycles **48**, alkenes **49**, halides **50**, and amines **51** (*44*), highlighting its broad synthetic utility (Fig. 4A).

To probe the upper limit of chain walking in this system, substrate **52a** was designed bearing a 32-carbon alkyl chain, which can be readily synthesized via a simple Wittig reaction. Notably, further extension of the carbon chain leads to a sharp decrease in the solubility of the halide precursor. Under standard conditions at room temperature with 2 mol % catalyst loading, **52a** was efficiently converted to product **52** in 95% yield, with all selectivity parameters exceeding 95/5. To the best of our knowledge, this represents the highest record of consecutive chain-walking events reported to date (Fig. 4B) (*45*).

To further explore enantioselective control, a ligand modification strategy was used to address the highly challenging task of two minimally different alkyl substituent discrimination (methyl versus ethyl) (Fig. 4C) (*46*, *47*). Using **L16**•CoCl_2_ catalyst, the fully aliphatic, directing-group–free commercial substrate **53a** was transformed into product **53** in 97% yield, with chemo-, site-, and regioselectivities all exceeding 95/5 and an enantiomeric ratio of 90/10.

Notably, the directional nature of olefins enables precise editing of C(*sp^3^*)-H bonds with comparable BDEs. As illustrated, substrate C(*sp^3^*)-H bonds H^1^, H^2^, and H^3^ exhibit BDEs of 99.6, 98.9, and 99.7 kcal/mol (*37*), respectively, yet selective activation was achieved simply by choosing appropriate olefin precursors (e.g., 3-heptanone, 5-nonanone, and 3-octanone). Reactions proceeded cleanly on gram scale with only 0.5 mol % catalyst and 1:1 HBdan (Fig. 4D). It is worth noting that for these three types of compounds, unambiguously correlating the site of C(*sp^3^*)-H functionalization with nuclear magnetic resonance (NMR) data is challenging, and all products are obtained as liquids.

To overcome this limitation, the supramolecular docking techniques recently reported by Huang group (*48*) was used to crystallize oily and flexible alkyl chain–containing molecules. The single-crystal structures of products **32**, **54**, and **55** were successfully obtained using this method, thereby confirming the feasibility of precise editing at similar C(*sp^3^*)-H sites.

### Mechanistic experiments

**Fig. 5.**
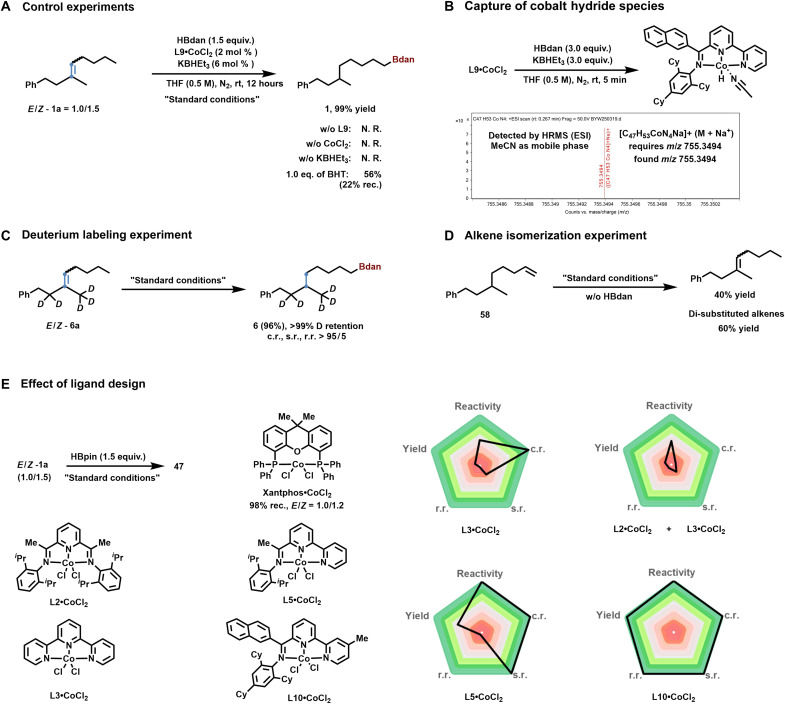
Mechanistic experiments. (**A**) Control experiments. (**B**) Capture of cobalt hydride species. (**C**) Deuterium labeling experiment. (**D**) Alkene isomerization experiment. (**E**) Effect of ligand design. ESI, electrospray ionization; MeCN, acetonitrile; N.R., no reaction; HRMS, high-resolution mass spectrometry.

To gain insight into the reaction mechanism, a series of mechanistic investigations was conducted (Fig. 5). Control experiments showed that removing the ligand, cobalt precursor, or additives completely suppressed product formation, indicating no background reactivity. The addition of 1.0 equivalent of the radical scavenger butylated hydroxytoluene had no effect on the outcome, effectively excluding a radical-mediated pathway (Fig. 5A). Moreover, high-resolution quadrupole time-of-flight analysis of the reaction mixture in the absence of alkene revealed a strong signal corresponding to a cobalt hydride species, suggesting that the catalytic cycle proceeds via a metal hydride intermediate (Fig. 5B).

Furthermore, deuterium-labeling experiments revealed a single mode of metal hydride insertion, specifically favoring the 2,1-insertion over the 1,2-insertion pathway (Fig. 5C). As a result, the deuterium content on the substrate remained unchanged. If a 1,2-insertion pathway were operative, deuterium redistribution would have been observed (*38*). In addition, an olefin isomerization experiment was conducted using terminal alkene **58** as the substrate (Fig. 5D). In the absence of borane, both trisubstituted and disubstituted alkenes were obtained. Given that olefin isomerization is a reversible process, these results support a reaction mechanism involving metal hydride–mediated olefin isomerization followed by hydroboration.

Literature reports indicate that the common ligand Xantphos can promote Co-catalyzed olefin isomerization (Fig. 5E) (*49*). Using **1a** as the substrate, full recovery of starting material was observed with an altered *E*/*Z* ratio, suggesting that metal hydride insertion into the olefin had occurred. However, no product formation was detected, indicating that the phosphine ligand alone is insufficient to drive the reaction.

To further evaluate the unique benefits of the desymmetric ligand design, competition experiments were conducted using two conventional symmetric tridentate ligands, PDI (pyridine diimine) and Tpy (terpyridine) (Fig. 5E). Notably, their coexistence disrupted the reaction, leading to a complete loss of the chemoselectivity typically imparted by Tpy. In contrast, the desymmetric ligand ImBpy—constructed through structural dissection and recombination of Tpy—enabled simultaneous control over reactivity and selectivity. These results highlight the advantages of desymmetric ligand design, wherein the enhanced reactivity cannot be reproduced by a simple combination of two symmetric ligand frameworks.

On the basis of these findings and literature precedents, a mechanistic pathway was proposed in which the cobalt hydride species undergoes alkene insertion followed by rapid chain walking along the alkyl backbone (see in fig. S13 for details). The involvement of a persistent radical effect (*38*) likely prolongs the lifetime of the active species, enabling efficient migration. Final σ-bond metathesis between the terminal alkyl metal intermediate and HBdan completes the catalytic cycle and regenerates the active hydride complex.

## DISCUSSION

In summary, an orderly chain-walking borylation strategy has been developed for the synthesis of boron-containing Y-shape analogs enabled by a rationally designed desymmetric ligand with cobalt catalyst. This method features broad substrate scope, good functional group tolerance, and high atom economy. We hope that this user-friendly approach can spark ideas in materials and pharmaceutical sciences and contribute to the development of practical tools for broader scientific communities.

## MATERIALS AND METHODS

### General synthetic procedure of *E*/*Z* mixed trisubstituted alkenes

Potassium tert-butoxide (24 mmol, 1.2 equiv) was added to a solution of alkyl triphenylphosphonium bromide (24 mmol, 1.2 equiv) in anhydrous THF (80 ml) at 0°C. The reaction mixture was stirred for 0.5 hours at room temperature. The corresponding alkyl ketones (20 mmol, 1.0 equiv) were then dissolved in THF solution and slowly added to the above mixture. The reaction mixture was stirred for 12 hours at room temperature. The crude product was isolated through the evaporation of volatiles in vacuo, and then the resulting mixture was purified by flash column chromatography on silica gel using petroleum ether/ethyl acetate (PE/EA) as the eluent to give the corresponding *E*/*Z* mixed trisubstituted alkenes.

### General synthetic procedure of HBdan

BH_3_⋅SMe_2_ (5.0 ml, 50 mmol, equiv) was added dropwise to a solution of 1,8-diaminonaphthalene (7.91 g, 50 mmol, 1.0 equiv) in anhydrous dichloromethane (25 ml) at 0°C for more than 30 min. The reaction mixture was stirred for 48 hours at room temperature. The crude product was isolated through the evaporation of volatiles in vacuo, and then the resulting mixture was purified by flash column chromatography on silica gel using [PE/EA = 100/3 (v/v)] as the eluent to give HBdan as a white solid.

### General synthetic procedure of Y-shape boron analogs

In the nitrogen-filled glove box, a 10-ml flame-dried flask was cooled at room temperature under nitrogen, charged with **L9**•CoCl_2_ (0.0038 g, 0.005 mmol, 0.02 equiv), HBdan (0.0630 g, 0.375 mmol, 1.5 equiv), and then alkene (0.25 mmol, 1.0 equiv), anhydrous THF (0.5 ml, 0.5 M), then KBHEt_3_ (15 μl, 1 M in THF, 0.015 mmol, 0.06 equiv) were added successively. The mixture was kept under room temperature and stirred for 12 hours. The reaction was quenched by PE. The mixture was filtered through a pad of silica gel and washed with ether (50 ml). NMR yield was monitored by ^1^H NMR analysis using phenyltrimethylsilane as internal standard. The resulting mixture was purified by flash column chromatography on silica gel using PE/EA/TEA as the eluent to give the corresponding Y-shape boron analogs.

## Supplementary Materials

### This PDF file includes:

Supplementary Text Figs. S1 to S13 References

## References

[1] Z. Fan, X. Chen, K. Tanaka, H. S. Park, N. Y. S. Lam, J. J. Wong, K. N. Houk, J.-Q. Yu, Molecular editing of aza-arene C–H bonds by distance, geometry and chirality. Nature 610, 87–93 (2022). 35944562 10.1038/s41586-022-05175-1 PMC10292866

[2] S. Das, C. D. Incarvito, R. H. Crabtree, G. W. Brudvig, Molecular recognition in the selective oxygenation of saturated C─H bonds by a dimanganese catalyst. Science 312, 1941–1943 (2006). 16809537 10.1126/science.1127899

[3] J. Wencel-Delord, F. Glorius, C–H bond activation enables the rapid construction and late-stage diversification of functional molecules. Nat. Chem. 5, 369–375 (2013). 23609086 10.1038/nchem.1607

[4] B. Prabagar, Y. Yang, Z. Shi, Site-selective C–H functionalization to access the arene backbone of indoles and quinolines. Chem. Soc. Rev. 50, 11249–11269 (2021). 34486584 10.1039/d0cs00334d

[5] H. Shi, Y. Lu, J. Weng, K. L. Bay, X. Chen, K. Tanaka, P. Verma, K. N. Houk, J.-Q. Yu, Differentiation and functionalization of remote C–H bonds in adjacent positions. Nat. Chem. 12, 399–404 (2020). 32123338 10.1038/s41557-020-0424-5 PMC7155936

[6] B. M. Trost, Selectivity: A key to synthetic efficiency. Science 219, 245–250 (1983). 17798254 10.1126/science.219.4582.245

[7] Y.-J. Liu, H. Xu, W.-J. Kong, M. Shang, H.-X. Dai, J.-Q. Yu, Overcoming the limitations of directed C–H functionalizations of heterocycles. Nature 515, 389–393 (2014). 25383516 10.1038/nature13885 PMC4248606

[8] D.-H. Wang, K. M. Engle, B.-F. Shi, J.-Q. Yu, Ligand-enabled reactivity and selectivity in a synthetically versatile aryl C–H olefination. Science 327, 315–319 (2010). 19965380 10.1126/science.1182512 PMC2879878

[9] D. Leow, G. Li, T.-S. Mei, J.-Q. Yu, Activation of remote meta-C–H bonds assisted by an end-on template. Nature 486, 518–522 (2012). 22739317 10.1038/nature11158 PMC3386562

[10] R.-Y. Tang, G. Li, J.-Q. Yu, Conformation-induced remote meta-C–H activation of amines. Nature 507, 215–220 (2014). 24622200 10.1038/nature12963 PMC3980735

[11] X.-C. Wang, W. Gong, L.-Z. Fang, R.-Y. Zhu, S. Li, K. M. Engle, J.-Q. Yu, Ligand-enabled meta-C–H activation using a transient mediator. Nature 519, 334–338 (2015). 25754328 10.1038/nature14214 PMC4368492

[12] Z. Zhang, K. Tanaka, J.-Q. Yu, Remote site-selective C–H activation directed by a catalytic bifunctional template. Nature 543, 538–542 (2017). 28273068 10.1038/nature21418 PMC5477648

[13] H. Chen, S. Schlecht, T. C. Semple, J. F. Hartwig, Thermal, catalytic, regiospecific functionalization of alkanes. Science 287, 1995–1997 (2000). 10720320 10.1126/science.287.5460.1995

[14] K. Liao, Y.-F. Yang, Y. Li, J. N. Sanders, K. N. Houk, D. G. Musaev, H. M. L. Davies, Design of catalysts for site-selective and enantioselective functionalization of non-activated primary C–H bonds. Nat. Chem. 10, 1048–1055 (2018). 30082883 10.1038/s41557-018-0087-7 PMC6650386

[15] R. Oeschger, B. Su, I. Yu, C. Ehinger, E. Romero, S. He, J. Hartwig, Diverse functionalization of strong alkyl C–H bonds by undirected borylation. Science 368, 736–741 (2020). 32409470 10.1126/science.aba6146 PMC7710342

[16] C. Shu, A. Noble, V. K. Aggarwal, Metal-free photoinduced C(*sp^3^*)–H borylation of alkanes. Nature 586, 714–719 (2020). 33116286 10.1038/s41586-020-2831-6

[17] M. Wang, Y. Huang, P. Hu, Terminal C(*sp^3^*)–H borylation through intermolecular radical sampling. Science 383, 537–544 (2024). 38300993 10.1126/science.adj9258

[18] A. Vasseur, J. Bruffaerts, I. Marek, Remote functionalization through alkene isomerization. Nat. Chem. 8, 209–219 (2016). 26892551 10.1038/nchem.2445

[19] H. Sommer, F. Juliá-Hernández, R. Martin, I. Marek, Walking metals for remote functionalization. ACS Cent. Sci. 4, 153–165 (2018). 29532015 10.1021/acscentsci.8b00005 PMC5833012

[20] C. Romano, R. Martin, Ni-catalysed remote C(*sp^3^*)–H functionalization using chain-walking strategies. Nat. Rev. Chem. 8, 833–850 (2024). 39354168 10.1038/s41570-024-00649-4

[21] J. Rodrigalvarez, F.-L. Haut, R. Martin, Regiodivergent sp^3^ C–H functionalization via Ni-catalyzed chain-walking reactions. JACS Au 3, 3270–3282 (2023). 38155646 10.1021/jacsau.3c00617 PMC10751781

[22] D. Janssen-Müller, B. Sahoo, S.-Z. Sun, R. Martin, Tackling remote sp^3^ C−H functionalization via Ni-catalyzed “chain-walking” reactions. Isr. J. Chem. 60, 195–206 (2020).

[23] Q. Wang, J. Kweon, D. Kim, S. Chang, Remote catalytic C(sp^3^)–H alkylation via relayed carbenoid transfer upon olefin chain walking. J. Am. Chem. Soc. 146, 31114–31123 (2024). 39475225 10.1021/jacs.4c11014

[24] C. Hou, Z. Liu, L. Gan, W. Fan, L. Huang, P. Chen, Z. Huang, G. Liu, Palladium-catalyzed remote hydrosulfonamidation of alkenes: Access to primary N-alkyl sulfamides by the SuFEx reaction. J. Am. Chem. Soc. 146, 13536–13545 (2024). 38693624 10.1021/jacs.4c03283

[25] W. N. Palmer, T. Diao, I. Pappas, P. J. Chirik, High-activity cobalt catalysts for alkene hydroboration with electronically responsive terpyridine and α-diimine ligands. ACS Catal. 5, 622–626 (2015).

[26] G. Wittig, U. Schöllkopf, Über triphenyl-phosphin-methylene als olefinbildende reagenzien (I. Mitteil). Chem. Ber. 87, 1318–1330 (1954).

[27] R. Zhang, T. Yu, G. Dong, Rhodium catalyzed tunable amide homologation through a hook-and-slide strategy. Science 382, 951–957 (2023). 37995236 10.1126/science.adk1001 PMC11102777

[28] J. V. Obligacion, P. J. Chirik, Earth-abundant transition metal catalysts for alkene hydrosilylation and hydroboration. Nat. Rev. Chem. 2, 15–34 (2018). 30740530 10.1038/s41570-018-0001-2 PMC6365001

[29] Y. Yang, The original design principles of the Y-series nonfullerene acceptors, from Y1 to Y6. ACS Nano 15, 18679–18682 (2021). 34854305 10.1021/acsnano.1c10365

[30] G. Yu, J. Gao, J. C. Hummelen, F. Wudl, A. J. Heeger, Polymer photovoltaic cells: Enhanced efficiencies via a network of internal donor-acceptor heterojunctions. Science 270, 1789–1791 (1995).

[31] M. Granström, K. Petritsch, A. C. Arias, A. Lux, M. R. Andersson, R. H. Friend, Laminated fabrication of polymeric photovoltaic diodes. Nature 395, 257–260 (1998).

[32] H. Yan, Z. Chen, Y. Zheng, C. Newman, J. R. Quinn, F. Dötz, M. Kastler, A. Facchetti, A high-mobility electron-transporting polymer for printed transistors. Nature 457, 679–686 (2009). 19158674 10.1038/nature07727

[33] J. Y. Oh, S. Rondeau-Gagné, Y.-C. Chiu, A. Chortos, F. Lissel, G.-J. N. Wang, B. C. Schroeder, T. Kurosawa, J. Lopez, T. Katsumata, J. Xu, C. Zhu, X. Gu, W.-G. Bae, Y. Kim, L. Jin, J. W. Chung, J. B. H. Tok, Z. Bao, Intrinsically stretchable and healable semiconducting polymer for organic transistors. Nature 539, 411–415 (2016). 27853213 10.1038/nature20102

[34] Y.-Q. Zheng, Y. Liu, D. Zhong, S. Nikzad, S. Liu, Z. Yu, D. Liu, H.-C. Wu, C. Zhu, J. Li, H. Tran, J. B.-H. Tok, Z. Bao, Monolithic optical microlithography of high-density elastic circuits. Science 373, 88–94 (2021). 34210882 10.1126/science.abh3551

[35] S. Wang, T. Liu, Y. Huang, C. Du, D. Wang, X. Wang, Q. Lv, Z. He, Y. Zhai, B. Sun, J. Sun, The effect of lengths of branched-chain fatty alcohols on the efficacy and safety of docetaxel-prodrug nanoassemblies. Acta Pharm. Sin. B 14, 1400–1411 (2024). 38486988 10.1016/j.apsb.2023.09.017 PMC10934334

[36] J. Guo, Z. Cheng, J. Chen, X. Chen, Z. Lu, Iron- and cobalt-catalyzed asymmetric hydrofunctionalization of alkenes and alkynes. Acc. Chem. Res. 54, 2701–2716 (2021). 34011145 10.1021/acs.accounts.1c00212

[37] Y. Liu, Y. Li, Q. Yang, J.-D. Yang, L. Zhang, S. Luo, Prediction of bond dissociation energy for organic molecules based on a machine-learning approach. Chin. J. Chem. 42, 1967–1974 (2024).

[38] Y. Bao, C. Zheng, K. Xiong, C. Hu, P. Lu, Y. Wang, Z. Lu, Enantioconvergent hydroboration of E/Z-mixed trisubstituted alkenes. J. Am. Chem. Soc. 146, 21089–21098 (2024). 38994866 10.1021/jacs.4c06585

[39] J. V. Obligacion, P. J. Chirik, Bis(imino)pyridine cobalt-catalyzed alkene isomerization–hydroboration: A strategy for remote hydrofunctionalization with terminal selectivity. J. Am. Chem. Soc. 135, 19107–19110 (2013). 24328236 10.1021/ja4108148

[40] J. Peng, J. H. Docherty, A. P. Dominey, S. P. Thomas, Cobalt-catalysed Markovnikov selective hydroboration of vinylarenes. Chem. Commun. 53, 4726–4729 (2017). 10.1039/c7cc01085k 28401978

[41] C. Bianchini, D. Gatteschi, G. Giambastiani, I. Guerrero Rios, A. Ienco, F. Laschi, C. Mealli, A. Meli, L. Sorace, A. Toti, F. Vizza, Electronic influence of the thienyl sulfur atom on the oligomerization of ethylene by cobalt(II) 6-(Thienyl)-2-(imino)pyridine catalysis. Organometallics 26, 726–739 (2007).

[42] Y. Li, Y. Li, H. Shi, H. Wei, H. Li, I. Funes-Ardoiz, G. Yin, Modular access to substituted cyclohexanes with kinetic stereocontrol. Science 376, 749–753 (2022). 35549424 10.1126/science.abn9124

[43] X. Liu, W. Zhang, Y. Wang, Z.-X. Zhang, L. Jiao, Q. Liu, Cobalt-catalyzed regioselective olefin isomerization under kinetic control. J. Am. Chem. Soc. 140, 6873–6882 (2018). 29781616 10.1021/jacs.8b01815

[44] X. Liu, Q. Zhu, D. Chen, L. Wang, L. Jin, C. Liu, Aminoazanium of DABCO: An amination reagent for alkyl and aryl pinacol boronates. Angew. Chem. Int. Ed. 59, 2745–2749 (2020). 10.1002/anie.201913388 31814182

[45] L. Lin, C. Romano, C. Mazet, Palladium-catalyzed long-range deconjugative isomerization of highly substituted α,β-unsaturated carbonyl compounds. J. Am. Chem. Soc. 138, 10344–10350 (2016). 27434728 10.1021/jacs.6b06390

[46] M. Wang, S. Liu, H. Liu, Y. Wang, Y. Lan, Q. Liu, Asymmetric hydrogenation of ketimines with minimally different alkyl groups. Nature 631, 556–562 (2024). 38806060 10.1038/s41586-024-07581-z

[47] Y.-F. Zhang, B. Wang, Z. Chen, J.-R. Liu, N.-Y. Yang, J.-M. Xiang, J. Liu, Q.-S. Gu, X. Hong, X.-Y. Liu, Asymmetric amination of alkyl radicals with two minimally different alkyl substituents. Science 388, 283–291 (2025). 40245132 10.1126/science.adu3996

[48] Y. Wu, L. Shi, L. Xu, J. Ying, X. Miao, B. Hua, Z. Chen, J. L. Sessler, F. Huang, Supramolecular docking structure determination of alkyl-bearing molecules. Nature 640, 676–682 (2025). 40205040 10.1038/s41586-025-08833-2 PMC12003189

[49] Y. Zhao, S. Ge, Synergistic hydrocobaltation and borylcobaltation enable regioselective migratory triborylation of unactivated alkenes. Angew. Chem. Int. Ed. 61, e202116133 (2022). 10.1002/anie.202116133 35088939

[50] T. T. Nguyen, M. J. Koh, T. J. Mann, R. R. Schrock, A. H. Hoveyda, Synthesis of E- and Z-trisubstituted alkenes by catalytic cross-metathesis. Nature 552, 347–354 (2017). 29293209 10.1038/nature25002 PMC5967255

[51] H. Kim, S. Y. Choi, S. Shin, Asymmetric synthesis of dihydropyranones *via* gold(I)-catalyzed intermolecular [4+2] annulation of propiolates and alkenes. Angew. Chem. Int. Ed. 57, 13130–13134 (2018). 10.1002/anie.201807514 30129705

[52] H. Li, Z. Lai, M. Peng, L. Ning, Q. Dong, Y. Hou, J. An, One-pot sequential hydrogen isotope exchange/reductive deuteration for the preparation of α,β-deuterated alcohols using deuterium oxide. Org. Lett. 24, 5319–5323 (2022). 35856804 10.1021/acs.orglett.2c01940

[53] D. Das, G. Sahoo, A. Biswas, R. Samanta, Rh(III)-catalyzed synthesis of highly substituted 2-pyridones using fluorinated diazomalonate. Chem. Asian. J. 15, 360–364 (2020). 31944607 10.1002/asia.201901620

[54] Z. Zheng, L. Chen, C. Qian, X. Zhu, Y. Yang, J. Liu, Y. Yang, Y. Liang, Copper-catalyzed synthesis of 2-acylbenzo[b]thiophenes from 3-(2-iodophenyl)-1-arylpropan-1-ones and potassium sulfide under aerobic conditions. Org. Biomol. Chem. 16, 8020–8024 (2018). 30334048 10.1039/c8ob02315h

[55] Q. Xia, X. Bao, C. Sun, D. Wu, X. Rong, Z. Liu, Y. Gu, J. Zhou, G. Liang, Design, synthesis and biological evaluation of novel 2-sulfonylindoles as potential anti-inflammatory therapeutic agents for treatment of acute lung injury. Eur. J. Med. Chem. 160, 120–132 (2018). 30326372 10.1016/j.ejmech.2018.10.014

[56] J.-P. Fontaine, V. Lapointe, M. Filliâtre, G. Bélanger, Synthesis of substituted indolines through photocatalyzed decarboxylative radical arylation. J. Org. Chem. 88, 6557–6564 (2023). 36877887 10.1021/acs.joc.2c02627

[57] R. Zhang, Z. Liu, Y. Liu, H. Yang, X. Wang, Z. Han, Z. Wang, K. Ding, Palladium-catalyzed regioselective asymmetric chemodivergent allylation of oxazolones with morita–baylis–hillman adducts. CCS Chem. 5, 2790–2798 (2023).

[58] B. Chen, L. Pagès, R. Dollet, C. Kouklovsky, S. Prévost, A. de la Torre, Regio- and chemoselective double allylic substitution of alkenyl vic-diols. Org. Lett. 26, 2393–2397 (2024). 38488643 10.1021/acs.orglett.4c00495

[59] K. Kubota, E. Yamamoto, H. Ito, Copper(I)-catalyzed borylative exo-cyclization of alkenyl halides containing unactivated double bond. J. Am. Chem. Soc. 135, 2635–2640 (2013). 23351005 10.1021/ja3104582

[60] S. Mayr, M. Marin-Luna, H. Zipse, Size-driven inversion of selectivity in esterification reactions: Secondary beat primary alcohols. J. Org. Chem. 86, 3456–3489 (2021). 33555864 10.1021/acs.joc.0c02848

[61] X. Chen, Z. Cheng, J. Guo, Z. Lu, Asymmetric remote C─H borylation of internal alkenes via alkene isomerization. Nat. Commun. 9, 3939 (2018). 30258070 10.1038/s41467-018-06240-y PMC6158179

[62] R. A. Altman, A. Shafir, A. Choi, P. A. Lichtor, S. L. Buchwald, An improved Cu-based catalyst system for the reactions of alcohols with aryl halides. J. Org. Chem. 73, 284–286 (2008). 18044928 10.1021/jo702024p

[63] F.-H. Yang, B. Hao, X. Yue, P.-C. Ma, Fluorescence and stimuli-responsive performance of polymer composites filled with tetraphenylethene derivatives. Polym. Chem. 13, 3126–3135 (2022).

[64] L. Britton, M. Skrodzki, G. S. Nichol, A. P. Dominey, P. Pawluć, J. H. Docherty, S. P. Thomas, Manganese-catalyzed C(*sp^2^*)–H borylation of furan and thiophene derivatives. ACS Catal. 11, 6857–6864 (2021).

[65] C. Chen, H. Wang, T. Li, D. Lu, J. Li, X. Zhang, X. Hong, Z. Lu, Cobalt-catalyzed asymmetric sequential hydroboration/isomerization/hydroboration of 2-aryl vinylcyclopropanes. Angew. Chem. Int. Ed. 61, e202205619 (2022). 10.1002/anie.202205619 35607762

[66] L. Krause, R. Herbst-Irmer, G. M. Sheldrick, D. Stalke, Comparison of silver and molybdenum microfocus x-ray sources for single-crystal structure determination. J. Appl. Cryst. 48, 3–10 (2015). 26089746 10.1107/S1600576714022985 PMC4453166

[67] O. V. Dolomanov, L. J. Bourhis, R. J. Gildea, J. A. K. Howard, H. Puschmann, OLEX2: A complete structure solution, refinement and analysis program. J. Appl. Cryst. 42, 339–341 (2009).

[68] Q. Liu, X.-D. Su, B.-B. Zhang, H.-R. Kong, Y.-L. Tu, Z.-X. Wang, X.-Y. Chen, dn-Alkyl phosphonium iodide salts as radical dn-alkylating reagents and their applications in the photoinduced synthesis of dn-alkylated heterocycles. CCS Chem. 7, 993–1004 (2025).

[69] F. Hou, Y. Ning, L. Song, Z. Tan, J. Yang, Z. Liu, F.-E. Chen, Rhodium-catalyzed asymmetric hydroboration/cyclization of 1,6-enynes enabled by spirosiladiphosphine ligands: Constructing chiral five-membered rings with a boron handle. Org. Lett. 25, 7810–7815 (2023). 37883235 10.1021/acs.orglett.3c02979

[70] Z.-Y. Xiao, Z.-L. Wang, Y.-H. Xu, Copper-catalyzed ring-opening hydrosilylation and hydroboration of arylidenecyclopropanes. Chin. J. Chem. 43, 385–392 (2025).

[71] Y. Liao, S. Liu, J. Lin, K. Chen, J. Tang, L. Zou, Q. Peng, X. Wang, Binuclear cobalt complex-catalyzed dehydrogenation of ammonia borane and transfer hydrogenation of alkenes, carbonyls and nitriles: High efficiency under mild conditions. Chin. J. Chem. 44, 987–994 (2026).

