# Supplementary material for: Precise borylation of targeted methyl group via an orderly chain-walking strategy
Source: Sci Adv. 2026 May 1;12(18):eaed6913. doi: 10.1126/sciadv.aed6913 (PMC13134603; doi:10.1126/sciadv.aed6913)
Supplement: Supplementary file 1 — Supplementary Text Figs. S1 to S13 References [file sciadv.aed6913_sm.pdf]

Science Advances
AAAS

# Supplementary Materials for

## Precise borylation of targeted methyl group via an orderly chain-walking strategy

Yinwei Bao *et al.*

Corresponding author: Zhan Lu, luzhan@zju.edu.cn

*Sci. Adv.* **12**, eaed6913 (2026)
DOI: 10.1126/sciadv.aed6913

**This PDF file includes:**

Supplementary Text
Figs. S1 to S13
References

# I. General Information

All air- and moisture-sensitive manipulations were carried out using standard high vacuum line, Schlenk or cannula techniques in Mikrouna, or Vigor inert atmosphere drybox containing an atmosphere of purified nitrogen. The Vigor drybox was equipped with a cold well designed for freezing samples. All solvent were distilled from sodium benzophenone ketyl prior to use. Pinacolborane (HBpin) (97%) was purchased from Aldrich and used as received. $NaBHEt_3$ (1.0 M in THF) and $KBHEt_3$ (1.0 M in THF) were purchased from Aldrich or AcroSeal and used as received. $CoCl_2$ (99.5%) was purchased from Aladdin and used as received. The other commercially available chemicals were purchased from bidepharm, energy chemical, or leyan and used as received.

The NMR spectra were recorded on a Bruker-400 instrument, Bruker-600 instrument, Oxford, or JEOL instrument. $^1H$ NMR chemical shifts were referenced to tetramethylsilane signal (0 ppm), $^{13}C$ NMR chemical shifts were referenced to the solvent resonance (77.00 ppm, $CDCl_3$). The following abbreviations (or combinations thereof) were used to explain multiplicities: s = singlet, d = doublet, t = triplet, m = multiplet, br = broad, q = quadruplet, PE = petroleum ether, EA = ethyl acetate. IR spectra were recorded on a Perkin-Elmer Spectrum One FTIR spectrometer with diamond ATR accessory. HPLC analyses were performed on a Shimadzu SPD-20A. High-resolution mass spectra (HRMS) were recorded on Waters XEVOG2-STOF or GCT Premier. Optical rotation data were obtained on aRudolph Research Analytical AUTOPOL® I automatic polarimeter.

# II. Procedures for The Preparation of Ligands and Metal Complexes

## Figure S1. Selected cobalt catalysts

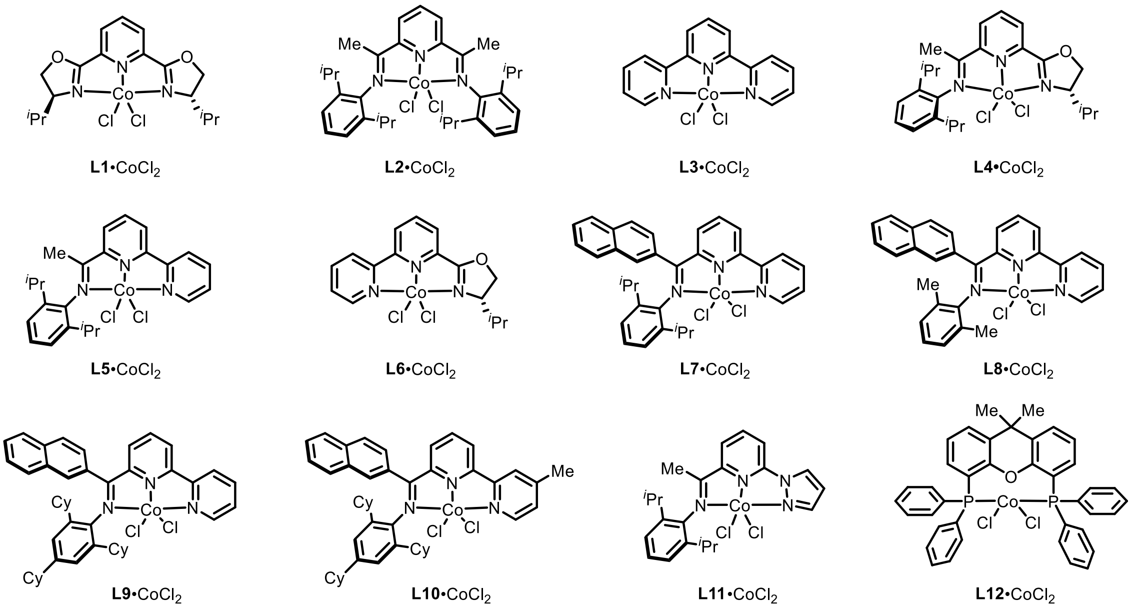


**L1 – L6, L11 - L12** and their cobalt catalysts were prepared according to the previously reported procedures (*38, 40, 41, 49*).

### Synthesis of **L7 and L7**·$CoCl_2$

**1-([2,2'-bipyridin]-6-yl)-N-(2,6-diisopropylphenyl)-1-(naphthalen-2-yl)methanimine (L7)**

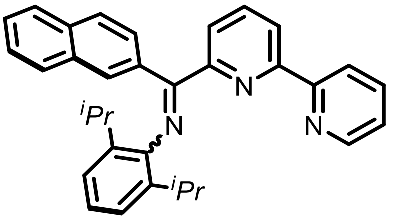

According to the previously reported procedures: 63% yield, yellow solid. $^1$H NMR: (400 MHz, $CDCl_3$) $\delta$ 8.63-6.99 (m, 17H), 3.14-2.92 (m, 2H), 1.18 (d, $J$ = 6.4 Hz, 6H), 0.95 (d, $J$ = 6.4 Hz, 6H); *As **L7** existed as an E/Z mixture, the carbon spectrum was included in the NMR Spectra without data interpretation*; IR (cm$^{-1}$): 2976, 2906, 1766, 1604, 1405, 1253; HRMS (EI) calculated for $[C_{33}H_{31}N_3]^+$ ($M^+$) requires m/z 469.2512, found m/z 469.2517.

**L7·$CoCl_2$**

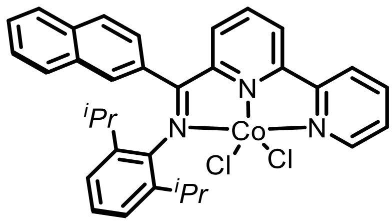


According to the previously reported procedures:[1] 89% yield, brown powder. Anal. Calcd for $C_{33}H_{31}Cl_2CoN_3$ + $0.5H_2O$: C, 65.14; H, 5.30; N, 6.91; Found: C, 65.57; H, 5.20; N, 6.96.

## Synthesis of **L8 and L8**·$CoCl_2$

**1-([2,2'-bipyridin]-6-yl)-N-(2,6-dimethylphenyl)-1-(naphthalen-2-yl)methanimine (L8)**

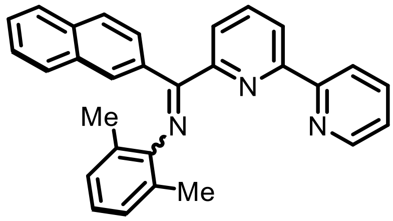


According to the previously reported procedures: 87% yield, yellow solid; $^1$H NMR: (400 MHz, $CDCl_3$) $\delta$ 8.64-7.39 (m, 13H), 7.16-6.72 (m, 4H), 2.16 (s, 4.3H), 2.11 (s, 1.7H); *As **L8** existed as an E/Z mixture, the carbon spectrum was included in the NMR Spectra without data interpretation*; IR (cm$^{-1}$): 2980, 2906, 1621, 1581, 1403, 1256; HRMS (EI) calculated for $[C_{29}H_{23}N_3]^+$ ($M^+$) requires m/z 413.1886, found m/z 413.1882.

**L8·$CoCl_2$**

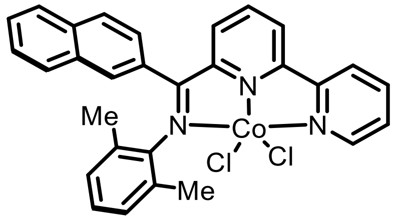


According to the previously reported procedures: 52% yield, brown powder. Anal. Calcd for $C_{29}H_{23}Cl_2CoN_3$ + $0.5H_2O$: C, 63.06; H, 4.38; N, 7.61; Found: C, 63.52; H, 4.33; N, 7.62.

## Synthesis of **L9 and L9**·$CoCl_2$

**1-([2,2'-bipyridin]-6-yl)-1-(naphthalen-2-yl)-N-(2,4,6-tricyclohexylphenyl)methanimine (L9)**

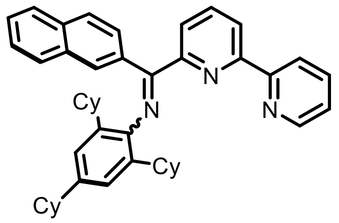


According to the previously reported procedures: 70% yield, yellow solid; $^{1}$H NMR: (400 MHz, $CDCl_3$) $\delta$ 8.61-7.09 (m, 14H), 6.80 (s, 2H), 2.60-2.30 (m, 3H), 2.04-1.08 (m, 30H); *As **L9** existed as an E/Z mixture, the carbon spectrum was included in the NMR Spectra without data interpretation*; IR ($cm^{-1}$): 2980, 2916, 1597, 1452, 1405, 1254; HRMS (ESI) calculated for $[C_{45}H_{50}N_3]^+$ (M + $H^+$) requires m/z 632.3999, found m/z 632.4004.

**L9·CoCl₂**

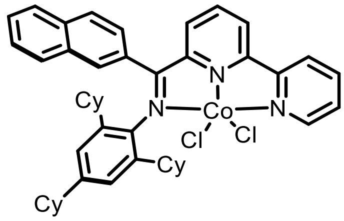


According to the previously reported procedures: 94% yield, brown powder. Anal. Calcd for $C_{45}H_{49}Cl_2CoN_3$ + $H_2O$: C, 69.32; H, 6.59; N, 5.39; Found: C, 69.44; H, 6.60; N, 5.23.

## Synthesis of **L10 and L10·CoCl₂**

**1-(4'-methyl-[2,2'-bipyridin]-6-yl)-1-(naphthalen-2-yl)-N-(2,4,6-tricyclohexylphenyl) methanimine (L10)**

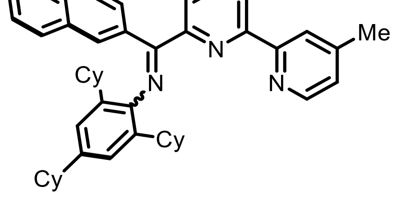


According to the previously reported procedures: 91% yield, yellow solid, $^{1}$H NMR: (400 MHz, $CDCl_3$) $\delta$ 8.52-7.06 (m, 13H), 6.81-6.78 (m, 2H), 2.59-2.20 (m, 6H), 2.03-1.06 (m, 30H); *As **L10** existed as an E/Z mixture, the carbon spectrum was included in the NMR Spectra without data interpretation*; IR ($cm^{-1}$): 2981, 2921, 1609, 1449, 1405, 1256; HRMS (ESI) calculated for $[C_{46}H_{52}N_3]^+$ (M + $H^+$) requires m/z 646.4156, found m/z 646.4159.

**L10·CoCl₂**

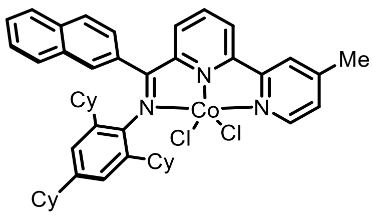


According to the previously reported procedures: 99% yield, green powder. Anal. Calcd for $C_{46}H_{51}Cl_2CoN_3 + 0.5H_2O$: C, 70.40; H, 6.68; N, 5.35; Found: C, 70.25; H, 6.49; N, 5.35.

# III. Procedures for The Synthesis of Alkenes

## Figure S2. General synthetic procedure of *E/Z* mixed trisubstituted alkenes

**Method A:**

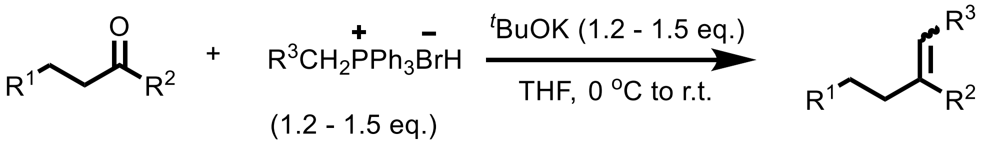


**eq. S1**

**Method B:**

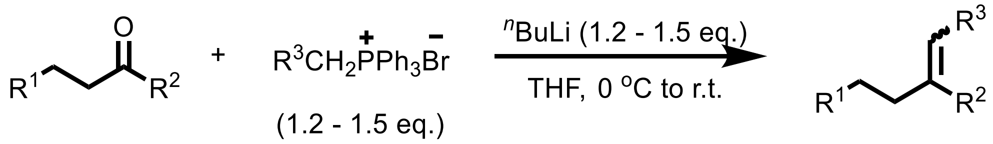


**eq. S2**

## Figure S3. Selected trisubstituted alkenes

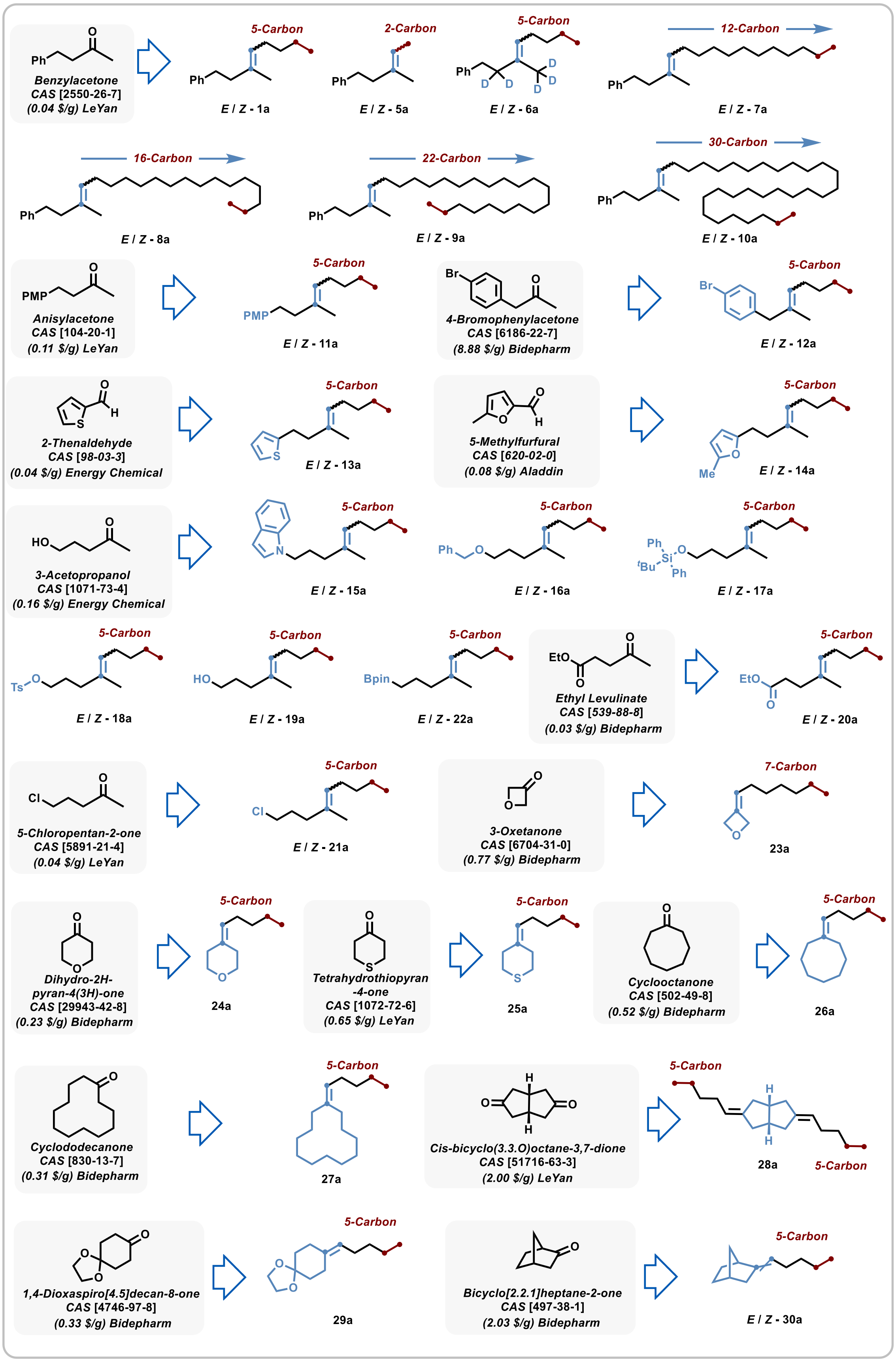

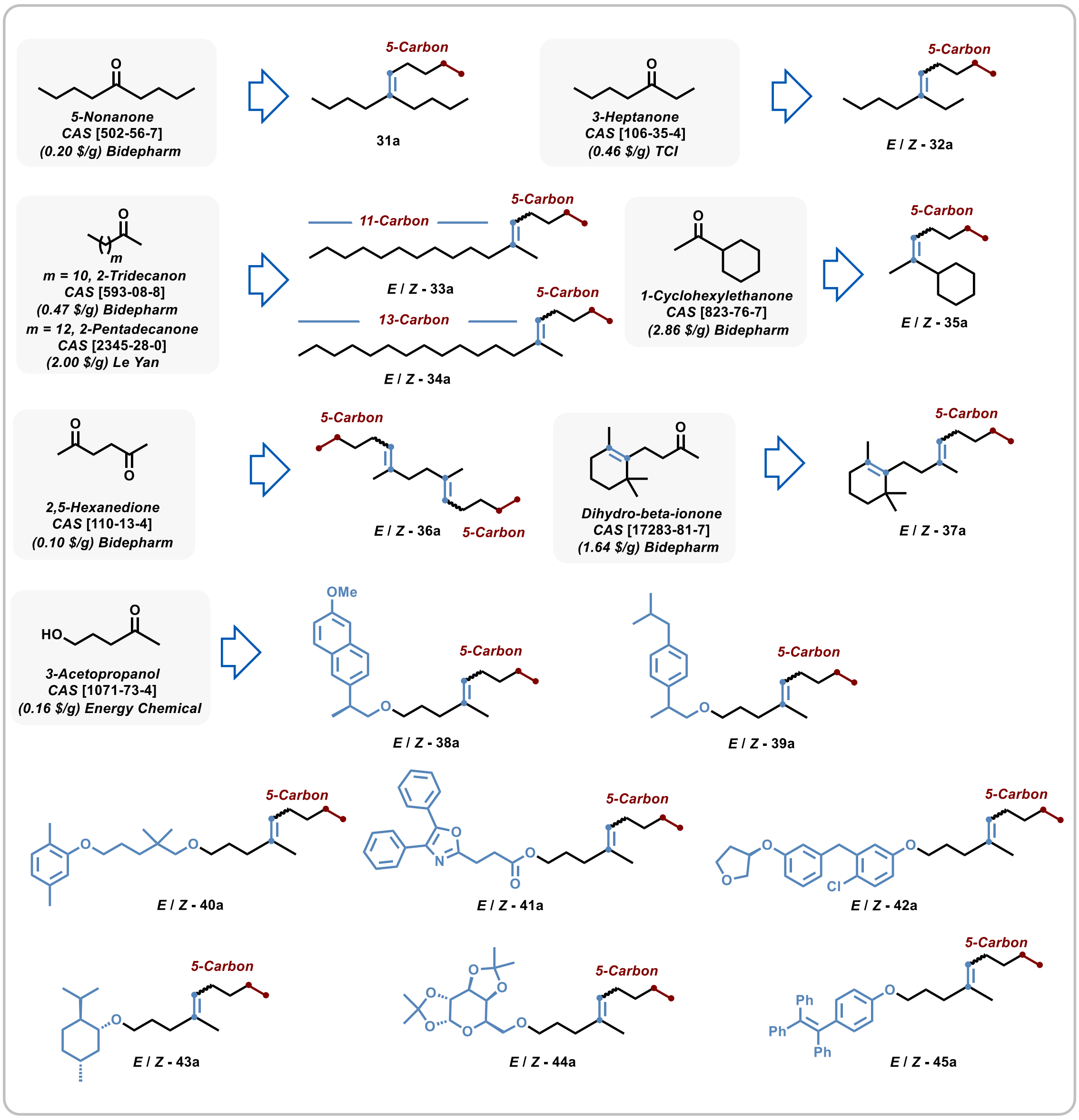


**(3-methyloct-3-en-1-yl)benzene (1a)**

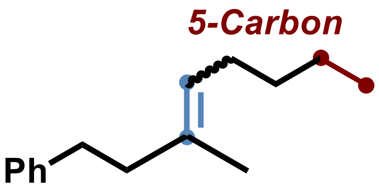


According to **method A**, benzylacetone (20 mmol) and amyltriphenylphosphonium bromide (30 mmol) were used as starting materials, the reaction afforded 3.79 g (*E* / *Z* = 1.0 / 1.5, 94% yield) of **1a** as a colorless oil. *The E/Z ratio of **1a** was determined by consulting previous literature (50).* $^1$H NMR: (400 MHz, $CDCl_3$) $\delta$ 7.28-7.15 (m, 5H), 5.16-5.11 (m, 1H), 2.72-2.64 (m, 2H), 2.30-2.25 (m, 2H), 2.00-1.86 (m, 2H), 1.72 (s, 1.8H for *Z* isomer), 1.64 (s, 1.2H for *E* isomer), 1.30-1.19 (m, 4H), 0.90-0.85 (m, 3H); *As **1a** existed as an E/Z mixture, the carbon spectrum was included both E and Z isomers*; $^{13}$C NMR:

(100 MHz, $CDCl_3$) $\delta$ 142.5, 142.4, 134.2, 134.1, 128.4, 128.23, 128.17, 126.2, 125.7, 125.6, 125.3, 41.7, 34.8, 34.5, 34.0, 32.2, 32.0, 27.6, 27.5, 23.4, 22.4, 22.3, 16.0, 14.0; HRMS (EI) calculated for $[C_{15}H_{22}]^+$ ($M^+$) requires m/z 202.1716, found m/z 202.1714.

**(3-methylpent-3-en-1-yl) benzene (5a)**

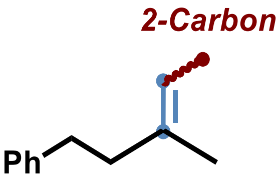


According to **method A**, benzylacetone (20 mmol) and methyltriphenylphosphonium bromide (24 mmol) were used as starting materials, the reaction afforded 2.63 g (*E* / *Z* = 1.0 / 1.1, 82% yield) of **5a** as a colorless oil. *The E/Z ratio of **5a** was determined by consulting previous literature (51).* $^1$H NMR: (400 MHz, $CDCl_3$) $\delta$ 7.28-7.17 (m, 5H), 5.28-5.18 (m, 1H), 2.72-2.65 (m, 2H), 2.34-2.23 (m, 2H), 1.72 (s, 1.6H for *Z* isomer), 1.66 (s, 1.4H for *E* isomer), 1.57 (d, $J$ = 5.6 Hz, 1.4H), 1.47 (d, $J$ = 5.2 Hz, 1.6H); *As **5a** existed as an E/Z mixture, the carbon spectrum was included both E and Z isomers*; $^{13}$C NMR: (100 MHz, $CDCl_3$) $\delta$ 142.6, 142.4, 135.3, 135.2, 128.4, 128.4, 128.2, 125.7, 125.6, 119.7, 118.8, 41.7, 34.8, 34.1, 33.6, 23.4, 15.8, 13.3, 13.1; HRMS (EI) calculated for $[C_{12}H_{16}]^+$ ($M^+$) requires m/z 160.1247, found m/z 160.1246.

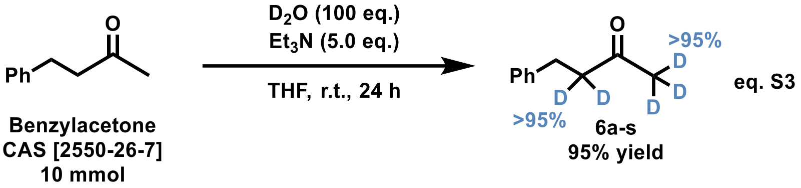


**(3-(methyl-d$_3$)oct-3-en-1-yl-2,2-d$_2$)benzene (6a)**

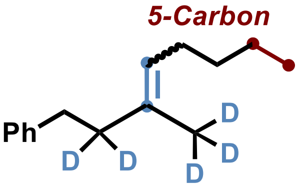


According to **method B**, **6a-s** (10 mmol) *which was synthesized by consulting previous literature* (*52*) and amyltriphenylphosphonium bromide (12 mmol) were used as starting materials, the reaction afforded 1.70 g (82% yield) of **6a** as a colorless oil. $^1$H NMR: (400 MHz, $CDCl_3$) $\delta$ 7.27-7.16 (m, 5H), 5.16-5.11

(m, 1H), 2.68-2.65 (m, 2H), 2.38-2.34 (m, 0.08H), 1.98-1.86 (m, 2H), 1.77-1.70 (m, 0.14H), 1.28-1.23 (m, 4H), 0.87 (dd, $J$ = 6.8, 6.0 Hz, 3H); $^2$H NMR: (92 MHz, $CDCl_3$) $\delta$ 2.38-2.34 (m, 1.92D), 1.77-1.70 (m, 2.86D); *As **6a** existed as an E/Z mixture, the carbon spectrum was included both E and Z isomers*; $^{13}$C NMR: (100 MHz, $CDCl_3$) $\delta$ 142.5, 142.4, 128.38, 128.37, 128.24, 128.18, 126.2, 125.7, 125.6, 125.4, 34.6, 34.3, 32.2, 32.0, 27.6, 27.5, 22.4, 22.3, 14.0; HRMS (EI) calculated for $[C_{15}H_{17}D_5]^+$ ($M^+$) requires m/z 207.2030, found m/z 207.2029.

**(3-methylpentadec-3-en-1-yl)benzene (7a)**

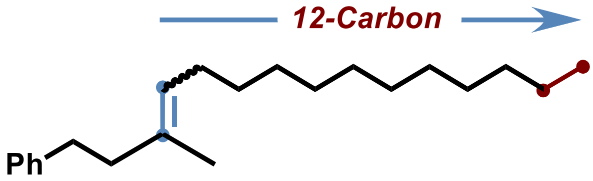


According to **method A**, benzylacetone (13 mmol) and dodecyltriphenylphosphonium bromide (16 mmol) were used as starting materials, the reaction afforded 3.66 g ($E$ / $Z$ = 1.0 / 1.5, 94% yield) of **7a** as a colorless oil. *The E/Z ratio of **5a** was inferred from previous rules (50).* $^1$H NMR: (400 MHz, $CDCl_3$) $\delta$ 7.27-7.15 (m, 5H), 5.16-5.11 (m, 1H), 2.71-2.64 (m, 2H), 2.33-2.25 (m, 2H), 1.99-1.87 (m, 2H), 1.72 (s, 1.8H for $Z$ isomer), 1.64 (s, 1.2H for $E$ isomer), 1.30-1.24 (m, 18H), 0.88 (dd, $J$ = 6.8, 6.4 Hz, 3H); *As **7a** existed as an E/Z mixture, the carbon spectrum was included both E and Z isomers*; $^{13}$C NMR: (100 MHz, $CDCl_3$) $\delta$ 142.5, 142.4, 134.14, 134.09, 128.4, 128.23, 128.18, 126.3, 125.7, 125.6, 125.4, 41.7, 34.8, 34.5, 34.02, 31.96, 30.0, 29.8, 29.73, 29.70, 29.6, 29.44, 29.40, 29.3, 27.9, 27.8, 23.4, 22.7, 16.1, 14.1; HRMS (EI) calculated for $[C_{22}H_{36}]^+$ ($M^+$) requires m/z 300.2812, found m/z 300.2811.

**(3-methylnonadec-3-en-1-yl)benzene (8a)**

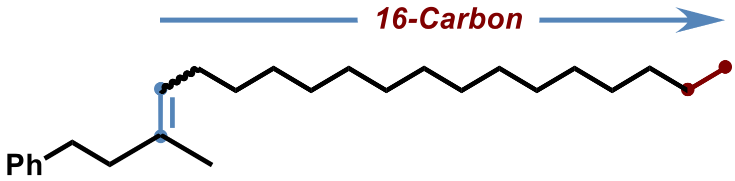


According to **method A**, benzylacetone (14.7 mmol) and (1-Hexadecyl) triphenylphosphoniumbromide (17.6 mmol) were used as starting materials, the reaction afforded 4.8760 g ($E$ / $Z$ = 1.0 / 1.5, 93% yield) of **8a** as a colorless oil. *The E/Z ratio of **8a** was inferred from previous rules (50).* $^1$H NMR: (400 MHz, $CDCl_3$) $\delta$ 7.28-7.14 (m, 5H), 5.16-5.12 (m, 1H), 2.72-2.64 (m, 2H), 2.33-2.25 (m, 2H), 1.99-1.87 (m,

2H), 1.72 (s, 1.8H for *Z* isomer), 1.64 (s, 1.2H for *E* isomer), 1.30-1.26 (m, 26H), 0.88 (dd, *J* = 6.8, 6.4 Hz, 3H); *As **8a** existed as an E/Z mixture, the carbon spectrum was included both E and Z isomers*; $^{13}$C NMR: (100 MHz, $CDCl_3$) *δ* 142.5, 142.4, 134.2, 134.1, 128.4, 128.23, 128.18, 126.3, 125.7, 125.6, 125.4, 41.7, 34.8, 34.5, 34.0, 31.9, 30.0, 29.8, 29.72, 29.68, 29.6, 29.43, 29.38, 29.3, 27.9, 27.8, 23.5, 22.7, 16.1, 14.1; HRMS (EI) calculated for $[C_{26}H_{44}]^+$ ($M^+$) requires m/z 356.3438, found m/z 356.3439.

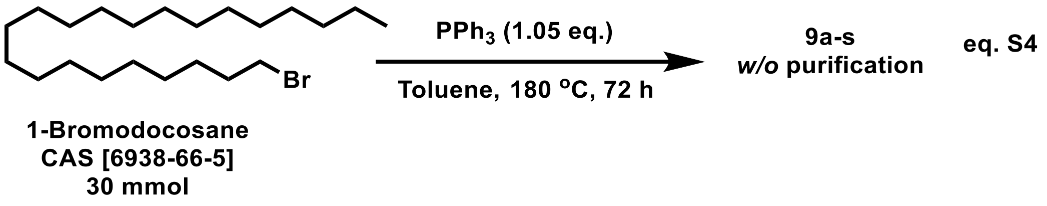


**(3-methylpentacos-3-en-1-yl)benzene (9a)**

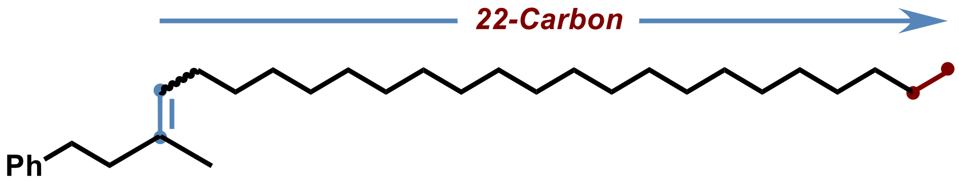


According to **method A**, benzylacetone (20 mmol) and **9a-s** (30 mmol) were used as starting materials, the reaction afforded 7.67 g (*E* / *Z* = 1.3 / 1.0, 87% yield) of **9a** as a white solid. *The E/Z ratio of **9a** was inferred from previous rules (50).* $^{1}$H NMR: (400.0 MHz, $CDCl_3$) *δ* 7.29-7.24 (m, 2H), 7.20-7.16 (m, 3H), 5.16-5.12 (m, 1H), 2.72-2.64 (m, 2H), 2.33-2.25 (m, 2H), 1.99-1.87 (m, 2H), 1.72 (s, 1.3H for *Z* isomer), 1.64 (s, 1.7H for *E* isomer), 1.35-1.11 (m, 38H), 0.88 (dd, *J* = 7.2, 6.4 Hz, 3H); *As **9a** existed as an E/Z mixture, the carbon spectrum was included both E and Z isomers*; $^{13}$C NMR: (100 MHz, $CDCl_3$) *δ* 142.51, 142.46, 134.2, 134.1, 128.4, 128.24, 128.19, 126.3, 125.7, 125.6, 125.4, 41.7, 34.8, 34.5, 34.0, 31.9, 30.0, 29.8, 29.72, 29.68, 29.6, 29.42, 29.38, 29.3, 27.9, 27.8, 23.5, 22.7, 16.1, 14.1; HRMS (EI) calculated for $[C_{32}H_{56}]^+$ ($M^+$) requires m/z 440.4377, found m/z 440.4381.

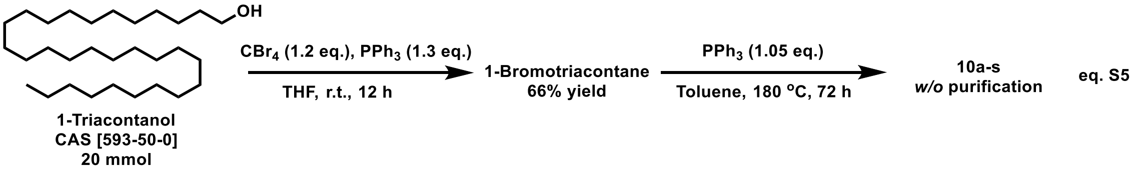


**(3-methyltritriacont-3-en-1-yl)benzene (10a)**

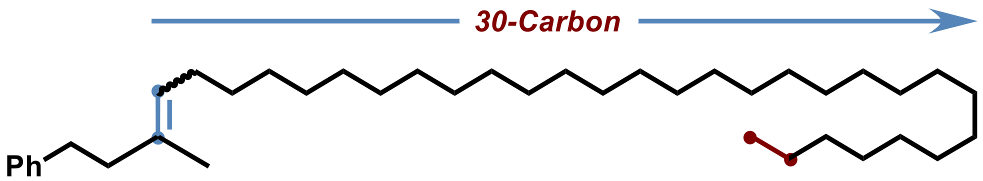


According to **method A**, benzylacetone (10 mmol) and **10a-s** (12 mmol) were used as starting materials, the reaction afforded 2.64 g (*E* / *Z* = 1.0 / 1.3, 48% yield) of **10a** as a white solid. *The E/Z ratio of **10a** was inferred from previous rules (50).* $^1$H NMR: (400 MHz, $CDCl_3$) $\delta$ 7.27-7.17 (m, 5H), 5.15-5.13 (m, 1H), 2.72-2.65 (m, 2H), 2.33-2.25 (m, 2H), 1.97-1.87 (m, 2H), 1.73 (s, 1.7H for *Z* isomer), 1.65 (s, 1.3H for *E* isomer), 1.35-1.15 (m, 54H), 0.88 (dd, $J$ = 6.8, 5.6 Hz, 3H); *As **10a** existed as an E/Z mixture, the carbon spectrum was included both E and Z isomers*; $^{13}$C NMR: (100 MHz, $CDCl_3$) $\delta$ 142.5, 142.4, 134.2, 134.1, 128.4, 128.23, 128.18, 126.3, 125.7, 125.6, 125.4, 41.7, 34.8, 34.5, 34.0, 31.9, 30.0, 29.8, 29.70, 29.66, 29.60, 29.41, 29.37, 29.30, 27.9, 27.8, 23.5, 22.7, 16.1, 14.1; HRMS (EI) calculated for $[C_{40}H_{72}]^+$ ($M^+$) requires m/z 552.5629, found m/z 552.5622.

**1-methoxy-4-(3-methyloct-3-en-1-yl)benzene (11a)**

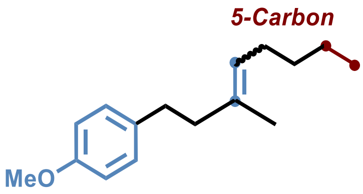


According to **method A**, anisylacetone (20 mmol) and amyltriphenylphosphonium bromide (30 mmol) were used as starting materials, the reaction afforded 4.22 g (*E* / *Z* = 1.0 / 1.7, 91% yield) of **11a** as a colorless oil. *The E/Z ratio of **11a** was inferred from previous rules (50).* $^1$H NMR: (400 MHz, $CDCl_3$) $\delta$ 7.08 (dd, $J$ = 8.4, 7.6 Hz, 2H), 6.82-6.79 (m, 2H), 5.16-5.10 (m, 1H), 3.76 (s, 3H), 2.66-2.59 (m, 2H), 2.30-2.21 (m, 2H), 1.98-1.88 (m, 2H), 1.71 (s, 1.9H for *Z* isomer), 1.63 (s, 1.1H for *E* isomer), 1.28-1.22 (m, 4H), 0.90-0.85 (m, 3H); *As **11a** existed as an E/Z mixture, the carbon spectrum was included both E and Z isomers*; $^{13}$C NMR: (100 MHz, $CDCl_3$) $\delta$ 157.7, 157.6, 134.6, 134.5, 134.22, 134.17, 129.2, 126.1, 125.3, 113.63, 113.58, 55.2, 41.9, 34.2, 33.8, 33.5, 32.2, 32.0, 27.6, 27.5, 23.4, 22.4, 22.3, 16.0, 14.0; HRMS (EI) calculated for $[C_{16}H_{24}O]^+$ ($M^+$) requires m/z 232.1822, found m/z 232.1823.

**1-bromo-4-(2-methylhept-2-en-1-yl)benzene (12a)**

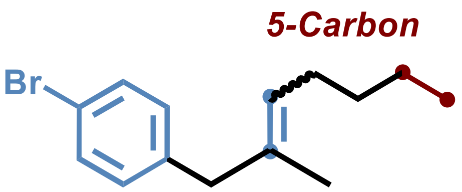


According to **method A**, 4-bromophenylacetone (20 mmol) and amyltriphenylphosphonium bromide (30 mmol) were used as starting materials, the reaction afforded 1.33 g (*E* / *Z* = 1.0 / 1.5, 25% yield) of **12a** as a colorless oil. *The E/Z ratio of **12a** was inferred from previous rules (50).* $^1$H NMR: (400 MHz, $CDCl_3$) $\delta$ 7.37 (d, *J* = 8.4 Hz, 2H), 7.02 (d, *J* = 8.0 Hz, 2H), 5.33-5.32 (m, 0.6H), 5.24-5.22 (m, 0.4H), 3.30 (s, 1.2H for *Z* isomer), 3.21 (s, 0.8H for *E* isomer), 2.11-1.99 (m, 2H), 1.58 (s, 1.8H for *Z* isomer), 1.50 (s, 1.2H for *E* isomer), 1.36-1.32 (m, 4H), 0.92-0.89 (m, 3H); *As **12a** existed as an E/Z mixture, the carbon spectrum was included both E and Z isomers*; $^{13}$C NMR: (100 MHz, $CDCl_3$) $\delta$ 139.5, 139.3, 133.6, 132.9, 131.3, 131.2, 130.5, 130.2, 127.4, 119.6, 119.5, 45.6, 37.2, 32.2, 31.9, 27.9, 27.7, 23.2, 22.44, 22.37, 15.7, 14.0; HRMS (EI) calculated for $[C_{14}H_{19}Br]^+$ ($M^+$) requires m/z 266.0665, found m/z 266.0665.

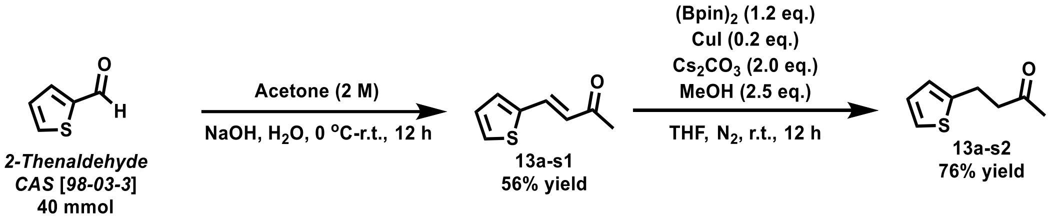


eq. S6

**2-(3-methyloct-3-en-1-yl)thiophene (13a)**

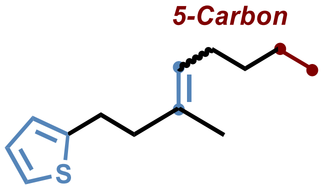


According to **method A**, **13a-s2** (15 mmol) *which was synthesized by consulting previous literature (53, 54)* and amyltriphenylphosphonium bromide (18 mmol) were used as starting materials, the reaction afforded 2.79 g (*E* / *Z* = 1.0 / 1.7, 89% yield) of **13a** as a colorless oil. *The E/Z ratio of **13a** was inferred from previous rules(51).* $^1$H NMR: (400 MHz, $CDCl_3$) $\delta$ 7.09 (dd, *J* = 5.2, 4.8 Hz, 1H), 6.91-6.88 (m, 1H), 6.79-6.76 (m, 1H), 5.19-5.18 (m, 1H), 2.94-2.86 (m, 2H), 2.40-2.32 (m, 2H), 1.99-1.91 (m, 2H), 1.72 (s, 1.9H for *Z* isomer), 1.64 (s, 1.1H for *E* isomer), 1.33-1.24 (m, 4H), 0.88 (dd, *J* = 7.2, 6.8 Hz, 3H); *As **13a** existed as an E/Z mixture, the carbon spectrum was included both E and Z isomers*; $^{13}$C NMR: (100 MHz, $CDCl_3$) $\delta$ 145.2, 145.1, 133.6, 133.5, 126.7, 126.6, 126.5, 125.9, 124.0, 123.9, 122.9,

122.8, 41.7, 34.1, 32.2, 31.9, 28.7, 28.4, 27.6, 27.5, 23.3, 22.4, 22.3, 15.9, 14.0; HRMS (EI) calculated for $[C_{13}H_{20}S]^+$ ($M^+$) requires m/z 208.1280, found m/z 208.1280.

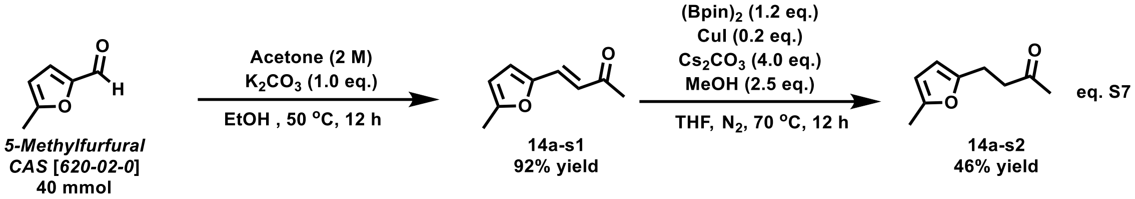


__**2-methyl-5-(3-methyloct-3-en-1-yl)furan (14a)**__

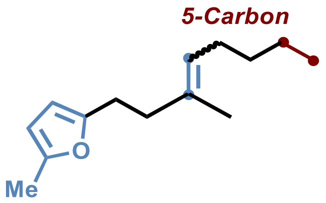


According to **method A**, **14a-s2** (16.9 mmol) *which was synthesized by consulting previous literature (53, 54)* and amyltriphenylphosphonium bromide (20.2 mmol) were used as starting materials, the reaction afforded 2.15 g ($E$ / $Z$ = 1.0 / 1.5, 62% yield) of **14a** as a colorless oil. *The E/Z ratio of **14a** was inferred from previous rules (50).* $^1$H NMR: (400 MHz, $CDCl_3$) $\delta$ 5.83 (s, 2H), 5.22-5.11 (m, 1H), 2.68-2.60 (m, 2H), 2.35-2.28 (m, 2H), 2.25 (s, 3H), 1.98-1.93 (m, 2H), 1.69 (s, 1.8H for $Z$ isomer), 1.62 (s, 1.2H for $E$ isomer), 1.38-1.16 (m, 4H), 0.97-0.87 (m, 3H); *As **14a** existed as an E/Z mixture, the carbon spectrum was included E and Z isomers*; $^{13}$C NMR: (100 MHz, $CDCl_3$) $\delta$ 154.3, 154.1, 150.1, 150.0, 133.9, 133.8, 126.4, 125.4, 105.8, 105.7, 105.3, 105.2, 38.2, 32.2, 32.0, 30.5, 27.6, 27.4, 27.0, 26.6, 23.2, 22.4, 22.3, 15.8, 14.0, 13.5; HRMS (EI) calculated for $[C_{14}H_{22}O]^+$ ($M^+$) requires m/z 206.1665, found m/z 206.1667.

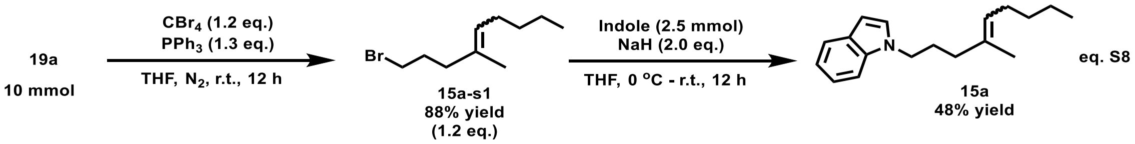


__**1-(4-methylnon-4-en-1-yl)-1H-indole (15a)**__

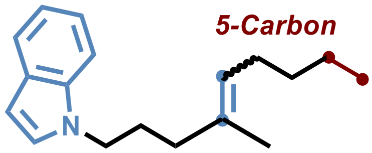

According to **the above method(*56*)**, the reaction afforded 0.31 g (*E* / *Z* = 1.0 / 2.8, 48% yield) of **15a** as a colorless oil. *The E/Z ratio of **15a** was inferred from previous rules (50).* $^{1}$H NMR: (400 MHz, $CDCl_3$) $\delta$ 7.63 (d, $J$ = 8.0 Hz, 1H), 7.33 (d, $J$ = 8.0 Hz, 1H), 7.20 (dd, $J$ = 8.0, 7.6 Hz, 1H), 7.11-7.07 (m, 2H), 6.48 (s, 1H), 5.19-5.16 (m, 1H), 4.08 (dd, $J$ = 7.6, 6.8 Hz, 2H), 2.08-1.90 (m, 6H), 1.68 (s, 2.2H for *Z* isomer), 1.59 (s, 0.8H for *E* isomer), 1.32-1.25 (m, 4H), 0.91-0.86 (m, 3H); *As **15a** existed as an E/Z mixture, the carbon spectrum was included E and Z isomers*; $^{13}$C NMR: (100 MHz, $CDCl_3$) $\delta$ 135.9, 133.5, 133.3, 128.6, 127.8, 127.6, 126.6, 125.9, 121.3, 121.2, 120.9, 119.2, 119.1, 109.4, 109.3, 100.9, 100.8, 46.1, 45.8, 36.7, 32.2, 32.0, 29.0, 28.4, 28.1, 27.6, 27.5, 23.2, 22.4, 15.8, 14.0; HRMS (EI) calculated for $[C_{18}H_{25}N]^+$ ($M^+$) requires m/z 255.1982, found m/z 255.1984.

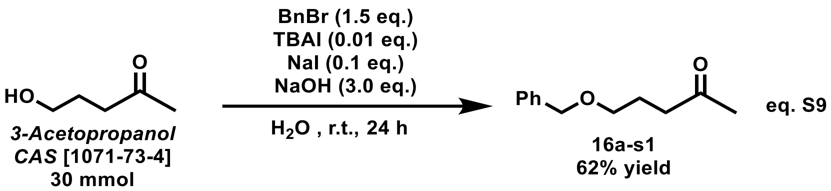


**(((4-methylnon-4-en-1-yl)oxy)methyl)benzene (16a)**

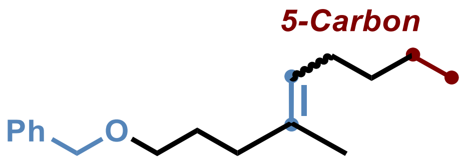


According to **method A**, **16a-s1** (15 mmol) *which was synthesized by consulting previous literature (56)* and amyltriphenylphosphonium bromide (18 mmol) were used as starting materials, the reaction afforded 3.11 g (*E* / *Z* = 1.0 / 1.7, 84% yield) of **16a** as a colorless oil. *The E/Z ratio of **16a** was inferred from previous rules (50).* $^{1}$H NMR: (400 MHz, $CDCl_3$) $\delta$ 7.34-7.23 (m, 5H), 5.16-5.13 (m, 1H), 4.49 (d, $J$ = 2.4 Hz, 2H), 3.47-3.43 (m, 2H), 2.12-1.96 (m, 4H), 1.74-1.68 (m, 3.9H for *Z* isomer), 1.60-1.59 (m, 1.1H for *E* isomer), 1.31-1.26 (m, 4H), 0.88 (t, $J$ = 6.0 Hz, 3H); *As **16a** existed as an E/Z mixture, the carbon spectrum was included E and Z isomers*; $^{13}$C NMR: (100 MHz, $CDCl_3$) $\delta$ 138.65, 138.61, 134.3, 134.1, 128.3, 127.57, 127.55, 127.4, 125.9, 125.0, 72.8, 70.1, 70.0, 36.0, 32.3, 32.0, 28.2, 28.02, 27.99, 27.6, 27.5, 23.3, 22.4, 22.3, 15.8, 14.0; HRMS (EI) calculated for $[C_{17}H_{26}O]^+$ ($M^+$) requires m/z 246.1978, found m/z 246.1976.

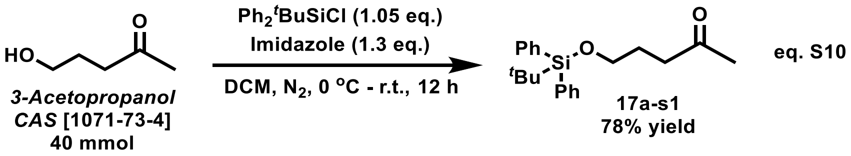


**tert-butyl((4-methylnon-4-en-1-yl)oxy)diphenylsilane (17a)**

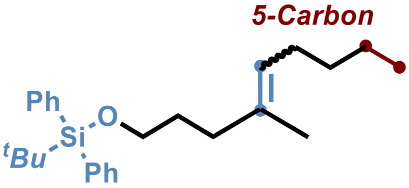


According to **method A**, **17a-s1** (20 mmol) *which was synthesized by consulting previous literature (57)* and amyltriphenylphosphonium bromide (30 mmol) were used as starting materials, the reaction afforded 5.31 g (67% yield) of **17a** as a colorless oil. $^1$H NMR: (400 MHz, $CDCl_3$) $\delta$ 7.68 (d, $J$ = 7.6 Hz, 4H), 7.42-7.35 (m, 6H), 5.12 (t, $J$ = 7.2 Hz, 1H), 3.68-3.63 (m, 2H), 2.11-1.96 (m, 4H), 1.75-1.56 (m, 5H), 1.29-1.28 (m, 4H), 1.06-1.05 (m, 9H), 0.88-0.87 (m, 3H); *As **17a** existed as an E/Z mixture, the carbon spectrum was included E and Z isomers*; $^{13}$C NMR: (100 MHz, $CDCl_3$) $\delta$ 135.6, 134.7, 134.4, 134.14, 134.10, 129.51, 129.47, 127.6, 125.6, 124.9, 63.8, 63.6, 35.8, 32.3, 32.1, 31.1, 30.9, 28.1, 27.6, 27.5, 26.9, 23.4, 22.42, 22.37, 19.2, 15.9, 14.1; HRMS (ESI) calculated for $[C_{26}H_{39}OSi]^+$ (M + H$^+$) requires m/z 395.2765, found m/z 395.2773.

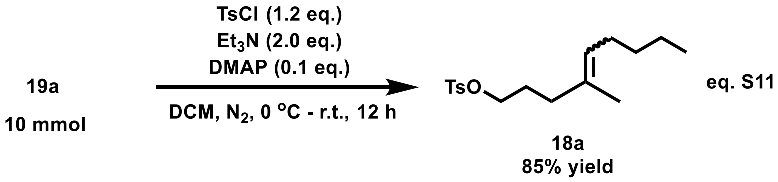


**4-methylnon-4-en-1-yl 4-methylbenzenesulfonate (18a)**

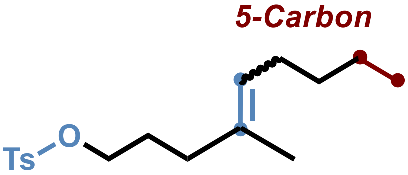


According to the **above method(*59*)**, the reaction afforded 2.63 g ($E$ / $Z$ = 1.0 / 2.3, 85% yield) of **18a** as a colorless oil. *The E/Z ratio of **18a** was inferred from previous rules (50).* $^1$H NMR: (400 MHz, $CDCl_3$) $\delta$ 7.79 (d, $J$ = 8.0 Hz, 2H), 7.34 (d, $J$ = 8.0 Hz, 2H), 5.14 (dd, $J$ = 7.2, 6.8 Hz, 0.7H for $Z$ isomer), 5.03 (t, $J$ = 7.2, 6.8 Hz, 0.3H for $E$ isomer), 4.03-3.98 (m, 2H), 2.45 (s, 3H), 2.05-1.86 (m, 4H), 1.77-1.68 (m, 2H), 1.61 (s, 2H for $Z$ isomer), 1.52 (s, 1H for $E$ isomer), 1.30-1.21 (m, 4H), 0.87 (dd, $J$ = 6.8, 5.2 Hz,

3H); *As **18a** existed as an E/Z mixture, the carbon spectrum was included E and Z isomers*; $^{13}$C NMR: (100 MHz, $CDCl_3$) $\delta$ 144.62, 144.57, 133.2, 133.1, 132.8, 132.6, 129.8, 129.7, 127.83, 127.81, 126.9, 126.1, 70.3, 70.1, 35.0, 32.1, 31.8, 27.5, 27.39, 27.37, 27.1, 26.9, 23.0, 22.3, 21.6, 15.6, 14.0, 13.9; HRMS (ESI) calculated for $[C_{17}H_{26}NaO_3]^+$ (M + $Na^+$) requires m/z 333.1495, found m/z 333.1497.

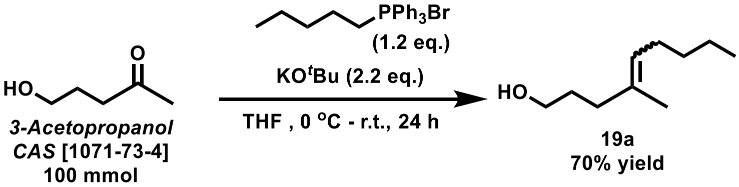


eq. S12

**<u>4-methylnon-4-en-1-ol (19a)</u>**

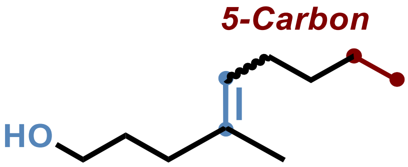


According to **method A after modification**, the reaction afforded 12.25 g (78% yield) of **19a** as a colorless oil. $^1$H NMR: (400 MHz, $CDCl_3$) $\delta$ 5.19-5.14 (m, 1H), 3.64-3.61 (m, 2H), 2.11-1.98 (m, 4H), 1.79 (brs, 1H), 1.70-1.61 (m, 5H), 1.35-1.25 (m, 4H), 0.91-0.87 (m, 3H); *As **19a** existed as an E/Z mixture, the carbon spectrum was included E and Z isomers*; $^{13}$C NMR: (100 MHz, $CDCl_3$) $\delta$ 134.40, 134.38, 126.0, 125.2, 62.9, 62.7, 36.0, 32.2, 32.0, 30.9, 30.7, 28.0, 27.5, 27.4, 23.3, 22.4, 22.3, 15.8, 13.97, 13.96; HRMS (EI) calculated for $[C_{10}H_{20}O]^+$ ($M^+$) requires m/z 156.1509, found m/z 156.1510.

**<u>ethyl 4-methylnon-4-enoate (20a)</u>**

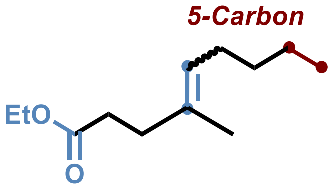


According to **method A**, ethyl levulinate (20 mmol) and amyltriphenylphosphonium bromide (24 mmol) were used as starting materials, the reaction afforded 1.26 g (*E* / *Z* = 1.0 / 2.0, 32% yield) of **20a** as a colorless oil. *The E/Z ratio of **20a** was inferred from previous rules (50).* $^1$H NMR: (400 MHz, $CDCl_3$) $\delta$ 5.19-5.16 (m, 1H), 4.16-4.11 (m, 2H), 2.42-2.29 (m, 4H), 1.99-1.98 (m, 2H), 1.68 (s, 2H for *Z* isomer), 1.61 (s, 1H for *E* isomer), 1.30-1.25 (m, 7H), 0.94-0.82 (m, 3H); *As **20a** existed as an E/Z mixture, the*

*carbon spectrum was included E and Z isomers*; $^{13}$C NMR: (100 MHz, $CDCl_3$) $\delta$ 173.4, 173.3, 133.0, 132.9, 126.8, 125.5, 60.2, 60.1, 34.7, 33.2, 32.9, 32.1, 31.8, 27.5, 27.4, 27.1, 22.9, 22.3, 22.2, 15.7, 14.1, 13.90, 13.89; HRMS (EI) calculated for $[C_{12}H_{22}O_2]^+$ ($M^+$) requires m/z 198.1614, found m/z 198.1616.

**1-chloro-4-methylnon-4-ene (21a)**

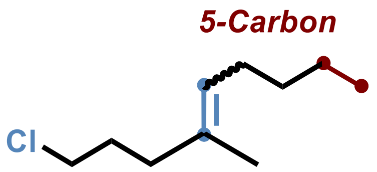


According to **method A**, 5-chloropentan-2-one (40 mmol) and amyltriphenylphosphonium bromide (50 mmol) were used as starting materials, the reaction afforded 1.14 g (*E* / *Z* = 1.0 / 1.1, 16% yield) of **21a** as a colorless oil. *The E/Z ratio of **21a** was inferred from previous rules (50).* $^1$H NMR: (400 MHz, $CDCl_3$) $\delta$ 5.23-5.13 (m, 1H), 3.53-3.46 (m, 2H), 2.18-2.10 (m, 2H), 1.99-1.83 (m, 4H), 1.68 (s, 1.6H for *Z* isomer), 1.59 (s, 1.4H for *E* isomer), 1.40-1.21 (m, 4H), 0.95-0.81 (m, 3H); *As **21a** existed as an E/Z mixture, the carbon spectrum was included E and Z isomers*; $^{13}$C NMR: (100 MHz, $CDCl_3$) $\delta$ 133.1, 132.9, 127.0, 126.1, 44.8, 44.6, 36.7, 32.2, 32.0, 30.9, 30.8, 28.9, 27.6, 27.5, 23.2, 22.4, 22.3, 15.8, 14.0; HRMS (EI) calculated for $[C_{10}H_{19}Cl]^+$ ($M^+$) requires m/z 174.1170, found m/z 174.1171.

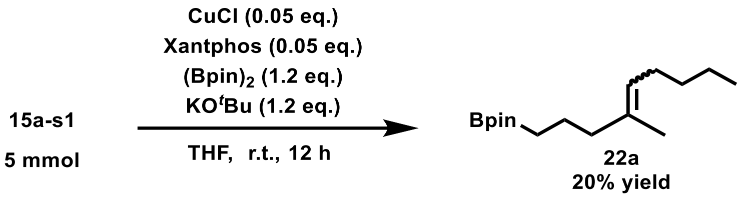


eq. S13

**4,4,5,5-tetramethyl-2-(4-methylnon-4-en-1-yl)-1,3,2-dioxaborolane (22a)**

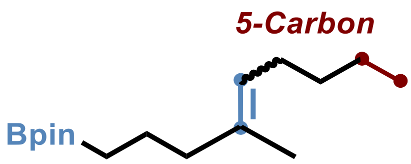


According to **the above method(*60*)**, the reaction afforded 0.27 g (*E* / *Z* = 1.0 / 1.7, 20% yield) of **22a** as a colorless oil. *The E/Z ratio of **22a** was inferred from previous rules (50).* $^1$H NMR: (400 MHz, $CDCl_3$) $\delta$ 5.11 (t, $J$ = 6.8 Hz, 1H), 2.03-1.97 (m, 4H), 1.66 (s, 1.9H for *Z* isomer), 1.57 (s, 1.1H for *E* isomer), 1.52-1.45 (m, 2H), 1.30-1.29 (m, 4H), 1.25 (s, 12H), 0.90-0.87 (m, 3H), 0.79-0.74 (m, 2H); *As **22a** existed as an E/Z mixture, the carbon spectrum was included E and Z isomers*; $^{13}$C NMR: (100 MHz, $CDCl_3$) $\delta$

135.1, 134.8, 125.5, 124.9, 82.85, 82.81, 42.4, 34.4, 32.4, 32.1, 27.6, 27.5, 24.8, 23.4, 22.5, 22.43, 22.36, 15.8, 14.0; HRMS (EI) calculated for $[C_{16}H_{31}BO_2]^+$ ($M^+$) requires m/z 266.2412, found m/z 266.2414.

**3-heptylideneoxetane (23a).**

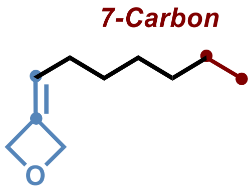


According to **method A**, 3-oxetanone (20 mmol) and heptyltriphenylphosphonium bromide (24 mmol) were used as starting materials, the reaction afforded 1.81 g (59% yield) of **23a** as a colorless oil. $^1$H NMR: (400 MHz, $CDCl_3$) $\delta$ 5.19 (d, $J$ = 15.6 Hz, 4H), 5.14-5.10 (m, 1H), 1.89-1.81 (m, 2H), 1.33-1.27 (m, 8H), 0.89 (dd, $J$ = 7.2, 6.0 Hz, 3H); $^{13}$C NMR: (100 MHz, $CDCl_3$) $\delta$ 133.5, 119.7, 79.4, 78.8, 31.6, 29.0, 28.8, 28.1, 22.5, 14.0; HRMS (EI) calculated for $[C_{10}H_{18}O]^+$ ($M^+$) requires m/z 154.1352, found m/z 154.1352.

**4-pentylidenetetrahydro-2H-pyran (24a)**

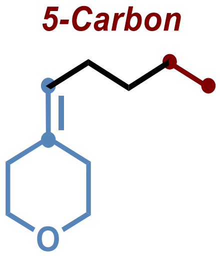


According to **method A**, dihydro-2H-pyran-4(3H)-one (20 mmol) and amyltriphenylphosphonium bromide (24 mmol) were used as starting materials, the reaction afforded 2.32 g (75% yield) of **24a** as a colorless oil. $^1$H NMR: (400 MHz, $CDCl_3$) $\delta$ 5.18 (dd, $J$ = 7.6, 7.2 Hz, 1H), 3.68-3.63 (m, 4H), 2.26 (dd, $J$ = 5.6, 5.2 Hz, 2H), 2.19 (dd, $J$ = 5.2, 5.6 Hz, 2H), 2.02-1.97 (m, 2H), 1.32-1.27 (m, 4H), 0.90 (dd, $J$ = 6.4, 4.4 Hz, 3H); $^{13}$C NMR: (100 MHz, $CDCl_3$) $\delta$ 133.9, 123.5, 69.7, 68.8, 36.9, 32.2, 29.7, 26.5, 22.2, 13.9; HRMS (EI) calculated for $[C_{10}H_{18}O]^+$ ($M^+$) requires m/z 154.1352, found m/z 154.1354.

**4-pentylidenetetrahydro-2H-thiopyran (25a)**

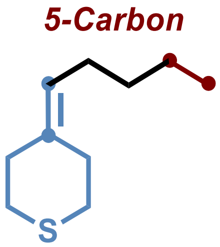


According to **method A**, tetrahydrothiopyran-4-one (10 mmol) and amyltriphenylphosphonium bromide (12 mmol) were used as starting materials, the reaction afforded 1.33 g (78% yield) of **25a** as a colorless oil. $^1$H NMR: (400 MHz, $CDCl_3$) $\delta$ 5.18 (t, $J$ = 7.2 Hz, 1H), 2.66-2.60 (m, 4H), 2.49 (dd, $J$ = 6.0, 4.8 Hz, 2H), 2.41 (dd, $J$ = 5.6, 5.2 Hz, 2H), 2.01-1.96 (m, 2H), 1.32-1.30 (m, 4H), 0.89 (t, $J$ = 6.8 Hz, 3H); $^{13}$C NMR: (100 MHz, $CDCl_3$) $\delta$ 136.1, 124.9, 38.9, 32.1, 31.2, 30.5, 30.3, 26.5, 22.3, 14.0; HRMS (EI) calculated for $[C_{10}H_{18}S]^+$ ($M^+$) requires m/z 170.1124, found m/z 170.1125.

**Pentylidenecyclooctane (26a)**

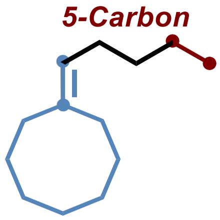


According to **method A**, cyclooctanone (20 mmol) and amyltriphenylphosphonium bromide (24 mmol) were used as starting materials, the reaction afforded 2.82 g (78% yield) of **26a** as a colorless oil. $^1$H NMR: (400 MHz, $CDCl_3$) $\delta$ 5.15 (dd, $J$ = 7.2, 6.8 Hz, 1H), 2.19-2.11 (m, 4H), 2.02-1.98 (m, 2H), 1.61-1.50 (m, 10H), 0.90 (dd, $J$ = 6.8, 6.4 Hz, 3H); $^{13}$C NMR: (100 MHz, $CDCl_3$) $\delta$ 140.5, 125.6, 37.8, 32.1, 29.1, 27.6, 27.3, 27.1, 26.4, 26.3, 26.1, 22.6, 14.1; HRMS (EI) calculated for $[C_{13}H_{24}]^+$ ($M^+$) requires m/z 180.1873, found m/z 180.1874.

**Pentylidenecyclododecane (27a)**

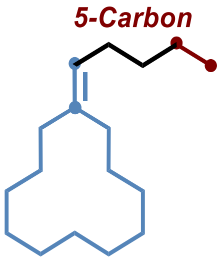

According to **method A**, cyclododecanone (20 mmol) and amyltriphenylphosphonium bromide (24 mmol) were used as starting materials, the reaction afforded 4.1748 g (88% yield) of **27a** as a colorless oil. $^1$H NMR: (400 MHz, $CDCl_3$) $\delta$ 5.19 (t, $J$ = 6.8 Hz, 1H), 2.06-2.00 (m, 6H), 1.54-1.51 (m, 2H), 1.46-1.43 (m, 2H), 1.33-1.31 (m, 18H), 0.90 (dd, $J$ = 7.2, 6.8 Hz, 3H); $^{13}$C NMR: (100 MHz, $CDCl_3$) $\delta$ 137.2, 125.4, 32.4, 31.7, 28.5, 27.7, 25.0, 24.7, 24.29, 24.27, 24.1, 24.0, 23.5, 23.2, 22.5, 22.3, 14.1; HRMS (EI) calculated for $[C_{17}H_{32}]^+$ ($M^+$) requires m/z 236.2499, found m/z 236.2500.

**(2E,3aS,5E,6aS)-2,5-dipentylideneoctahydropentalene (28a)**

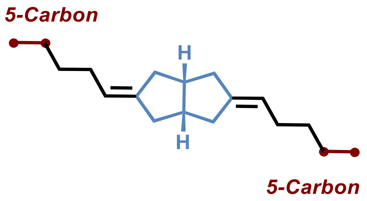


According to **method A**, cis-bicyclo(3.3.O)octane-3,7-dione (10 mmol) and amyltriphenylphosphonium bromide (24 mmol) were used as starting materials, the reaction afforded 1.29 g (52% yield) of **28a** as a colorless oil. $^1$H NMR: (400 MHz, $CDCl_3$) $\delta$ 5.20 (dd, $J$ = 6.8, 6.4 Hz, 2H), 2.44-2.37 (m, 6H), 2.07-1.93 (m, 8H), 1.30-1.29 (m, 8H), 0.90-0.87 (m, 6H); 13C NMR: (100 MHz, CDCl3) $\delta$ 143.04, 143.01, 121.4, 43.2, 43.0, 42.7, 39.5, 38.7, 34.9, 34.1, 32.0, 29.2, 22.4, 22.4, 14.0; HRMS (EI) calculated for $[C_{18}H_{30}]^+$ ($M^+$) requires m/z 246.2342, found m/z 246.2344.

**8-pentylidene-1,4-dioxaspiro[4.5]decane (29a).**

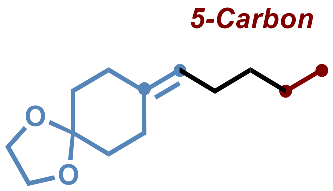


According to **method A**, 1,4-dioxaspiro[4.5]decan-8-one (20 mmol) and amyltriphenylphosphonium bromide (24 mmol) were used as starting materials, the reaction afforded 3.92 g (93% yield) of **29a** as a colorless oil. $^1$H NMR: (400 MHz, $CDCl_3$) $\delta$ 5.14 (t, $J$ = 7.2 Hz, 1H), 3.96 (s, 4H), 2.28-2.20 (m, 4H), 2.00-1.98 (m, 2H), 1.69-1.63 (m, 4H), 1.31-1.29 (m, 4H), 0.89 (t, $J$ = 6.8 Hz, 3H); $^{13}$C NMR: (100 MHz, $CDCl_3$) $\delta$ 136.4, 123.0, 109.0, 64.2, 36.3, 35.5, 33.5, 32.2, 27.0, 24.9, 22.2, 13.9; HRMS (EI) calculated for $[C_{13}H_{22}O_2]^+$ ($M^+$) requires m/z 210.1614, found m/z 210.1616.

<u>**(1S,4R)-2-pentylidenebicyclo[2.2.1]heptane (30a).**</u>

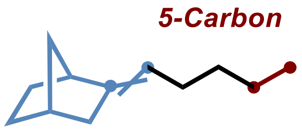


According to **method A**, bicyclo[2.2.1]heptane-2-one (20 mmol) and amyltriphenylphosphonium bromide (24 mmol) were used as starting materials, the reaction afforded 2.94 g (*E* / *Z* = 2.3 / 1.0, 90% yield) of **30a** as a colorless oil. *The E/Z ratio of **30a** was inferred from previous rules (50).* $^{1}$H NMR: (400 MHz, $CDCl_3$) $\delta$ 5.16 (t, $J$ = 7.2 Hz, 0.3H for *Z* isomer), 4.97 (t, $J$ = 7.2 Hz, 0.7H for *E* isomer), 2.90-2.59 (m, 1H), 2.35-2.30 (m, 1H), 2.16-1.81 (m, 4H), 1.60-1.53 (m, 2H), 1.33-1.22 (m, 8H), 0.91-0.86 (m, 3H); *As **30a** existed as an E/Z mixture, the carbon spectrum was included E and Z isomers*; $^{13}$C NMR: (100 MHz, $CDCl_3$) $\delta$ 145.7, 144.9, 118.0, 117.1, 45.3, 40.0, 39.2, 39.1, 38.8, 36.6, 36.3, 35.8, 32.5, 32.0, 30.1, 29.5, 29.0, 28.7, 28.5, 28.4, 22.4, 22.3, 14.1, 14.0; HRMS (EI) calculated for $[C_{12}H_{20}]^+$ ($M^+$) requires m/z 164.1560, found m/z 164.1561.

<u>**5-butyldec-5-ene (31a)**</u>

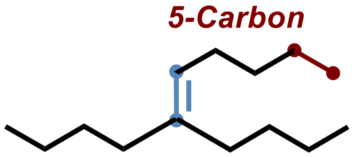


According to **method A**, 5-nonanone (30 mmol) and amyltriphenylphosphonium bromide (45 mmol) were used as starting materials, the reaction afforded 5.26 g (89% yield) of **31a** as a colorless oil. $^{1}$H NMR: (400 MHz, $CDCl_3$) $\delta$ 5.09 (dd, $J$ = 7.2, 6.8 Hz, 1H), 2.01-1.94 (m, 6H), 1.37-1.26 (m, 12H), 0.92-0.88 (m, 9H); $^{13}$C NMR: (100 MHz, $CDCl_3$) $\delta$ 139.5, 124.7, 36.7, 32.5, 30.8, 30.6, 29.8, 27.4, 22.9, 22.5, 22.4, 14.0; HRMS (EI) calculated for $[C_{14}H_{28}]^+$ ($M^+$) requires m/z 196.2186, found m/z 196.2187.

<u>**5-ethyldec-5-ene (32a)**</u>

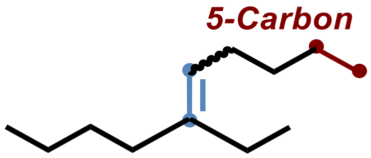


According to **method A**, 3-heptanone (20 mmol) and amyltriphenylphosphonium bromide (30 mmol) were used as starting materials, the reaction afforded 3.04 g (90% yield) of **32a** as a colorless oil. $^{1}$H

NMR: (400 MHz, $CDCl_3$) δ 5.11-5.05 (m, 1H), 2.04-1.96 (m, 6H), 1.38-1.26 (m, 8H), 1.00-0.88 (m, 9H); *As **32a** existed as an E/Z mixture, the carbon spectrum was included E and Z isomers*; $^{13}C$ NMR: (100 MHz, $CDCl_3$) δ 141.04, 141.03, 124.1, 123.5, 36.3, 32.5, 30.8, 30.5, 29.9, 29.6, 27.4, 27.3, 23.0, 22.9, 22.5, 22.5, 22.46, 22.44, 14.05, 13.99, 13.3, 13.0; HRMS (EI) calculated for $[C_{12}H_{24}]^+$ ($M^+$) requires m/z 168.1873, found m/z 168.1874.

**6-methylheptadec-5-ene (33a)**

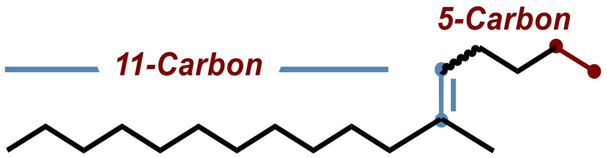


According to **method A**, 2-tridecanon (20.2 mmol) and amyltriphenylphosphonium bromide (24 mmol) were used as starting materials, the reaction afforded 5.09 g (*E* / *Z* = 1.0 / 1.3, 99% yield) of **33a** as a colorless oil. *The E/Z ratio of **33a** was inferred from previous rules (50).* $^1H$ NMR: (400 MHz, $CDCl_3$) δ 5.11 (dd, $J$ = 7.2, 6.8 Hz, 1H), 2.02-1.94 (m, 4H), 1.67 (s, 1.7H for *Z* isomer), 1.58 (s, 1.3H for *E* isomer), 1.37-1.26 (m, 22H), 0.89-0.87 (m, 6H); *As **33a** existed as an E/Z mixture, the carbon spectrum was included E and Z isomers*; $^{13}C$ NMR: (100 MHz, $CDCl_3$) δ 135.4, 135.1, 125.3, 124.5, 39.8, 32.4, 32.2, 32.0, 31.8, 29.74, 29.71, 29.67, 29.63, 29.4, 29.3, 28.1, 28.0, 27.6, 27.5, 23.4, 22.7, 22.5, 22.4, 15.8, 14.1, 14.0; HRMS (EI) calculated for $[C_{18}H_{36}]^+$ ($M^+$) requires m/z 252.2812, found m/z 252.2813.

**6-methylnonadec-5-ene (34a)**

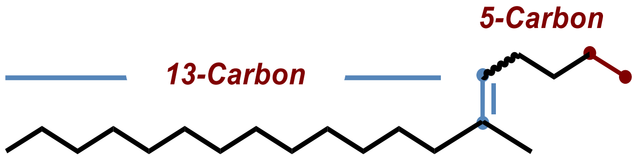


According to **method A**, 2-pentadecanone (20 mmol) and amyltriphenylphosphonium bromide (24 mmol) were used as starting materials, the reaction afforded 4.9250 g (*E* / *Z* = 1.0 / 1.3, 88% yield) of **34a** as a colorless oil. *The E/Z ratio of **34a** was inferred from previous rules (50).* $^1H$ NMR: (400 MHz, $CDCl_3$) δ 5.11 (t, $J$ = 6.8 Hz, 1H), 2.01-1.94 (m, 4H), 1.66 (s, 1.7H for *Z* isomer), 1.58 (s, 1.3H for *E* isomer), 1.37-1.26 (m, 26H), 0.89-0.86 (m, 6H); *As **34a** existed as an E/Z mixture, the carbon spectrum was included E and Z isomers*; $^{13}C$ NMR: (100 MHz, $CDCl_3$) δ 135.4, 135.1, 125.2, 124.5, 39.8, 32.4, 32.2, 32.0, 31.8,

29.73, 29.70, 29.66, 29.61, 29.4, 29.3, 28.1, 28.0, 27.6, 27.5, 23.4, 22.7, 22.45, 22.39, 15.8, 14.1, 14.0; HRMS (EI) calculated for $[C_{20}H_{40}]^+$ ($M^+$) requires m/z 280.3125, found m/z 280.3128.

**Hept-2-en-2-ylcyclohexane (35a)**

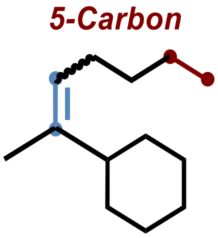


According to **method A**, 1-cyclohexylethanone (20 mmol) and amyltriphenylphosphonium bromide (30 mmol) were used as starting materials, the reaction afforded 3.02 g ($E$ / $Z$ = 9.0 / 1.0, 83% yield) of **35a** as a colorless oil. *The E/Z ratio of **35a** was inferred from previous rules (50).* $^1$H NMR: (400 MHz, $CDCl_3$) $\delta$ 5.11 (dd, $J$ = 7.2, 6.0 Hz, 0.1H for $Z$ isomer), 5.05 (t, $J$ = 7.2 Hz, 0.9H for $E$ isomer), 2.41-1.98 (m, 3H), 1.75-1.46 (m, 8H), 1.32-1.12 (m, 9H), 0.91-0.88 (m, 3H); *As **35a** existed as an E/Z mixture, the carbon spectrum was included E and Z isomers*; $^{13}$C NMR: (100 MHz, $CDCl_3$) $\delta$ 140.2, 140.0, 124.4, 122.6, 47.4, 39.6, 32.4, 32.2, 32.0, 31.1, 27.5, 26.9, 26.8, 26.7, 26.5, 26.3, 22.39, 22.37, 19.6, 14.1, 14.0; HRMS (EI) calculated for $[C_{13}H_{24}]^+$ ($M^+$) requires m/z 180.1873, found m/z 180.1873.

**6,9-dimethyltetradeca-5,9-diene (36a)**

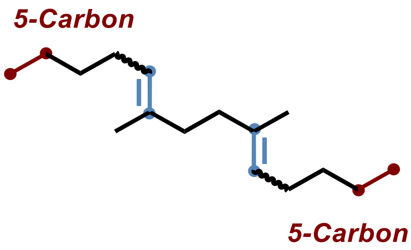


According to **method A**, 2,5-hexanedione (20 mmol) and amyltriphenylphosphonium bromide (48 mmol) were used as starting materials, the reaction afforded 1.72 g (39% yield) of **36a** as a colorless oil. $^1$H NMR: (400 MHz, $CDCl_3$) $\delta$ 5.14-5.10 (m, 2H), 2.12-1.98 (m, 7H), 1.71-1.59 (m, 6H), 1.33-1.30 (m, 9H), 0.91-0.90 (m, 6H); *As **36a** existed as an E/Z mixture, the carbon spectrum was included E and Z isomers*; $^{13}$C NMR: (100 MHz, $CDCl_3$) $\delta$ 135.2, 135.1, 134.9, 134.8, 125.5, 125.4, 124.71, 124.67, 38.4, 38.2, 32.34, 32.30, 32.1, 32.0, 30.8, 30.7, 27.63, 27.58, 27.53, 27.46, 23.50, 23.45, 22.44, 22.41, 22.35, 22.32, 16.0, 15.9, 14.0; HRMS (EI) calculated for $[C_{16}H_{30}]^+$ ($M^+$) requires m/z 222.2342, found m/z 222.2344.

1,3,3-trimethyl-2-(3-methyloct-3-en-1-yl)cyclohex-1-ene (37a)

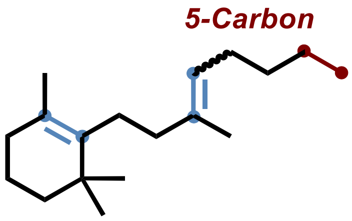


According to **method A**, dihydro-beta-ionone and amyltriphenylphosphonium bromide were used as starting materials, the reaction afforded 4.33 g (87% yield) of **37a** as a colorless oil. $^{1}$H NMR: (400 MHz, $CDCl_3$) $\delta$ 5.17-5.09 (m, 1H), 2.04-1.91 (m, 8H), 1.74-1.53 (m, 8H), 1.44-1.40 (m, 2H), 1.32-1.31 (m, 4H), 1.02-1.00 (m, 6H), 0.90-0.88 (m, 3H); *As **37a** existed as an E/Z mixture, the carbon spectrum was included E and Z isomers*; $^{13}$C NMR: (100 MHz, $CDCl_3$) $\delta$ 137.2, 135.8, 135.7, 127.0, 126.9, 125.1, 124.1, 40.3, 39.9, 35.0, 32.80, 32.77, 32.6, 32.5, 32.1, 28.6, 28.0, 27.7, 27.6, 27.4, 23.3, 22.5, 22.4, 19.8, 19.6, 16.0, 14.0; HRMS (EI) calculated for $[C_{18}H_{32}]^+$ ($M^+$), requires m/z 248.2499, found m/z 248.2500.

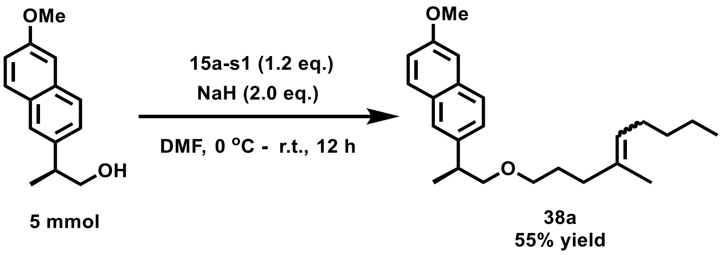


eq. S14

(*S*)-2-methoxy-6-(1-((4-methylnon-4-en-1-yl)oxy)propan-2-yl)naphthalene (38a)

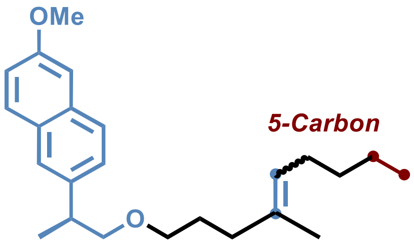


According to **the above method (*60*)**, the reaction afforded 0.98 g (55% yield) of **38a** as a colorless oil. $^{1}$H NMR: (400 MHz, $CDCl_3$) $\delta$ 7.68-7.59 (m, 3H), 7.34 (d, $J$ = 7.2 Hz, 1H), 7.12-7.10 (m, 2H), 5.13-5.05 (m, 1H), 3.89 (s, 3H), 3.62-3.58 (m, 1H), 3.49 (dd, $J$ = 9.2, 7.6 Hz, 1H), 3.39 (dd, $J$ = 6.8, 6.4 Hz, 2H), 3.17-3.12 (m, 1H), 2.04-1.92 (m, 4H), 1.65-1.56 (m, 5H), 1.38-1.27 (m, 7H), 0.88-0.86 (m, 3H); *As **38a** existed as an E/Z mixture, the carbon spectrum was included E and Z isomers*; $^{13}$C NMR: (100 MHz, $CDCl_3$) $\delta$ 157.2, 139.7, 134.4, 134.2, 133.3, 129.1, 129.0, 126.65, 126.63, 125.8, 125.3, 125.0, 118.6,

105.5, 70.7, 70.6, 55.2, 39.94, 39.88, 36.0, 32.3, 32.0, 28.1, 28.0, 27.9, 27.6, 27.4, 23.3, 22.4, 22.3, 18.4, 15.8, 14.05, 14.02; HRMS (EI) calculated for $[C_{24}H_{34}O_2]^+$ ($M^+$), requires m/z 354.2553, found m/z 354.2559.

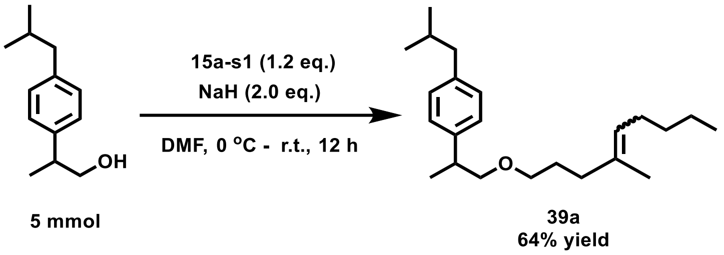


39a
64% yield

eq. S15

**1-isobutyl-4-(1-((4-methylnon-4-en-1-yl)oxy)propan-2-yl)benzene (39a)**

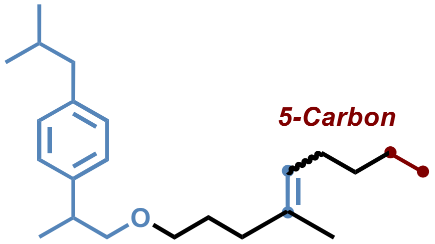


According to **the above method (*60*)**, the reaction afforded 1.05 g (64% yield) of **39a** as a colorless oil. *The E/Z ratio of **39a** was inferred from previous rules (50).* $^1$H NMR: (400 MHz, $CDCl_3$) $\delta$ 7.13 (d, $J$ = 7.6 Hz, 2H), 7.07 (d, $J$ = 8.0 Hz, 2H), 5.13-5.08 (m, 1H), 3.53-3.50 (m, 1H), 3.42-3.38 (m, 3H), 3.01-2.96 (m, 1H), 2.44 (d, $J$ = 6.8 Hz, 2H), 2.06-1.97 (m, 4H), 1.87-1.81 (m, 1H), 1.66-1.57 (m, 4H), 1.57 (s, 1H), 1.30-1.28 (m, 7H), 0.90-0.89 (m, 9H); *As **39a** existed as an E/Z mixture, the carbon spectrum was included E and Z isomers*; $^{13}$C NMR: (100 MHz, $CDCl_3$) $\delta$ 141.7, 139.5, 134.5, 134.2, 129.0, 127.0, 125.8, 125.0, 70.7, 70.5, 45.0, 39.6, 39.5, 36.0, 32.3, 32.1, 30.2, 28.2, 28.0, 27.9, 27.6, 27.5, 23.3, 22.4, 22.3, 18.3, 14.0; HRMS (EI) calculated for $[C_{23}H_{38}O]^+$ ($M^+$), requires m/z 330.2917, found m/z 330.2919.

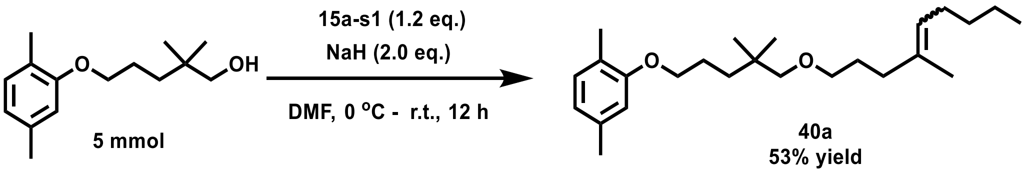


40a
53% yield

eq. S16

**2-((4,4-dimethyl-5-((4-methylnon-4-en-1-yl)oxy)pentyl)oxy)-1,4-dimethylbenzene (40a)**

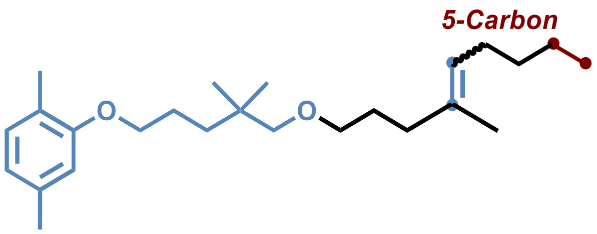


According to **the above method (*60*)**, the reaction afforded 0.99 g of **40a** (53% yield) as a colorless oil. $^{1}$H NMR: (400 MHz, $CDCl_3$) $\delta$ 7.00 (d, $J$ = 7.2 Hz, 1H), 6.66-6.62 (m, 2H), 5.13 (t, $J$ = 6.4 Hz, 1H), 3.91 (dd, $J$ = 6.4, 6.0 Hz, 2H), 3.42-3.32 (m, 2H), 3.10 (s, 2H), 2.30 (s, 3H), 2.18 (s, 3H), 2.10-1.97 (m, 4H), 1.76-1.56 (m, 7H), 1.43-1.29 (m, 6H), 0.97-0.81 (m, 9H); *As **40a** existed as an E/Z mixture, the carbon spectrum was included E and Z isomers*; $^{13}$C NMR: (100 MHz, $CDCl_3$) $\delta$ 157.1, 136.4, 134.7, 134.4, 130.2, 125.7, 124.9, 123.6, 120.5, 112.0, 79.7, 71.1, 71.0, 68.7, 36.1, 35.49, 35.46, 34.3, 32.3, 32.1, 28.2, 28.1, 28.0, 27.6, 27.5, 24.6, 24.2, 23.4, 22.4, 22.3, 21.4, 15.9, 15.8, 14.0; HRMS (EI) calculated for $[C_{25}H_{42}O_2]^+$ ($M^+$) requires m/z 374.3179, found m/z 374.3182.

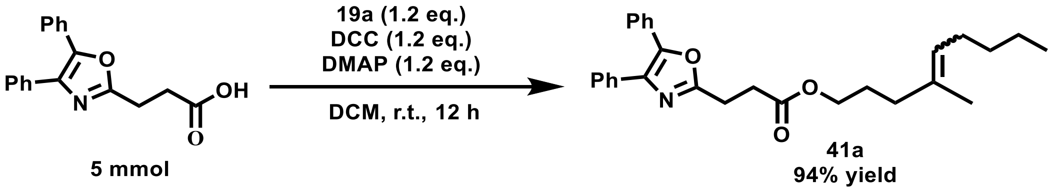


eq. S17

**<u>4-methylnon-4-en-1-yl 3-(4,5-diphenyloxazol-2-yl)propanoate (41a)</u>**

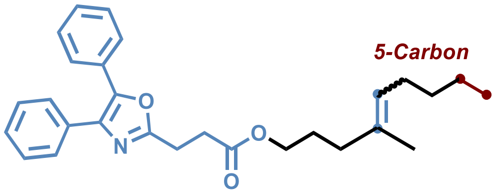


According to **the above method (*61*)**, the reaction afforded 2.03 g of **41a** (*E* / *Z* = 1.0 / 2.0, 94% yield) as a colorless oil. *The E/Z ratio of **41a** was inferred from previous rules (50).* $^{1}$H NMR: (400 MHz, $CDCl_3$) $\delta$ 7.63 (d, $J$ = 7.2 Hz, 2H), 7.57 (d, $J$ = 7.2 Hz, 2H), 7.35-7.32 (m, 6H), 5.18-5.10 (m, 1H), 4.10 (t, $J$ = 6.4 Hz, 2H), 3.19 (dd, $J$ = 7.6, 7.2 Hz, 2H), 2.91 (t, $J$ = 7.6 Hz, 2H), 2.09-1.96 (m, 4H), 1.75-1.70 (m, 2H), 1.66 (s, 2H for *Z* isomer), 1.57 (s, 1H for *E* isomer), 1.38-1.18 (m, 4H), 0.88 (dd, $J$ = 6.0, 5.6 Hz, 3H); *As **41a** existed as an E/Z mixture, the carbon spectrum was included E and Z isomers*; $^{13}$C NMR: (100 MHz, $CDCl_3$) $\delta$ 172.0, 161.7, 161.7, 145.3, 135.1, 133.4, 133.3, 132.4, 128.9, 128.6, 128.5, 128.4, 128.0, 127.8, 126.5, 126.4, 125.6, 64.52, 64.47, 35.7, 32.1, 31.9, 31.1, 27.8, 27.5, 27.4, 26.8, 26.7, 23.5,

23.1, 22.4, 22.3, 15.7, 14.0; HRMS (EI) calculated for $[C_{28}H_{33}NO_3]^+$ ($M^+$) requires m/z 431.2455, found m/z 431.2459.

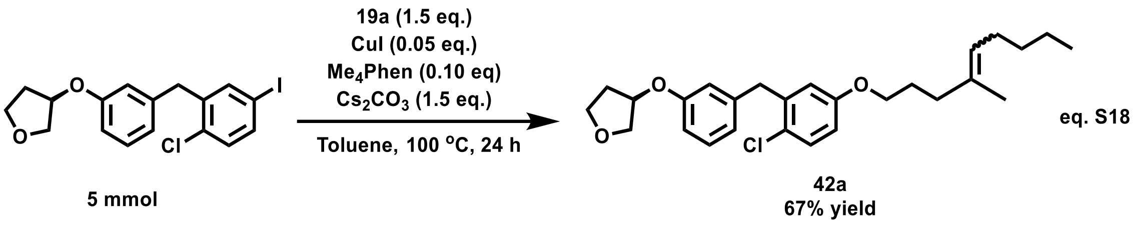


<u>**3-(3-(2-chloro-5-((4-methylnon-4-en-1-yl)oxy)benzyl)phenoxy)tetrahydrofuran (42a)**</u>

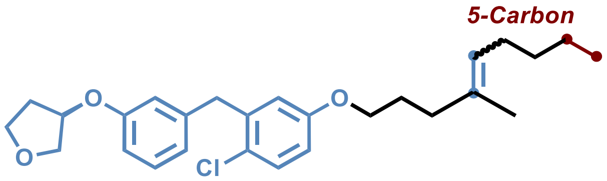


According to **the above method (*62*)**, the reaction afforded 1.43 g of **42a** (*E* / *Z* = 1.0 / 2.0, 67% yield) as a colorless oil. *The E/Z ratio of* ***42a*** *was inferred from previous rules (50).* $^1$H NMR: (400 MHz, $CDCl_3$) *δ* 7.22 (d, *J* = 5.2 Hz, 1H), 7.09 (d, *J* = 8.0 Hz, 2H), 6.78-6.67 (m, 4H), 5.18-5.14 (m, 1H), 4.93-4.77 (m, 1H), 3.97-3.81 (m, 8H), 2.20-2.08 (m, 4H), 1.98-1.79 (m, 4H), 1.68 (s, 2H for *Z* isomer), 1.60 (s, 1H for *E* isomer), 1.28-1.19 (m, 4H), 0.87-0.84 (m, 3H); *As* ***42a*** *existed as an E/Z mixture, the carbon spectrum was included E and Z isomers*; $^{13}$C NMR: (100 MHz, $CDCl_3$) 157.8, 157.7, 155.8, 139.8, 133.54, 133.49, 131.7, 129.9, 126.5, 125.5, 125.2, 117.15, 117.07, 115.2, 113.2, 77.1, 73.0, 67.5, 67.3, 67.1, 38.4, 35.7, 32.9, 32.1, 31.9, 27.7, 27.5, 27.4, 27.24, 27.18, 23.2, 22.3, 22.2, 15.8, 13.9; HRMS (EI) calculated for $[C_{27}H_{35}ClO_3]^+$ ($M^+$) requires m/z 442.2269, found m/z 442.2276.

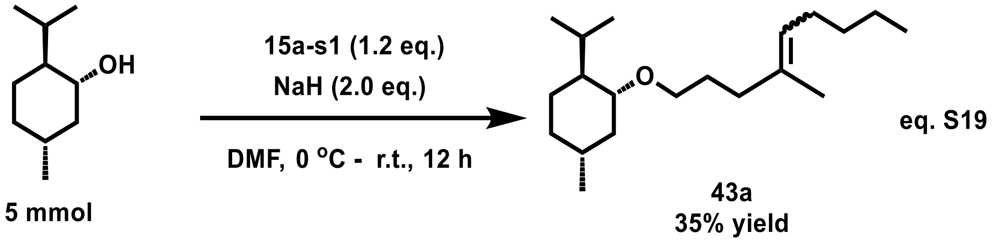


<u>**(1S,2S)-1-isopropyl-4-methyl-2-((4-methylnon-4-en-1-yl)oxy)cyclohexane (43a)**</u>

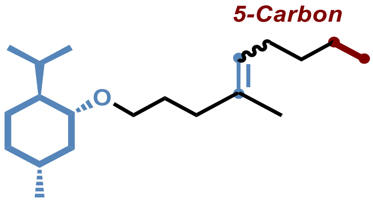

According to **the above method (*60*)**, the reaction afforded 0.5184 g of **43a** (35% yield) as a colorless oil. $^1$H NMR: (400 MHz, $CDCl_3$) $\delta$ 5.14-5.11 (m, 1H), 3.64-3.56 (m, 1H), 3.37-2.97 (m, 2H), 2.25-2.22 (m, 1H), 2.10-1.97 (m, 5H), 1.68-1.56 (m, 8H), 1.30-1.19 (m, 6H), 0.92-0.77 (m, 14H); *As **43a** existed as an E/Z mixture, the carbon spectrum was included E and Z isomers*; $^{13}$C NMR: (100 MHz, $CDCl_3$) $\delta$ 134.7, 134.4, 125.6, 124.9, 79.2, 79.1, 68.5, 68.1, 48.3, 40.6, 40.5, 36.2, 34.6, 32.3, 32.1, 31.6, 28.8, 28.5, 28.5, 27.6, 27.5, 25.6, 25.5, 23.4, 23.4, 23.3, 22.41, 22.36, 22.34, 21.0, 20.9, 16.3, 16.2, 15.8, 14.0; HRMS (EI) calculated for $[C_{20}H_{38}O]^+$ ($M^+$) requires m/z 294.2917, found m/z 294.2919.

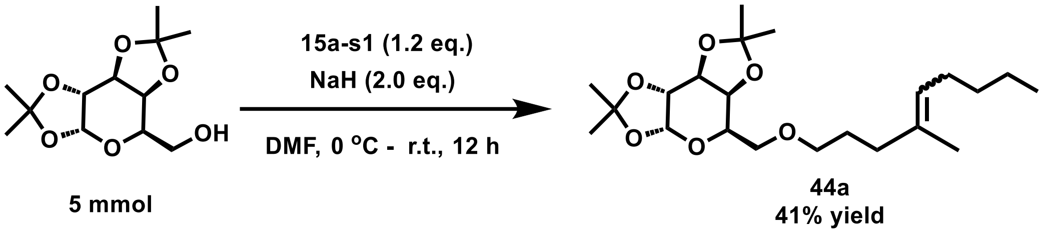


eq. S20

**(3aR,5R,5aS,8aS,8bR)-2,2,7,7-tetramethyl-5-(((4-methylnon-4-en-1-yl)oxy)methyl) tetrahydro-5H-bis([1,3]dioxolo)[4,5-b:4',5'-d]pyran (44a)**

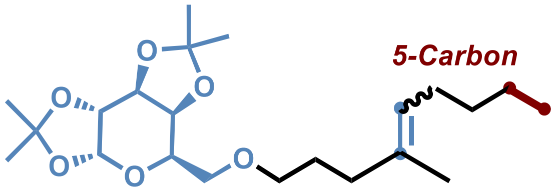


According to **the above method (*60*)**, the reaction afforded 0.81 g of **44a** (41% yield) as a colorless oil. $^1$H NMR: (400 MHz, $CDCl_3$) $\delta$ 5.54 (d, $J$ = 4.4 Hz, 1H), 5.13 (dd, $J$ = 7.2, 6.4 Hz, 1H), 4.60 (d, $J$ = 7.6 Hz, 1H), 4.31-4.26 (m, 2H), 4.02-3.91 (m, 1H), 3.64-3.45 (m, 4H), 2.08-1.96 (m, 4H), 1.70-1.59 (m, 5H), 1.54 (s, 3H), 1.45 (s, 3H), 1.34-1.29 (m, 10H), 0.97-0.80 (m, 3H); *As **44a** existed as an E/Z mixture, the carbon spectrum was included E and Z isomers*; $^{13}$C NMR: (100 MHz, $CDCl_3$) $\delta$ 134.3, 134.1, 125.7, 124.9, 109.1, 108.4, 96.3, 71.13, 71.09, 71.04, 70.97, 70.5, 69.23, 69.17, 66.64, 66.57, 35.8, 32.2, 32.0, 28.0, 27.8, 27.5, 27.3, 26.0, 25.9, 24.8, 24.3, 23.2, 22.3, 22.2, 15.8, 13.95, 13.93; HRMS (ESI) calculated for $[C_{22}H_{38}NaO_6]^+$ ($M + Na^+$) requires m/z 421.2561, found m/z 421.2563.

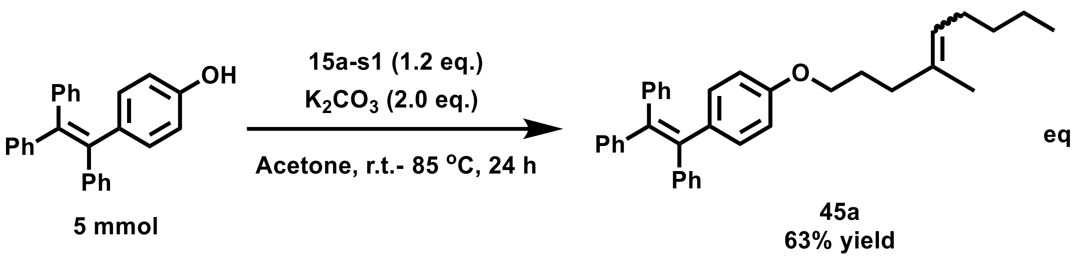


eq. S21

<u>**(2-(4-((4-methylnon-4-en-1-yl)oxy)phenyl)ethene-1,1,2-triyl)tribenzene (45a)**</u>

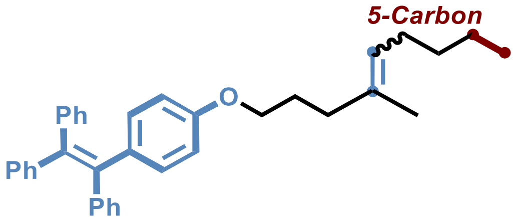


According to **the above method (*63*)**, the reaction afforded 1.5413 g of **45a** (*E* / *Z* = 1.0 / 2.0, 63% yield) as a yellow oil. *The E/Z ratio of **45a** was inferred from previous rules (50).* $^{1}$H NMR: (400 MHz, $CDCl_3$) $\delta$ 7.11-7.00 (m, 15H), 6.91 (d, $J$ = 8.4 Hz, 2H), 6.61 (d, $J$ = 8.4 Hz, 2H), 5.18-5.15 (m, 1H), 3.84 (dd, $J$ = 6.4, 6.0 Hz, 2H), 2.18-1.80 (m, 6H), 1.68 (s, 2H for *Z* isomer), 1.60 (s, 1H for *E* isomer), 1.29-1.26 (m, 4H), 0.87-0.86 (m, 3H); *As **45a** existed as an E/Z mixture, the carbon spectrum was included E and Z isomers*; $^{13}$C NMR: (100 MHz, $CDCl_3$) $\delta$ 157.63, 157.56, 144.04, 143.99, 140.6, 139.9, 135.9, 135.8, 133.8, 132.5, 131.4, 131.34, 131.31, 127.7, 127.6, 127.5, 126.5, 126.3, 126.2, 125.4, 113.54, 113.50, 67.3, 67.0, 35.9, 32.2, 32.0, 27.9, 27.6, 27.5, 27.43, 27.40, 23.2, 22.4, 22.3, 15.9, 14.0; HRMS (EI) calculated for $[C_{36}H_{38}O]^+$ ($M^+$) requires m/z 486.2917, found m/z 486.2923.

# IV. Reaction Optimization

## Figure S4. Selective Hydroboration of 1a

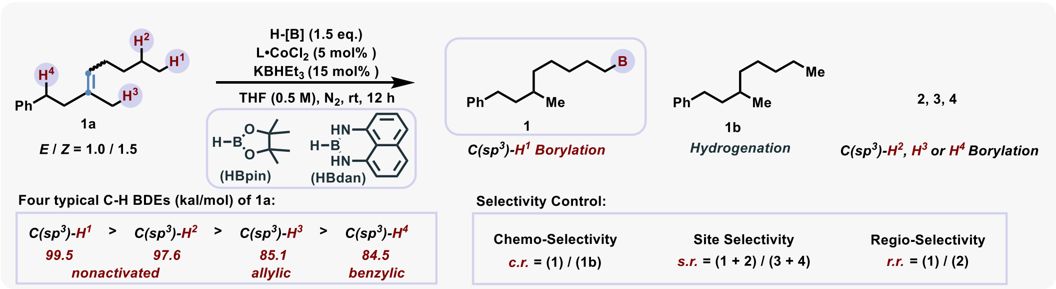


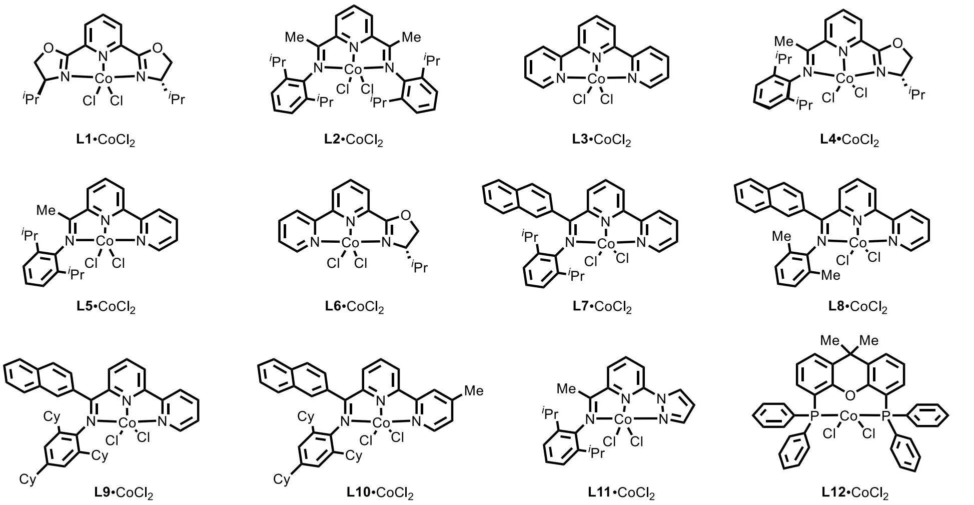


| Entry[a] | L•$CoCl_2$ | Conv. (%)[b] | Yield of **1**[b] (%) | Chemo Selectivity[b] | Site Selectivity[b] | Regio Selectivity[b] |
|---|---|---|---|---|---|---|
| 1 | **L1**•$CoCl_2$ | 36 | Trace | <5/95 | / | / |
| 2 | **L2**•$CoCl_2$ | 31 | Trace | <5/95 | / | / |
| 3 | **L3**•$CoCl_2$ | 50 | 10 | >95/5 | 80/20 | 25/75 |
| 4 | **L4**•$CoCl_2$ | 89 | 22 | 50/50 | 75/25 | 66/33 |
| 5 | **L5**•$CoCl_2$ | >99 | 50 | >95/5 | >95/5 | 50/50 |
| 6 | **L6**•$CoCl_2$ | 17 | Trace | <5/95 | / | / |
| 7 | **L7**•$CoCl_2$ | >99 | 75 | >95/5 | >95/5 | 75/25 |
| 8 | **L8**•$CoCl_2$ | >99 | 66 | >95/5 | >95/5 | 66/33 |
| 9 | **L9**•$CoCl_2$ | >99 | 92 | >95/5 | >95/5 | 92/8 |
| 9[c] | **L9**•$CoCl_2$ | >99 | 93 | >95/5 | >95/5 | 93/7 |
| 9[c,d] | **L9•$CoCl_2$** | >99 | **>99(98)** | **>95/5** | **>95/5** | **>95/5** |
| 10[c] | **L10**•$CoCl_2$ | >99 | 96 | >95/5 | >95/5 | >95/5 |
| 11 | **L11**•$CoCl_2$ | 65 | 30 | >95/5 | >95/5 | 50/50 |
| 12 | **L12**•$CoCl_2$ | Trace | Trace | / | / | / |

[a] The reaction was conducted using **1a** (0.25 mmol), HBpin (0.375 mmol), cobalt complex (0.0125 mmol), and additives (3 equiv. to cobalt precatalyst) in THF at room temperature in golvebox for 12 h. [b] Determined by $^1$H NMR using phenyltrimethylsilane as an internal standard after conversion to the corresponding alcohol [c] cobalt complex (0.005 mmol). [d] HBdan (0.375 mmol) as boron source.

## V. Precise Borylation of Targeted Methyl Group via an Orderly Chain-Walking Strategy

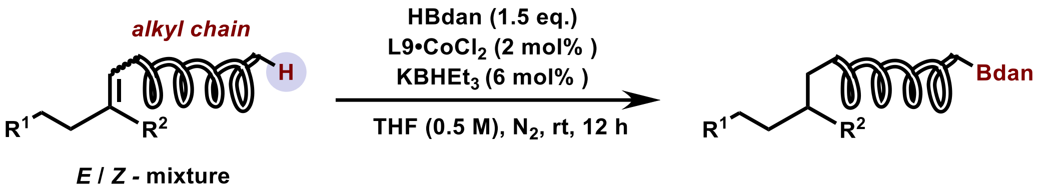


eq. S22

**General procedure** for precise borylation of targeted methyl group via an orderly chain-walking strategy: In the nitrogen filled glove box, a 10 mL flame-dried flask was cooled at room temperature under nitrogen, charged with **L9**•$CoCl_2$ (0.0038 g, 0.005 mmol, 0.02 equiv.), HBdan (0.0630 g, 0.375 mmol, 1.5 equiv.), then alkene (0.25 mmol, 1.0 equiv.), dry THF (0.5 mL, 0.5 M), then $KBHEt_3$ (15 μL, 1M in THF, 0.015 mmol, 0.06 equiv.) were added successively. The mixture was kept under room temperature and stirred for 12 h. The reaction was quenched by PE. The mixture was filtered through a pad of silica gel and washed with ether (50 mL). NMR yield was monitored by $^1$H NMR analysis using phenyltrimethylsilane as internal standard. The resulting mixture was purified by flash column chromatography on silica gel using PE and EA as the eluent to give the corresponding product.

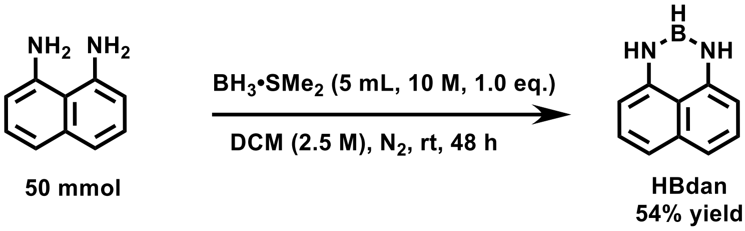


eq. S23

HBdan was prepared according to the general procedure after modification: to a solution of 1,8-diaminonaphthalene (7.91 g, 50 mmol) in anhydrous DCM (25 mL) at 0°C was added $BH_3 \cdot SMe_2$ (5.0 mL, 50 mmol) dropwise over 30 min. The reaction mixture was stirred for 48 h at room temperature. The crude product was isolated

through the evaporation of volatiles in vacuo, then the resulting mixture was purified by flash column chromatography on silica gel using (PE/EA = 100/3 (v/v) as the eluent to give the corresponding product as a white solid (4.55 g, 54% yield). $^1$H NMR: (400 MHz, $CDCl_3$) $\delta$ 7.09-6.99 (m, 4H), 6.26 (d, $J$ = 7.2 Hz, 2H), 5.79 (brs, 2H), 4.04 (brs, 1H); *Analytical data were in accordance with those previously reported (64).*

**2-(6-methyl-8-phenyloctyl)-2,3-dihydro-1*H*-naphtho[1,8-*de*][1,3,2]diazaborinine (1)**

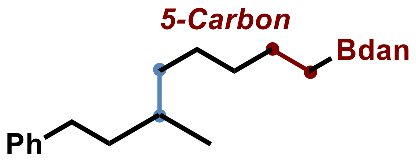


Prepared according to the general procedure: using **L9**•$CoCl_2$ (0.0041 g, 0.0054 mmol), HBdan (0.0620 g, 0.369 mmol), **1a** (0.0512 g, 0.253 mmol), 0.5 mL of THF and $KBHEt_3$ (15 µL, 1M in THF, 0.0150 mmol), stir at r.t. for 12 hours, the residue was purified by preparative TLC (PE/EA/TEA = 100/5/2 (v/v/v), $R_f$ = 0.40) to afford **1** (0.0920 g, 98% yield) as a colorless oil. $^1$H NMR: (400 MHz, $CDCl_3$) $\delta$ 7.28-7.16 (m, 5H), 7.10-6.98 (m, 4H), 6.27 (d, $J$ = 6.8 Hz, 2H), 5.56 (brs, 2H), 2.67-2.53 (m, 2H), 1.68-1.55 (m, 1H), 1.50-1.16 (m, 10H), 0.93 (d, $J$ = 4.4 Hz, 3H), 0.83 (dd, $J$ = 7.6, 7.2 Hz, 2H); $^{13}$C NMR: (100 MHz, $CDCl_3$) $\delta$ 143.1, 141.2, 136.3, 128.3, 128.2, 127.5, 125.5, 119.5, 117.3, 105.4, 38.9, 36.8, 33.5, 32.8, 32.5, 26.8, 24.8, 19.6 *the boron-bound carbons were not detected due to quadrupolar relaxation*; IR (cm$^{-1}$): 2926, 2851, 1601, 1509, 1456, 1413 cm$^{-1}$; HRMS (EI) calculated for $[C_{25}H_{31}BN_2]^+$ ($M^+$) requires m/z 370.2575, found m/z 370.2576.

**2-(3-methyl-5-phenylpentyl)-2,3-dihydro-1*H*-naphtho[1,8-*de*][1,3,2]diazaborinine (5)**

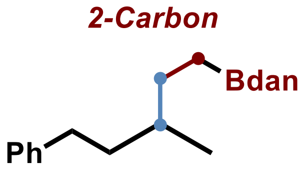


Prepared according to the general procedure: using **L9**•$CoCl_2$ (0.0044 g, 0.0058 mmol), HBdan (0.0648 g, 0.386 mmol), **5a** (0.0406 g, 0.254 mmol), 0.5 mL of THF and $KBHEt_3$ (15 µL, 1M in THF, 0.0150 mmol), stir at r.t. for 12 hours, the residue was purified by preparative TLC (PE/EA/TEA = 100/11/2 (v/v/v), $R_f$ = 0.70) to afford **5** (0.0823 g, 99% yield) as a colorless oil. $^1$H NMR: (400 MHz, $CDCl_3$) $\delta$ 7.29-7.16 (m, 5H), 7.10-6.98 (m, 4H), 6.27 (d, $J$ = 7.2 Hz, 2H), 5.55 (brs, 2H), 2.70-2.54 (m, 2H), 1.71-

1.66 (m, 1H), 1.51-1.38 (m, 3H), 1.30-1.19 (m, 1H), 0.97 (d, $J$ = 3.6 Hz, 3H), 0.90-0.75 (m, 2H); $^{13}$C NMR: (100 MHz, $CDCl_3$) $\delta$ 143.0, 141.1, 136.3, 128.33, 128.27, 127.5, 125.6, 119.5, 117.3, 105.4, 38.5, 34.5, 33.4, 31.7, 19.3 *the boron-bound carbons were not detected due to quadrupolar relaxation*; IR ($cm^{-1}$): 2918, 2858, 1601, 1508, 1413, 1372 $cm^{-1}$; HRMS (EI) calculated for $[C_{22}H_{25}BN_2]^+$ ($M^+$) requires m/z 328.2105, found m/z 328.2107.

**2-(6-(methyl-d$_3$)-8-phenyloctyl-7,7-d$_2$)-2,3-dihydro-1*H*-naphtho[1,8-*de*][1,3,2]diazaborinine (6)**

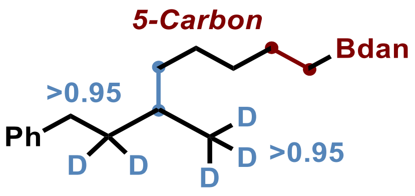


Prepared according to the general procedure: using **L9**•$CoCl_2$ (0.0038 g, 0.0050 mmol), HBdan (0.0640 g, 0.381 mmol), **6a** (0.0520 g, 0.251 mmol), 0.5 mL of THF and $KBHEt_3$ (15 μL, 1M in THF, 0.0150 mmol), stir at r.t. for 12 hours, the residue was purified by preparative TLC (PE/EA/TEA = 100/5/2 (v/v/v), $R_f$ = 0.40) to afford **6** (0.0903 g, 96% yield) as a colorless oil. $^1$H NMR: (400 MHz, $CDCl_3$) $\delta$ 7.29-7.15 (m, 5H), 7.10-6.98 (m, 4H), 6.28 (d, $J$ = 7.2 Hz, 2H), 5.58 (brs, 2H), 2.65-2.52 (m, 2H), 1.42-1.22 (m, 8H), 1.16-1.12 (m, 1H), 0.84 (dd, $J$ = 8.0, 7.2 Hz, 2H); $^2$H NMR: (92 MHz, $CDCl_3$) $\delta$ 1.66-1.45 (m, 1.92D), 0.93 (s, 2.86D); $^{13}$C NMR: (100 MHz, $CDCl_3$) $\delta$ 143.2, 141.2, 136.3, 128.3, 128.2, 127.5, 125.5, 119.5, 117.3, 105.4, 36.7, 33.3, 32.8, 32.1, 26.8, 24.8 *the boron-bound carbons were not detected due to quadrupolar relaxation*; IR ($cm^{-1}$): 2920, 2853, 1601, 1505, 1412, 1371 $cm^{-1}$; HRMS (EI) calculated for $[C_{25}H_{26}D_5BN_2]^+$ ($M^+$) requires m/z 375.2889, found m/z 375.2890.

**2-(13-methyl-15-phenylpentadecyl)-2,3-dihydro-1*H*-naphtho[1,8-*de*][1,3,2]diazaborinine (7)**

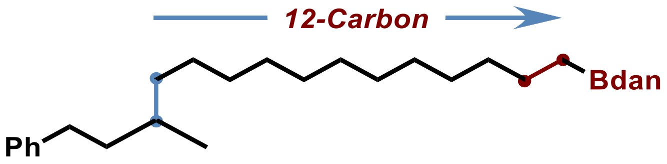


Prepared according to the general procedure: using **L9**•$CoCl_2$ (0.0045 g, 0.0059 mmol), HBdan (0.0635 g, 0.378 mmol), **7a** (0.0752 g, 0.250 mmol), 0.5 mL of THF and $KBHEt_3$ (15 μL, 1M in THF, 0.0150 mmol), stir at r.t. for 12 hours, the residue was purified by preparative TLC (PE/EA/TEA = 100/3/2 (v/v/v), $R_f$ = 0.40) to afford **7** (0.1040 g, 89% yield) as a colorless oil. $^1$H NMR: (400 MHz, $CDCl_3$) $\delta$

7.28-7.14 (m, 5H), 7.10-6.97 (m, 4H), 6.26 (d, $J$ = 7.2 Hz, 2H), 5.56 (brs, 2H), 2.67-2.51 (m, 2H), 1.65-1.58 (m, 1H), 1.43-1.13 (m, 24H), 0.91 (d, $J$ = 6.4 Hz, 3H), 0.83 (dd, $J$ = 8.0, 7.6 Hz, 2H); $^{13}$C NMR: (100 MHz, $CDCl_3$) $\delta$ 143.2, 141.2, 136.3, 128.3, 128.2, 127.5, 125.5, 119.5, 117.3, 105.4, 38.9, 36.9, 33.5, 32.5, 30.0, 29.7, 29.6, 29.5, 27.0, 24.8, 19.6 *the boron-bound carbons were not detected due to quadrupolar relaxation*; IR ($cm^{-1}$): 2922, 2853, 1601, 1505, 1412, 1371 $cm^{-1}$; HRMS (EI) calculated for $[C_{32}H_{45}BN_2]^+$ ($M^+$) requires m/z 468.3670, found m/z 468.3673.

**2-(17-methyl-19-phenylnonadecyl)-2,3-dihydro-1*H*-naphtho[1,8-*de*][1,3,2]diazaborinine (8)**

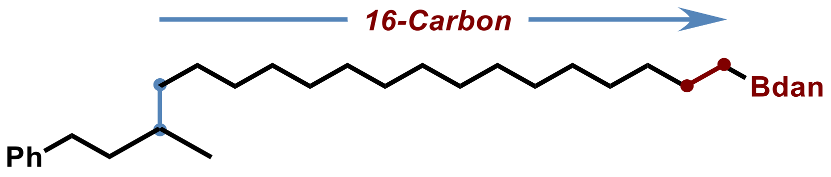


Prepared according to the general procedure: using **L9**•$CoCl_2$ (0.0042 g, 0.0055 mmol), HBdan (0.0650 g, 0.387 mmol), **8a** (0.0892 g, 0.250 mmol), 0.5 mL of THF and $KBHEt_3$ (15 µL, 1M in THF, 0.0150 mmol), stir at r.t. for 12 hours, the residue was purified by preparative TLC (PE/EA/TEA = 100/6/2 (v/v/v), $R_f$ = 0.60) to afford **8** (0.1222 g, 93% yield) as a colorless oil. $^1$H NMR: (400 MHz, $CDCl_3$) $\delta$ 7.28-7.14 (m, 5H), 7.10-6.98 (m, 4H), 6.27 (d, $J$ = 7.2 Hz, 2H), 5.58 (brs, 2H), 2.68-2.51 (m, 2H), 1.63-1.59 (m, 1H), 1.43-1.13 (m, 32H), 0.91 (d, $J$ = 6.0 Hz, 3H), 0.84 (dd, $J$ = 8.0, 7.6 Hz, 2H); $^{13}$C NMR: (100 MHz, $CDCl_3$) $\delta$ 143.2, 141.2, 136.3, 128.3, 128.2, 127.5, 125.5, 119.5, 117.3, 105.3, 38.9, 36.9, 33.5, 32.5, 30.0, 29.7, 29.6, 29.5, 27.0, 24.8, 19.6 *the boron-bound carbons were not detected due to quadrupolar relaxation*; IR ($cm^{-1}$): 2921, 2852, 1730, 1602, 1505, 1411 $cm^{-1}$; HRMS (ESI) calculated for $[C_{36}H_{54}BN_2]^+$ ($M + H^+$) requires m/z 525.4375, found m/z 525.4375.

**2-(23-methyl-25-phenylpentacosyl)-2,3-dihydro-1*H*-naphtho[1,8-*de*][1,3,2]diazaborinine (9)**

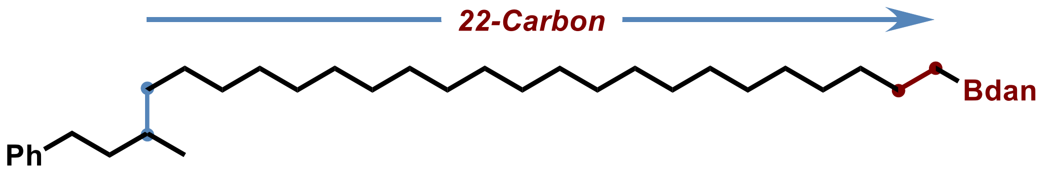


Prepared according to the general procedure: using **L9**•$CoCl_2$ (0.0041 g, 0.0054 mmol), HBdan (0.0641 g, 0.382 mmol), **9a** (0.1101 g, 0.250 mmol), 0.5 mL of THF and $KBHEt_3$ (15 µL, 1M in THF, 0.0150 mmol), stir at r.t. for 12 hours, the crude product was purified by column chromatography on silica gel

using PE/TEA =100/2 (v/v) as the eluent to afford **9** (0.1456 g, 96% yield) as a white solid. $^{1}$H NMR: (400 MHz, $CDCl_3$) $\delta$ 7.28-7.13 (m, 5H), 7.09-6.97 (m, 4H), 6.26 (d, $J$ = 7.2 Hz, 2H), 5.55 (brs, 2H), 2.67-2.51 (m, 2H), 1.65-1.58 (m, 1H), 1.44-1.12 (m, 44H), 0.91 (d, $J$ = 6.0 Hz, 3H), 0.82 (dd, $J$ = 8.0, 7.6 Hz, 2H); $^{13}$C NMR: (100 MHz, $CDCl_3$) $\delta$ 143.2, 141.2, 136.3, 128.3, 128.2, 127.5, 125.5, 119.5, 117.3, 105.4, 38.9, 36.9, 33.5, 32.51, 32.49, 30.0, 29.7, 29.6, 29.5, 27.0, 24.8, 19.6 *the boron-bound carbons were not detected due to quadrupolar relaxation*; IR (cm$^{-1}$): 2921, 2851, 1601, 1505, 1411, 1372 cm$^{-1}$; HRMS (ESI) calculated for $[C_{42}H_{66}BN_2]^+$ (M + H$^+$) requires m/z 609.5314, found m/z 609.5316.

**2-(31-methyl-33-phenyltritriacontyl)-2,3-dihydro-1*H*-naphtho[1,8-*de*][1,3,2]diazaborinine (10)**

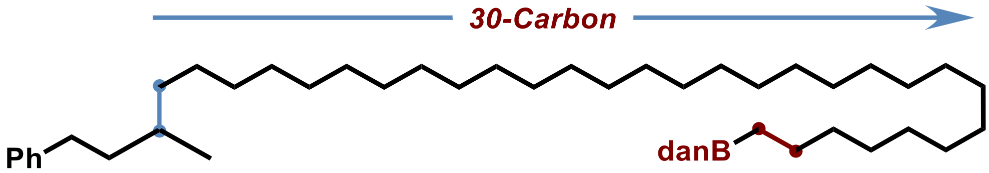


Prepared according to the general procedure: using **L9**•$CoCl_2$ (0.0047 g, 0.0062 mmol), HBdan (0.0650 g, 0.387 mmol), **10a** (0.1386 g, 0.251 mmol), 1.0 mL of THF and $KBHEt_3$ (15 µL, 1M in THF, 0.0150 mmol), stir at r.t. for 12 hours, the crude product was purified by column chromatography on silica gel using PE/TEA =100/2 (v/v) as the eluent to afford **10** (0.1733 g, 96% yield) as a white solid. $^{1}$H NMR: (400 MHz, $CDCl_3$) $\delta$ 7.28-7.14 (m, 5H), 7.10-6.97 (m, 4H), 6.27 (d, $J$ = 7.2 Hz, 2H), 5.57 (brs, 2H), 2.70-2.48 (m, 2H), 1.65-1.59 (m, 1H), 1.42-1.13 (m, 60H), 0.91 (d, $J$ = 5.6 Hz, 3H), 0.84 (dd, $J$ = 8.0, 7.6 Hz, 2H); $^{13}$C NMR: (100 MHz, $CDCl_3$) $\delta$ 143.2, 141.2, 136.3, 128.3, 128.2, 127.5, 125.5, 119.5, 117.3, 105.4, 39.0, 36.9, 33.5, 32.5, 30.0, 29.7, 29.6, 29.5, 27.0, 24.8, 19.6 *the boron-bound carbons were not detected due to quadrupolar relaxation*; IR (cm$^{-1}$): 2921, 2854, 1602, 1504, 1458, 1410 cm$^{-1}$; HRMS (ESI) calculated for $[C_{50}H_{82}BN_2]^+$ (M + H$^+$) requires m/z 721.6566, found m/z 721.6570.

**2-(8-(4-methoxyphenyl)-6-methyloctyl)-2,3-dihydro-1*H*-naphtho[1,8-*de*][1,3,2]diazaborinine (11)**

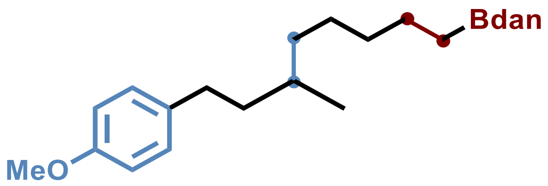


Prepared according to the general procedure: using **L9**•$CoCl_2$ (0.0043 g, 0.0057 mmol), HBdan (0.0653 g, 0.389 mmol), **11a** (0.0582 g, 0.251 mmol), 0.5 mL of THF and $KBHEt_3$ (15 µL, 1M in THF, 0.0150

mmol), stir at r.t. for 12 hours, the residue was purified by preparative TLC (PE/EA/TEA = 100/20/2 (v/v/v), $R_f$ = 0.60) to afford **11** (0.0932 g, 93% yield) as a colorless oil. $^1$H NMR: (400 MHz, $CDCl_3$) $\delta$ 7.10-6.97 (m, 6H), 6.80 (d, $J$ = 8.4 Hz, 2H), 6.26 (d, $J$ = 6.8 Hz, 2H), 5.57 (brs, 2H), 3.76 (s, 3H), 2.62-2.46 (m, 2H), 1.62-1.11 (m, 11H), 0.91 (d, $J$ = 6.4 Hz, 3H), 0.81 (dd, $J$ = 8.0, 7.6 Hz, 2H); $^{13}$C NMR: (100 MHz, $CDCl_3$) $\delta$ 157.5, 141.2, 136.3, 135.2, 129.1, 127.5, 119.5, 117.2, 113.6, 105.3, 55.2, 39.1, 36.8, 32.8, 32.5, 32.3, 26.8, 24.8, 19.6 *the boron-bound carbons were not detected due to quadrupolar relaxation*; IR (cm$^{-1}$): 2921, 2853, 1602, 1508, 1458, 1411 cm$^{-1}$; HRMS (EI) calculated for $[C_{26}H_{33}BN_2O]^+$ ($M^+$) requires m/z 400.2680, found m/z 400.2685.

**2-(7-(4-bromophenyl)-6-methylheptyl)-2,3-dihydro-1*H*-naphtho[1,8-*de*][1,3,2]diazaborinine (12)**

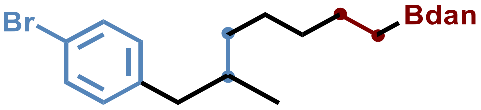


Prepared according to the general procedure: using **L9**•$CoCl_2$ (0.0040 g, 0.0053 mmol), HBdan (0.0653 g, 0.389 mmol), **12a** (0.0701 g, 0.264 mmol), 0.5 mL of THF and $KBHEt_3$ (15 μL, 1M in THF, 0.0150 mmol), stir at r.t. for 12 hours, the residue was purified by preparative TLC (PE/EA/TEA = 100/10/2 (v/v/v), $R_f$ = 0.50) to afford **12** (0.1019 g, 89% yield) as a colorless oil. $^1$H NMR: (400 MHz, $CDCl_3$) $\delta$ 7.37 (d, $J$ = 8.0 Hz, 2H), 7.08 (dd, $J$ = 8.0, 7.6 Hz, 2H), 7.00-6.98 (m, 4H), 6.28 (d, $J$ = 7.2 Hz, 2H), 5.57 (brs, 2H), 2.57 (dd, $J$ = 13.2, 6.0 Hz, 1H), 2.30 (dd, $J$ = 13.2, 8.0 Hz, 1H), 1.71-1.63 (m, 1H), 1.42-1.10 (m, 8H), 0.87-0.77 (m, 5H); $^{13}$C NMR: (100 MHz, $CDCl_3$) $\delta$ 141.2, 140.5, 136.3, 131.1, 130.9, 127.5, 119.5, 119.3, 117.3, 105.4, 43.0, 36.5, 34.9, 32.6, 26.9, 24.8, 19.3 *the boron-bound carbons were not detected due to quadrupolar relaxation*; IR (cm$^{-1}$): 2977, 2922, 1602, 1509, 1456, 1410 cm$^{-1}$; HRMS (EI) calculated for $[C_{24}H_{28}BBrN_2]^+$ ($M^+$) requires m/z 434.1523, found m/z 434.1525.

**2-(6-methyl-8-(thiophen-2-yl)octyl)-2,3-dihydro-1*H*-naphtho[1,8-*de*][1,3,2]diazaborinine (13)**

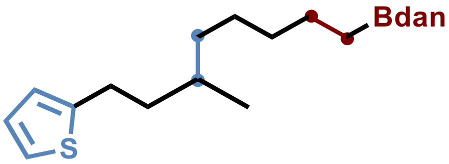


Prepared according to the general procedure after modification: using **L9**•$CoCl_2$ (0.0079 g, 0.010 mmol), HBdan (0.0638 g, 0.380 mmol), **13a** (0.0519 g, 0.250 mmol), 0.5 mL of THF and $KBHEt_3$ (30 μL, 1M in THF, 0.0150 mmol), stir at r.t. for 12 hours, the residue was purified by preparative TLC (PE/EA/ TEA

= 100/10/2 (v/v/v), $R_f$ = 0.60) to afford **13** (0.0737 g, 78% yield) as a colorless oil. $^1$H NMR: (400 MHz, $CDCl_3$) $\delta$ 7.10-7.06 (m, 3H), 6.99 (d, $J$ = 8.0 Hz, 2H), 6.90 (dd, $J$ = 4.8, 3.6 Hz, 1H), 6.77 (d, $J$ = 2.4 Hz, 1H), 6.28 (d, $J$ = 7.2 Hz, 2H), 5.58 (brs, 2H), 2.90-2.75 (m, 2H), 1.73-1.65 (m, 1H), 1.53-1.17 (m, 10H), 0.92 (d, $J$ = 6.0 Hz, 3H), 0.84 (dd, $J$ = 8.0, 7.6 Hz, 2H); $^{13}$C NMR: (100 MHz, $CDCl_3$) $\delta$ 146.1, 141.2, 136.3, 127.5, 126.6, 123.8, 122.7, 119.5, 117.3, 105.4, 39.0, 36.7, 32.8, 32.3, 27.5, 26.8, 24.8, 19.5 *the boron-bound carbons were not detected due to quadrupolar relaxation*; IR (cm$^{-1}$): 2982, 2921, 1601, 1508, 1411, 1258 cm$^{-1}$; HRMS (EI) calculated for $[C_{23}H_{29}BN_2S]^+$ ($M^+$) requires m/z 376.2139, found m/z 376.2145.

**2-(6-methyl-8-(5-methylfuran-2-yl)octyl)-2,3-dihydro-1*H*-naphtho[1,8-*de*][1,3,2]diazabrinine (14)**

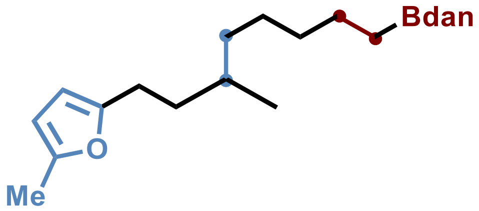


Prepared according to the general procedure: using **L9**•$CoCl_2$ (0.0038 g, 0.0050 mmol), HBdan (0.0630 g, 0.375 mmol), **14a** (0.0514 g, 0.250 mmol), 0.5 mL of THF and $KBHEt_3$ (15 µL, 1M in THF, 0.0150 mmol), stir at r.t. for 12 hours, the residue was purified by preparative TLC (PE/EA/TEA = 100/11/2 (v/v/v), $R_f$ = 0.60) to afford **14** (0.0867 g, 93% yield) as a colorless oil. $^1$H NMR: (400 MHz, $CDCl_3$) $\delta$ 7.09 (dd, $J$ = 8.0, 7.2 Hz, 2H), 6.99 (d, $J$ = 8.4 Hz, 2H), 6.29 (d, $J$ = 7.2 Hz, 2H), 5.83 (s, 2H), 5.60 (brs, 2H), 2.64-2.48 (m, 2H), 2.25 (s, 3H), 1.68-1.62 (m, 1H), 1.43-1.16 (m, 10H), 0.90 (d, $J$ = 6.0 Hz, 3H), 0.85 (t, $J$ = 8.0 Hz, 2H); $^{13}$C NMR: (100 MHz, $CDCl_3$) $\delta$ 155.0, 150.0, 141.2, 136.3, 127.5, 119.5, 117.3, 105.7, 105.4, 104.9, 36.7, 35.2, 32.8, 32.4, 26.8, 25.7, 24.8, 19.5, 13.5 *the boron-bound carbons were not detected due to quadrupolar relaxation*; IR (cm$^{-1}$): 2979, 2921, 1601, 1508, 1410, 1259 cm$^{-1}$; HRMS (EI) calculated for $[C_{24}H_{31}BN_2O]^+$ ($M^+$) requires m/z 374.2524, found m/z 374.2529.

**2-(8-(1*H*-indol-1-yl)-6-methyloctyl)-2,3-dihydro-1*H*-naphtho[1,8-*de*][1,3,2]diazaborinine (15)**

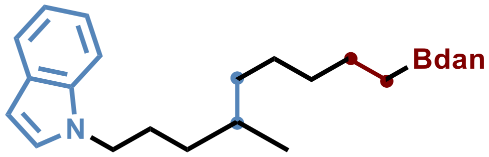

Prepared according to the general procedure: using **L9**•$CoCl_2$ (0.0041 g, 0.0054 mmol), HBdan (0.0635 g, 0.378 mmol), **15a** (0.0634 g, 0.248 mmol), 0.5 mL of THF and $KBHEt_3$ (15 µL, 1M in THF, 0.0150 mmol), stir at r.t. for 12 hours, the residue was purified by preparative TLC (PE/EA/TEA = 100/13/2 (v/v/v), $R_f$ = 0.50) to afford **15** (0.0984 g, 93% yield) as a colorless oil. $^1H$ NMR: (400 MHz, $CDCl_3$) $\delta$ 7.63 (d, $J$ = 8.0 Hz, 1H), 7.33 (d, $J$ = 8.4 Hz, 1H), 7.21-7.18 (m, 1H), 7.11-7.06 (m, 4H), 6.99 (d, $J$ = 8.0 Hz, 2H), 6.48 (d, $J$ = 2.8 Hz, 1H), 6.26 (d, $J$ = 7.2 Hz, 2H), 5.55 (brs, 2H), 4.06 (t, $J$ = 7.2 Hz, 2H), 1.89-1.72 (m, 2H), 1.40-1.03 (m, 11H), 0.85-0.79 (m, 5H); $^{13}C$ NMR: (100 MHz, $CDCl_3$) $\delta$ 141.2, 136.3, 135.9, 128.5, 127.7, 127.5, 121.2, 120.9, 119.5, 119.1, 117.3, 109.3, 105.3, 100.8, 46.7, 36.7, 34.1, 32.7, 32.5, 27.7, 26.8, 24.8, 19.5 *the boron-bound carbons were not detected due to quadrupolar relaxation*; IR ($cm^{-1}$): 2981, 2920, 1601, 1508, 1411, 1258 $cm^{-1}$; HRMS (ESI) calculated for $[C_{28}H_{35}BN_3]^+$ (M + $H^+$) requires m/z 424.2919, found m/z 424.2920.

**2-(9-(benzyloxy)-6-methylnonyl)-2,3-dihydro-1*H*-naphtho[1,8-*de*][1,3,2]diazaborinine (16)**

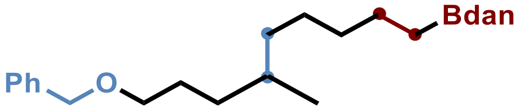


Prepared according to the general procedure: using **L9**•$CoCl_2$ (0.0039 g, 0.0051 mmol), HBdan (0.0630 g, 0.375 mmol), **16a** (0.0617 g, 0.251 mmol), 0.5 mL of THF and $KBHEt_3$ (15 µL, 1M in THF, 0.0150 mmol), stir at r.t. for 12 hours, the residue was purified by preparative TLC (PE/EA/TEA = 100/7/2 (v/v/v), $R_f$ = 0.40) to afford **16** (0.1024 g, 99% yield) as a colorless oil. $^1H$ NMR: (400 MHz, $CDCl_3$) $\delta$ 7.34-7.21 (m, 5H), 7.10-6.97 (m, 4H), 6.26 (d, $J$ = 7.2 Hz, 2H), 5.57 (brs, 2H), 4.49 (s, 2H), 3.44 (dd, $J$ = 6.8, 6.4 Hz, 2H), 1.69-1.13 (m, 13H), 0.87-0.80 (m, 5H); $^{13}C$ NMR: (100 MHz, $CDCl_3$) $\delta$ 141.2, 138.6, 136.3, 128.3, 127.6, 127.5, 127.4, 119.5, 117.2, 105.3, 72.8, 70.8, 36.8, 33.2, 32.7, 32.6, 27.2, 26.8, 24.8, 19.6; IR ($cm^{-1}$): 2982, 2908, 1601, 1510, 1410, 1257 $cm^{-1}$; HRMS (EI) calculated for $[C_{27}H_{35}BN_2O]^+$ ($M^+$) requires m/z 414.2837, found m/z 414.2841.

**2-(9-((tert-butyldiphenylsilyl)oxy)-6-methylnonyl)-2,3-dihydro-1*H*-naphtho[1,8-*de*][1,3,2] diazaborinine (17)**

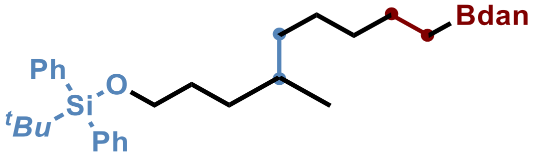

Prepared according to the general procedure: using **L9**•$CoCl_2$ (0.0039 g, 0.0051 mmol), HBdan (0.0630 g, 0.375 mmol), **17a** (0.0986 g, 0.250 mmol), 0.5 mL of THF and $KBHEt_3$ (15 µL, 1M in THF, 0.0150 mmol), stir at r.t. for 12 hours, the residue was purified by preparative TLC (PE/EA/TEA = 100/5/2 (v/v/v), $R_f$ = 0.50) to afford **17** (0.1402 g, >99% yield) as a colorless oil. $^1H$ NMR: (400 MHz, $CDCl_3$) $\delta$ 7.67 (d, $J$ = 6.4 Hz, 4H), 7.42-7.35 (m, 6H), 7.10-6.98 (m, 4H), 6.26 (d, $J$ = 7.6 Hz, 2H), 5.56 (brs, 2H), 3.64 (t, $J$ = 6.4 Hz, 2H), 1.63-1.09 (m, 13H), 1.05 (s, 9H), 0.85-0.81 (m, 5H); $^{13}C$ NMR: (100 MHz, $CDCl_3$) $\delta$ 141.2, 136.3, 135.6, 134.2, 129.5, 127.55, 127.51, 119.5, 117.3, 105.4, 64.3, 36.9, 32.9, 32.8, 32.5, 30.0, 26.9, 24.8, 19.7, 19.2 *the boron-bound carbons were not detected due to quadrupolar relaxation*; IR ($cm^{-1}$): 2974, 2918, 1601, 1507, 1410, 1257 $cm^{-1}$; HRMS (ESI) calculated for $[C_{36}H_{48}BN_2OSi]^+$ (M + $H^+$) requires m/z 563.3623, found m/z 563.3627.

**4-methyl-9-(1*H*-naphtho[1,8-*de*][1,3,2]diazaborinin-2(3*H*)-yl)nonyl 4-methylbenzene sulfonate (18)**

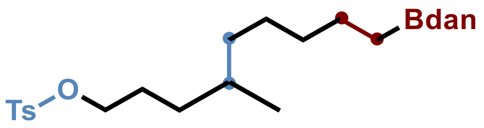


Prepared according to the general procedure: using **L9**•$CoCl_2$ (0.0040 g, 0.0053 mmol), HBdan (0.0625 g, 0.372 mmol), **18a** (0.0779 g, 0.251 mmol), 0.5 mL of THF and $KBHEt_3$ (15 µL, 1M in THF, 0.0150 mmol), stir at r.t. for 12 hours, the residue was purified by preparative TLC (PE/EA/TEA = 100/25/2 (v/v/v), $R_f$ = 0.40) to afford **18** (0.1142 g, 95% yield) as a colorless oil. $^1H$ NMR: (400 MHz, $CDCl_3$) $\delta$ 7.78 (d, $J$ = 8.0 Hz, 2H), 7.32 (d, $J$ = 8.0 Hz, 2H), 7.10-6.98 (m, 4H), 6.29 (d, $J$ = 7.2 Hz, 2H), 5.62 (brs, 2H), 4.00 (dd, $J$ = 6.8, 6.4 Hz, 2H), 2.42 (s, 3H), 1.67-1.08 (m, 13H), 0.86-0.80 (m, 5H); $^{13}C$ NMR: (100 MHz, $CDCl_3$) $\delta$ 144.6, 141.2, 136.2, 133.1, 129.8, 127.8, 127.5, 119.5, 117.2, 105.3, 71.1, 36.6, 32.6, 32.4, 32.2, 26.7, 26.4, 24.7, 21.6, 19.4 *the boron-bound carbons were not detected due to quadrupolar relaxation*; IR ($cm^{-1}$): 2980, 2919, 1601, 1510, 1411, 1257 $cm^{-1}$; HRMS (ESI) calculated for $[C_{27}H_{36}BN_2O_3S]^+$ (M + $H^+$) requires m/z 479.2534, found m/z 479.2533.

**4-methylnonane-1,9-diol (19)**

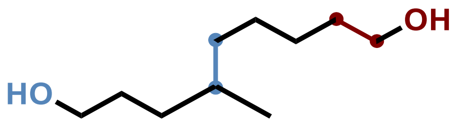


Prepared according to the general procedure after modification: using **L9**•$CoCl_2$ (0.0039 g, 0.0051 mmol), HBdan (0.1240 g, 0.738 mmol), 0.5 mL of THF, $KBHEt_3$ (15 µL, 1M in THF, 0.0150 mmol), and **19a**

(0.0385 g, 0.247 mmol), stir at r.t. for 12 hours, the mixture was filtered through a pad of silica gel and washed with ether (50 mL), then concentrated under vacuum, to a solution of the residue in 5 mL THF, $H_2SO_4$ (0.7 mL, 2 M in $H_2O$) was added dropwise, the mixture was stirred at room temperature for 12 h, then extracted with ether (15 mL x 3). The organic layers were combined, washed with brine, dried over $Na_2SO_4$, concentrated under vacuum to afford alkylboronic acid. To a solution of above alkylboronic acid in 5 mL THF, NaOH (4.0 mL, 3 M in $H_2O$), MeOH (0.5 mL), $H_2O_2$ (3.0 mL, 30% in $H_2O$) was added dropwise, the mixture was stirred at room temperature for 3 h, then extracted with ether (15 mL x 3). The organic layers were combined, washed with brine, dried over $Na_2SO_4$, concentrated under vacuum, the residue was purified by preparative TLC (PE/EA = 50/60(v/v), $R_f$ = 0.40) to afford **19** (0.0246 g, 57% yield) as a colorless oil. $^1H$ NMR: (400 MHz, $CDCl_3$) $\delta$ 3.63 (dd, $J$ = 11.6, 4.8 Hz, 4H), 1.66 (brs, 2H), 1.57-1.11 (m, 13H), 0.87 (d, $J$ = 6.4 Hz, 3H); $^{13}C$ NMR: (100 MHz, $CDCl_3$) $\delta$ 63.3, 62.9, 36.8, 32.8, 32.7, 32.5, 30.2, 26.7, 26.0, 19.6; IR ($cm^{-1}$): 2975, 2904, 1599, 1404, 1254 $cm^{-1}$; HRMS (ESI) calculated for $[C_{10}H_{23}O_2]^+$ (M + $H^+$) requires m/z 175.1693, found m/z 175.1693.

**Ethyl 4-methyl-9-(1*H*-naphtho[1,8-*de*][1,3,2]diazaborinin-2(3*H*)-yl)nonanoate (20)**

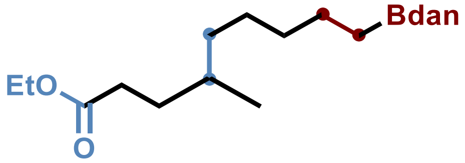


Prepared according to the general procedure after modification: using **L9**•$CoCl_2$ (0.0040 g, 0.0053 mmol), HBdan (0.0638 g, 0.380 mmol), 0.5 mL of THF, $KBHEt_3$ (15 µL, 1M in THF, 0.0150 mmol) and **20a** (0.0490 g, 0.247 mmol), stir at r.t. for 12 hours, the residue was purified by preparative TLC (PE/EA/TEA = 100/20/2 (v/v/v), $R_f$ = 0.50) to afford **20** (0.0851 g, 94% yield) as a colorless oil. $^1H$ NMR: (400 MHz, $CDCl_3$) $\delta$ 7.10-6.98 (m, 4H), 6.28 (d, $J$ = 7.2 Hz, 2H), 5.61 (brs, 2H), 4.12 (q, $J$ = 6.8 Hz, 2H), 2.35-2.25 (m, 2H), 1.70-1.62 (m, 1H), 1.48-1.14 (m, 13H), 0.88-0.82 (m, 5H); $^{13}C$ NMR: (100 MHz, $CDCl_3$) $\delta$ 174.2, 141.2, 136.3, 127.5, 119.5, 117.2, 105.3, 60.2, 36.5, 32.6, 32.3, 32.1, 31.8, 26.7, 24.7, 19.3, 14.2 *the boron-bound carbons were not detected due to quadrupolar relaxation*; IR ($cm^{-1}$): 2984, 2904, 1600, 1455, 1408, 1258 $cm^{-1}$; HRMS (EI) calculated for $[C_{22}H_{31}BN_2O_2]^+$ ($M^+$) requires m/z 366.2473, found m/z 366.2479.

**2-(9-chloro-6-methylnonyl)-2,3-dihydro-1*H*-naphtho[1,8-*de*][1,3,2]diazaborinine (21)**

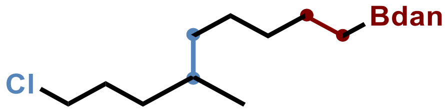


Prepared according to the general procedure after modification: using **L9**•$CoCl_2$ (0.0040 g, 0.0053 mmol), HBdan (0.0632 g, 0.376 mmol), 0.5 mL of THF, $KBHEt_3$ (15 μL, 1M in THF, 0.0150 mmol) and **21a** (0.0438 g, 0.252 mmol), stir at r.t. for 12 hours, the residue was purified by preparative TLC (PE/EA/TEA = 100/7/2 (v/v/v), $R_f$ = 0.50) to afford **21** (0.0808 g, 94% yield) as a colorless oil. $^1$H NMR: (400 MHz, $CDCl_3$) $\delta$ 7.10-6.98 (m, 4H), 6.27 (d, $J$ = 7.2 Hz, 2H), 5.58 (brs, 2H), 3.51 (t, $J$ = 6.8 Hz, 2H), 1.85-1.67 (m, 2H), 1.47-1.07 (m, 11H), 0.88-0.81 (m, 5H); $^{13}$C NMR: (100 MHz, $CDCl_3$) $\delta$ 141.2, 136.3, 127.5, 119.5, 117.3, 105.3, 45.5, 36.7, 34.1, 32.7, 32.2, 30.3, 26.8, 24.8, 19.6 *the boron-bound carbons were not detected due to quadrupolar relaxation*; IR ($cm^{-1}$): 2979, 2924, 1601, 1454, 1410, 1258 $cm^{-1}$; HRMS (EI) calculated for $[C_{20}H_{28}BClN_2]^+$ ($M^+$) requires m/z 342.2029, found m/z 342.2036.

**2-(6-methyl-9-(4,4,5,5-tetramethyl-1,3,2-dioxaborolan-2-yl)nonyl)-2,3-dihydro-1*H*-naphtho [1,8-*de*][1,3,2]diazaborinine (22)**

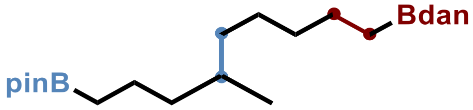


Prepared according to the general procedure: using **L9**•$CoCl_2$ (0.0037 g, 0.0049 mmol), HBdan (0.0628 g, 0.374 mmol), **22a** (0.0665 g, 0.251 mmol), 0.5 mL of THF and $KBHEt_3$ (15 μL, 1M in THF, 0.0150 mmol), stir at r.t. for 12 hours, the residue was purified by preparative TLC (PE/EA/TEA = 100/12/2 (v/v/v), $R_f$ = 0.50) to afford **22** (0.0764 g, 71% yield) as a colorless oil. $^1$H NMR: (400 MHz, $CDCl_3$) $\delta$ 7.10-6.98 (m, 4H), 6.29 (d, $J$ = 7.2 Hz, 2H), 5.61 (brs, 2H), 1.43-1.09 (m, 25H), 0.85-0.82 (m, 5H), 0.76 (dd, $J$ = 8.0, 7.6 Hz, 2H); $^{13}$C NMR: (100 MHz, $CDCl_3$) $\delta$ 141.2, 136.3, 127.5, 119.5, 117.2, 105.3, 82.8, 39.9, 36.9, 32.8, 32.5, 26.8, 24.8, 21.4, 19.7 *the boron-bound carbons were not detected due to quadrupolar relaxation*; IR ($cm^{-1}$): 2970, 2906, 1601, 1454, 1409, 1258 $cm^{-1}$; HRMS (EI) calculated for $[C_{26}H_{40}B_2N_2O_2]^+$ ($M^+$) requires m/z 434.3270, found m/z 434.3281.

**2-(7-(oxetan-3-yl)heptyl)-2,3-dihydro-1*H*-naphtho[1,8-*de*][1,3,2]diazaborinine (23)**

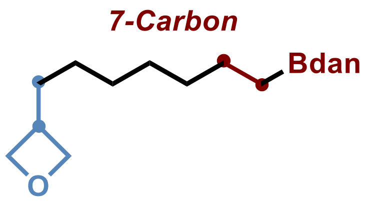


Prepared according to the general procedure: using **L9**•$CoCl_2$ (0.0048 g, 0.0063 mmol), HBdan (0.0648 g, 0.386 mmol), **23a** (0.0390 g, 0.253 mmol), 0.5 mL of THF and $KBHEt_3$ (15 μL, 1M in THF, 0.0150 mmol), stir at r.t. for 12 hours, the residue was purified by preparative TLC (PE/EA/TEA = 100/20/2 (v/v/v), $R_f$ = 0.50) to afford **23** (0.0487 g, 60% yield) as a colorless oil. $^1$H NMR: (400 MHz, $CDCl_3$) $\delta$ 7.11-6.98 (m, 4H), 6.28 (d, $J$ = 7.2 Hz, 2H), 5.61 (brs, 2H), 4.77 (dd, $J$ = 8.0, 6.0 Hz, 2H), 4.36 (t, $J$ = 6.0 Hz, 2H), 2.97-2.93 (m, 1H), 1.69-1.63 (m, 2H), 1.43-1.15 (m, 10H), 0.84 (dd, $J$ = 8.0, 7.6 Hz, 2H); $^{13}$C NMR: (100 MHz, $CDCl_3$) $\delta$ 141.2, 136.3, 127.5, 119.5, 117.3, 105.3, 77.8, 35.2, 33.7, 32.4, 29.40, 29.36, 27.0, 24.8 *the boron-bound carbons were not detected due to quadrupolar relaxation*; IR (cm$^{-1}$): 2922, 2854, 1601, 1513, 1413, 1371 cm$^{-1}$; HRMS (ESI) calculated for $[C_{20}H_{28}BN_2O]^+$ (M + H$^+$) requires m/z 323.2289, found m/z 323.2291.

**2-(5-(tetrahydro-2*H*-pyran-4-yl)pentyl)-2,3-dihydro-1*H*-naphtho[1,8-*de*][1,3,2]diazaborinine (24)**

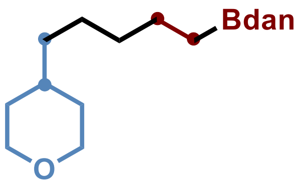


Prepared according to the general procedure: using **L9**•$CoCl_2$ (0.0039 g, 0.0051 mmol), HBdan (0.0682 g, 0.406 mmol), **24a** (0.0392 g, 0.255 mmol), 0.5 mL of THF and $KBHEt_3$ (15 μL, 1M in THF, 0.0150 mmol), stir at r.t. for 12 hours, the residue was purified by preparative TLC (PE/EA/TEA = 100/33/2 (v/v/v), $R_f$ = 0.50) to afford **24** (0.0810 g, 99% yield) as a white solid. $^1$H NMR: (400 MHz, $CDCl_3$) $\delta$ 7.11-6.98 (m, 4H), 6.28 (d, $J$ = 7.2 Hz, 2H), 5.60 (brs, 2H), 4.01-3.88 (m, 2H), 3.36 (dd, $J$ = 12.0, 11.6 Hz, 2H), 1.63-1.24 (m, 13H), 0.85 (dd, $J$ = 8.4, 7.2 Hz, 2H); $^{13}$C NMR: (100 MHz, $CDCl_3$) $\delta$ 141.2, 136.3, 127.5, 119.5, 117.3, 105.3, 68.2, 36.8, 34.9, 33.2, 32.6, 26.2, 24.8 *the boron-bound carbons were not detected due to quadrupolar relaxation*; IR (cm$^{-1}$): 2972, 2918, 1601, 1411, 1375, 1258 cm$^{-1}$; HRMS (EI) calculated for $[C_{20}H_{27}BN_2O]^+$ (M$^+$) requires m/z 322.2211, found m/z 322.2217.

**2-(5-(tetrahydro-2H-thiopyran-4-yl)pentyl)-2,3-dihydro-1*H*-naphtho[1,8-*de*][1,3,2]diaza-borinine (25)**

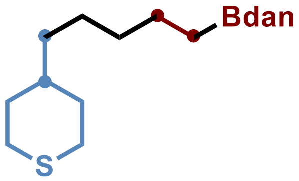


Prepared according to the general procedure: using **L9**•$CoCl_2$ (0.0040 g, 0.0053 mmol), HBdan (0.0642 g, 0.382 mmol), **25a** (0.0426 g, 0.250 mmol), 0.5 mL of THF and $KBHEt_3$ (15 µL, 1M in THF, 0.0150 mmol), stir at r.t. for 12 hours, the crude product was purified by column chromatography on silica gel using PE/EA/TEA =100/5/2 (v/v/v) as the eluent to afford **25** (0.0803 g, 95% yield) as a white solid. $^1H$ NMR: (400 MHz, $CDCl_3$) $\delta$ 7.10-6.98 (m, 4H), 6.27 (d, $J$ = 7.2 Hz, 2H), 5.58 (brs, 2H), 2.68-2.55 (m, 4H), 1.99-1.96 (m, 2H), 1.42-1.21 (m, 11H), 0.83 (t, $J$ = 8.0 Hz, 2H); $^{13}C$ NMR: (100 MHz, $CDCl_3$) $\delta$ 141.1, 136.2, 127.5, 119.5, 117.3, 105.3, 37.2, 37.1, 34.2, 32.6, 28.8, 26.2, 24.7 *the boron-bound carbons were not detected due to quadrupolar relaxation*; IR ($cm^{-1}$): 2984, 2915, 1599, 1508, 1411, 1372 $cm^{-1}$; HRMS (ESI) calculated for $[C_{20}H_{28}BN_2S]^+$ (M + $H^+$) requires m/z 339.2061, found m/z 339.2063.

**2-(5-cyclooctylpentyl)-2,3-dihydro-1*H*-naphtho[1,8-*de*][1,3,2]diazaborinine (26)**

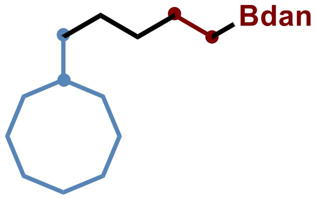


Prepared according to the general procedure: using **L9**•$CoCl_2$ (0.0040 g, 0.0053 mmol), HBdan (0.0630 g, 0.375 mmol), **26a** (0.0451 g, 0.251 mmol), 0.5 mL of THF and $KBHEt_3$ (15 µL, 1M in THF, 0.0150 mmol), stir at r.t. for 12 hours, the residue was purified by preparative TLC (PE/EA/TEA = 100/2/2 (v/v/v), $R_f$ = 0.50) to afford **26** (0.0868 g, >99% yield) as a colorless oil. $^1H$ NMR: (400 MHz, $CDCl_3$) $\delta$ 7.10-6.98 (m, 4H), 6.27 (d, $J$ = 6.8 Hz, 2H), 5.58 (brs, 2H), 1.64-1.42 (m, 15H), 1.31-1.19 (m, 8H), 0.83 (t, $J$ = 7.6 Hz, 2H); $^{13}C$ NMR: (100 MHz, $CDCl_3$) $\delta$ 141.2, 136.3, 127.5, 119.5, 117.3, 105.3, 38.2, 37.2, 32.8, 32.5, 27.3, 26.3, 25.6, 24.8 *the boron-bound carbons were not detected due to quadrupolar relaxation*; IR ($cm^{-1}$): 2963, 2915, 1601, 1455, 1410, 1259 $cm^{-1}$; HRMS (EI) calculated for $[C_{23}H_{33}BN_2]^+$ ($M^+$) requires m/z 348.2731, found m/z 348.2737.

**2-(5-cyclododecylpentyl)-2,3-dihydro-1*H*-naphtho[1,8-*de*][1,3,2]diazaborinine (27)**

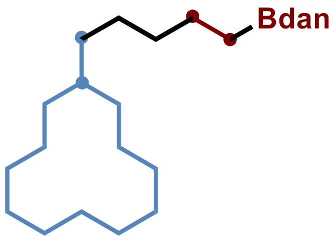


Prepared according to the general procedure: using **L9**•$CoCl_2$ (0.0042 g, 0.0055 mmol), HBdan (0.0596 g, 0.355 mmol), **27a** (0.0593 g, 0.251 mmol), 0.5 mL of THF and $KBHEt_3$ (15 µL, 1M in THF, 0.0150 mmol), stir at r.t. for 12 hours, the residue was purified by preparative TLC (PE/EA/TEA = 100/2/2 (v/v/v), $R_f$ = 0.50) to afford **27** (0.0997 g, 98% yield) as a colorless oil. $^1$H NMR: (400 MHz, $CDCl_3$) $\delta$ 7.10-6.98 (m, 4H), 6.28 (d, $J$ = 7.2 Hz, 2H), 5.58 (brs, 2H), 1.53-1.20 (m, 31H), 0.84 (dd, $J$ = 8.0, 7.6 Hz, 2H); $^{13}$C NMR: (100 MHz, $CDCl_3$) $\delta$ 141.2, 136.3, 127.5, 119.5, 117.3, 105.3, 35.0, 33.9, 32.9, 29.1, 27.3, 24.8, 24.1, 23.33, 23.27, 21.7 *the boron-bound carbons were not detected due to quadrupolar relaxation*; IR ($cm^{-1}$): 2978, 2910, 1601, 1454, 1409, 1257 $cm^{-1}$; HRMS (EI) calculated for $[C_{27}H_{41}BN_2]^+$ ($M^+$) requires m/z 404.3357, found m/z 404.3359.

**(3as,6as)-2,5-bis(5-(1*H*-naphtho[1,8-*de*][1,3,2]diazaborinin-2(3*H*)-yl)pentyl)octahydro pentalene (28)**

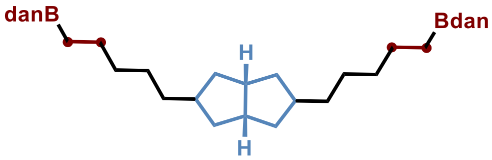


Prepared according to the general procedure after modification: using **L9**•$CoCl_2$ (0.0039 g, 0.0051 mmol), HBdan (0.1282 g, 0.763 mmol), **28a** (0.00618 g, 0.251 mmol), 0.5 mL of THF and $KBHEt_3$ (15 µL, 1M in THF, 0.0150 mmol), stir at r.t. for 12 hours, the crude product was purified by column chromatography on silica gel using PE/TEA =100/2 (v/v) as the eluent to afford **28** (0.1035 g, 71% yield) as a white solid. $^1$H NMR: (400 MHz, $CDCl_3$) $\delta$ 7.10-6.98 (m, 8H), 6.27 (d, $J$ = 7.2 Hz, 4H), 5.57 (brs, 4H), 2.49-2.27 (m, 2H), 1.97-1.56 (m, 4H), 1.47-1.07 (m, 20H), 0.85-0.61 (m, 6H); $^{13}$C NMR: (100 MHz, $CDCl_3$) $\delta$ 141.2, 136.3, 127.5, 119.5, 117.3, 105.3, 45.0, 44.1, 42.4, 42.0, 41.8, 41.7, 41.3, 41.1, 39.9, 39.8, 37.3, 35.6, 35.1, 34.9, 34.8, 32.8, 28.8, 28.7, 28.6, 24.78, 24.75 *the boron-bound carbons were not detected due to quadrupolar relaxation*; IR ($cm^{-1}$): 2977, 2917, 1601, 1508, 1411, 1259 $cm^{-1}$; HRMS (ESI) calculated for $[C_{38}H_{49}B_2N_4]^+$ (M + $H^+$) requires m/z 583.4138, found m/z 583.4142.

**2-(5-(1,4-dioxaspiro[4.5]decan-8-yl)pentyl)-2,3-dihydro-1*H*-naphtho[1,8-*de*][1,3,2] diazaborinine (29)**

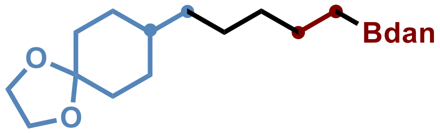


Prepared according to the general procedure: using **L9**•$CoCl_2$ (0.0040 g, 0.0053 mmol), HBdan (0.0630 g, 0.375 mmol), **29a** (0.0525 g, 0.250 mmol), 0.5 mL of THF and $KBHEt_3$ (15 µL, 1M in THF, 0.0150 mmol), stir at r.t. for 12 hours, the residue was purified by preparative TLC (PE/EA/TEA = 100/14/2 (v/v/v), $R_f$ = 0.50) to afford **29** (0.0945 g, >99% yield) as a colorless oil. $^1$H NMR: (400 MHz, $CDCl_3$) $\delta$ 7.10-6.98 (m, 4H), 6.28 (d, $J$ = 7.2 Hz, 2H), 5.60 (brs, 2H), 3.93 (s, 4H), 1.75-1.24 (m, 17H), 0.83 (t, $J$ = 8.0 Hz, 2H); $^{13}$C NMR: (100 MHz, $CDCl_3$) $\delta$ 141.2, 136.3, 127.5, 119.5, 117.2, 109.2, 105.3, 64.15, 64.10, 36.3, 36.1, 34.5, 32.7, 30.2, 27.0, 24.8 *the boron-bound carbons were not detected due to quadrupolar relaxation*; IR (cm$^{-1}$): 2982, 2919, 1601, 1411, 1374, 1260 cm$^{-1}$; HRMS (EI) calculated for $[C_{23}H_{31}BN_2O_2]^+$ ($M^+$) requires m/z 378.2473, found m/z 378.2479.

**2-(5-((1*S*,4*R*)-bicyclo[2.2.1]heptan-2-yl)pentyl)-2,3-dihydro-1*H*-naphtho[1,8-*de*][1,3,2] diazaborinine (30)**

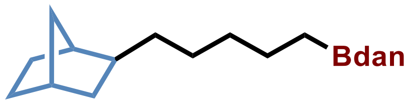


Prepared according to the general procedure: using **L9**•$CoCl_2$ (0.0044 g, 0.0058 mmol), HBdan (0.0650 g, 0.387 mmol), **30a** (0.0412 g, 0.251 mmol), 0.5 mL of THF and $KBHEt_3$ (15 µL, 1M in THF, 0.0150 mmol), stir at r.t. for 12 hours, the residue was purified by preparative TLC (PE/EA/TEA = 100/5/2 (v/v/v), $R_f$ = 0.50) to afford **30** (0.0798 g, 96% yield) as a colorless oil. $^1$H NMR: (400 MHz, $CDCl_3$) $\delta$ 7.10-6.97 (m, 4H), 6.27 (d, $J$ = 7.6 Hz, 2H), 5.57 (brs, 2H), 2.16-1.93 (m, 2H), 1.73 (s, 1.5H), 1.51-1.28 (m, 12H), 1.15-0.99 (m, 2.8H), 0.83 (dd, $J$ = 8.0, 7.2 Hz, 2H), 0.61-0.54 (m, 0.7H); $^{13}$C NMR: (100 MHz, $CDCl_3$) $\delta$ 141.2, 136.3, 127.5, 119.5, 117.3, 105.4, 42.3, 41.1, 40.0, 39.9, 39.8, 38.3, 37.2, 37.1, 36.9, 36.5, 35.2, 32.84, 32.77, 32.7, 30.2, 28.8, 28.7, 27.8, 24.8, 22.3 *the boron-bound carbons were not detected due to quadrupolar relaxation*; IR (cm$^{-1}$): 2944, 2862, 1601, 1508, 1412, 1370 cm$^{-1}$; HRMS (ESI) calculated for $[C_{22}H_{30}BN_2]^+$ ($M + H^+$) requires m/z 333.2497, found m/z 333.2495.

**2-(6-butyldecyl)-2,3-dihydro-1*H*-naphtho[1,8-*de*][1,3,2]diazaborinine (31)**

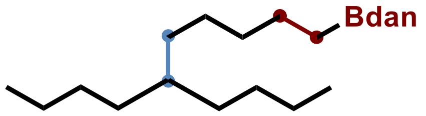


Prepared according to the general procedure: using **L9**•$CoCl_2$ (0.0039 g, 0.0051 mmol), HBdan (0.0630 g, 0.375 mmol), **31a** (0.0494 g, 0.252 mmol), 0.5 mL of THF and $KBHEt_3$ (15 µL, 1M in THF, 0.0150 mmol), stir at r.t. for 12 hours, the residue was purified by preparative TLC (PE/EA/TEA = 100/2/2 (v/v/v), $R_f$ = 0.50) to afford **31** (0.0907 g, >99% yield) as a colorless oil. $^1$H NMR: (400 MHz, $CDCl_3$) $\delta$ 7.10-6.98 (m, 4H), 6.27 (d, $J$ = 7.6 Hz, 2H), 5.57 (brs, 2H), 1.42-1.23 (m, 21H), 0.91-0.87 (m, 6H), 0.83 (dd, $J$ = 8.0, 7.6 Hz, 2H); $^{13}$C NMR: (100 MHz, $CDCl_3$) $\delta$ 141.2, 136.3, 127.5, 119.5, 117.3, 105.3, 37.3, 33.6, 33.3, 33.0, 28.9, 26.6, 24.8, 23.2, 14.2 *the boron-bound carbons were not detected due to quadrupolar relaxation*; IR (cm$^{-1}$): 2964, 2920, 1602, 1456, 1411, 1256 cm$^{-1}$; HRMS (EI) calculated for $[C_{24}H_{37}BN_2]^+$ ($M^+$) requires m/z 364.3044, found m/z 364.3052.

**2-(6-ethyldecyl)-2,3-dihydro-1*H*-naphtho[1,8-*de*][1,3,2]diazaborinine (32)**

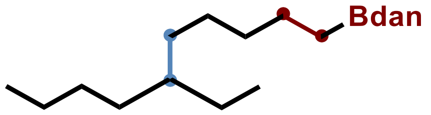


Prepared according to the general procedure: using **L9**•$CoCl_2$ (0.0041 g, 0.0054 mmol), HBdan (0.0630 g, 0.375 mmol), **32a** (0.0423 g, 0.252 mmol), 0.5 mL of THF and $KBHEt_3$ (15 µL, 1M in THF, 0.0150 mmol), stir at r.t. for 12 hours, the residue was purified by preparative TLC (PE/EA/TEA = 100/2/2 (v/v/v), $R_f$ = 0.50) to afford **32** (0.0832 g, 99% yield) as a colorless oil. $^1$H NMR: (400 MHz, $CDCl_3$) $\delta$ 7.10-6.98 (m, 4H), 6.27 (d, $J$ = 7.2 Hz, 2H), 5.57 (brs, 2H), 1.42-1.22 (m, 17H), 0.89 (dd, $J$ = 7.2, 6.4 Hz, 3H), 0.85-0.81 (m, 5H); $^{13}$C NMR: (100 MHz, $CDCl_3$) $\delta$ 141.2, 136.3, 127.5, 119.5, 117.3, 105.3, 38.8, 33.1, 33.0, 32.8, 29.0, 26.6, 25.9, 24.8, 23.2, 14.2, 10.9 *the boron-bound carbons were not detected due to quadrupolar relaxation*; IR (cm$^{-1}$): 2963, 2920, 1602, 1456, 1411, 1336 cm$^{-1}$; HRMS (EI) calculated for $[C_{22}H_{33}BN2]^+$ ($M^+$) requires m/z 336.2731, found m/z 336.2738.

**2-(6-methylheptadecyl)-2,3-dihydro-1*H*-naphtho[1,8-*de*][1,3,2]diazaborinine (33)**

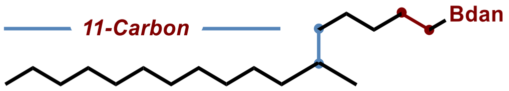


Prepared according to the general procedure: using **L9**•$CoCl_2$ (0.0039 g, 0.0051 mmol), HBdan (0.0628 g, 0.374 mmol), **33a** (0.0631 g, 0.250 mmol), 0.5 mL of THF and $KBHEt_3$ (15 μL, 1M in THF, 0.0150 mmol), stir at r.t. for 12 hours, the residue was purified by preparative TLC (PE/EA/TEA = 100/3/2 (v/v/v), $R_f$ = 0.50) to afford **33** (0.1038 g, 99% yield) as a colorless oil. $^1$H NMR: (400 MHz, $CDCl_3$) $\delta$ 7.10-6.98 (m, 4H), 6.27 (d, $J$ = 7.2 Hz, 2H), 5.57 (brs, 2H), 1.41-1.09 (m, 29H), 0.89-0.81 (m, 8H); $^{13}$C NMR: (100 MHz, $CDCl_3$) $\delta$ 141.2, 136.3, 127.5, 119.5, 117.3, 105.3, 37.1, 37.0, 32.85, 32.76, 31.9, 30.0, 29.7, 29.7, 29.7, 29.4, 27.1, 26.9, 24.8, 22.7, 19.7, 14.1 *the boron-bound carbons were not detected due to quadrupolar relaxation*; IR (cm$^{-1}$): 2965, 2922, 1602, 1456, 1410, 1257 cm$^{-1}$; HRMS (EI) calculated for $[C_{28}H_{45}BN_2]^+$ ($M^+$) requires m/z 420.3670, found m/z 420.3680.

**2-(6-methylnonadecyl)-2,3-dihydro-1*H*-naphtho[1,8-*de*][1,3,2]diazaborinine (34)**

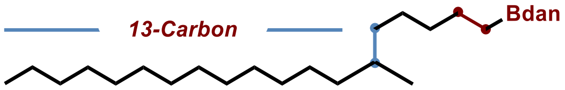


Prepared according to the general procedure: using **L9**•$CoCl_2$ (0.0040 g, 0.0053 mmol), HBdan (0.0630 g, 0.375 mmol), **34a** (0.0705 g, 0.251 mmol), 0.5 mL of THF and $KBHEt_3$ (15 μL, 1M in THF, 0.0150 mmol), stir at r.t. for 12 hours, the residue was purified by preparative TLC (PE/EA/TEA = 100/3/2 (v/v/v), $R_f$ = 0.50) to afford **34** (0.1108 g, 99% yield) as a colorless oil. $^1$H NMR: (400 MHz, $CDCl_3$) $\delta$ 7.10-6.98 (m, 4H), 6.27 (d, $J$ = 7.2 Hz, 2H), 5.57 (brs, 2H), 1.41-1.09 (m, 33H), 0.89-0.81 (m, 8H); $^{13}$C NMR: (100 MHz, $CDCl_3$) $\delta$ 141.2, 136.3, 127.5, 119.5, 117.3, 105.3, 37.1, 37.0, 32.85, 32.76, 31.9, 30.0, 29.71, 29.66, 29.4, 27.1, 26.9, 24.8, 22.7, 19.7, 14.1 *the boron-bound carbons were not detected due to quadrupolar relaxation*; IR (cm$^{-1}$): 2962, 2921, 1602, 1458, 1411, 1256 cm$^{-1}$; HRMS (EI) calculated for $[C_{30}H_{49}BN_2]^+$ ($M^+$) requires m/z 448.3983, found m/z 448.3988.

**2-(6-cyclohexylheptyl)-2,3-dihydro-1H-naphtho[1,8-de][1,3,2]diazaborinine (35)**

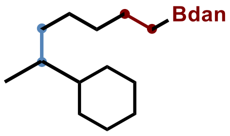

Prepared according to the general procedure: using **L9**•$CoCl_2$ (0.0040 g, 0.0053 mmol), HBdan (0.0631 g, 0.376 mmol), **35a** (0.0450 g, 0.250 mmol), 0.5 mL of THF and $KBHEt_3$ (15 µL, 1M in THF, 0.0150 mmol), stir at r.t. for 12 hours, the residue was purified by preparative TLC (PE/EA/TEA = 100/2/2 (v/v/v), $R_f$ = 0.50) to afford **35** (0.0842 g, 97% yield) as a colorless oil. $^1H$ NMR: (400 MHz, $CDCl_3$) $\delta$ 7.10-6.98 (m, 4H), 6.27 (d, $J$ = 7.2 Hz, 2H), 5.58 (brs, 2H), 1.74-1.52 (m, 5H), 1.42-0.94 (m, 15H), 0.85-0.80 (m, 5H); $^{13}C$ NMR: (100 MHz, $CDCl_3$) $\delta$ 141.2, 136.3, 127.5, 119.5, 117.3, 105.3, 42.7, 38.0, 34.0, 32.9, 30.7, 28.6, 27.4, 27.0, 26.9, 26.8, 24.8, 16.1 *the boron-bound carbons were not detected due to quadrupolar relaxation*; IR ($cm^{-1}$): 2964, 2919, 1602, 1411, 1376, 1259 $cm^{-1}$; HRMS (EI) calculated for $[C_{23}H_{33}BN_2]^+$ ($M^+$) requires m/z 348.2731, found m/z 348.2732.

**2,2'-(6,9-dimethyltetradecane-1,14-diyl)bis(2,3-dihydro-1*H*-naphtho[1,8-*de*][1,3,2]diaza-borinine) (36)**

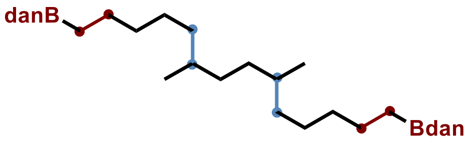


Prepared according to the general procedure after modification: using **L9**•$CoCl_2$ (0.0154 g, 0.020 mmol), HBdan (0.1270 g, 0.756 mmol), **36a** (0.0556 g, 0.250 mmol), 0.5 mL of THF and $KBHEt_3$ (60 µL, 1M in THF, 0.060 mmol),, stir at r.t. for 12 hours, the residue was purified by preparative TLC (PE/EA/TEA = 100/10/2 (v/v/v), $R_f$ = 0.50) to afford **36** (0.1213 g, 87% yield) as a colorless oil. $^1H$ NMR: (400 MHz, $CDCl_3$) $\delta$ 7.10-6.98 (m, 8H), 6.27 (d, $J$ = 7.2 Hz, 4H), 5.57 (brs, 4H), 1.41-1.08 (m, 22H), 0.85-0.81 (m, 10H); $^{13}C$ NMR: (100 MHz, $CDCl_3$) $\delta$ 141.2, 136.3, 127.5, 119.5, 117.3, 105.3, 37.1, 36.8, 34.4, 34.3, 33.1, 33.0, 32.8, 26.95, 26.90, 24.8, 19.8, 19.7 *the boron-bound carbons were not detected due to quadrupolar relaxation*; IR ($cm^{-1}$): 2979, 2920, 1602, 1411, 1377, 1256 $cm^{-1}$; HRMS (ESI) calculated for $[C_{36}H_{49}B_2N_4]^+$ ($M + H^+$) requires m/z 559.4138, found m/z 559.4146.

**2-(6-methyl-8-(2,6,6-trimethylcyclohex-1-en-1-yl)octyl)-2,3-dihydro-1*H*-naphtho[1,8-*de*][1,3,2]diazaborinine (37)**

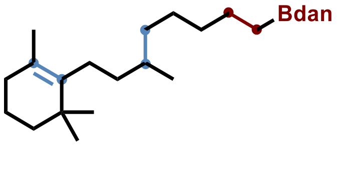

Prepared according to the general procedure: using **L9**•$CoCl_2$ (0.0040 g, 0.0053 mmol), HBdan (0.0628 g, 0.374 mmol), **37a** (0.0623 g, 0.251 mmol), 0.5 mL of THF and $KBHEt_3$ (15 μL, 1M in THF, 0.0150 mmol), stir at r.t. for 12 hours, the residue was purified by preparative TLC (PE/EA/TEA = 100/2/2 (v/v/v), $R_f$ = 0.50) to afford **37** (0.1021 g, 98% yield) as a colorless oil. $^1H$ NMR: (400 MHz, $CDCl_3$) $\delta$ 7.10-6.98 (m, 4H), 6.28 (d, $J$ = 7.2 Hz, 2H), 5.58 (brs, 2H), 2.04-1.88 (m, 4H), 1.58-1.14 (m, 18H), 0.98 (s, 6H), 0.91-0.82 (m, 5H); $^{13}C$ NMR: (100 MHz, $CDCl_3$) $\delta$ 141.2, 137.8, 136.3, 127.5, 126.3, 119.5, 117.3, 105.4, 39.9, 37.5, 36.8, 34.9, 33.9, 32.8, 32.7, 28.7, 28.6, 27.0, 26.3, 24.8, 19.8, 19.6 *the boron-bound carbons were not detected due to quadrupolar relaxation*; IR ($cm^{-1}$): 2967, 2921, 1602, 1410, 1378, 1257 $cm^{-1}$; HRMS (EI) calculated for $[C_{28}H_{41}BN_2]^+$ ($M^+$) requires m/z 416.3357, found m/z 416.3363.

**2-(9-((*S*)-2-(6-methoxynaphthalen-2-yl)propoxy)-6-methylnonyl)-2,3-dihydro-1*H*-naphtho [1,8-*de*][1,3,2]diazaborinine (38)**

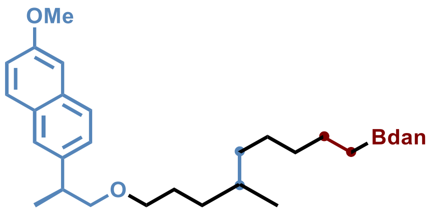


Prepared according to the general procedure: using **L9**•$CoCl_2$ (0.0046 g, 0.0060 mmol), HBdan (0.0681 g, 0.405 mmol), **38a** (0.0897 g, 0.253 mmol), 0.5 mL of THF and $KBHEt_3$ (15 μL, 1M in THF, 0.0150 mmol), stir at r.t. for 12 hours, the residue was purified by preparative TLC (PE/EA/TEA = 100/14/2 (v/v/v), $R_f$ = 0.50) to afford **38** (0.1309 g, 99% yield) as a colorless oil. $^1H$ NMR: (400 MHz, $CDCl_3$) $\delta$ 7.67 (d, $J$ = 8.4 Hz, 2H), 7.58 (s, 1H), 7.34 (d, $J$ = 8.4 Hz, 1H), 7.12-6.98 (m, 6H), 6.27 (d, $J$ = 7.2 Hz, 2H), 5.59 (brs, 2H), 3.88 (s, 3H), 3.60 (dd, $J$ = 8.0, 7.2 Hz, 1H), 3.50 (dd, $J$ = 8.8, 8.4 Hz, 1H), 3.40 (dd, $J$ = 6.8, 6.4 Hz, 2H), 3.14 (q, $J$ = 6.8 Hz, 1H), 1.56-1.08 (m, 16H), 0.84-0.80 (m, 5H); $^{13}C$ NMR: (100 MHz, $CDCl_3$) $\delta$ 157.2, 141.2, 139.7, 136.3, 133.3, 129.1, 129.0, 127.5, 126.7, 126.6, 125.4, 119.5, 118.6, 117.2, 105.5, 105.3, 76.7, 71.5, 55.2, 39.8, 36.8, 33.2, 32.8, 32.5, 27.1, 26.8, 24.8, 19.6, 18.4 *the boron-bound carbons were not detected due to quadrupolar relaxation*; IR ($cm^{-1}$): 2962, 2922, 1602, 1410, 1377, 1261 $cm^{-1}$; HRMS (ESI) calculated for $[C_{34}H_{44}BN_2O_2]^+$ (M + $H^+$) requires m/z 523.3490, found m/z 523.3494.

**2-(9-(2-(4-isobutylphenyl)propoxy)-6-methylnonyl)-2,3-dihydro-1*H*-naphtho[1,8-*de*][1,3,2]diazaborinine (39)**

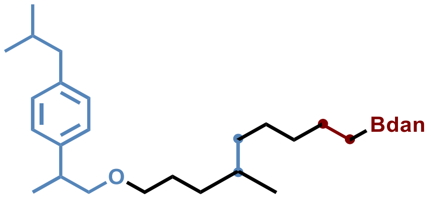


Prepared according to the general procedure: using **L9**•$CoCl_2$ (0.0042 g, 0.0055 mmol), HBdan (0.0639 g, 0.380 mmol), **39a** (0.0831 g, 0.252 mmol), 0.5 mL of THF and $KBHEt_3$ (15 μL, 1M in THF, 0.0150 mmol), stir at r.t. for 12 hours, the residue was purified by preparative TLC (PE/EA/TEA = 100/17/2 (v/v/v), $R_f$ = 0.50) to afford **39** (0.1217 g, 97% yield) as a colorless oil. $^1$H NMR: (400 MHz, $CDCl_3$) $\delta$ 7.13-6.98 (m, 8H), 6.28 (d, $J$ = 7.2 Hz, 2H), 5.59 (brs, 2H), 3.52 (dd, $J$ = 9.2, 6.8 Hz, 1H), 3.42-3.38 (m, 3H), 2.98 (q, $J$ = 6.8 Hz, 1H), 2.43 (d, $J$ = 7.2 Hz, 2H), 1.87-1.80 (m, 1H), 1.56-1.09 (m, 16H), 0.90-0.82 (m, 11H); $^{13}$C NMR: (100 MHz, $CDCl_3$) $\delta$ 141.7, 141.2, 139.5, 136.3, 129.0, 127.5, 127.0, 119.5, 117.3, 105.3, 76.9, 71.4, 45.0, 39.5, 36.8, 33.2, 32.8, 32.6, 30.2, 27.1, 26.8, 24.8, 22.4, 19.6, 18.4 *the boron-bound carbons were not detected due to quadrupolar relaxation*; IR (cm$^{-1}$): 2963, 2919, 1602, 1456, 1410, 1256 cm$^{-1}$; HRMS (ESI) calculated for $[C_{33}H_{48}BN_2O]^+$ (M + H$^+$) requires m/z 499.3854, found m/z 499.3860.

**2-(9-((5-(2,5-dimethylphenoxy)-2,2-dimethylpentyl)oxy)-6-methylnonyl)-2,3-dihydro-1*H*-naphtho[1,8-*de*][1,3,2]diazaborinine (40)**

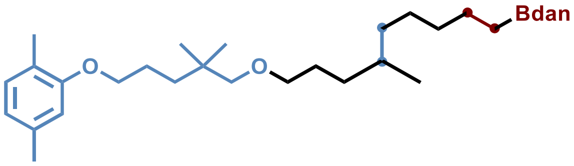


Prepared according to the general procedure: using **L9**•$CoCl_2$ (0.0044 g, 0.0058 mmol), HBdan (0.0645 g, 0.384 mmol), **40a** (0.0941 g, 0.251 mmol), 0.5 mL of THF and $KBHEt_3$ (15 μL, 1M in THF, 0.0150 mmol), stir at r.t. for 12 hours, the residue was purified by preparative TLC (PE/EA/TEA = 100/10/2 (v/v/v), $R_f$ = 0.50) to afford **40** (0.1283 g, 94% yield) as a colorless oil. $^1$H NMR: (400 MHz, $CDCl_3$) $\delta$ 7.10-6.98 (m, 5H), 6.65-6.61 (m, 2H), 6.27 (d, $J$ = 7.6 Hz, 2H), 5.58 (brs, 2H), 3.90 (dd, $J$ = 6.8, 6.4 Hz, 2H), 3.37 (dd, $J$ = 6.8, 6.4 Hz, 2H), 3.09 (s, 2H), 2.30 (s, 3H), 2.18 (s, 3H), 1.78-1.73 (m, 2H), 1.61-1.07

(m, 15H), 0.91 (s, 6H), 0.87-0.81 (m, 5H); $^{13}$C NMR: (100 MHz, $CDCl_3$) $\delta$ 157.1, 141.2, 136.4, 136.3, 130.2, 127.5, 123.5, 120.5, 119.5, 117.3, 112.0, 105.3, 79.7, 71.9, 68.7, 36.9, 35.4, 34.3, 33.3, 32.8, 32.6, 27.1, 26.9, 24.8, 24.6, 24.2, 21.4, 19.7, 15.8 *the boron-bound carbons were not detected due to quadrupolar relaxation*; IR (cm$^{-1}$): 2961, 2917, 1602, 1411, 1378, 1260 cm$^{-1}$; HRMS (ESI) calculated for $[C_{35}H_{52}BN_2O_2]^+$ (M + H$^+$) requires m/z 543.4116, found m/z 543.4119.

**4-methyl-9-(1*H*-naphtho[1,8-*de*][1,3,2]diazaborinin-2(3*H*)-yl)nonyl 3-(4,5-diphenyloxazol-2-yl)propanoate (41)**

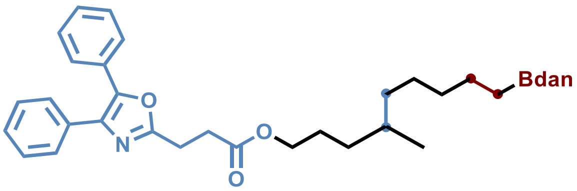


Prepared according to the general procedure after modification: using **L9**•$CoCl_2$ (0.0040 g, 0.0053 mmol), HBdan (0.0630 g, 0.375 mmol), 0.5 mL of THF, $KBHEt_3$ (15 µL, 1M in THF, 0.0150 mmol) and **41a** (0.1146 g, 0.266 mmol), stir at r.t. for 12 hours, the residue was purified by preparative TLC (PE/EA/TEA = 100/25/2 (v/v/v), $R_f$ = 0.50) to afford **41** (0.1456 g, 92% yield) as a colorless oil. $^1$H NMR: (400 MHz, $CDCl_3$) $\delta$ 7.64-7.56 (m, 4H), 7.37-7.28 (m, 6H), 7.09-6.97 (m, 4H), 6.27 (d, $J$ = 6.8 Hz, 2H), 5.62 (brs, 2H), 4.10 (t, $J$ = 6.8 Hz, 2H), 3.18 (dd, $J$ = 8.0, 7.2 Hz, 2H), 2.91 (t, $J$ = 7.6 Hz, 2H), 1.66-1.09 (m, 13H), 0.85-0.80 (m, 5H); $^{13}$C NMR: (100 MHz, $CDCl_3$) $\delta$ 172.0, 161.7, 145.3, 141.2, 136.2, 135.0, 132.4, 128.9, 128.6, 128.5, 128.4, 128.0, 127.8, 127.5, 126.4, 119.5, 117.2, 105.3, 65.2, 36.7, 32.9, 32.7, 32.3, 31.1, 26.8, 26.1, 24.7, 23.5, 19.5 *the boron-bound carbons were not detected due to quadrupolar relaxation*; IR (cm$^{-1}$): 2979, 2904, 1601, 1508, 1409, 1256 cm$^{-1}$; HRMS (ESI) calculated for $[C_{38}H_{43}BN_3O_3]^+$ (M + H$^+$) requires m/z 600.3392, found m/z 600.3396.

**2-(9-(4-chloro-3-(3-((tetrahydrofuran-3-yl)oxy)benzyl)phenoxy)-6-methylnonyl)-2,3-dihydro-1*H*-naphtho[1,8-*de*][1,3,2]diazaborinine (42)**

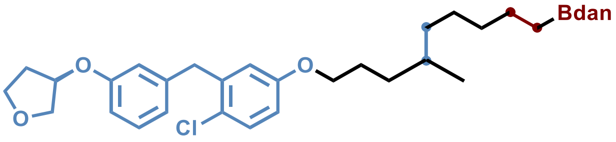

Prepared according to the general procedure: using **L9**•$CoCl_2$ (0.0042 g, 0.0055 mmol), HBdan (0.0612 g, 0.364 mmol), **42a** (0.1072 g, 0.250 mmol), 0.5 mL of THF and $KBHEt_3$ (15 µL, 1M in THF, 0.0150 mmol), stir at r.t. for 12 hours, the residue was purified by preparative TLC (PE/EA/TEA = 100/33/2 (v/v/v), $R_f$ = 0.50) to afford **42** (0.1415 g, 93% yield) as a colorless oil. $^1H$ NMR: (400 MHz, $CDCl_3$) $\delta$ 7.24-7.23 (m, 1H), 7.10-6.97 (m, 6H), 6.78-6.67 (m, 4H), 6.27 (d, $J$ = 7.2 Hz, 2H), 5.60 (brs, 2H), 4.85 (s, 1H), 3.97-3.82 (m, 8H), 2.16-2.06 (m, 2H), 1.74-1.69 (m, 2H), 1.41-1.14 (m, 11H), 0.89-0.81 (m, 5H); $^{13}C$ NMR: (100 MHz, $CDCl_3$) $\delta$ 157.8, 155.8, 141.2, 139.8, 136.2, 131.8, 129.9, 127.5, 125.2, 119.5, 117.25, 117.16, 115.3, 113.3, 105.3, 77.2, 73.1, 68.5, 67.1, 38.5, 36.7, 33.0, 32.9, 32.7, 32.5, 26.8, 26.7, 24.8, 19.6 *the boron-bound carbons were not detected due to quadrupolar relaxation*; IR ($cm^{-1}$): 2981, 2902, 1601, 1511, 1407, 1245 $cm^{-1}$; HRMS (ESI) calculated for $[C_{37}H_{45}BClN_2O_3]^+$ (M + $H^+$) requires m/z 611.3206, found m/z 611.3210.

**2-(9-(((1*S*,2*S*)-2-isopropyl-5-methylcyclohexyl)oxy)-6-methylnonyl)-2,3-dihydro-1*H*-naphtho [1,8-*de*][1,3,2]diazaborinine (43)**

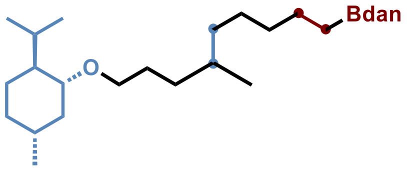


Prepared according to the general procedure: using **L9**•$CoCl_2$ (0.0037 g, 0.0049 mmol), HBdan (0.0650 g, 0.387 mmol), **43a** (0.0740 g, 0.251 mmol), 0.5 mL of THF and $KBHEt_3$ (15 µL, 1M in THF, 0.0150 mmol), stir at r.t. for 12 hours, the residue was purified by preparative TLC (PE/EA/TEA = 100/17/2 (v/v/v), $R_f$ = 0.50) to afford **43** (0.1121 g, 96% yield) as a colorless oil. $^1H$ NMR: (400 MHz, $CDCl_3$) $\delta$ 7.10-6.98 (m, 4H), 6.28 (d, $J$ = 6.8 Hz, 2H), 5.59 (brs, 2H), 3.59 (q, $J$ = 7.2 Hz, 1H), 3.22 (q, $J$ = 7.6 Hz, 1H), 2.98 (dt, $J$ = 10.4, 3.6 Hz, 1H), 2.27-2.19 (m, 1H), 2.09 (d, $J$ = 11.6 Hz, 1H), 1.65-1.01 (m, 18H), 0.92-0.76 (m, 16H); $^{13}C$ NMR: (100 MHz, $CDCl_3$) $\delta$ 141.2, 136.3, 127.5, 119.5, 117.2, 105.3, 79.1, 69.0, 68.9, 48.2, 40.5, 36.9, 36.8, 34.6, 33.4, 32.80, 32.77, 32.6, 31.5, 27.81, 27.79, 26.9, 25.5, 24.8, 23.3, 22.4, 21.0, 19.7, 19.6, 16.2 *the boron-bound carbons were not detected due to quadrupolar relaxation*; IR ($cm^{-1}$): 2961, 2906, 1602, 1454, 1409, 1254 $cm^{-1}$; HRMS (ESI) calculated for $[C_{30}H_{48}BN_2O]^+$ (M + $H^+$) requires m/z 463.3854, found m/z 463.3856.

**2-(6-methyl-9-(((3a*R*,5*R*,5a*S*,8a*S*,8b*R*)-2,2,7,7-tetramethyltetrahydro-5*H*-bis([1,3]dioxolo) [4,5-b:4',5'-d]pyran-5-yl)methoxy)nonyl)-2,3-dihydro-1*H*-naphtho[1,8-*de*][1,3,2] diazaborinine (44)**

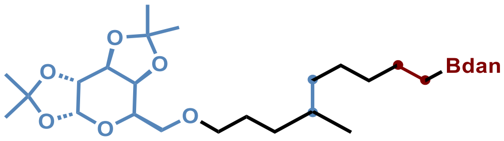


Prepared according to the general procedure: using **L9**•$CoCl_2$ (0.0040 g, 0.0053 mmol), HBdan (0.0631 g, 0.376 mmol), **44a** (0.0996 g, 0.250 mmol), 0.5 mL of THF and $KBHEt_3$ (15 µL, 1M in THF, 0.0150 mmol), stir at r.t. for 12 hours, the residue was purified by preparative TLC (PE/EA/TEA = 100/14/2 (v/v/v), $R_f$ = 0.50) to afford **44** (0.1407 g, 99% yield) as a colorless oil. $^1H$ NMR: (400 MHz, $CDCl_3$) $\delta$ 7.10-6.98 (m, 4H), 6.28 (d, $J$ = 7.2 Hz, 2H), 5.62 (brs, 2H), 5.54 (d, $J$ = 5.2 Hz, 1H), 4.60 (dd, $J$ = 8.0, 2.0 Hz, 1H), 4.32-4.25 (m, 2H), 3.97 (dd, $J$ = 6.4, 6.0 Hz, 1H), 3.66-3.44 (m, 4H), 1.60-1.10 (m, 25H), 0.86-0.82 (m, 5H); $^{13}C$ NMR: (100 MHz, $CDCl_3$) $\delta$ 141.2, 136.2, 127.5, 119.5, 117.2, 109.1, 108.5, 105.3, 96.3, 71.9, 71.1, 70.6, 69.22, 69.20, 66.6, 36.8, 33.0, 32.8, 32.5, 27.0, 26.8, 26.0, 25.9, 24.9, 24.8, 24.4, 19.6 *the boron-bound carbons were not detected due to quadrupolar relaxation*; IR ($cm^{-1}$): 2980, 2920, 1601, 1409, 1379, 1255 $cm^{-1}$; HRMS (ESI) calculated for $[C_{32}H_{48}BN_2O_6]^+$ (M + $H^+$) requires m/z 567.3600, found m/z 567.3606.

**2-(6-methyl-9-(4-(1,2,2-triphenylvinyl)phenoxy)nonyl)-2,3-dihydro-1*H*-naphtho[1,8-*de*][1,3,2]diazaborinine (45)**

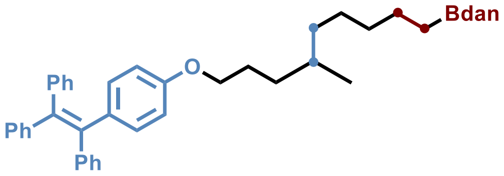


Prepared according to the general procedure: using **L9**•$CoCl_2$ (0.0039 g, 0.0051 mmol), HBdan (0.0630 g, 0.375 mmol), **45a** (0.1240 g, 0.255 mmol), 0.5 mL of THF and $KBHEt_3$ (15 µL, 1M in THF, 0.0150 mmol), stir at r.t. for 12 hours, the residue was purified by preparative TLC (PE/EA/TEA = 100/13/2 (v/v/v), $R_f$ = 0.50) to afford **45** (0.1639 g, 98% yield) as a white solid. $^1H$ NMR: (400 MHz, $CDCl_3$) $\delta$ 7.07-6.97 (m, 19H), 6.91 (d, $J$ = 8.4 Hz, 2H), 6.61 (d, $J$ = 8.4 Hz, 2H), 6.27 (d, $J$ = 7.2 Hz, 2H), 5.58 (brs, 2H), 3.84 (dd, $J$ = 6.4, 6.0 Hz, 2H), 1.74-1.69 (m, 2H), 1.53-1.14 (m, 11H), 0.89-0.82 (m, 5H); $^{13}C$ NMR: (100 MHz, $CDCl_3$) $\delta$ 157.6, 144.04, 143.98, 141.2, 140.5, 139.9, 136.3, 135.8, 132.5, 131.4, 131.33,

131.31, 127.7, 127.5, 126.3, 126.2, 119.5, 117.3, 113.5, 105.4, 68.1, 36.8, 33.1, 32.8, 32.6, 26.8, 24.8, 19.6 *the boron-bound carbons were not detected due to quadrupolar relaxation*; IR ($cm^{-1}$): 2979, 2912, 1601, 1410, 1336, 1247 $cm^{-1}$; HRMS (ESI) calculated for $[C_{46}H_{48}BN_2O]^+$ (M + $H^+$) requires m/z 655.3854, found m/z 655.3855.

# VI. Gram-Scale Reaction and Further Transformations

## Figure S5. Gram-Scale Reaction and Further Transformations

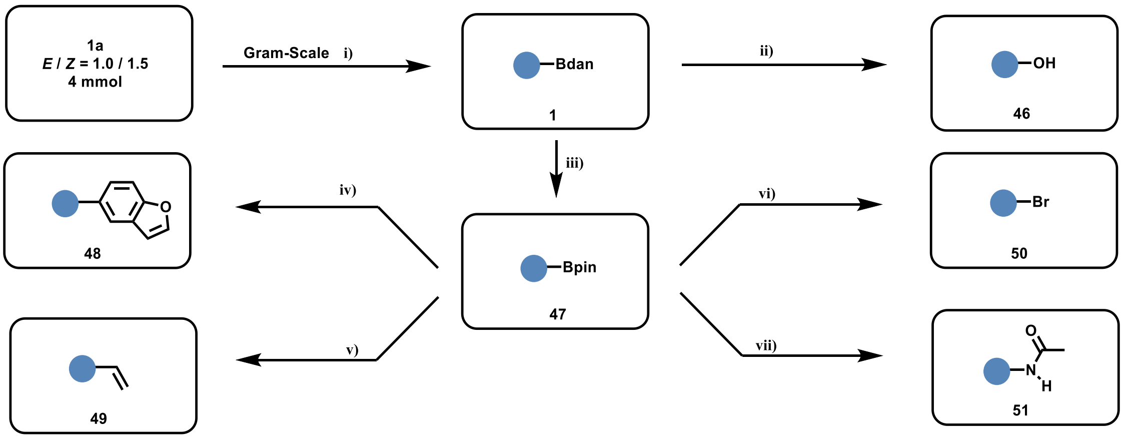


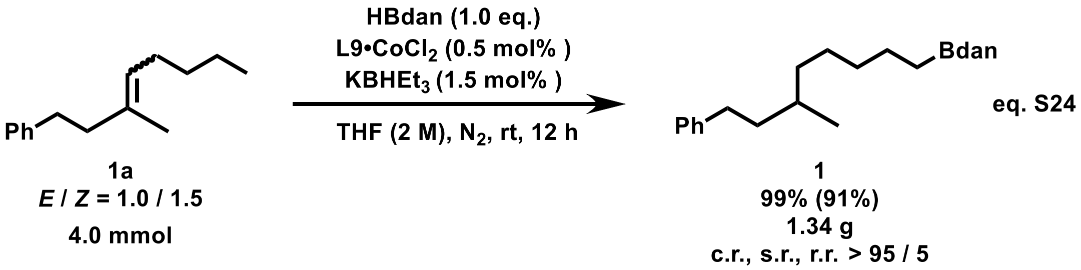


<u>**2-(6-methyl-8-phenyloctyl)-2,3-dihydro-1*H*-naphtho[1,8-*de*][1,3,2]diazaborinine (1)**</u>

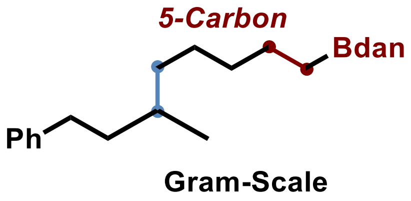


Prepared according to the general procedure: using **L9**•$CoCl_2$ (0.0154 g, 0.0203 mmol), HBdan (0.6760 g, 4.022 mmol), **1a** (0.8100 g, 4.010 mmol), 2.0 mL of THF and $KBHEt_3$ (60 μL, 1M in THF, 0.060 mmol), stir at r.t. for 12 hours, the crude product was purified by column chromatography on silica gel using PE/TEA =100/2 (v/v) as the eluent to afford **1** (1.34 g, 91% yield) as a colorless oil.

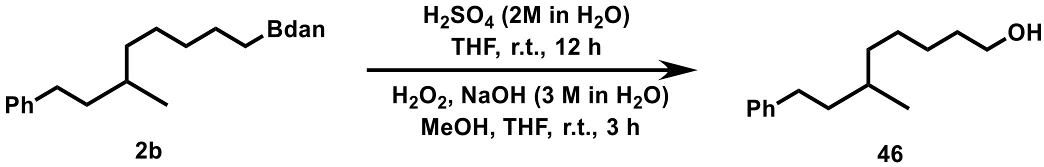


**2b** 0.25 mmol → **46** 99%

eq. S25

<u>**6-methyl-8-phenyloctan-1-ol (46)**</u>

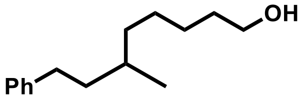


Prepared according to the general procedure: to a solution of **1** (0.0926 g, 0.25 mmol, 1.0 equiv.) in 5 mL THF, $H_2SO_4$ (0.35 mL, 2 M in $H_2O$) was added dropwise, the mixture was stirred at room temperature for 12 h, then extracted with $Et_2O$ (15 mL x 3). The organic layers were combined, washed with brine, dried over $Na_2SO_4$, concentrated under vacuum to afford alkylboronic acid. To a solution of above alkylboronic acid in 5 mL THF, NaOH (4.0 mL, 3 M in $H_2O$), MeOH (0.5 mL), $H_2O_2$ (3.0 mL, 30% in $H_2O$) was added dropwise, the mixture was stirred at room temperature for 3 h, then extracted with $Et_2O$ (15 mL x 3). The organic layers were combined, washed with brine, dried over $Na_2SO_4$, concentrated under vacuum, the residue was purified by preparative TLC (PE/EA = 100/20 (v/v), $R_f$ = 0.50) to afford **46** (0.0551 g, 99% yield) as a colorless oil. $^1H$ NMR: (400 MHz, $CDCl_3$) $\delta$ 7.29-7.14 (m, 5H), 3.62 (dd, $J$ = 6.8, 6.4 Hz, 2H), 2.68-2.52 (m, 2H), 1.65-1.13 (m, 12H), 0.92 (d, $J$ = 6.4 Hz, 3H); $^{13}C$ NMR: (100 MHz, $CDCl_3$) $\delta$ 143.0, 128.3, 128.2, 125.5, 62.9, 38.9, 36.8, 33.4, 32.7, 32.3, 26.7, 26.0, 19.5; IR ($cm^{-1}$): 2970, 2903, 1601, 1455, 1406, 1253 $cm^{-1}$; HRMS (ESI) calculated for $[C_{15}H_{24}NaO]^+$ (M + $Na^+$) requires m/z 243.1719, found m/z 243.1720.

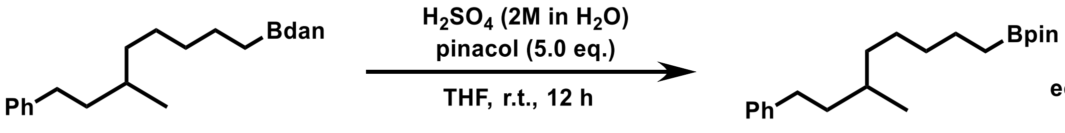


**1** 2.0 mmol → **47** 78%

eq. S26

<u>**4,4,5,5-tetramethyl-2-(6-methyl-8-phenyloctyl)-1,3,2-dioxaborolane (47)**</u>

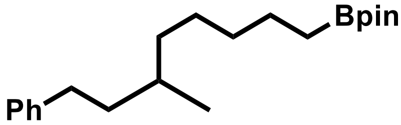

According to the general procedure: to a solution of **1** (0.7306 g, 1.97 mmol, 1.0 equiv.), pinacol (1.15 g, 9.7 mmol, 5.0 eq.) in 5 mL THF, $H_2SO_4$ (3.0 mL, 2 M in $H_2O$) was added dropwise, the mixture was stirred at room temperature for 12 h, then extracted with $Et_2O$ (15 mL x 3). The organic layers were combined, washed with brine, dried over $Na_2SO_4$, concentrated under vacuum, the crude product was purified by column chromatography on silica gel using PE/TEA =100/2 (v/v) as the eluent to afford **47** (0.52 g, 78% yield) as a colorless oil. $^1$H NMR: (400 MHz, $CDCl_3$) $\delta$ 7.29-7.25 (m, 2H), 7.18-7.14 (m, 3H), 2.67-2.53 (m, 2H), 1.65-1.55 (m, 1H), 1.42-1.24 (m, 20H), 0.91 (d, $J$ = 6.4 Hz, 3H), 0.77 (dd, $J$ = 8.0, 7.6 Hz, 2H); $^{13}$C NMR: (100 MHz, $CDCl_3$) $\delta$ 143.2, 128.3, 128.2, 125.5, 82.8, 39.0, 36.8, 33.5, 32.7, 32.5, 26.7, 24.8, 24.0, 19.6 *the boron-bound carbons were not detected due to quadrupolar relaxation*; IR (cm$^{-1}$): 2977, 2924, 1378, 1320, 1252, 1146 cm$^{-1}$; HRMS (ESI) calculated for $[C_{21}H_{35}BNaO_2]^+$ (M + $Na^+$) requires m/z 353.2622, found m/z 353.2625.

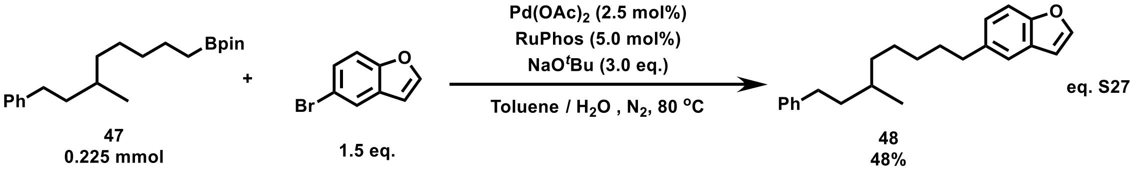


eq. S27

**5-(6-methyl-8-phenyloctyl)benzofuran (48)**

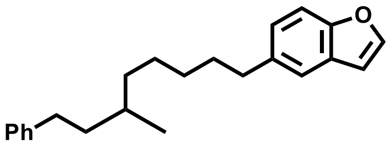


According to the previous reported method (*65*): a 10 mL flame-dried Schlenk flask cooled under nitrogen was charged with **47** (0.7444 g, 0.225 mmol), 5-bromobenzofuran (0.0713 g, 0.375 mmol), $Pd(OAc)_2$ (0.0015 g, 0.006 mmol), RuPhos (0.0056 g, 0.013 mmol), NaO$^t$Bu (0.0705 g, 0.75 mmol), toluene (1 mL) and $H_2O$ (0.1 mL). The mixture was added and stirred at 80 °C for 18 h, then diluted with EA and filtered on silica gel. The solvent was concentrated and the residue was purified by preparative TLC (PE/EA = 100/0 (v/v), $R_f$ = 0.50) to afford **48** (0.0330 g, 48% yield) as a colorless oil. $^1$H NMR: (400 MHz, $CDCl_3$) $\delta$ 7.57 (d, $J$ = 2.0 Hz, 1H), 7.41-7.09 (m, 8H), 6.70 (d, $J$ = 1.2 Hz, 1H), 2.68 (dd, $J$ = 8.0, 7.6 Hz, 2H), 2.64-2.51 (m, 2H), 1.70-1.58 (m, 3H), 1.45-1.16 (m, 8H), 0.91 (d, $J$ = 6.0 Hz, 3H); $^{13}$C NMR: (100 MHz, $CDCl_3$) $\delta$ 153.5, 145.0, 143.2, 137.3, 128.3, 128.2, 127.4, 125.5, 124.9, 120.3, 110.9,

106.4, 38.9, 36.8, 35.9, 33.5, 32.5, 32.1, 29.6, 26.8, 19.6; IR ($cm^{-1}$): 2979, 2931, 1726, 1520, 1465, 1373 $cm^{-1}$; HRMS (EI) calculated for $[C_{23}H_{28}O]^+$ ($M + H^+$) requires m/z 320.2135, found m/z 320.2133.

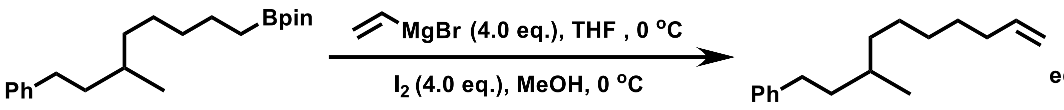


47
0.25 mmol

49
73%

eq. S28

**(3-methyldec-9-en-1-yl)benzene (49)**

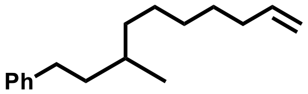


According to the previous reported method (*38*): a 10 mL flame-dried Schlenk flask cooled under nitrogen was charged with **47** (0.0794 g, 0.24 mmol) and THF (2 mL), Vinyl magnesium bromide solution (1.0 mL, 1.0 M in THF, 1.0 mmol) was added dropwise into the stirring mixture at 0 °C. The resulting solution was then stirred at 0 °C for 3 h. Iodine crystals (0.2538 g, 1.0 mmol) dissolved in methanol (2.0 mL) was added dropwise using into the stirring mixture at 0 °C. The reaction was then stirred at 0 °C for 1 h and warmed to room temperature. The reaction was quenched with saturated aqueous $Na_2S_2O_3$ (2 mL), extracted with $Et_2O$ (10 mL × 3). The organic layers were combined, washed with brine, dried over $Na_2SO_4$ and concentrated in vacuo. The crude product was purified by column chromatography on silica gel using PE as the eluent to afford the desired product **49** as colorless oil (0.0404 g, 73%). $^1$H NMR: (400 MHz, $CDCl_3$) $\delta$ 7.28-7.14 (m, 5H), 5.86-5.76 (m, 1H), 5.01-4.92 (m, 2H), 2.68-2.52 (m, 2H), 2.06-2.01 (m, 2H), 1.65-1.58 (m, 1H), 1.45-1.21 (m, 10H), 0.92 (d, $J$ = 6.4 Hz, 3H); $^{13}$C NMR: (100 MHz, $CDCl_3$) $\delta$ 143.2, 139.2, 128.3, 128.2, 125.5, 114.1, 38.9, 36.8, 33.8, 33.5, 32.5, 29.4, 29.0, 26.8, 19.6; IR ($cm^{-1}$): 2976, 2923, 1600, 1454, 1404, 1252 $cm^{-1}$; HRMS (EI) calculated for $[C_{17}H_{26}]^+$ ($M^+$) requires m/z 230.2029, found m/z 230.2028.

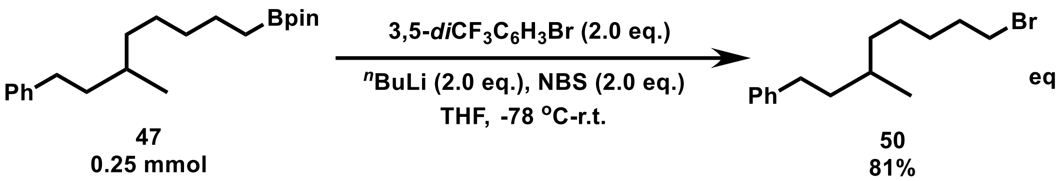


47
0.25 mmol

50
81%

eq. S29

**(8-bromo-3-methyloctyl)benzene (50)**

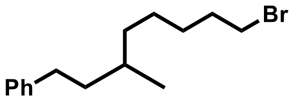

According to the previous reported method (*38*): a 10 mL flame-dried Schlenk flask cooled under nitrogen was charged with 3,5-bis(trifluoromethyl)-1-bromobenzene (0.1506 g, 0.5 mmol) and THF (3 mL), then cooled to -78 °C. A solution of *n*BuLi (200 μL, 2.5 M in hexanes, 0.5 mmol) was added dropwise. After stirred at -78 ºC for 1 h, a solution of **47** (0.0826 g, 0.25 mmol) in THF (1 mL) was then added dropwise. The reaction mixture was allowed to stir at -78 ºC for 1 h and at rt. for 1 h. Then a solution of NBS (0.0908 g, 0.5 mmol) in THF (1 mL) was added dropwise and stir at rt. for 1 h. The reaction was quenched with saturated aqueous $Na_2S_2O_3$ (2 mL), extracted with ethyl acetate (15 mL × 3). The organic layers were combined, washed with brine, dried over $Na_2SO_4$ and concentrated in vacuo. The crude product was purified by column chromatography on silica gel using PE / EA =100 / 1 as the eluent to afford the desired product **50** as a colorless oil (0.0574 g, 81%).$^{1}$H NMR: (400 MHz, $CDCl_3$) $\delta$ 7.29-7.15 (m, 5H), 3.40 (dd, *J* = 7.2, 6.8 Hz, 2H), 2.67-2.53 (m, 2H), 1.89-1.82 (m, 2H), 1.65-1.58 (m, 1H), 1.46-1.17 (m, 8H), 0.92 (d, *J* = 6.0 Hz, 3H); $^{13}$C NMR: (100 MHz, $CDCl_3$) $\delta$ 143.0, 128.32, 128.25, 125.5, 38.9, 36.7, 34.0, 33.4, 32.8, 32.4, 28.4, 26.1, 19.5; IR (cm$^{-1}$): 2977, 2924, 1454, 1378, 1320, 1252 cm$^{-1}$; HRMS (EI) calculated for $[C_{15}H_{23}Br]^+$ ($M^+$) requires m/z 282.0978, found m/z 282.0976.

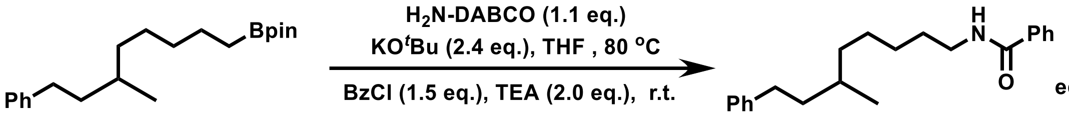


47
0.25 mmol

51
79%

eq. S30

***N*-(6-methyl-8-phenyloctyl)benzamide (51)**

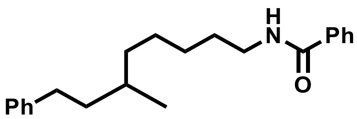

According to the modification of previous reported method (*38*): in the glove box, to a solution of **47** (0.0811 g, 0.245 mmol) and $H_2N$-DABCO (*44*) (0.1068 g, 0.275 mmol) in 4 mL THF was added KO$^t$Bu (0.0667 g, 0.60 mmol) at room temperature. The resulting mixture was then heated to 80 ºC and stirred for 3 h, then BzCl (48 μL, 0.375 mmol) and TEA (70 μL, 0.5 mmol) was added and stirred for 3 h at room temperature. The resulting suspension was then quenched with saturated $NH_4Cl$, extracted with EtOAc, dried with $Na_2SO_4$, filtered and concentrated. The residue was purified by silica gel

chromatography to afford compound **51** as a white solid (0.0626 g, 79%). $^1$H NMR: (400 MHz, $CDCl_3$) $\delta$ 7.75 (d, $J$ = 7.2 Hz, 2H), 7.49-7.39 (m, 3H), 7.28-7.14 (m, 5H), 6.26 (brs, 1H), 3.45-3.40 (m, 2H), 2.68-2.51 (m, 2H), 1.67-1.14 (m, 11H), 0.92 (d, $J$ = 6.4 Hz, 3H); $^{13}$C NMR: (100 MHz, $CDCl_3$) $\delta$ 167.5, 143.0, 134.8, 131.2, 128.5, 128.3, 128.2, 126.8, 125.5, 40.1, 38.8, 36.7, 33.4, 32.3, 29.6, 27.3, 26.6, 19.5; IR ($cm^{-1}$): 2974, 2905, 1640, 1544, 1403, 1253 $cm^{-1}$; HRMS (ESI) calculated for $[C_{22}H_{29}NNaO]^+$ (M + $Na^+$) requires m/z 346.2141, found m/z 346.2144.

## Figure S6. Testing the Long Alkyl Chain (>30)

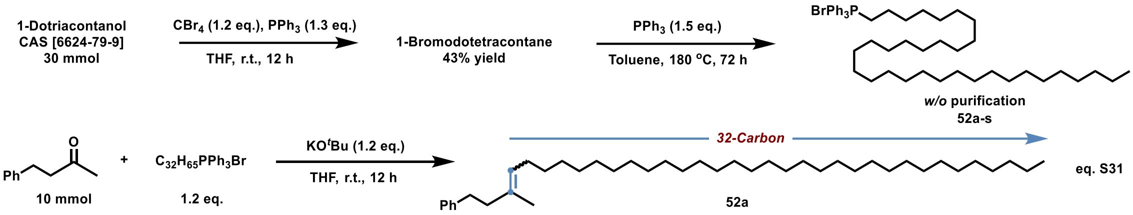


**(3-methylpentatriacont-3-en-1-yl)benzene (52a)**

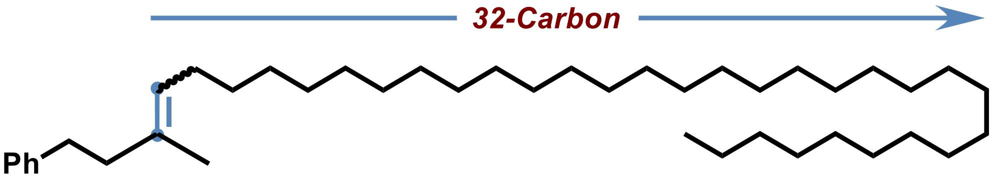


According to **method A**, benzylacetone (10 mmol) and **52a-s** (12 mmol) were used as starting materials, the reaction afforded 1.03 g ($E$ / $Z$ = 1.0 / 1.5, 18% yield) of **52a** as a white solid. *The E/Z ratio of **52a** was inferred from previous rules (50).* $^1$H NMR: (400 MHz, $CDCl_3$) $\delta$ 7.29-7.24 (m, 2H), 7.19-7.16 (m, 3H), 5.16-5.13 (m, 1H), 2.72-2.64 (m, 2H), 2.33-2.25 (m, 2H), 1.97-1.87 (m, 2H), 1.72 (s, 1.8H for $Z$ isomer), 1.64 (s, 1.2H for $E$ isomer), 1.52-1.25 (m, 58H), 0.88 (dd, $J$ = 7.2, 6.4 Hz, 3H); *As **52a** existed as an E/Z mixture, the carbon spectrum was included E and Z isomers*; $^{13}$C NMR: (100 MHz, $CDCl_3$) $\delta$ 142.52, 142.46, 134.2, 134.1, 128.4, 128.24, 128.18, 126.3, 125.7, 125.6, 125.4, 41.6, 34.8, 34.5, 34.0, 31.9, 30.0, 29.8, 29.7, 29.6, 29.41, 29.36, 29.30, 27.9, 27.8, 23.4, 22.7, 16.1, 14.1; HRMS (EI) calculated for $[C_{42}H_{76}]^+$ ($M^+$) requires m/z 580.5942, found m/z 580.5945.

**2-(33-methyl-35-phenylpentatriacontyl)-2,3-dihydro-1*H*-naphtho[1,8-de][1,3,2]diazaborinine (52)**

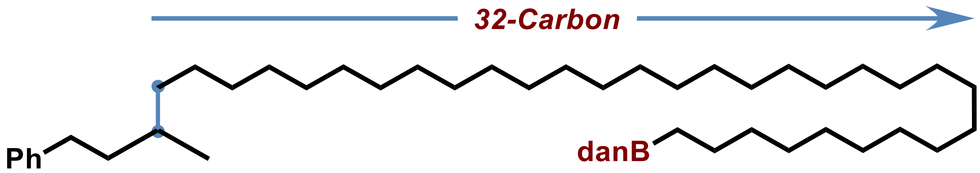


Prepared according to the general procedure: using **L9**•$CoCl_2$ (0.0054 g, 0.0071 mmol), HBdan (0.0650 g, 0.387 mmol), **52a** (0.1430 g, 0.246 mmol), 1.0 mL of THF and $KBHEt_3$ (15 µL, 1M in THF, 0.0150 mmol), stir at r.t. for 12 hours, the crude product was purified by column chromatography on silica gel using PE/EA/TEA =100/2/2 (v/v/v) as the eluent to afford **52** (0.1745 g, 95% yield) as a white solid. $^1$H NMR: (400 MHz, $CDCl_3$) $\delta$ 7.28-7.14 (m, 5H), 7.10-6.97 (m, 4H), 6.27 (d, $J$ = 7.2 Hz, 2H), 5.57 (brs, 2H), 2.68-2.51 (m, 2H), 1.65-1.58 (m, 1H), 1.45-1.36 (m, 4H), 1.30-1.14 (m, 60H), 0.91 (d, $J$ = 6.0 Hz, 3H), 0.83 (dd, $J$ = 8.0, 7.6 Hz, 2H); $^{13}$C NMR: (100 MHz, $CDCl_3$) $\delta$ 143.2, 141.2, 136.3, 128.3, 128.2, 127.5, 125.5, 119.5, 117.3, 105.4, 39.0, 36.9, 33.5, 32.52, 32.49, 30.0, 29.7, 29.6, 29.5, 27.0, 24.8, 19.6 *the boron-bound carbons were not detected due to quadrupolar relaxation*; IR (cm$^{-1}$): 2917, 2850, 1603, 1507, 1465, 1411 cm$^{-1}$; HRMS (ESI) calculated for $[C_{52}H_{86}BN_2]^+$ (M + H$^+$) requires m/z 749.6879, found m/z 749.6879.

## Figure S7. Differentiating between Me & Et Groups

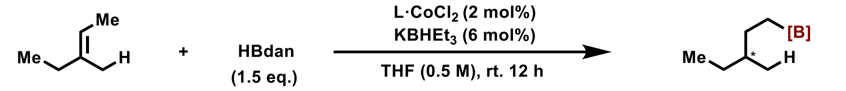


*trans-3-methyl-2-pentene* (53a)
0.25 mmol

53
*s.r. > 95 / 5; r.r. > 95 / 5*

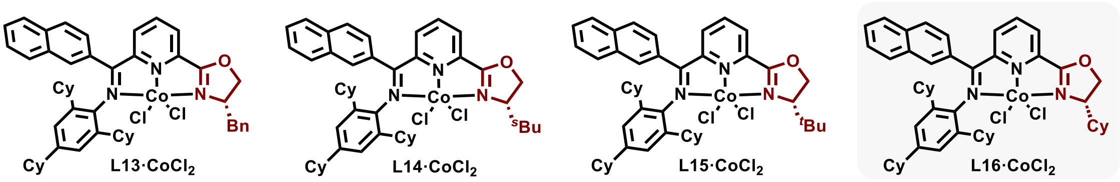


| L13·CoCl2 | L14·CoCl2 | L15·CoCl2 | L16·CoCl2 |
|---|---|---|---|
| 98% yield<br>e.r. = 77 / 23 | >99% yield<br>e.r. = 80 / 20 | >99% yield<br>e.r. = 85 / 15 | 94% yield<br>e.r. = 87 / 13 |

| Entry[a] | Change of conditions | Yield of **53** (%) | e.r. of **53** (%) |
|---|---|---|---|
| 1 | 2-Me-THF as solvent | 58 | 88 / 12 |
| 2 | $Et_2O$ as solvent | 80 | 88 / 12 |
| 3 | Dioxane as solvent | 13 | 89 / 11 |
| 4 | $^i$Pr$_2$O as solvent | 47 | 90 / 10 |
| 5 | MTBE as solvent | <5 | / |
| 6 | CPME as solvent | 80 | 90 / 10 |
| 7 | KO$^t$Bu as additive | 9 | Racemic |

| | | | |
|---|---|---|---|
| 8 | NaO$^t$Bu as additive | / | / |
| 9 | LiO$^t$Bu as additive | 26 | Racemic |
| **10** | **KBH$^s$Bu$_3$ as additive** | **>99(97)** | **90.7 / 9.3** |
| 11 | $^i$Pr$_2$O as solvent, KBH$^s$Bu$_3$ as additive | >99 | 88 / 12 |
| 12 | CPME as solvent, KBH$^s$Bu$_3$ as additive | >99 | 88 / 12 |

**2-(3-methylpentyl)-2,3-dihydro-1*H*-naphtho[1,8-de][1,3,2]diazaborinine (53)**

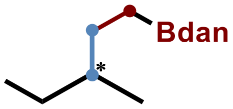


Prepared according to the general procedure: using **L16**•CoCl$_2$ (0.0043 g, 0.0051 mmol), HBdan (0.0630 g, 0.375 mmol), **53a** (0.0214 g, 0.255 mmol), 0.5 mL of THF and KBHEt$_3$ (15 µL, 1M in THF, 0.0150 mmol), stir at r.t. for 12 hours, the residue was purified by preparative TLC (PE/EA/TEA = 100/2/2 (v/v/v), R$_f$ = 0.30) to afford **53** (0.0623 g, 97% yield) as a colorless oil. Optical Rotation: $[\alpha]^{20}$ D = +11.3 (c 1.04, CHCl$_3$), 90/10 e.r. Determined by HPLC, HPLC conditions: Chiral AD-H, n-hexane/i-PrOH = 99.5/0.5, 1.0 mL/min, n = 220 nm, tr 25.1 (major), 26.4 (minor). $^1$H NMR: (400 MHz, CDCl$_3$) $\delta$ 7.10-6.98 (m, 4H), 6.28 (d, $J$ = 7.2 Hz, 2H), 5.58 (brs, 2H), 1.47-1.11 (m, 5H), 0.90-0.87 (m, 6H), 0.85-0.74 (m, 2H); $^{13}$C NMR: (100 MHz, CDCl$_3$) $\delta$ 141.2, 136.3, 127.5, 119.5, 117.3, 105.3, 36.5, 31.4, 29.1, 18.9, 11.4 *the boron-bound carbons were not detected due to quadrupolar relaxation*; IR (cm$^{-1}$): 2965, 2906, 1601, 1508, 1411, 1374 cm$^{-1}$; HRMS (ESI) calculated for $[C_{16}H_{22}BN_2]^+$ (M + H$^+$) requires m/z 253.1871, found m/z 253.1872.

## Figure S8. Precise "Editing" in multiple similar sites

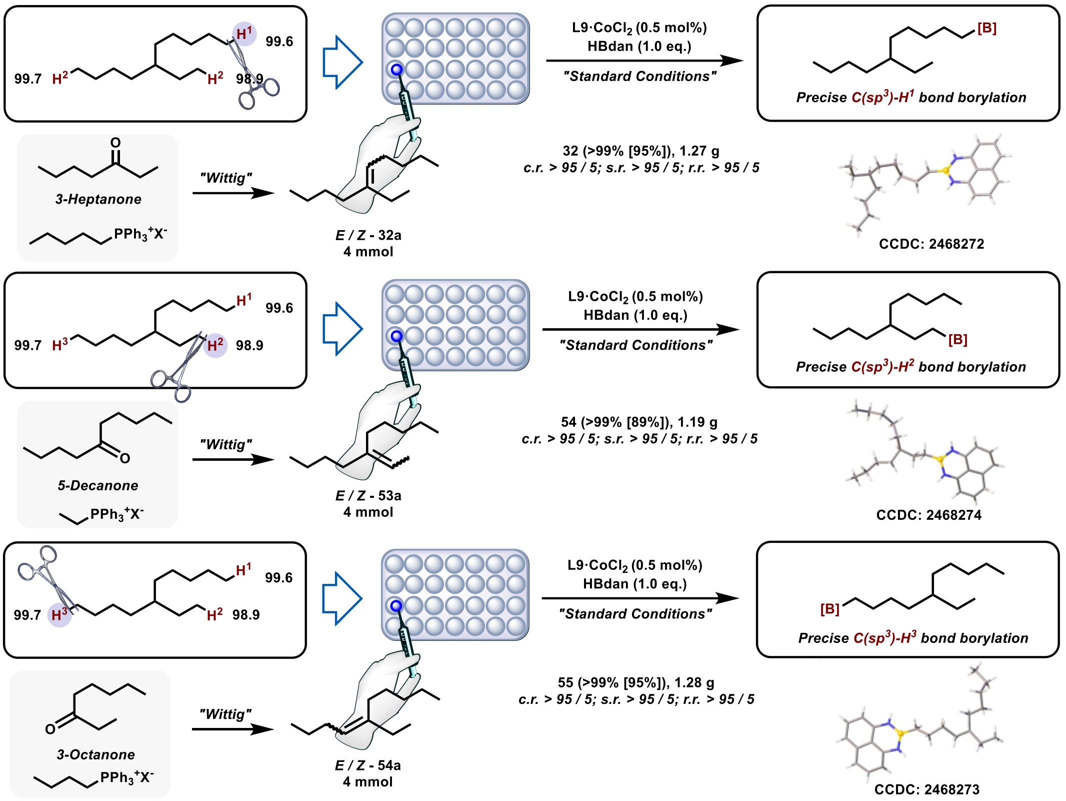


**General procedure for preparing single crystals of guest-included EtP5-MOF-2**

The mother liquor associated with as-synthesized single crystals of **EtP5-MOF-2** was decanted off. The resulting crystals were washed with DMF (3 ×10 mL) three times to remove unreacted reagents. The crystals obtained in this way were then picked up physically, blotted with tissues, and immersed in guest (products **32**, **54**, and **55**) contained in a 2 mL vial, allowing to stand for 10 minutes at room temperature. The single crystals were subsequently subjected to single crystal X-ray diffraction (SCXRD) measurements. See detailed operations in a recently reported Supramolecular Docking technique (*48*).

**Single crystal X-ray diffraction analyses**

SCXRD data were collected on Bruker D8 VENTURE diffractometers with different X-ray sources and detectors (Turbo X-ray Source (TXS) MoKα radiation ($\lambda = 0.71073$ Å) with a PHOTON II CMOS detector, Excillum MetalJet GaKα radiation ($\lambda = 1.34139$ Å) with a PHOTON II CMOS detector, and INCOATEC IμS DIAMOND CuKα radiation ($\lambda = 1.54184$ Å with a PHOTON III CMOS detector) by Shiyanjia Lab

(www.shiyanjia.com). Data were collected at room temperature (300 K) or lower temperatures (100 K, 193 K, 221 K, 238 K). The reduced temperatures were controlled using a KRYOFLEX II low temperature attachment or an Oxford Cryosystems Cryostream 800 cryostat.

Single crystals were mounted on MicroMesh (MiTeGen) using paratone oil. All data collection was performed in a shutterless mode, unit cell was determined by a Bruker APEX software suite (APEX4[S1] or APEX5[S2] during the course of data collection). The data sets were reduced and a multi-scan spherical absorption correction was implemented by Bruker SAINT v8.40B[S3] and SADABS-2016/2 (*66*) or TWINABS-2012/1 for some twinning structures[S4]. The target structures were solved by the intrinsic phasing method using the SHELXT 2018/2[S5] and refined with full-matrix least squares on $F^2$ using the SHELXL 2019/3[S6] (using OLEX2 1.5 (*67*) as the graphical interface). Unless otherwise mentioned, all non-H atoms in the target structure were refined anisotropically and all H atoms were assigned isotropic displacement coefficients U(H) = 1.2 U or 1.5 U, and their coordinates were allowed to ride on their respective atoms.

The refinement procedure of guest molecules could be divided into the following steps: First, the structure of **EtP5-MOF-2** was refined anisotropically and all H atoms were placed into geometrically calculated positions. In this step, constraints like AFIX 66 and/or restraints like DFIX, SADI, DANG, SIMU, ISOR, RIGU could be used to make the whole MOF structure reasonable. After this step, assignments of the electron density peaks were made. Some constrains and/or restrains such as AFIX 66, DFIX, SADI, DANG, SAME, SIMU, ISOR and RIGU could be used if necessary in the refinement of the target molecules. Once the whole molecule was localized and fixed, an anisotropic refinement was carried out. H atoms expected to be present in the target molecule were placed into geometrically calculated positions. At last, residual electron densities in the voids due to highly disordered solvents and/or potential guest molecules were treated with the solvent-mask routine of OLEX2 or SQUEEZE of PLATON. The $F_{obs}$ electron density maps shown in this work are drawn using OLEX2.

S1 Bruker APEX4, Bruker AXS Inc.: Madison (WI), USA, 2022.

S2 Bruker APEX5, Bruker AXS Inc.: Madison (WI), USA, 2023.

S3 SAINT V8.40B, Bruker AXS Inc.: Madison (WI), USA, 2022.

S4 TWINABS, V 2012/1, Bruker AXS Inc., Madison, Wisconsin, USA, 2012.

S5 Sheldrick, G. M. SHELXT-integrated space-group and crystal-structure determination. *Acta Cryst.* **A71**, 3−8 (2015).

S6 Sheldrick, G. M. Crystal structure refinement with SHELXL. *Acta Cryst.* **C71**, 3−8 (2015).

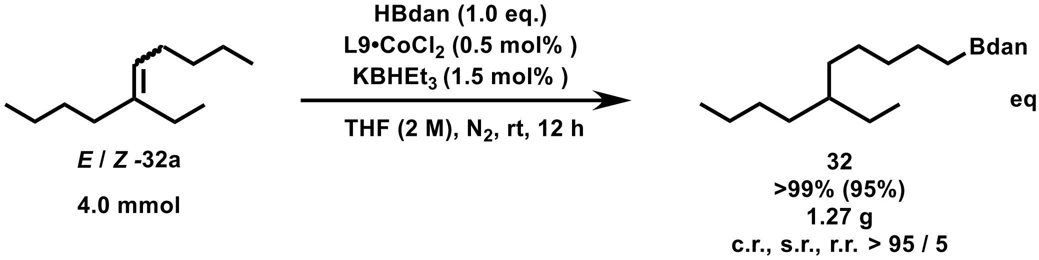


eq. S32

**2-(6-ethyldecyl)-2,3-dihydro-1H-naphtho[1,8-de][1,3,2]diazaborinine (32)**

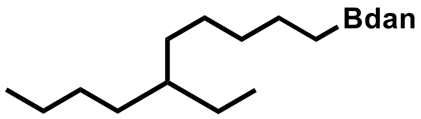


**Gram-Scale**

Prepared according to the general procedure: using **L9**•$CoCl_2$ (0.0154 g, 0.0203 mmol), HBdan (0.7400 g, 4.401 mmol), **32a** (0.6788 g, 4.036 mmol), 2.0 mL of THF and $KBHEt_3$ (60 μL, 1M in THF, 0.0600 mmol), stir at r.t. for 12 hours, the crude product was purified by column chromatography on silica gel using PE/EA/TEA =100/2/2 (v/v/v) as the eluent to afford **32** (1.27 g, 95% yield) as a colorless oil.

Molecular structure of **32** at 50 % probability ellipsoids.

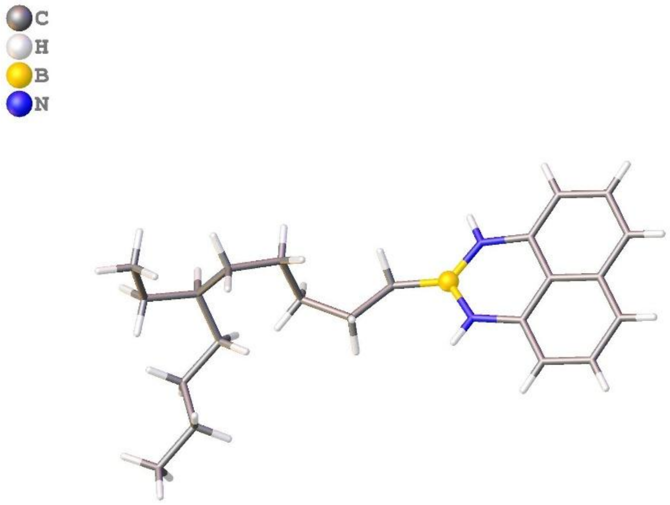


**Figure S9.** Crystal data and structure refinement for **32**

| | |
|---|---|
| CCDC | 2468272 |
| Empirical formula | $C_{132.5}H_{116.25}B_{0.25}N_{2.5}O_{16}Zn_2$ |
| Formula weight | 2132.97 |
| Temperature/K | 193.00 |
| Crystal system | triclinic |
| Space group | P-1 |
| a/Å | 16.8905(13) |
| b/Å | 19.8330(16) |
| c/Å | 26.668(2) |
| α/° | 102.835(5) |
| β/° | 99.539(4) |
| γ/° | 90.173(4) |
| Volume/Å$^3$ | 8582.4(12) |
| Z | 2 |
| $\rho_{calc}$g/cm$^3$ | 0.825 |
| μ/mm$^{-1}$ | 0.696 |
| F(000) | 2236.0 |
| Crystal size/mm$^3$ | 0.13 × 0.12 × 0.09 |
| Radiation | CuKα (λ = 1.54178) |
| 2Θ range for data collection/° | 4.574 to 138.726 |
| Index ranges | -20 ≤ h ≤ 20, -23 ≤ k ≤ 23, -26 ≤ l ≤ 32 |
| Reflections collected | 191292 |
| Independent reflections | 30416 [$R_{int}$ = 0.1199, $R_{sigma}$ = 0.0913] |
| Data/restraints/parameters | 30416/789/1508 |
| Goodness-of-fit on F$^2$ | 1.075 |
| Final R indexes [I>=2σ (I)] | $R_1$ = 0.1456, $wR_2$ = 0.3232 |
| Final R indexes [all data] | $R_1$ = 0.2060, $wR_2$ = 0.3527 |
| Largest diff. peak/hole / e Å$^{-3}$ | 1.19/-1.31 |

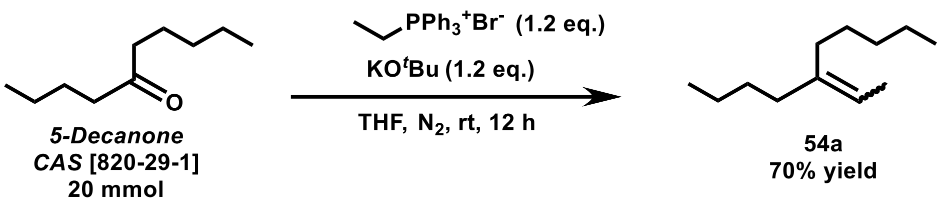


eq. S33

**5-ethylidenedecane (54a)**

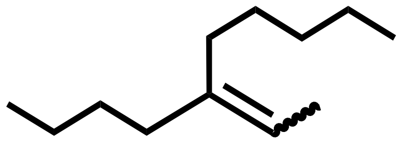

According to **method A**, 5-decanone **CAS [820-29-1]** (21 mmol) and ethyltriphenylphosphonium bromide **CAS [1530-32-1]** (25 mmol) were used as starting materials, the reaction afforded 2.46 g (70% yield) of **54a** as a colorless oil. $^{1}$H NMR: (400 MHz, $CDCl_3$) $\delta$ 5.18 (q, $J$ = 13.2, 6.8 Hz, 1H), 2.02-1.96 (m, 4H), 1.57 (d, $J$ = 6.8 Hz, 3H), 1.39-1.24 (m, 10H), 0.93-0.87 (m, 6H); *As **54a** existed as an E/Z mixture, the carbon spectrum was included E and Z isomers*; $^{13}$C NMR: (100 MHz, $CDCl_3$) $\delta$ 140.7, 118.02, 118.01, 37.0, 36.8, 32.0, 31.8, 30.53, 30.50, 29.7, 29.4, 28.0, 27.9, 22.9, 22.7, 22.6, 14.07, 14.05, 14.02, 13.1; HRMS (EI) calculated for $[C_{12}H_{24}]^+$ ($M^+$) requires m/z 168.1873, found m/z 168.1875.

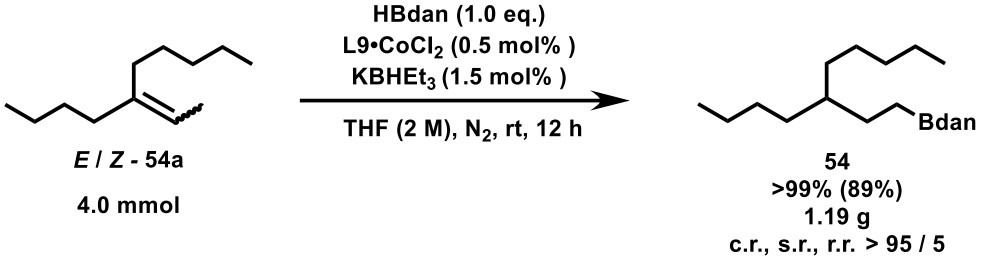


eq. S34

**2-(3-butyloctyl)-2,3-dihydro-1*H*-naphtho[1,8-*de*][1,3,2]diazaborinine (54)**

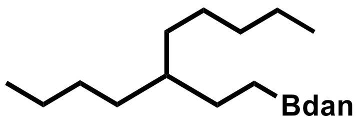


**Gram-Scale**

Prepared according to the general procedure: using **L9**•$CoCl_2$ (0.0163 g, 0.021 mmol), HBdan (0.6750 g, 4 mmol), **54a** (0.6749 g, 4 mmol), 2.0 mL of THF and $KBHEt_3$ (60 μL, 1M in THF, 0.060 mmol), stir at r.t. for 12 hours, the crude product was purified by column chromatography on silica gel using PE/EA/TEA =100/2/2 (v/v/v) as the eluent to afford **54** (1.19 g, 89% yield) as a colorless oil. $^{1}$H NMR: (400 MHz, $CDCl_3$) $\delta$ 7.08-6.96 (m, 4H), 6.23 (d, $J$ = 7.2 Hz, 2H), 5.52 (brs, 2H), 1.41-1.25 (m, 17H),

0.92-0.88 (m, 6H), 0.75 (t, *J* = 8.4 Hz, 2H); $^{13}C$ NMR: (100 MHz, $CDCl_3$) $\delta$ 141.2, 136.3, 127.5, 119.5, 117.2, 105.3, 39.3, 33.2, 32.9, 32.4, 28.9, 28.2, 26.4, 23.2, 22.7, 14.2, 14.1 *the boron-bound carbons were not detected due to quadrupolar relaxation*; IR (cm$^{-1}$): 2962, 2919, 1601, 1506, 1410, 1374 cm$^{-1}$; HRMS (ESI) calculated for $[C_{22}H_{34}BN_2]^+$ (M + $H^+$) requires m/z 337.2810, found m/z 337.2813.

Molecular structure of **54** at 50 % probability ellipsoids.

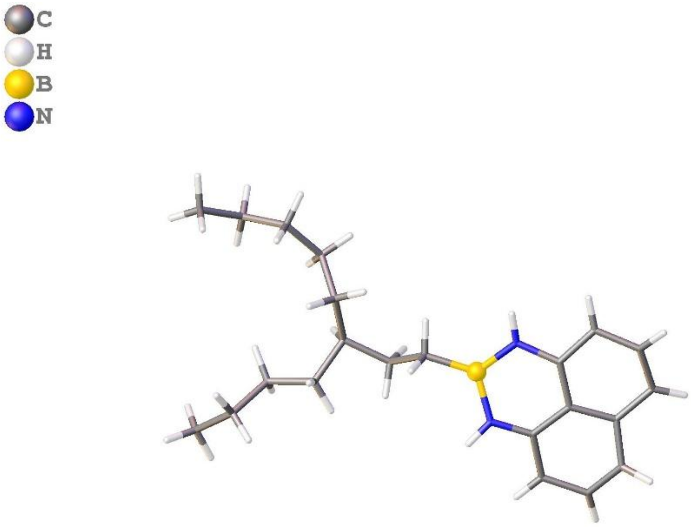


**Figure S10.** Crystal data and structure refinement for **54**

| | |
|---|---|
| CCDC | 2468274 |
| Empirical formula | $C_{138}H_{124.5}B_{0.5}N_3O_{16}Zn_2$ |
| Formula weight | 2217.04 |
| Temperature/K | 193.00 |
| Crystal system | triclinic |
| Space group | P-1 |
| a/Å | 17.0600(16) |
| b/Å | 19.7099(18) |
| c/Å | 26.753(2) |
| α/° | 72.786(3) |
| β/° | 83.851(3) |
| γ/° | 89.923(3) |
| Volume/Å$^3$ | 8538.8(14) |
| Z | 2 |
| $\rho_{calc}$g/cm$^3$ | 0.862 |
| μ/mm$^{-1}$ | 0.479 |
| F(000) | 2328.0 |
| Crystal size/mm$^3$ | 0.13 × 0.12 × 0.09 |
| Radiation | GaKα (λ = 1.34139) |
| 2Θ range for data collection/° | 3.026 to 108.186 |
| Index ranges | -20 ≤ h ≤ 19, -23 ≤ k ≤ 23, -32 ≤ l ≤ 32 |
| Reflections collected | 93863 |
| Independent reflections | 31145 [$R_{int}$ = 0.0875, $R_{sigma}$ = 0.1000] |

| | |
|---|---|
| Data/restraints/parameters | 31145/1532/1876 |
| Goodness-of-fit on $F^2$ | 1.088 |
| Final R indexes [I>=2σ (I)] | $R_1$ = 0.1246, $wR_2$ = 0.3109 |
| Final R indexes [all data] | $R_1$ = 0.1816, $wR_2$ = 0.3419 |
| Largest diff. peak/hole / e Å$^{-3}$ | 0.95/-0.82 |

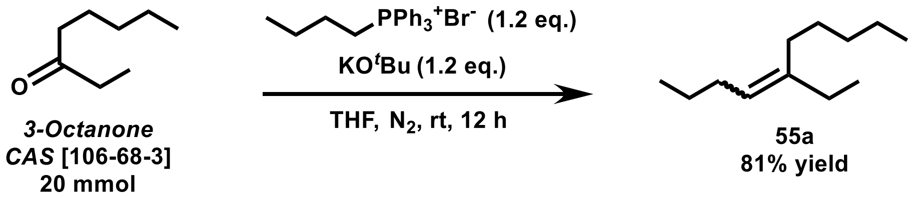


eq. S35

**5-ethyldec-4-ene (55a)**

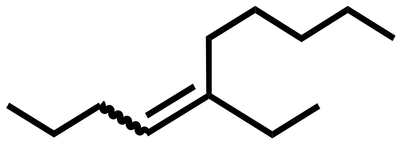

According to **method A**, 3-octanone (20 mmol) and butyltriphenylphosphonium bromide (24 mmol) were used as starting materials, the reaction afforded 2.73 g (81% yield) of **55a** as a colorless oil. $^1$H NMR: (400 MHz, $CDCl_3$) $\delta$ 5.11-5.05 (m, 1H), 2.02-1.96 (m, 6H), 1.39-1.28 (m, 8H), 1.00-0.87 (m, 9H); *As **55a** existed as an E/Z mixture, the carbon spectrum was included E and Z isomers*; $^{13}$C NMR: (100 MHz, $CDCl_3$) $\delta$ 141.3, 123.9, 123.3, 36.5, 32.0, 31.7, 30.1, 29.8, 29.7, 29.6, 28.2, 28.0, 23.3, 23.0, 22.6, 14.10, 14.07, 13.89, 13.86, 13.2, 13.0; HRMS (EI) calculated for $[C_{12}H_{24}]^+$ ($M^+$) requires m/z 168.1873, found m/z 168.1873.

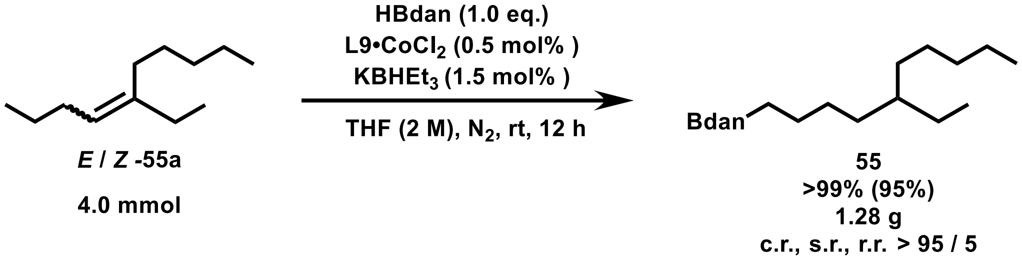


eq. S36

**2-(5-ethyldecyl)-2,3-dihydro-1*H*-naphtho[1,8-*de*][1,3,2]diazaborinine (55)**

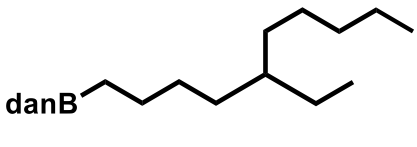


Gram-Scale

Prepared according to the general procedure: using **L9**•$CoCl_2$ (0.0154 g, 0.020 mmol), HBdan (0.6738 g, 4 mmol), **55a** (0.6734 g, 4 mmol), 2.0 mL of THF and $KBHEt_3$ (60 µL, 1M in THF, 0.060 mmol), stir at r.t. for 12 hours, the crude product was purified by column chromatography on silica gel using PE/EA/TEA =100/2/2 (v/v/v) as the eluent to afford **55** (1.28 g, 95% yield) as a colorless oil. $^1$H NMR: (400 MHz, $CDCl_3$) $\delta$ 7.08-6.96 (m, 4H), 6.22 (d, $J$ = 7.2 Hz, 2H), 5.50 (brs, 2H), 1.36-1.12 (m, 17H), 0.88 (dd, $J$ = 7.2, 6.8 Hz, 3H), 0.84 (dd, $J$ = 7.6, 6.8 Hz, 3H), 0.78 (dd, $J$ = 8.0, 7.2 Hz, 2H); $^{13}$C NMR: (100 MHz, $CDCl_3$) $\delta$ 141.2, 136.3, 127.5, 119.5, 117.2, 105.3, 38.7, 33.1, 33.0, 32.4, 29.6, 26.4, 25.8, 25.2, 22.7, 14.1, 10.8 *the boron-bound carbons were not detected due to quadrupolar relaxation*; IR ($cm^{-1}$): 2963, 2921, 1601, 1507, 1411, 1374 $cm^{-1}$; HRMS (ESI) calculated for $[C_{22}H_{34}BN_2]^+$ ($M + H^+$) requires m/z 337.2810, found m/z 337.2809.

Molecular structure of **55** at 50 % probability ellipsoids.

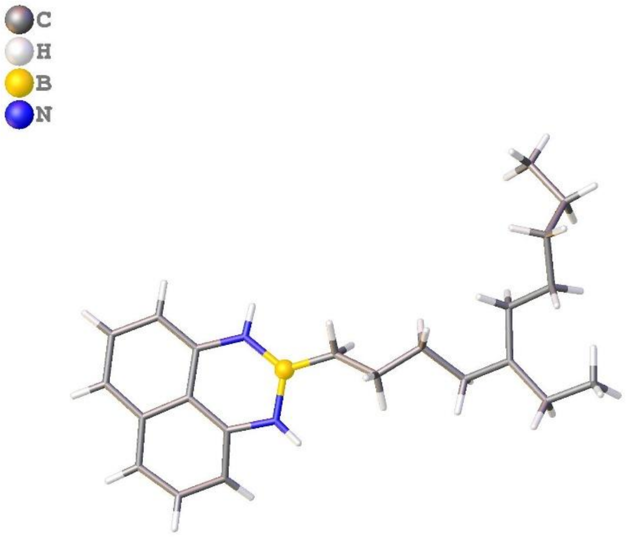


**Figure S11.** Crystal data and structure refinement for **55**

| | |
|---|---|
| CCDC | 2468273 |
| Empirical formula | $C_{134.33}H_{118.67}B_{0.33}N_{2.67}O_{16}Zn_2$ |
| Formula weight | 2160.65 |
| Temperature/K | 193.00 |
| Crystal system | triclinic |
| Space group | P-1 |
| a/Å | 16.971(2) |
| b/Å | 19.826(3) |
| c/Å | 26.781(4) |
| α/° | 73.755(5) |
| β/° | 83.600(6) |
| γ/° | 89.874(5) |
| Volume/Å$^3$ | 8593(2) |

| | |
|---|---|
| Z | 2 |
| $\rho_{calc}$g/cm$^3$ | 0.835 |
| μ/mm$^{-1}$ | 0.470 |
| F(000) | 2266.0 |
| Crystal size/mm$^3$ | 0.13 × 0.11 × 0.09 |
| Radiation | GaKα (λ = 1.34139) |
| 2Θ range for data collection/° | 13.312 to 110.352 |
| Index ranges | -20 ≤ h ≤ 20, -23 ≤ k ≤ 24, -32 ≤ l ≤ 32 |
| Reflections collected | 73757 |
| Independent reflections | 31856 [$R_{int}$ = 0.1217, $R_{sigma}$ = 0.1927] |
| Data/restraints/parameters | 31856/2571/1462 |
| Goodness-of-fit on F$^2$ | 1.248 |
| Final R indexes [I>=2σ (I)] | $R_1$ = 0.1628, w$R_2$ = 0.3683 |
| Final R indexes [all data] | $R_1$ = 0.2576, w$R_2$ = 0.4082 |
| Largest diff. peak/hole / e Å$^{-3}$ | 1.32/-1.07 |

# VII. Mechanistic studies

**Figure S12.** Mechanistic studies

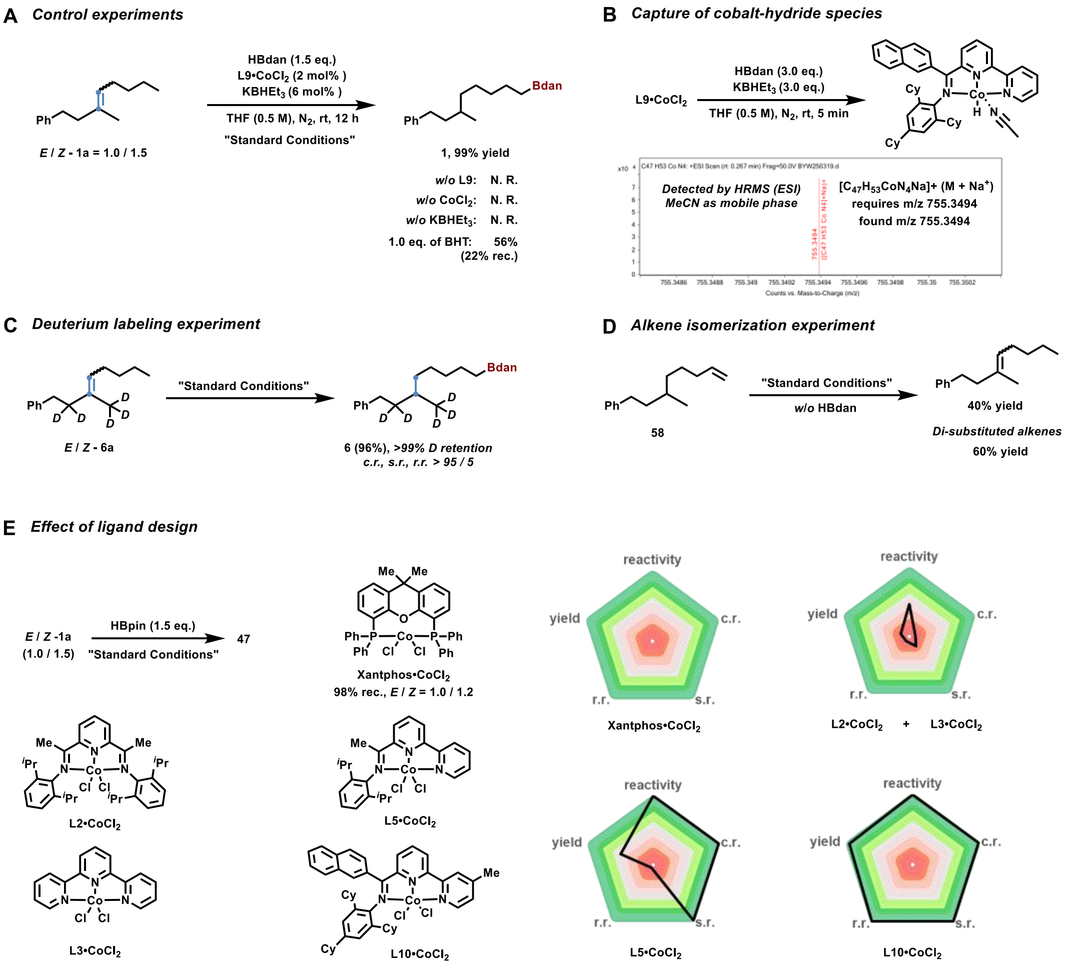


## Control experiments:

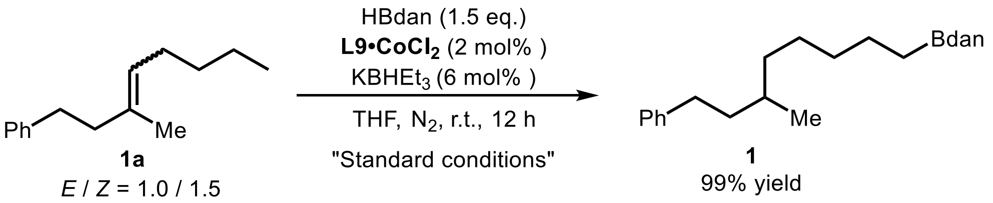


| entry | variation from the conditions | rec. of **1a** | yield of **2a** | eq |
|---|---|---|---|---|
| 1 | *w/o* **L9** | Quant<br>*E* / *Z* = 1.0 / 1.5 | - - - | S37 |
| 2 | *w/o* $CoCl_2$ | Quant<br>*E* / *Z* = 1.0 / 1.5 | - - - | S38 |
| 3 | *w/o* $KBHEt_3$ | Quant<br>*E* / *Z* = 1.0 / 1.5 | - - - | S39 |
| 4 | 1.0 eq. of BHT | 22% | 56% | S40 |

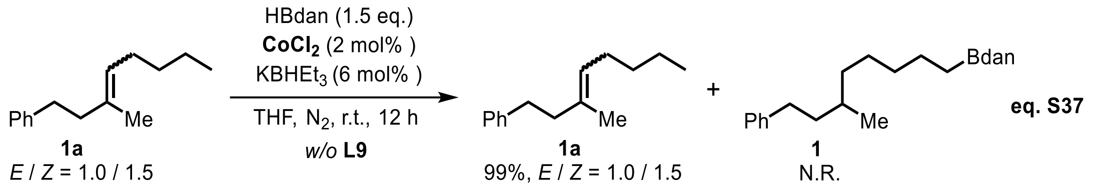


Prepared according to the modification of general procedure: a 10 mL flame-dried flask was cooled at room temperature under nitrogen, charged with $CoCl_2$ (0.0010 g, 0.008 mmol), HBdan (0.0629 g, 0.375 mmol), then **1a** (0.0508 g, 0.251 mmol), dry THF (0.5 mL, 0.5 M), $KBHEt_3$ (15 µL, 1M in THF, 0.015 mmol) were added successively. The mixture was kept under room temperature and stirred for 12 h. The reaction was quenched by PE. The mixture was filtered through a pad of silica gel and washed with ether (50 mL). NMR yield was monitored by $^1$H NMR analysis using phenyltrimethylsilane as internal standard. A mixture of recovery **1a** (99%, *E* / *Z* = 1.0 / 1.5) was observed.

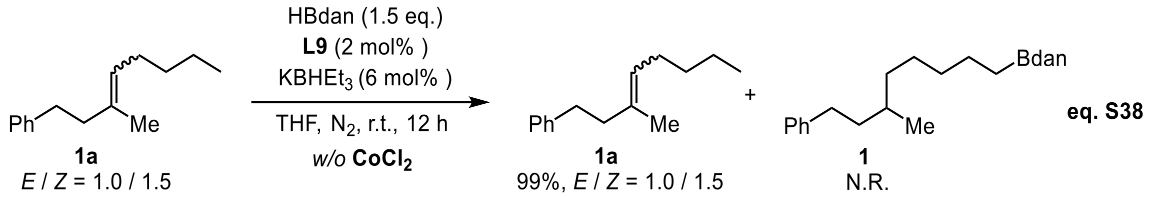


Prepared according to the modification of general procedure: a 10 mL flame-dried flask was cooled at room temperature under nitrogen, charged with **L9** (0.0032 g, 0.005 mmol), HBdan (0.0631 g, 0.375 mmol), then **1a** (0.0508 g, 0.251 mmol), dry THF (0.5 mL, 0.5 M), $KBHEt_3$ (15 µL, 1M in THF, 0.015 mmol) were added successively. The mixture was kept under room temperature and stirred for 12 h. The reaction was quenched by PE. The mixture was filtered through a pad of silica gel and washed with ether (50 mL). NMR yield was monitored by $^1$H NMR analysis using phenyltrimethylsilane as internal standard. A mixture of recovery **1a** (99%, *E* / *Z* = 1.0 / 1.5) was observed.

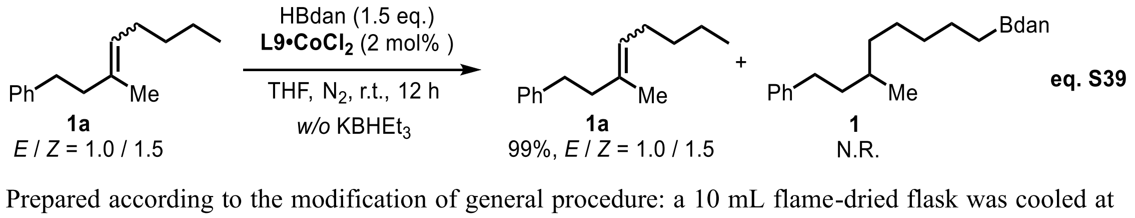


Prepared according to the modification of general procedure: a 10 mL flame-dried flask was cooled at room temperature under nitrogen, charged with **L9**•$CoCl_2$ (0.0040 g, 0.005 mmol), HBdan (0.0629 g, 0.375 mmol), then **1a** (0.0508 g, 0.251 mmol), dry THF (0.5 mL, 0.5 M) were added successively. The mixture was kept under room temperature and stirred for 12 h. The reaction was quenched by PE. The mixture was filtered through a pad of silica gel and washed with ether (50 mL). NMR yield was

monitored by $^{1}$H NMR analysis using phenyltrimethylsilane as internal standard. A mixture of recovery **1a** (99%, $E$ / $Z$ = 1.0 / 1.5) was observed.

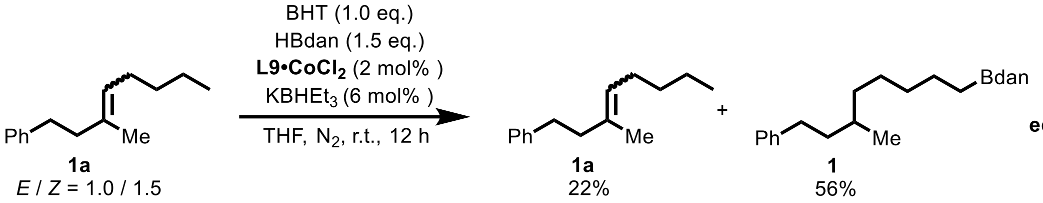


**eq. S40**

Prepared according to the modification of general procedure: a 10 mL flame-dried flask was cooled at room temperature under nitrogen, charged with **L9**•$CoCl_2$ (0.0038 g, 0.005 mmol), HBdan (0.0630 g, 0.375 mmol), BHT (0.0554 g, 0.251 mmol), then **1a** (0.0508 g, 0.251 mmol), dry THF (0.5 mL, 0.5 M), $KBHEt_3$ (15 μL, 1M in THF, 0.015 mmol) were added successively. The mixture was kept under room temperature and stirred for 12 h. The reaction was quenched by PE. The mixture was filtered through a pad of silica gel and washed with ether (50 mL). NMR yield was monitored by $^{1}$H NMR analysis using phenyltrimethylsilane as internal standard. A mixture of recovery **1a** (22%), **1** (56%) was observed.

## Capture of cobalt-hydrogen species:

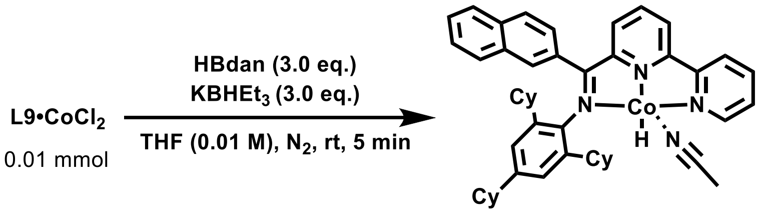


**eq. S41**

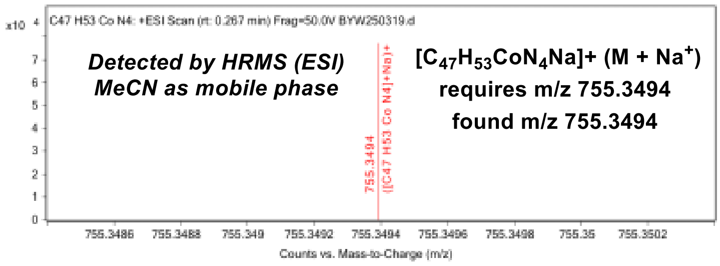


Prepared according to the modification of general procedure: a 10 mL flame-dried flask was cooled at room temperature under nitrogen, charged with **L9**•$CoCl_2$ (0.0076 g, 0.010 mmol), HBdan (0.0050 g, 0.030 mmol), then dry THF (1.0 mL, 0.01 M), $KBHEt_3$ (30 μL, 1M in THF, 0.030 mmol) were added successively. The mixture was kept under room temperature and stirred for 5 min. The mixture was filtered and a Q-TOF-MS analysis experiment using a mixture of the reaction solution was conducted (MeCN as solvent). Indeed, a signal of 755.3494 (M + $Na^+$) is detected, displaying that cobalt hydride species coordinated might be present.

Deuterium labeling experiments:

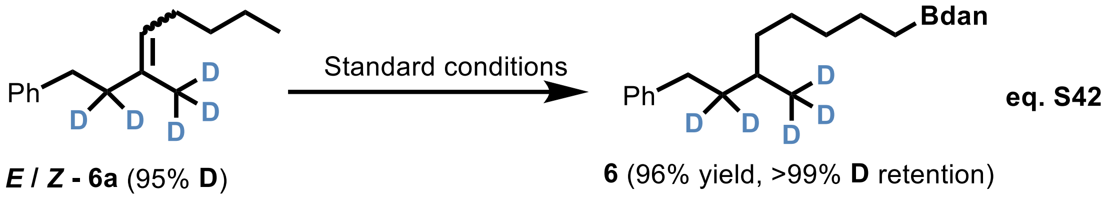


Eq.S42 was prepared according to the modification of general procedure: a 10 mL flame-dried flask was cooled at room temperature under nitrogen, charged with using **L9**•$CoCl_2$ (0.0038 g, 0.0050 mmol), HBdan (0.0640 g, 0.381 mmol), **6a** (0.0520 g, 0.251 mmol), 0.5 mL of THF and $KBHEt_3$ (15 µL, 1M in THF, 0.0150 mmol), stir at r.t. for 12 hours, the reaction was quenched by PE. The mixture was filtered through a pad of silica gel and washed with ether (50 mL). The combined filtrates were concentrated in vacuo and yields were monitored by $^1$H NMR analysis. The residue was purified by preparative TLC (PE/EA/TEA = 100/5/2 (v/v/v), $R_f$ = 0.40) to afford **6** (0.0903 g, 96% yield) as a colorless oil. The D-incorporation was determined by $^1$H NMR and $^2$H NMR.

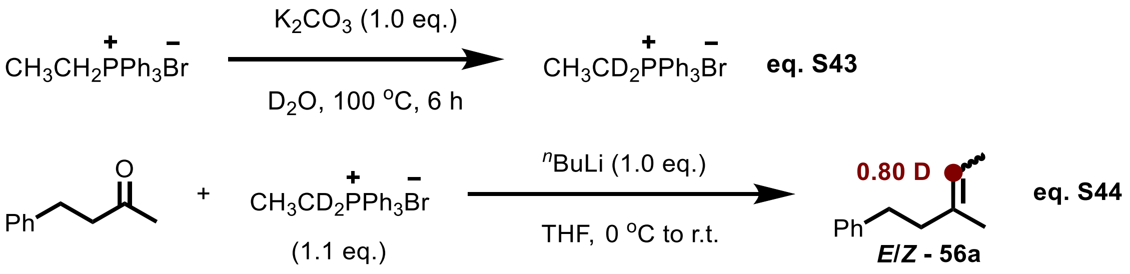


Eq. S44 was prepared according to the modification of **method B**, benzylacetone (5 mmol) and 57a-s (5.5 mmol) *which was synthesized by consulting previous literature* (*68*) were used as starting materials, the reaction afforded 0.34 g (*E* / *Z* = 1.1 / 1.0, 43% yield) of **57a** as a colorless oil. $^1$H NMR: (400 MHz, $CDCl_3$) $\delta$ 7.29-7.17 (m, 5H), 5.23 (q, $J$ = 6.8 Hz, 0.2H), 2.72-2.65 (m, 2H), 2.34-2.25 (m, 2H), 1.72 (s, 1.4 for *Z* isomer), 1.66 (s, 1.6H for *E* isomer), 1.57 (s, 1.6H), 1.47 (s, 1.4H); *The E/Z ratio of 56a was inferred from previous rules(50)*

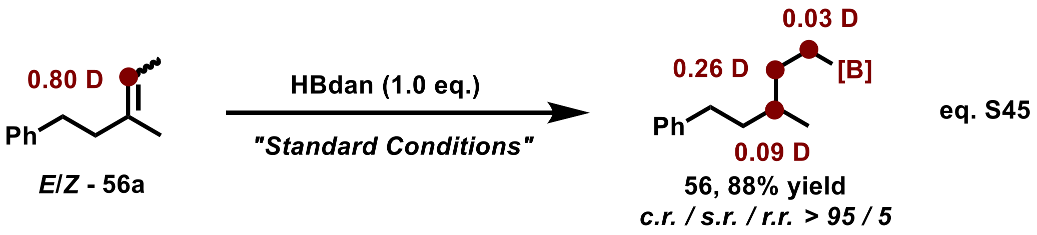

Eq.S45 was prepared according to the modification of general procedure: a 10 mL flame-dried flask was cooled at room temperature under nitrogen, charged with using **L9**•$CoCl_2$ (0.0042 g, 0.0055 mmol), HBdan (0.0423 g, 0.252 mmol), **56a** (0.0403 g, 0.250 mmol), 0.5 mL of THF and $KBHEt_3$ (15 μL, 1M in THF, 0.0150 mmol), stir at r.t. for 12 hours, the reaction was quenched by PE. The mixture was filtered through a pad of silica gel and washed with ether (50 mL). The combined filtrates were concentrated in vacuo and yields were monitored by $^1H$ NMR analysis. The residue was purified by preparative TLC (PE/EA/TEA = 100/11/2 (v/v/v), $R_f$ = 0.60) to afford **56** (0.0727 g, 88% yield) as a colorless oil. The D-incorporation was determined by $^1H$ NMR and $^2H$ NMR. 20 μL (0.1108 mmol) of $CDCl_3$ was added to a solution of **56** (0.220 mmol in $CHCl_3$).

## $^1H$ NMR-spectrum (400 MHz, $CDCl_3$) of 56

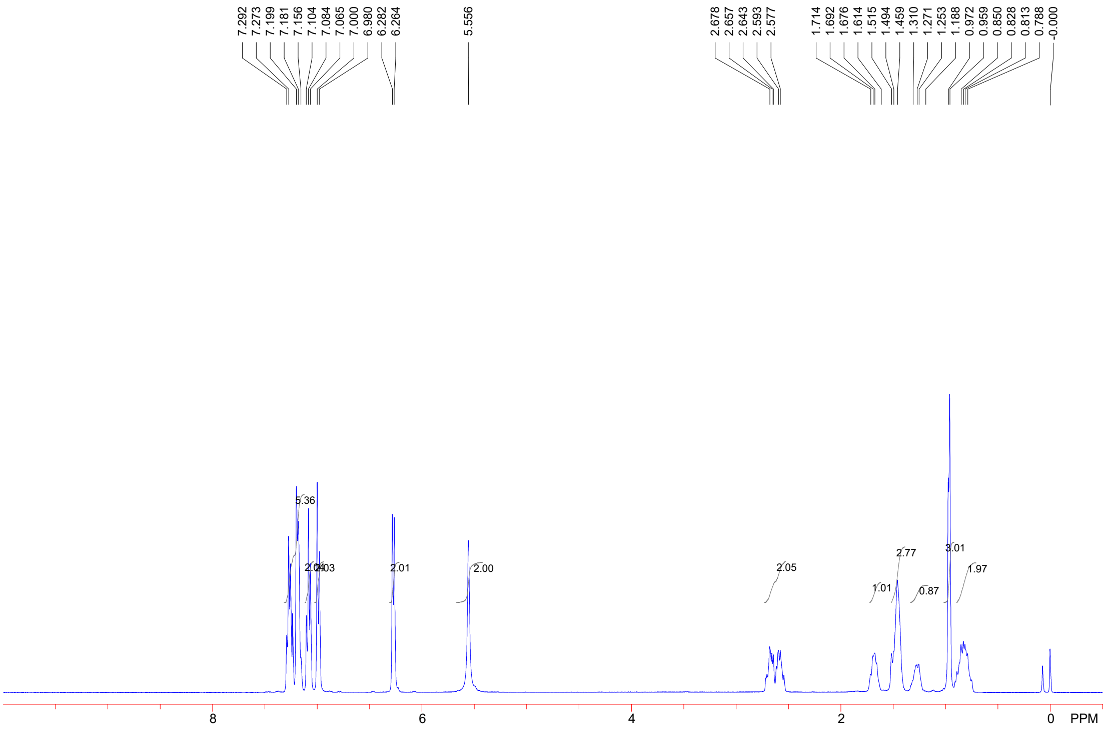


## $^2H$ NMR-spectrum (92 MHz, $CHCl_3$) of 56

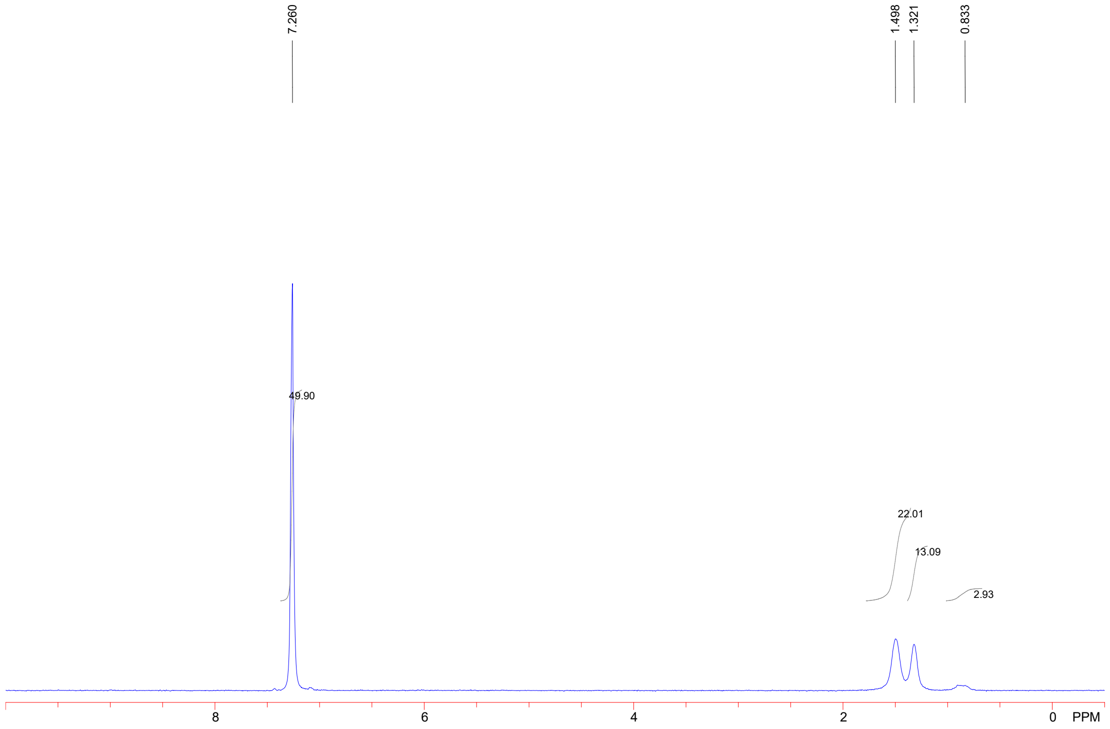


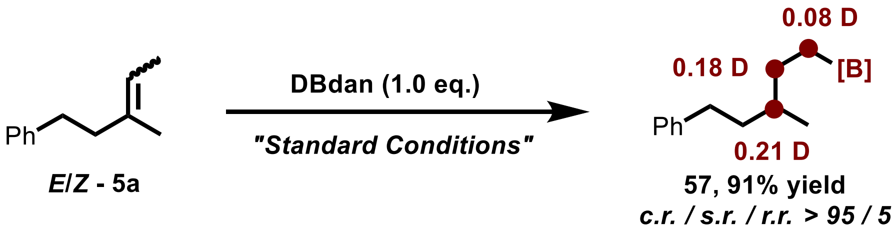


eq. S46

DBdan was prepared according to the previous procedure (*69*).

Eq.S46 was prepared according to the modification of general procedure: a 10 mL flame-dried flask was cooled at room temperature under nitrogen, charged with using **L9**•$CoCl_2$ (0.0041 g, 0.0054 mmol), DBdan (0.0430 g, 0.254 mmol), **5a** (0.0401 g, 0.251 mmol), 0.5 mL of THF and $KBHEt_3$ (15 µL, 1M in THF, 0.0150 mmol), stir at r.t. for 12 hours, the reaction was quenched by PE. The mixture was filtered through a pad of silica gel and washed with ether (50 mL). The combined filtrates were concentrated in vacuo and yields were monitored by $^1H$ NMR analysis. The residue was purified by preparative TLC (PE/EA/TEA = 100/11/2 (v/v/v), $R_f$ = 0.60) to afford **57** (0.0750 g, 91% yield) as a colorless oil. The D-incorporation was determined by $^1H$ NMR and $^2H$ NMR. 20 µL (0.1108 mmol) of $CDCl_3$ was added to a solution of **57** (0.2275 mmol in $CHCl_3$).

## $^1H$ NMR-spectrum (400 MHz, $CDCl_3$) of 57

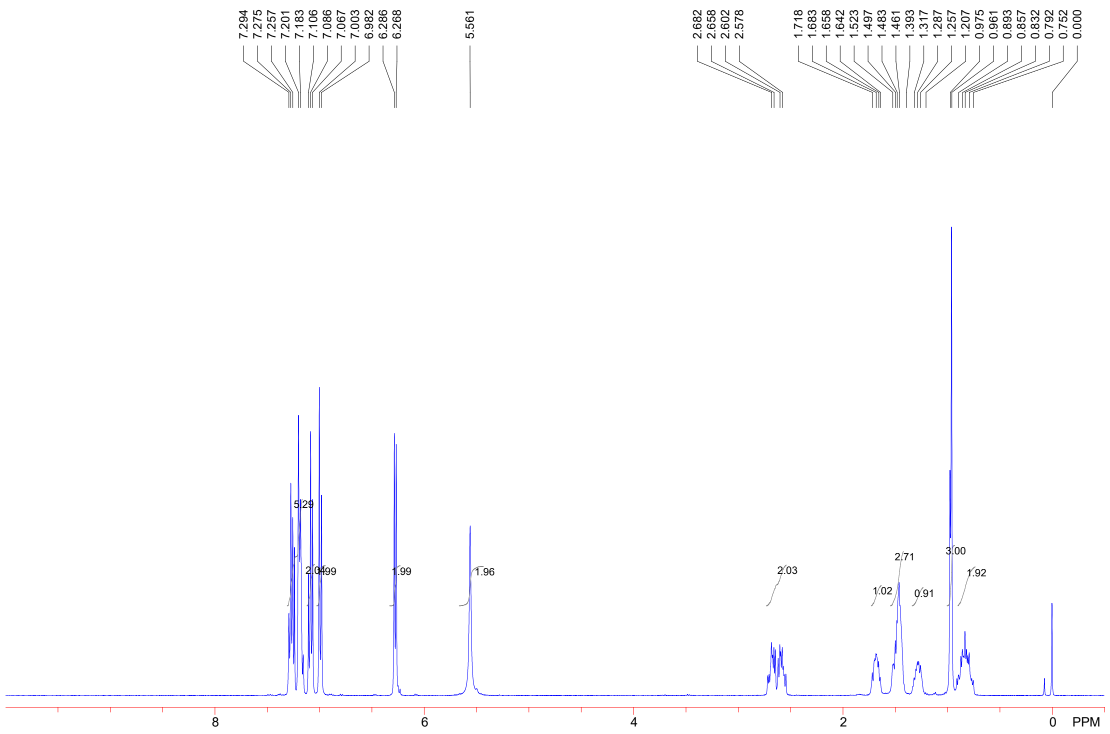


**$^2$H NMR-spectrum (92 MHz, $CHCl_3$) of 57**

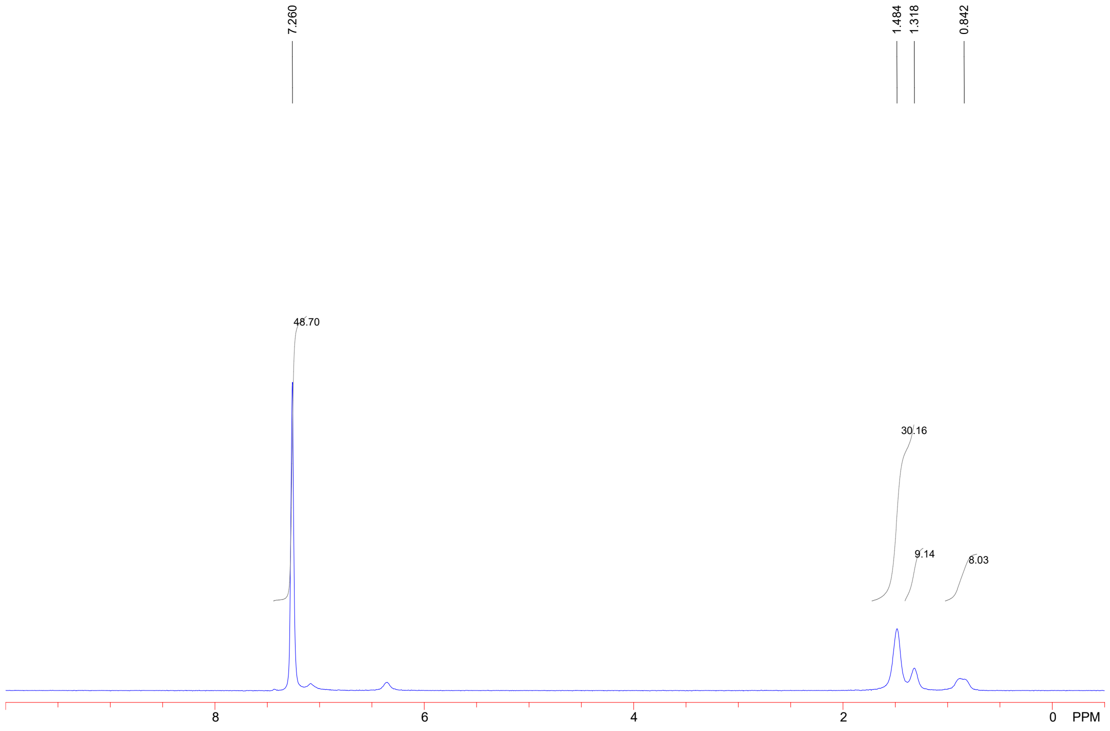


Alkene isomerization experiment:

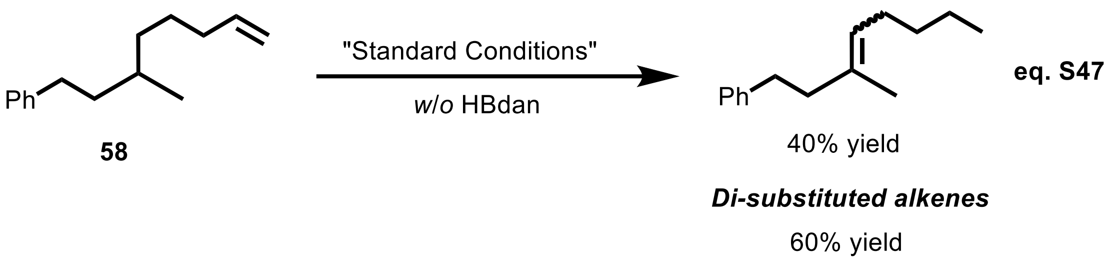


Eq.S47 was prepared according to the modification of general procedure: a 10 mL flame-dried flask was cooled at room temperature under nitrogen, charged with using **L9**•$CoCl_2$ (0.0038 g, 0.0050 mmol), **56** (0.0505 g, 0.250 mmol), 0.5 mL of THF and $KBHEt_3$ (15 µL, 1M in THF, 0.0150 mmol), stir at r.t. for 12 hours, the reaction was quenched by PE. The mixture was filtered through a pad of silica gel and washed with ether (50 mL). The combined filtrates were concentrated in vacuo and yields were monitored by $^1$H NMR analysis. Based on the speculated cobalt hydrogen species, the mixture of di-substituted alkenes and tri-substituted alkenes obtained indicates that the reaction underwent olefin isomerization process involving cobalt-hydrogen species.

## Effect of ligand design:

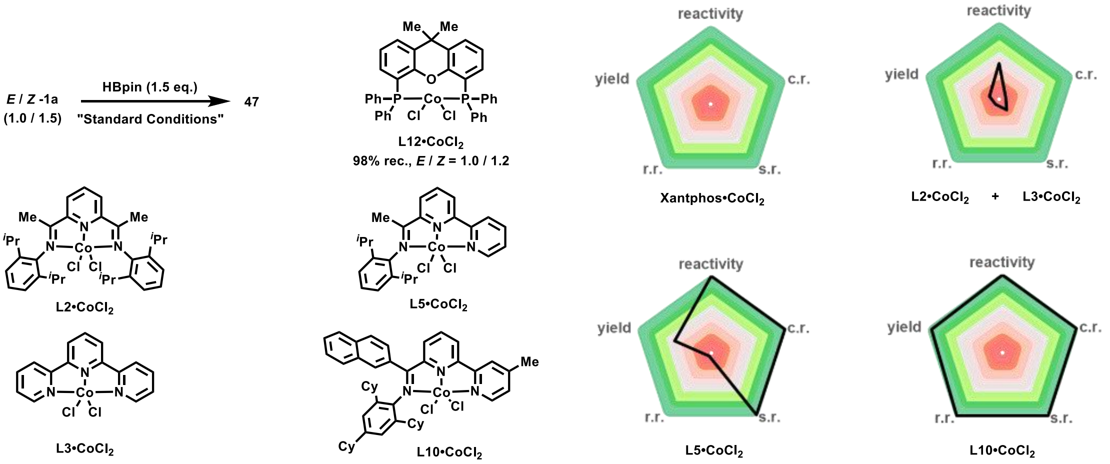


| Entry[a] | L•$CoCl_2$ | Conv. [b] (%) | Yield of **47** [b] (%) | Chemo Selectivity [b] | Site Selectivity [b] | Regio Selectivity [b] |
|---|---|---|---|---|---|---|
| 1 | **L12**•$CoCl_2$ | Trace | Trace | / | / | / |
| 2 | **(L2+L3)** •$CoCl_2$ | 29 | 13 | 62/38 | 76/24 | 59/41 |
| 3 | **L5**•$CoCl_2$ | 100 | 50 | >95/5 | >95/5 | 50/50 |
| 4 | **L10**•$CoCl_2$ | 100 | 96 | >95/5 | >95/5 | >95/5 |

Through literature research (*49*), we found that the common dual phosphorus ligand (Xantphos) (*70*) can also be used as a catalytic system for metal cobalt catalyzed olefin isomerization reaction. Using **1a** as the reaction raw material, we can only obtain

equivalent amount of raw material recovery, but the *E*/*Z* ratio has changed, indicating that the insertion process of metal hydrogen into the olefin has occurred. The lack of product indicates that the phosphorus ligand cannot promote the occurrence of this reaction.

Furthermore, we investigated whether ligand module recombination is an absolute factor affecting the reaction. Surprisingly, when **L2** and **L3** were simultaneously introduced into the reaction, the activity, yield, and selectivity of the reaction were not ideal. On the contrary, when the recombinant ligand **L5** was used, the reaction was significantly improved. When the ligand **L10** was further modified with substituents, all five indicators of the reaction reached the ideal standards, which further illustrates the importance of a suitable ligand skeleton.

**Figure S13.** Proposed Mechanism

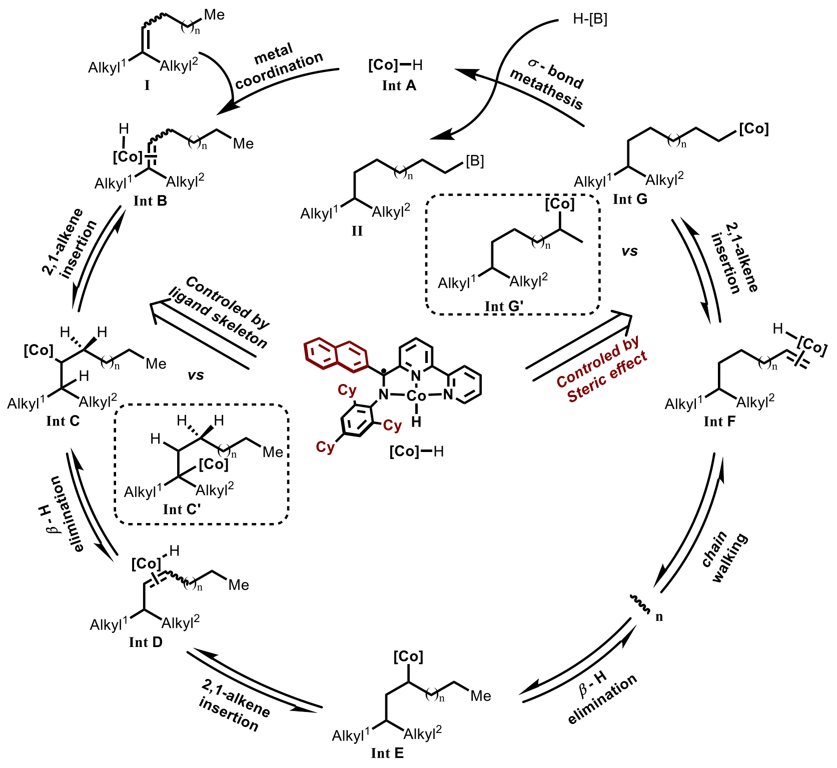


Based on the experimental studies and the previously reported literatures on hydroboration reactions (*38*), the probable mechanism was proposed. The active cobalt-hydride species **Int A** was generated from **Imbpy**-cobalt precatalyst in the presence of $KBHEt_3$. It was noted that a total of three equivalents of additives to the precatalyst was added: one equivalent of $KBHEt_3$ was used for the single-electron reduction of the

ligand skeleton, and the other equivalent was used for ligand exchange to form cobalt-hydride species, during which a slight excess of $KBHEt_3$ was added in order to enable the smooth production of cobalt-hydride species (*71*). Then **Int A** coordinated with trisubstituted alkenes to form key intermediate **Int B**. Then migratory 2,1-insertion of trisubstituted alkenes into the cobalt-hydride bond generated the 2° cobalt species **Int C**. Subsequent $\beta$-H elimination formed the **Int D**, then went through rapid chain-walking process to form **Int F**. The alkyl cobalt species **Int G** was obtained by specifically 2,1-insertion of the newly-formed C-C double bond into the cobalt-hydride bond. This species then underwent $\sigma$-bond metathesis with HBdan, producing procuct II and regenerating the cobalt-hydride species **Int A**.

**IX NMR Spectra: $^1$H NMR-spectrum (400 MHz, $CDCl_3$) of L7**

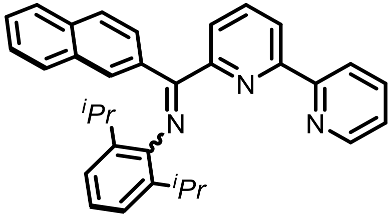


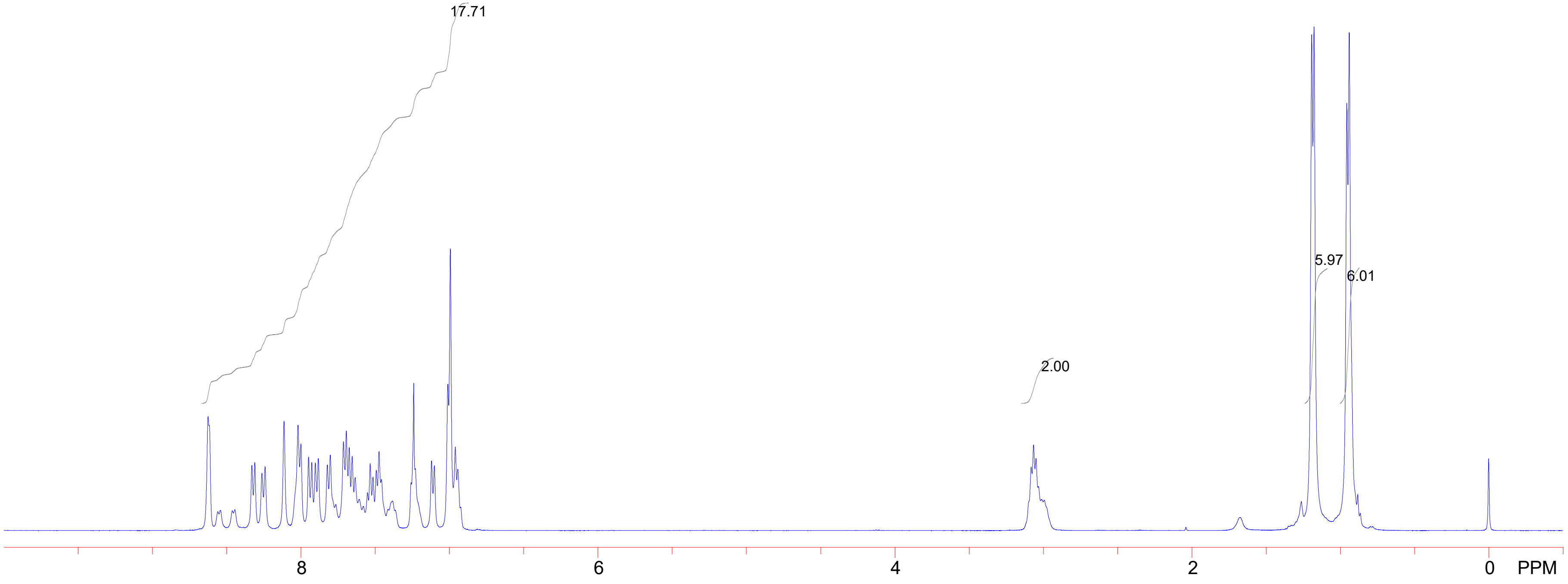

**$^{13}C$ NMR-spectrum (100 MHz, $CDCl_3$) of L7**

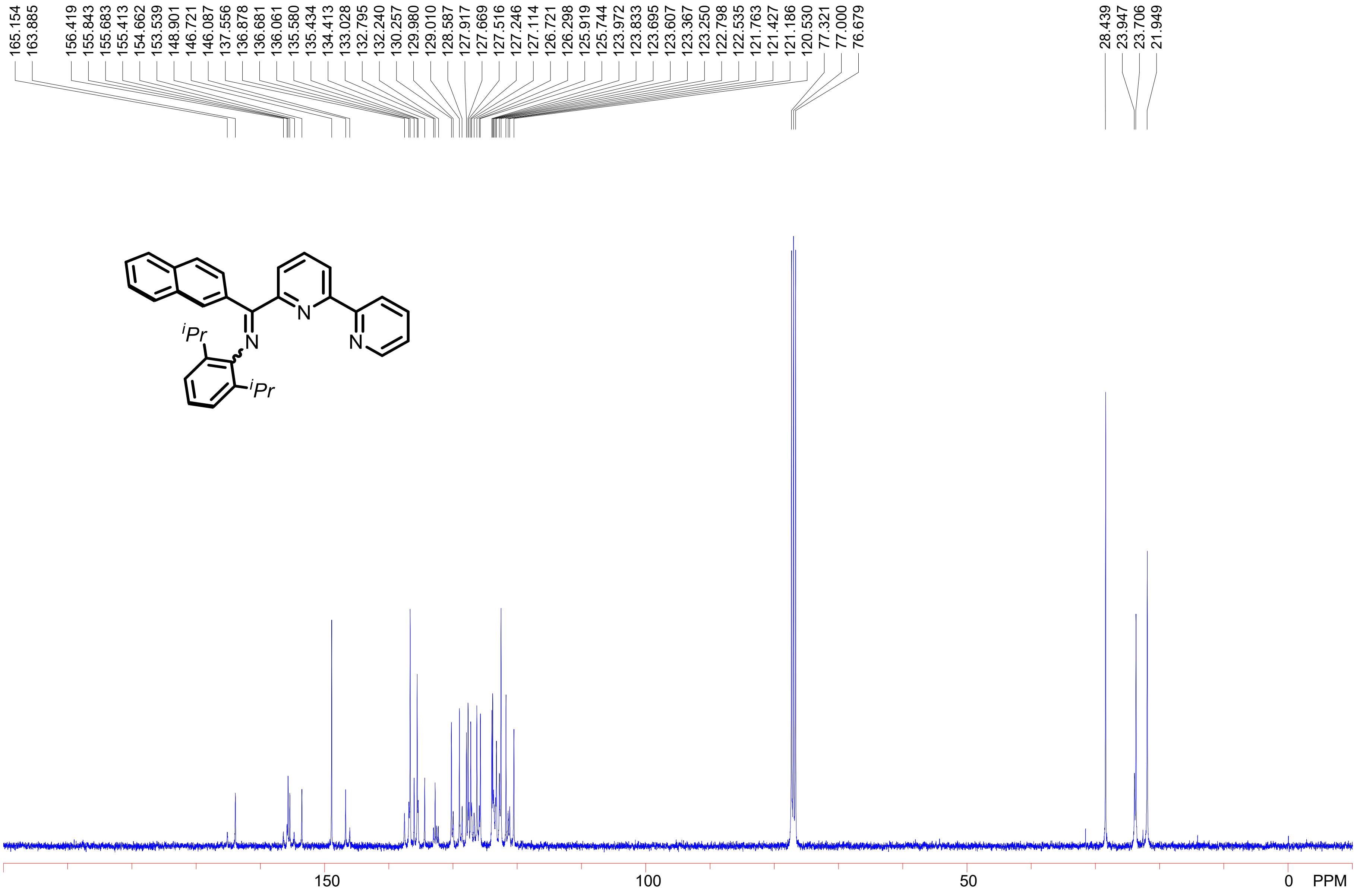

**$^{1}$H NMR-spectrum (400 MHz, $CDCl_3$) of L8**

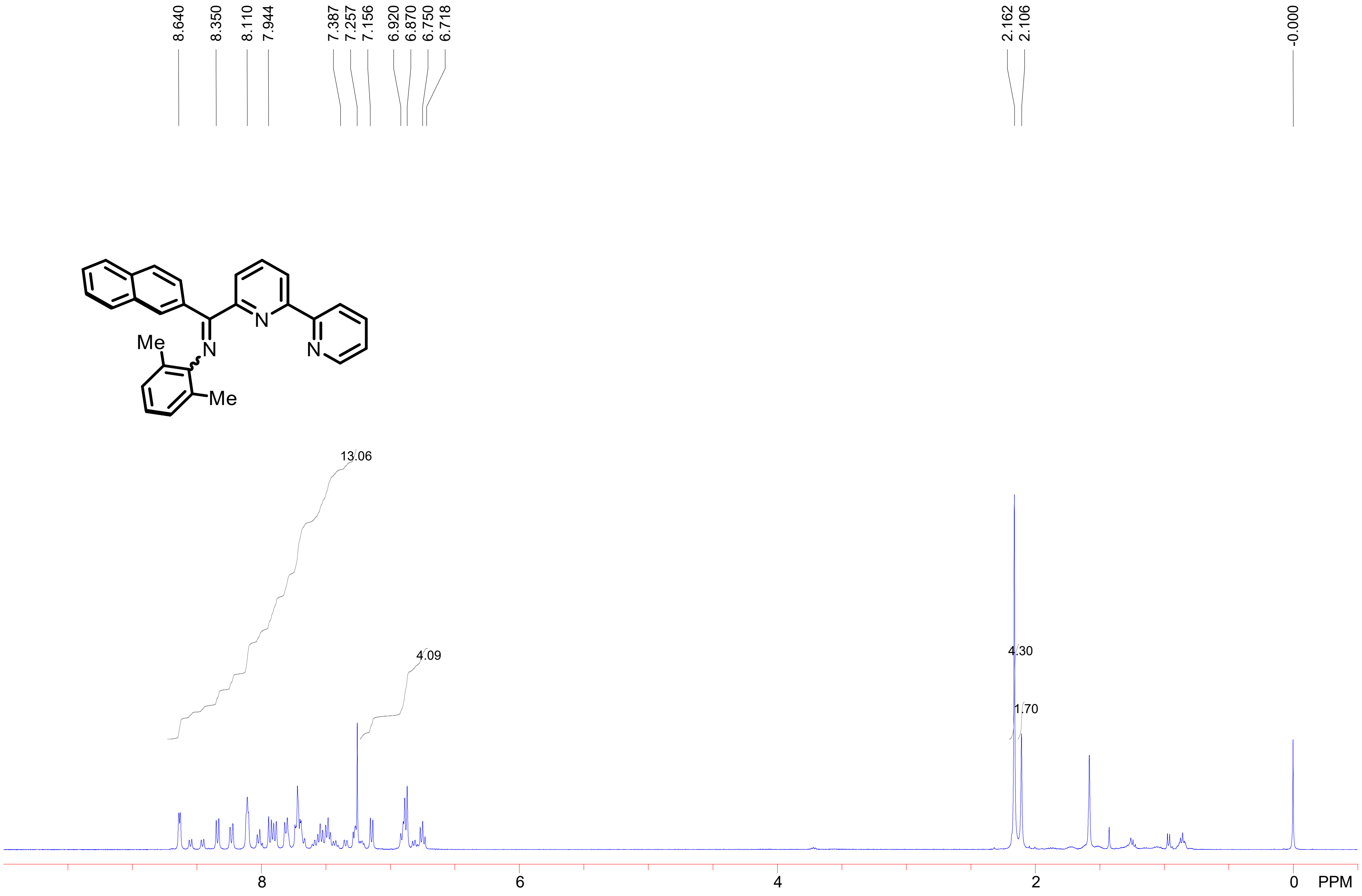

**$^{13}$C NMR-spectrum (100 MHz, $CDCl_3$) of L8**

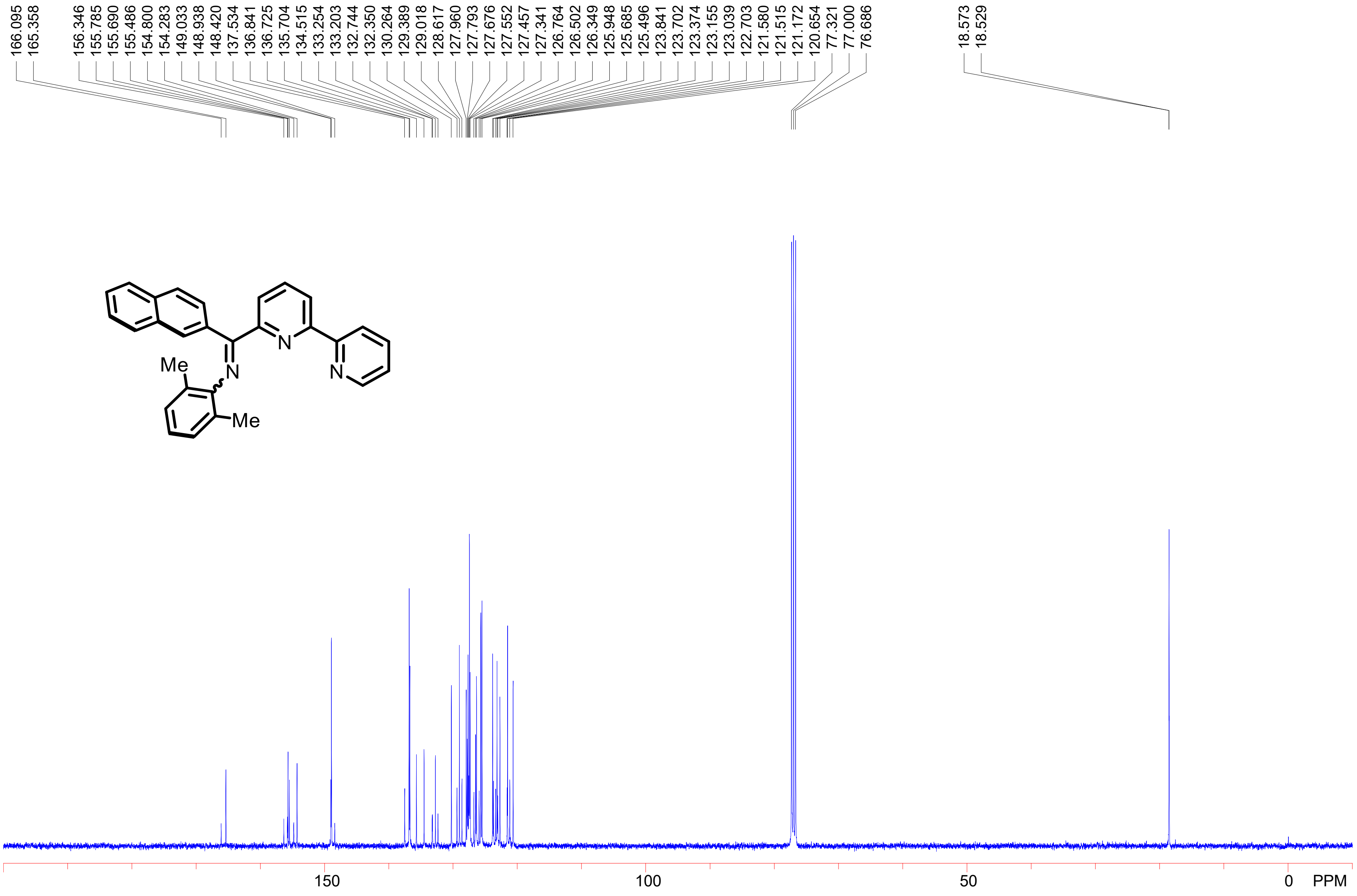

**$^{1}$H NMR-spectrum (400 MHz, $CDCl_3$) of L9**

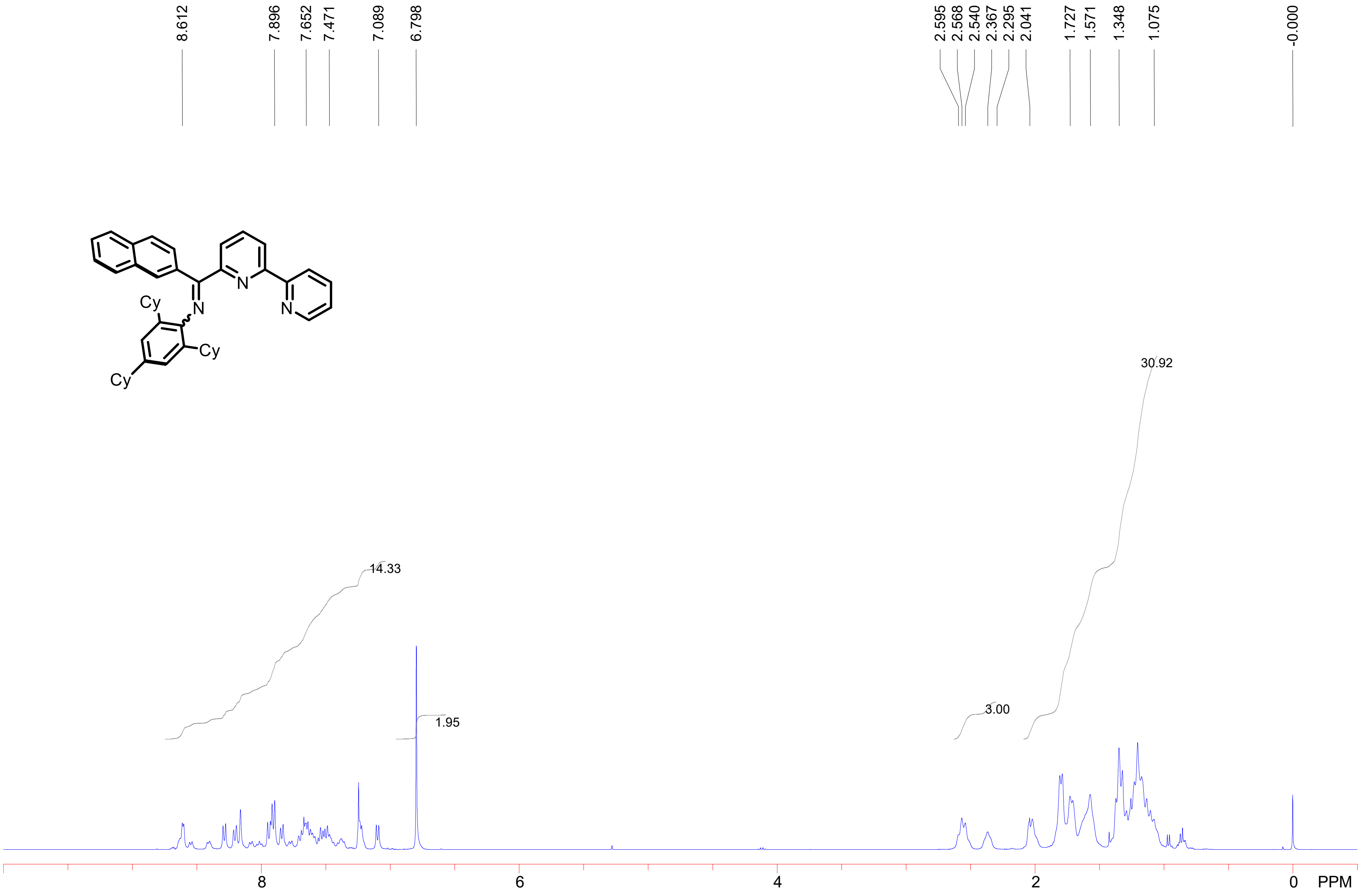

**$^{13}$C NMR-spectrum (100 MHz, $CDCl_3$) of L9**

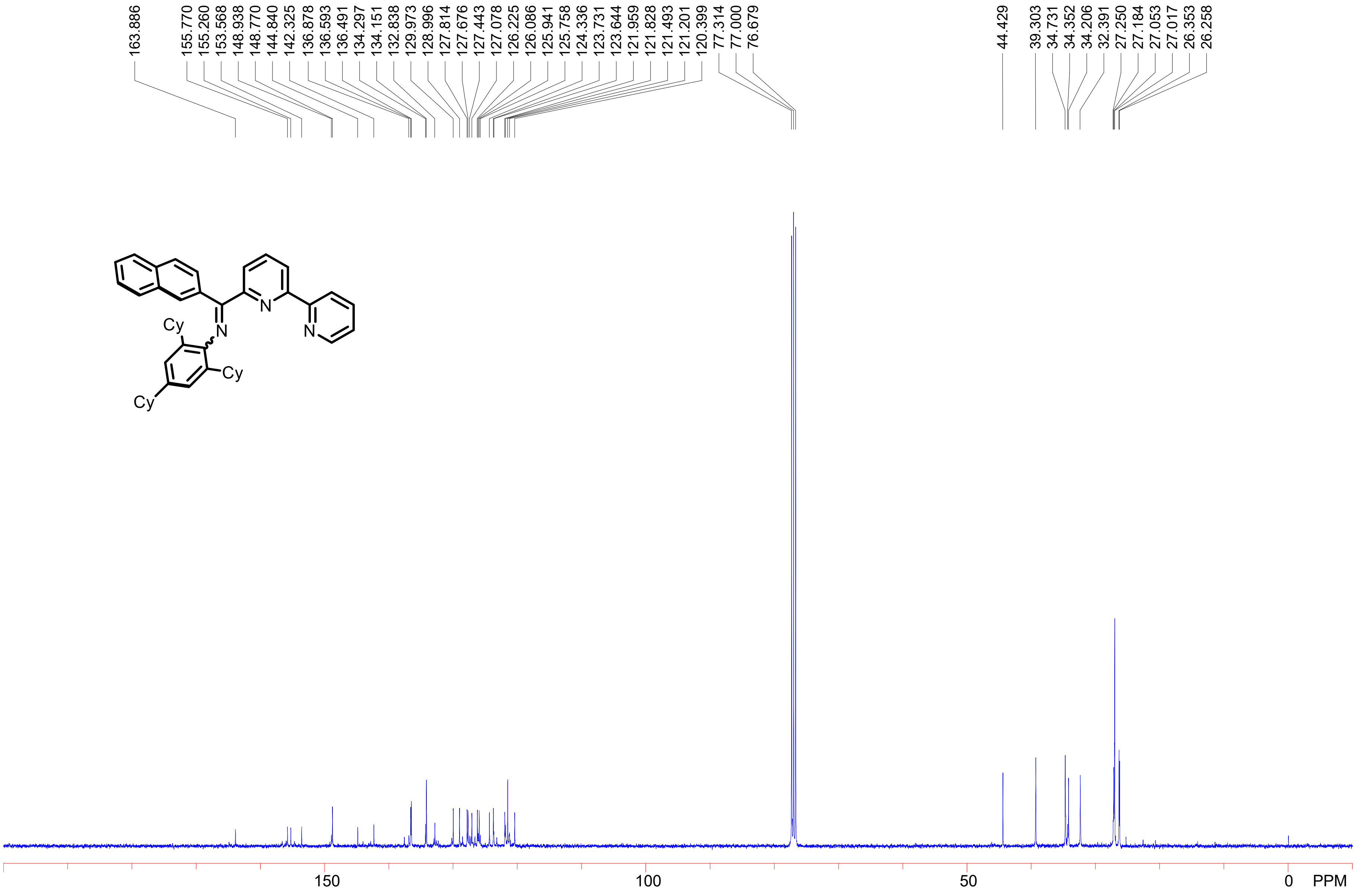

$^{1}$H NMR-spectrum (400 MHz, $CDCl_3$) of L10

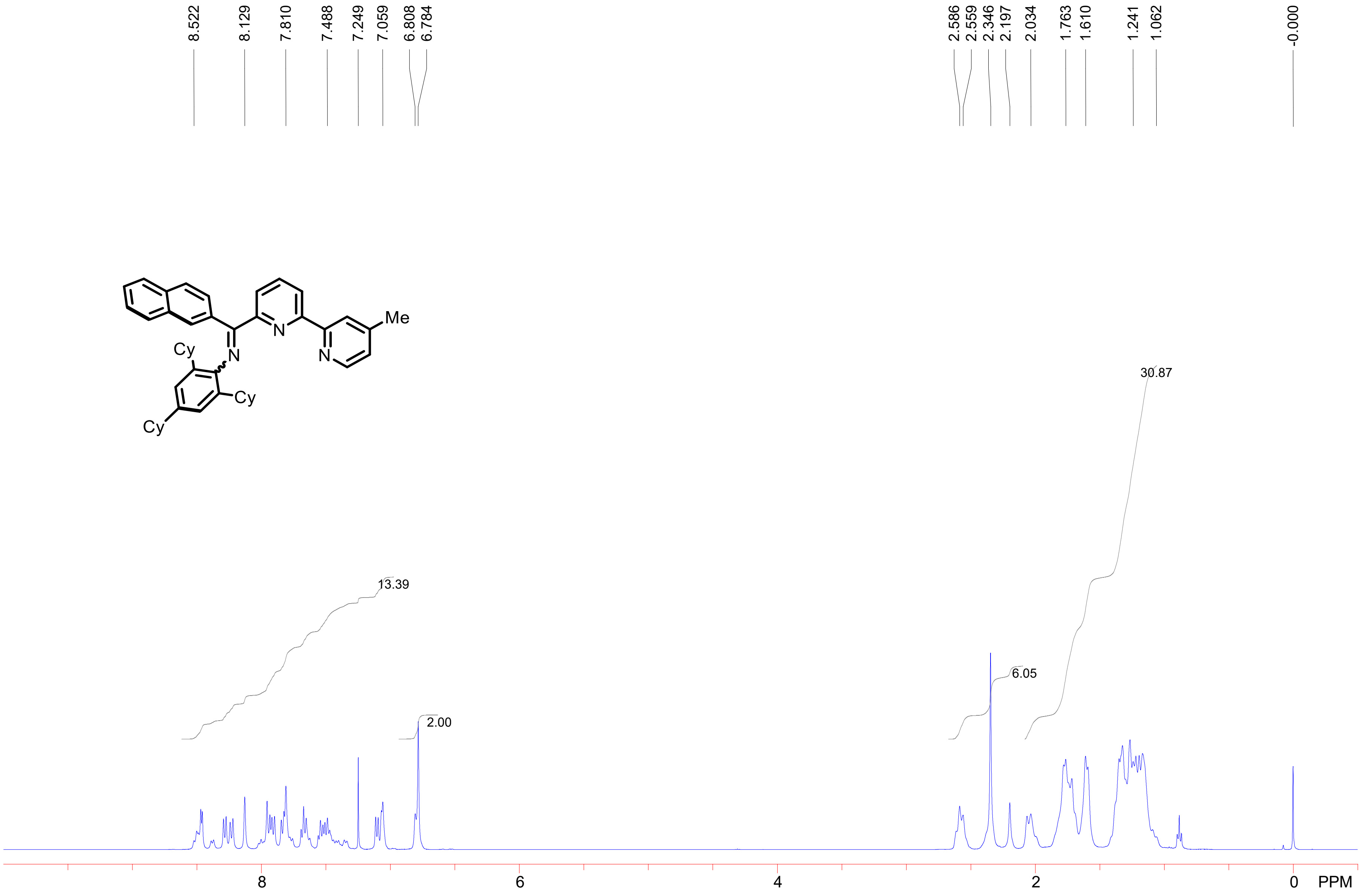

**$^{13}C$ NMR-spectrum (100 MHz, $CDCl_3$) of L10**

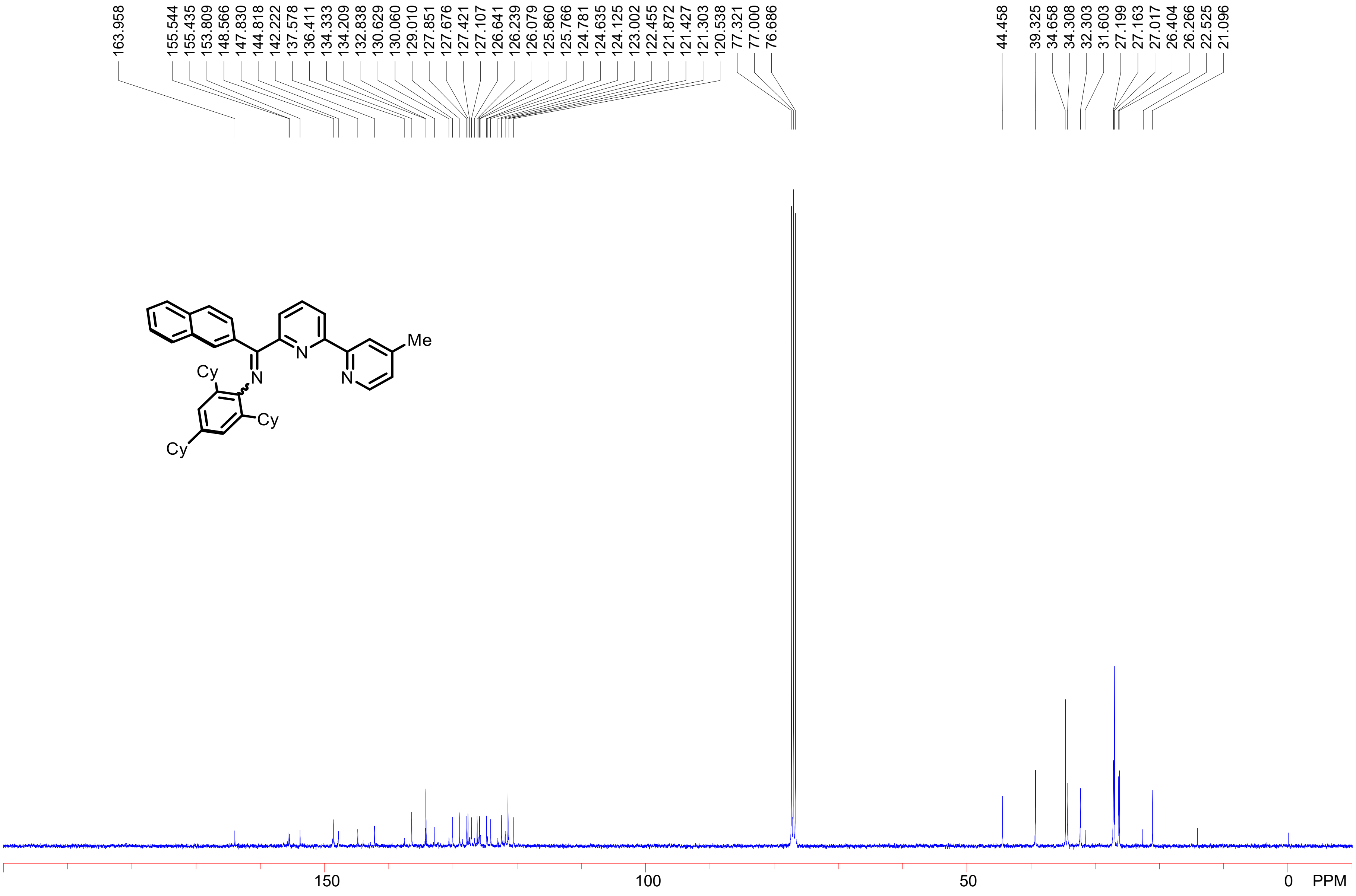

# $^{1}$H NMR-spectrum (400 MHz, $CDCl_3$) of 1a

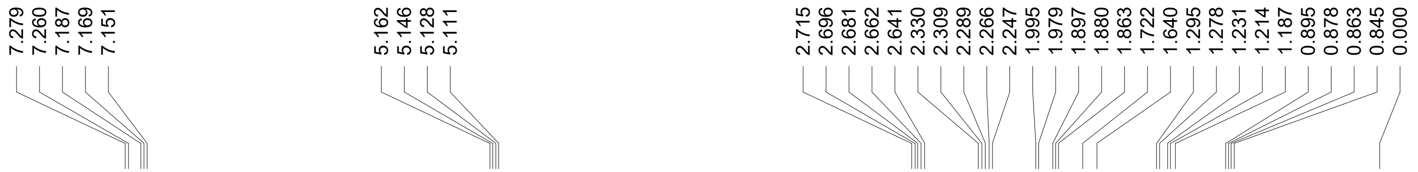


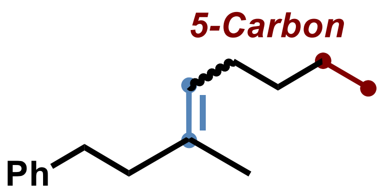


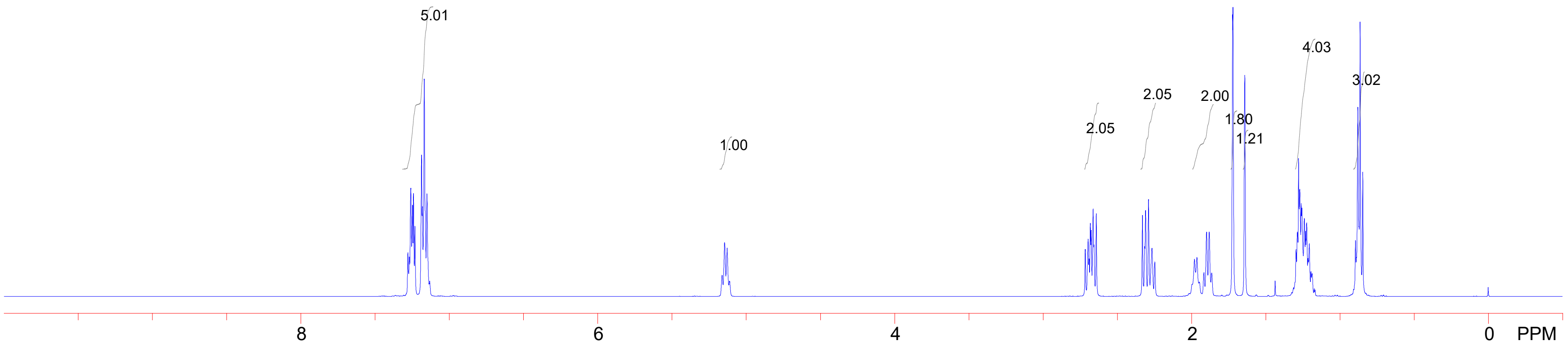

$^{13}$C NMR-spectrum (100 MHz, $CDCl_3$) of 1a

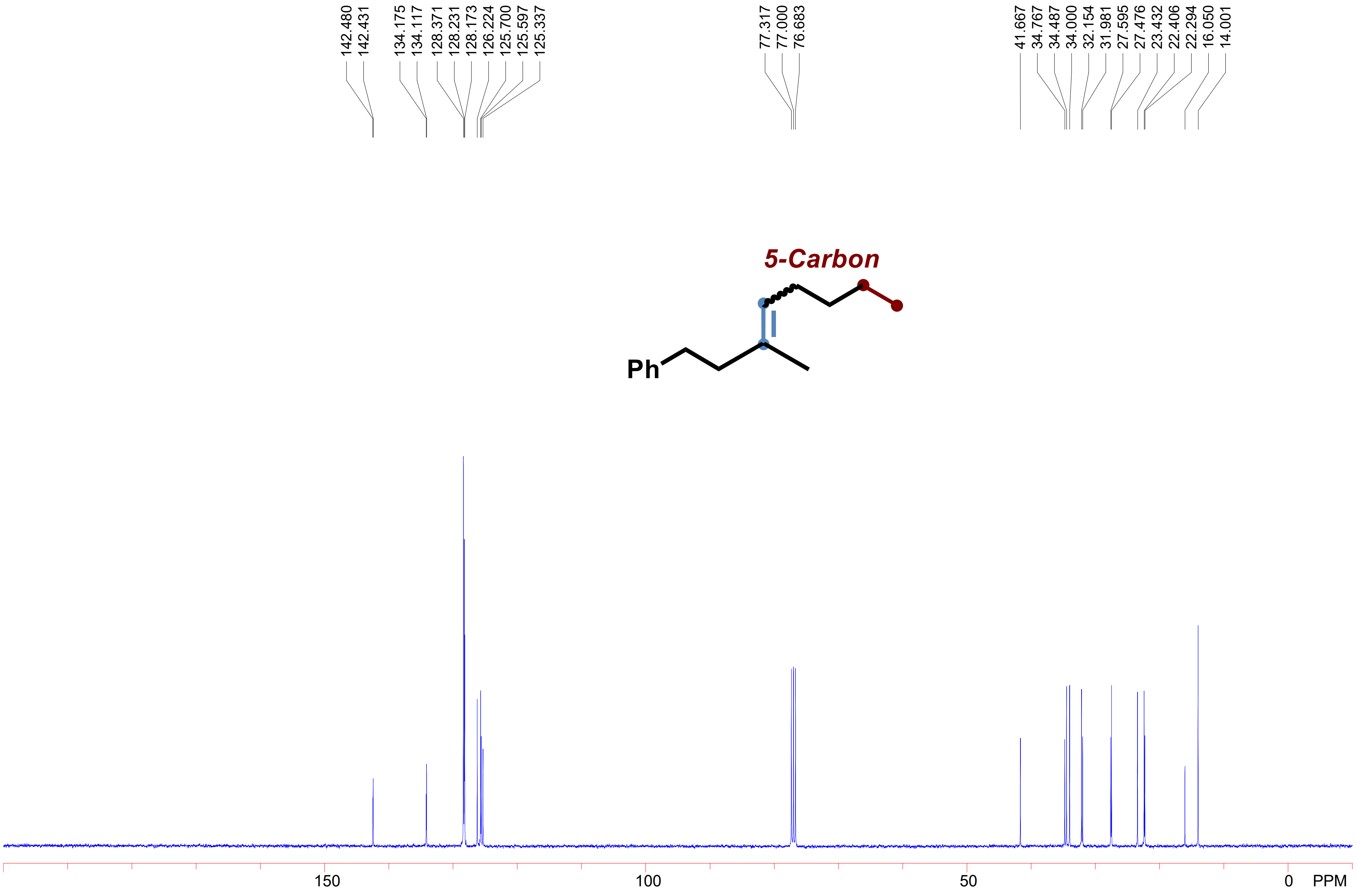

# $^{1}H$ NMR-spectrum (400 MHz, $CDCl_3$) of 5a

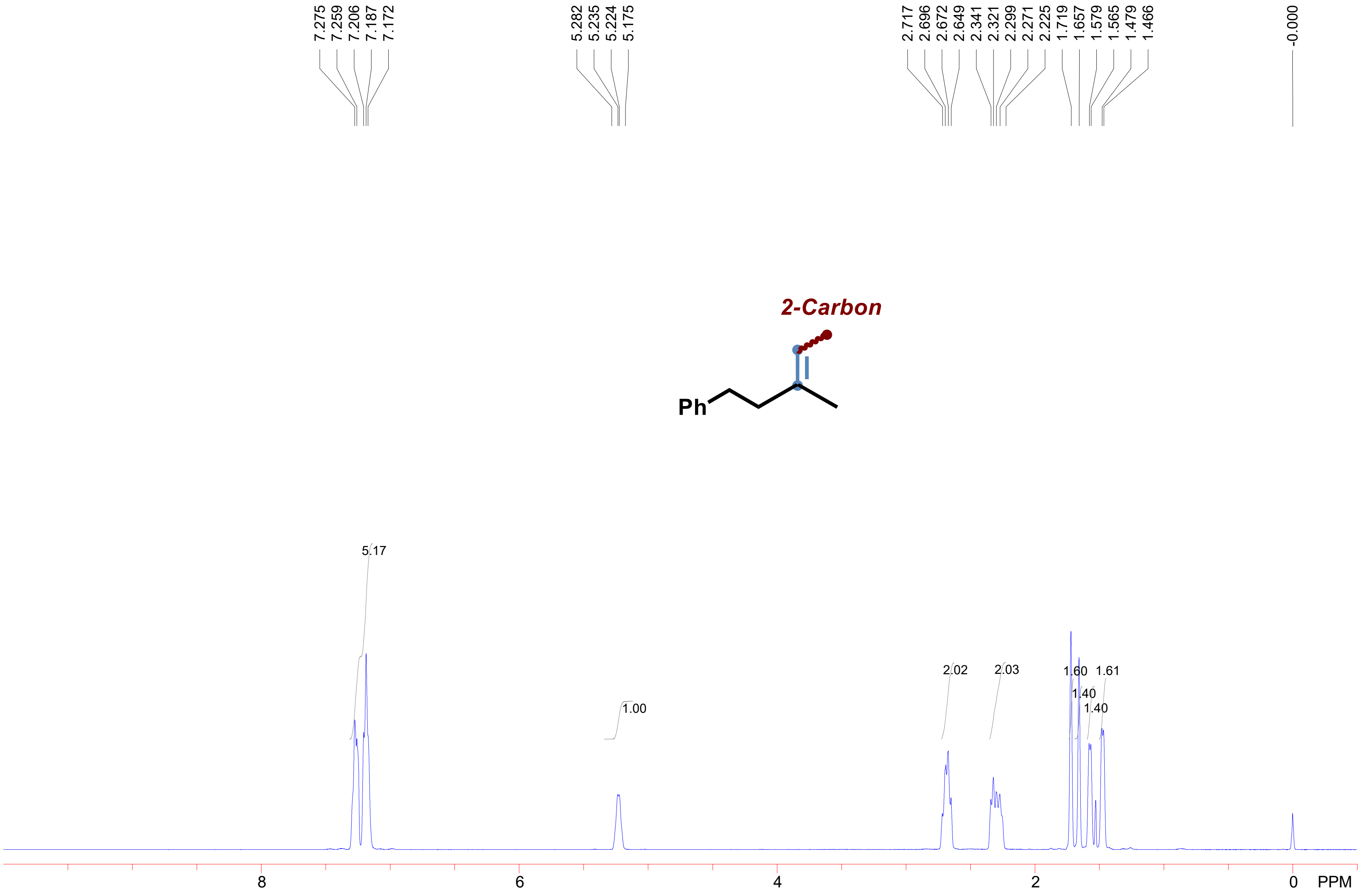

$^{13}$C NMR-spectrum (100 MHz, $CDCl_3$) of 5a

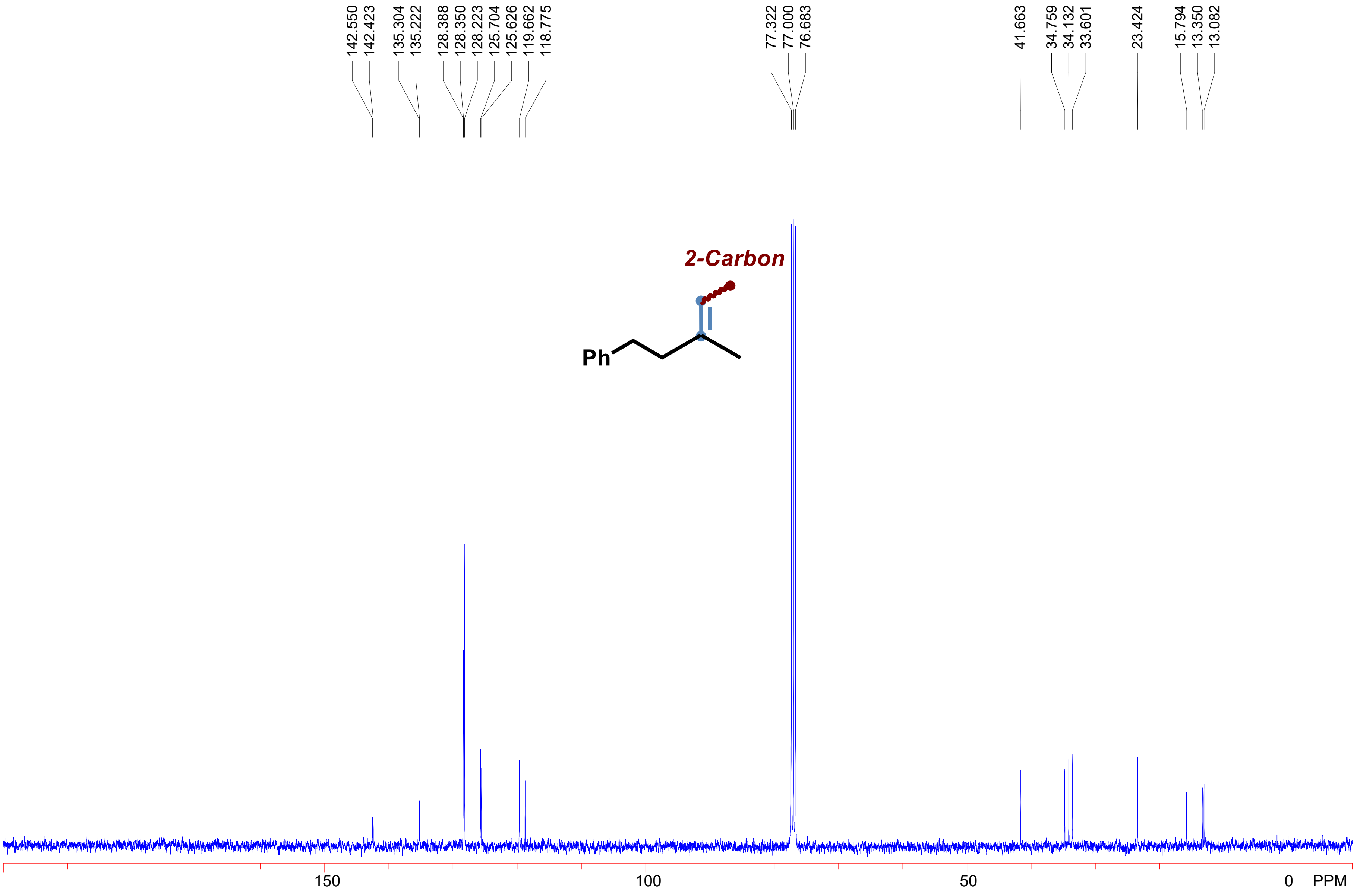

**$^{1}$H NMR-spectrum (400 MHz, CDCl3) of 6a**

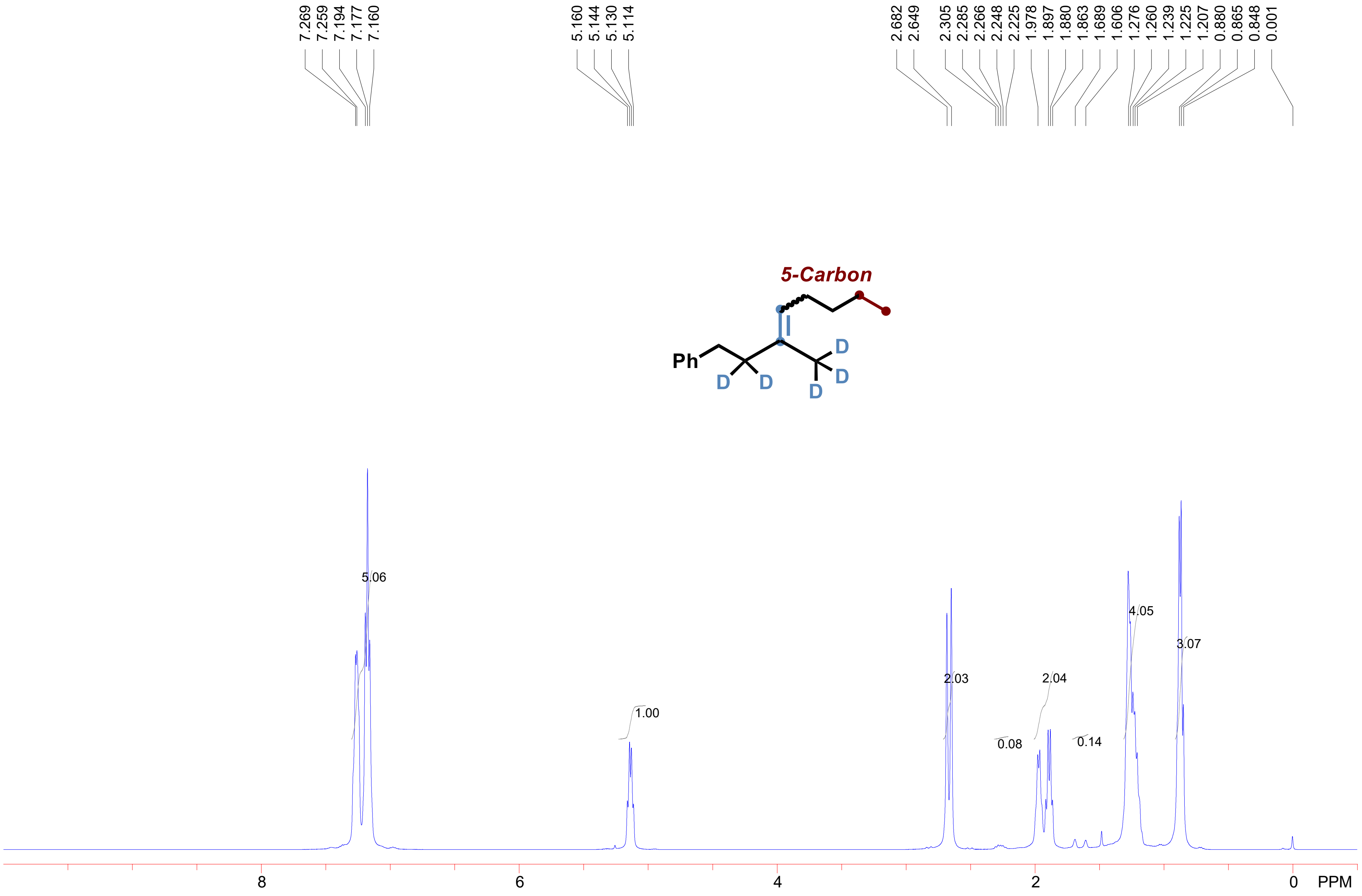

$^{2}$H NMR-spectrum (92 MHz, $CDCl_3$) of 6a

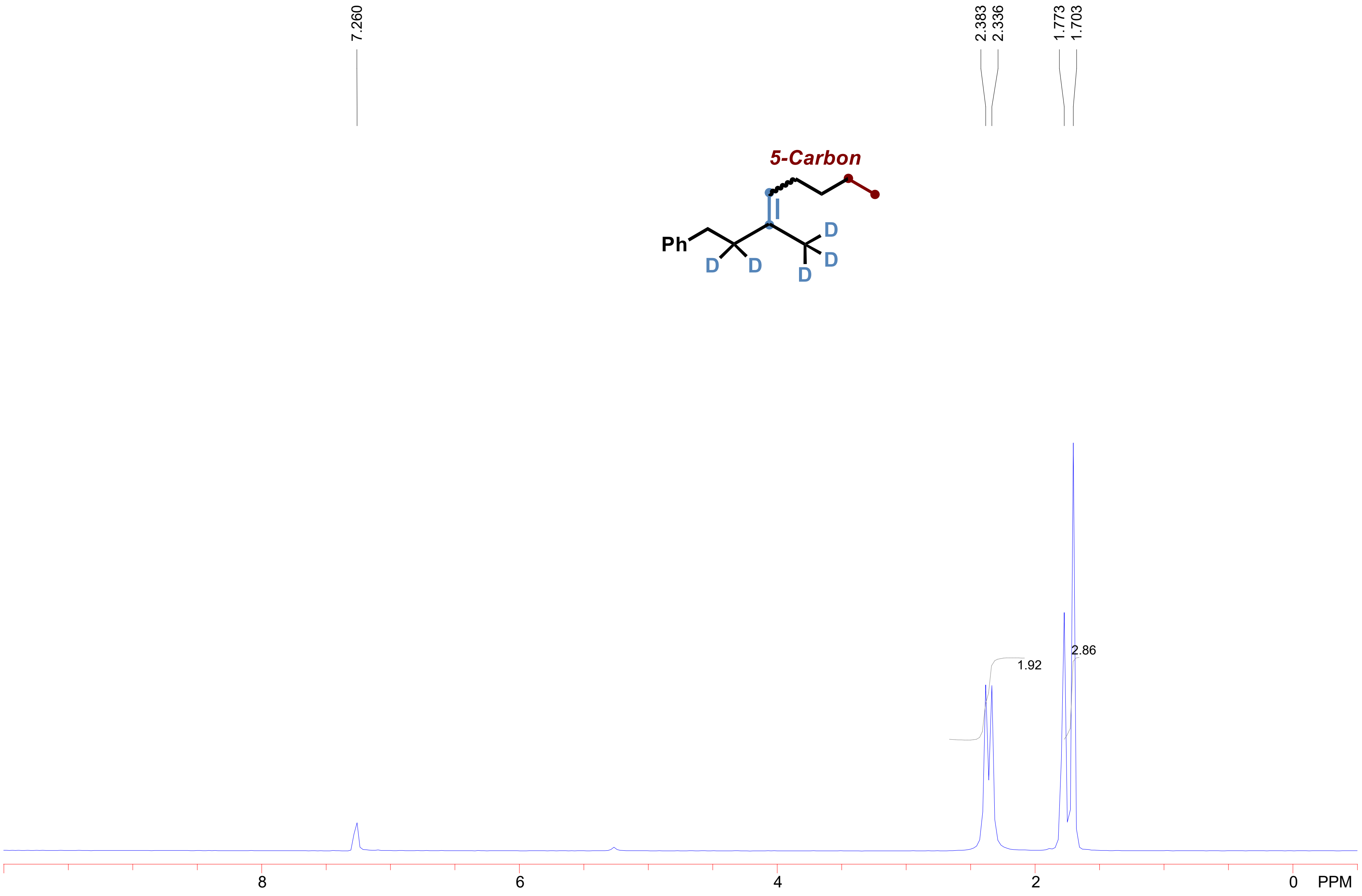

## $^{13}C$ NMR-spectrum (100 MHz, $CDCl_3$) of 6a

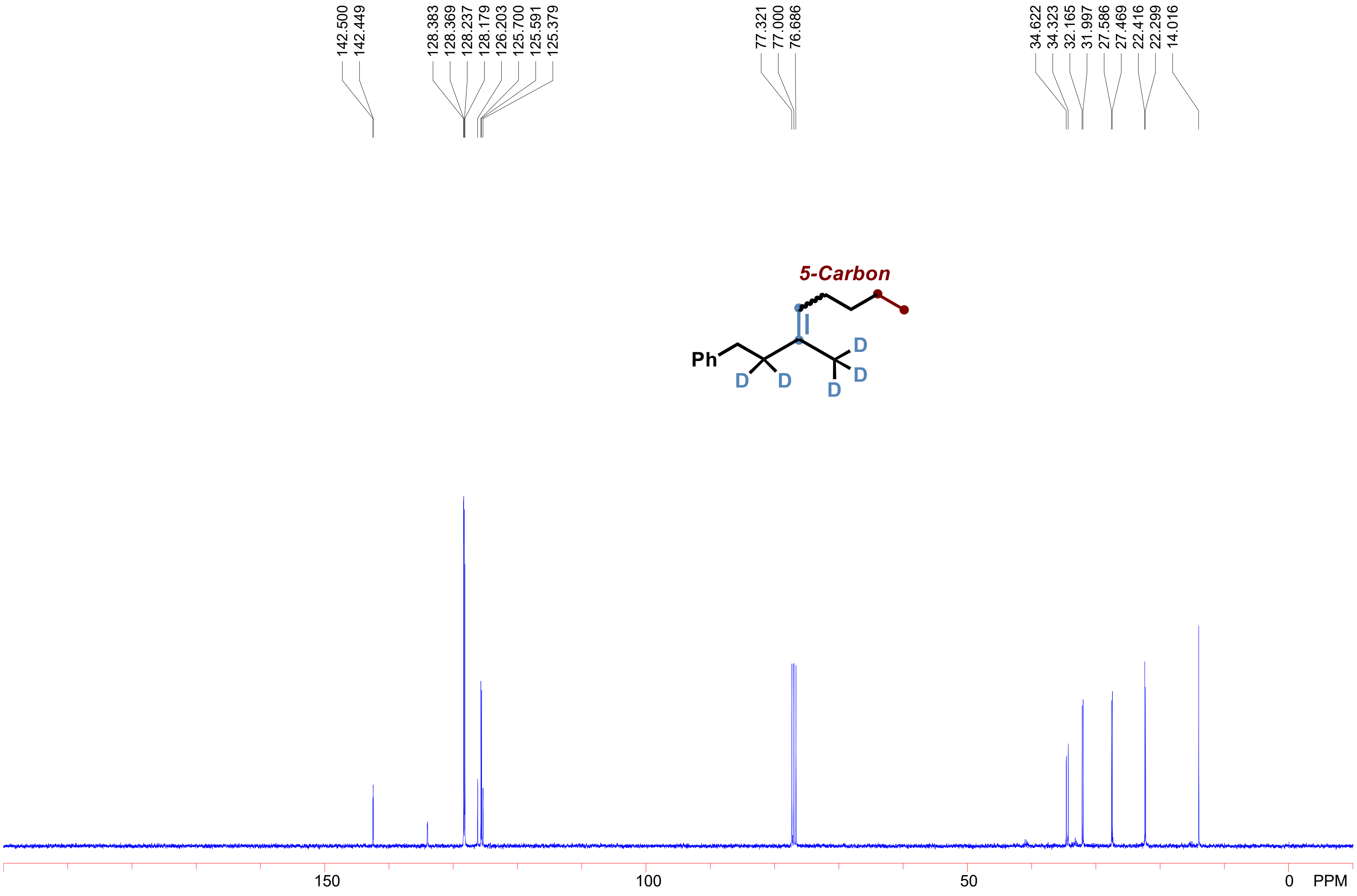

**$^{1}$H NMR-spectrum (400 MHz, CDCl3) of 7a**

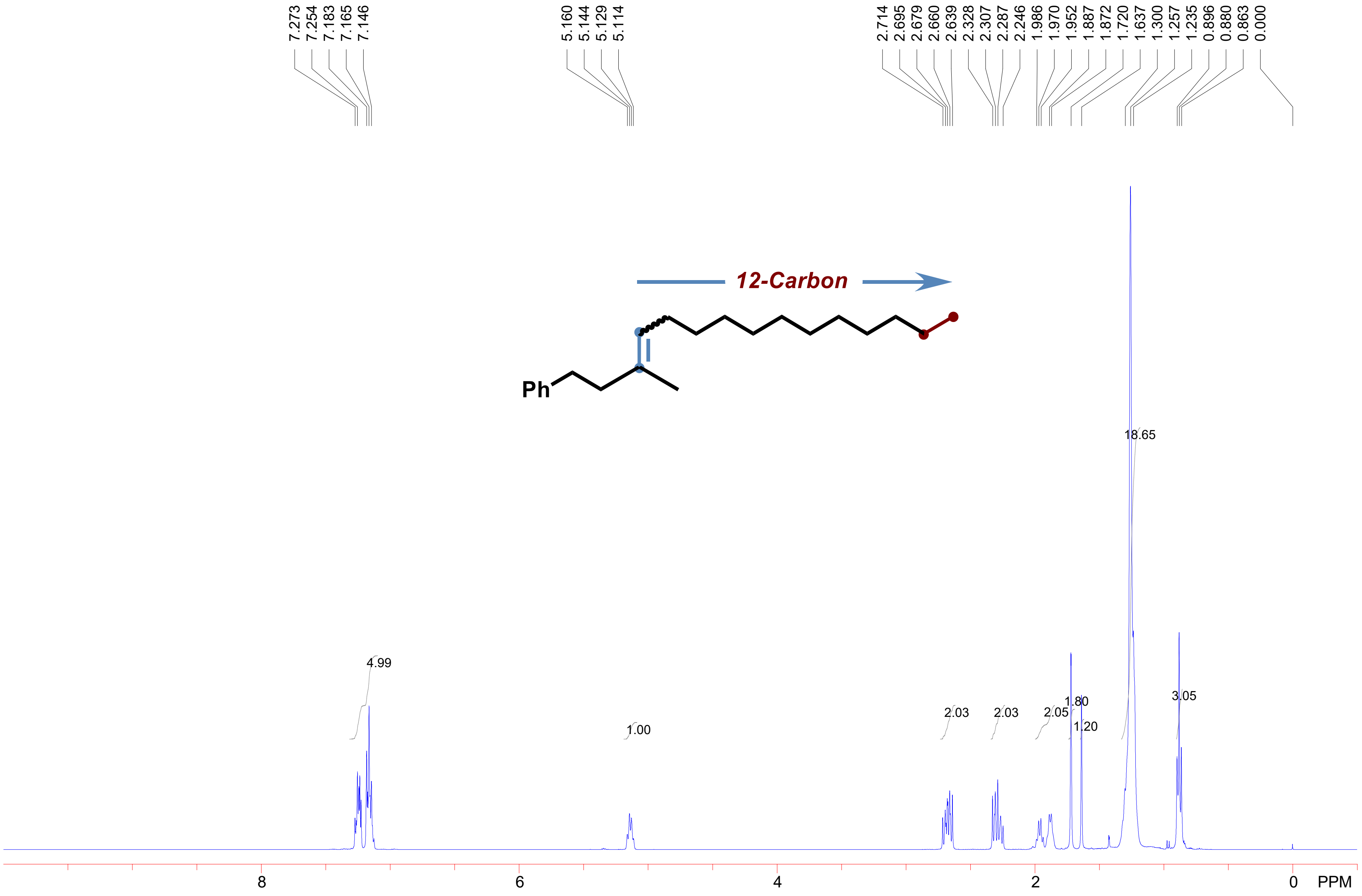

$^{13}$C NMR-spectrum (100 MHz, $CDCl_3$) of 7a

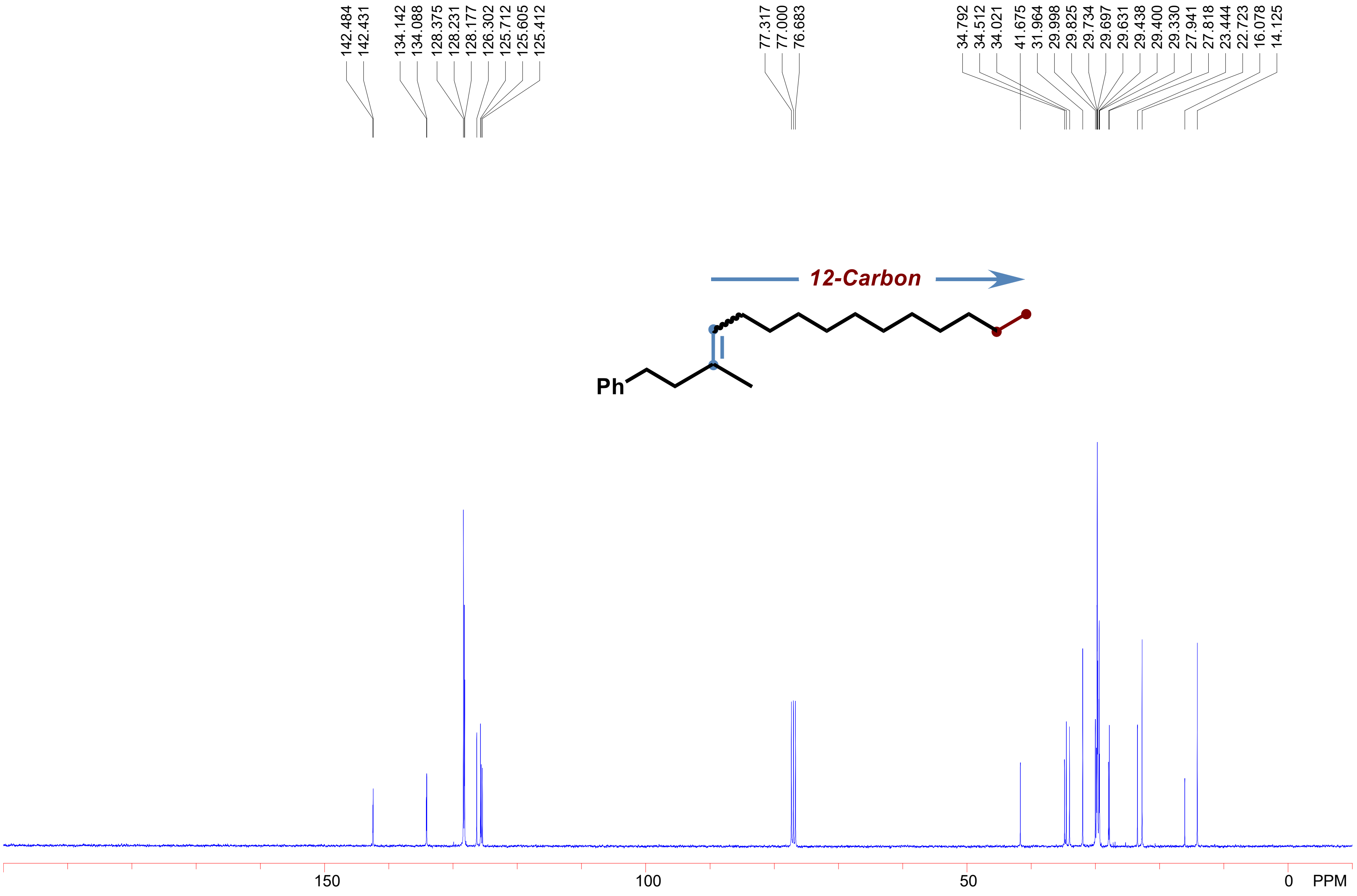

$^1$H NMR-spectrum (400 MHz, $CDCl_3$) of 8a

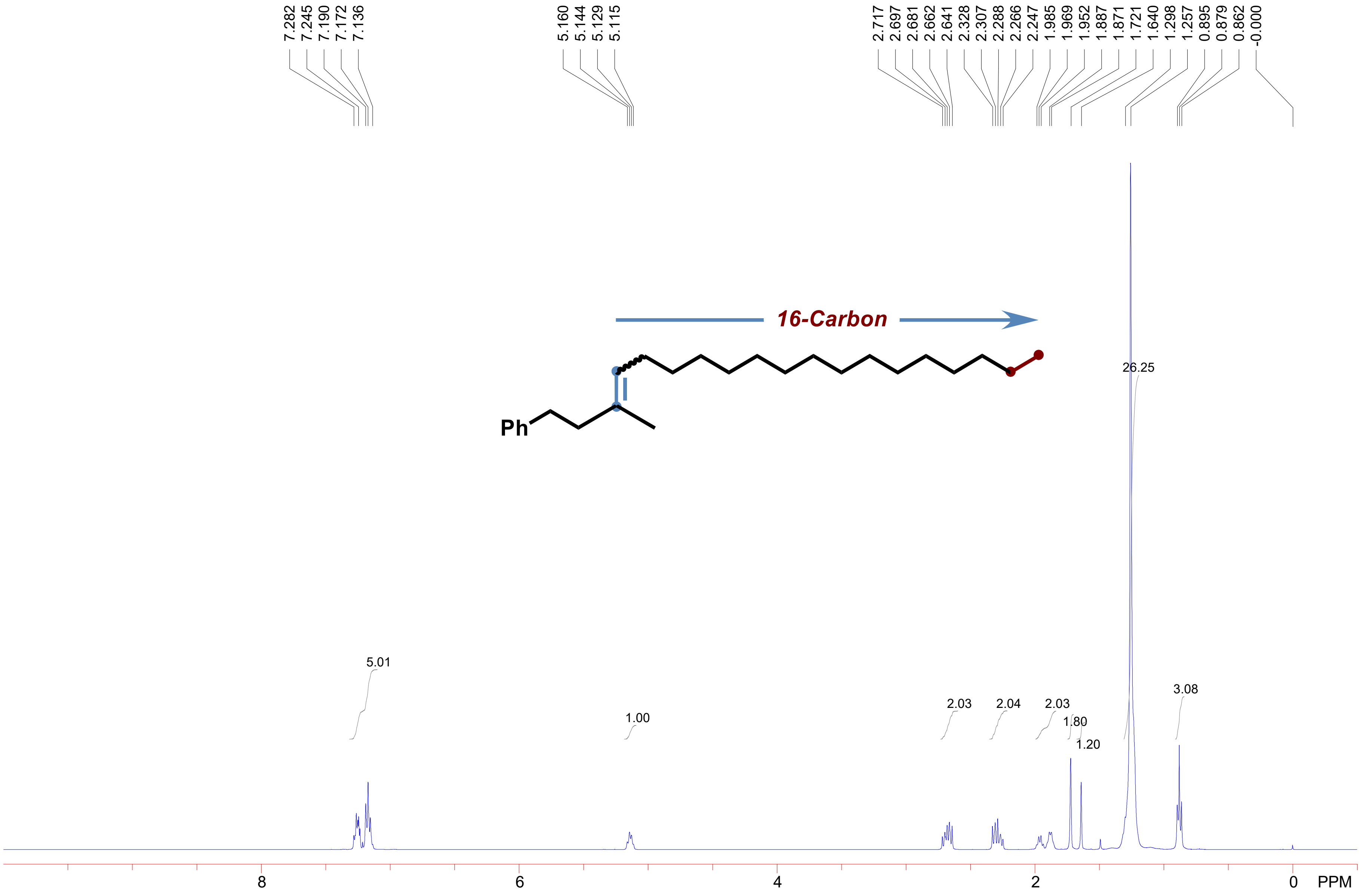

$^{13}C$ NMR-spectrum (100 MHz, $CDCl_3$) of 8a

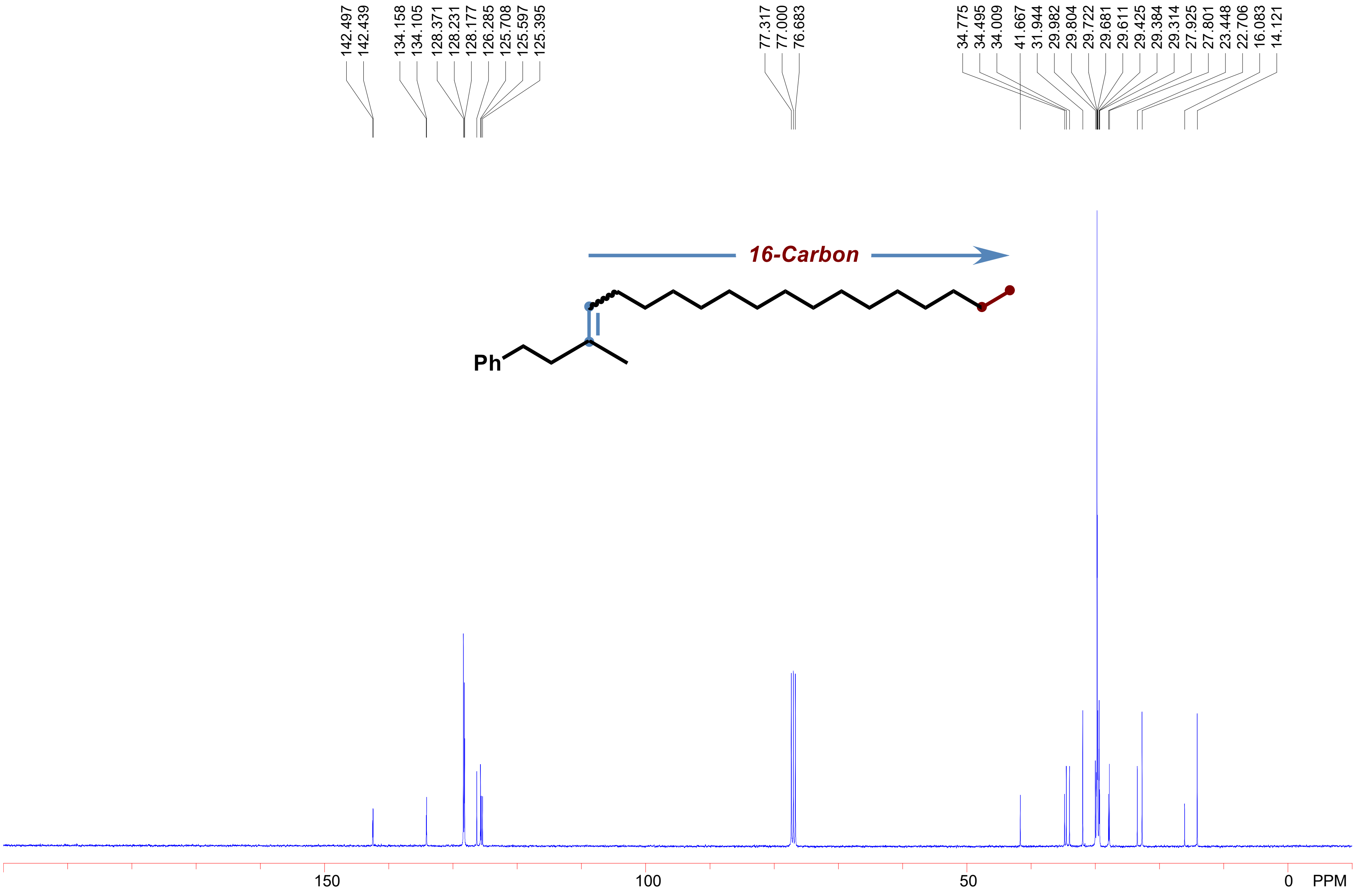

$^{1}H$ NMR-spectrum (400 MHz, $CDCl_3$) of 9a

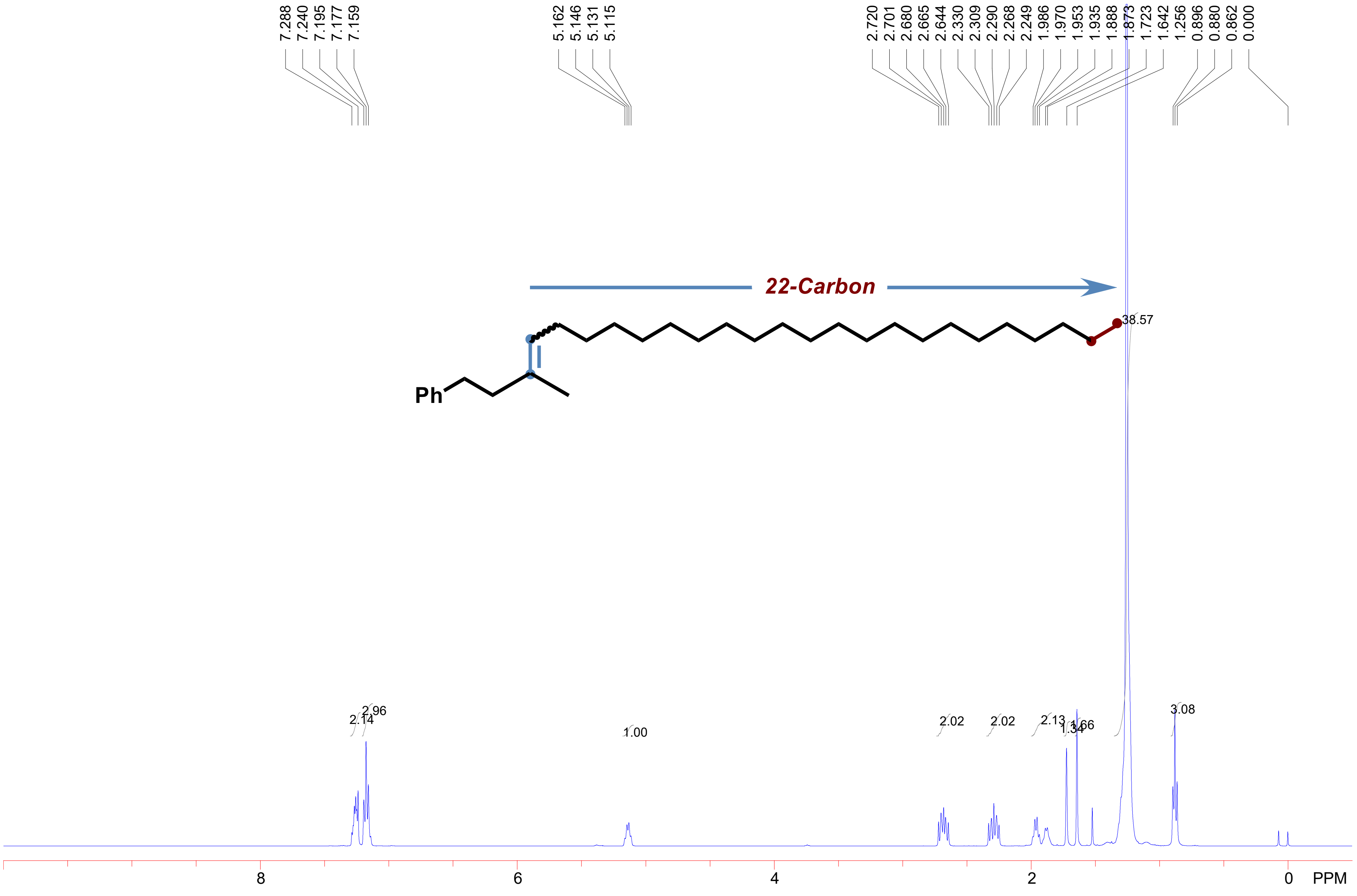

$^{13}$C NMR-spectrum (100 MHz, $CDCl_3$) of 9a

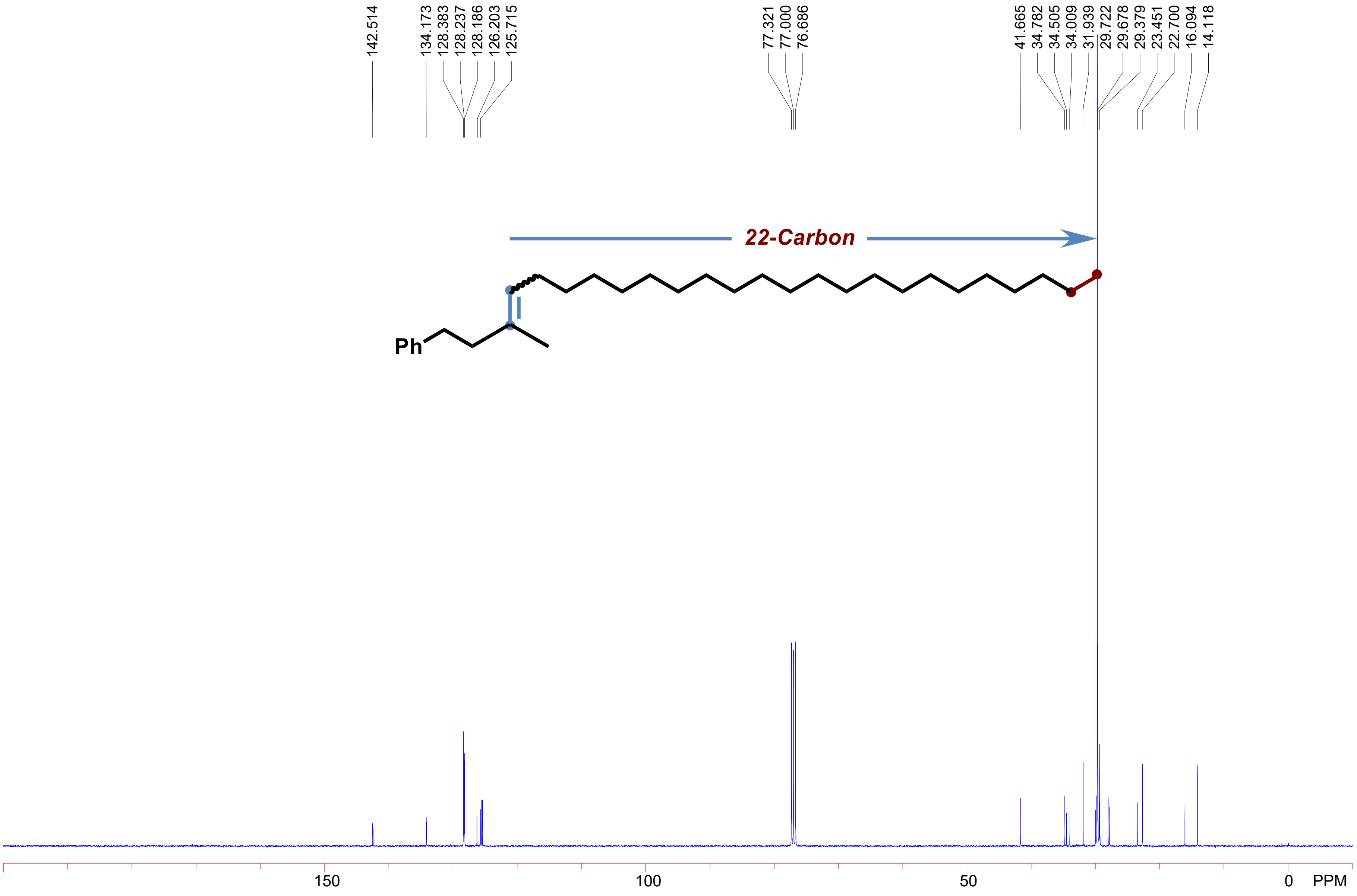

$^{1}$H NMR-spectrum (400 MHz, $CDCl_3$) of 10a

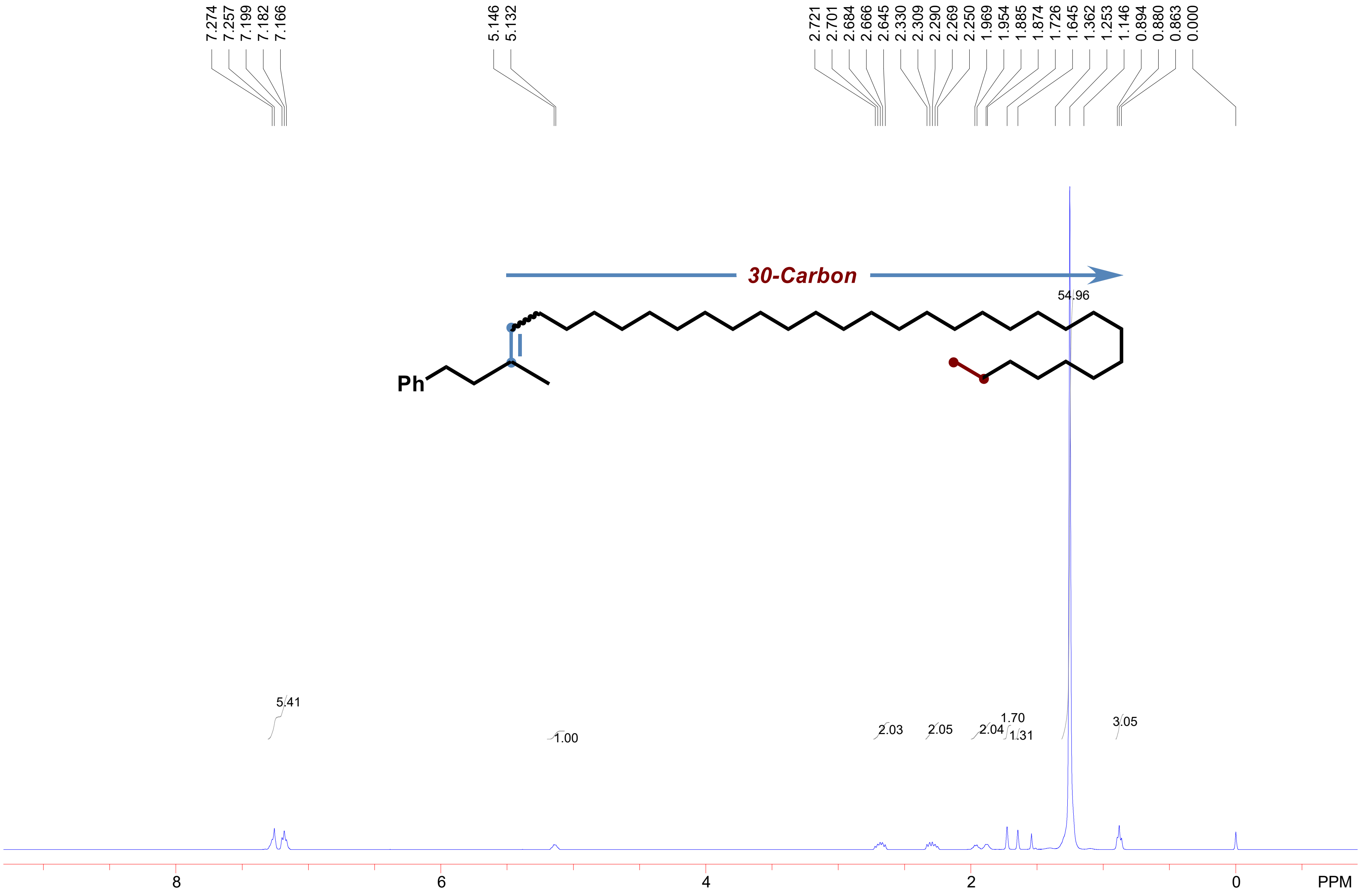

$^{13}C$ NMR-spectrum (100 MHz, $CDCl_3$) of 10a

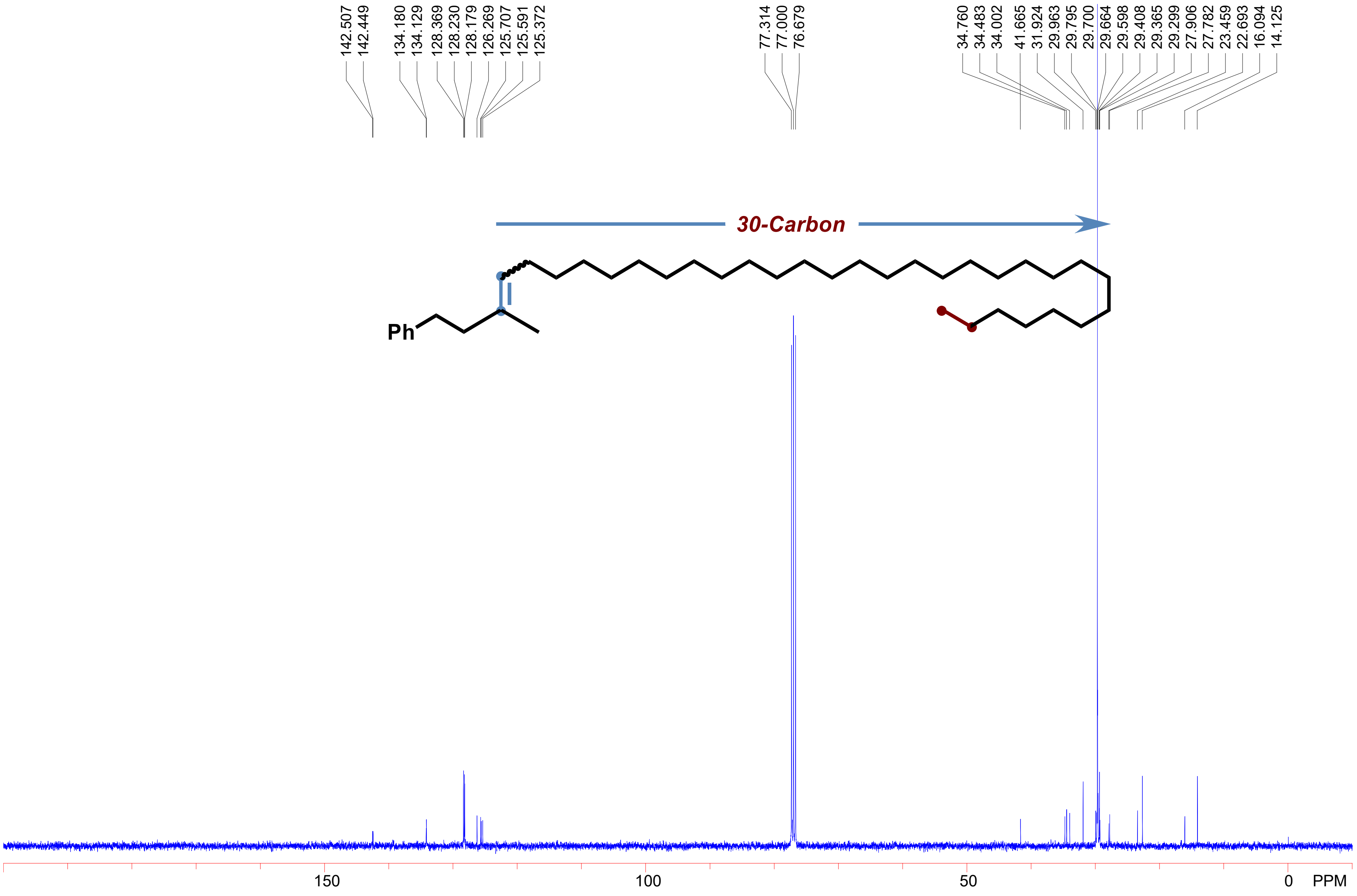

**$^{1}$H NMR-spectrum (400 MHz, CDCl3) of 11a**

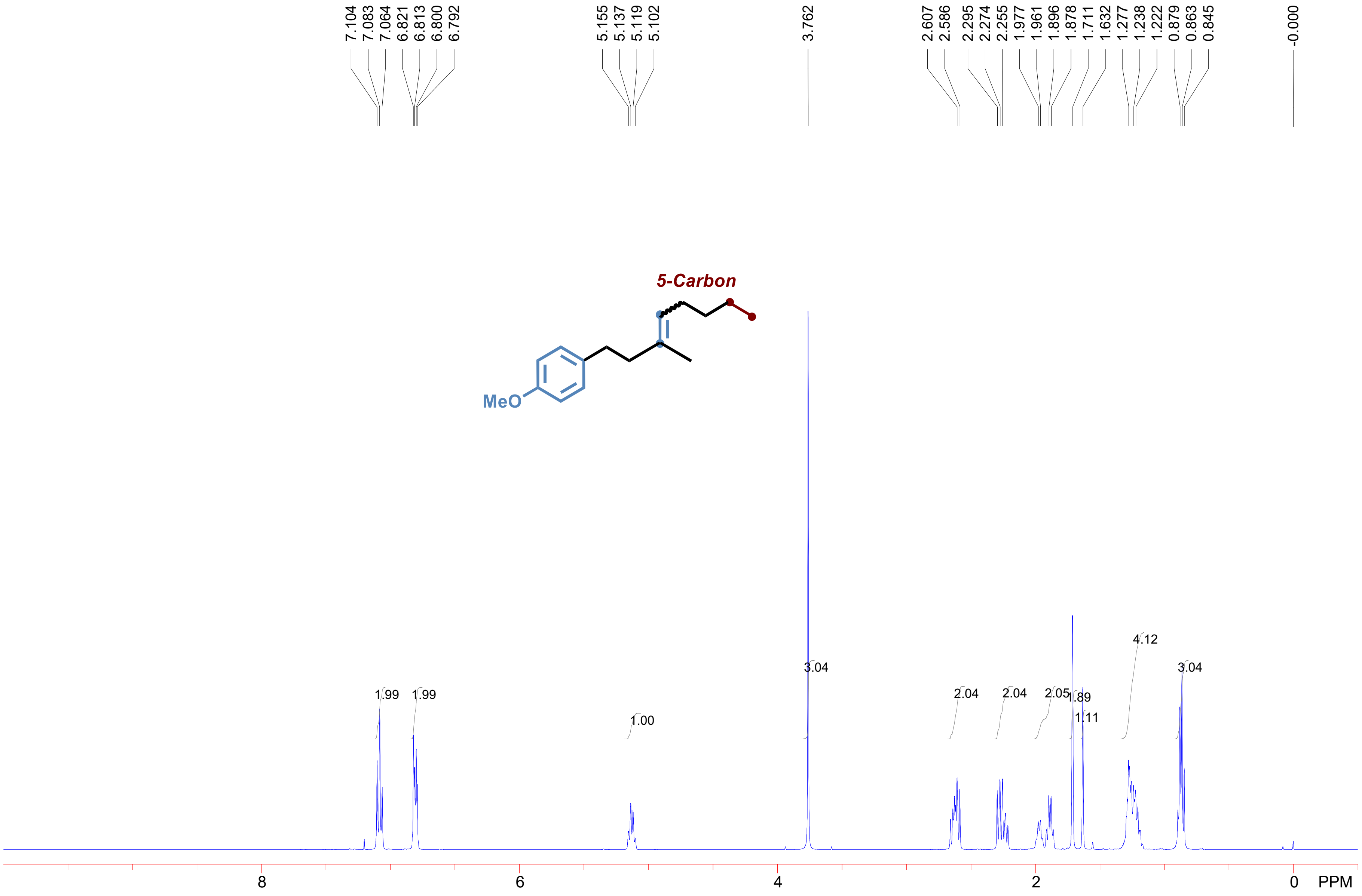

$^{13}$C NMR-spectrum (100 MHz, CDCl$_3$) of 11a

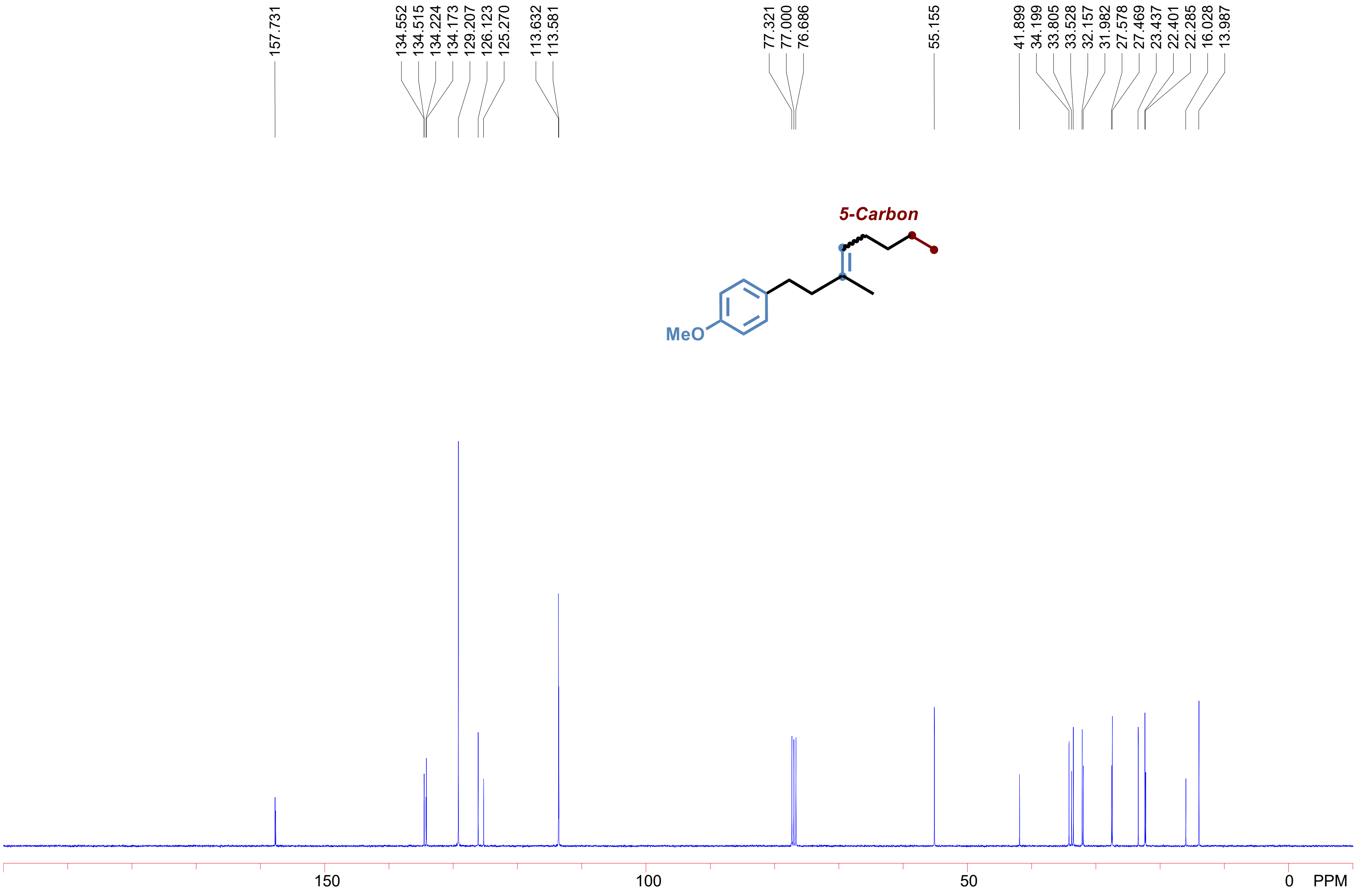

# $^{1}$H NMR-spectrum (400 MHz, $CDCl_3$) of 12a

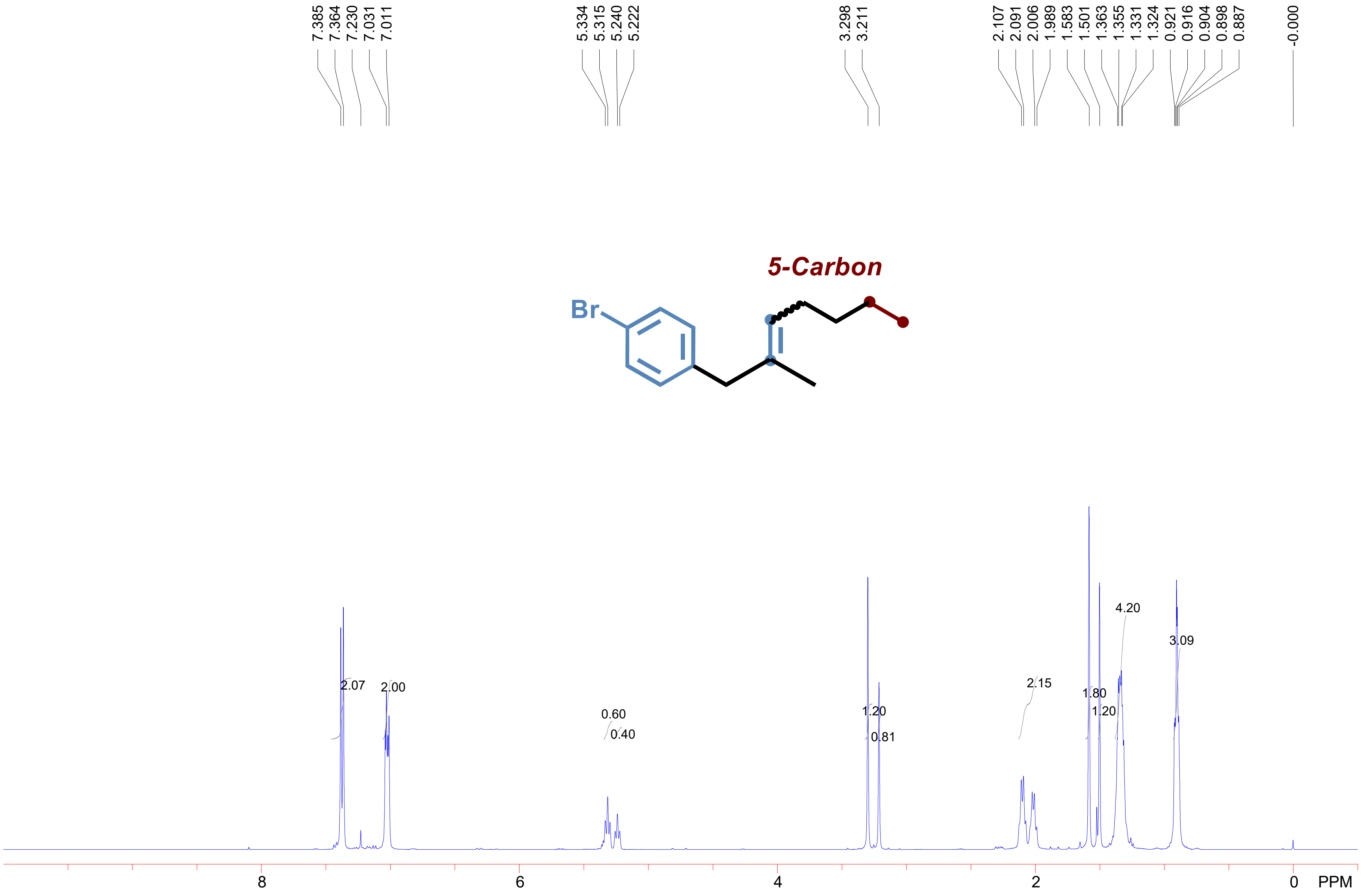

$^{13}$C NMR-spectrum (100 MHz, CDCl3) of 12a

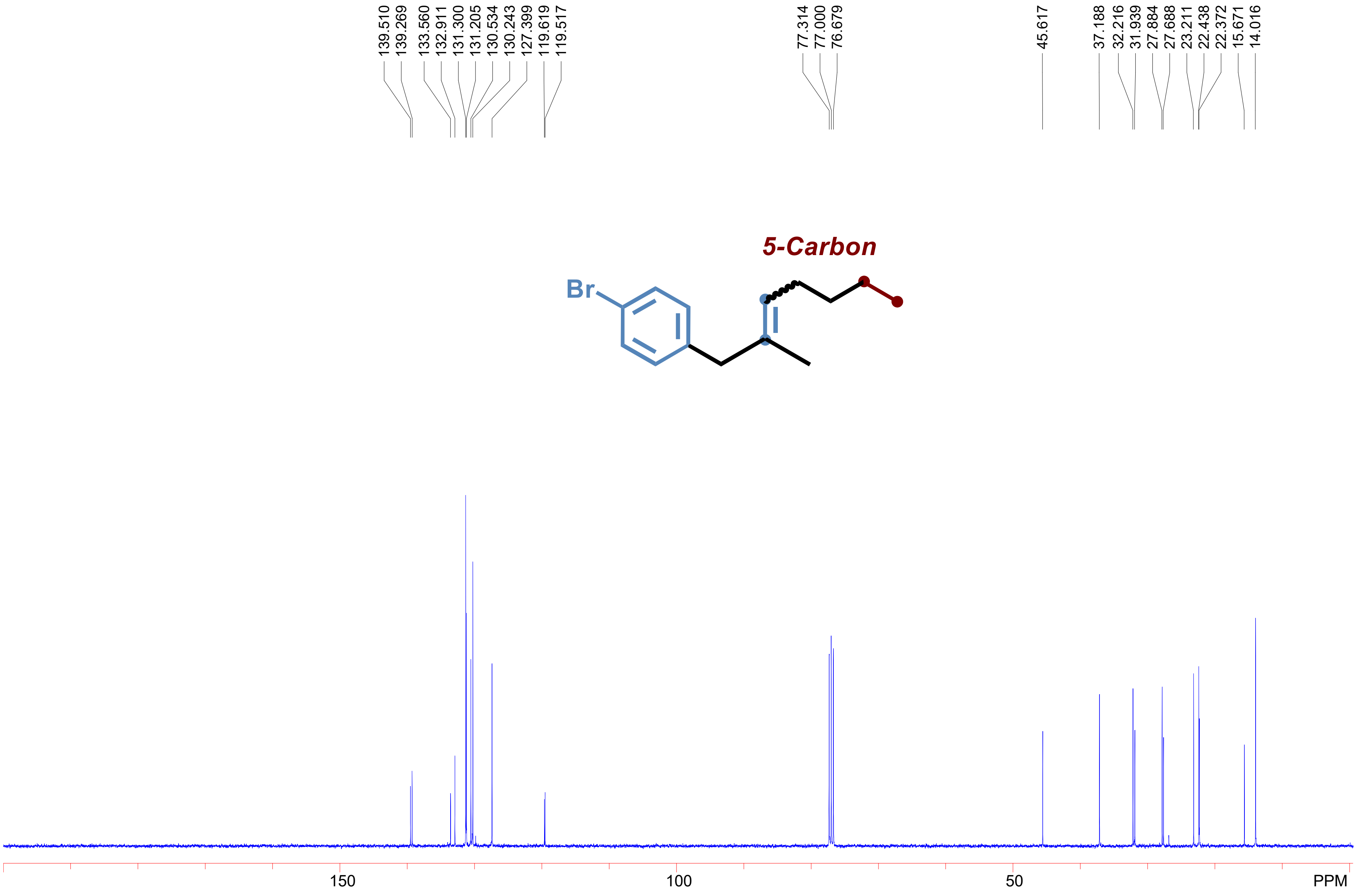

**$^{1}$H NMR-spectrum (400 MHz, $CDCl_3$) of 13a**

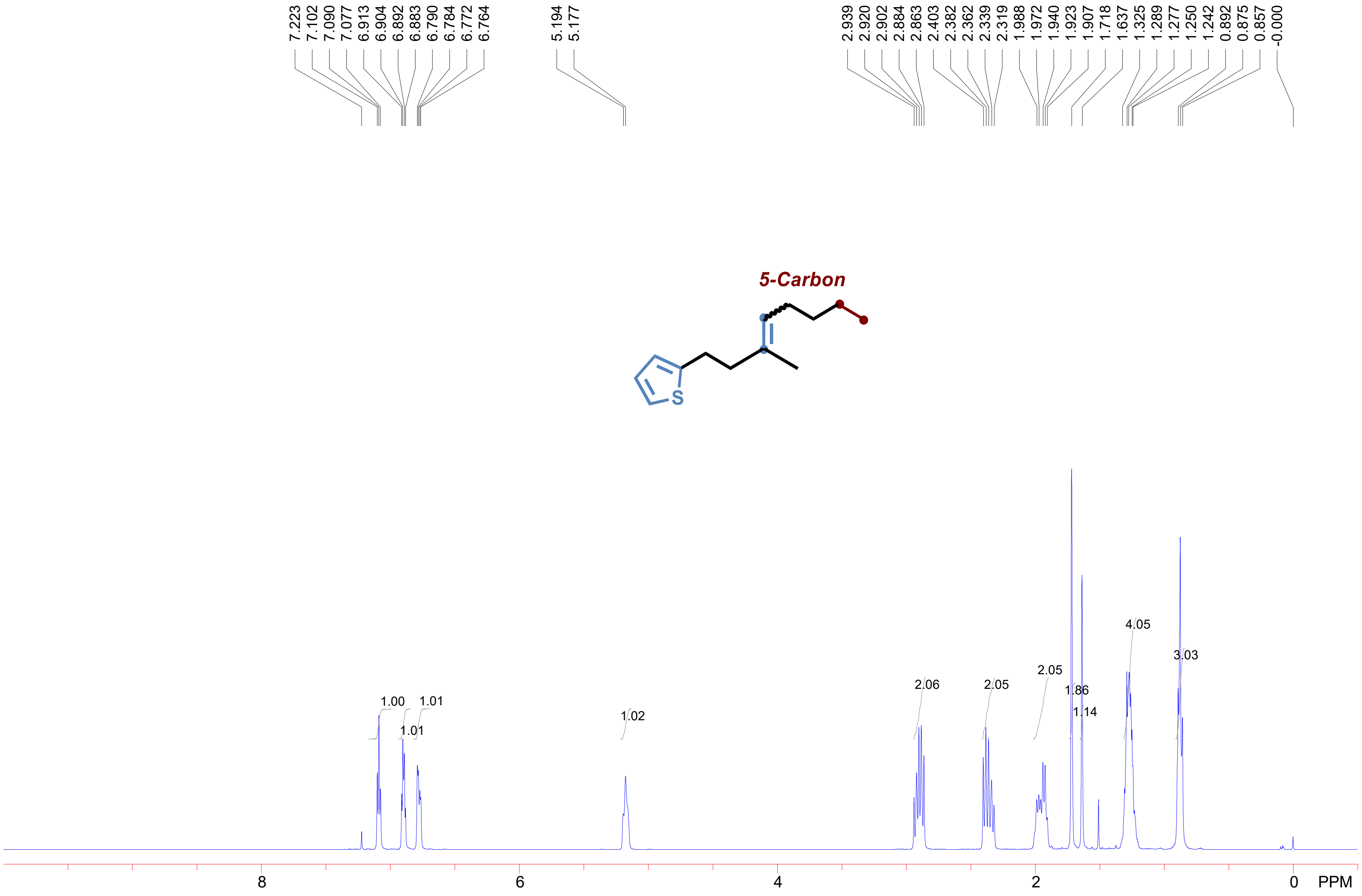

**$^{13}$C NMR-spectrum (100 MHz, $CDCl_3$) of 13a**

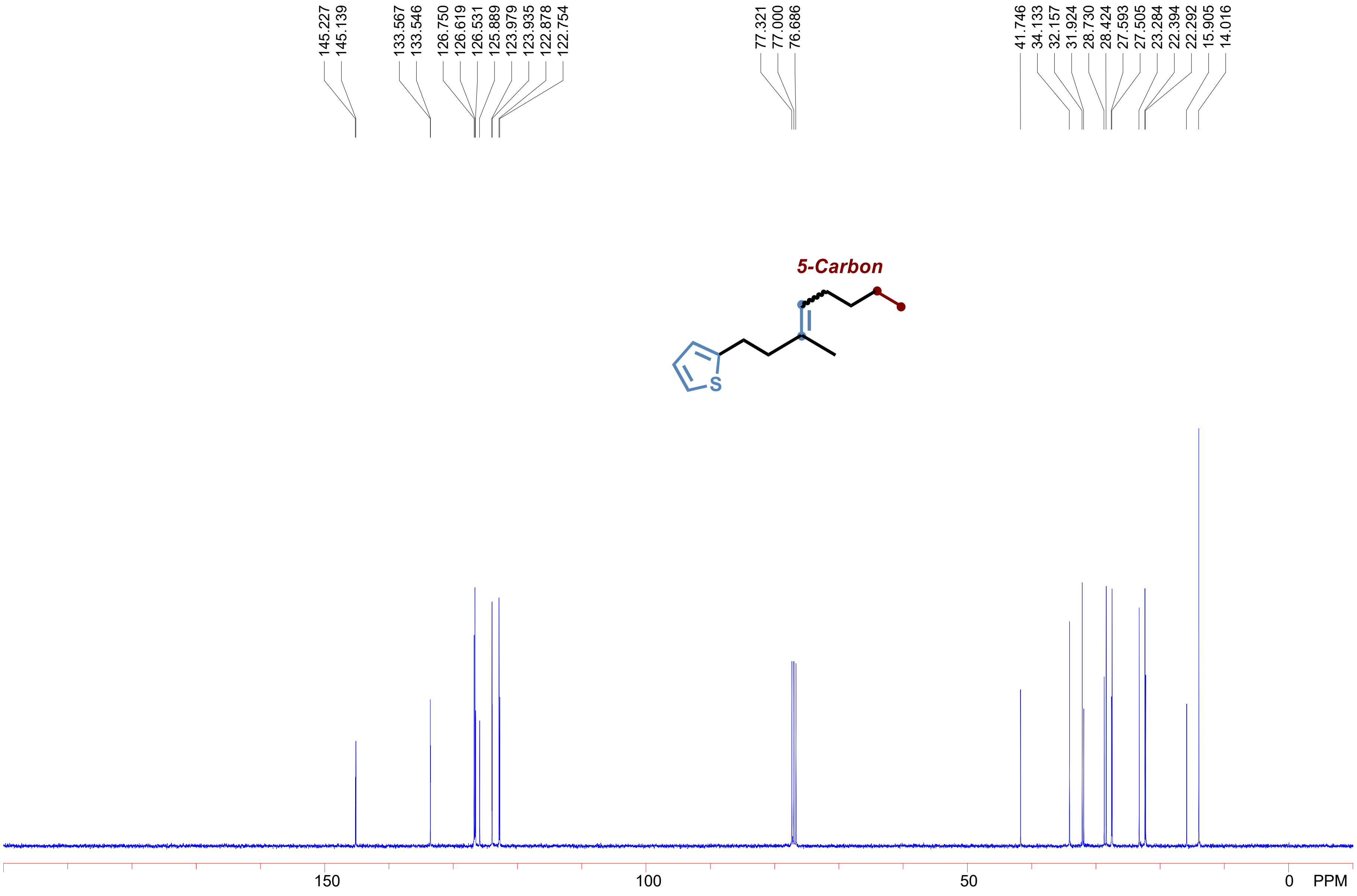

## $^{1}$H NMR-spectrum (400 MHz, $CDCl_3$) of 14a

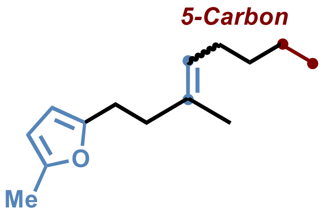


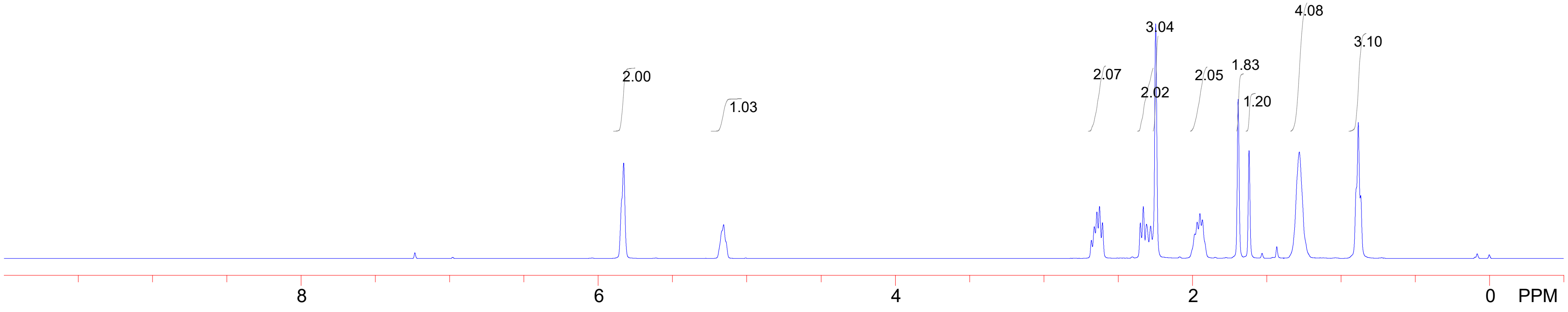

$^{13}$C NMR-spectrum (100 MHz, $CDCl_3$) of 14a

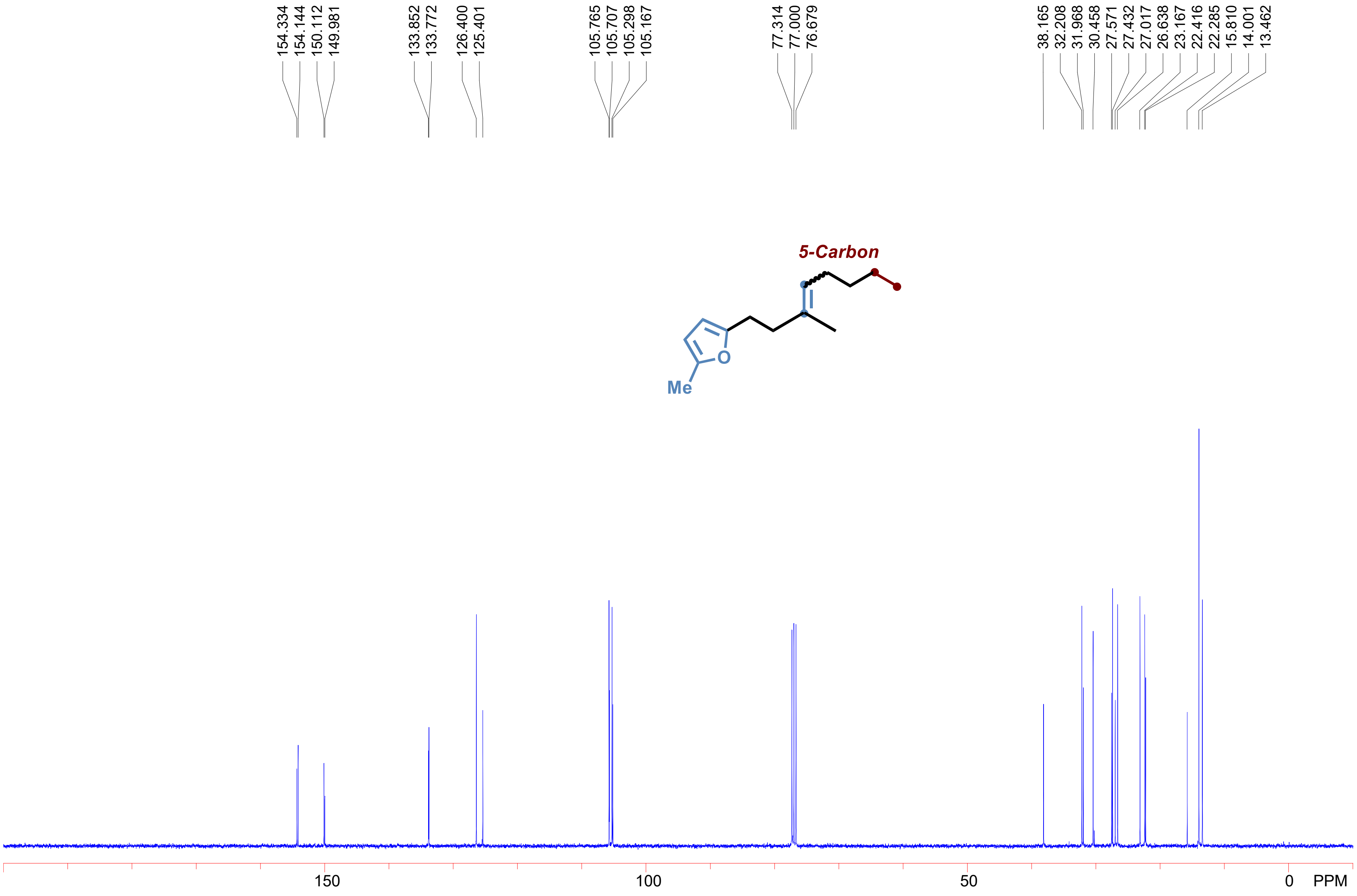

**$^{1}$H NMR-spectrum (400 MHz, CDCl3) of 15a**

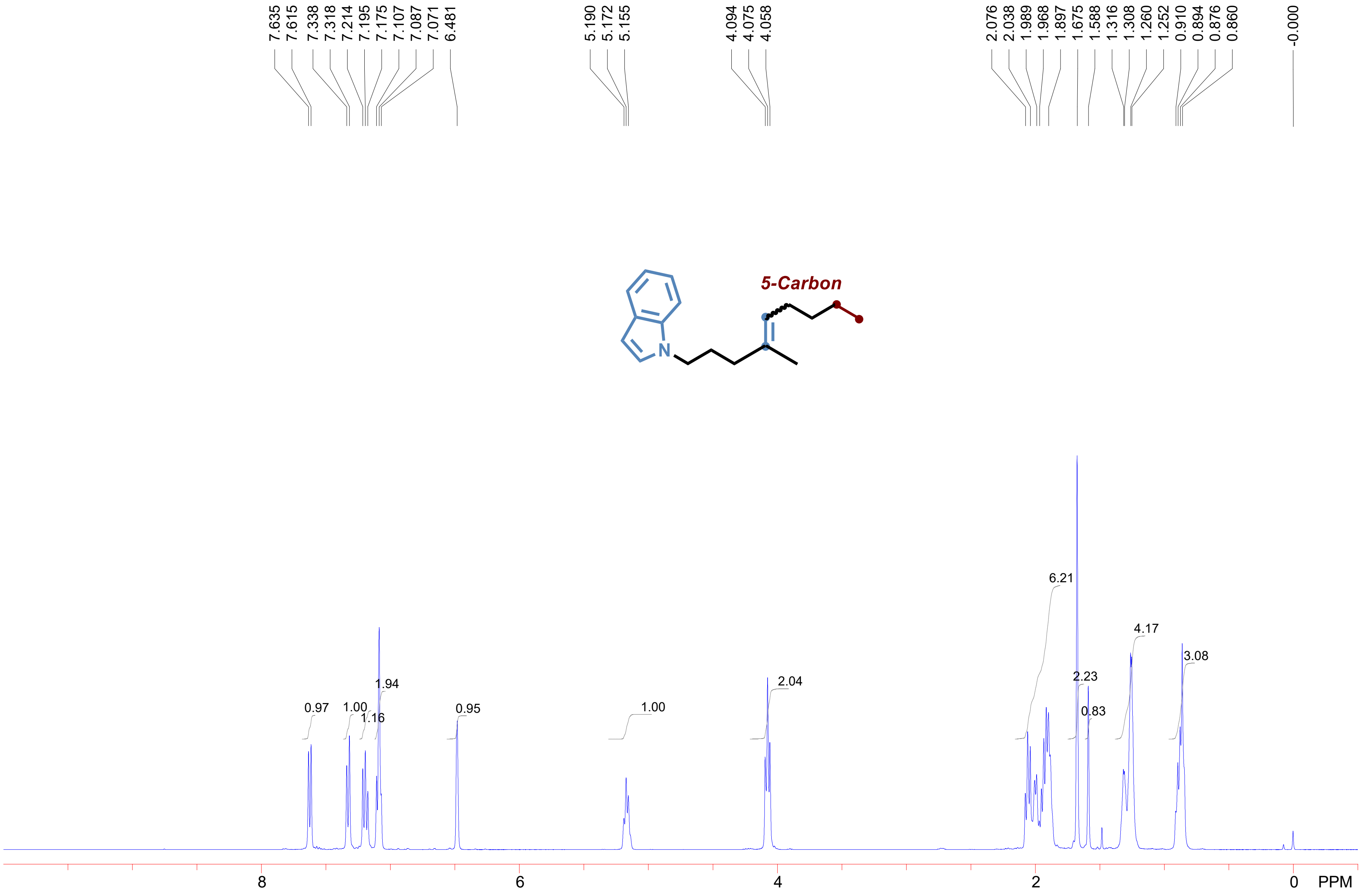

**$^{13}$C NMR-spectrum (100 MHz, $CDCl_3$) of 15a**

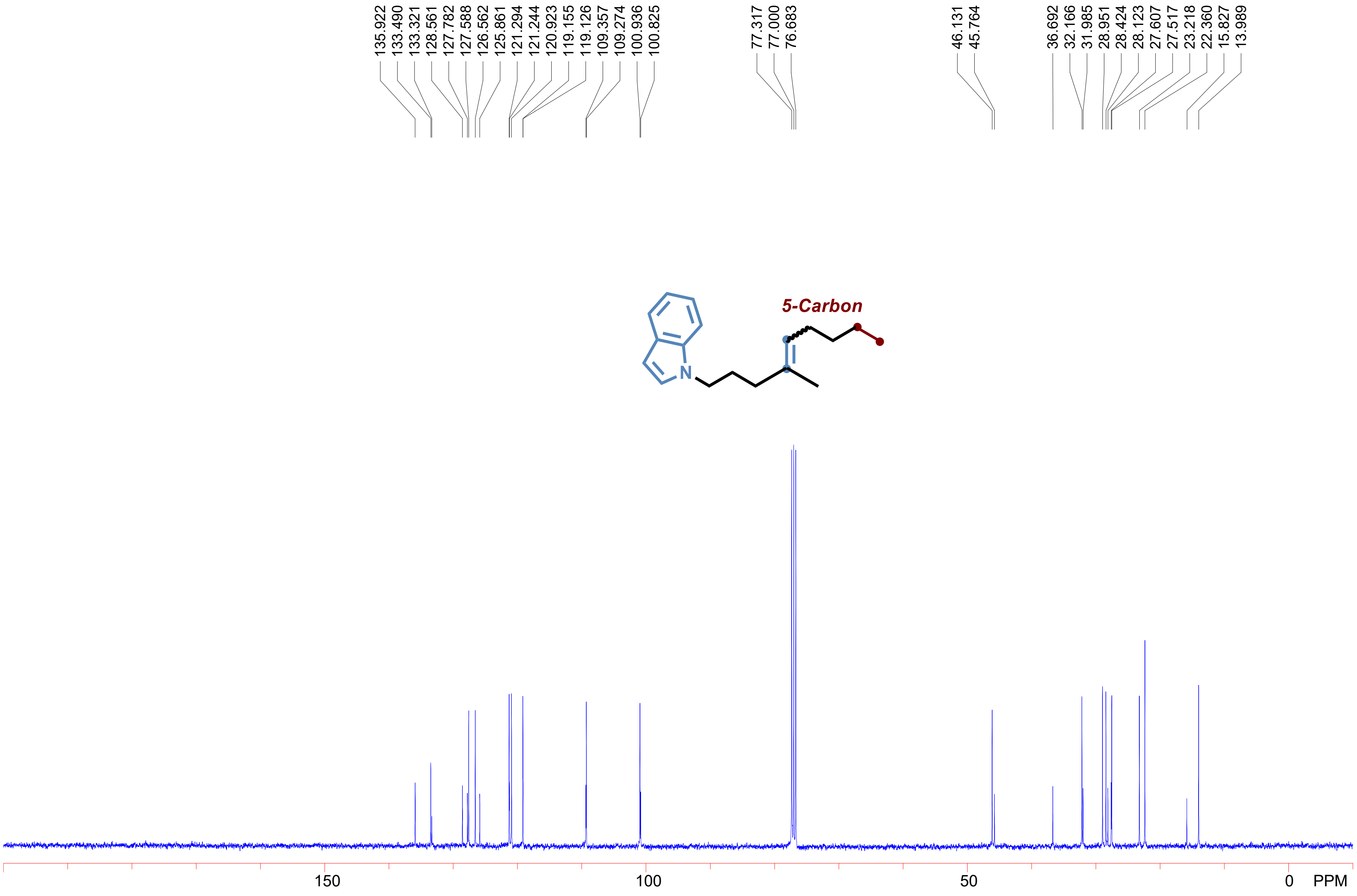

$^{1}$H NMR-spectrum (400 MHz, CDCl3) of 16a

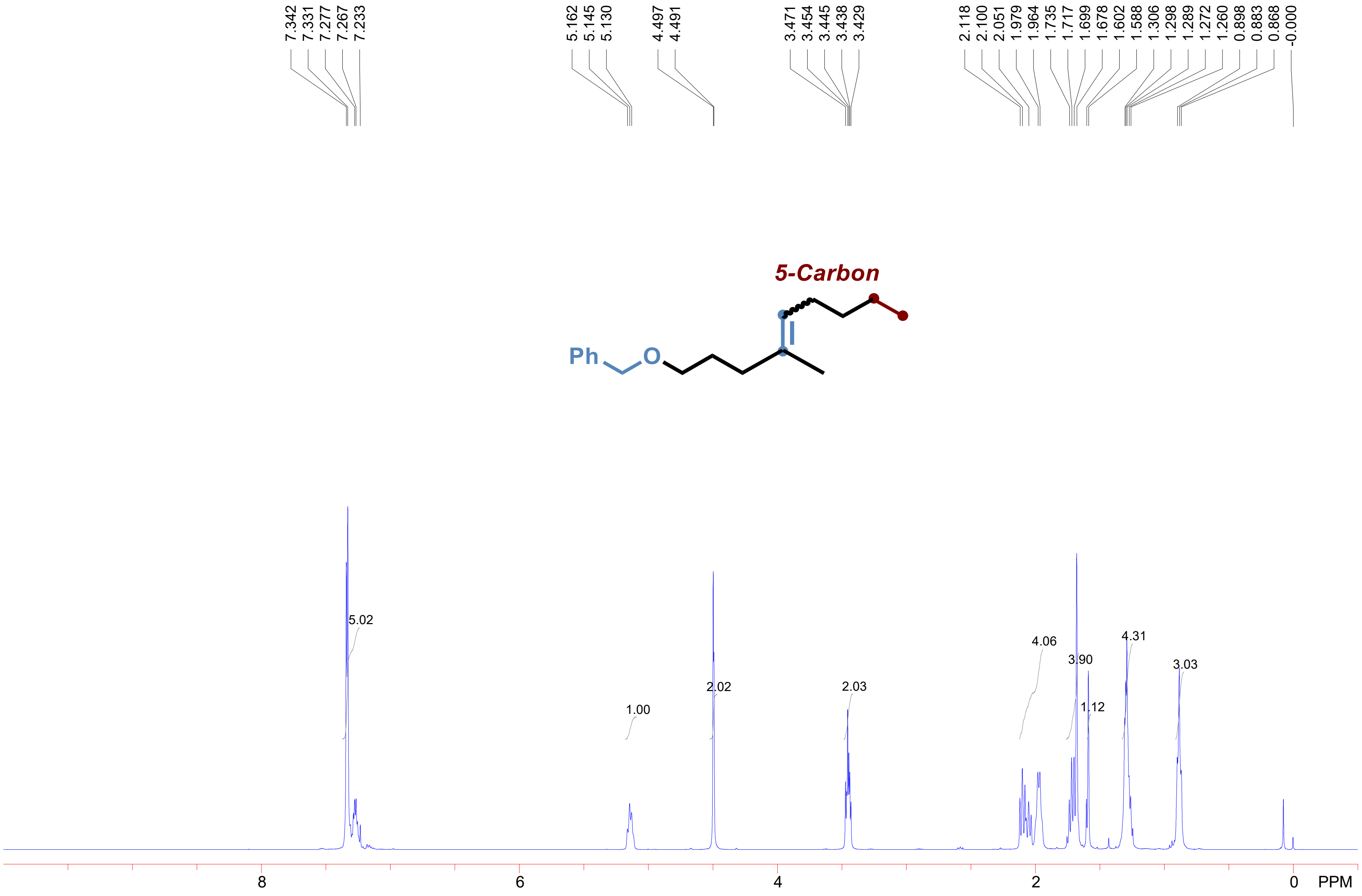

$^{13}$C NMR-spectrum (100 MHz, $CDCl_3$) of 16a

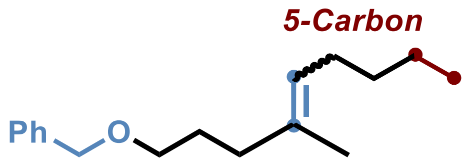


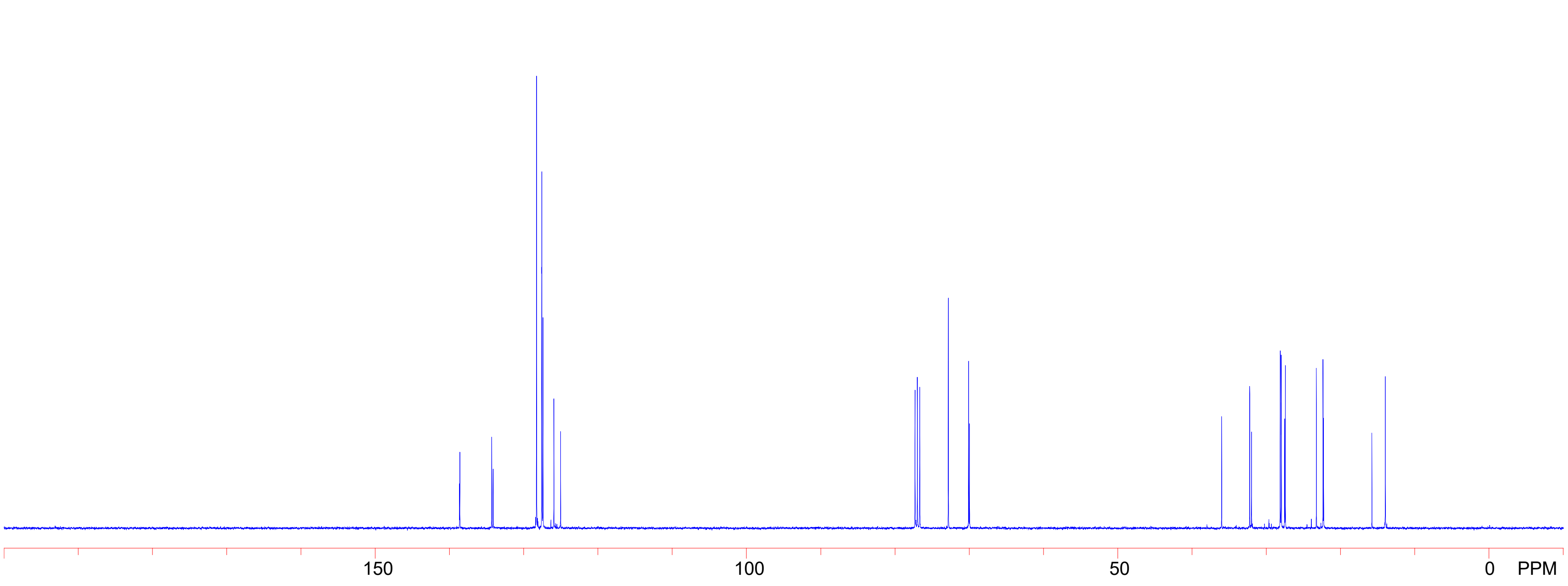

## $^{1}$H NMR-spectrum (400 MHz, $CDCl_3$) of 17a

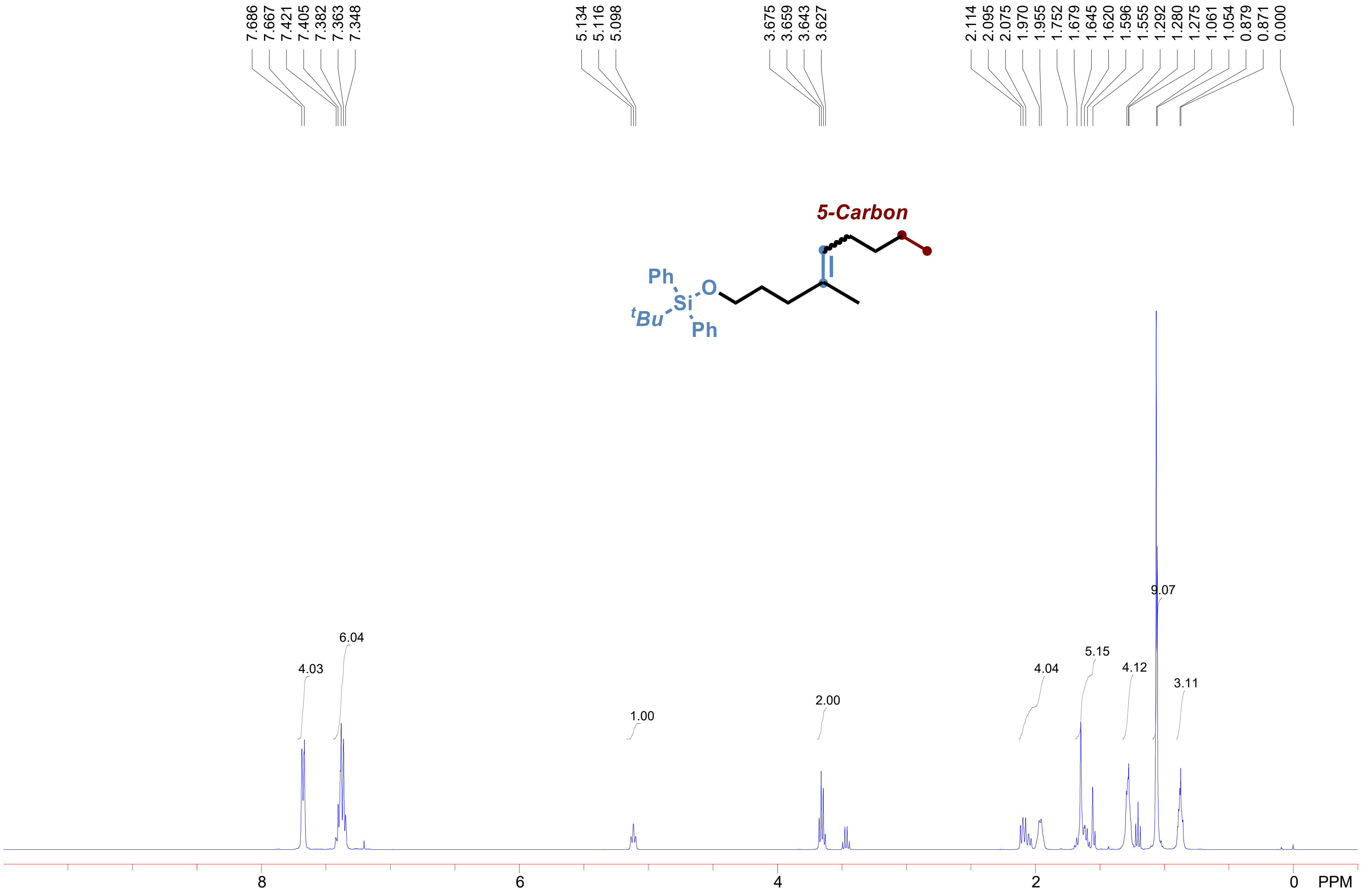

**$^{13}$C NMR-spectrum (100 MHz, CDCl$_3$) of 17a**

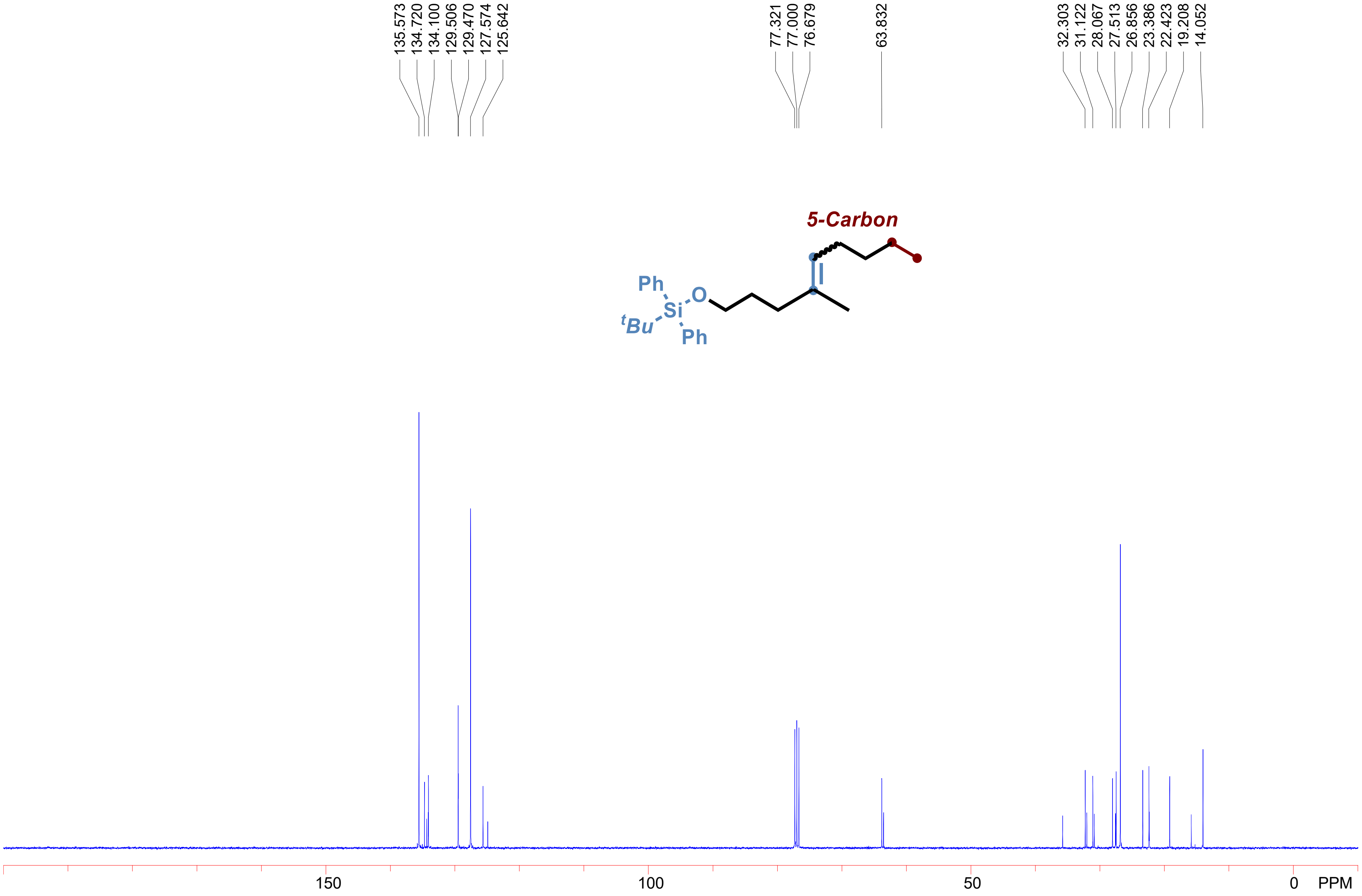

**$^{1}$H NMR-spectrum (400 MHz, $CDCl_3$) of 18a**

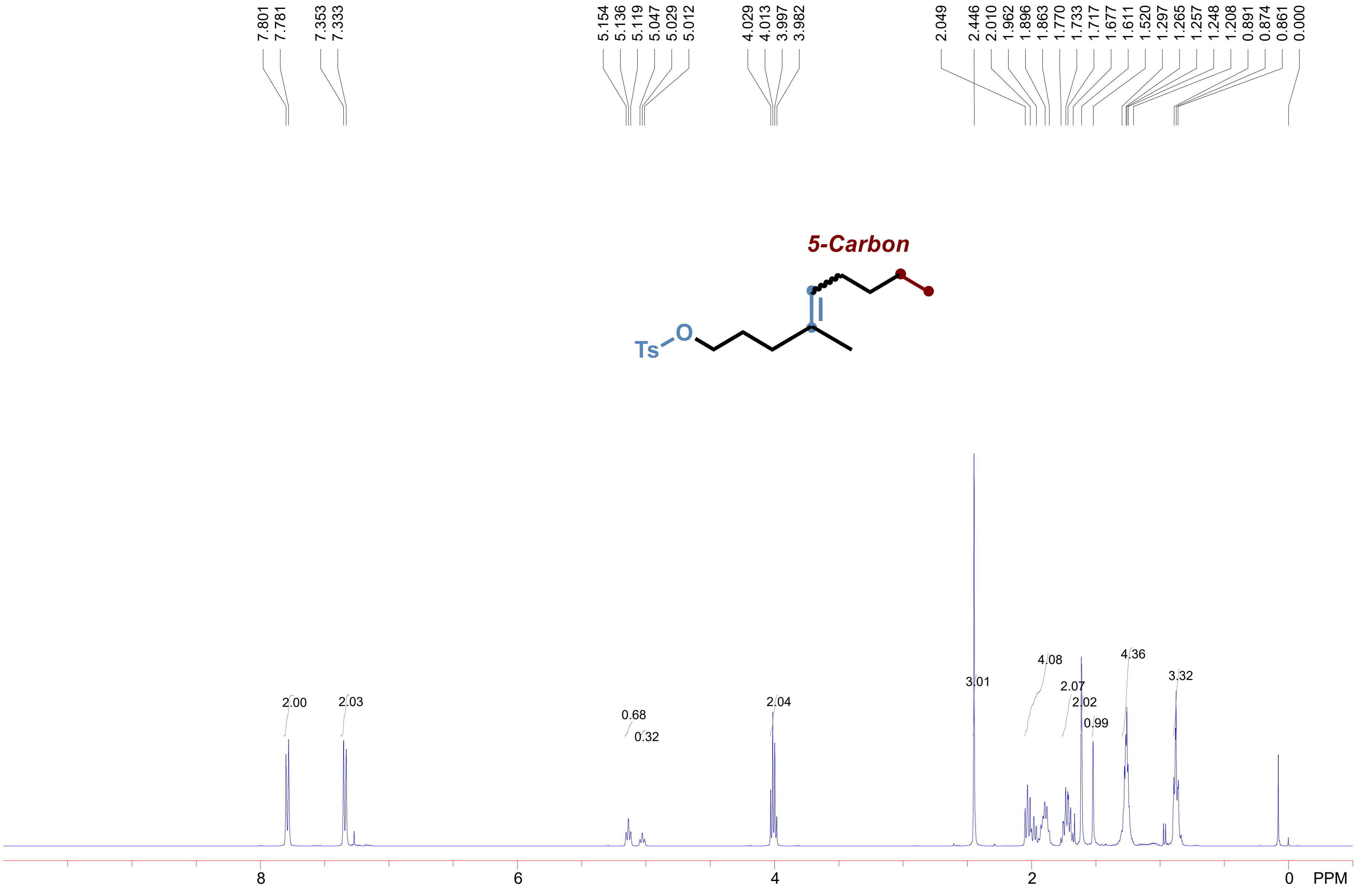

**$^{13}$C NMR-spectrum (100 MHz, $CDCl_3$) of 18a**

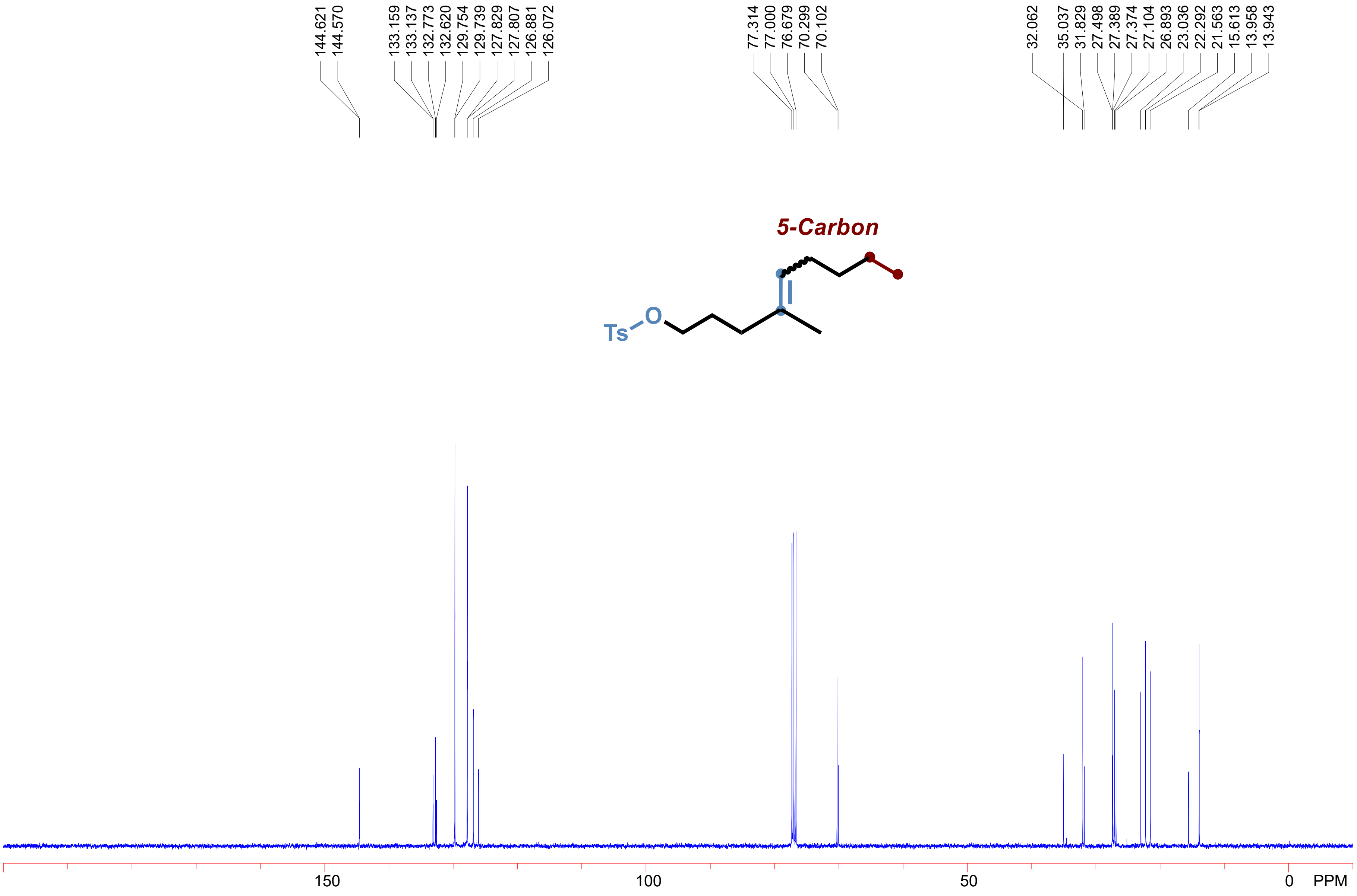

**$^{1}$H NMR-spectrum (400 MHz, CDCl3) of 19a**

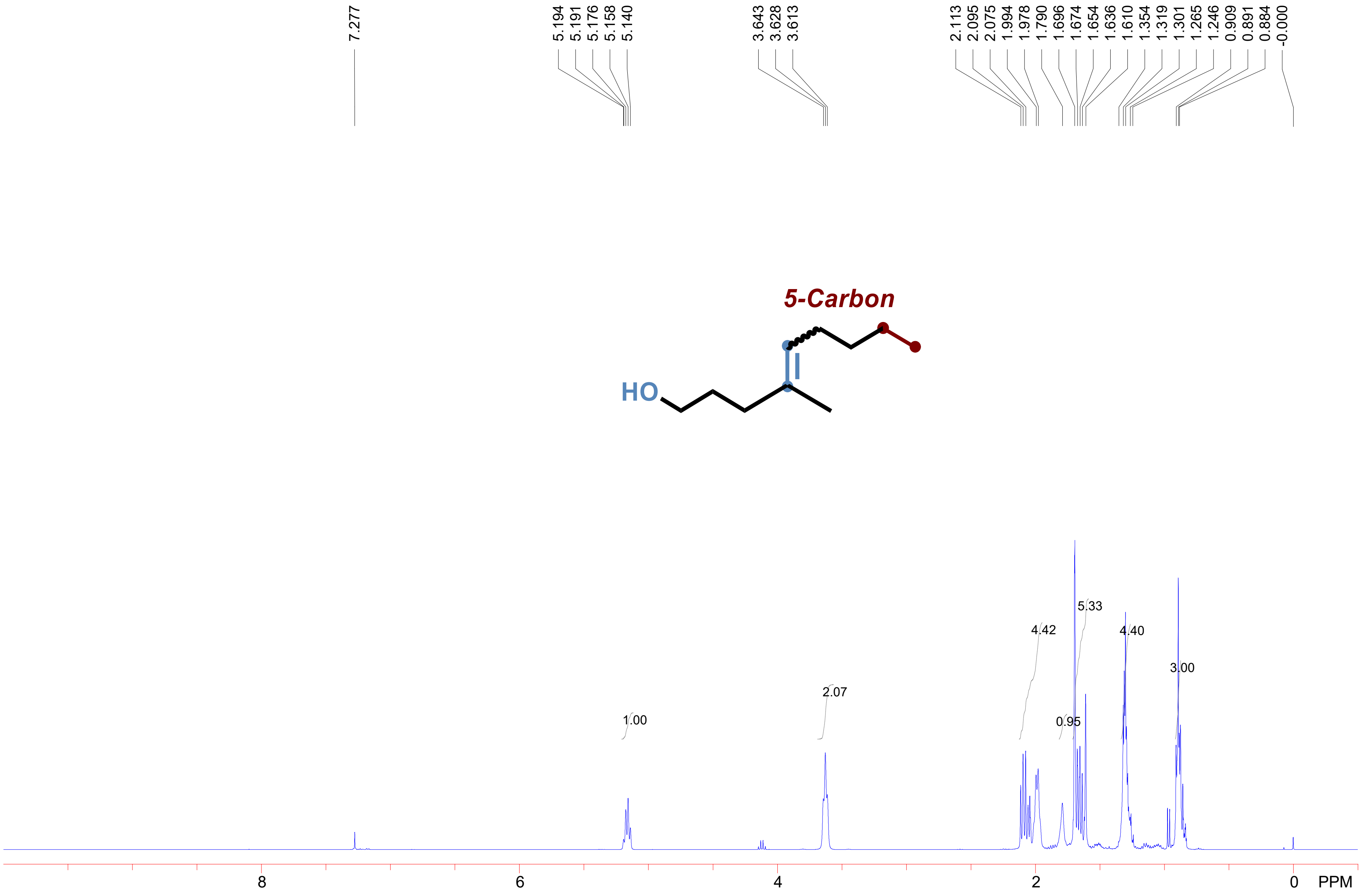

$^{13}C$ NMR-spectrum (100 MHz, $CDCl_3$) of 19a

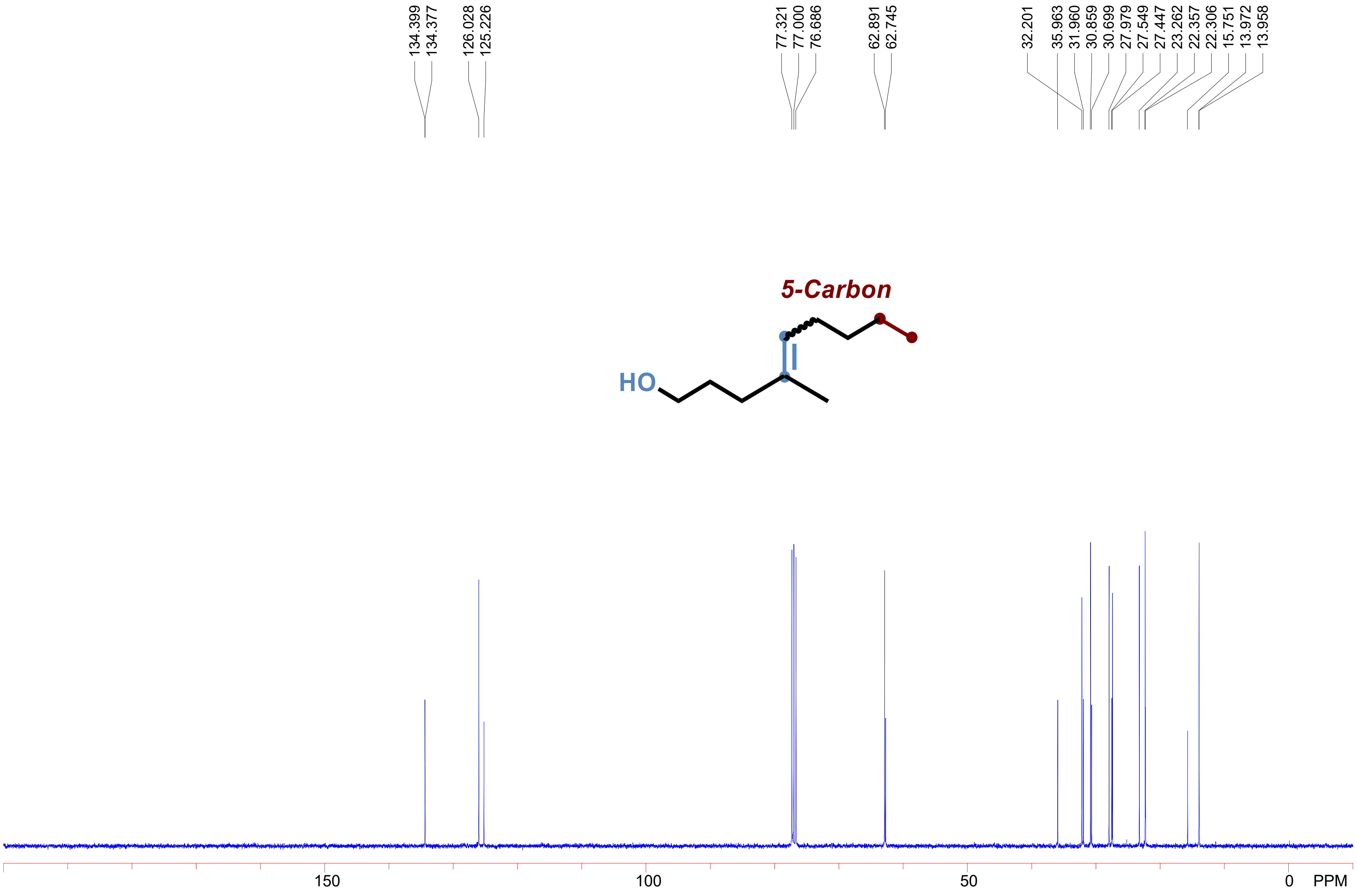

$^{1}$H NMR-spectrum (400 MHz, $CDCl_3$) of 20a

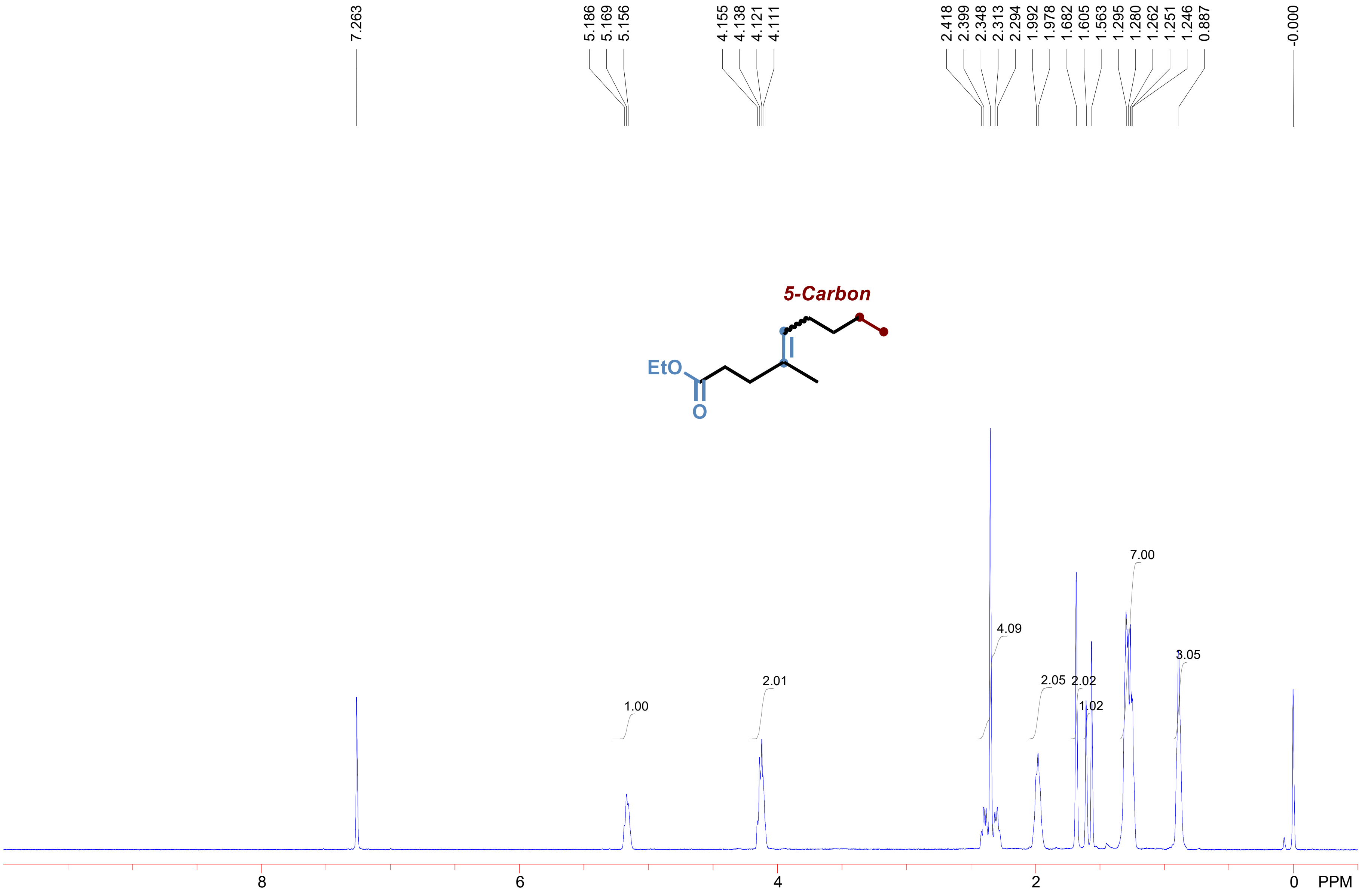

$^{13}C$ NMR-spectrum (100 MHz, $CDCl_3$) of 20a

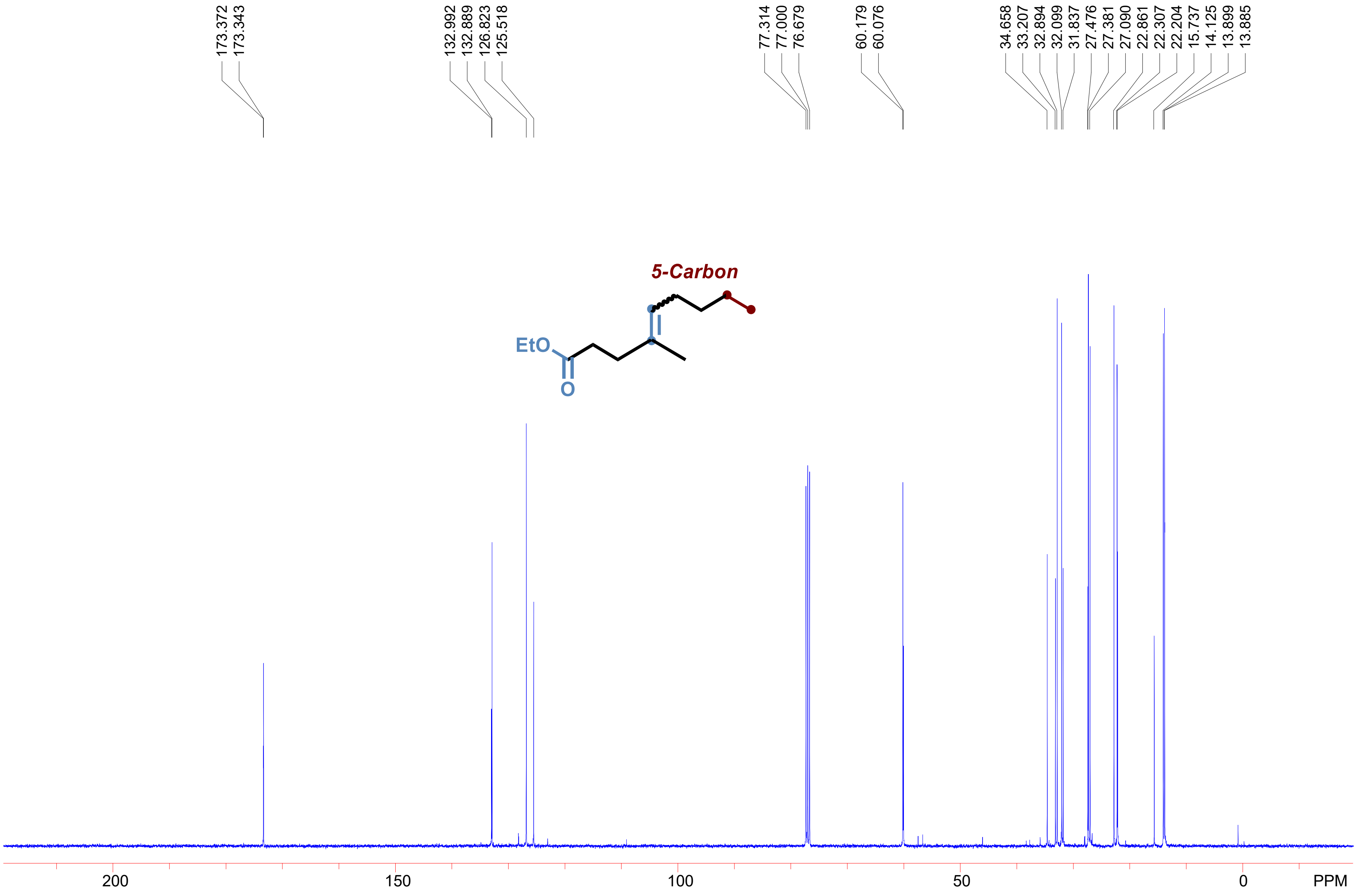

**$^{1}$H NMR-spectrum (400 MHz, CDCl3) of 21a**

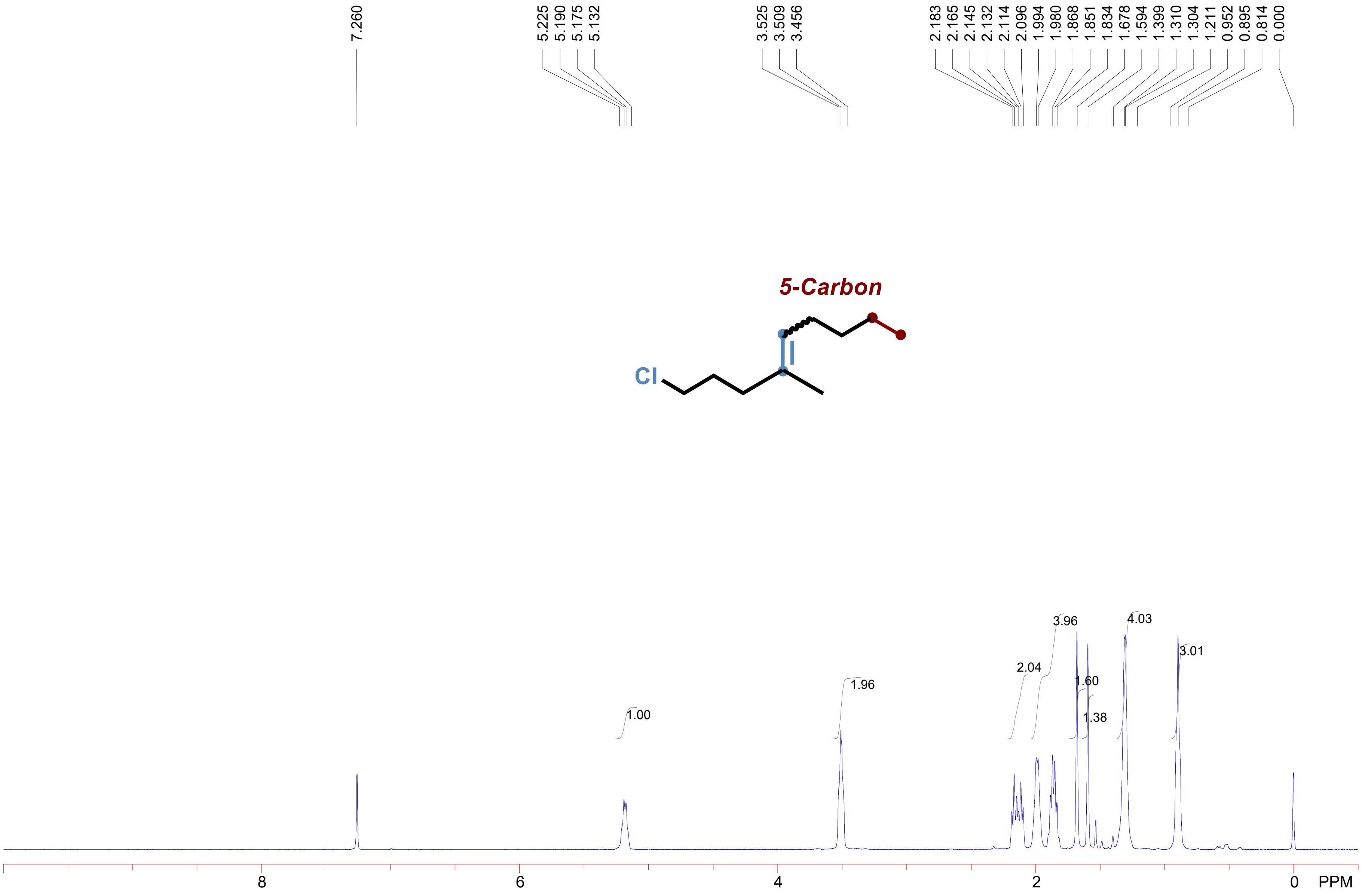

**$^{13}$C NMR-spectrum (100 MHz, $CDCl_3$) of 21a**

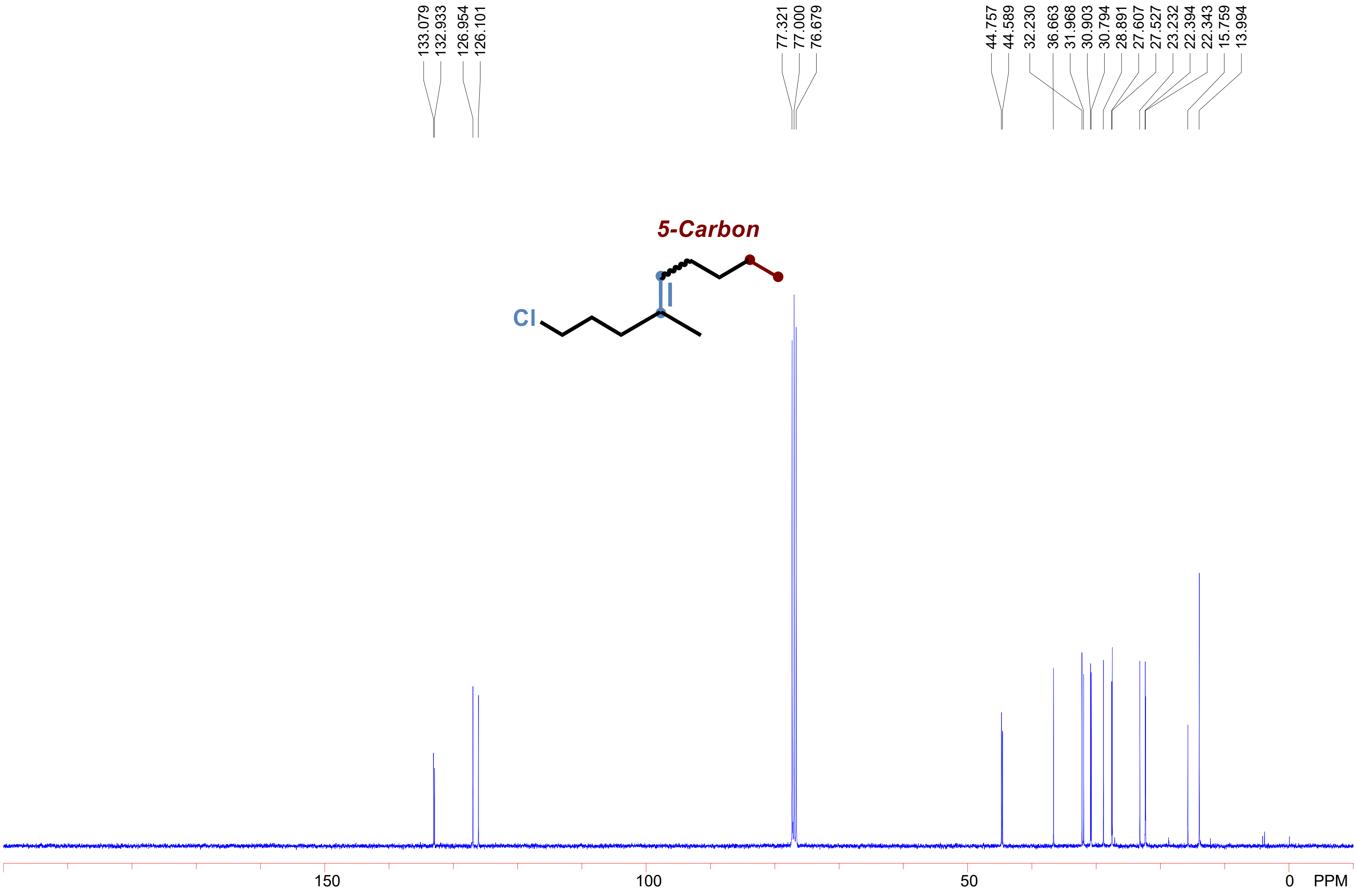

**$^{1}$H NMR-spectrum (400 MHz, CDCl3) of 22a**

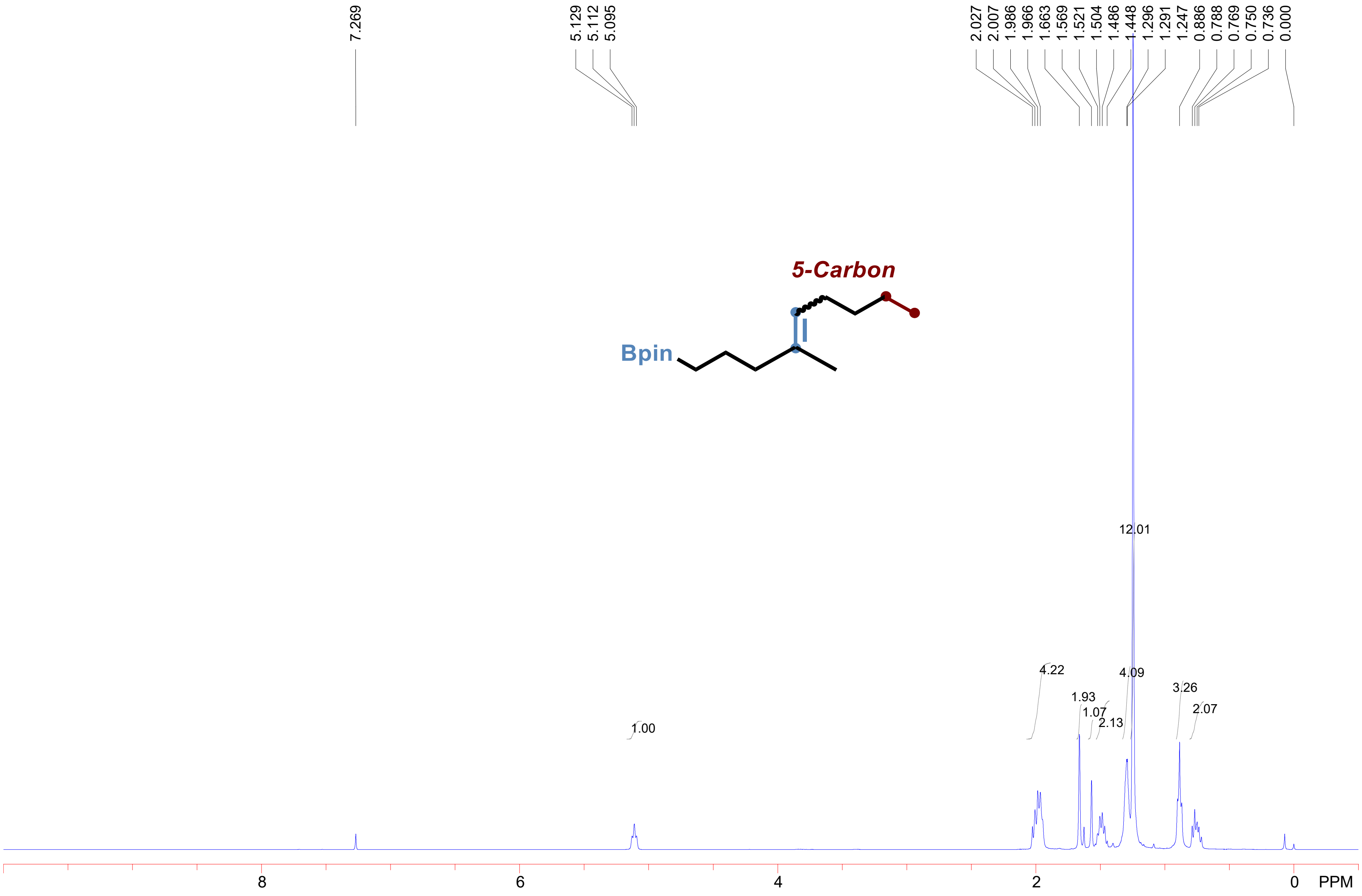

$^{13}$C NMR-spectrum (100 MHz, CDCl$_3$) of 22a

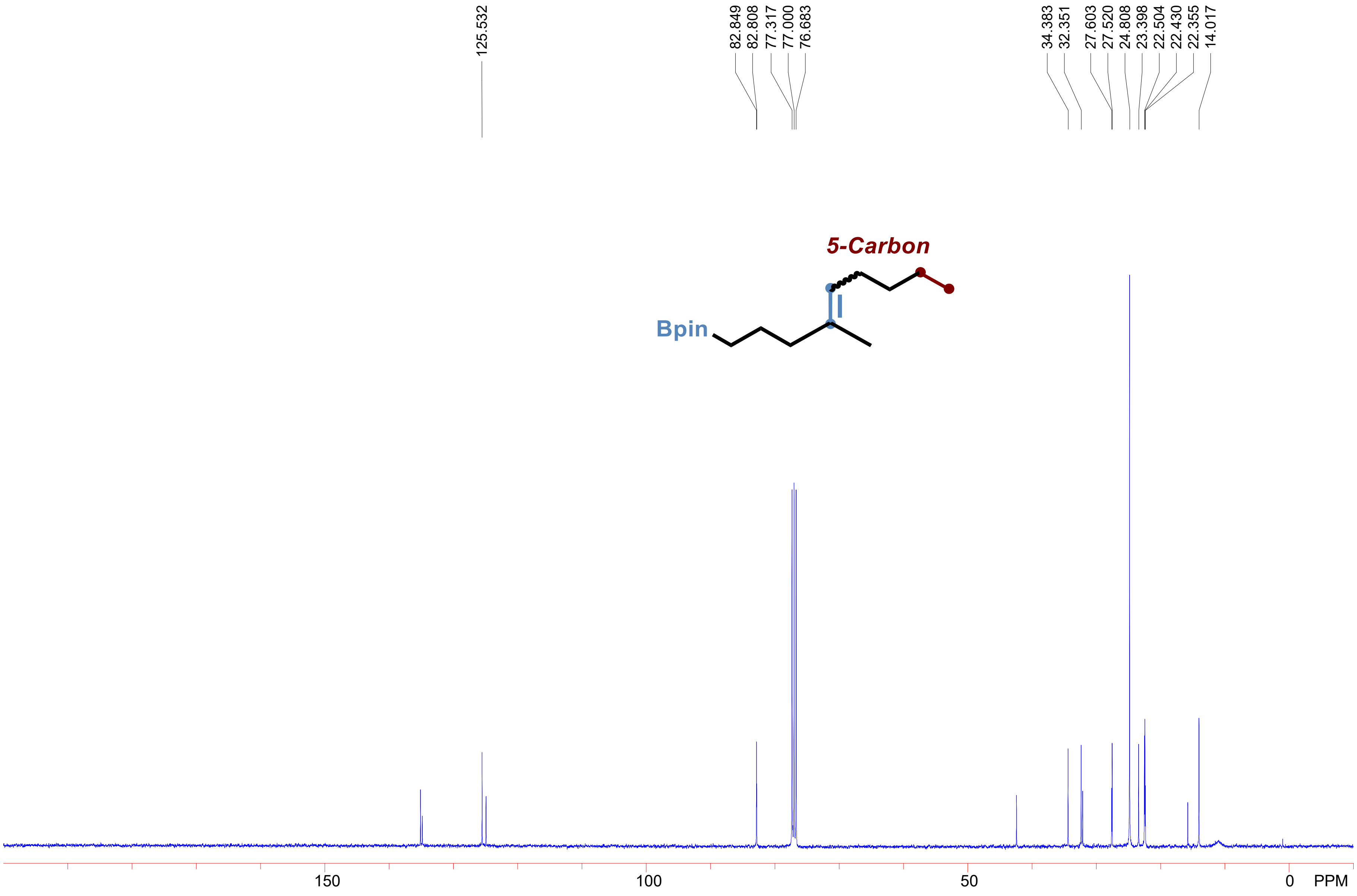

$^{1}$H NMR-spectrum (400 MHz, CDCl3) of 23a

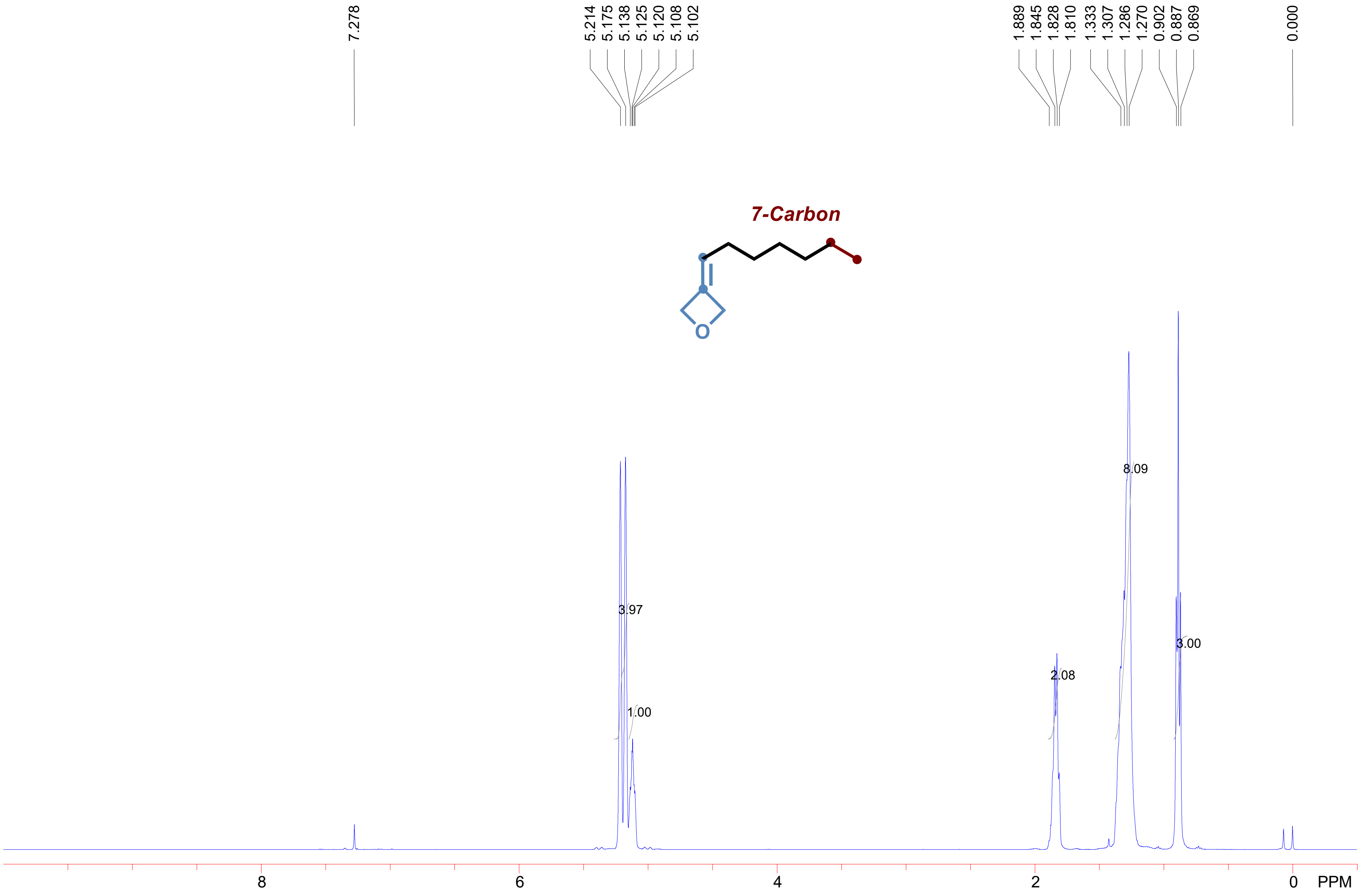

$^{13}$C NMR-spectrum (100 MHz, $CDCl_3$) of 23a

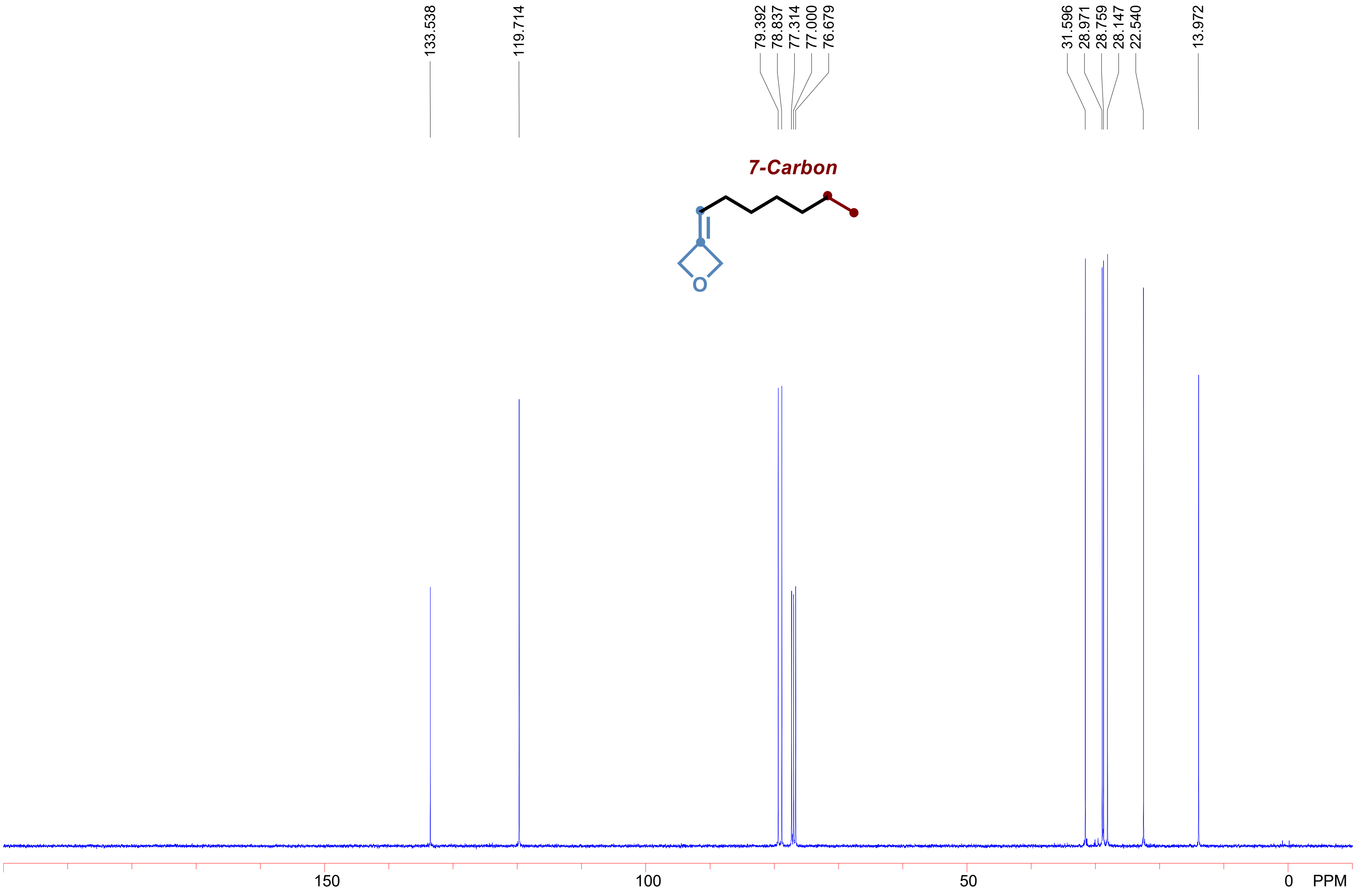

$^{1}$H NMR-spectrum (400 MHz, $CDCl_3$) of 24a

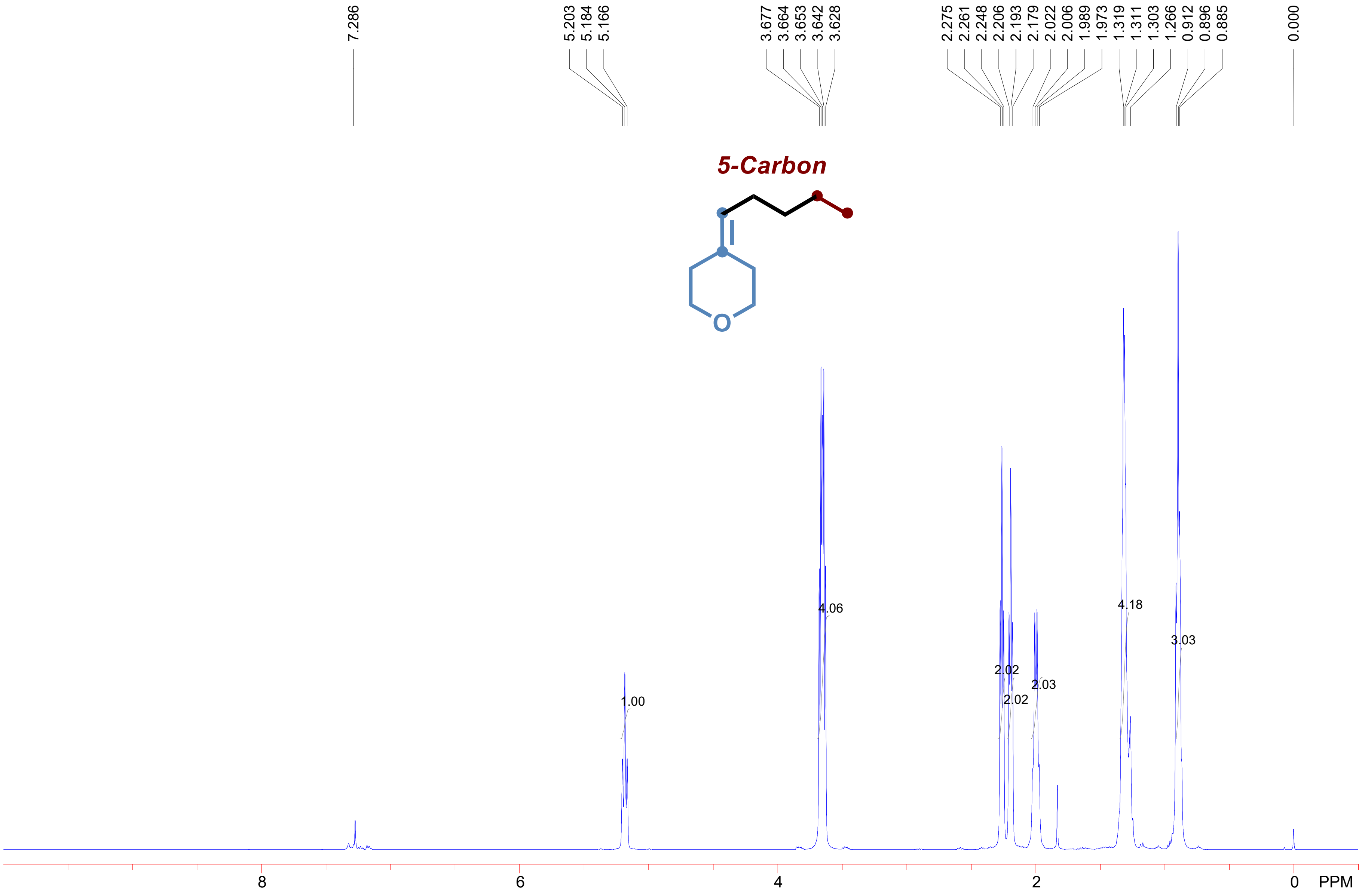

**$^{13}$C NMR-spectrum (100 MHz, CDCl$_3$) of 24**

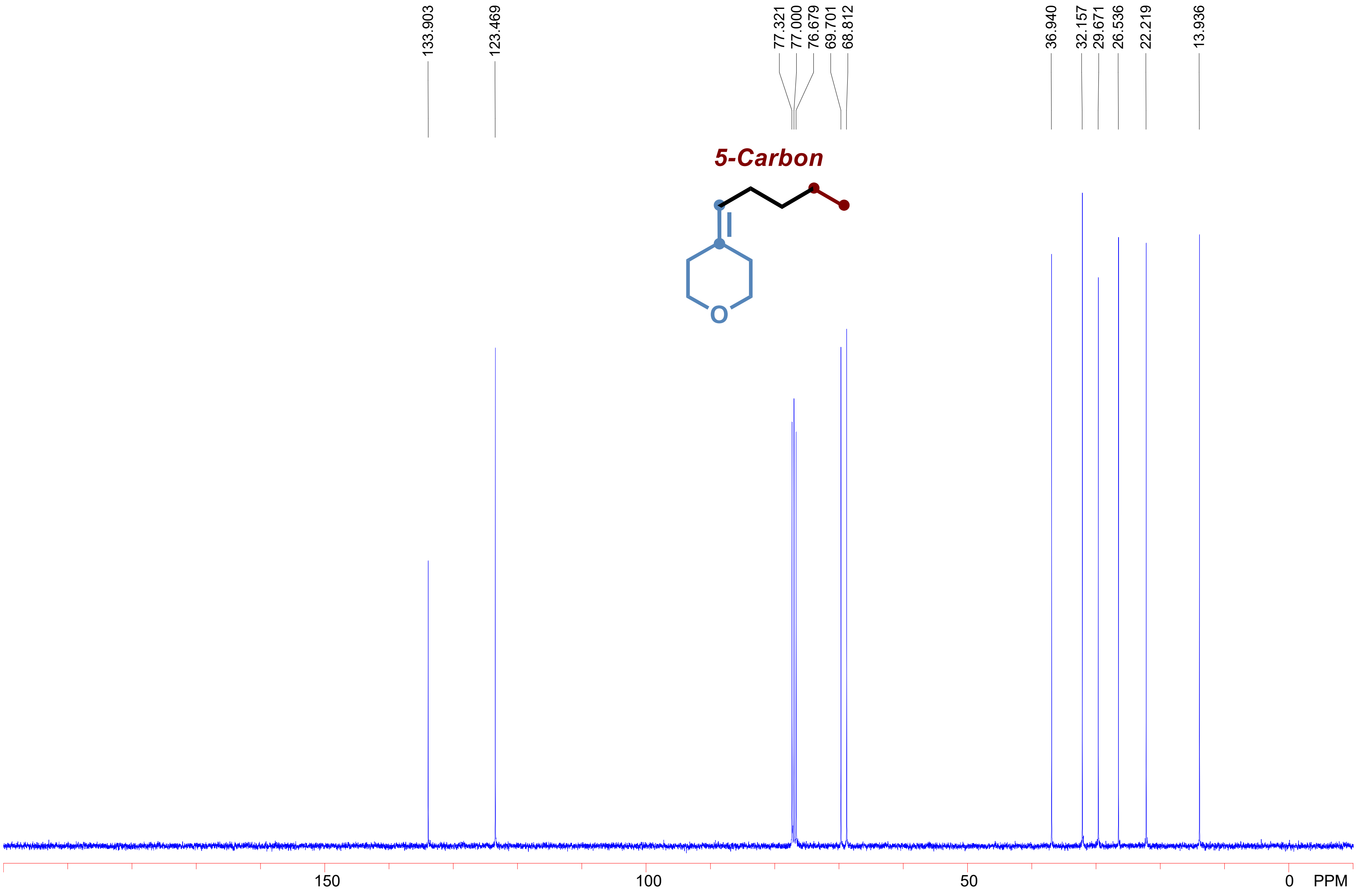

$^{1}$H NMR-spectrum (400 MHz, $CDCl_3$) of 25a

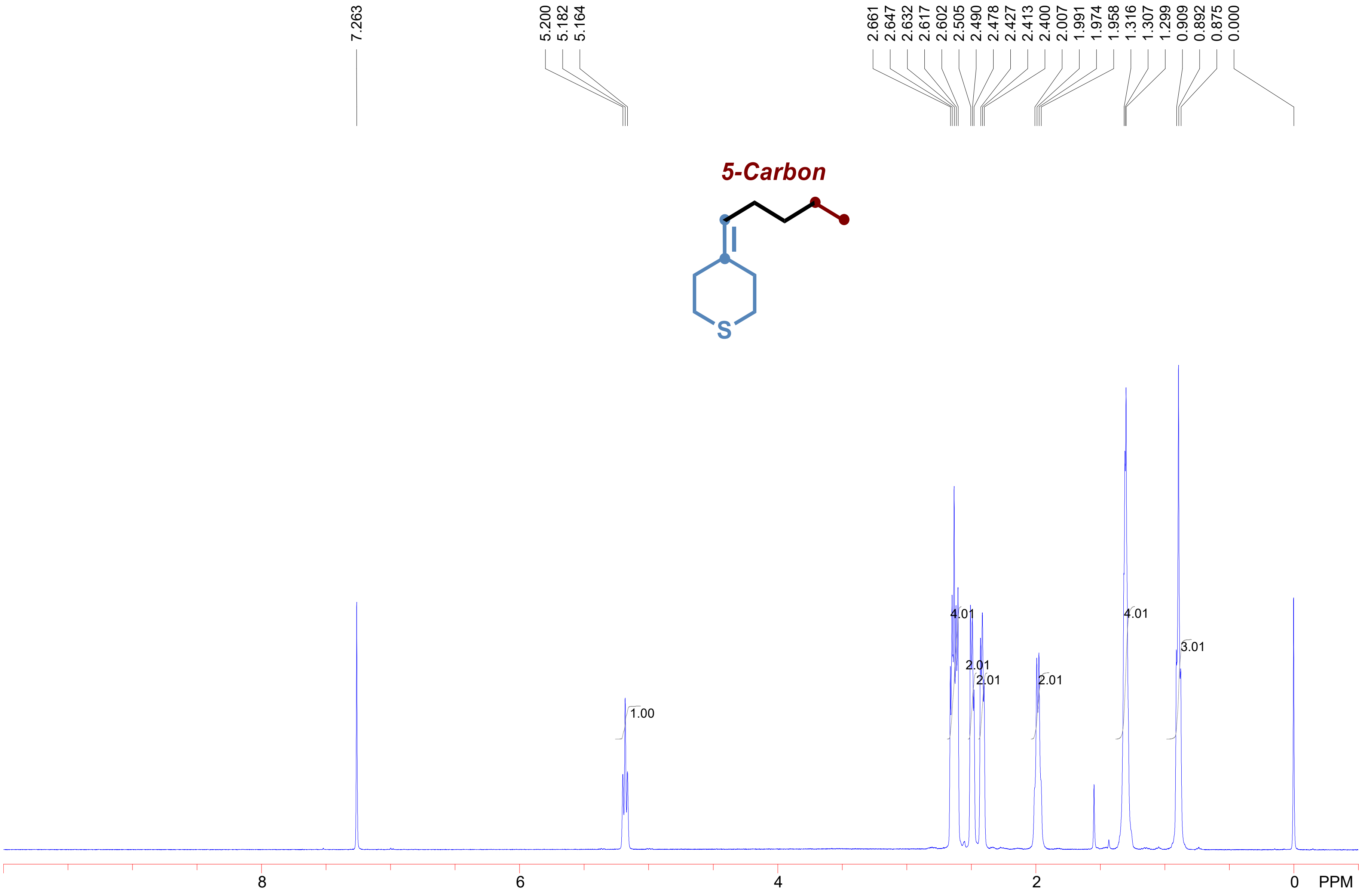

$^{13}$C NMR-spectrum (100 MHz, $CDCl_3$) of 25a

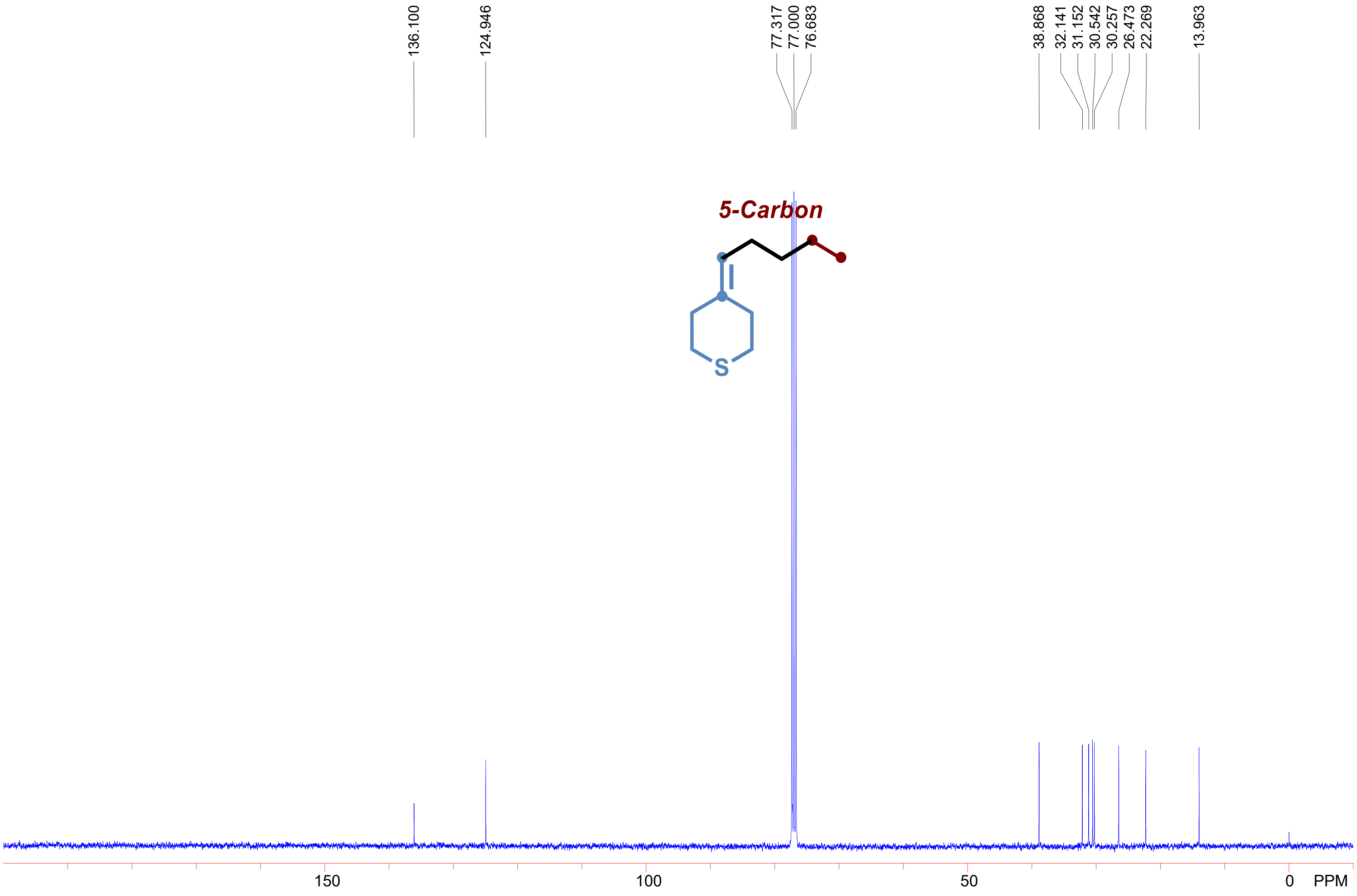

$^{1}$H NMR-spectrum (400 MHz, CDCl3) of 26a

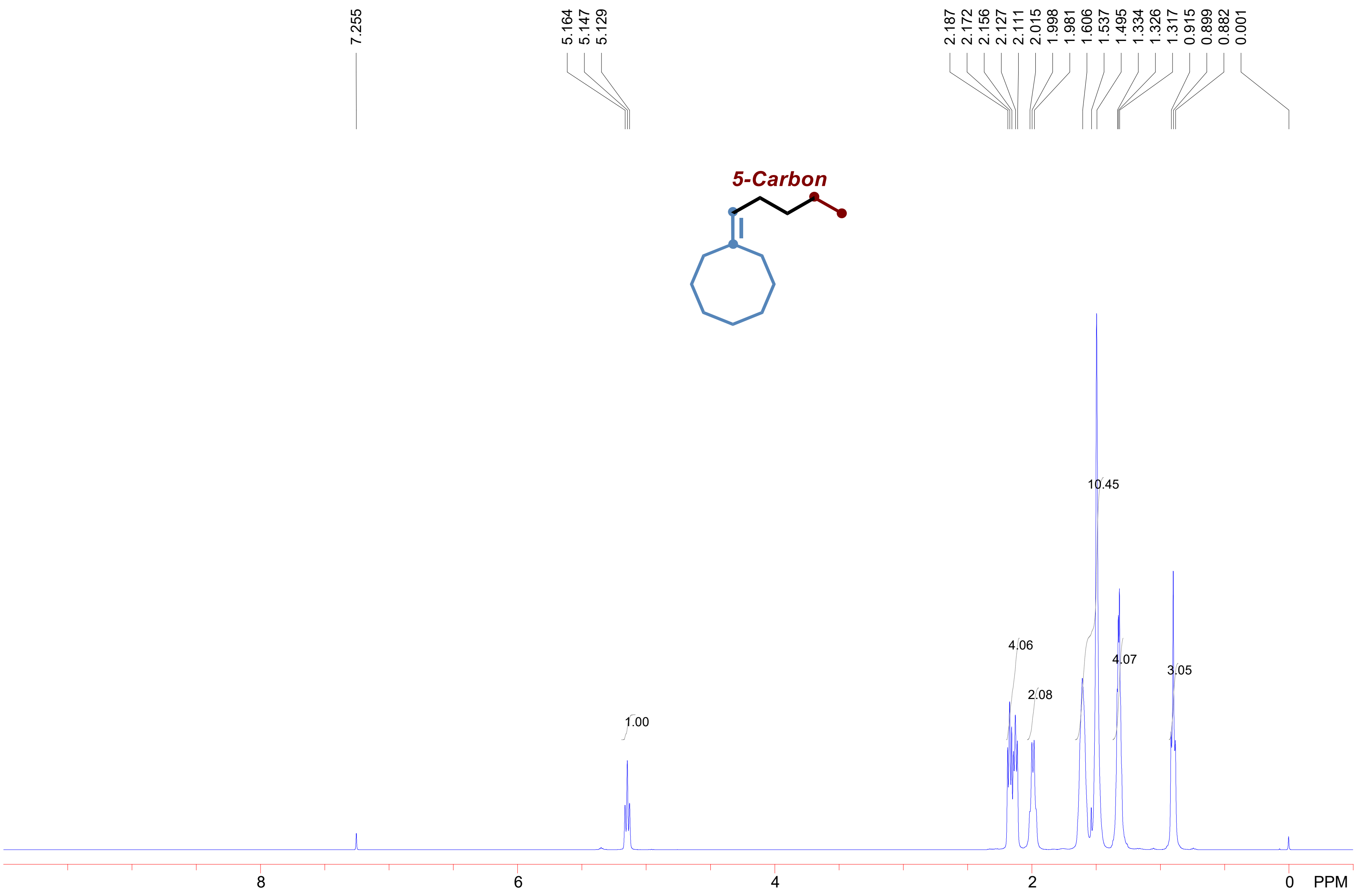

$^{13}$C NMR-spectrum (100 MHz, $CDCl_3$) of 26a

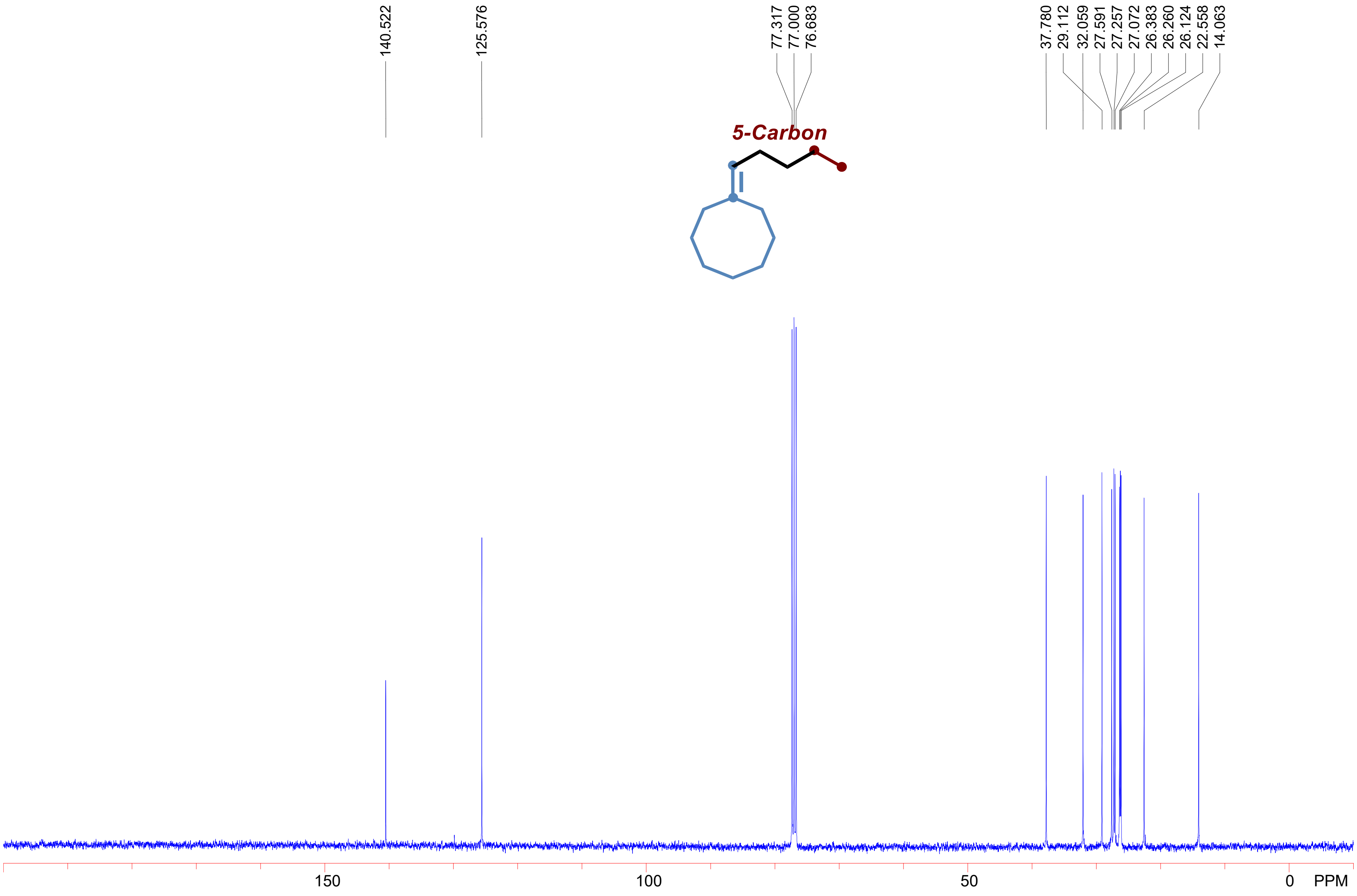

$^{1}$H NMR-spectrum (400 MHz, $CDCl_3$) of 27a

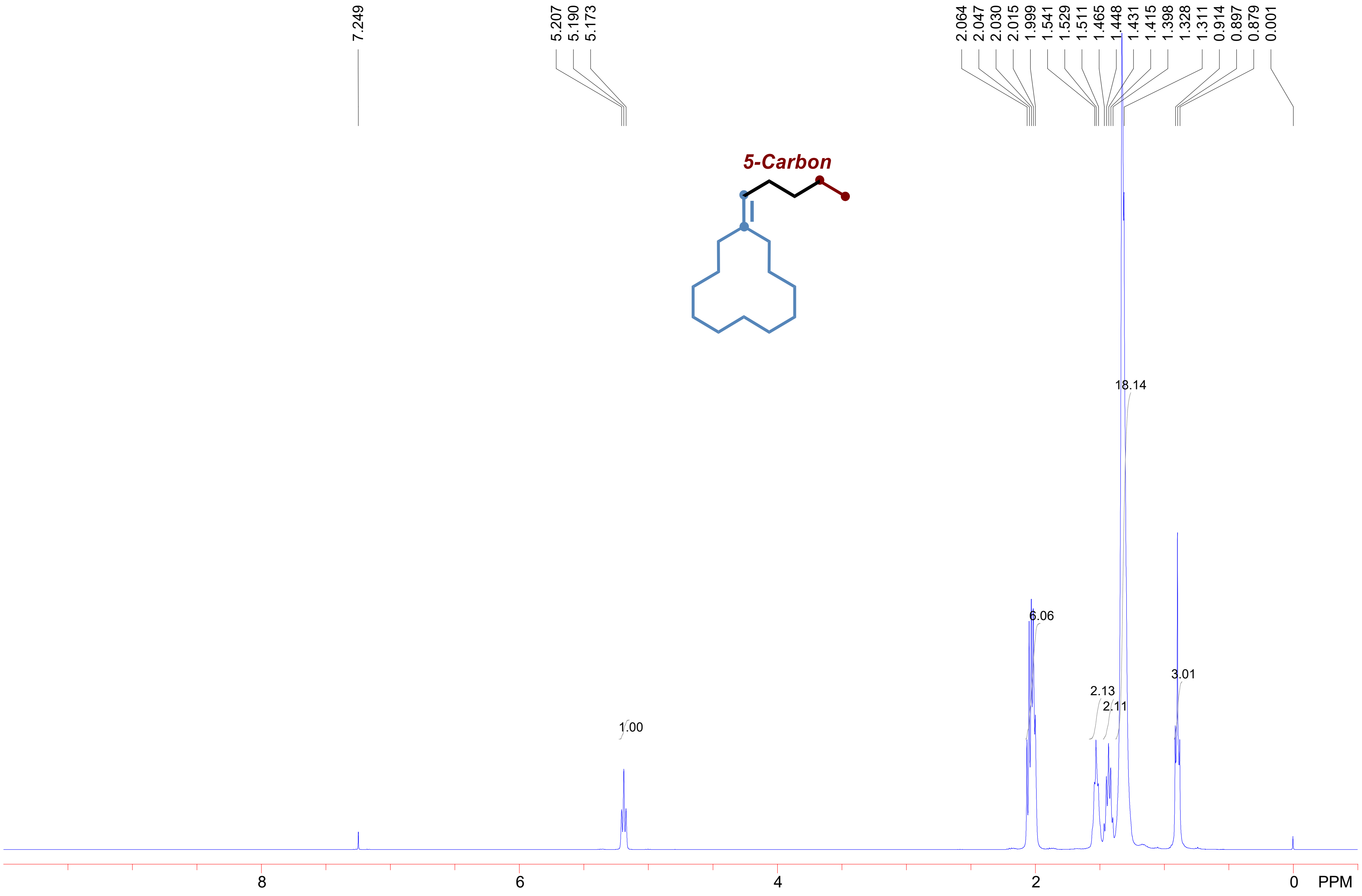

**$^{13}$C NMR-spectrum (100 MHz, $CDCl_3$) of 27a**

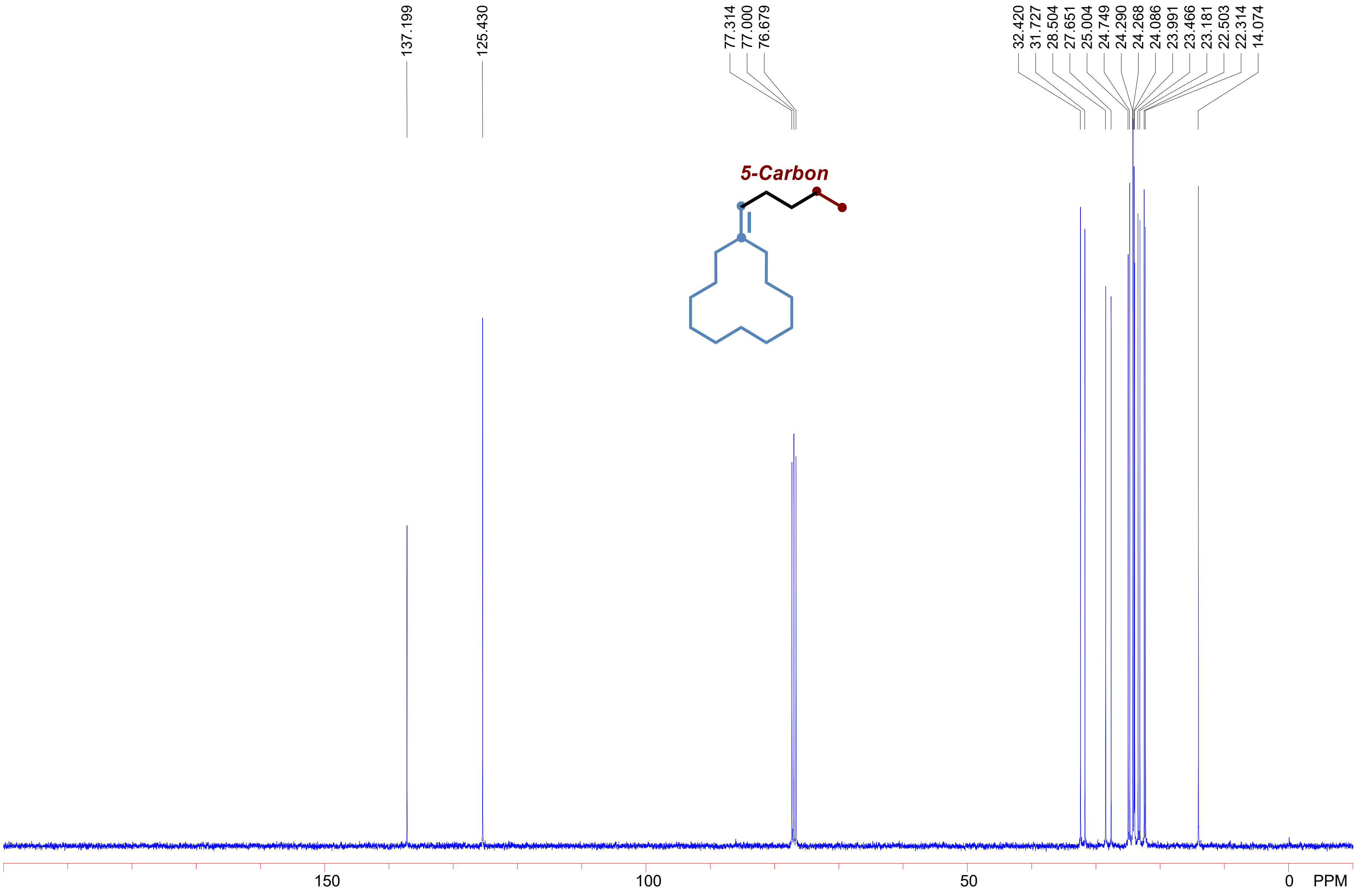

$^{1}$H NMR-spectrum (400 MHz, CDCl3) of 28a

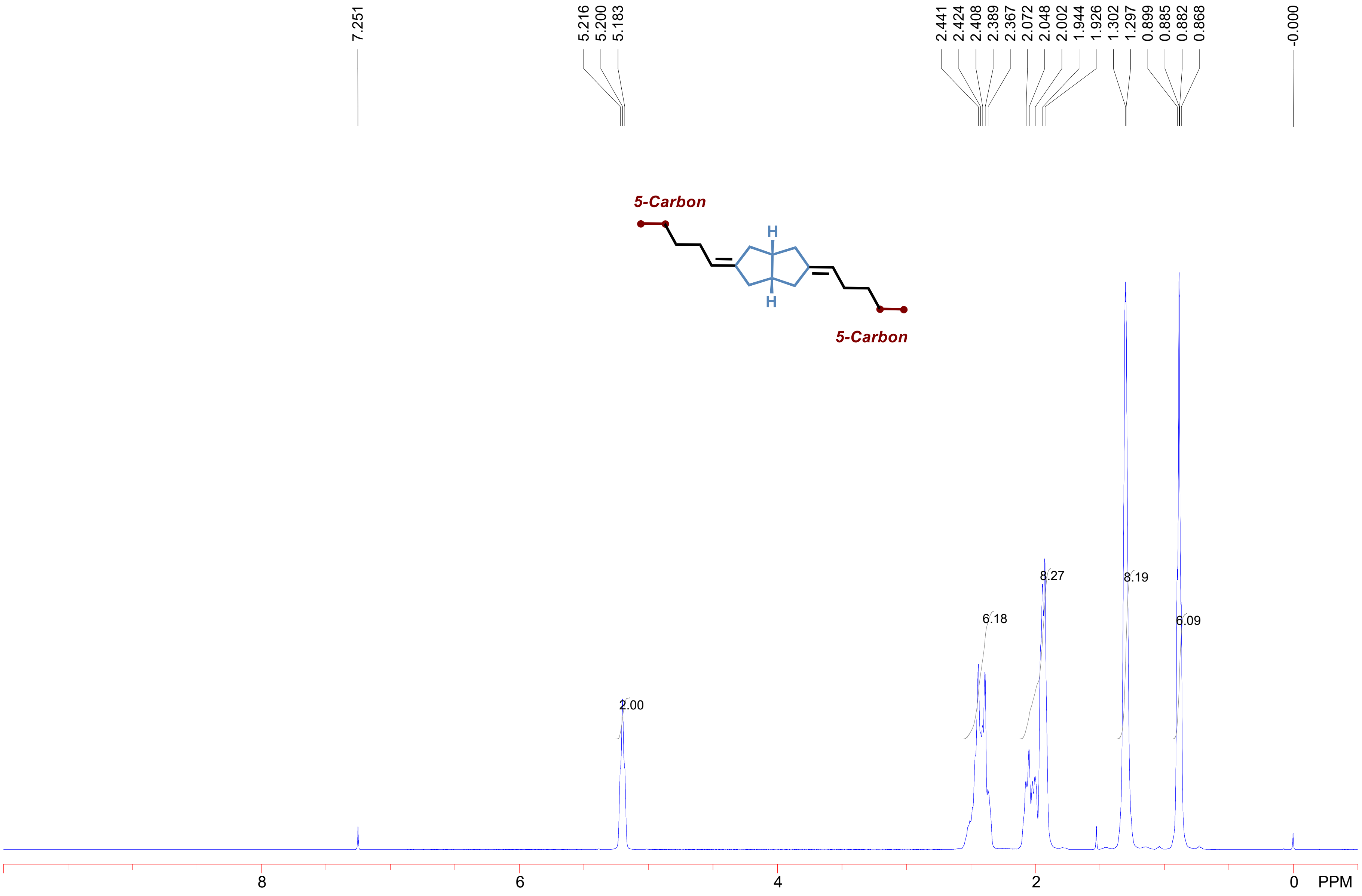

**$^{13}C$ NMR-spectrum (100 MHz, $CDCl_3$) of 28a**

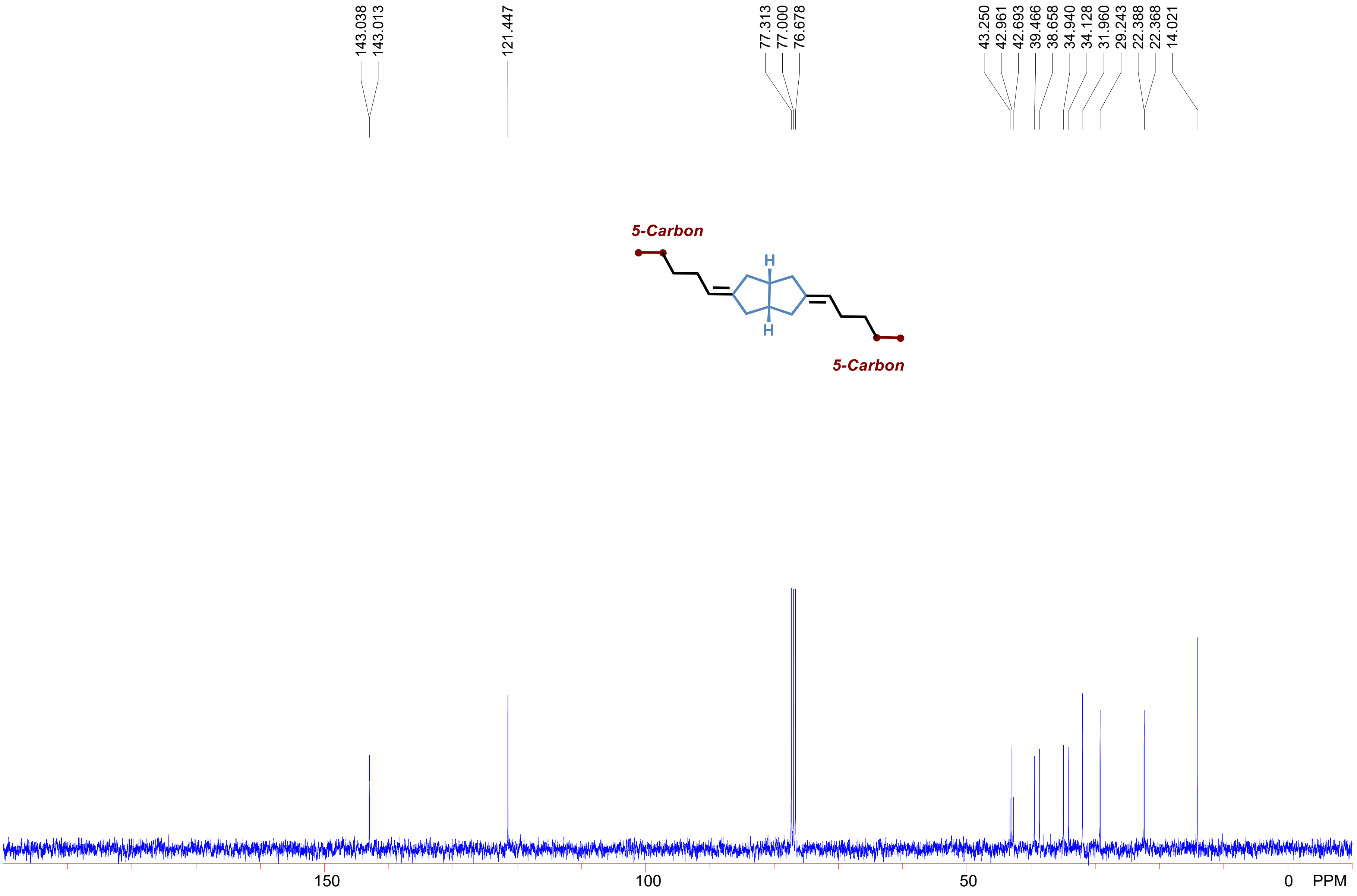

# $^{1}$H NMR-spectrum (400 MHz, CDCl3) of 29a

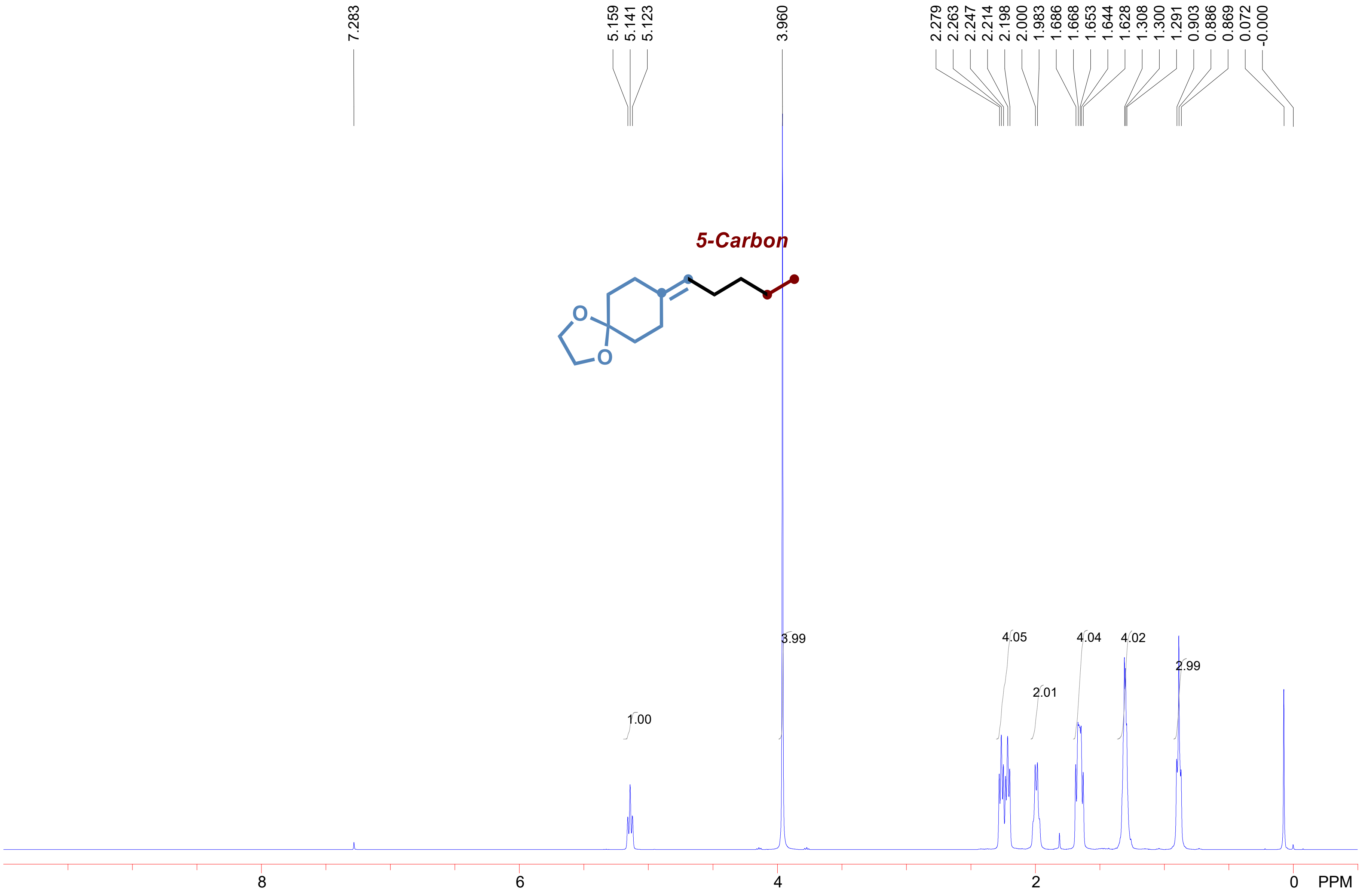

$^{13}$C NMR-spectrum (100 MHz, $CDCl_3$) of 29a

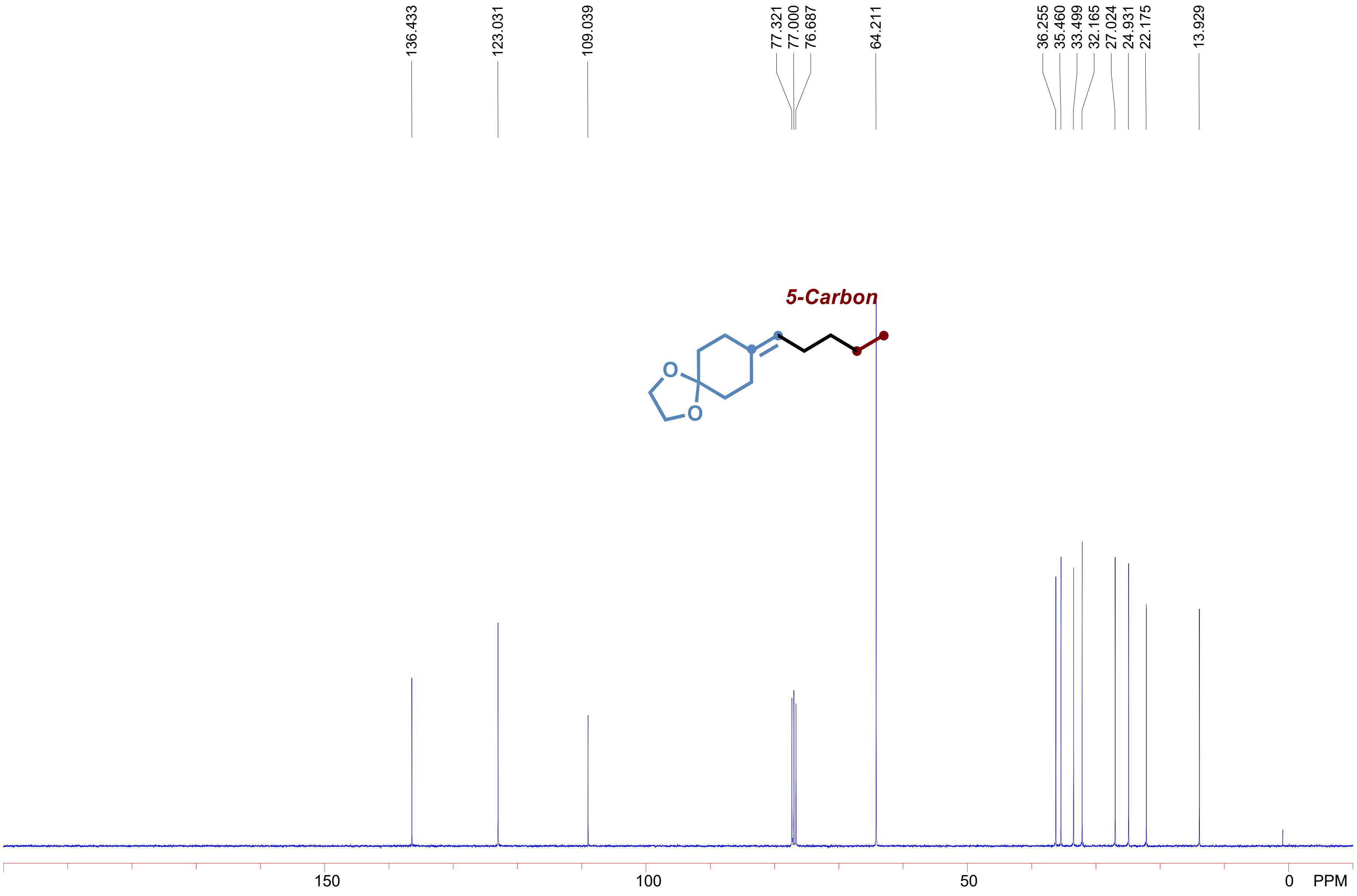

**$^{1}$H NMR-spectrum (400 MHz, CDCl3) of 30a**

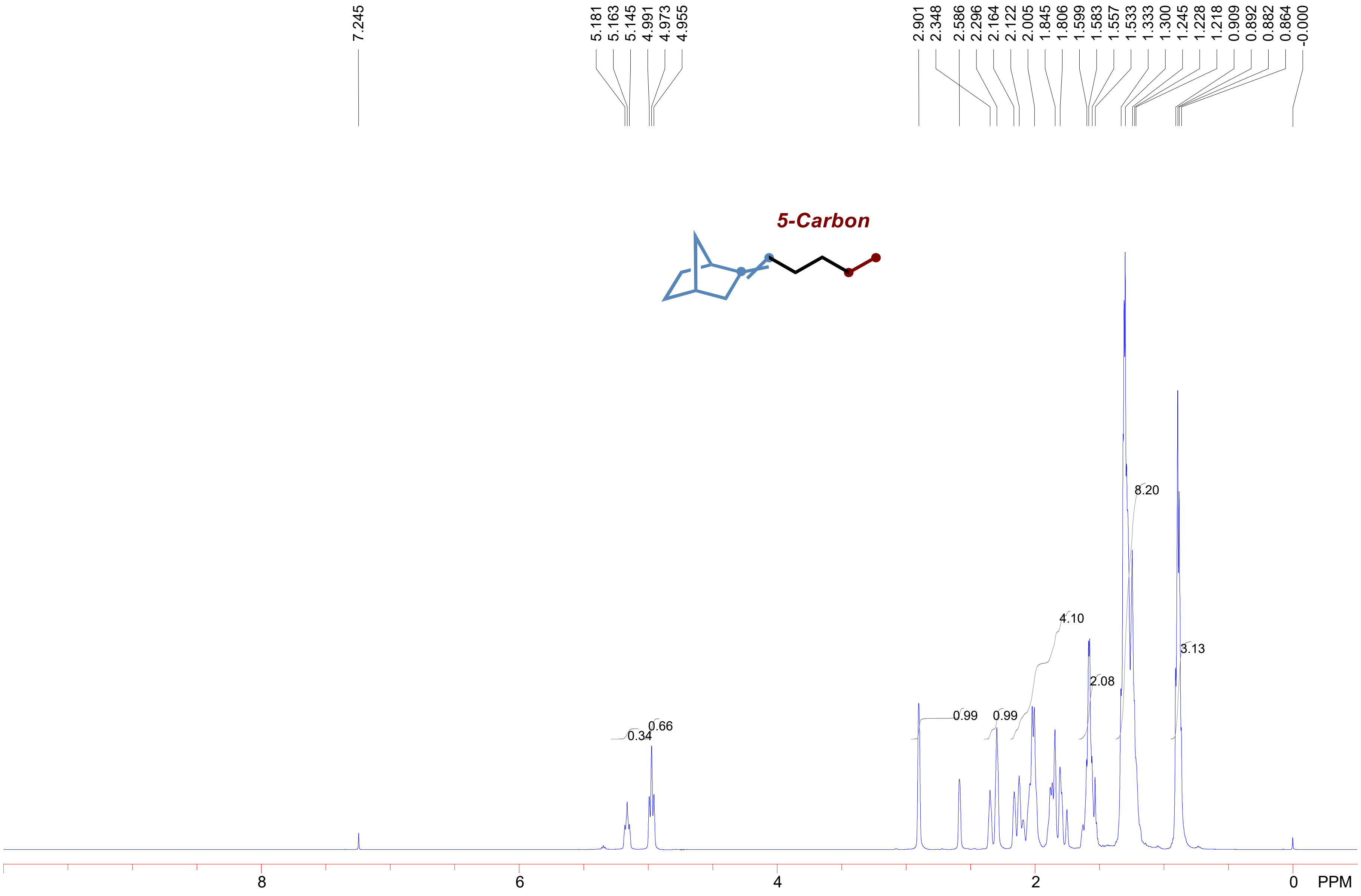

**$^{13}$C NMR-spectrum (100 MHz, $CDCl_3$) of 30a**

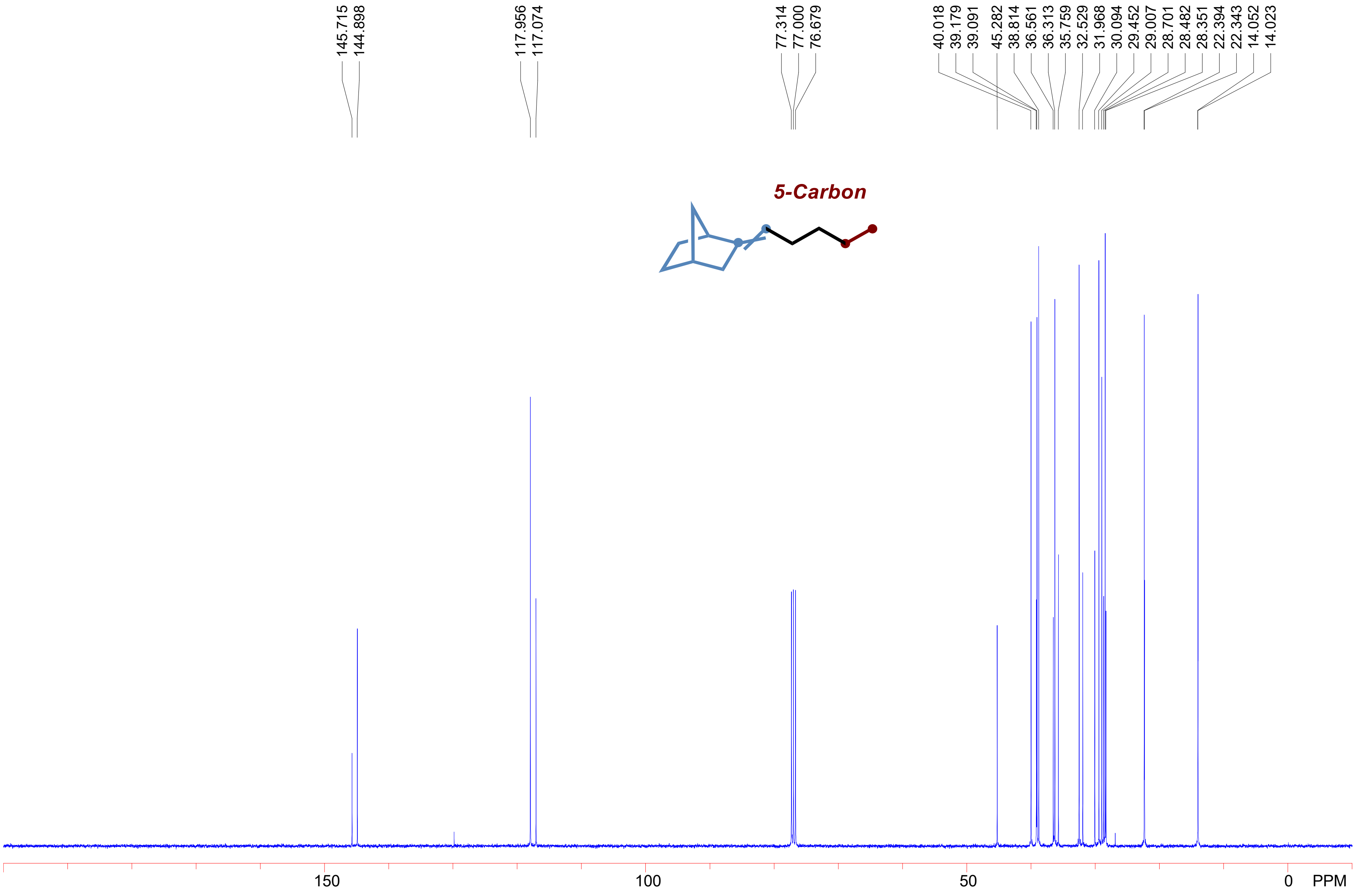

$^{1}$H NMR-spectrum (400 MHz, CDCl3) of 31a

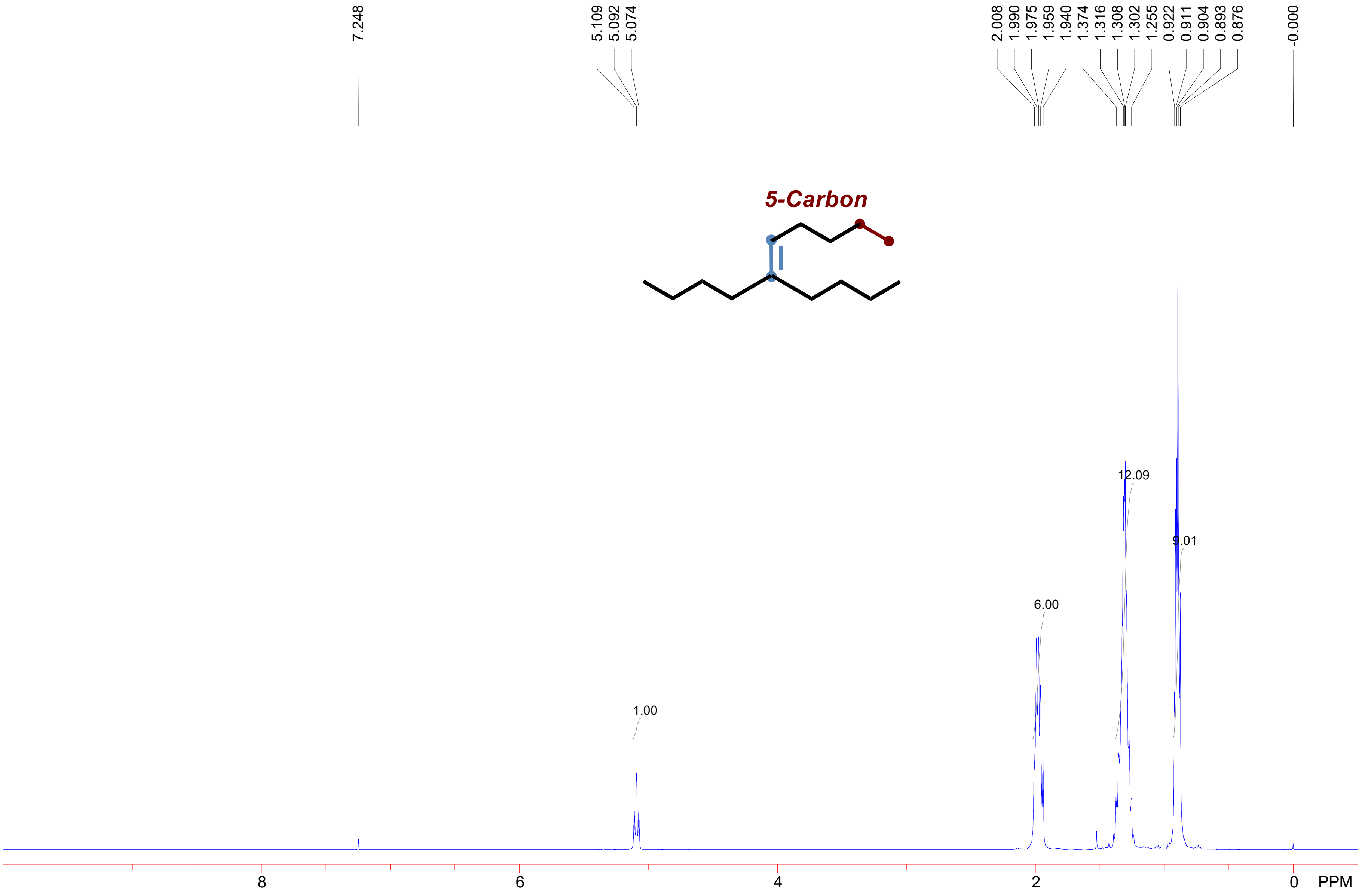

**$^{13}$C NMR-spectrum (100 MHz, $CDCl_3$) of 31a**

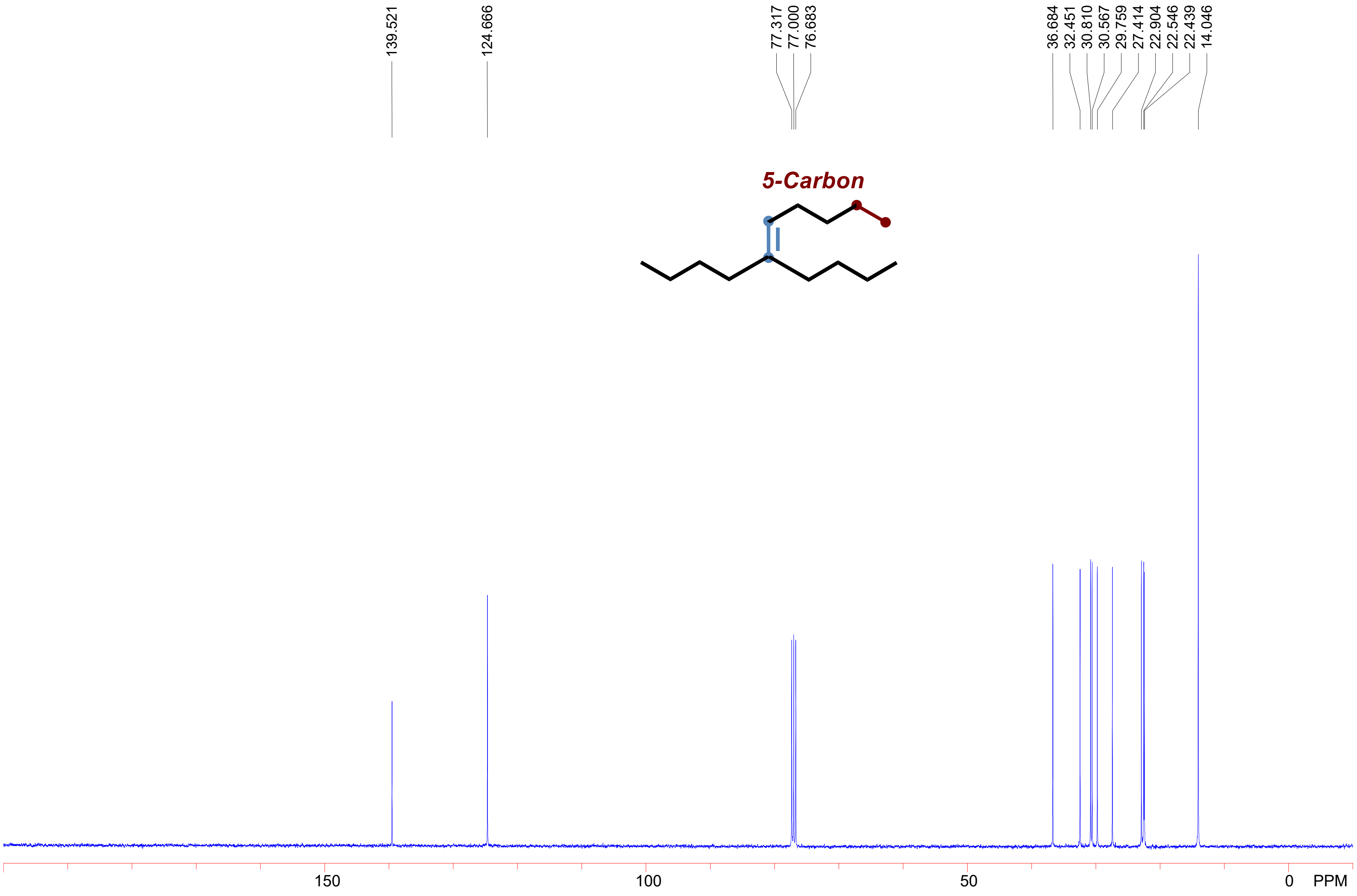

**$^{1}$H NMR-spectrum (400 MHz, $CDCl_3$) of 32a**

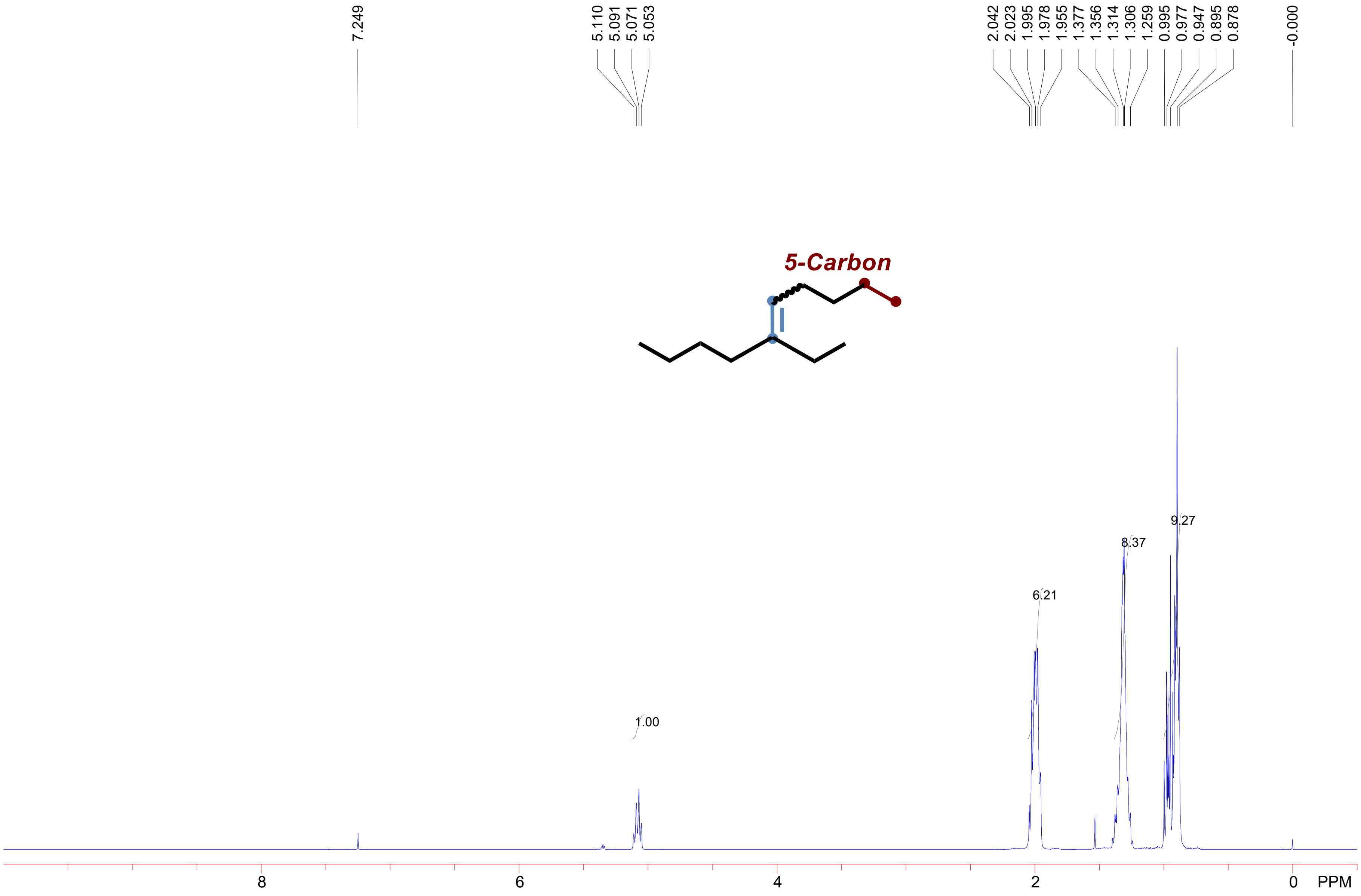

**$^{13}$C NMR-spectrum (100 MHz, $CDCl_3$) of 32a**

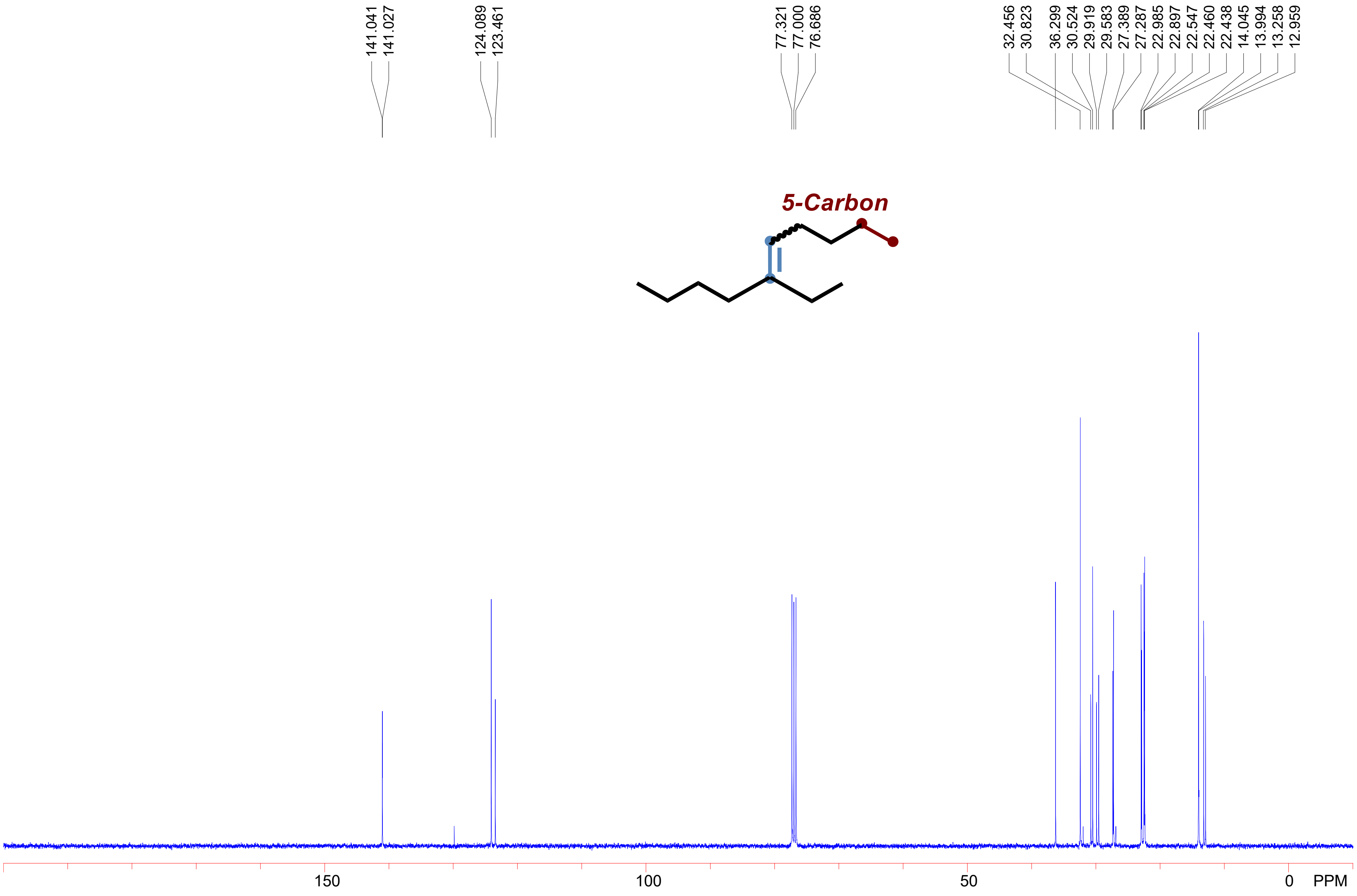

$^{1}$H NMR-spectrum (400 MHz, $CDCl_3$) of 33a

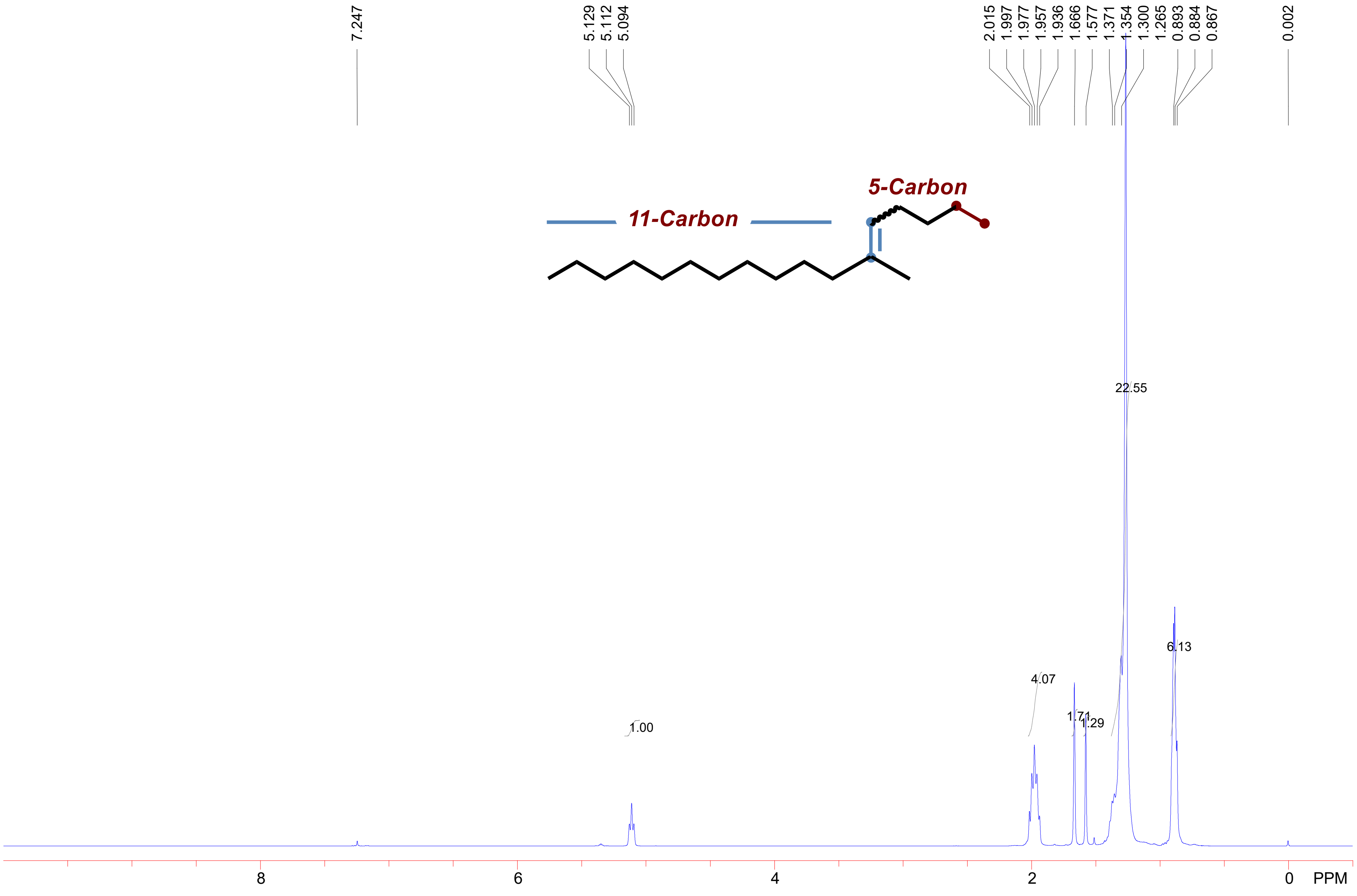

$^{13}$C NMR-spectrum (100 MHz, CDCl3) of 33a

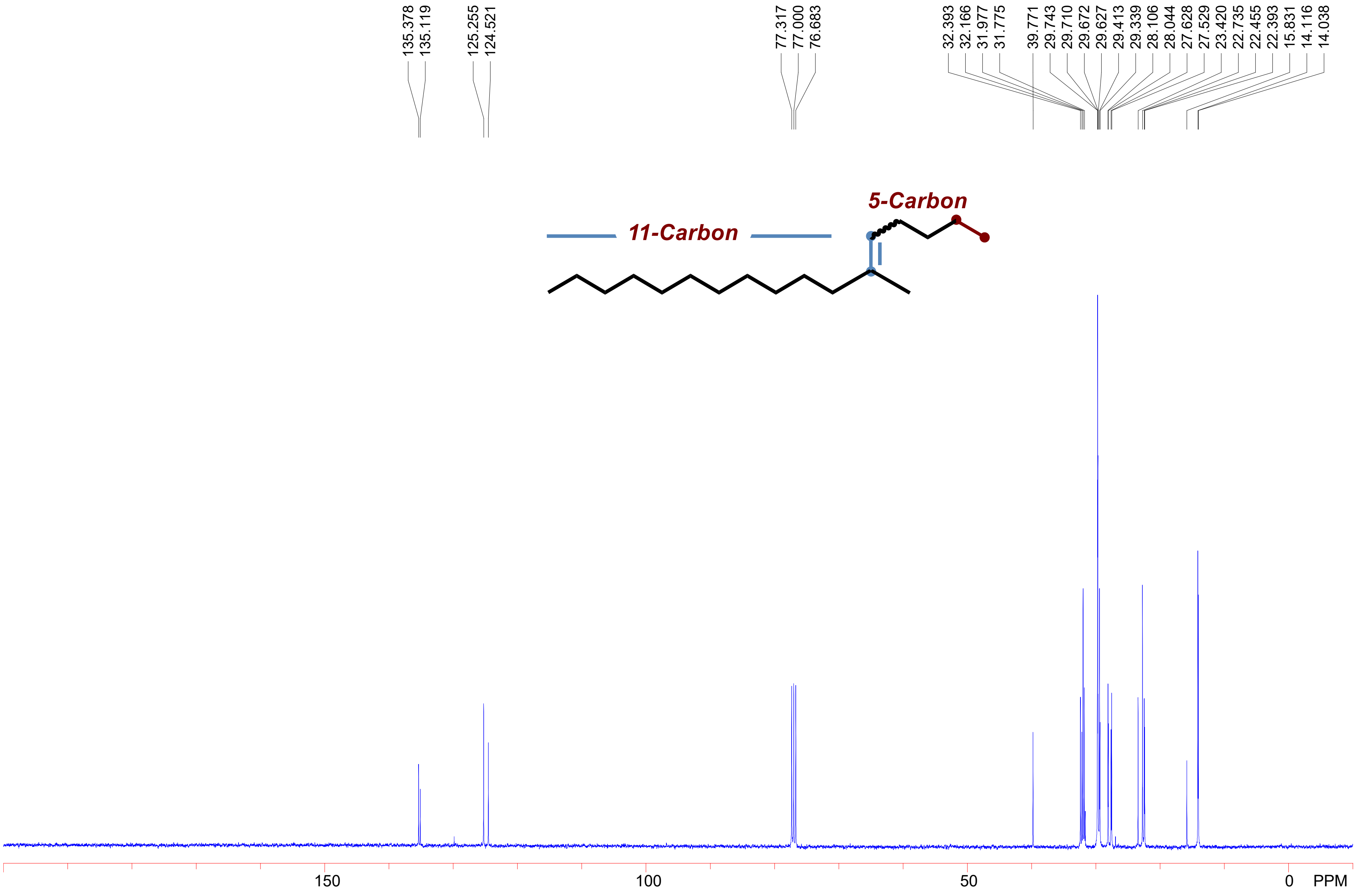

$^1$H NMR-spectrum (400 MHz, CDCl$_3$) of 34a

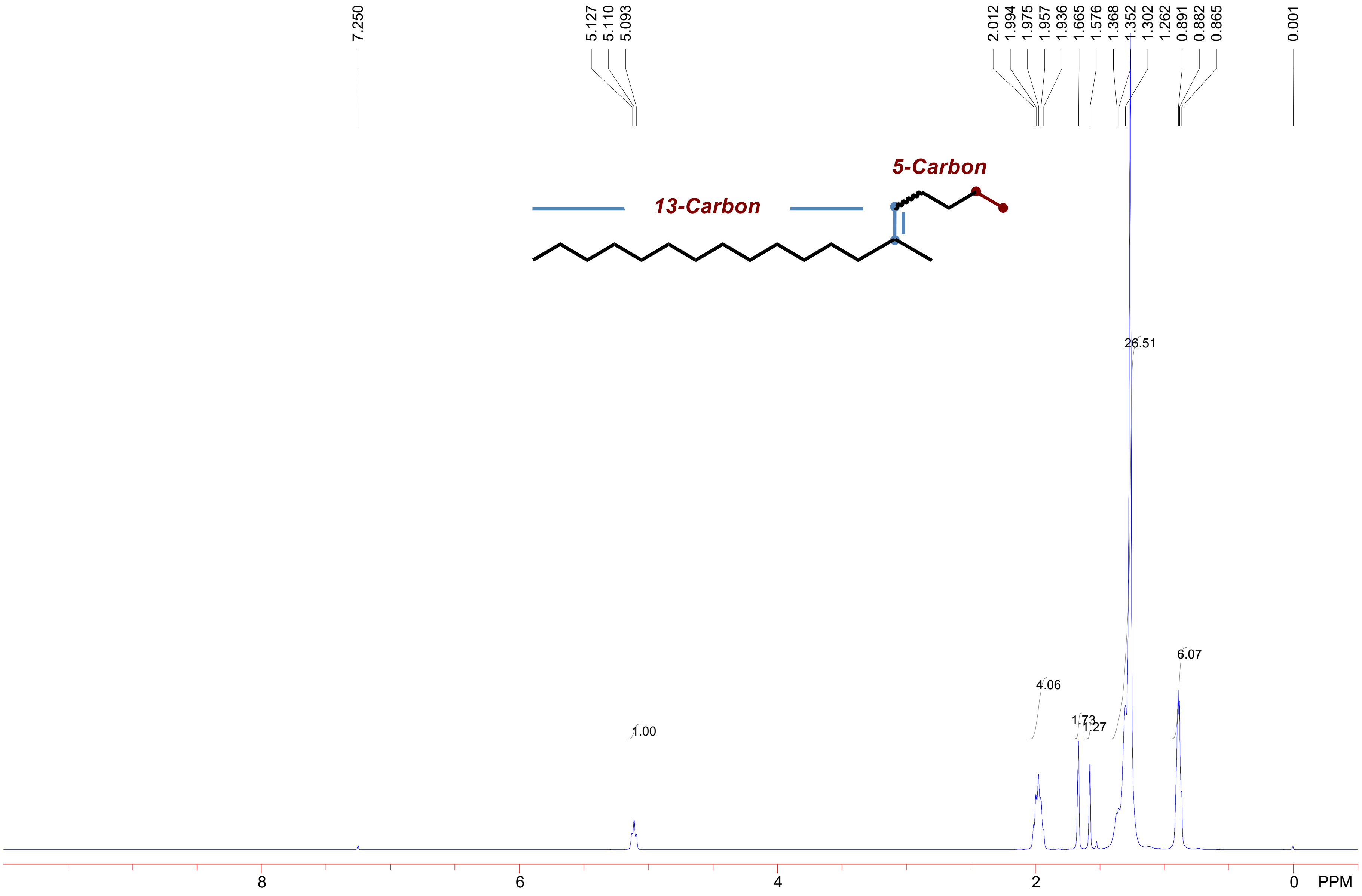

$^{13}$C NMR-spectrum (100 MHz, CDCl3) of 34a

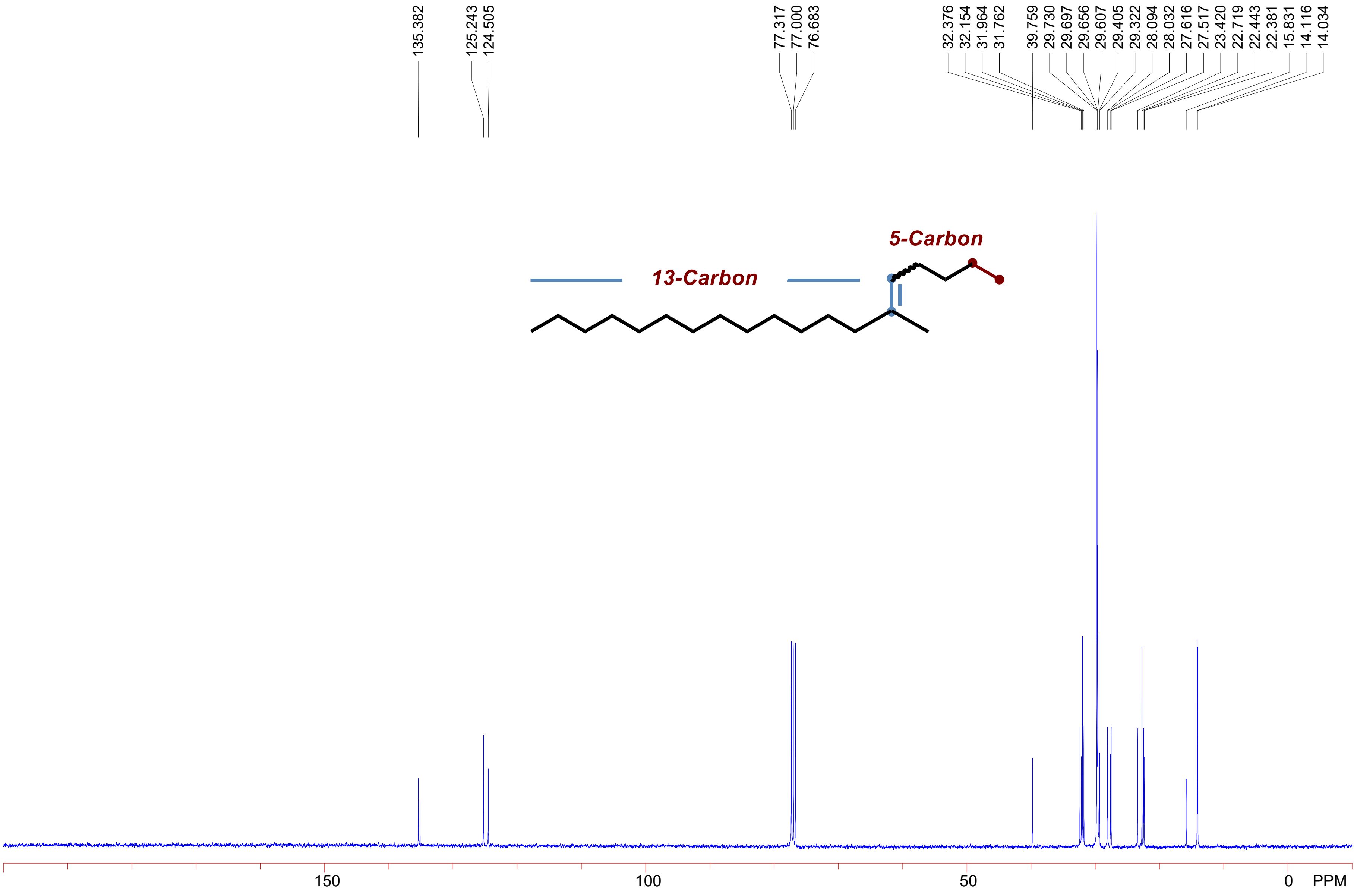

**$^{1}$H NMR-spectrum (400 MHz, CDCl3) of 35a**

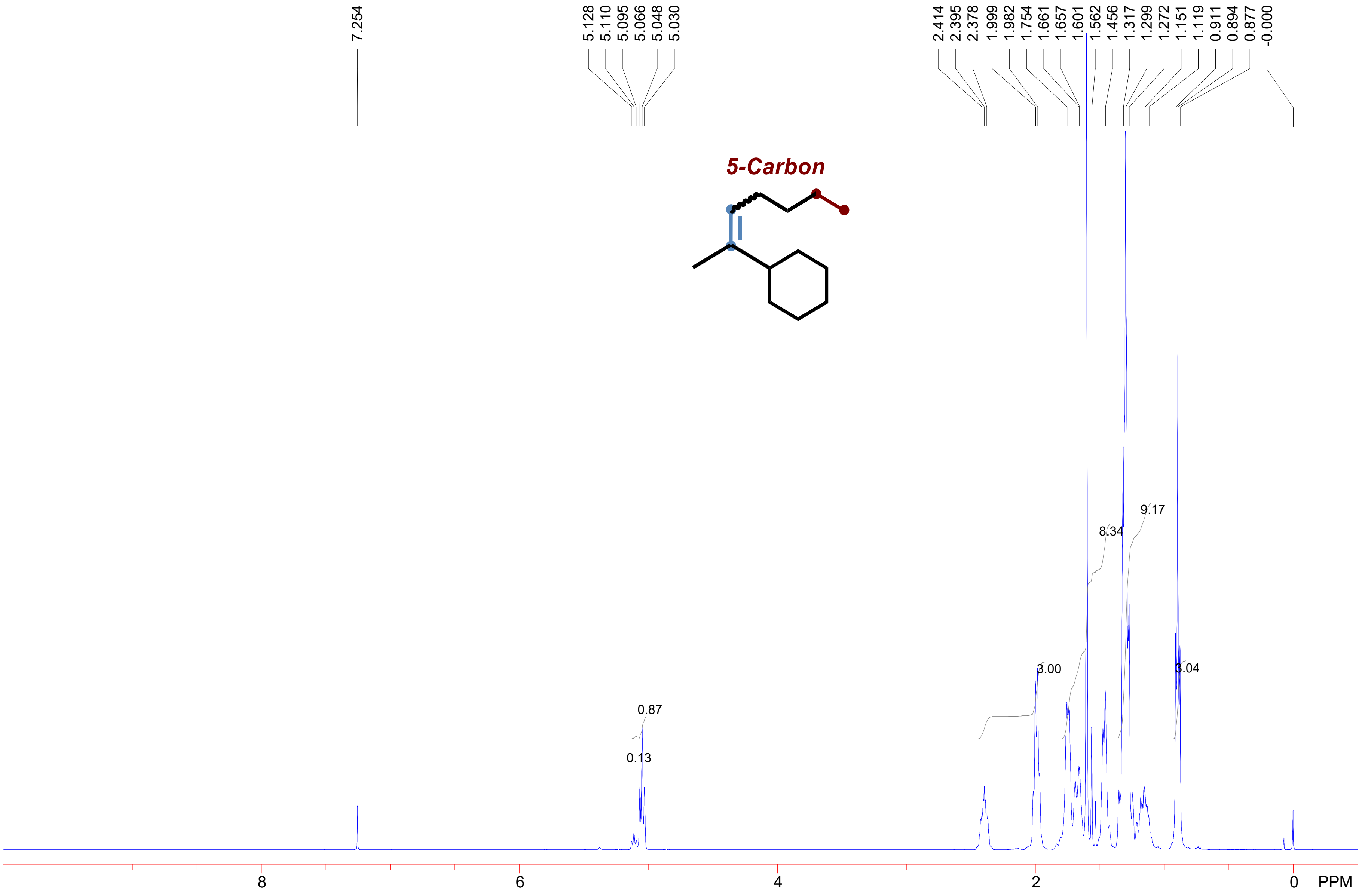

**$^{13}$C NMR-spectrum (100 MHz, $CDCl_3$) of 35a**

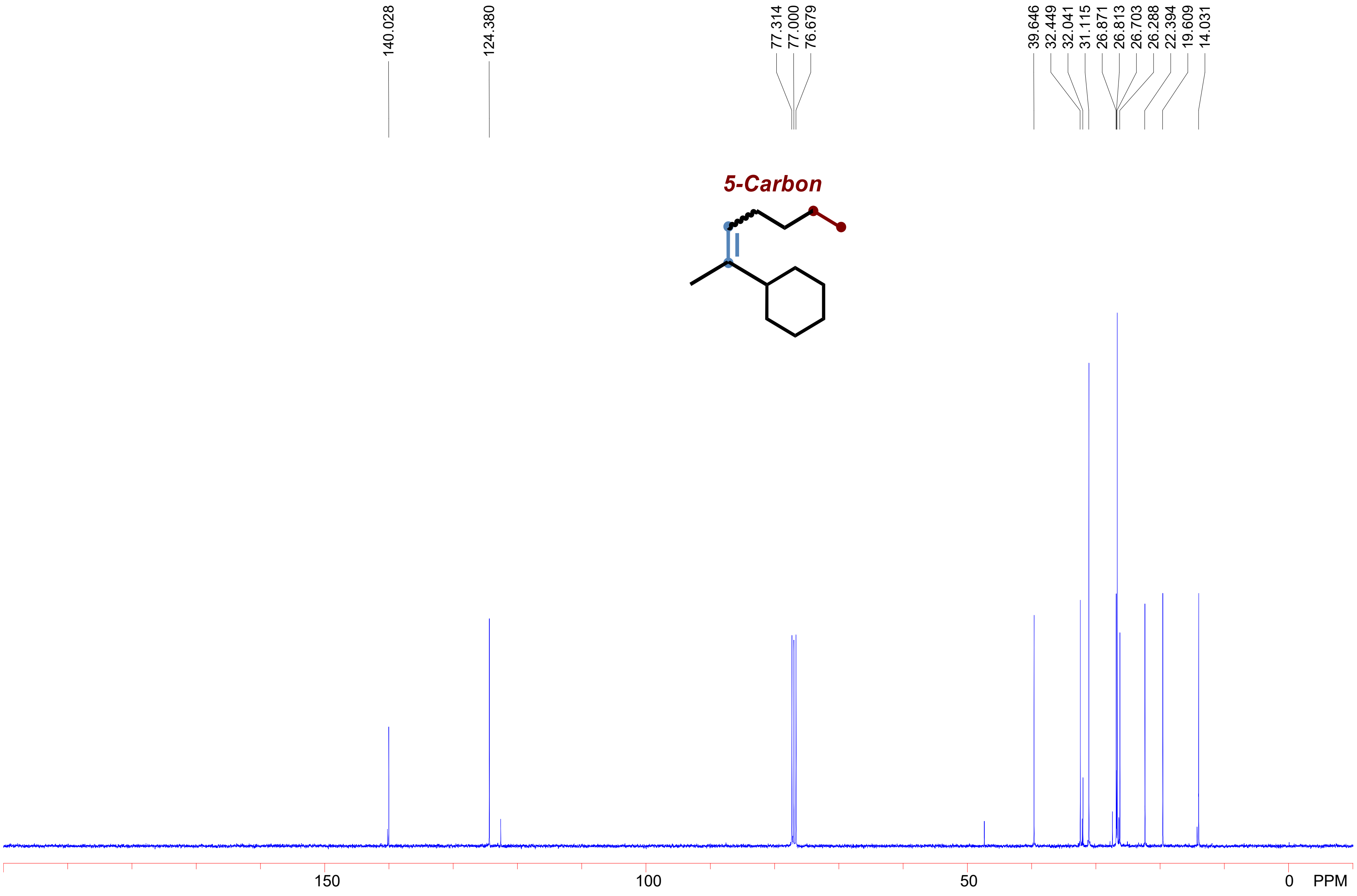

$^{1}$H NMR-spectrum (400 MHz, $CDCl_3$) of 36a

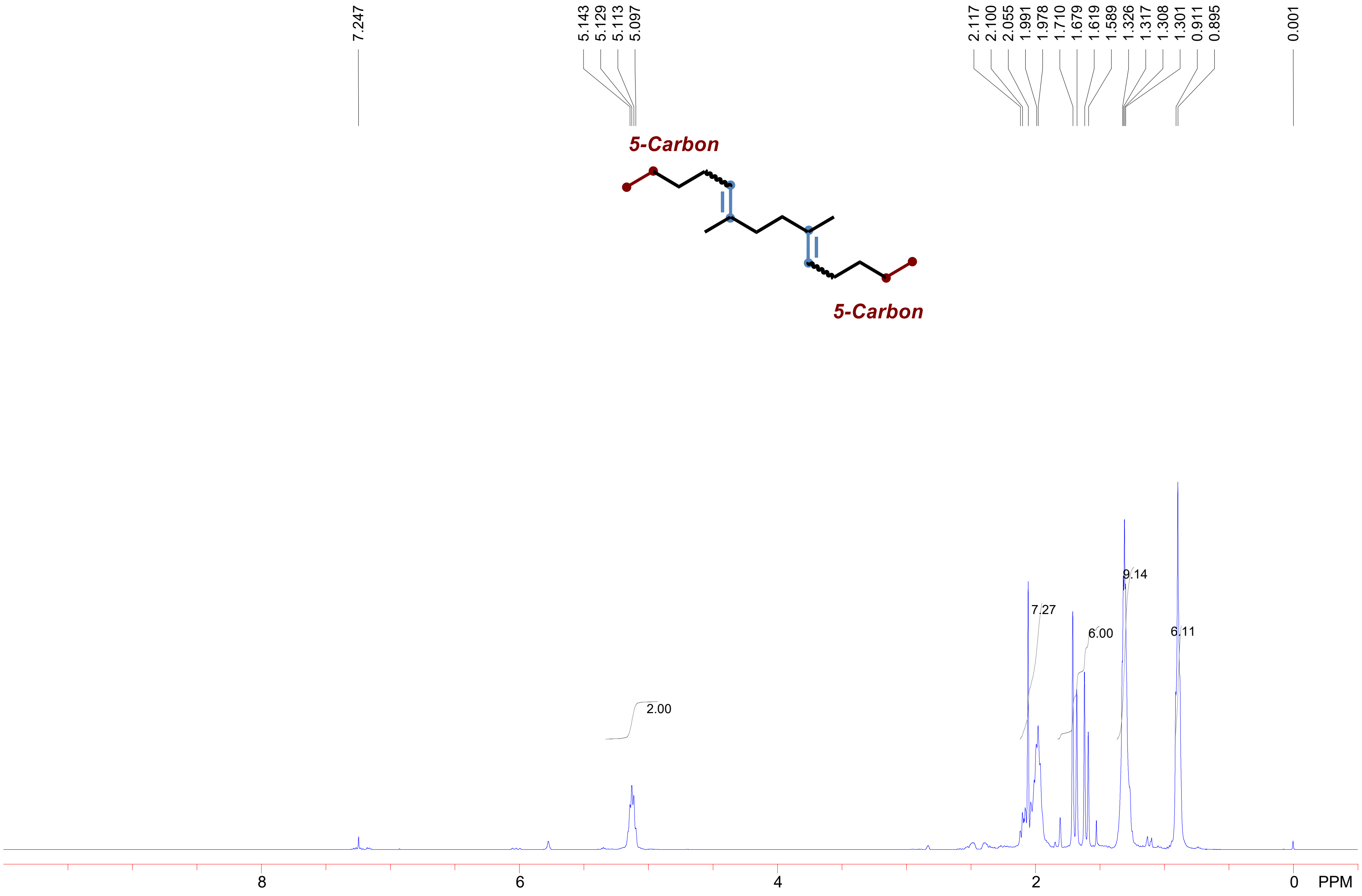

$^{13}$C NMR-spectrum (100 MHz, $CDCl_3$) of 36a

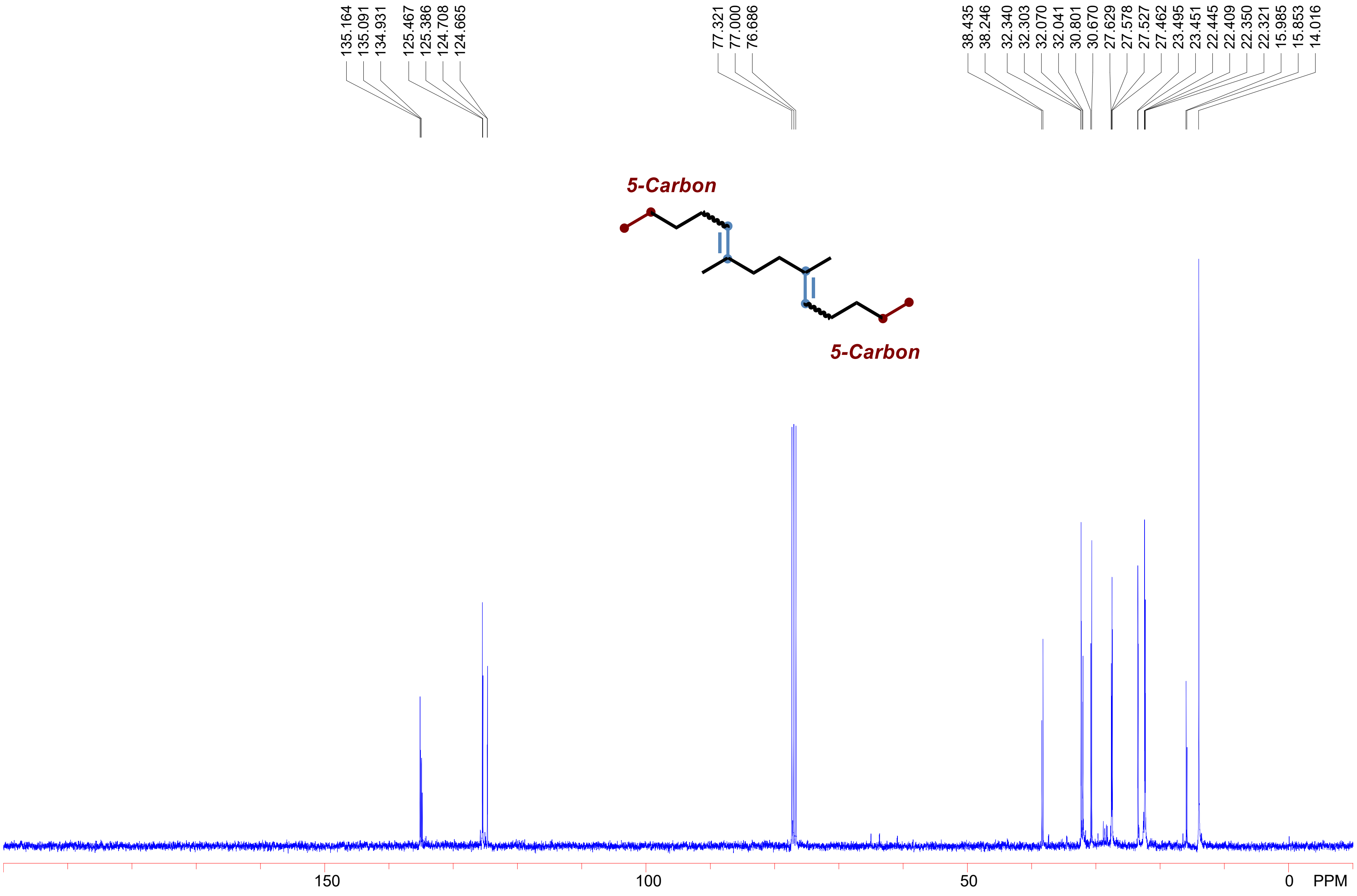

$^{1}$H NMR-spectrum (400 MHz, $CDCl_3$) of 37a

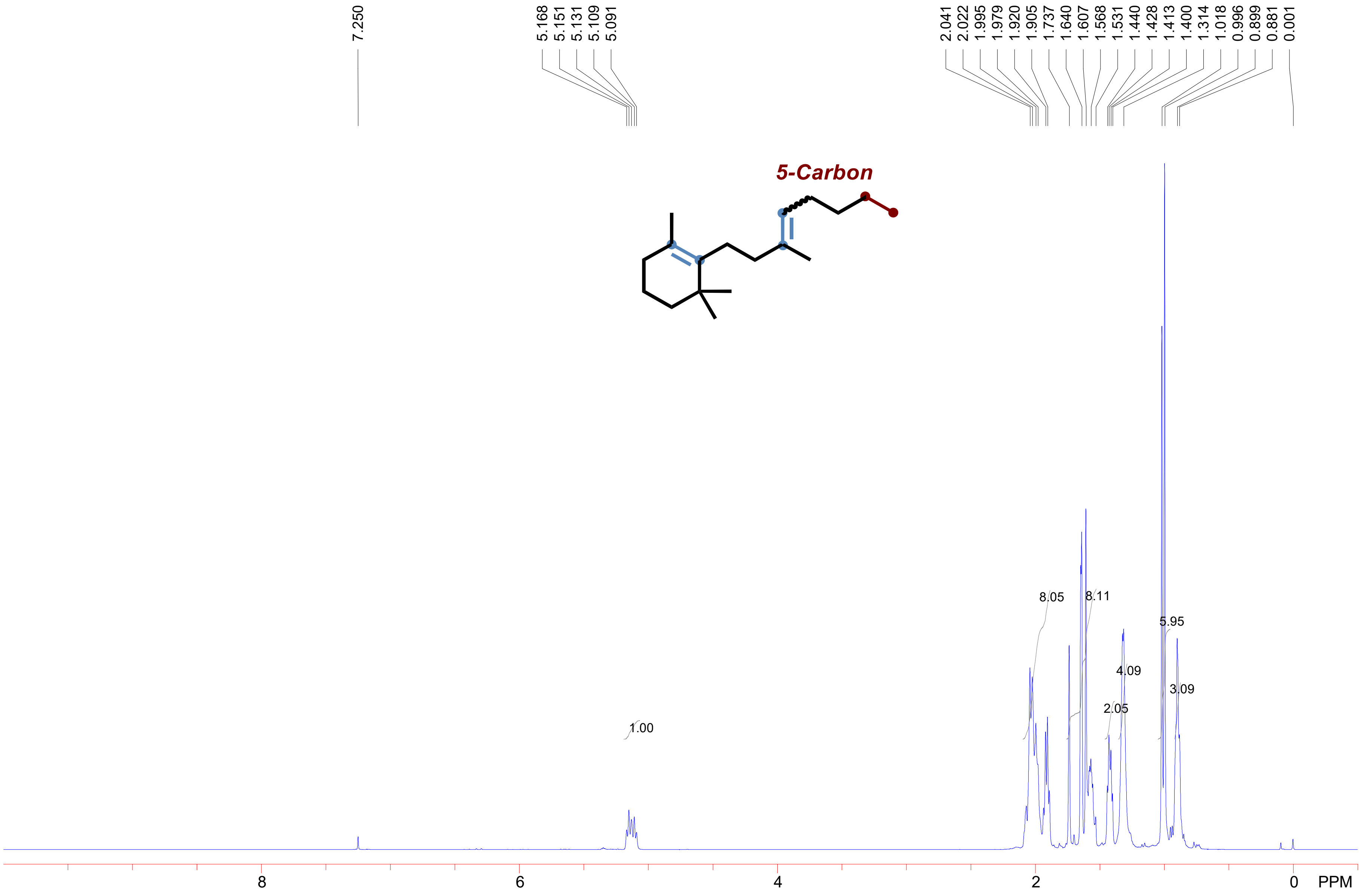

$^{13}$C NMR-spectrum (100 MHz, CDCl3) of 37a

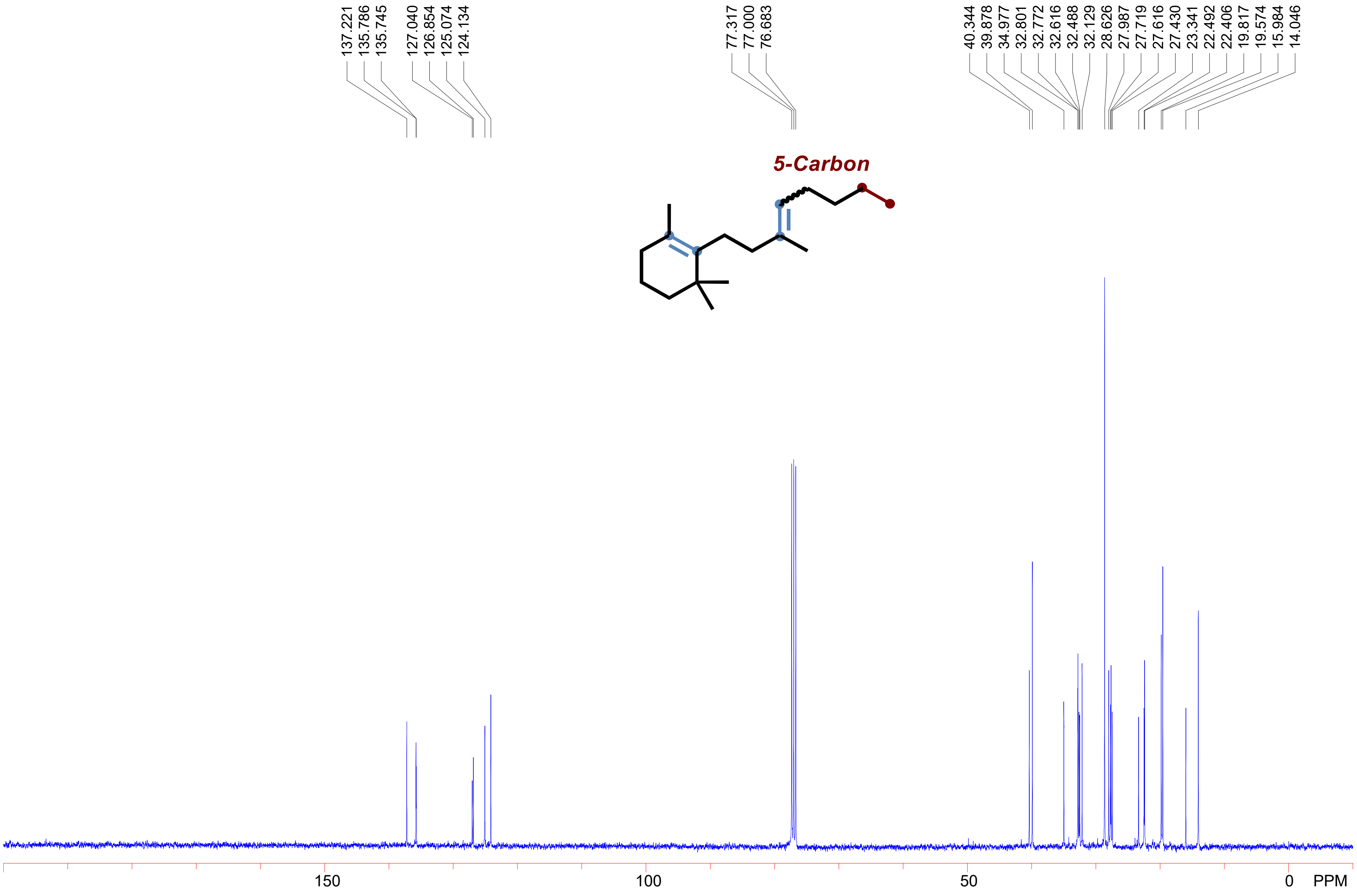

$^{1}$H NMR-spectrum (400 MHz, $CDCl_3$) of 38a

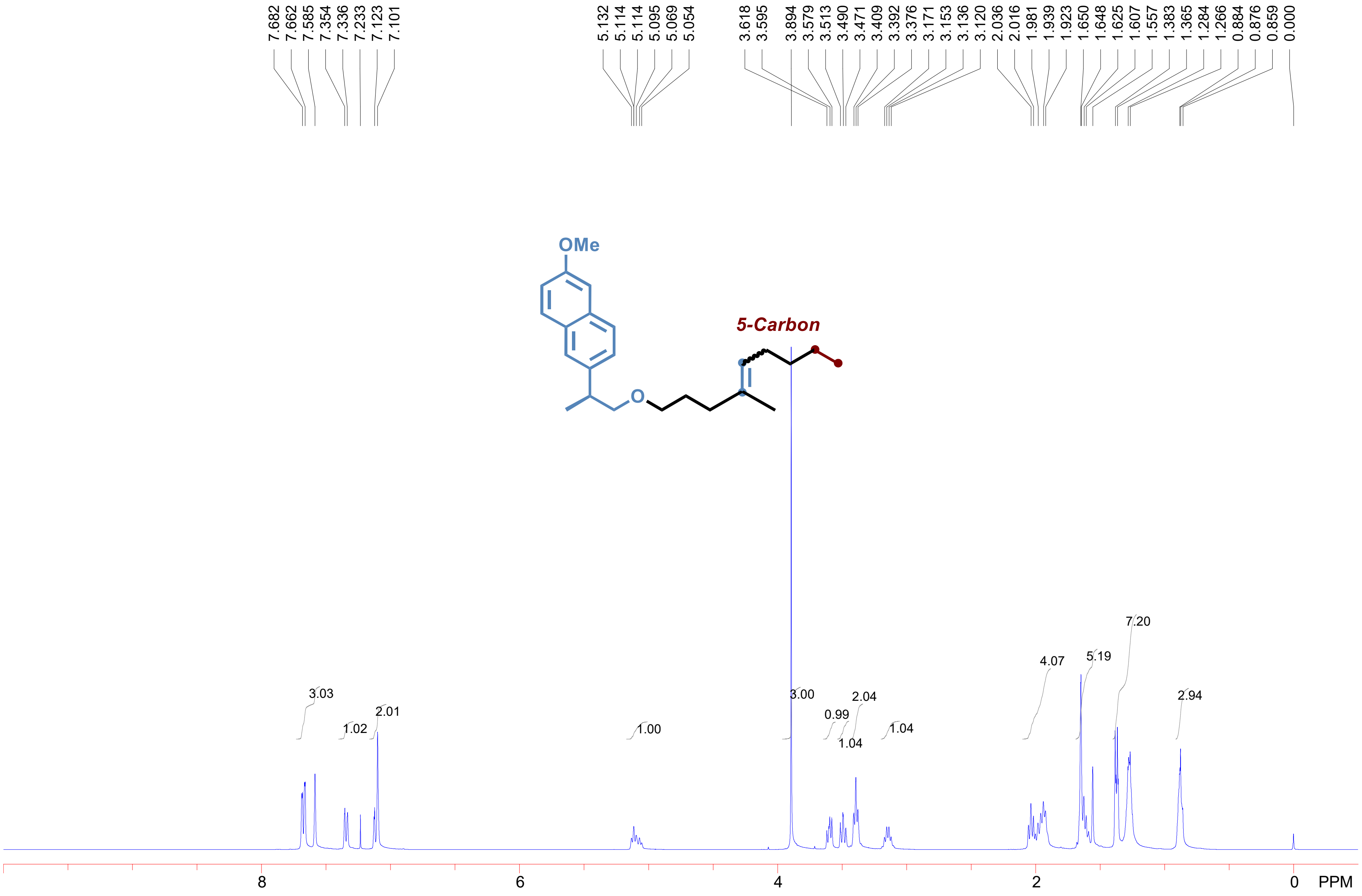

**$^{13}$C NMR-spectrum (100 MHz, $CDCl_3$) of 38a**

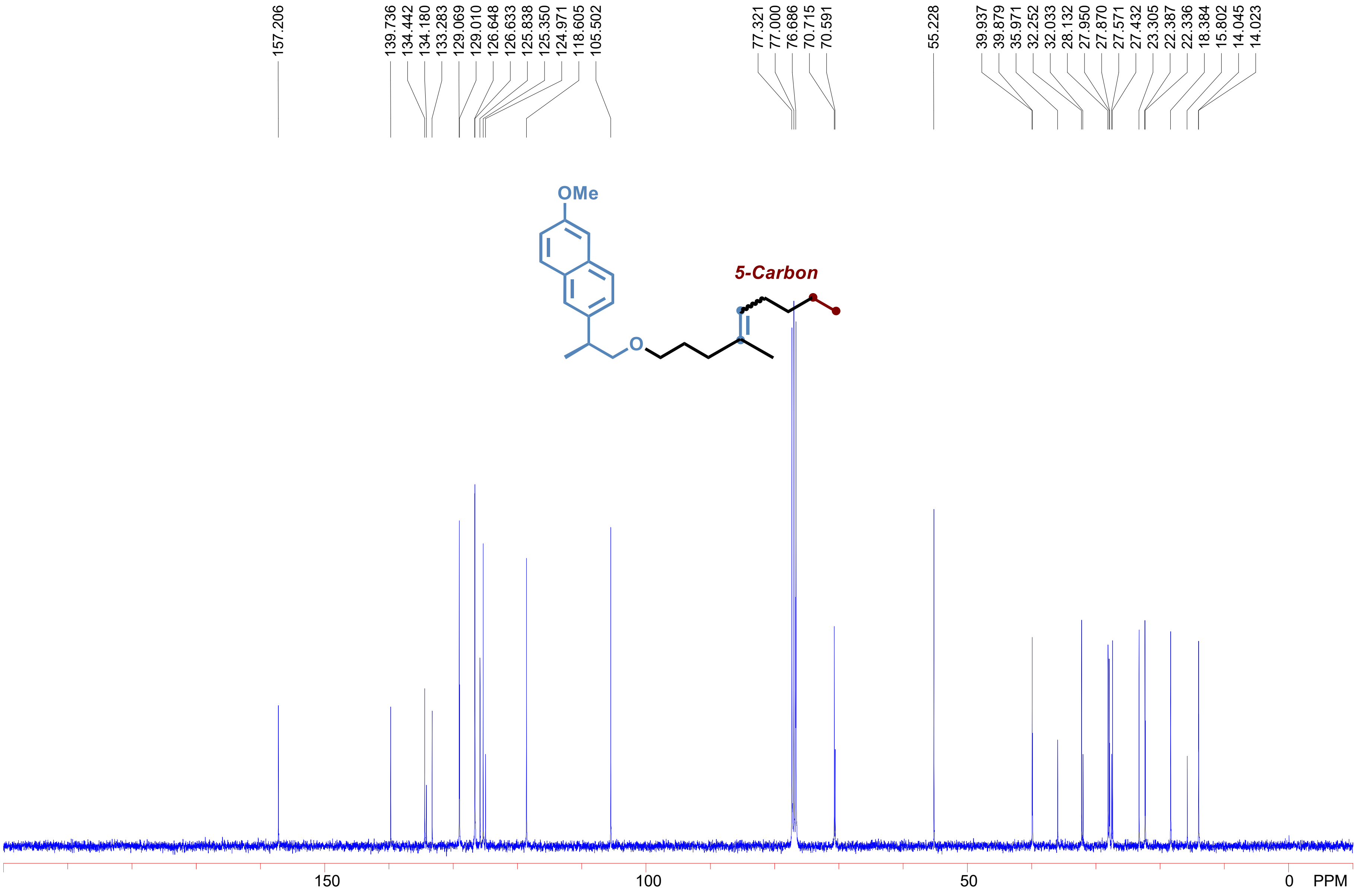

## $^{1}$H NMR-spectrum (400 MHz, $CDCl_3$) of 39a

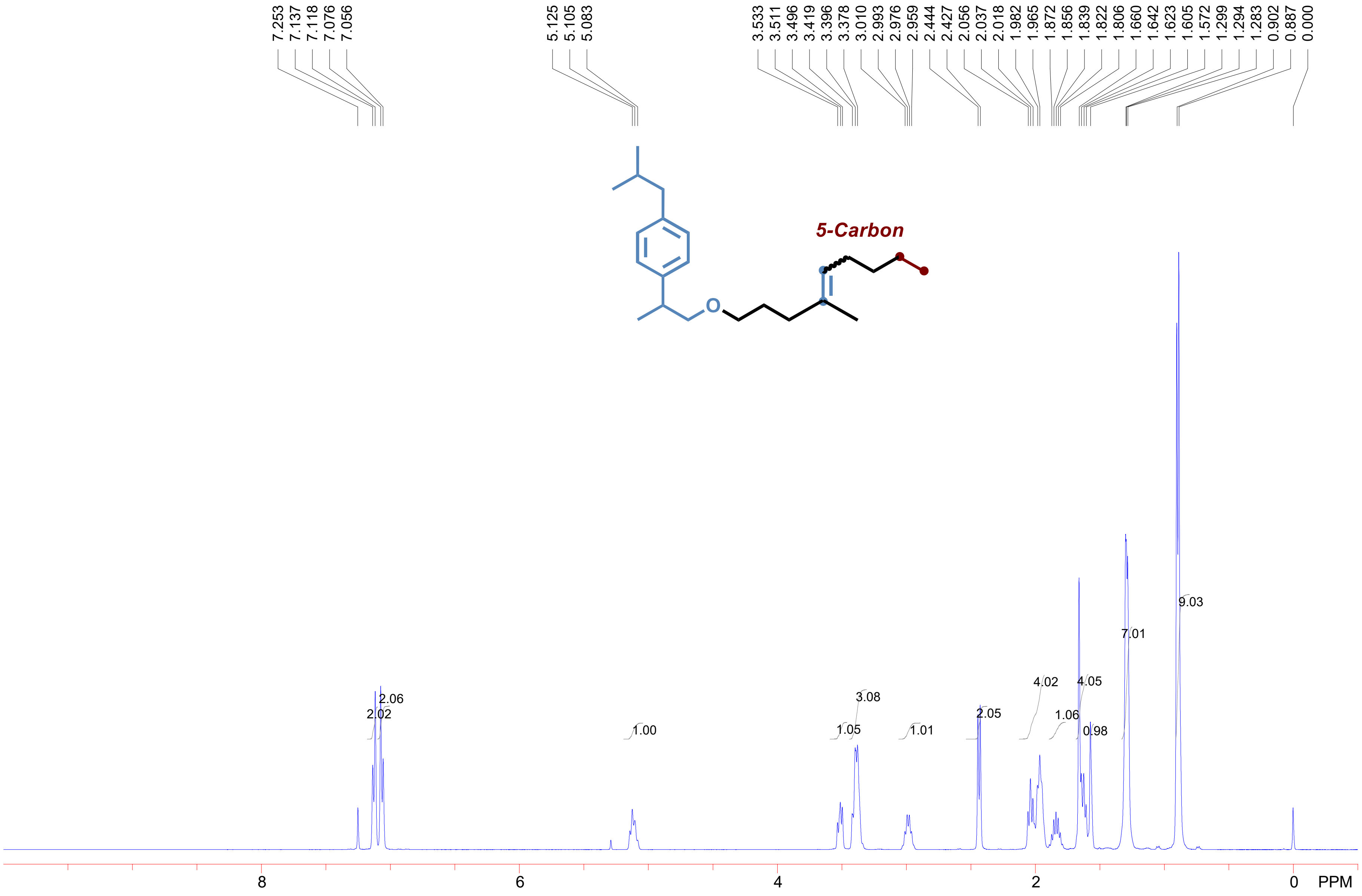

## $^{13}$C NMR-spectrum (100 MHz, $CDCl_3$) of 39a

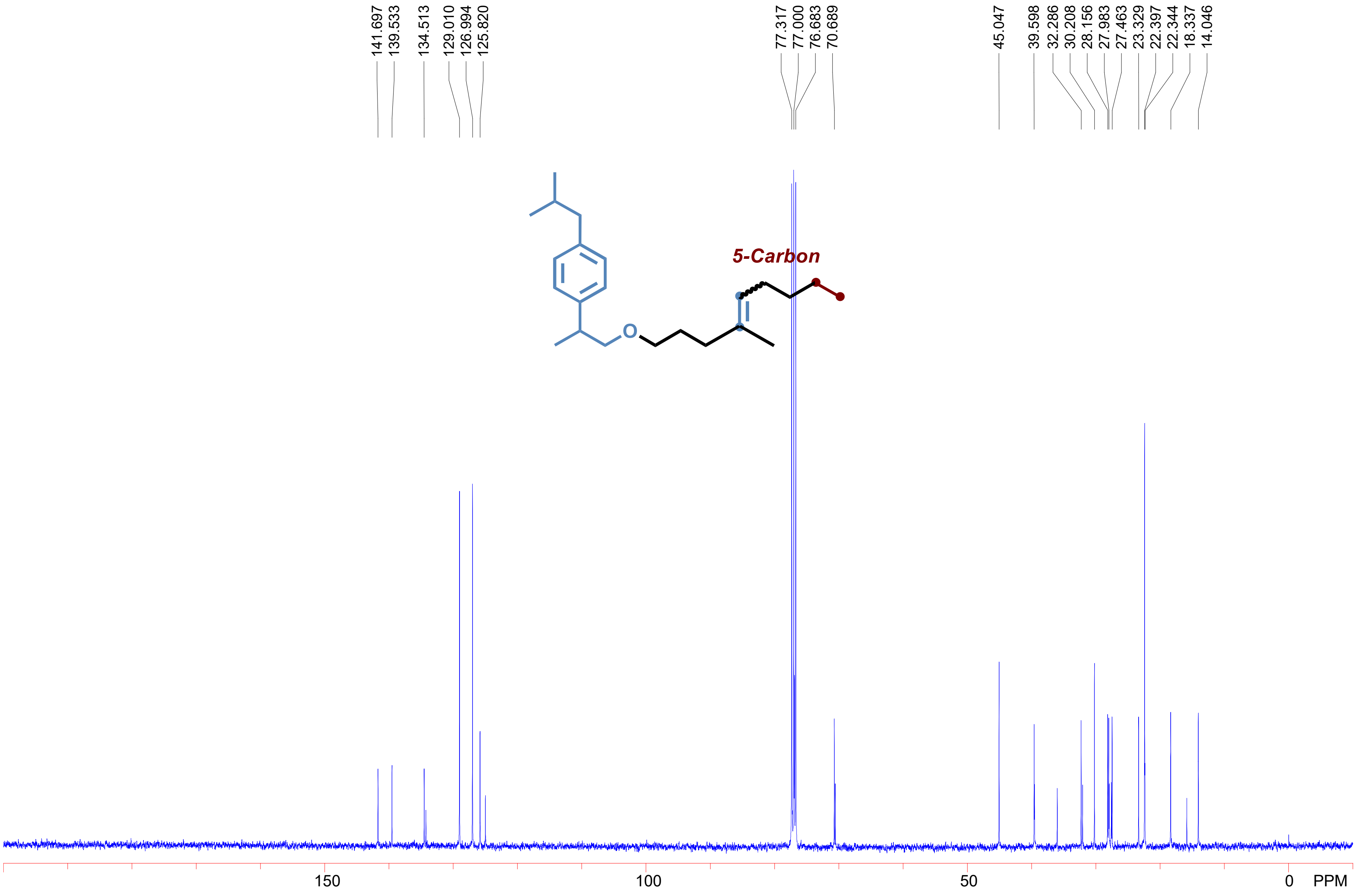

$^{1}$H NMR-spectrum (400 MHz, $CDCl_3$) of 40a

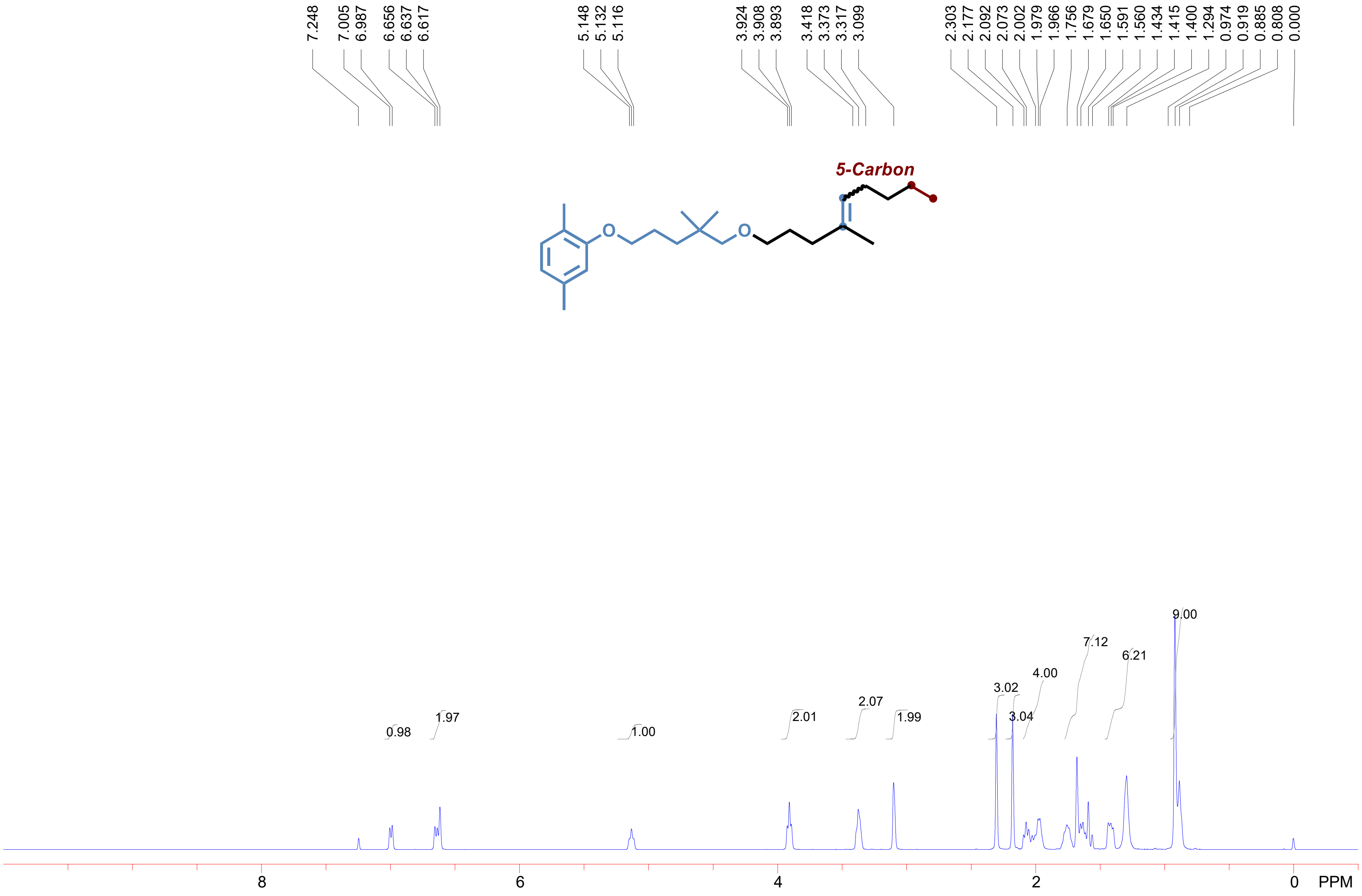

**$^{13}$C NMR-spectrum (100 MHz, $CDCl_3$) of 40a**

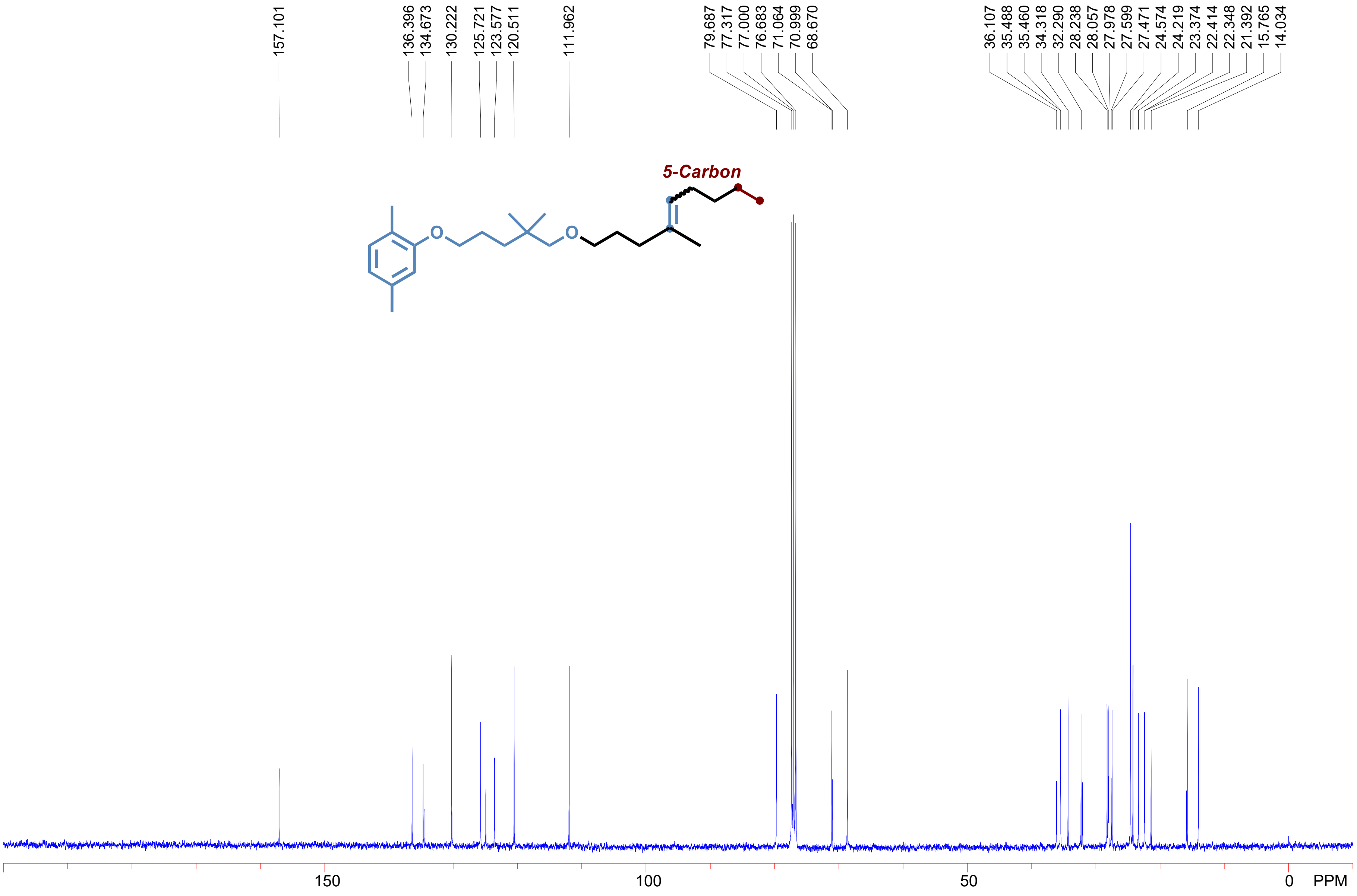

**$^{1}$H NMR-spectrum (400 MHz, CDCl3) of 41a**

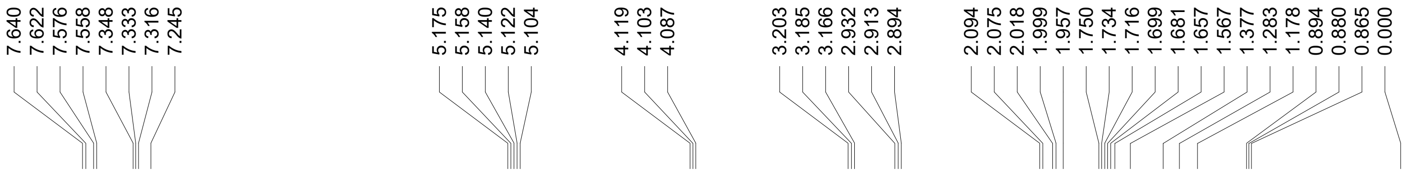


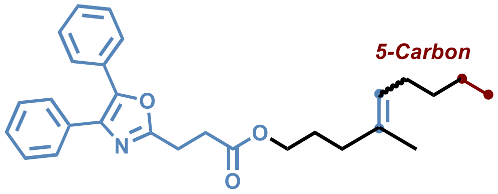


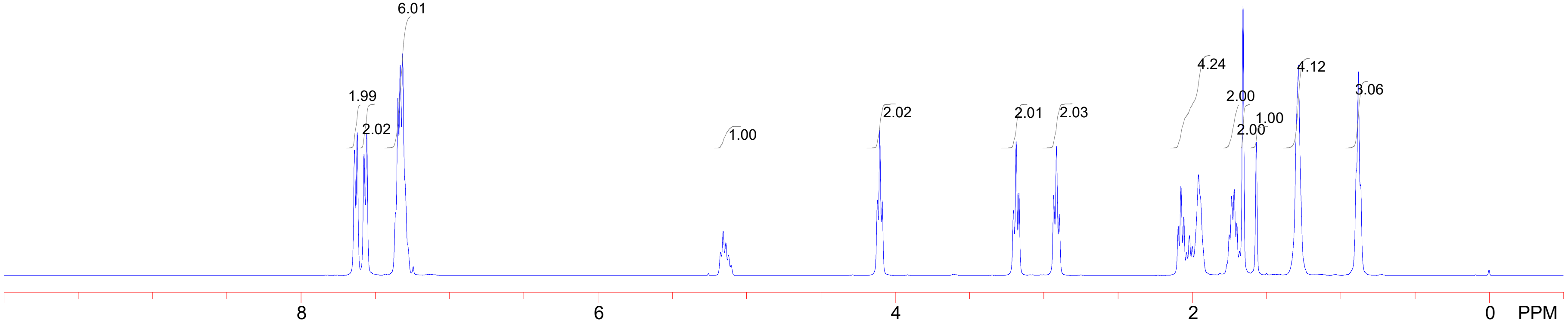

**$^{13}$C NMR-spectrum (100 MHz, $CDCl_3$) of 41a**

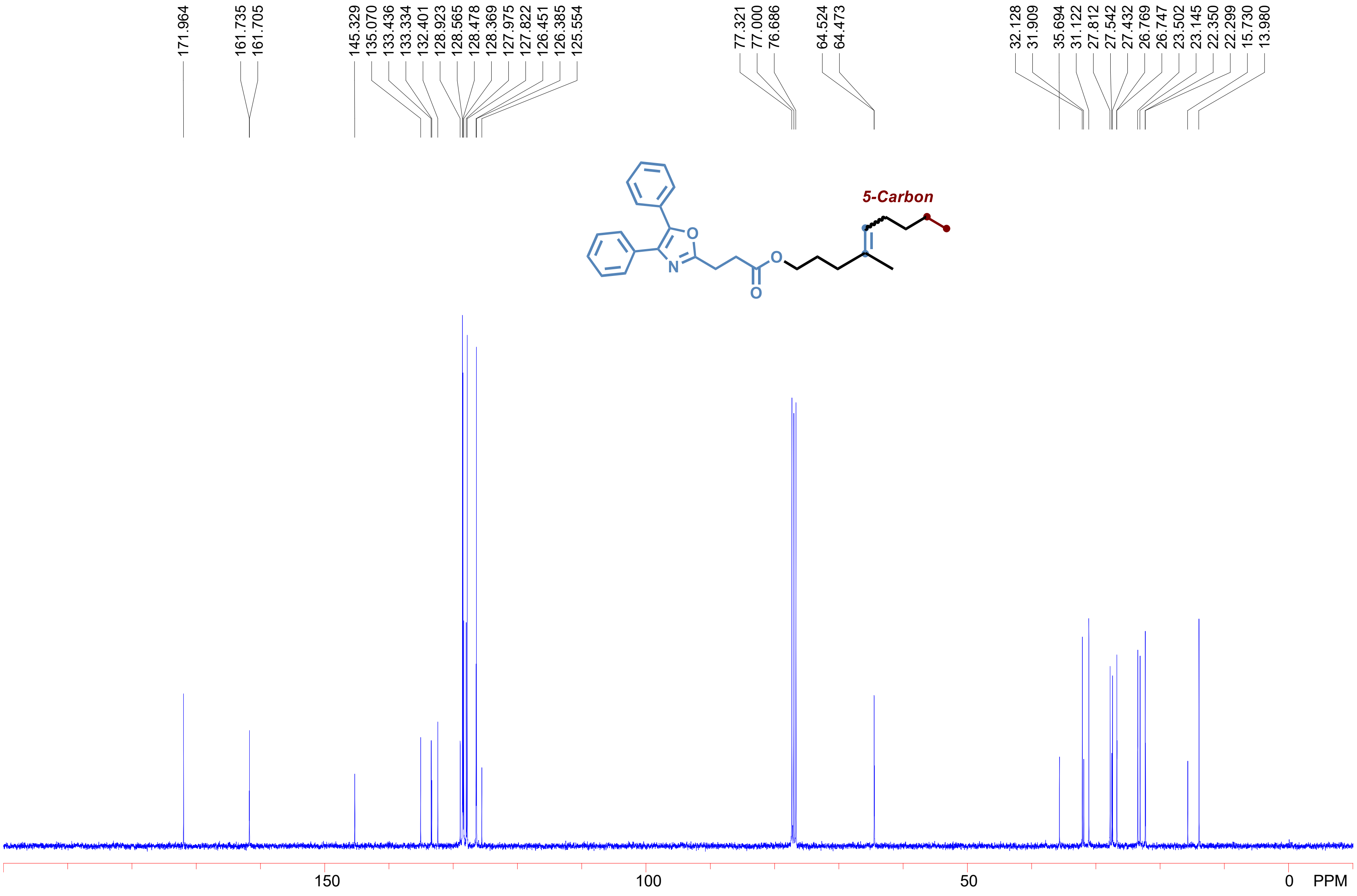

$^{1}$H NMR-spectrum (400 MHz, $CDCl_3$) of 42a

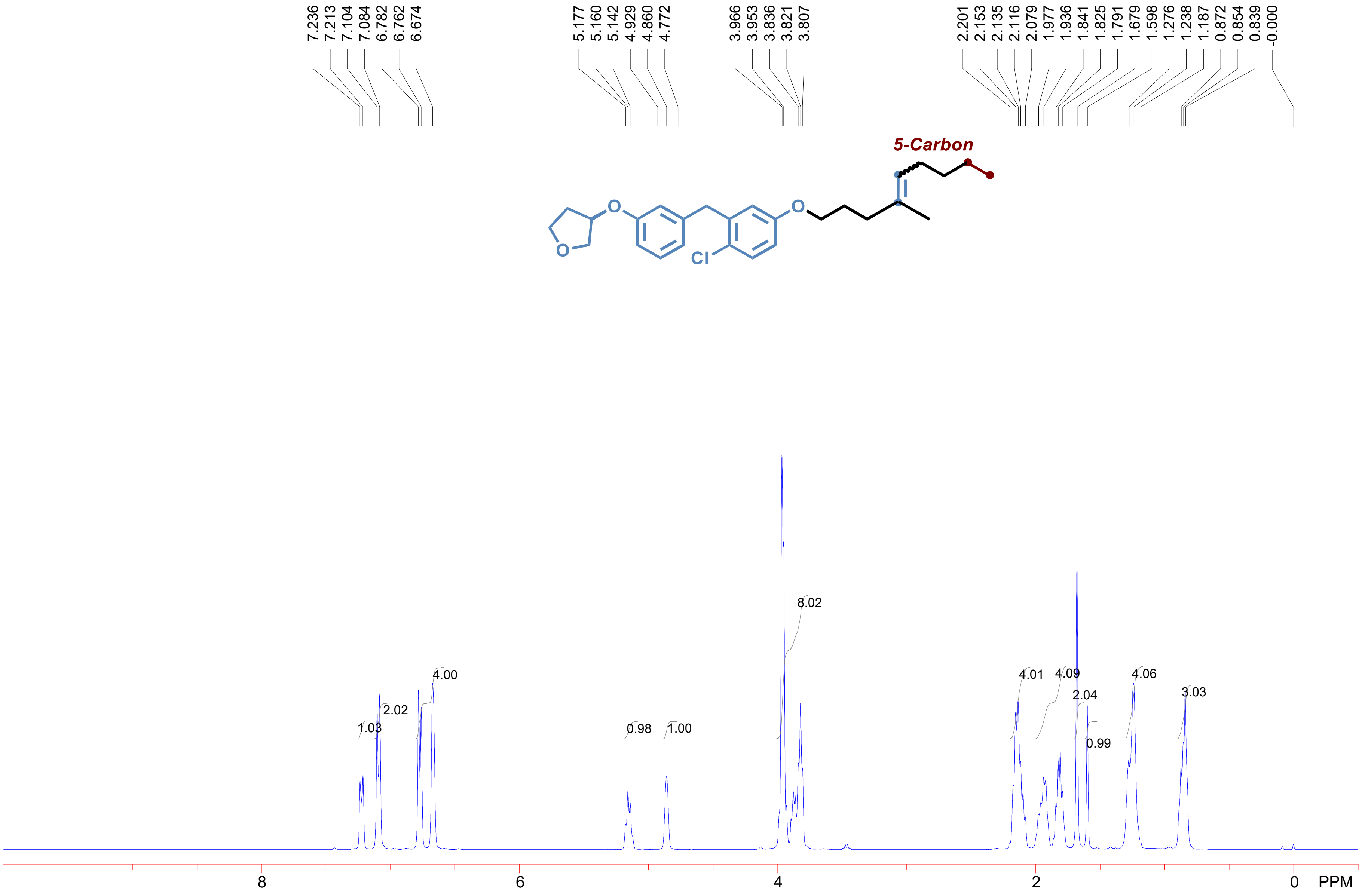

**$^{13}C$ NMR-spectrum (100 MHz, $CDCl_3$) of 42a**

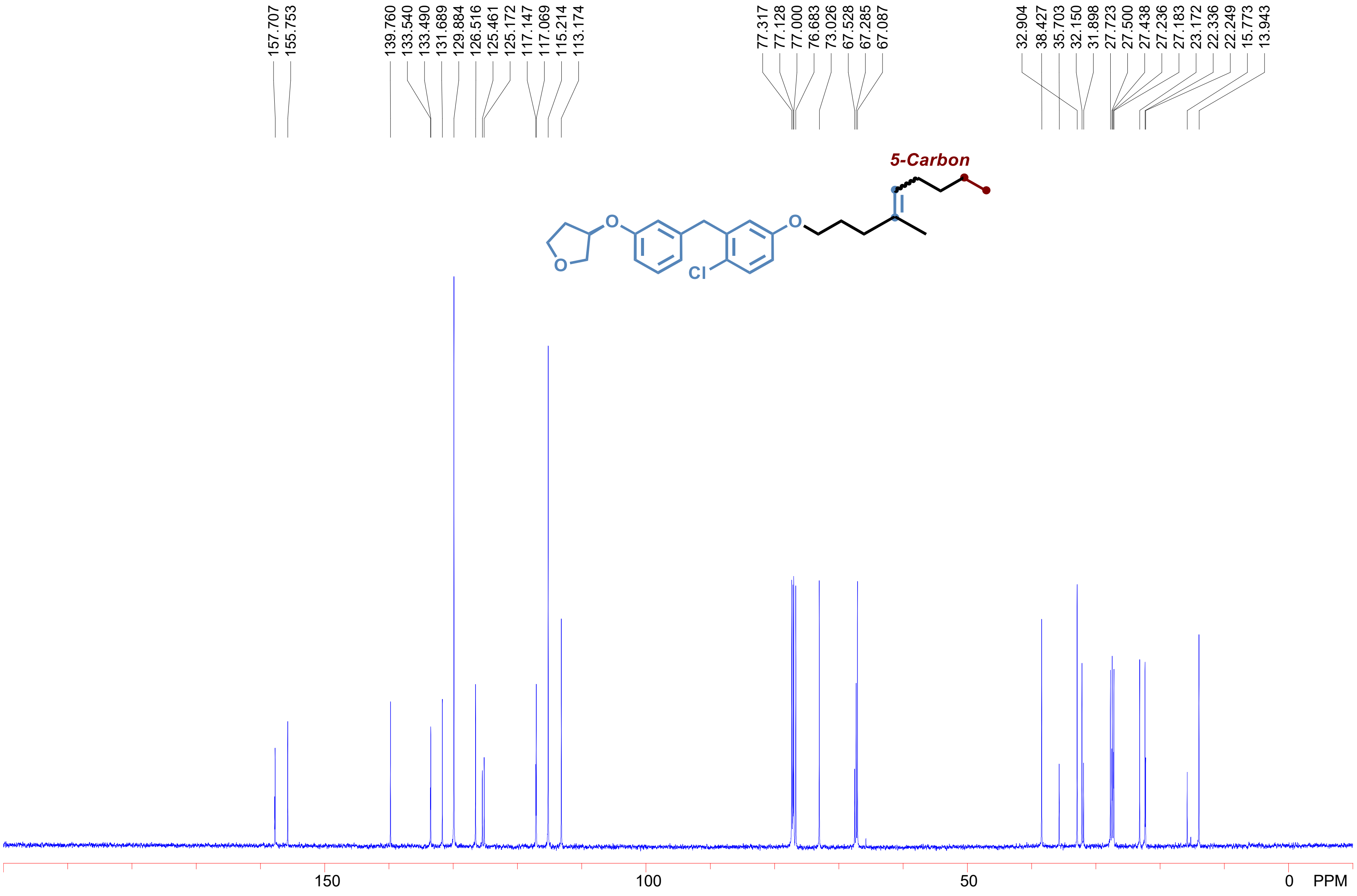

$^{1}H$ NMR-spectrum (400 MHz, $CDCl_3$) of 43a

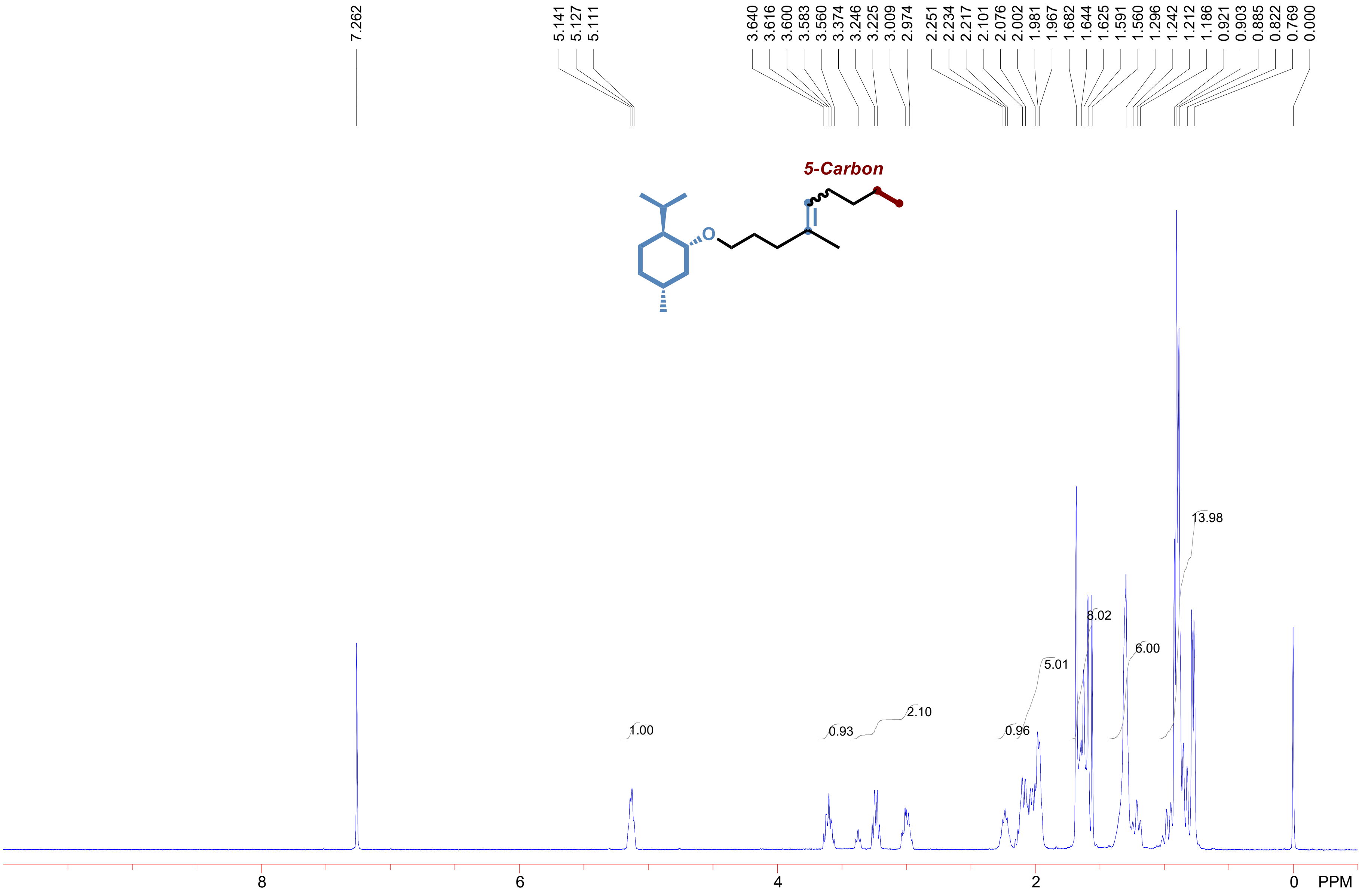

**$^{13}$C NMR-spectrum (100 MHz, CDCl₃) of 43a**

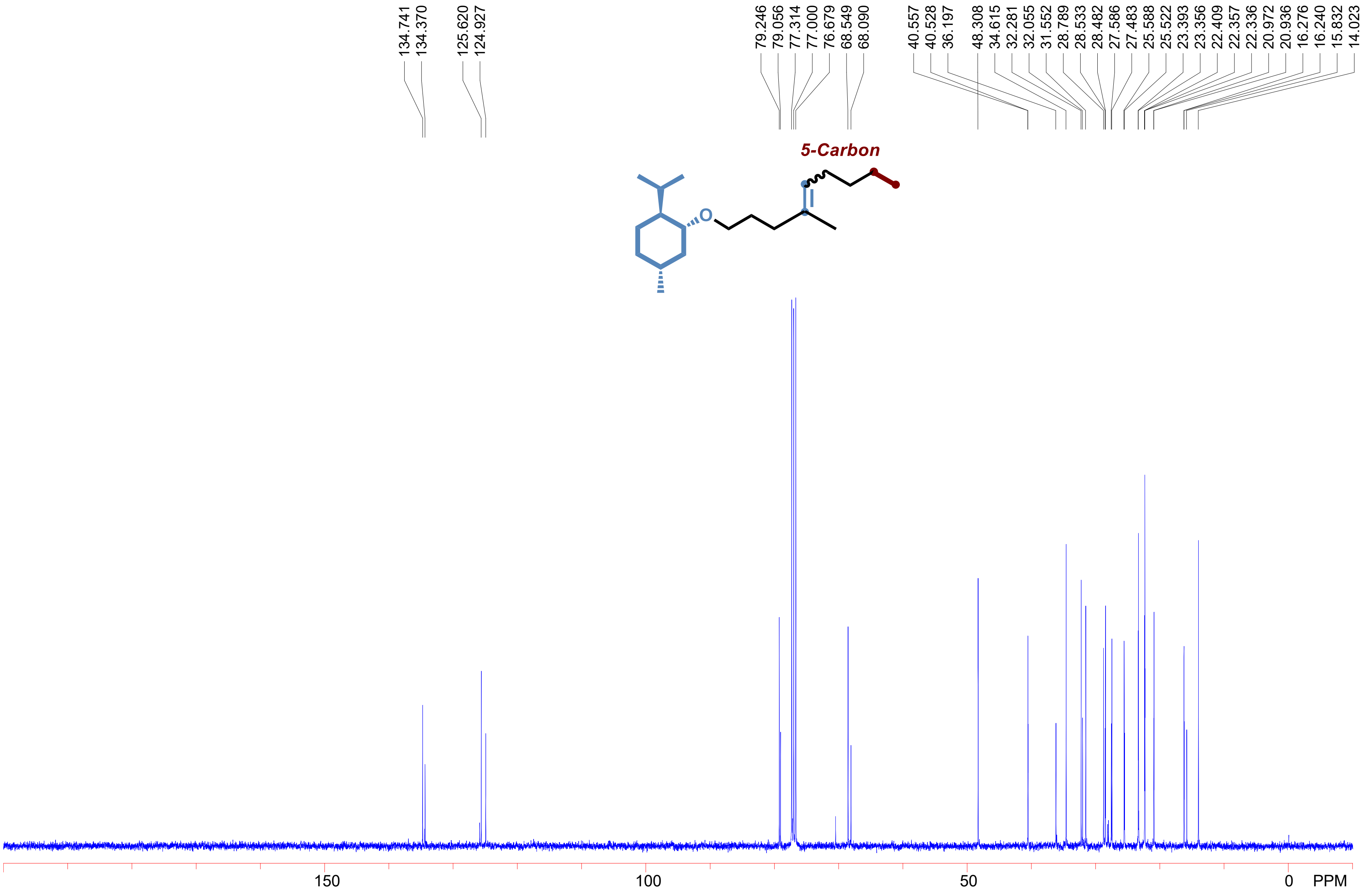

$^{1}$H NMR-spectrum (400 MHz, $CDCl_3$) of 44a

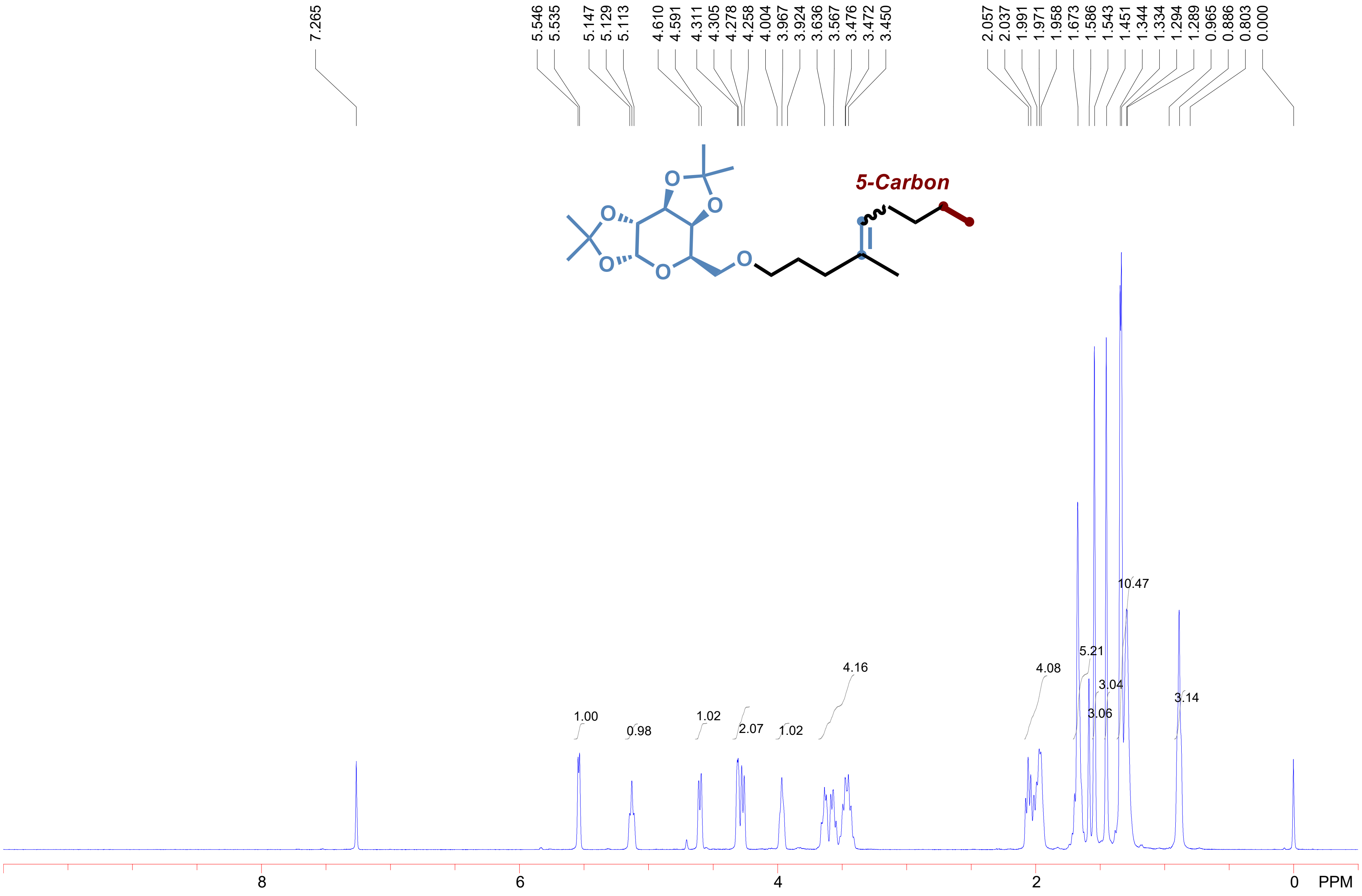

**$^{13}$C NMR-spectrum (100 MHz, CDCl₃) of 44a**

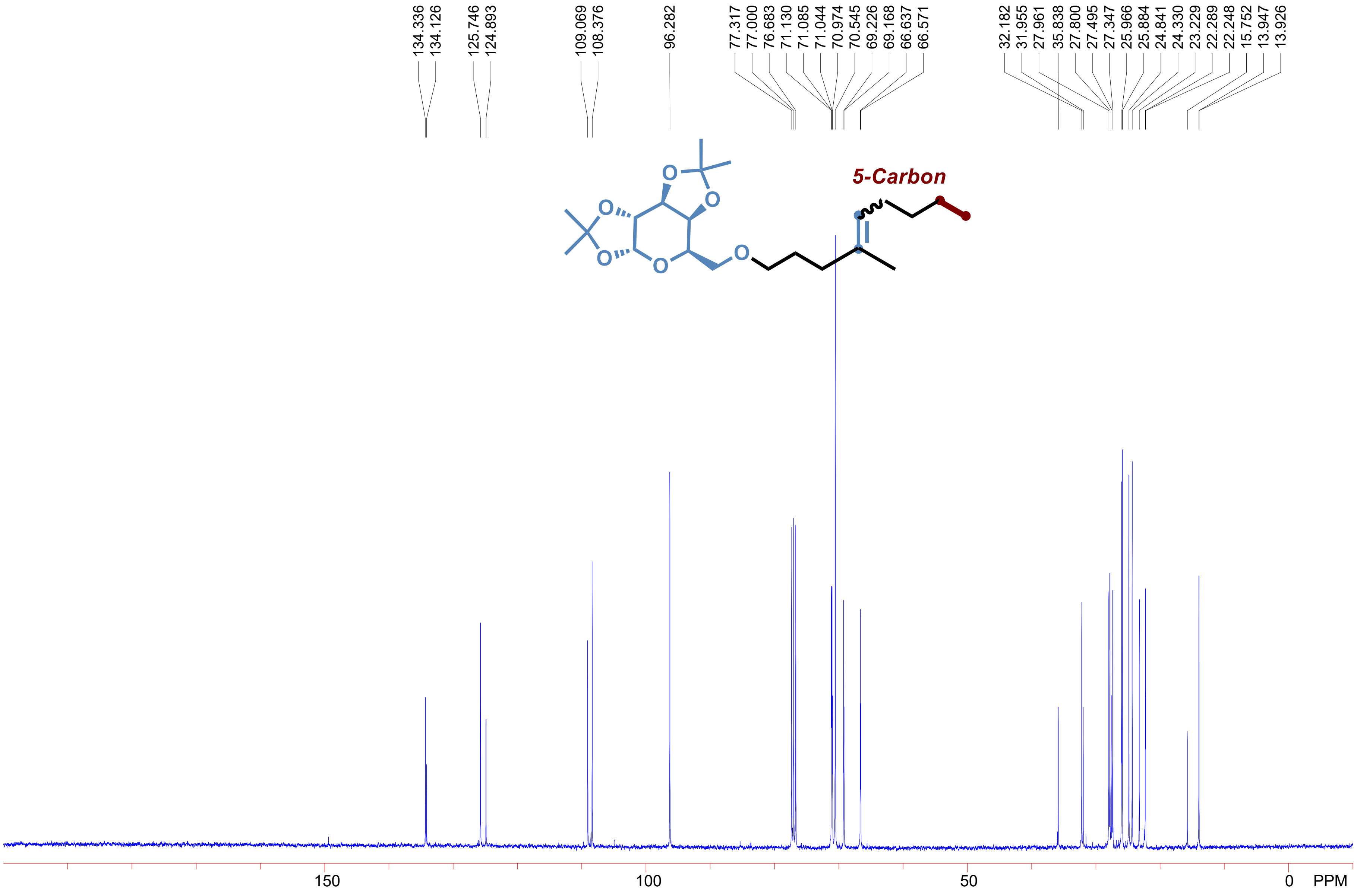

$^{1}$H NMR-spectrum (400 MHz, CDCl3) of 45a

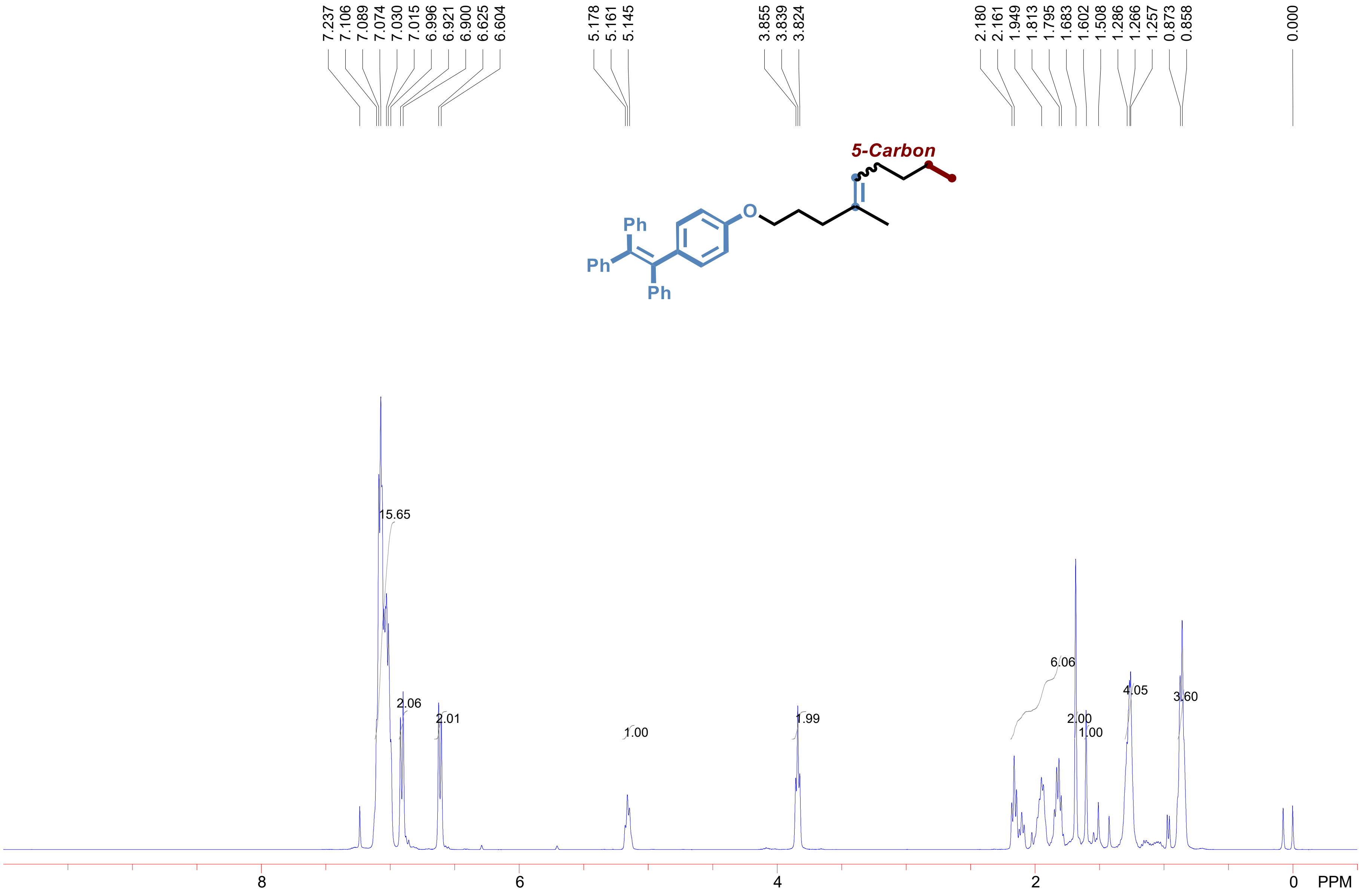

**$^{13}$C NMR-spectrum (100 MHz, $CDCl_3$) of 45a**

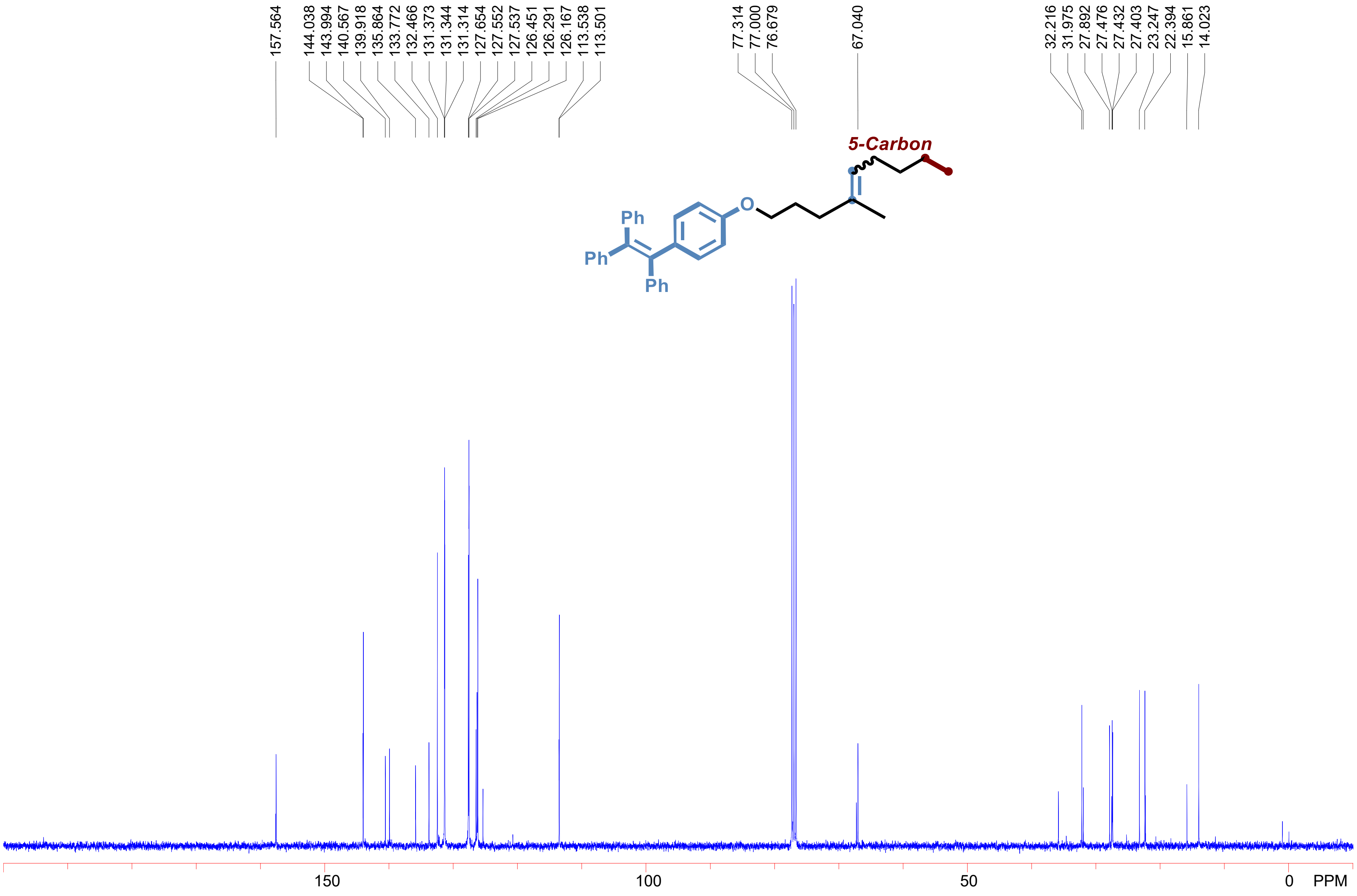

**$^{1}$H NMR-spectrum (400 MHz, CDCl3) of 52a**

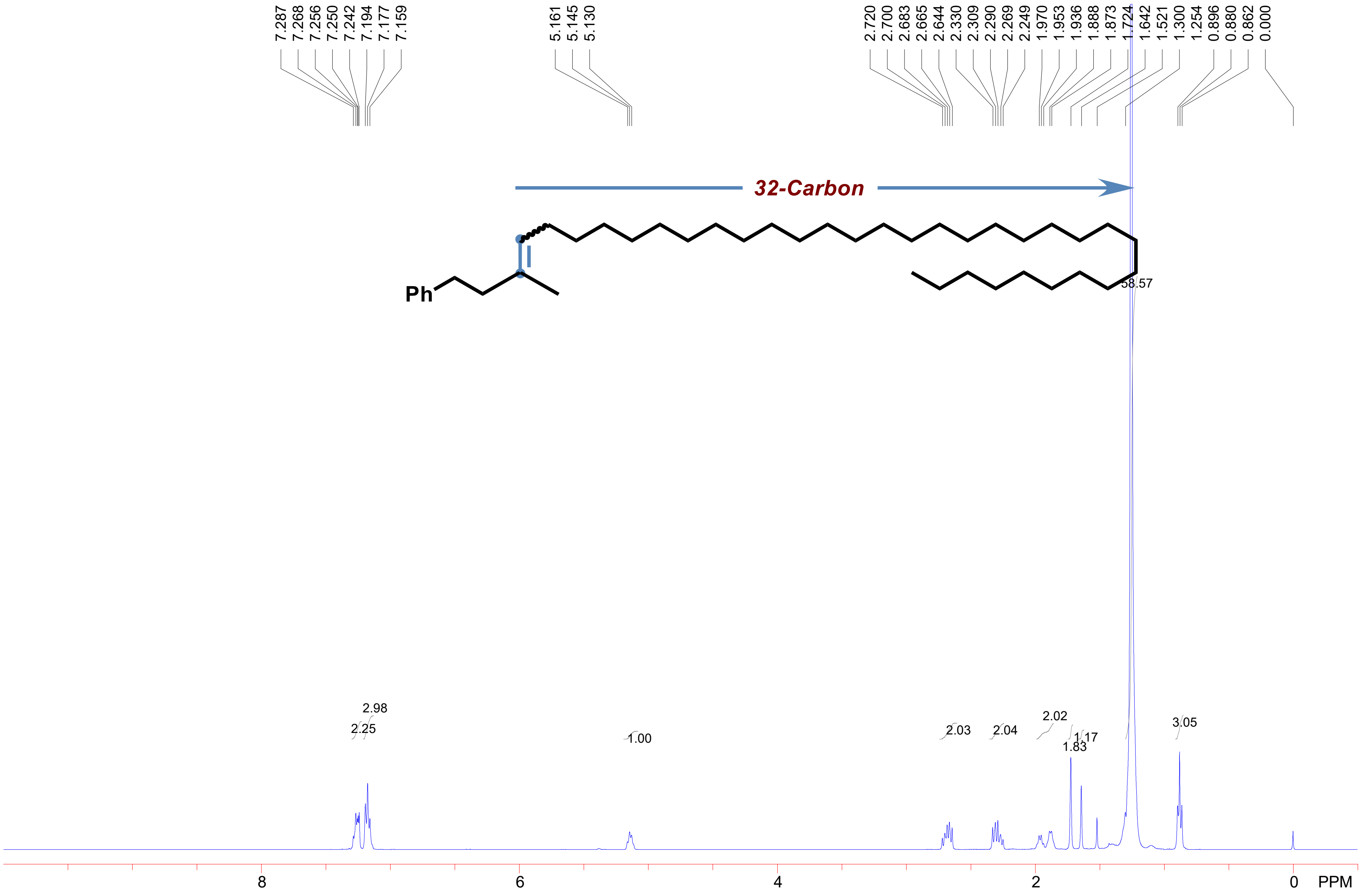

**$^{13}C$ NMR-spectrum (100 MHz, $CDCl_3$) of 52a**

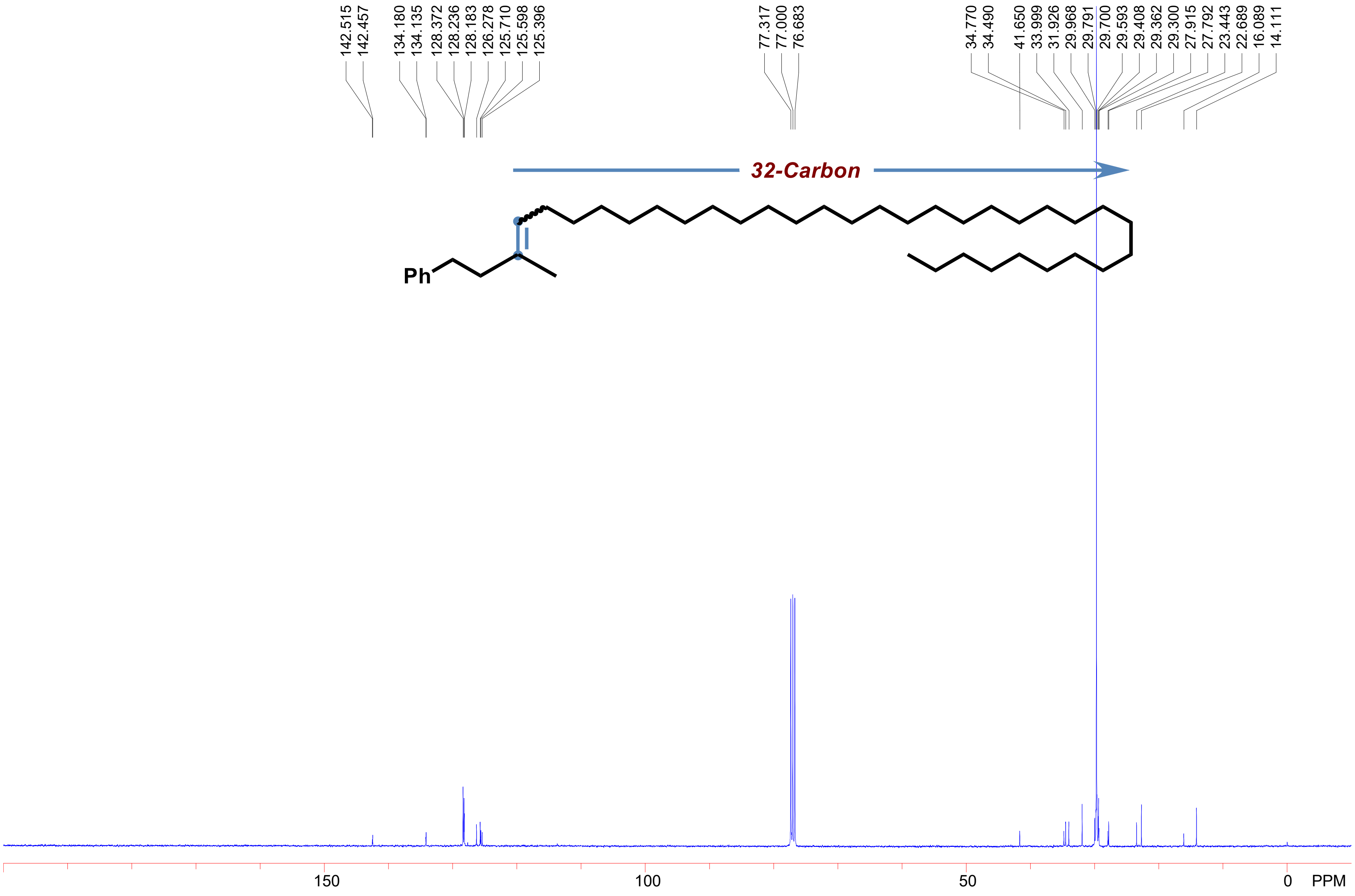

**$^{1}$H NMR-spectrum (400 MHz, $CDCl_3$) of 54a**

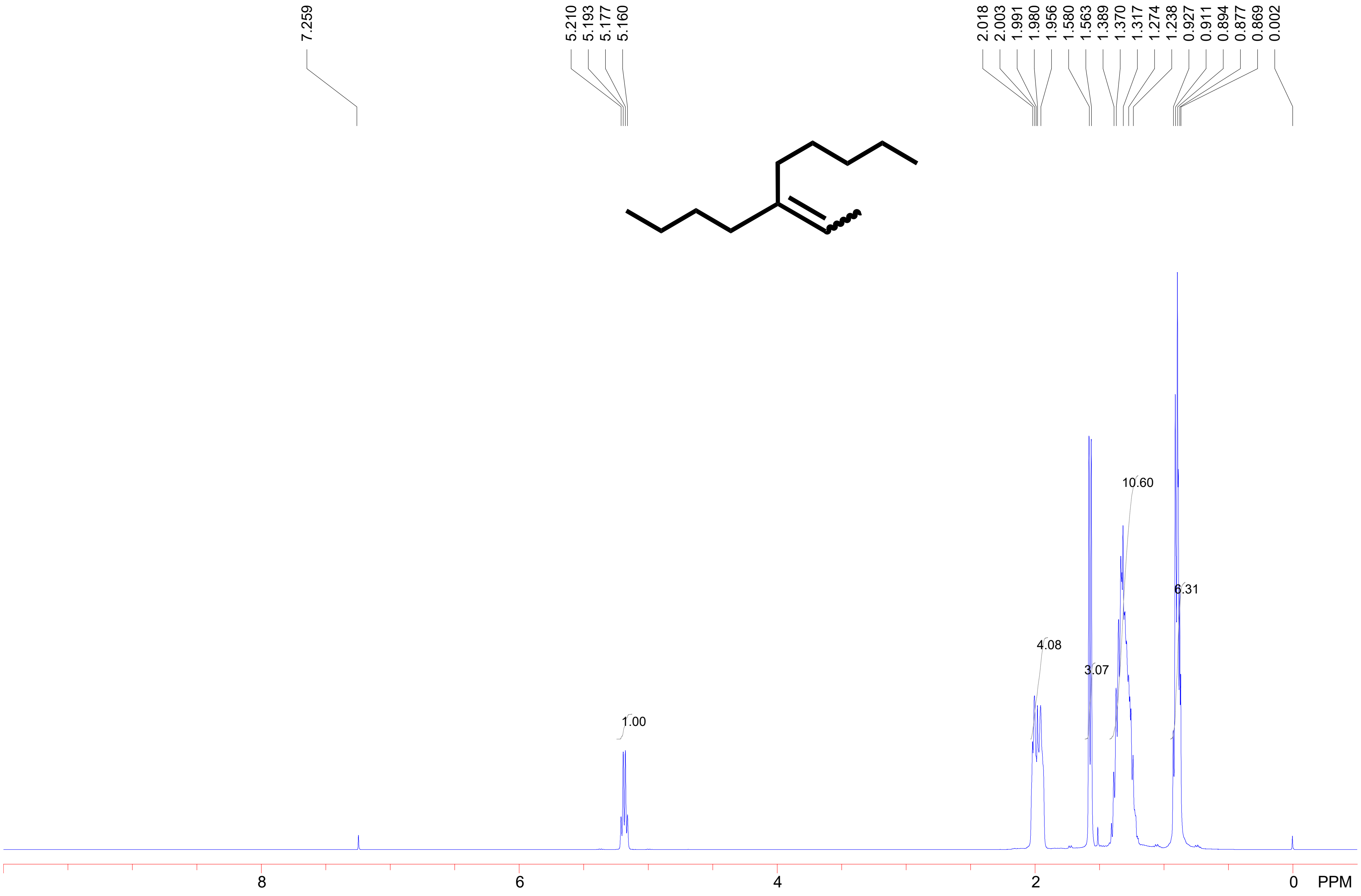

$^{13}$C NMR-spectrum (100 MHz, CDCl$_3$) of 54a

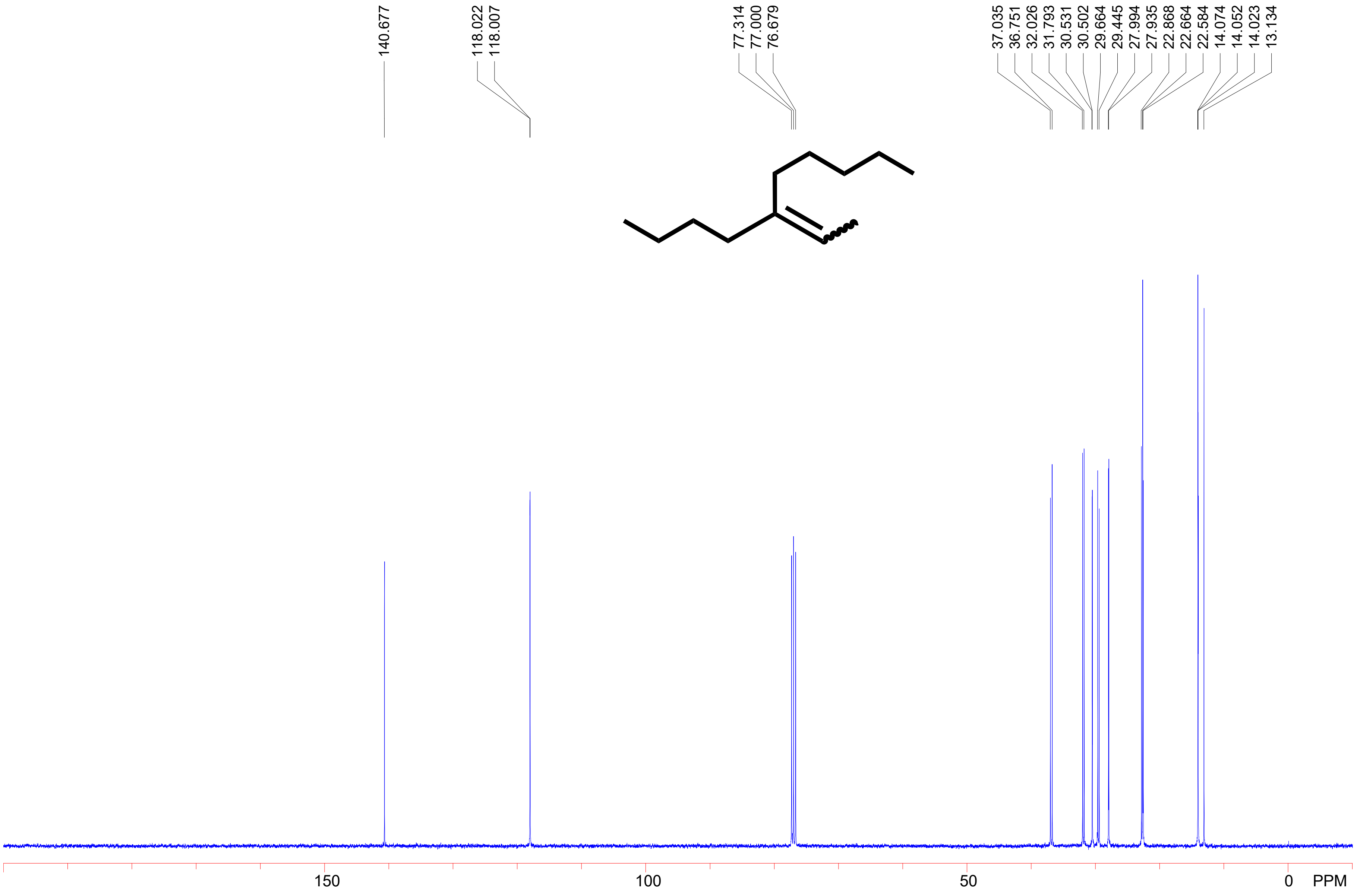

**$^{1}$H NMR-spectrum (400 MHz, $CDCl_3$) of 55a**

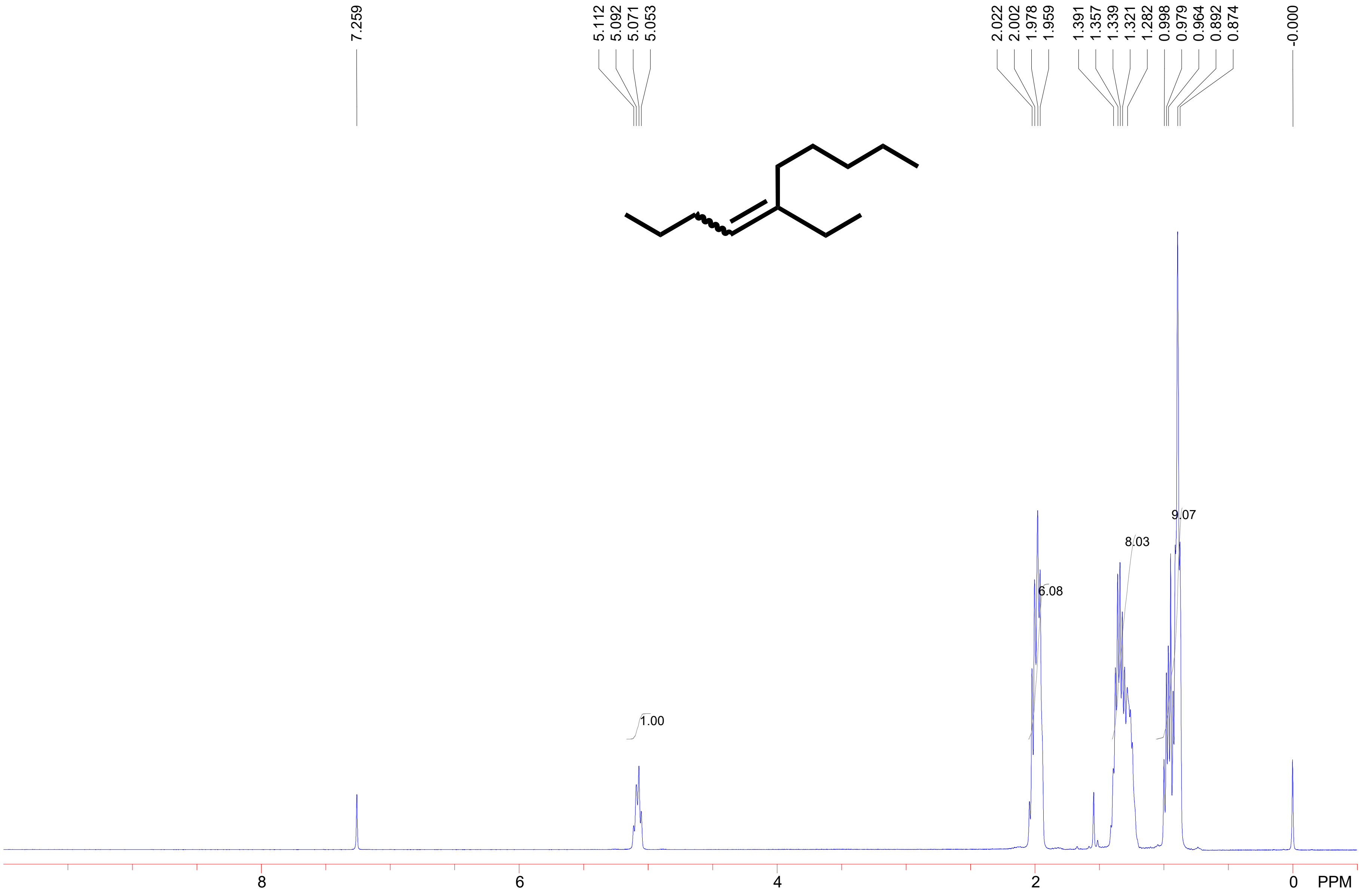

**$^{13}$C NMR-spectrum (100 MHz, $CDCl_3$) of 55a**

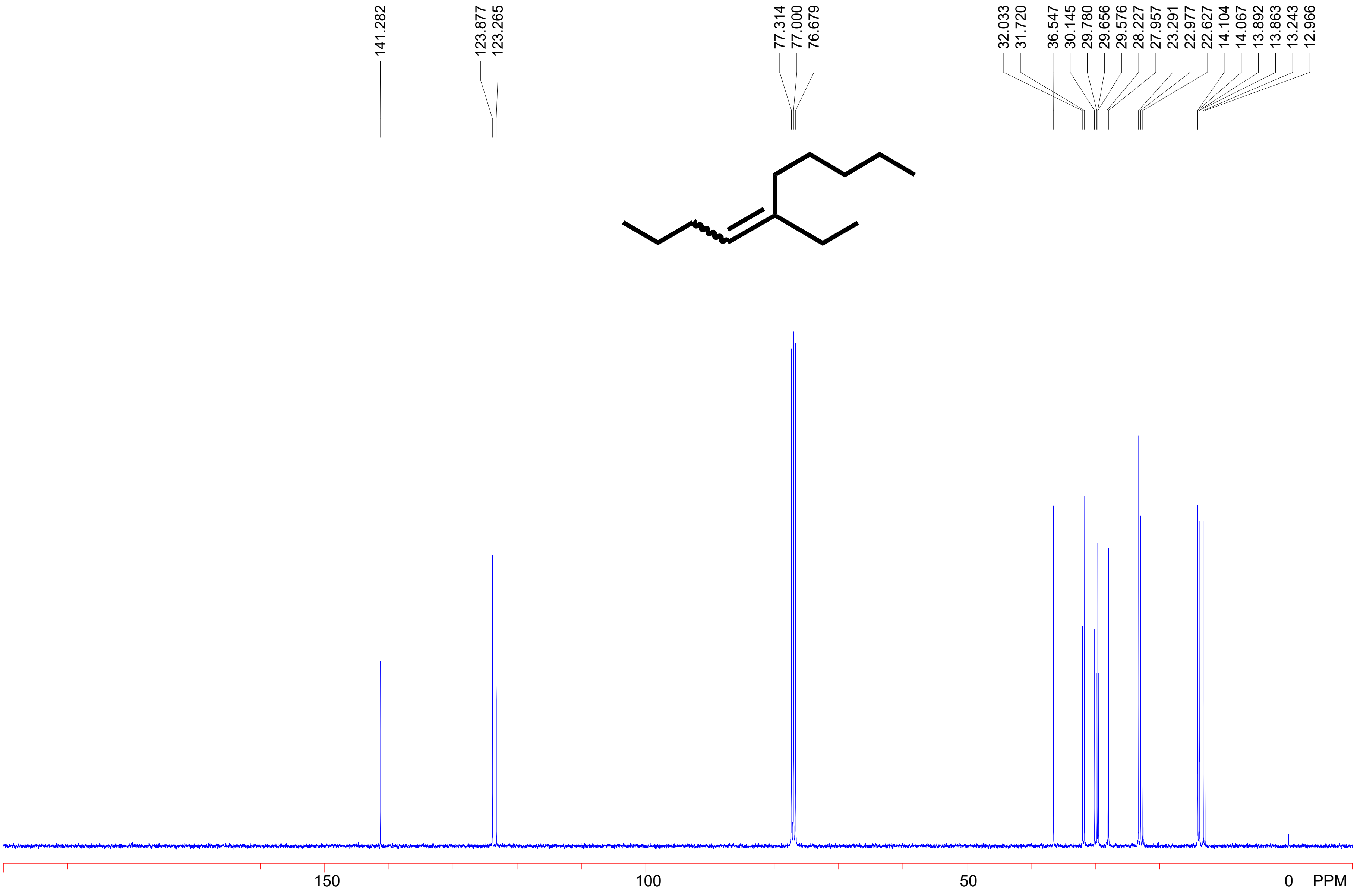

$^{1}$H NMR-spectrum (400 MHz, CDCl3) of 56a

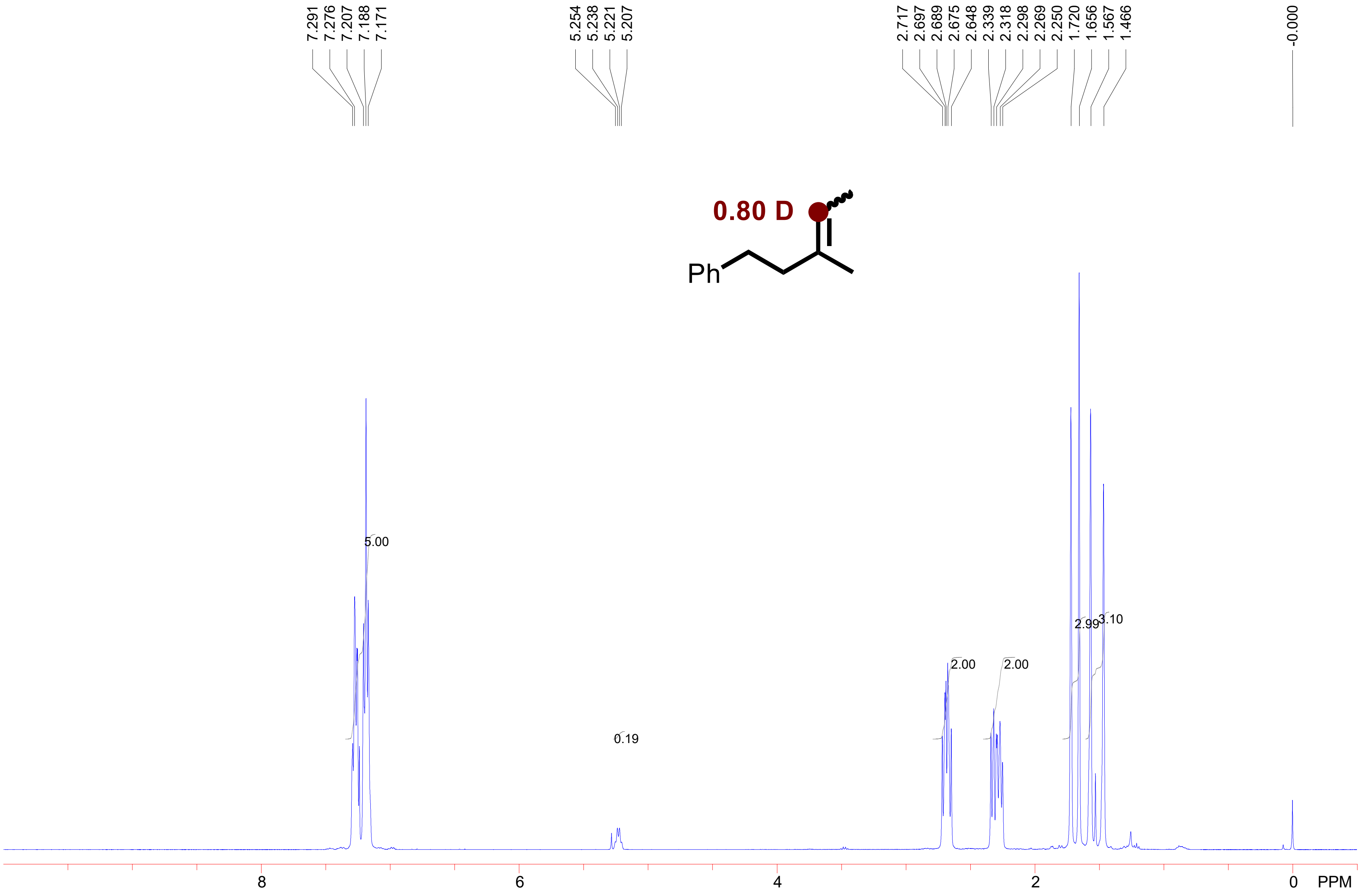

$^{1}$H NMR-spectrum (400 MHz, $CDCl_3$) of HBdan

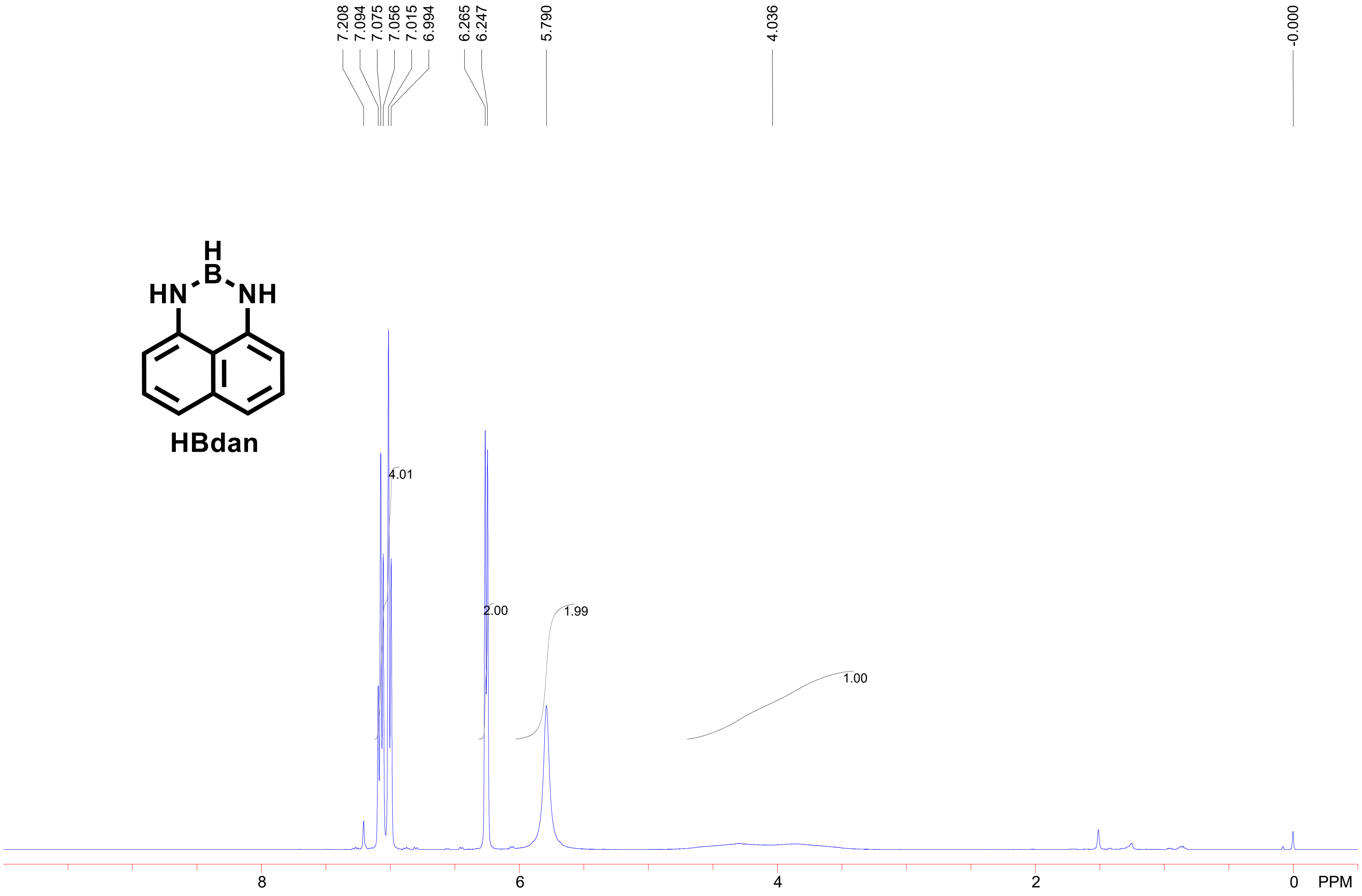

**$^1$H NMR-spectrum (400 MHz, $CDCl_3$) of 1**

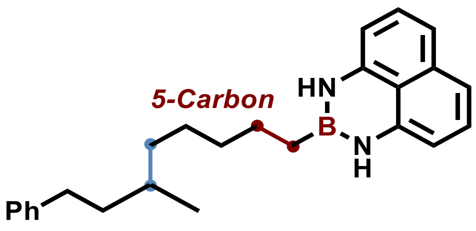


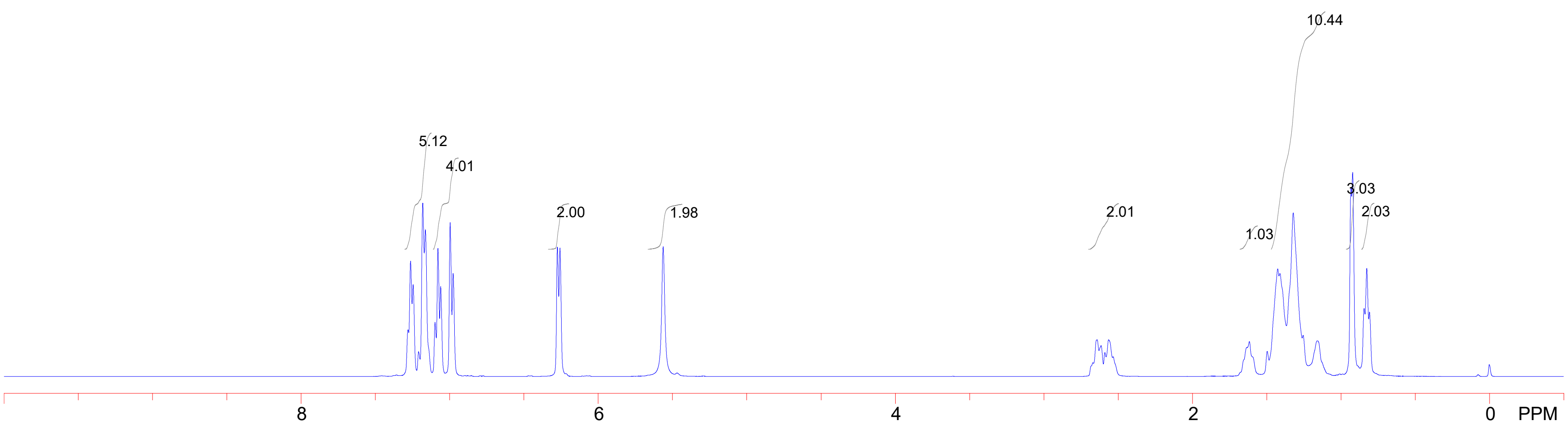

**$^{13}$C NMR-spectrum (100 MHz, $CDCl_3$) of 1**

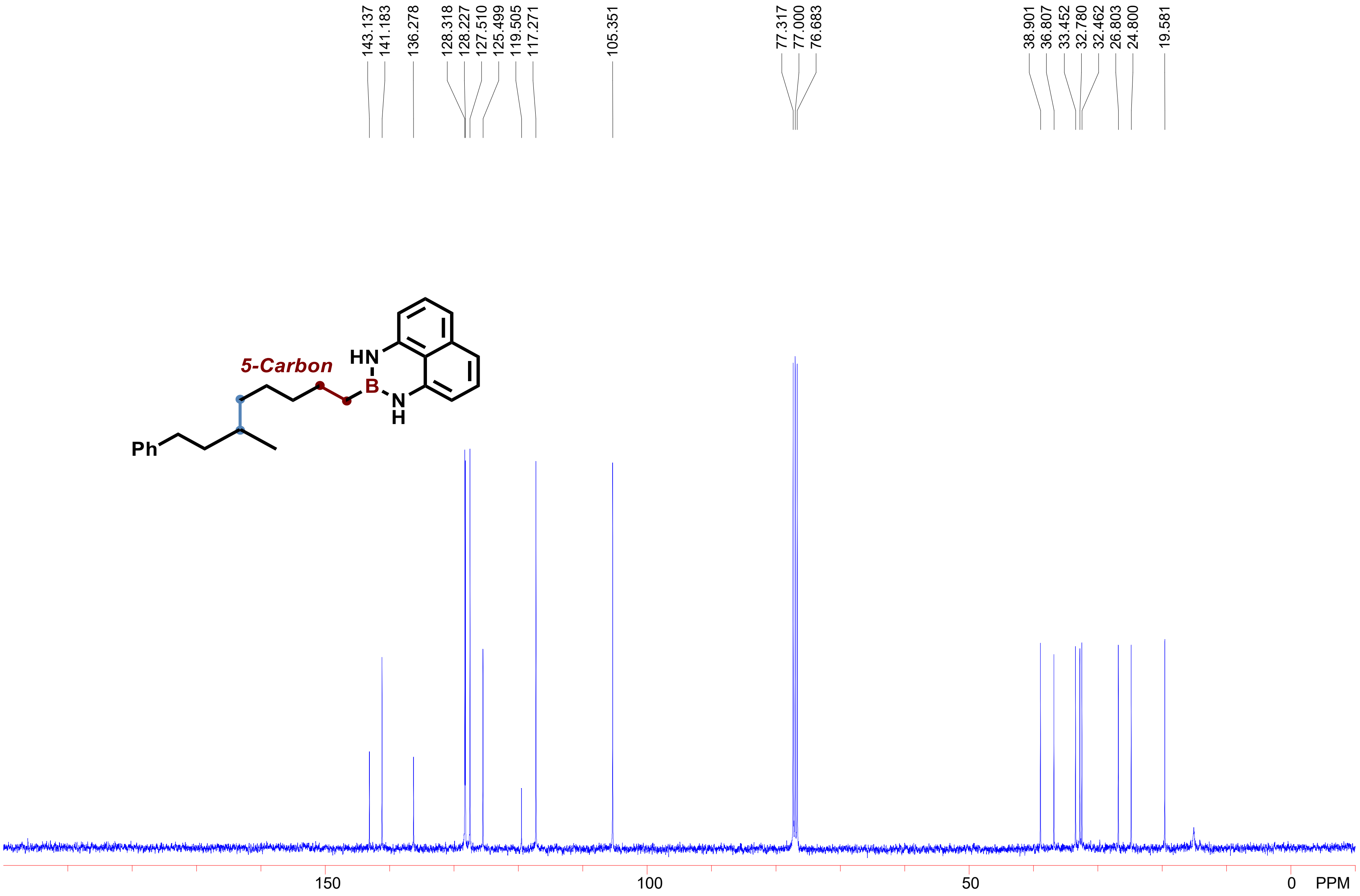

$^{1}$H NMR-spectrum (400 MHz, $CDCl_3$) of 5

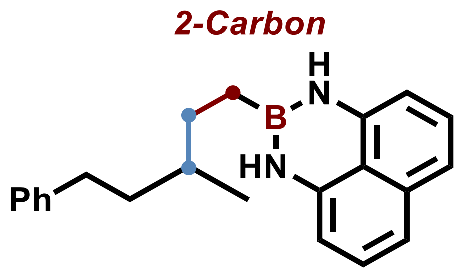


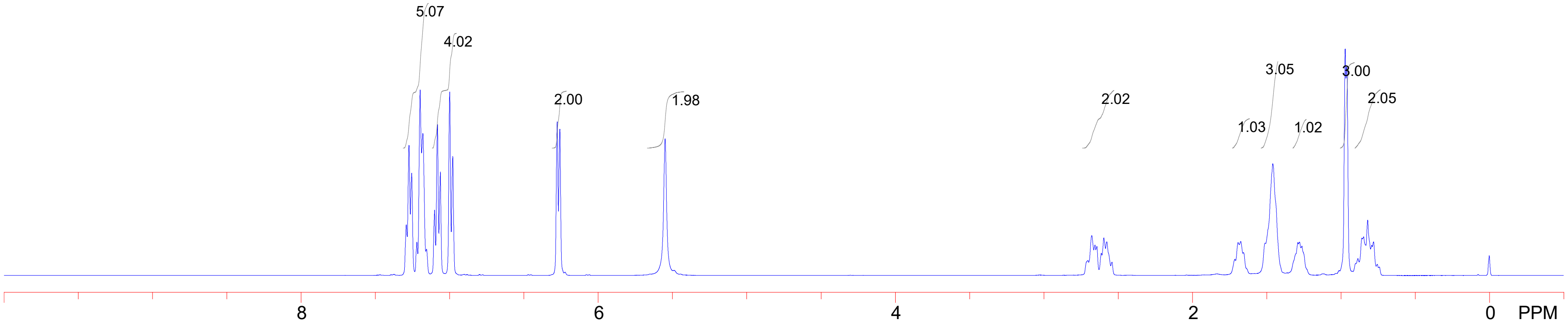

$^{13}C$ NMR-spectrum (100 MHz, $CDCl_3$) of 5

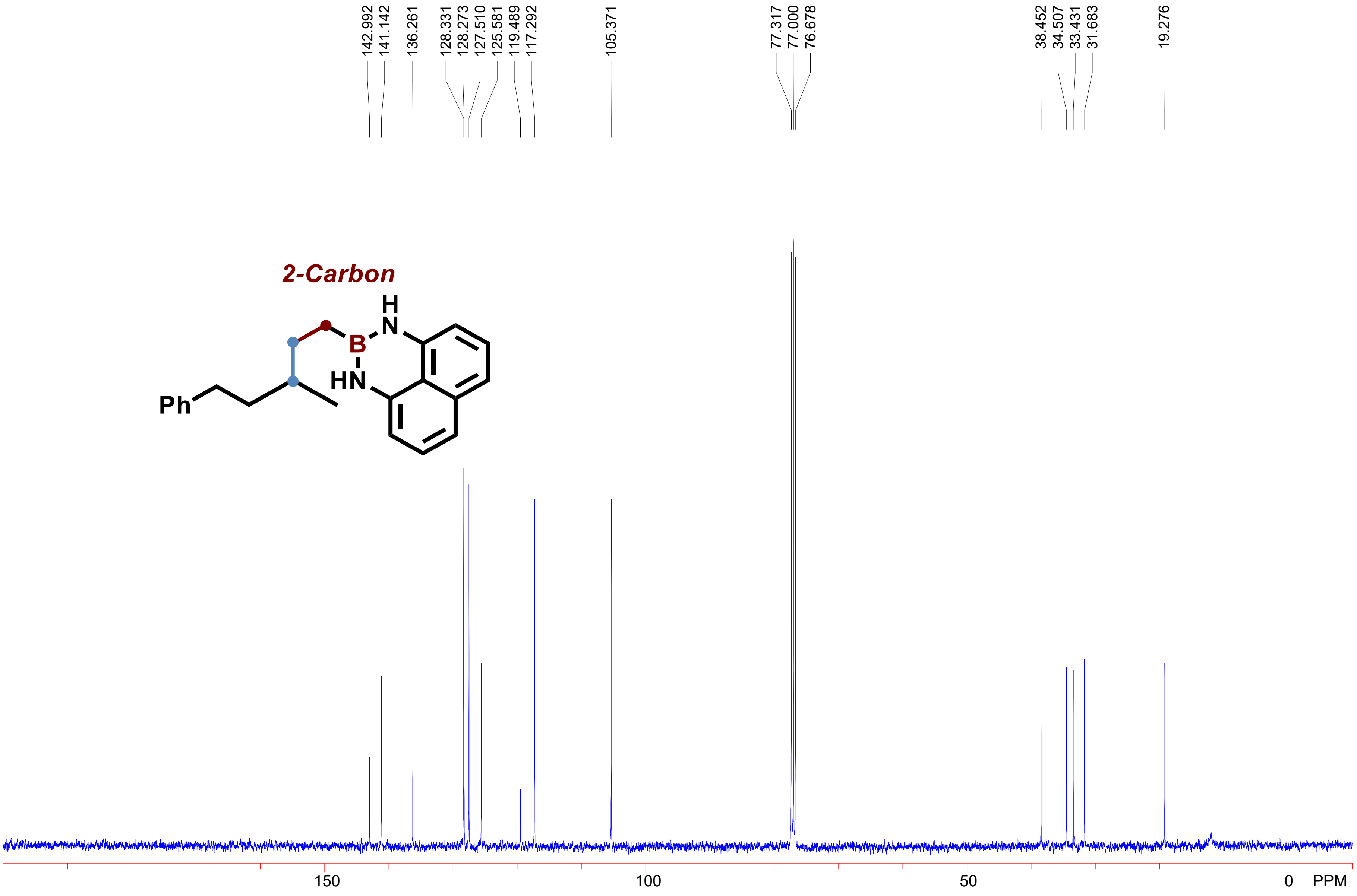

**$^{1}$H NMR-spectrum (400 MHz, $CDCl_3$) of 6**

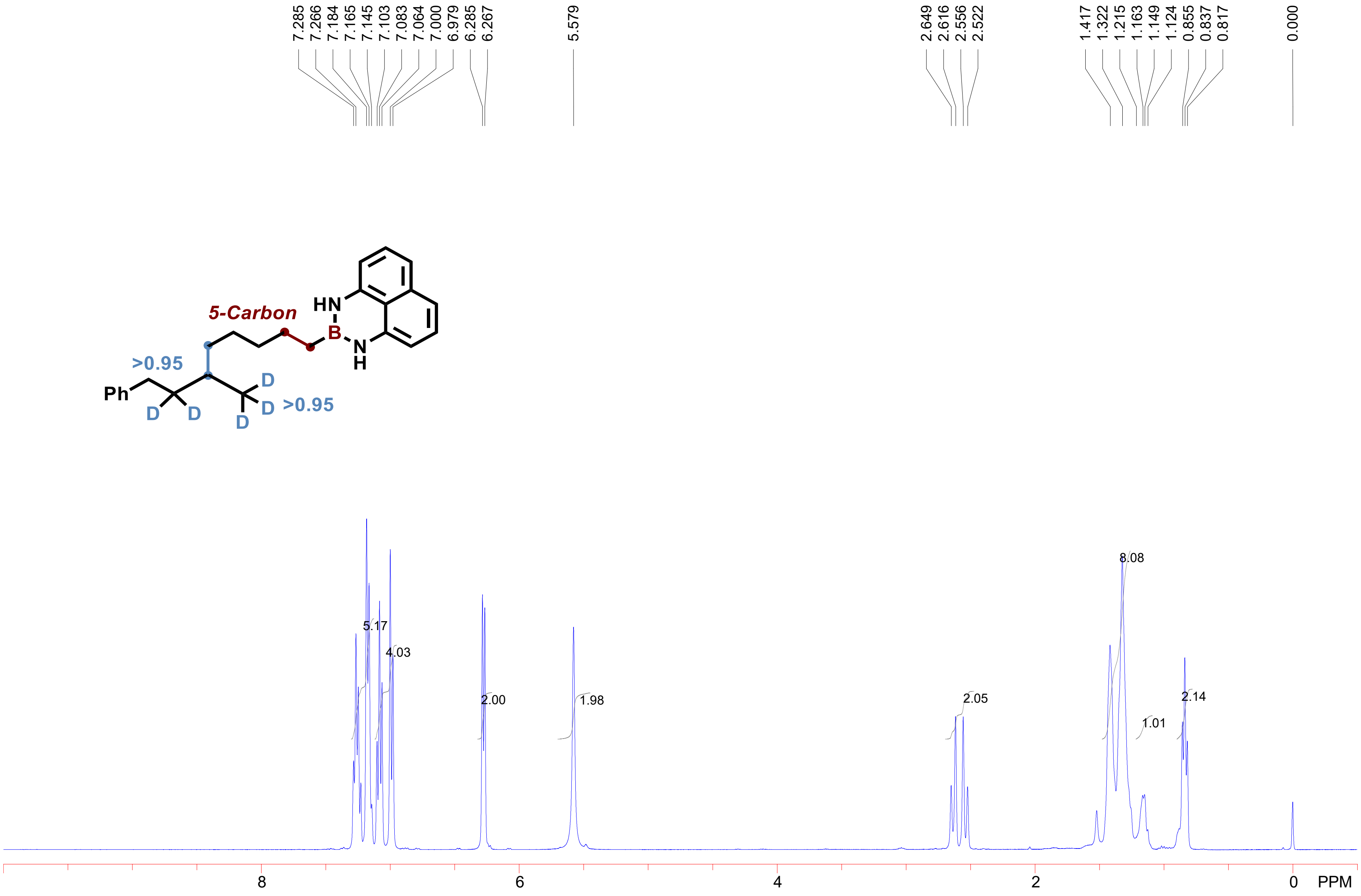

**$^2$H NMR-spectrum (92 MHz, $CDCl_3$) of 6**

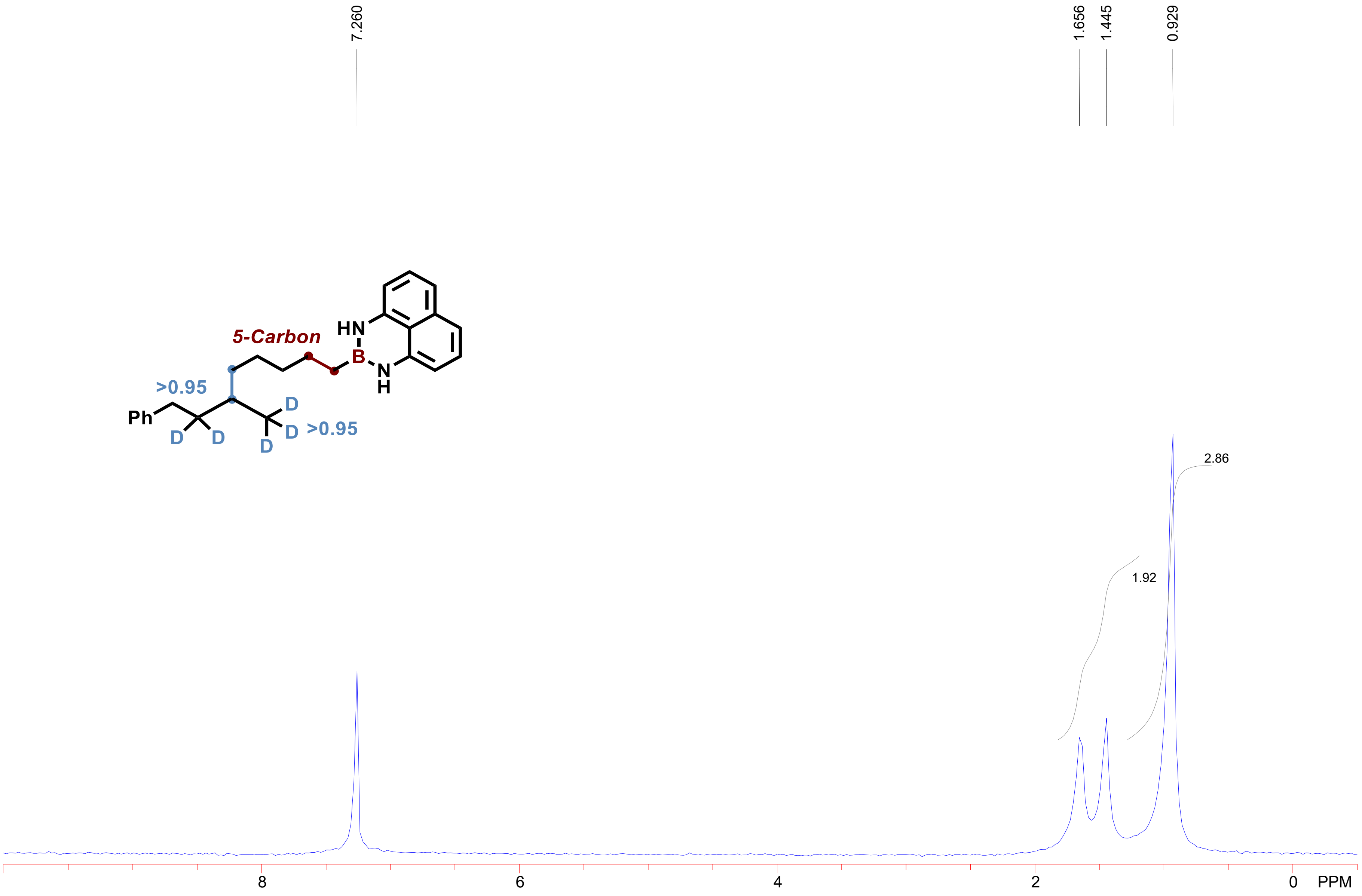

**$^{13}$C NMR-spectrum (100 MHz, CDCl3) of 6**

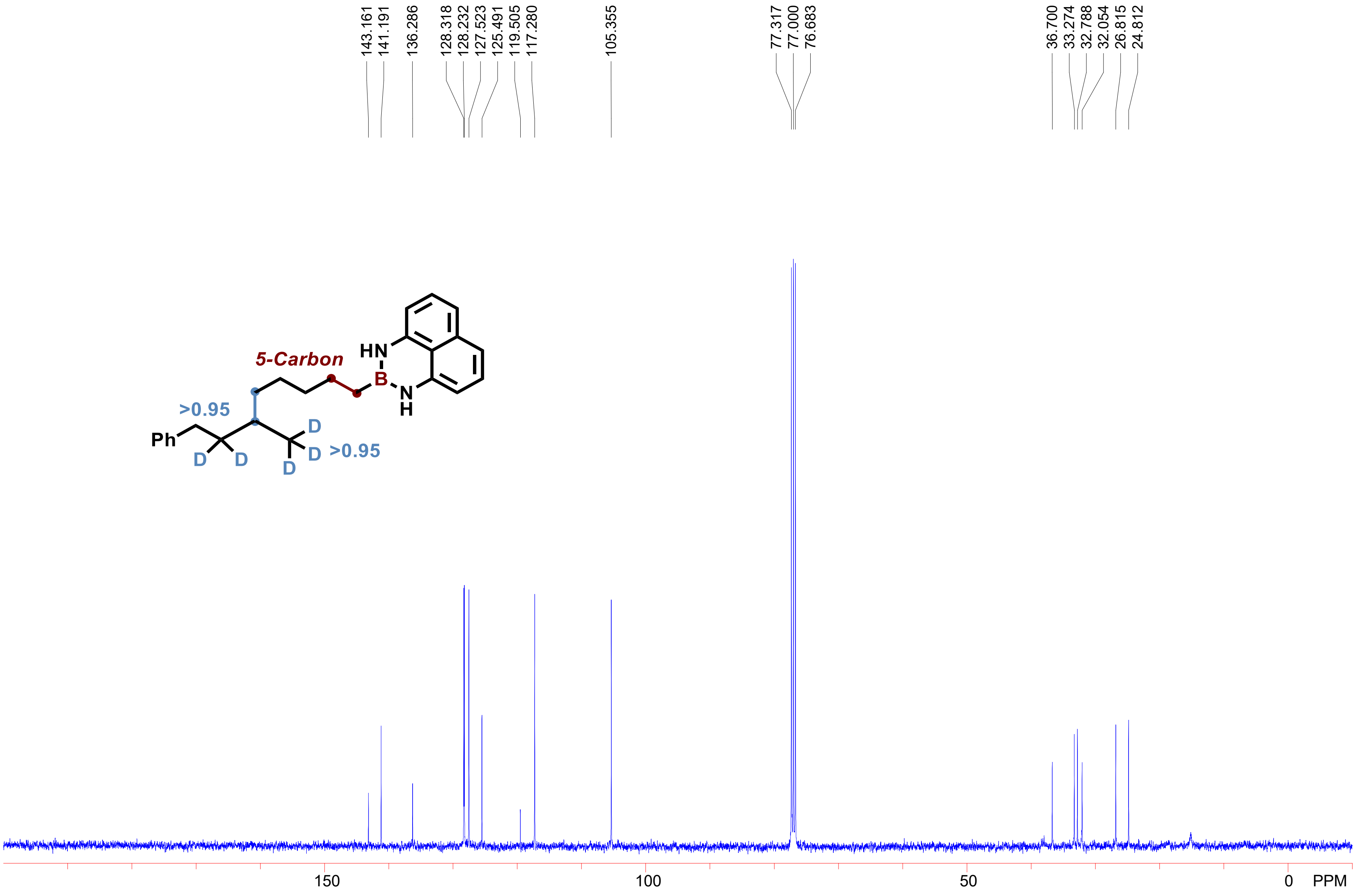

$^{1}H$ NMR-spectrum (400 MHz, $CDCl_3$) of 7

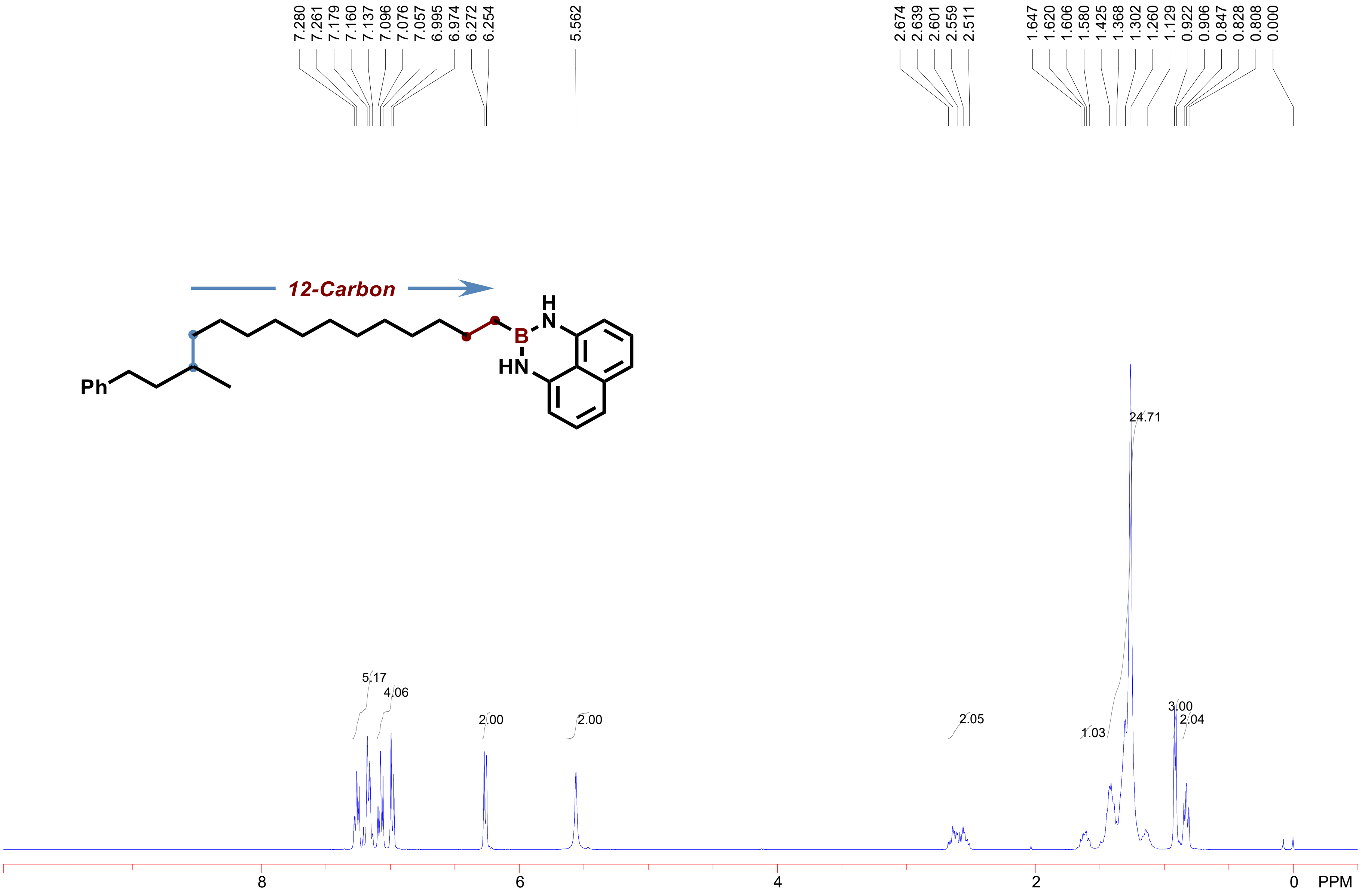

$^{13}$C NMR-spectrum (100 MHz, CDCl3) of 7

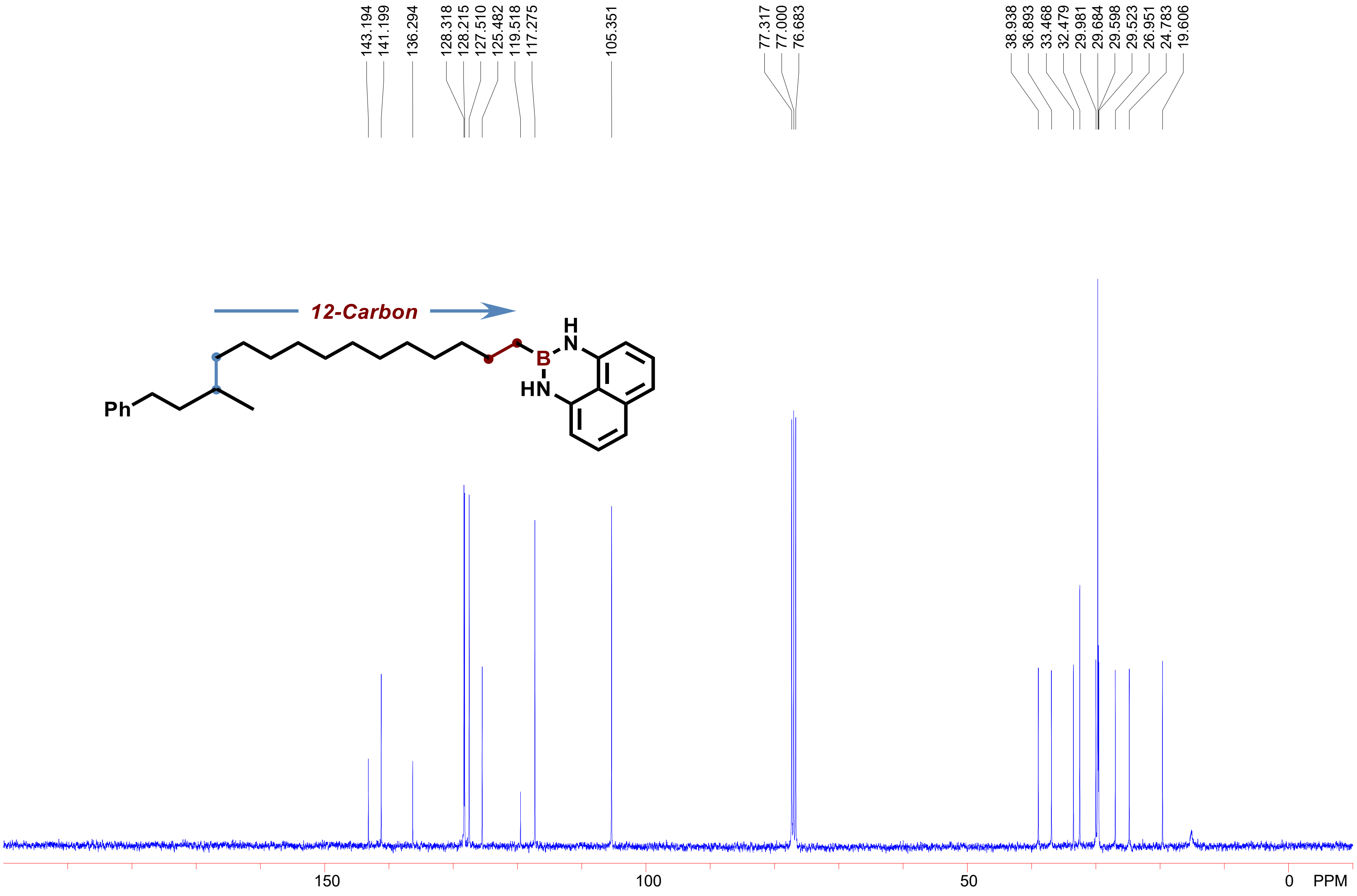

$^{1}H$ NMR-spectrum (400 MHz, $CDCl_3$) of 8

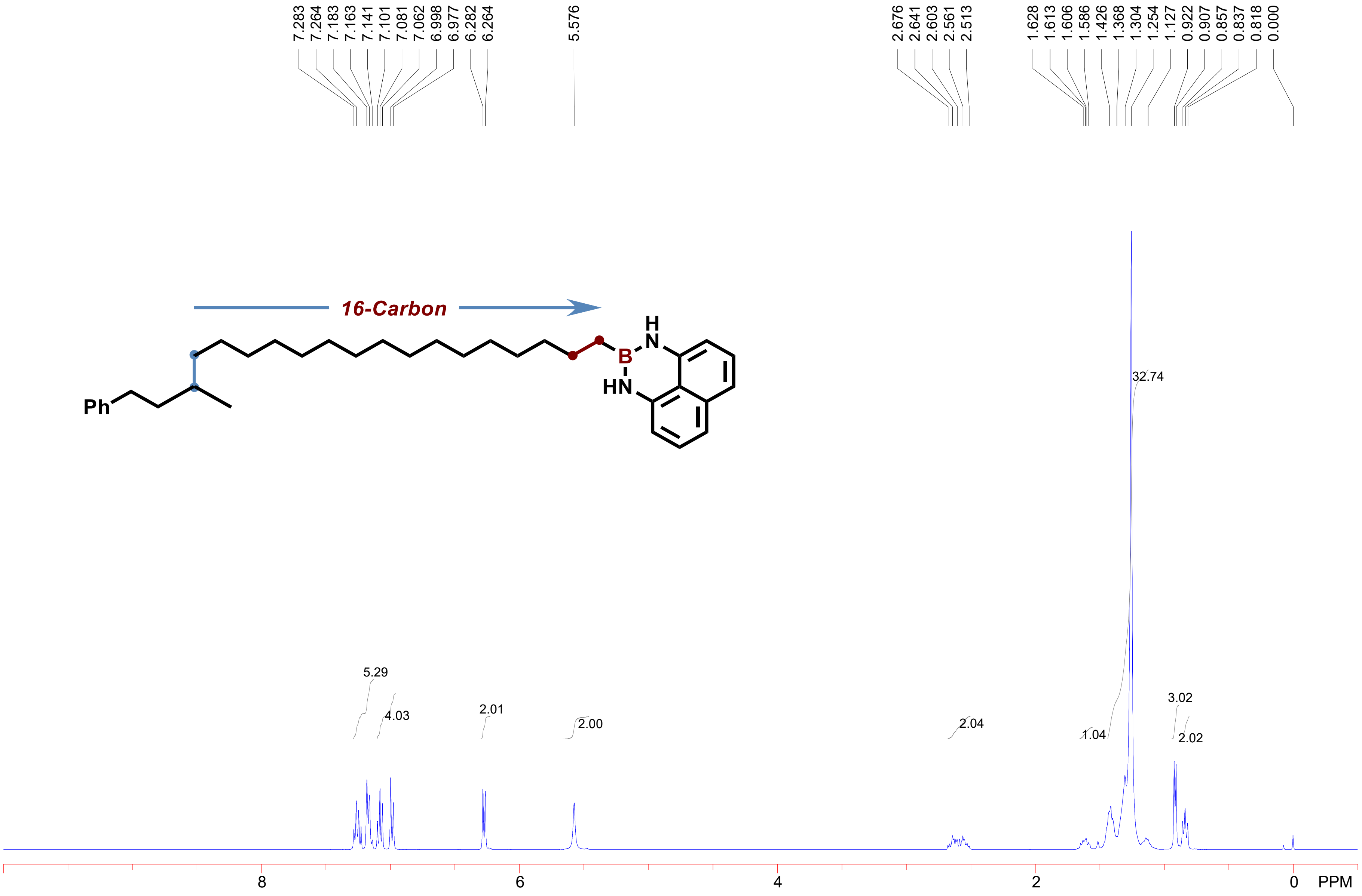

$^{13}$C NMR-spectrum (100 MHz, CDCl3) of 8

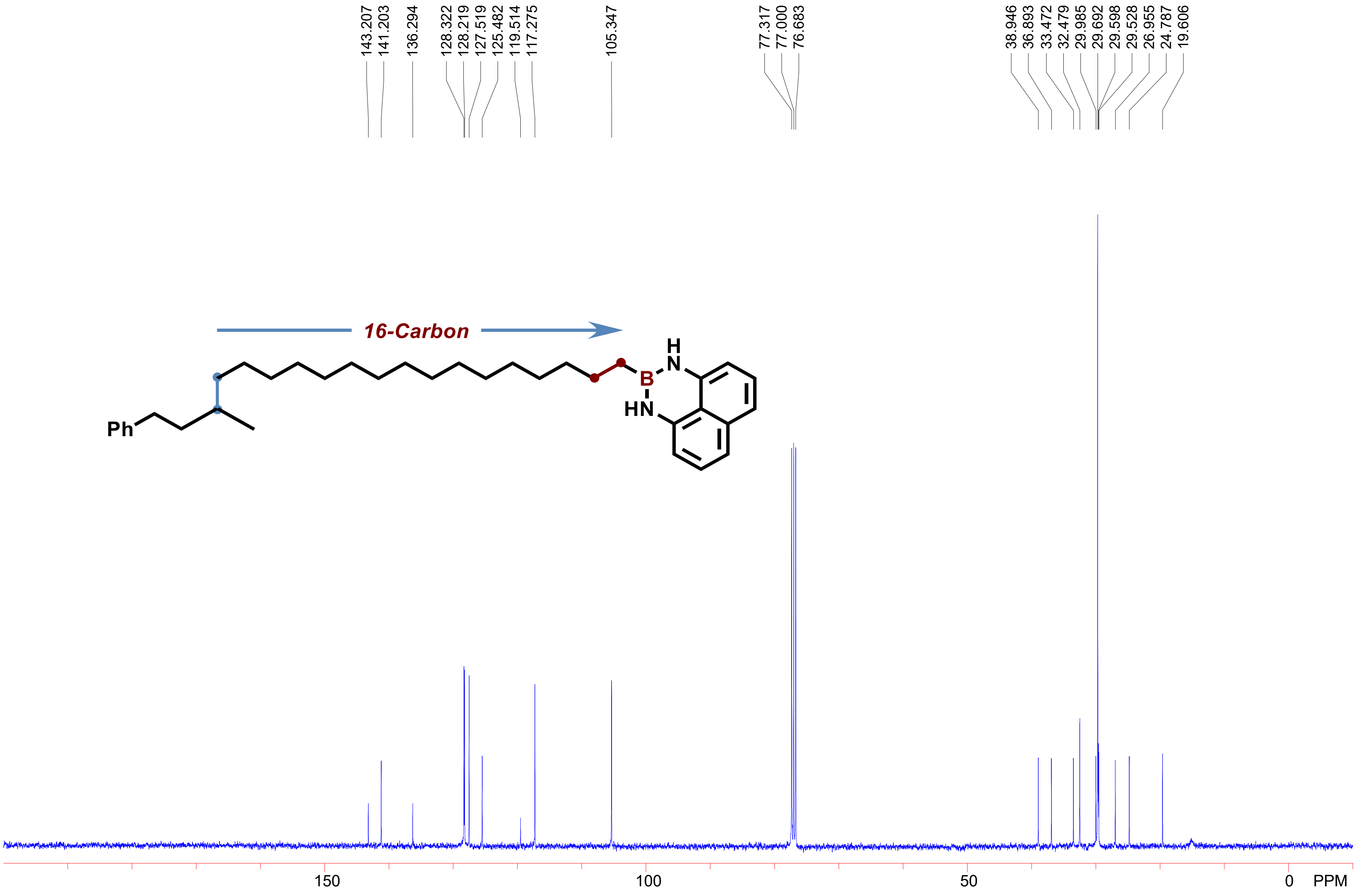

## $^{1}$H NMR-spectrum (400 MHz, $CDCl_3$) of 9

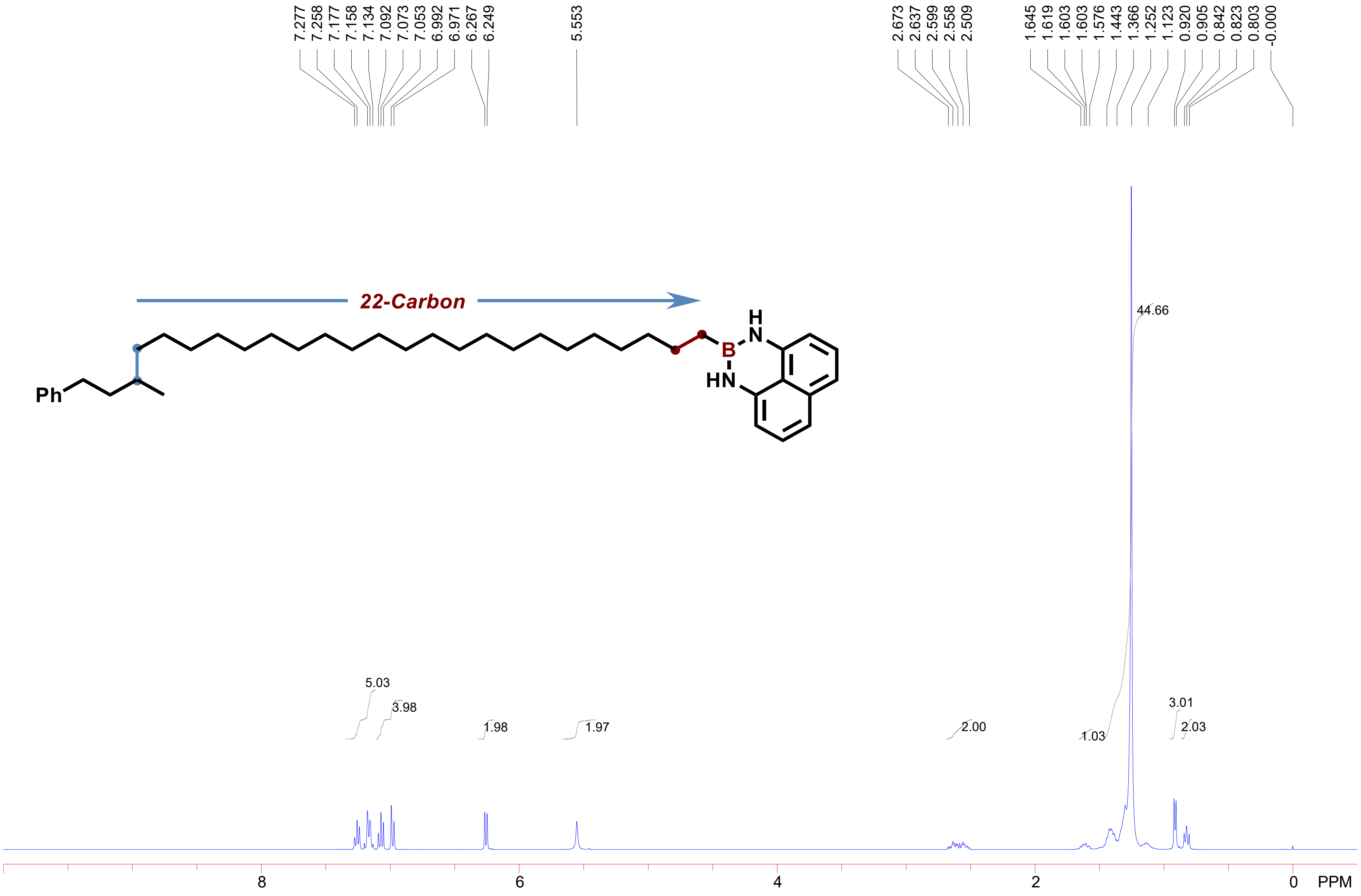

$^{13}$C NMR-spectrum (100 MHz, $CDCl_3$) of 9

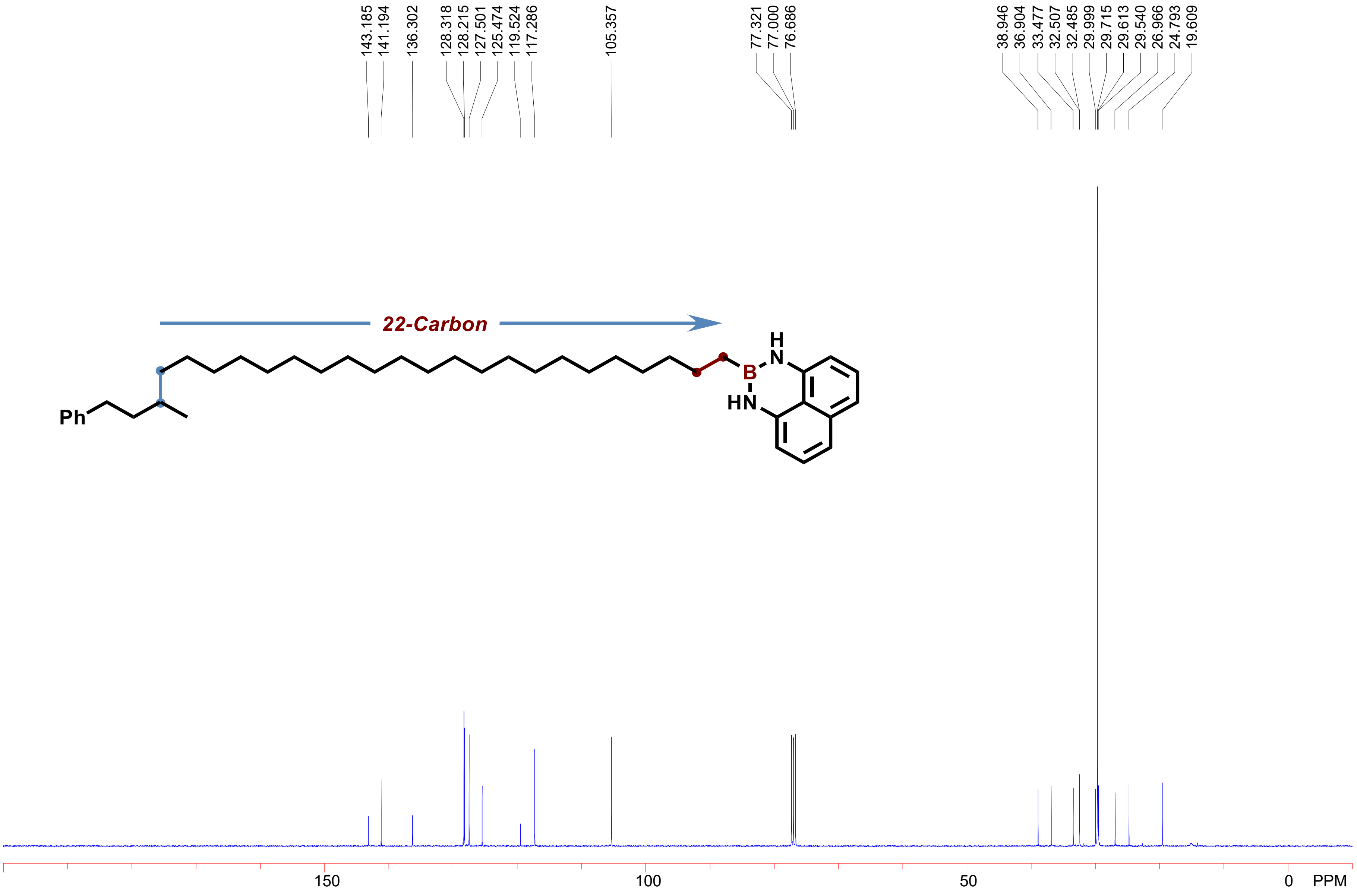

## $^{1}$H NMR-spectrum (400 MHz, $CDCl_3$) of 10

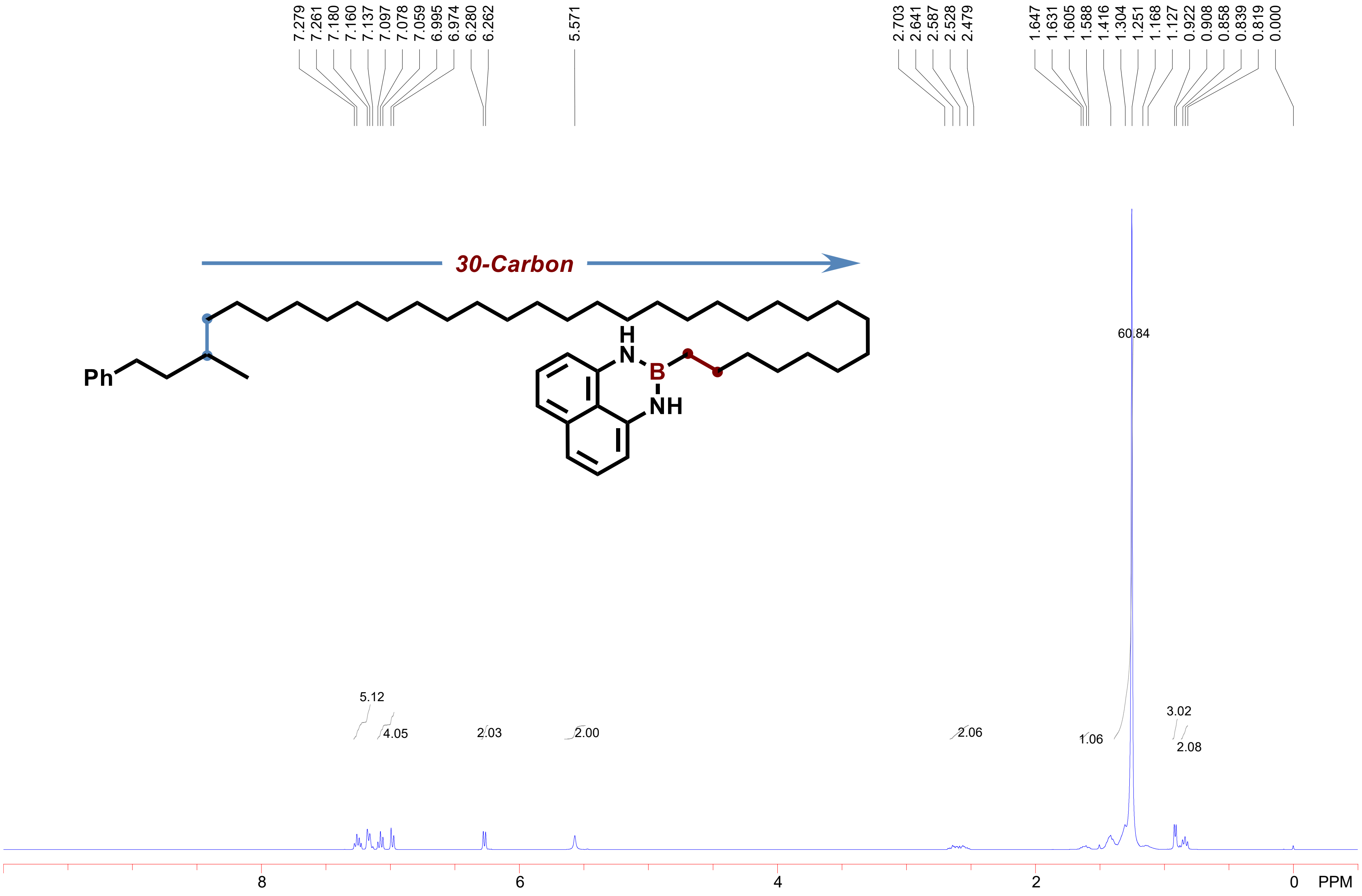

$^{13}$C NMR-spectrum (100 MHz, CDCl3) of 10

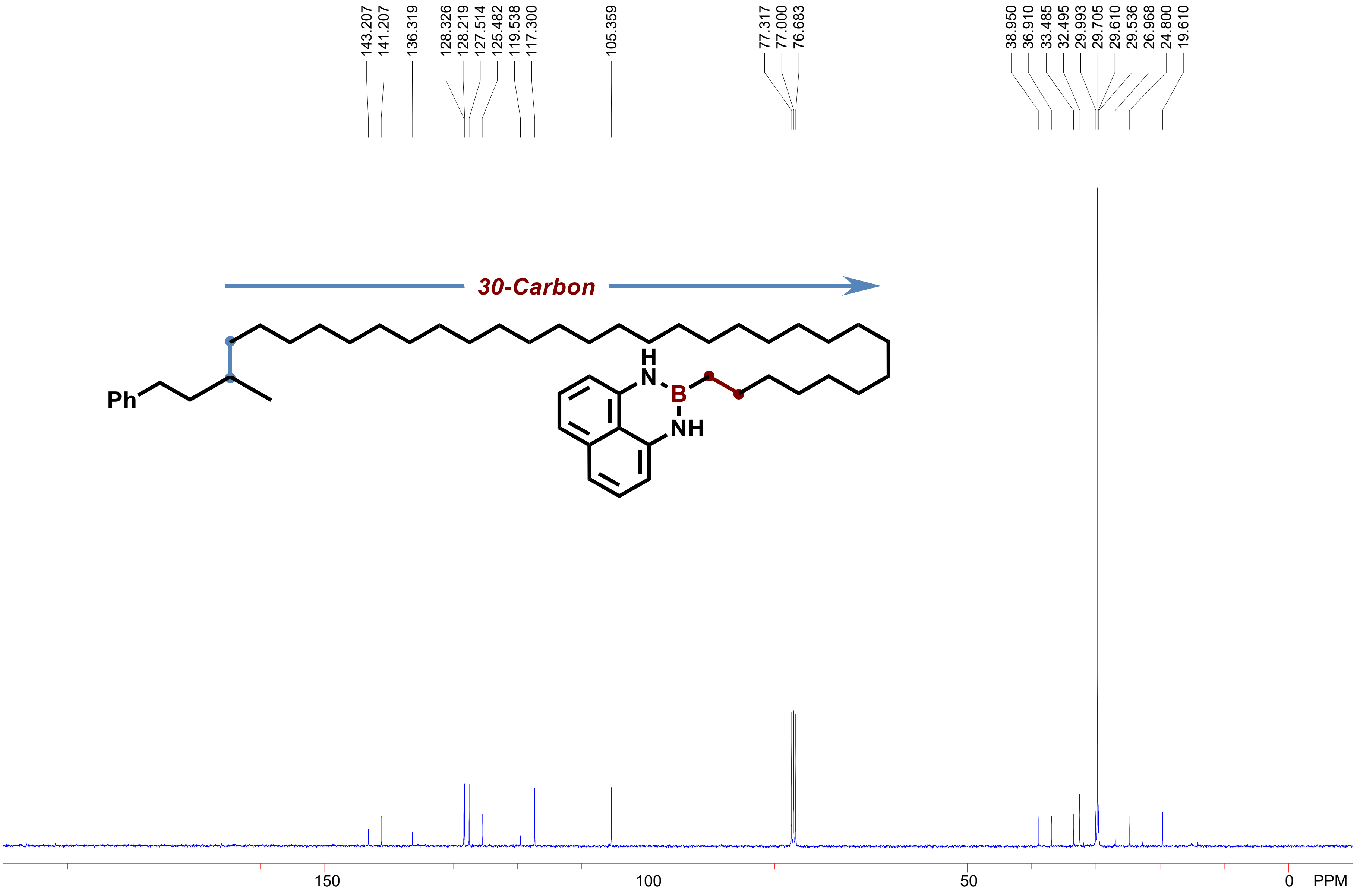

**$^{1}$H NMR-spectrum (400 MHz, CDCl3) of 11**

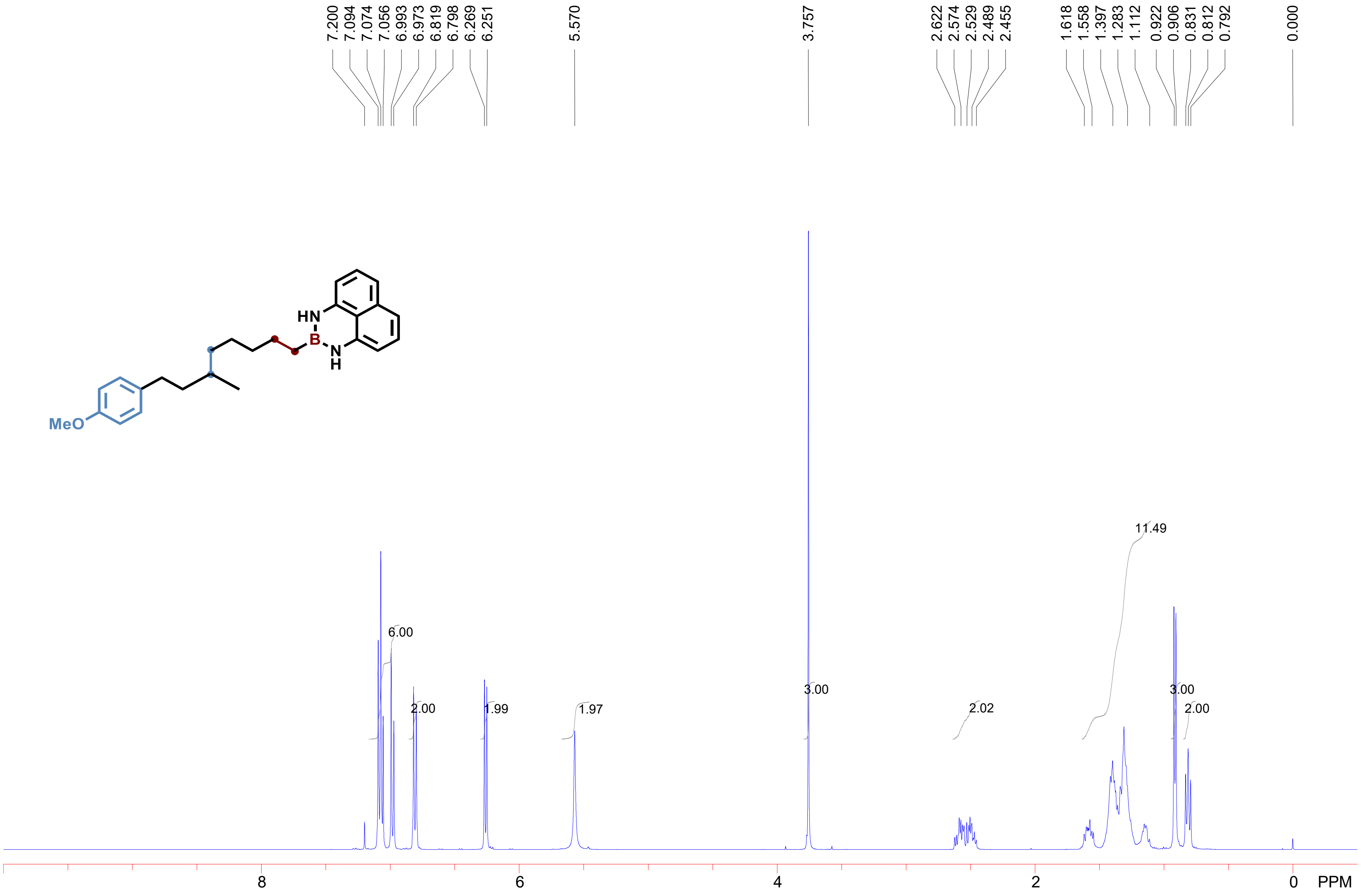

### $^{13}$C NMR-spectrum (100 MHz, $CDCl_3$) of 11

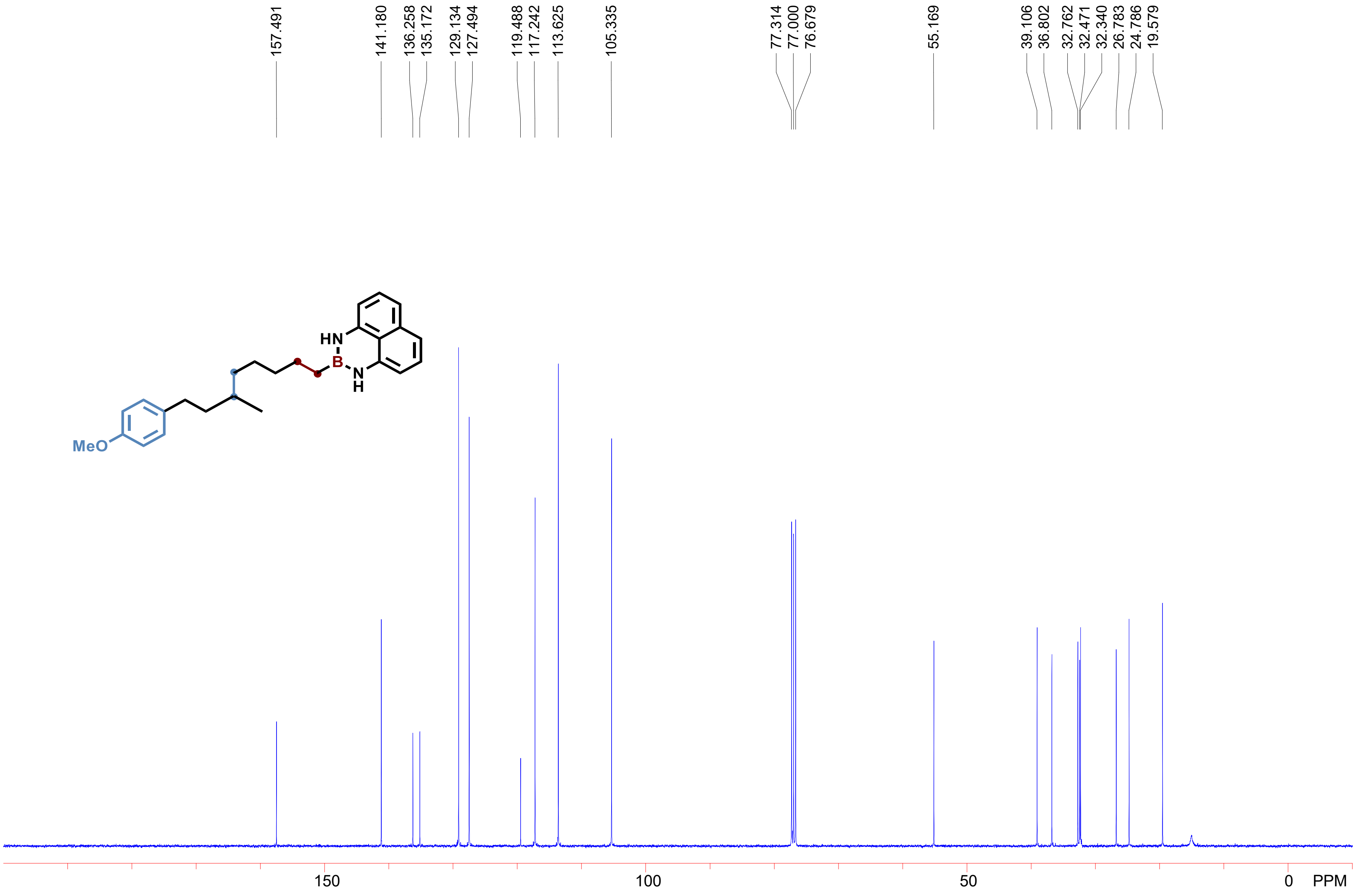

**$^{1}$H NMR-spectrum (400 MHz, CDCl3) of 12**

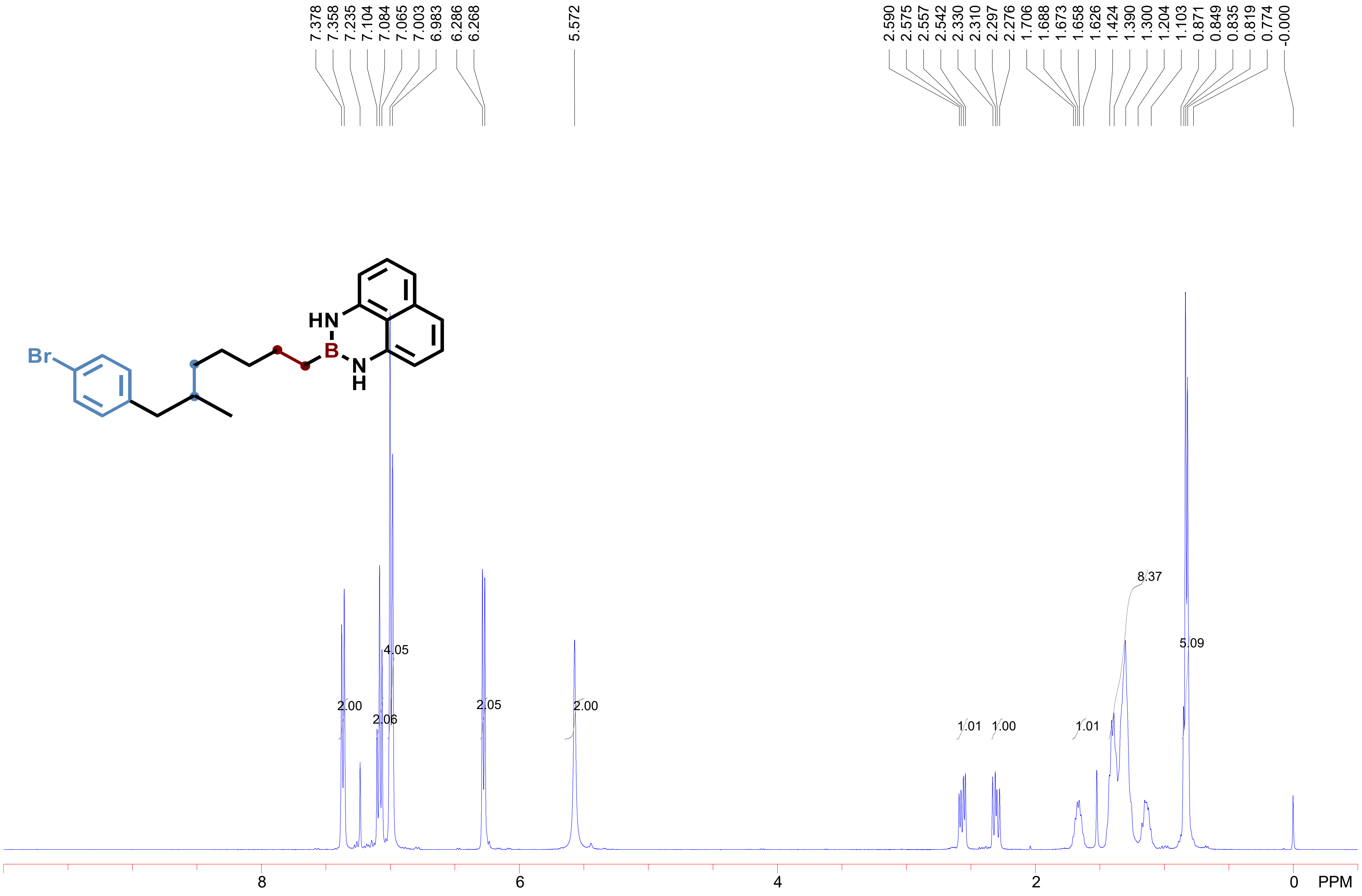

$^{13}C$ NMR-spectrum (100 MHz, $CDCl_3$) of 12

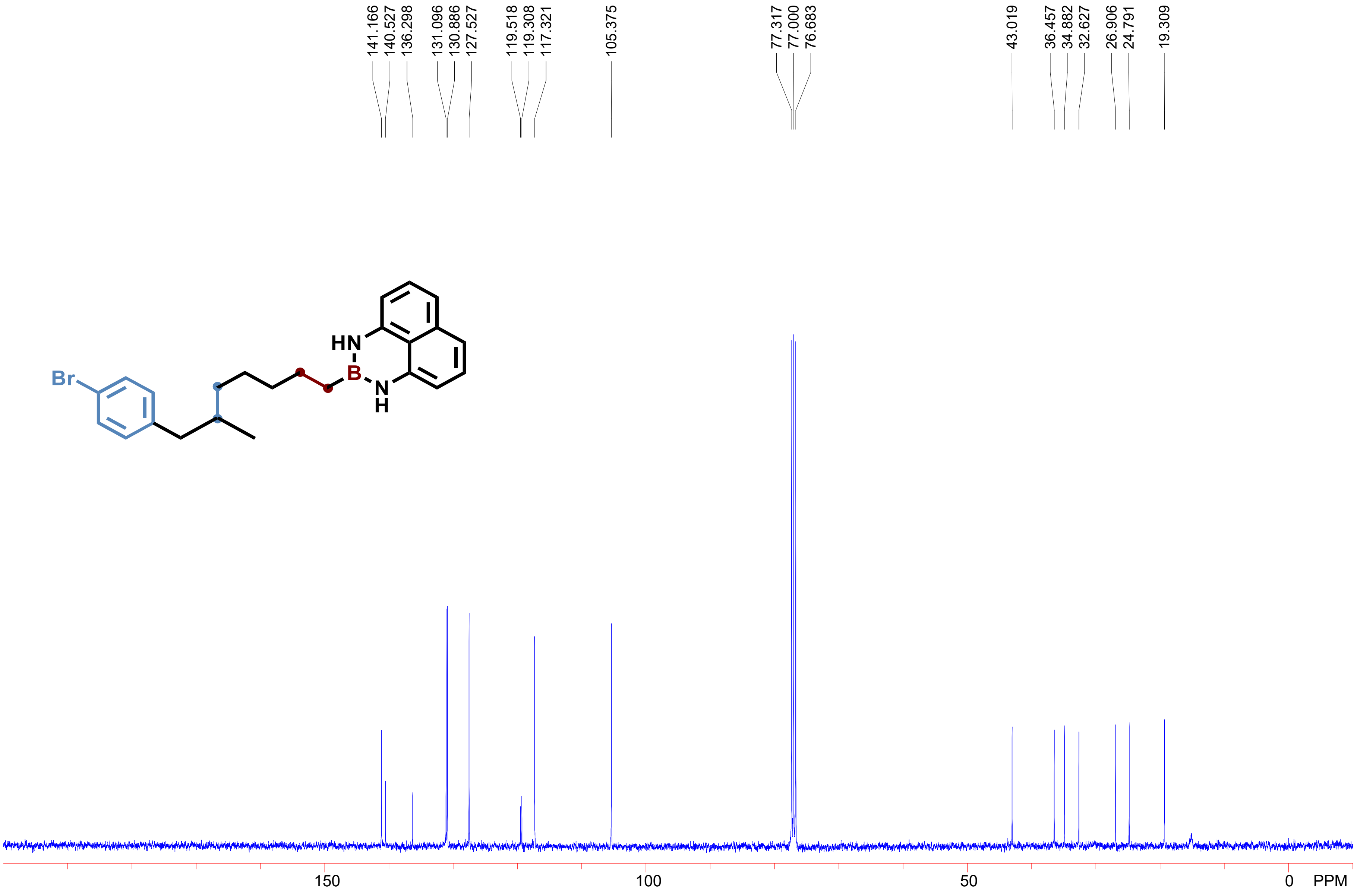

$^{1}$H NMR-spectrum (400 MHz, $CDCl_3$) of 13

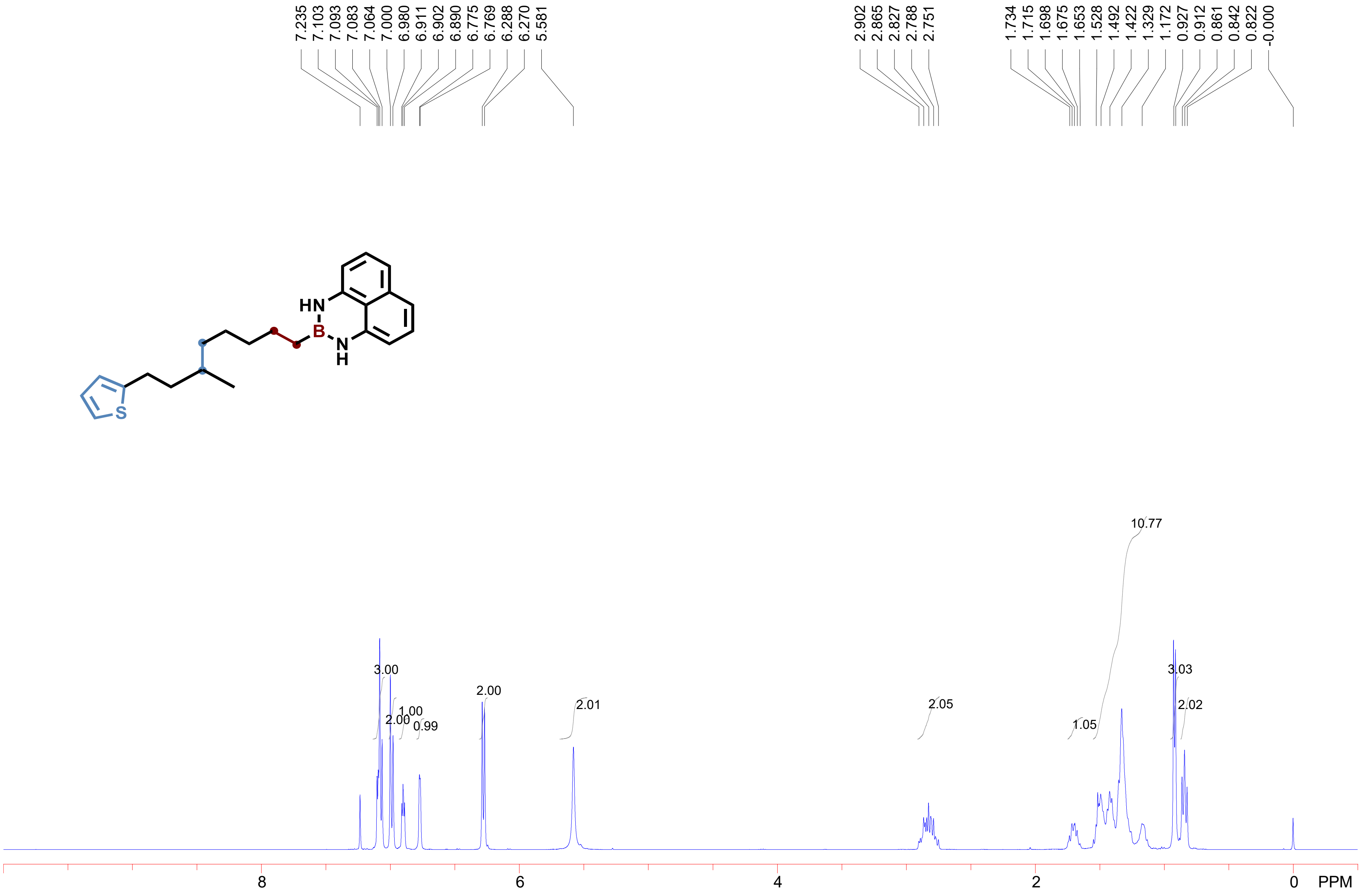

**$^{13}$C NMR-spectrum (100 MHz, CDCl₃) of 13**

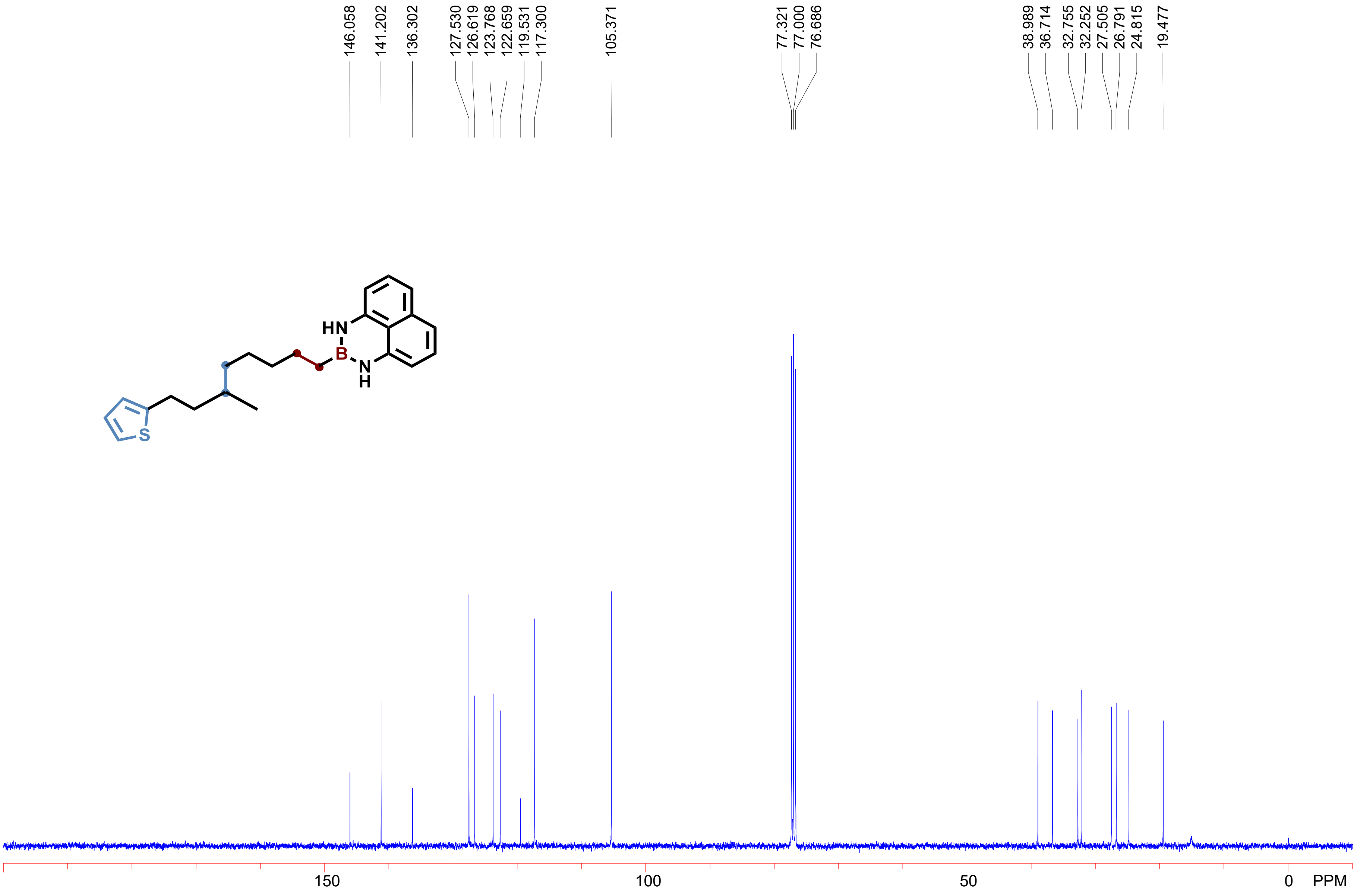

**$^{1}$H NMR-spectrum (400 MHz, CDCl3) of 14**

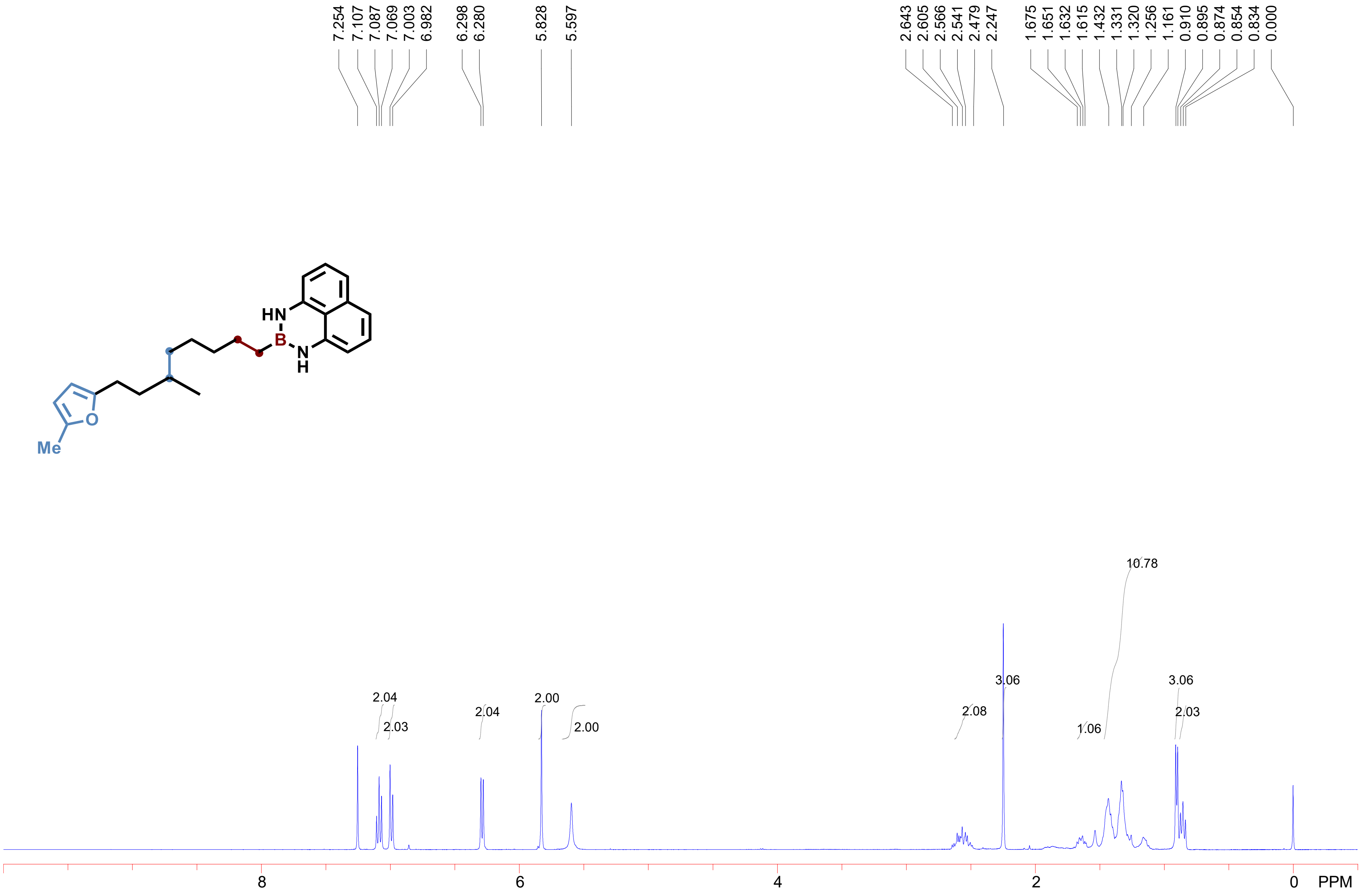

## $^{13}$C NMR-spectrum (100 MHz, $CDCl_3$) of 14

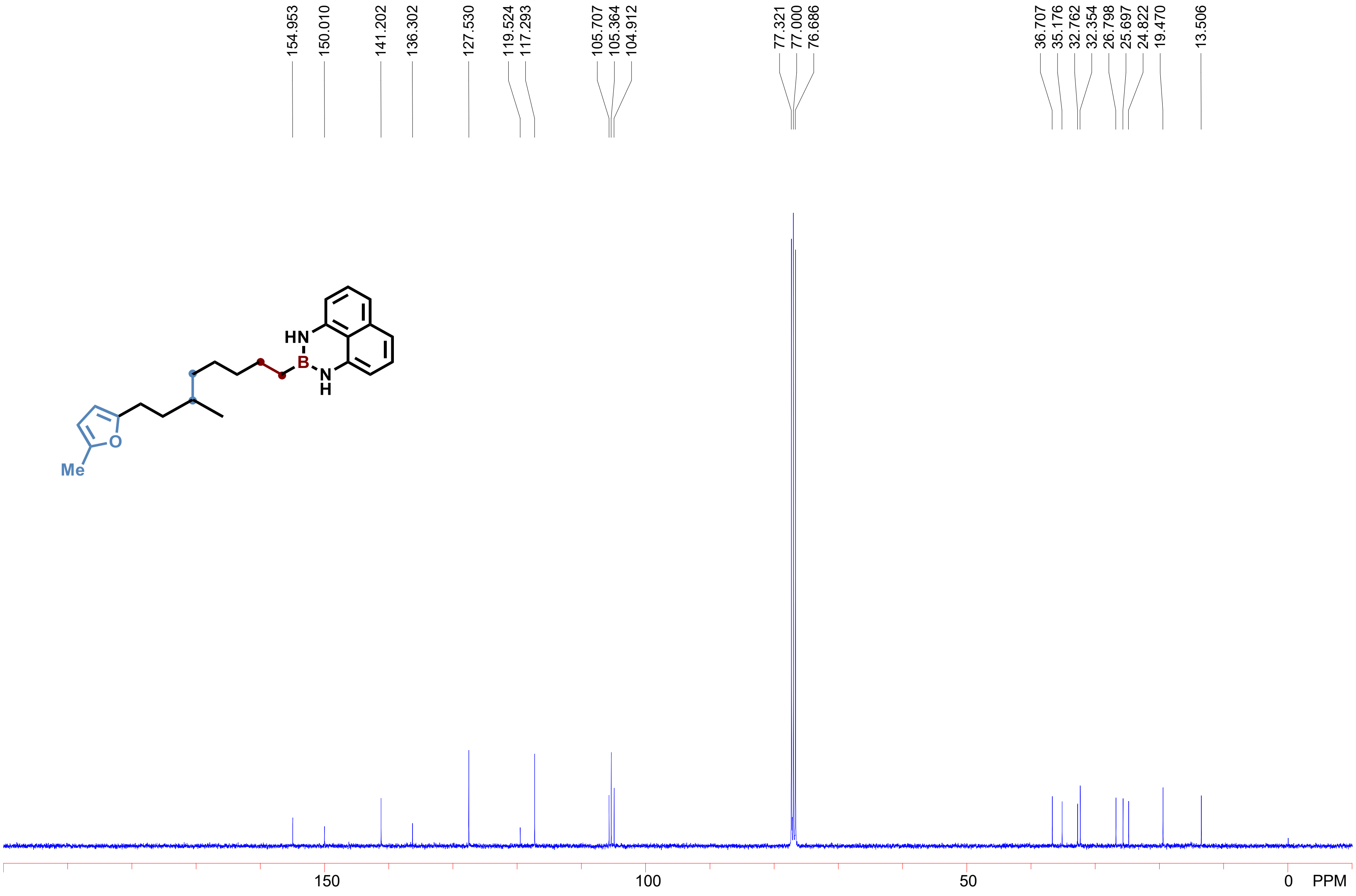

**$^{1}$H NMR-spectrum (400 MHz, $CDCl_3$) of 15**

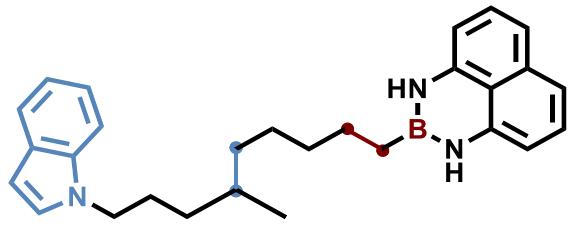


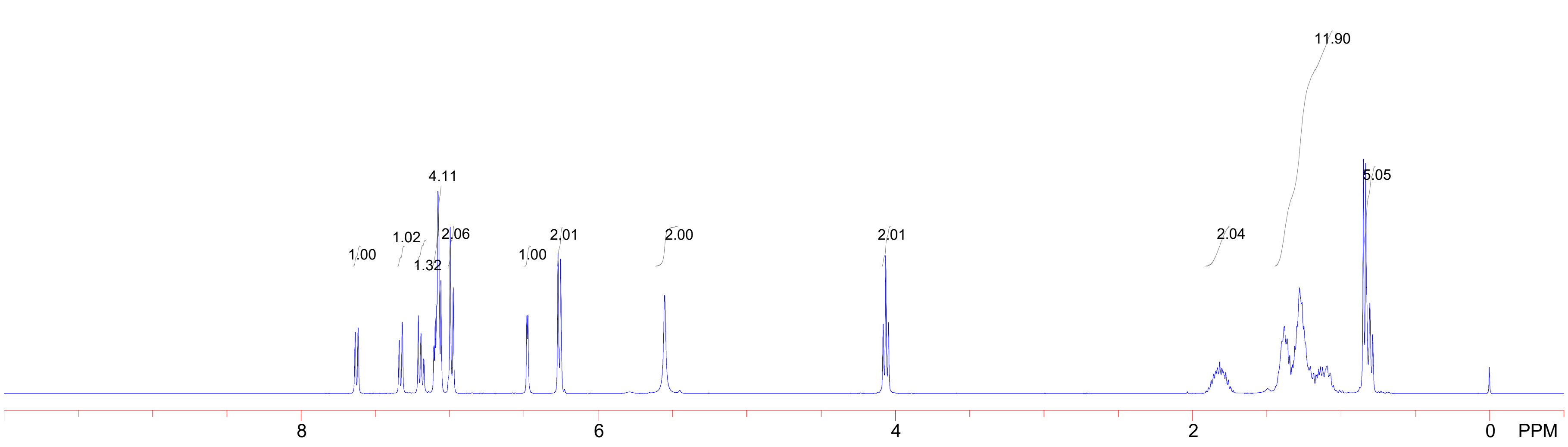

**$^{13}C$ NMR-spectrum (100 MHz, $CDCl_3$) of 15**

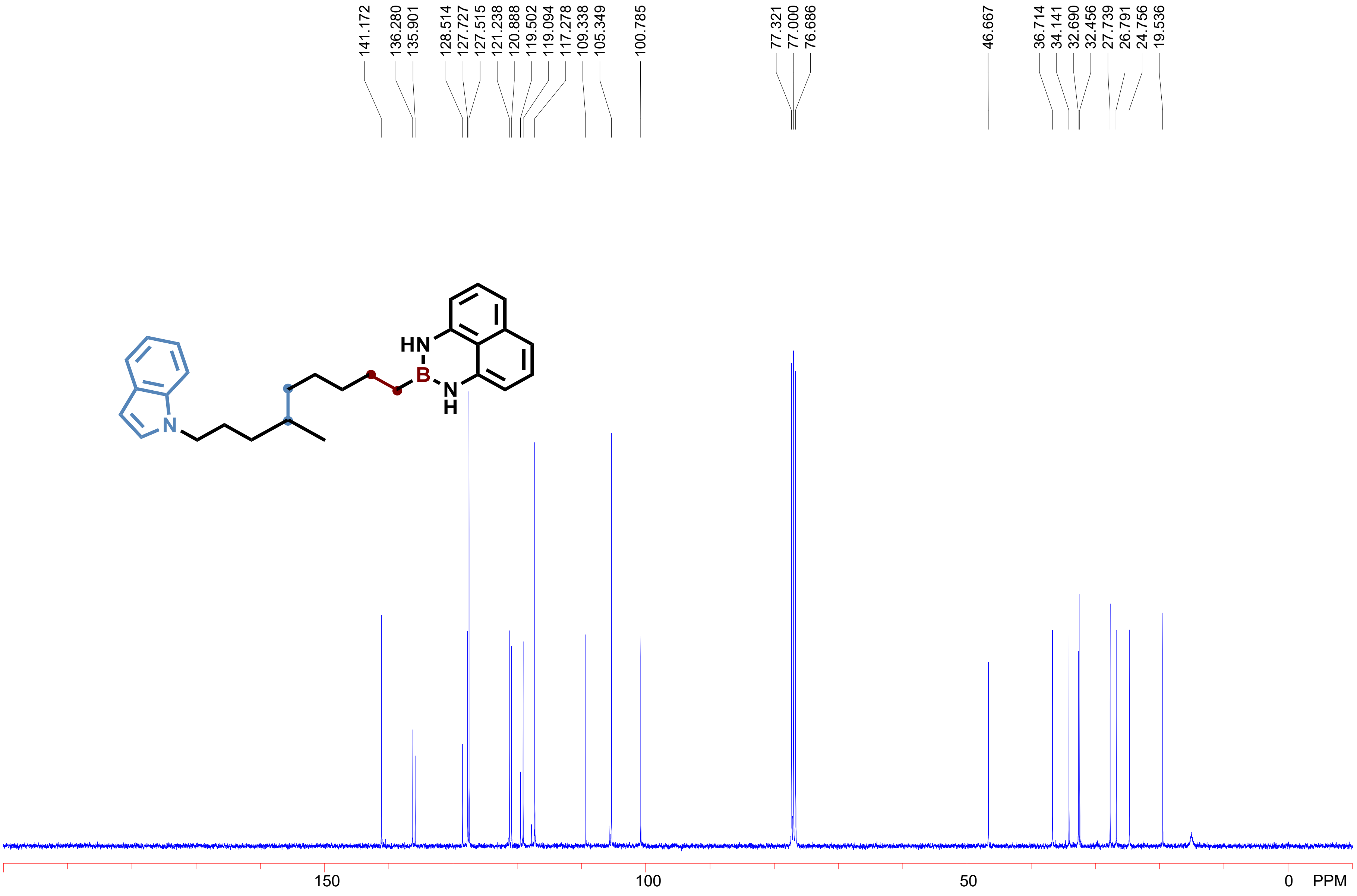

**$^{1}$H NMR-spectrum (400 MHz, CDCl3) of 16**

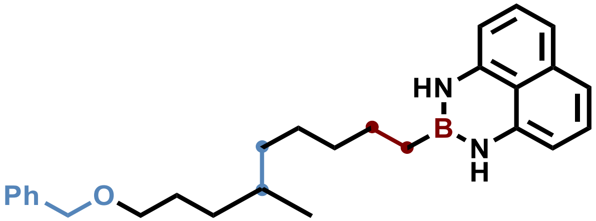


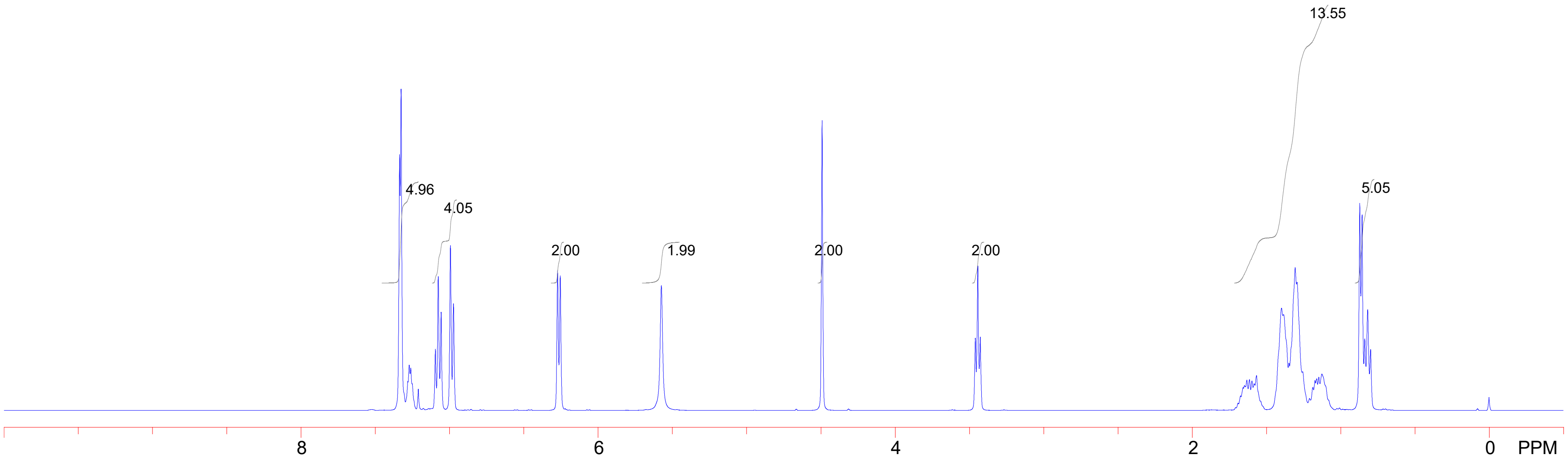

**$^{13}$C NMR-spectrum (100 MHz, CDCl3) of 16**

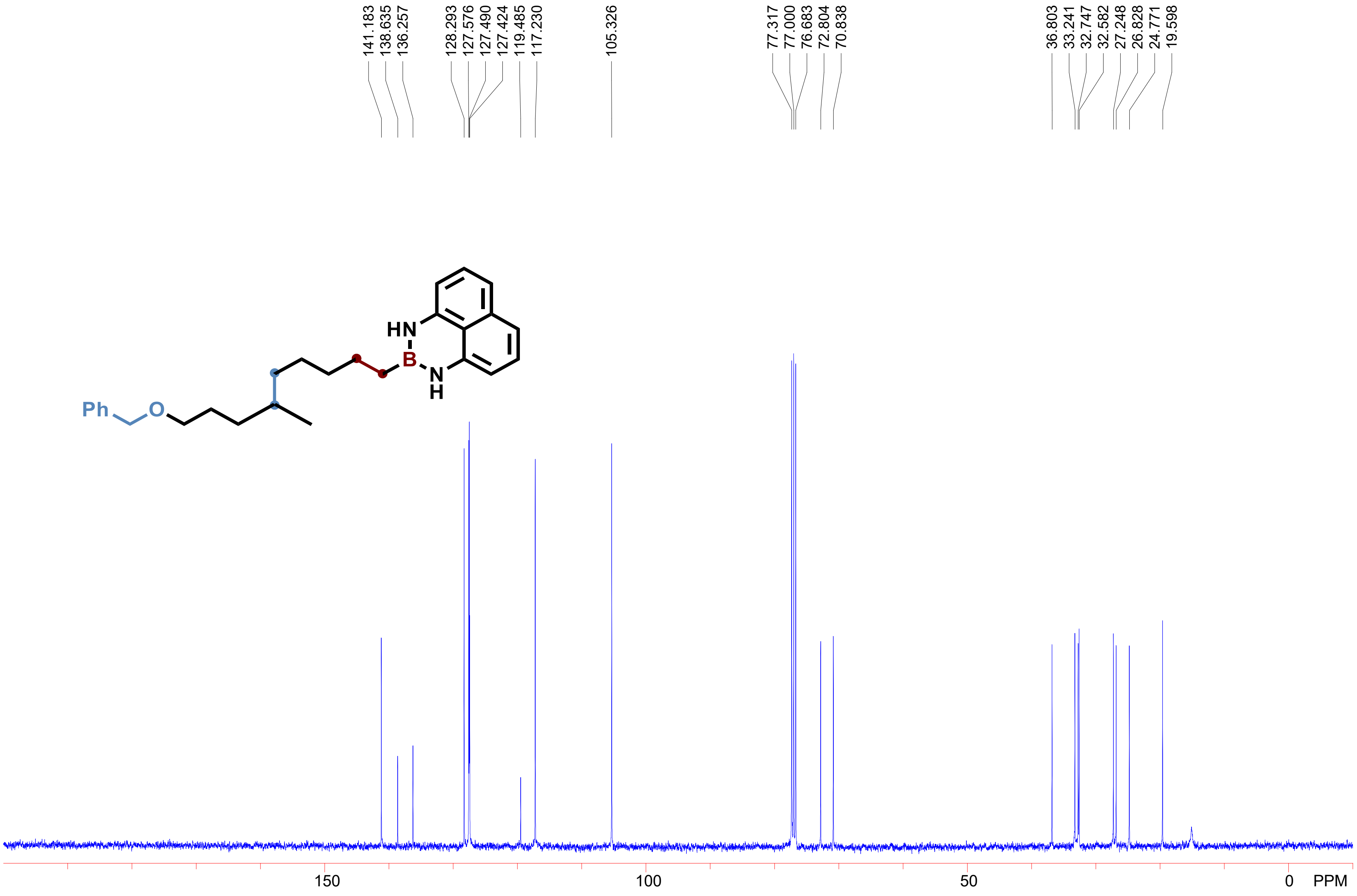

# $^{1}$H NMR-spectrum (400 MHz, CDCl3) of 17

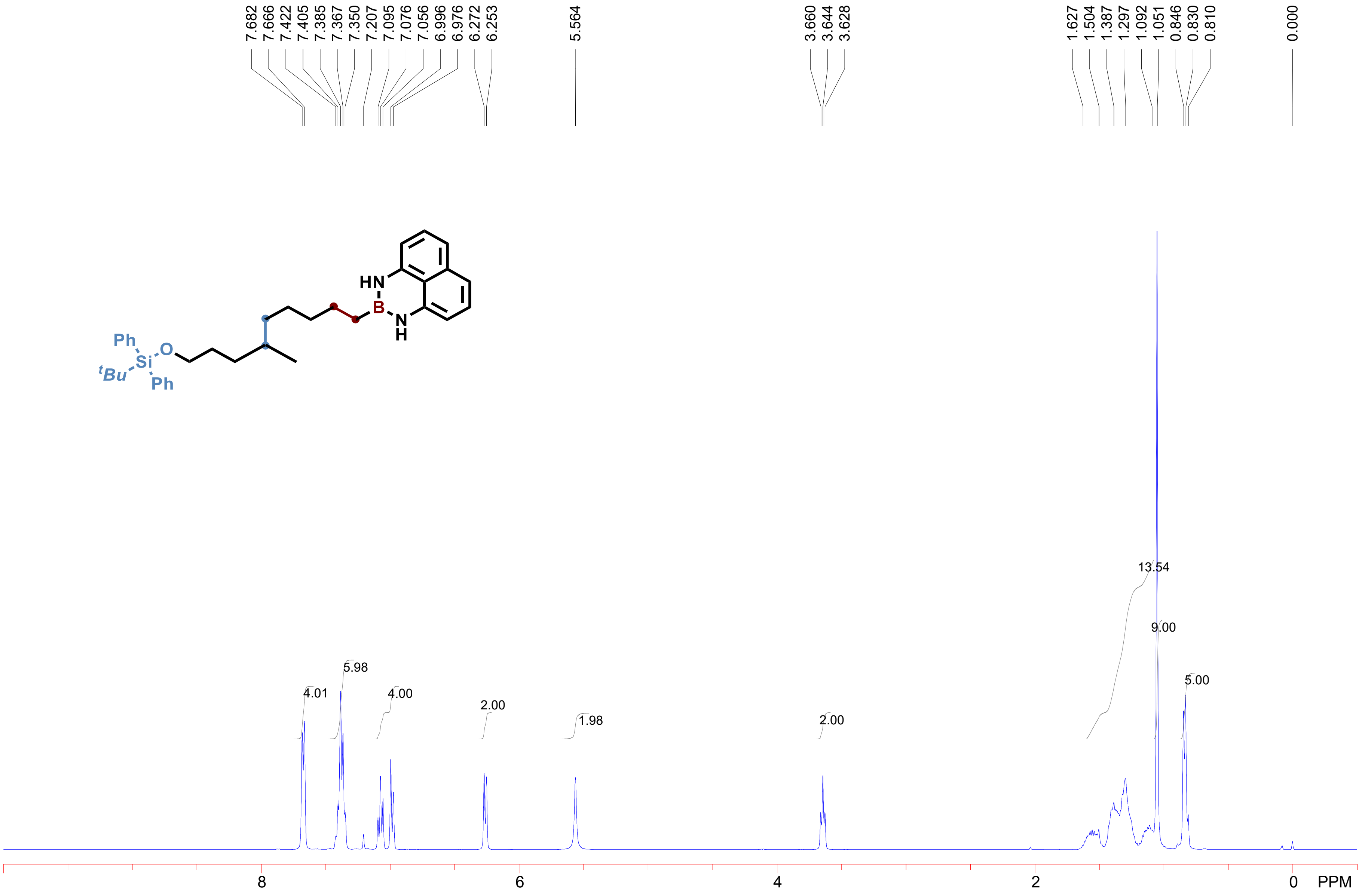

## $^{13}$C NMR-spectrum (100 MHz, $CDCl_3$) of 17

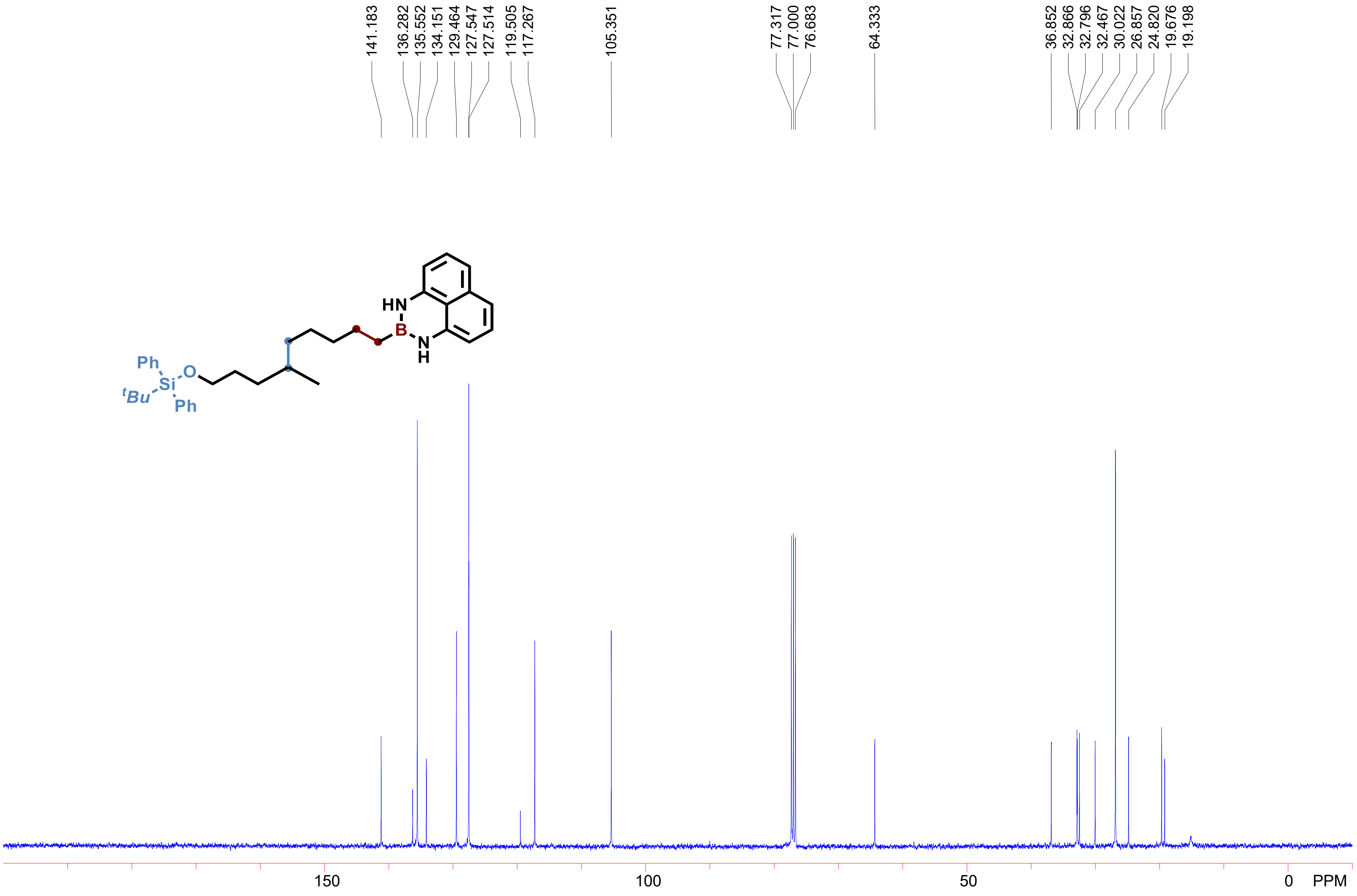

**$^{1}$H NMR-spectrum (400 MHz, CDCl3) of 18**

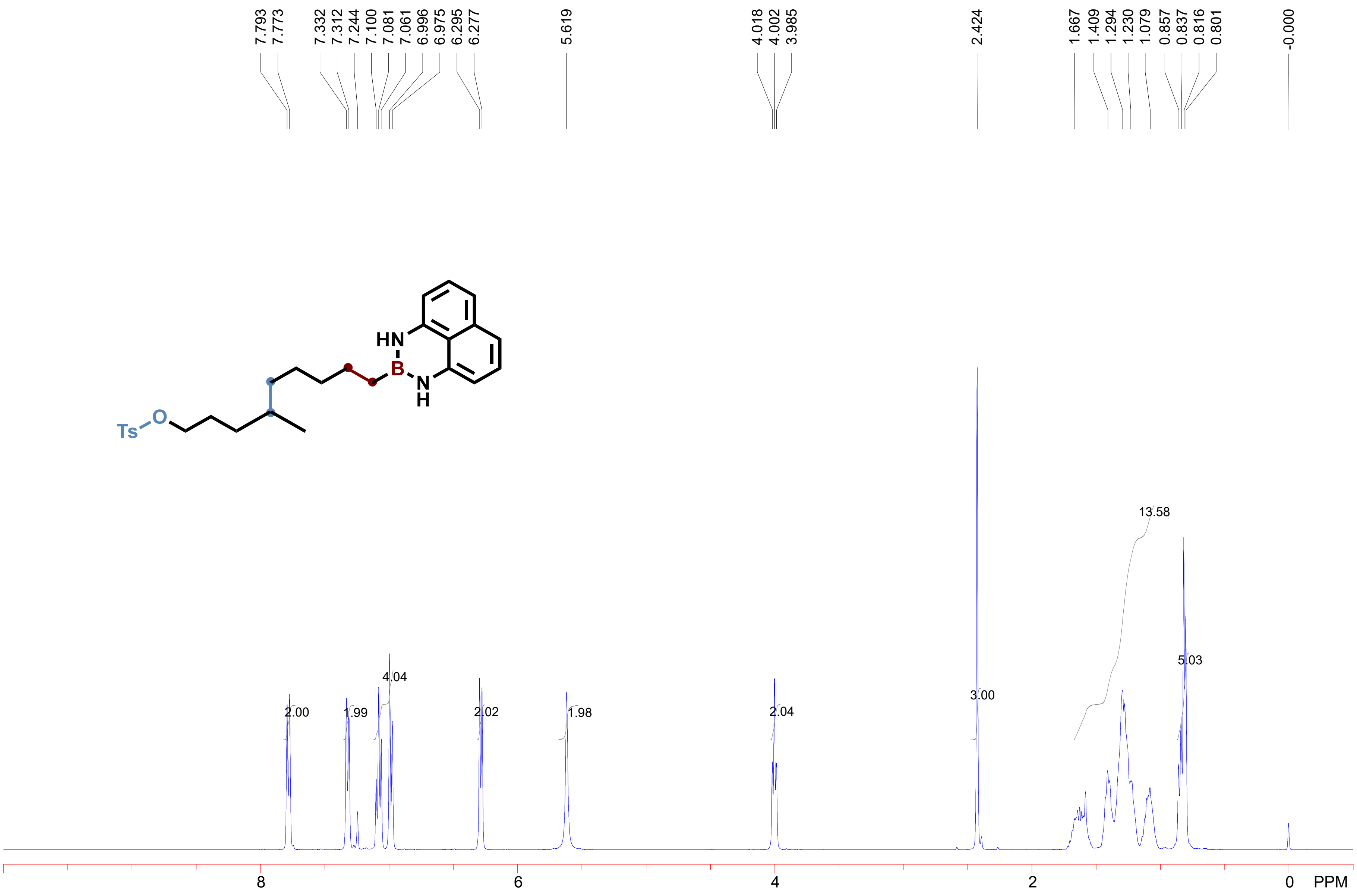

## $^{13}C$ NMR-spectrum (100 MHz, $CDCl_3$) of 18

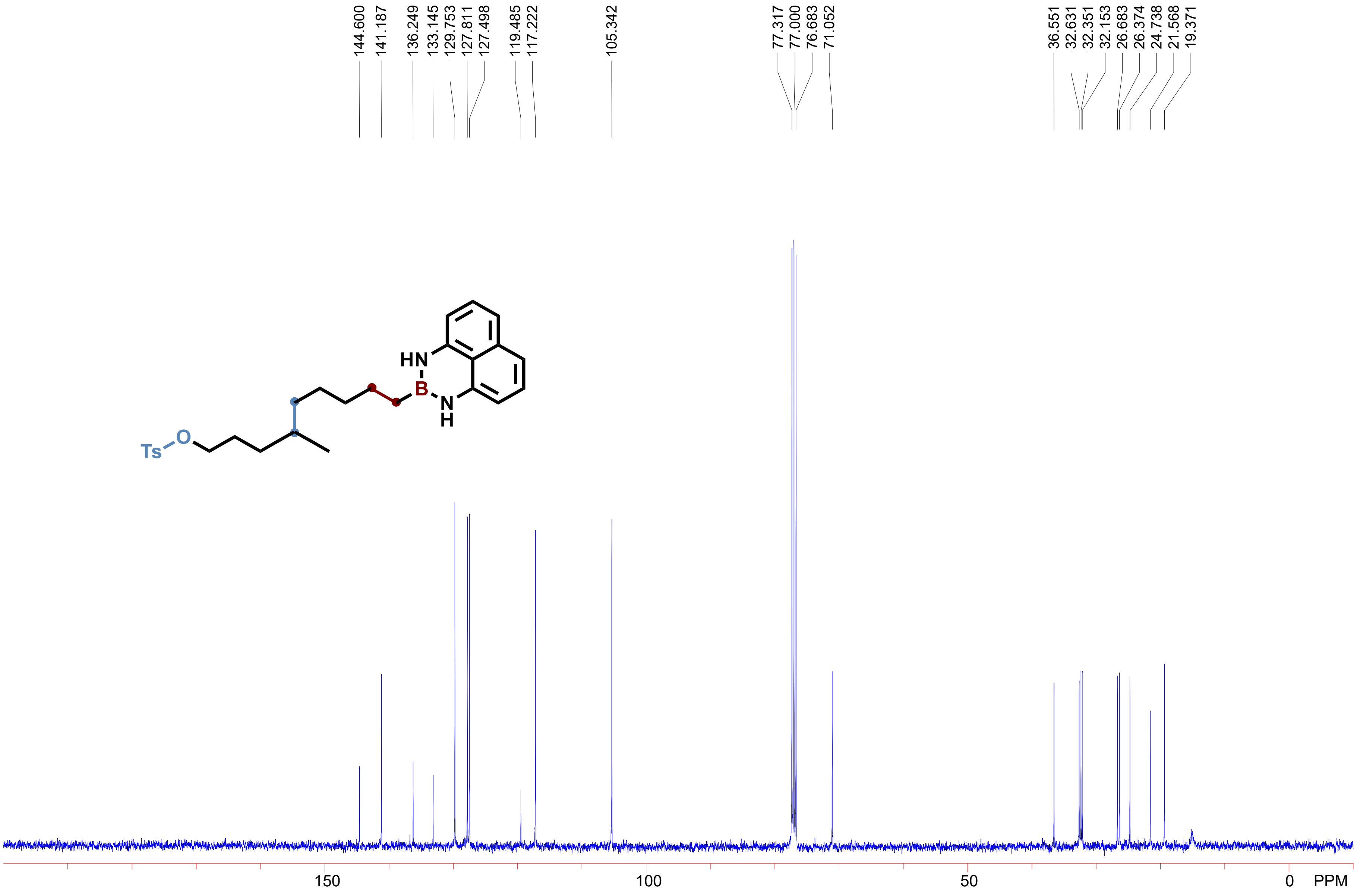

$^{1}$H NMR-spectrum (400 MHz, $CDCl_3$) of 19

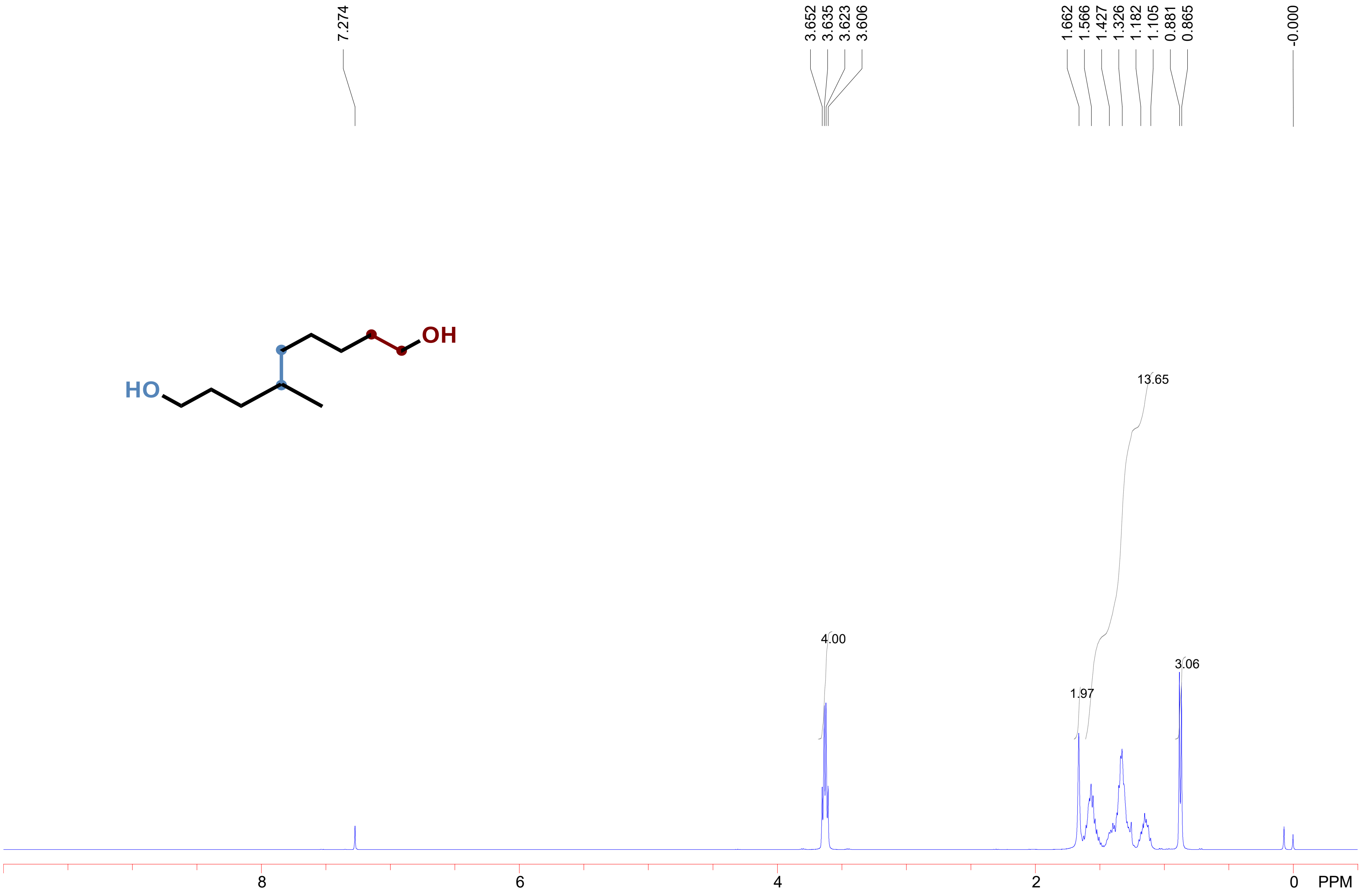

**$^{13}$C NMR-spectrum (100 MHz, $CDCl_3$) of 19**

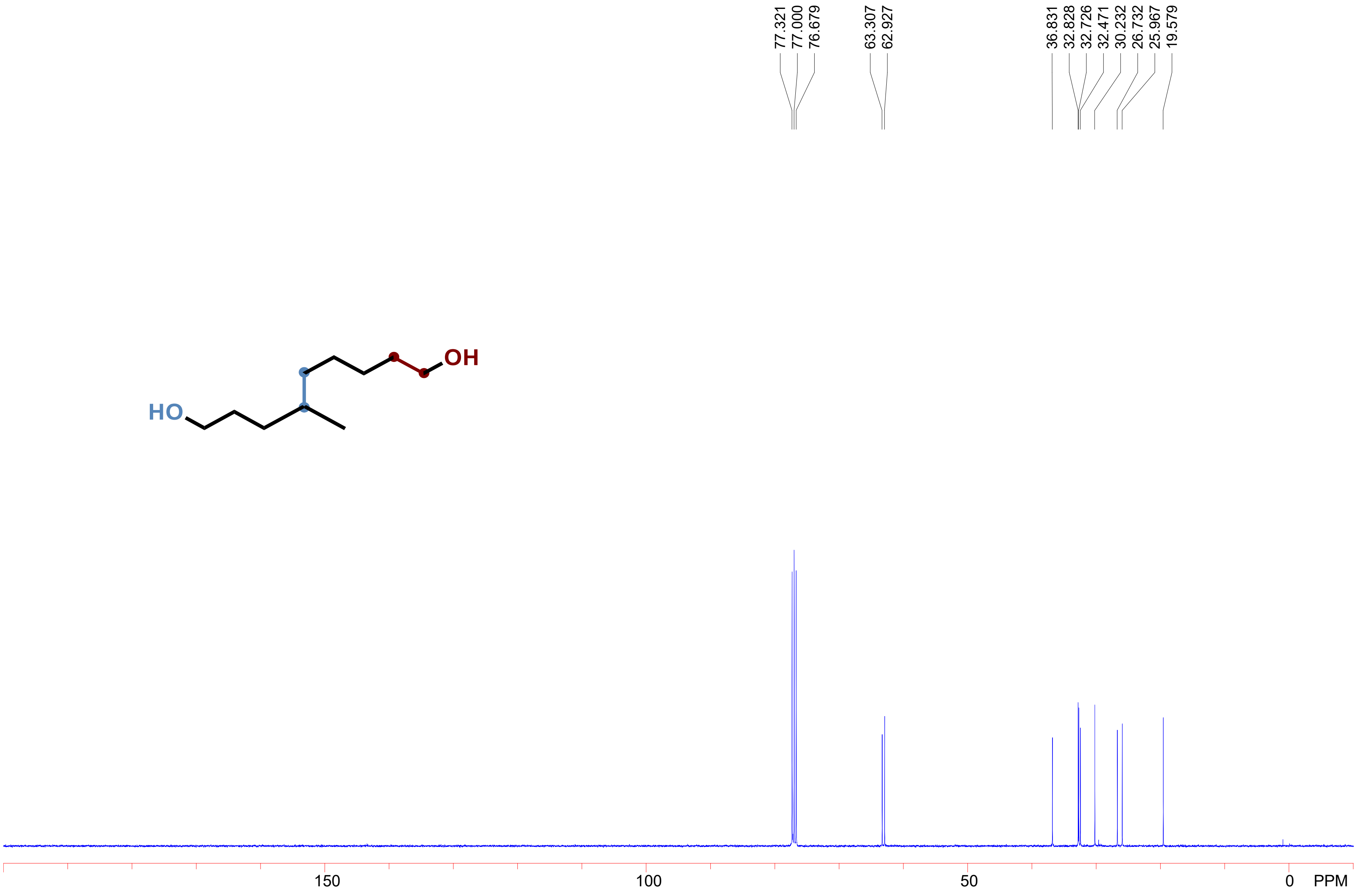

## $^{1}$H NMR-spectrum (400 MHz, $CDCl_3$) of 20

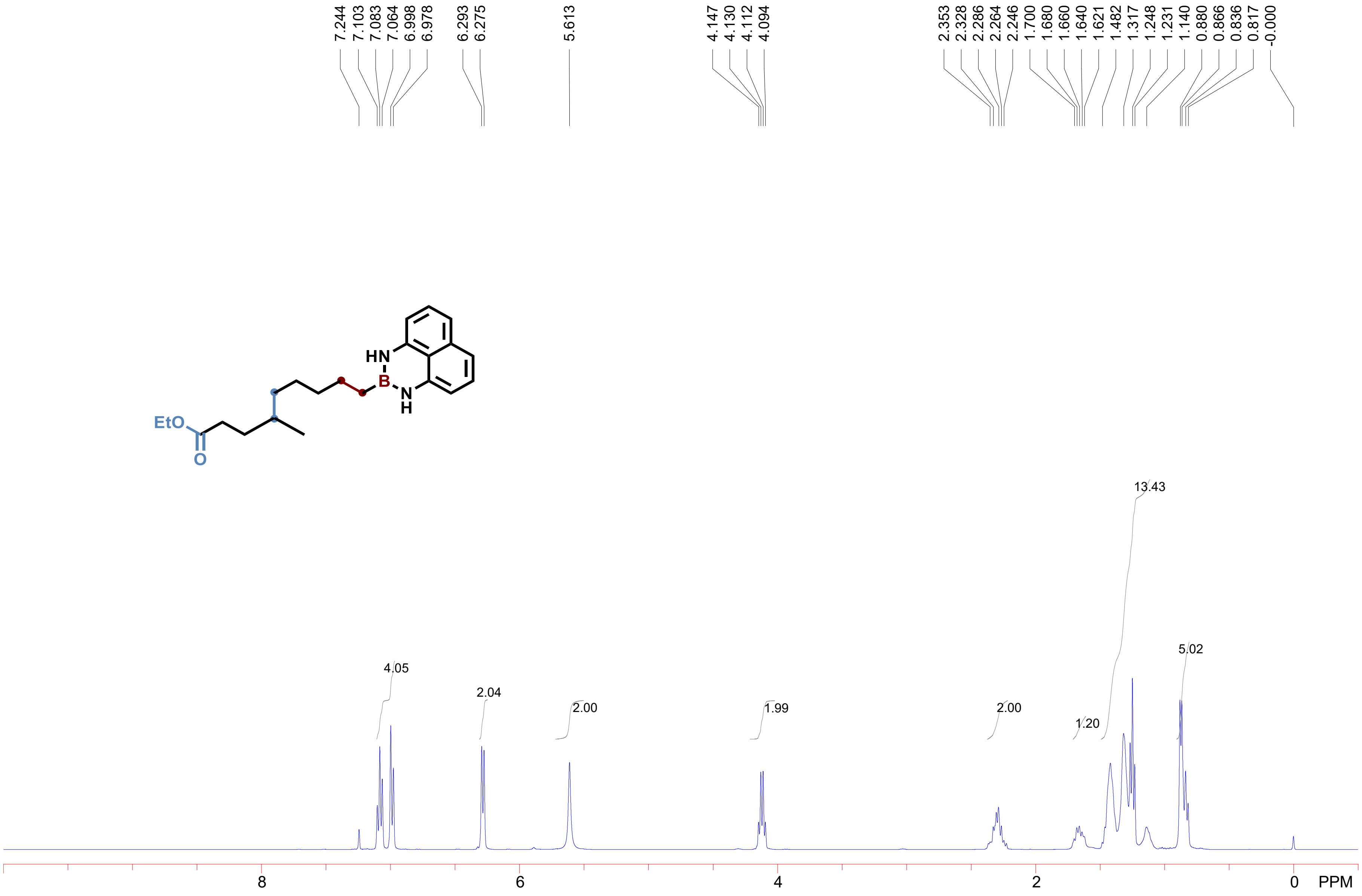

## $^{13}$C NMR-spectrum (100 MHz, $CDCl_3$) of 20

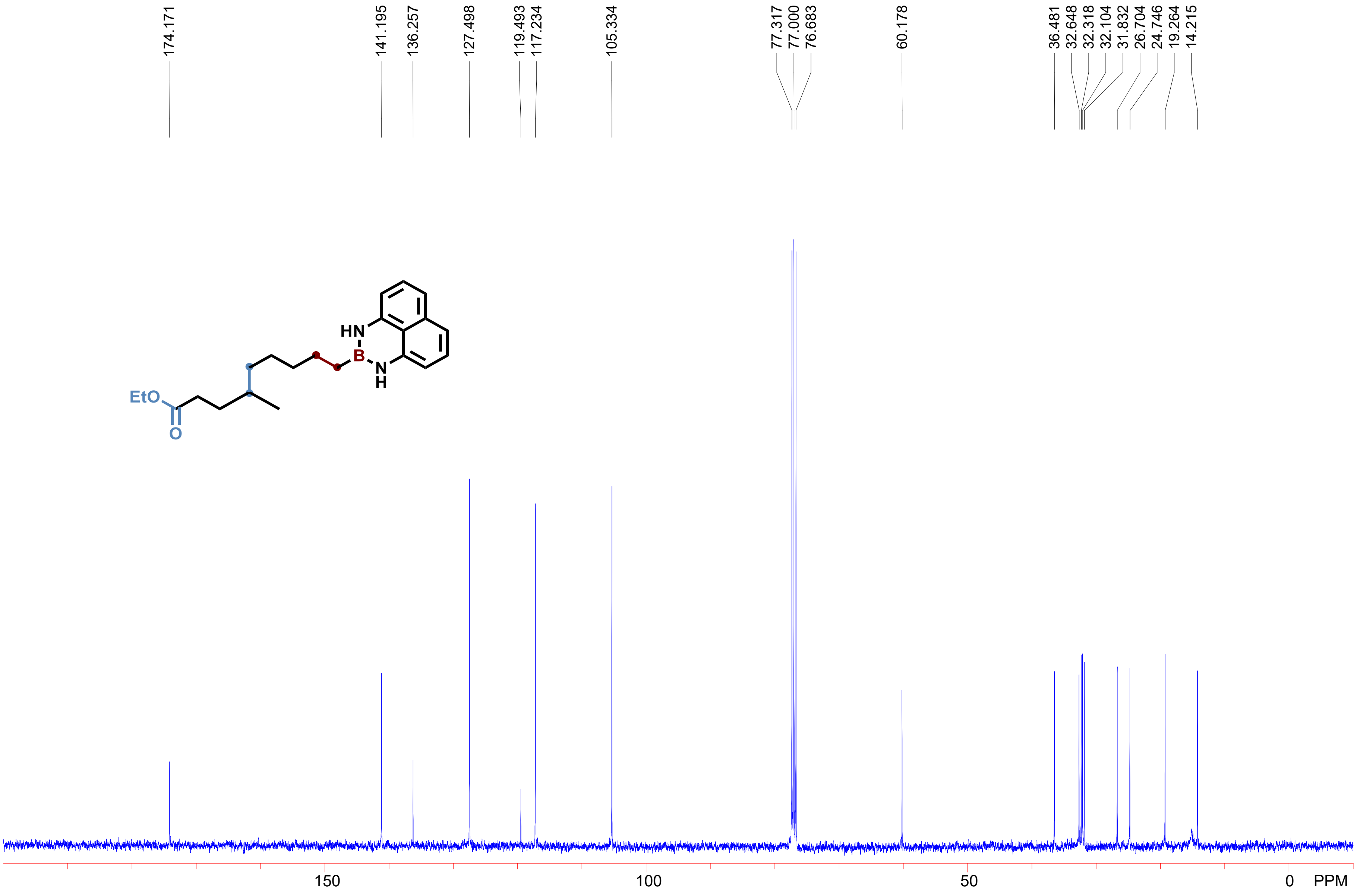

## $^{1}$H NMR-spectrum (400 MHz, $CDCl_3$) of 21

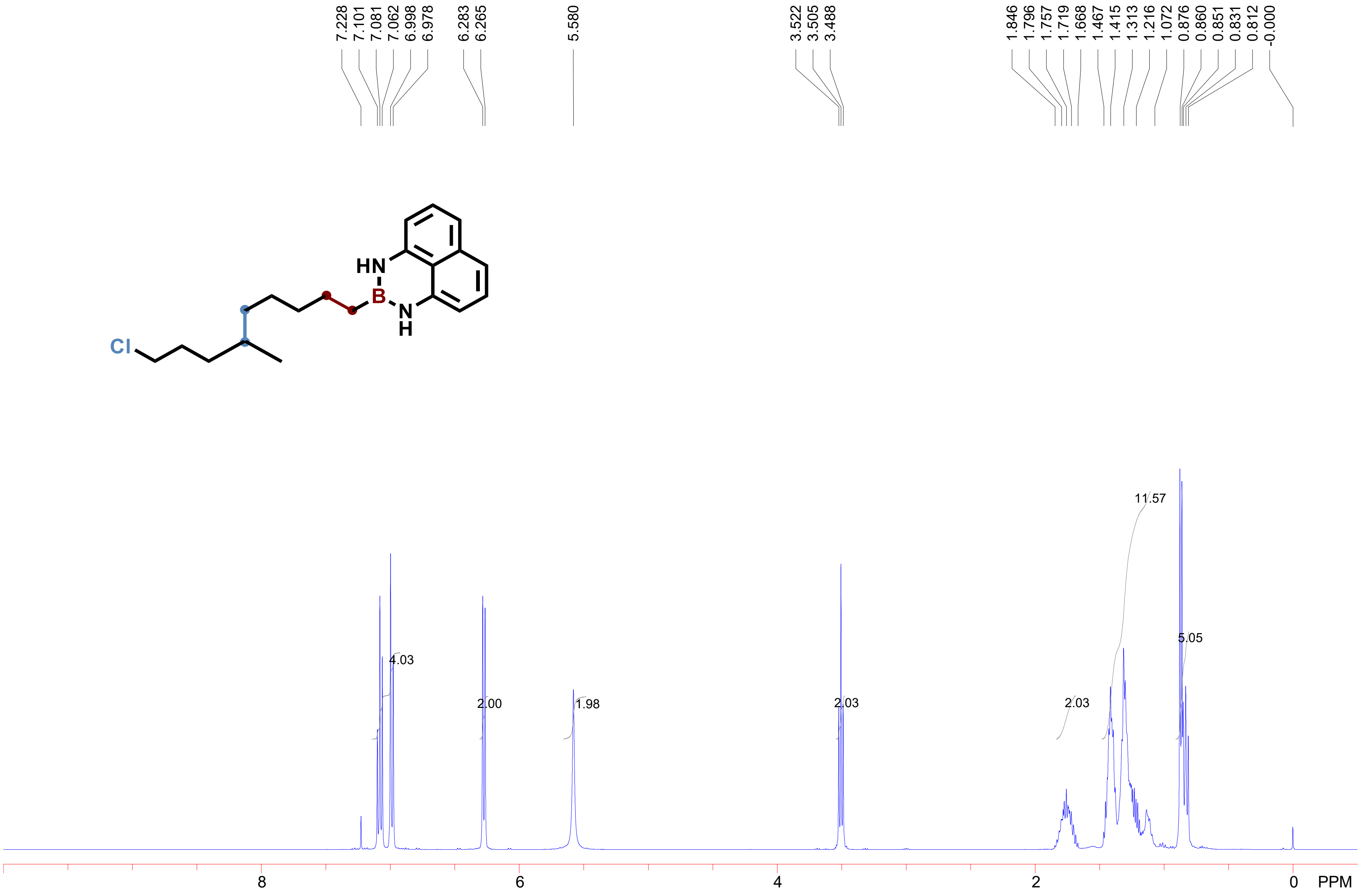

**$^{13}$C NMR-spectrum (100 MHz, CDCl$_3$) of 21**

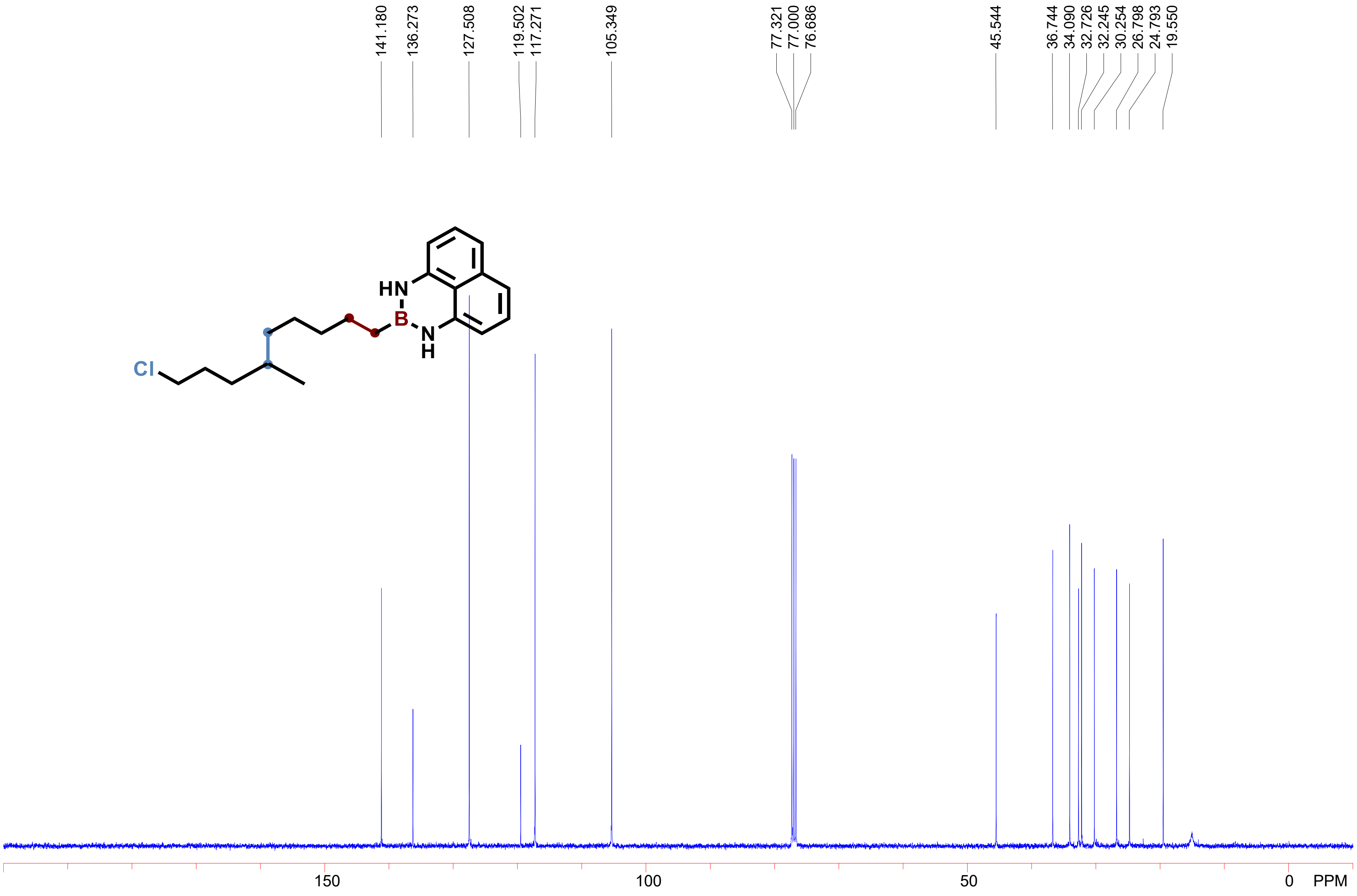

**$^{1}$H NMR-spectrum (400 MHz, CDCl₃) of 22**

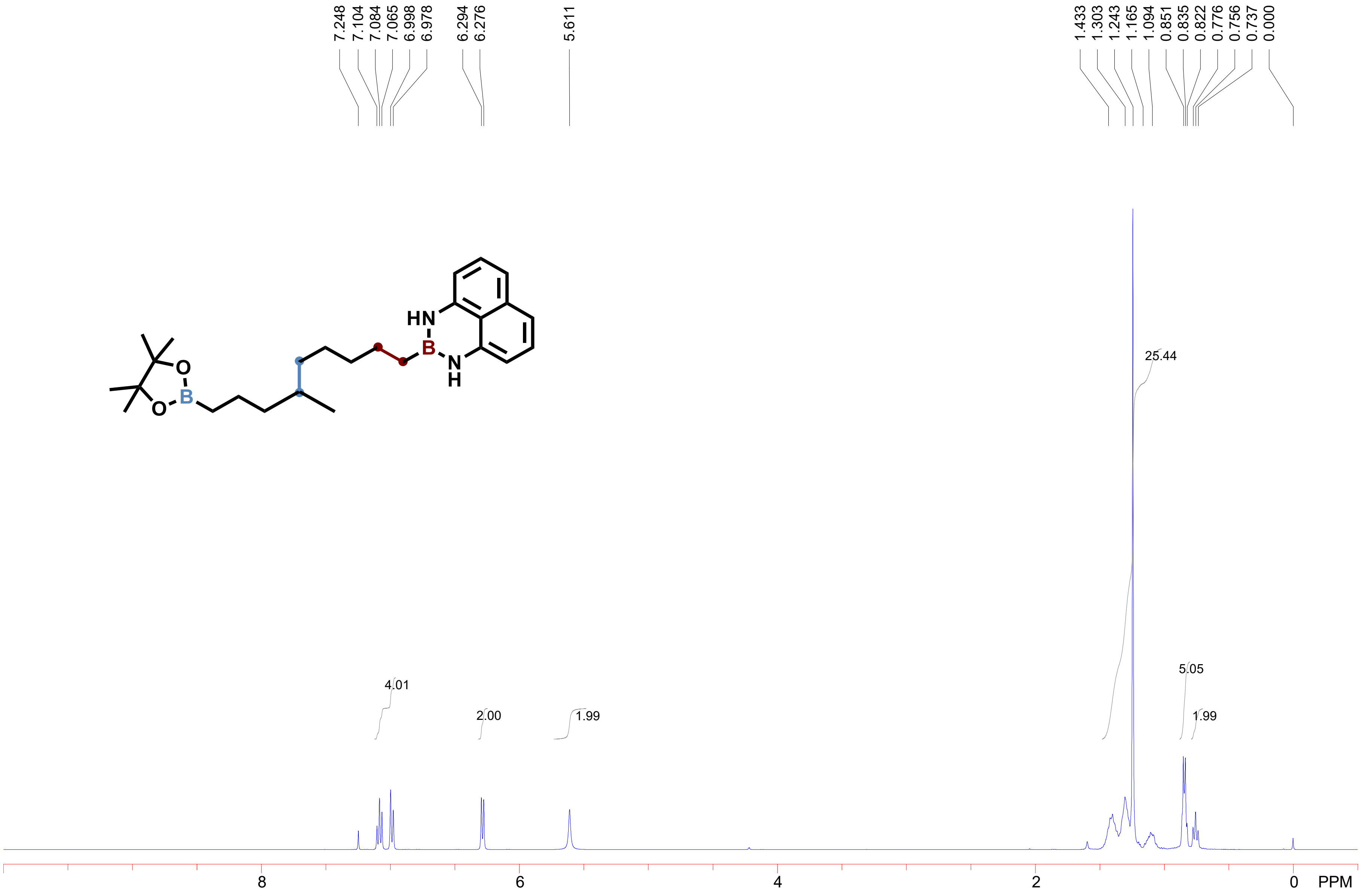

**$^{13}$C NMR-spectrum (100 MHz, $CDCl_3$) of 22**

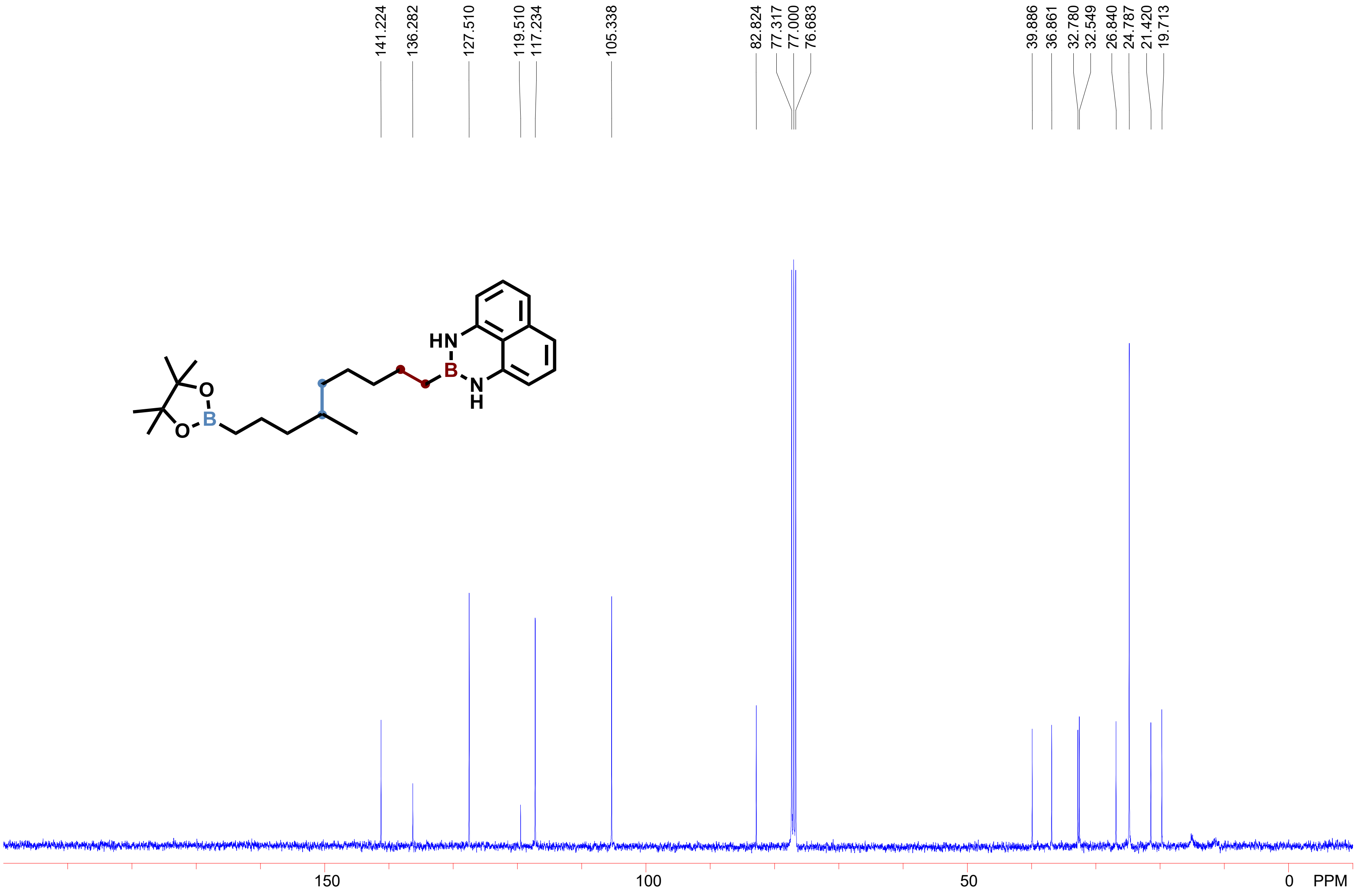

## $^{1}$H NMR-spectrum (400 MHz, $CDCl_3$) of 23

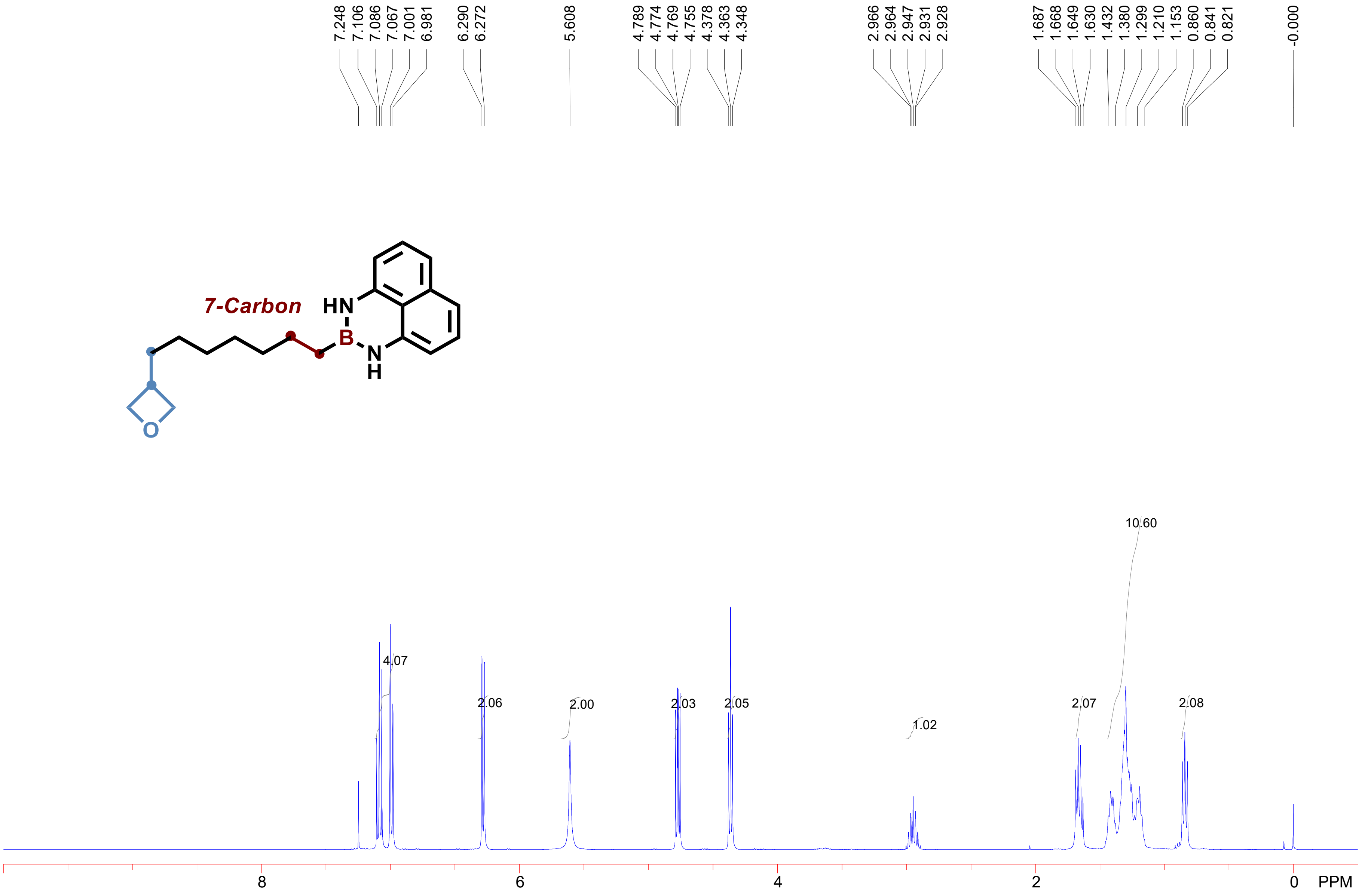

$^{13}C$ NMR-spectrum (100 MHz, $CDCl_3$) of 23

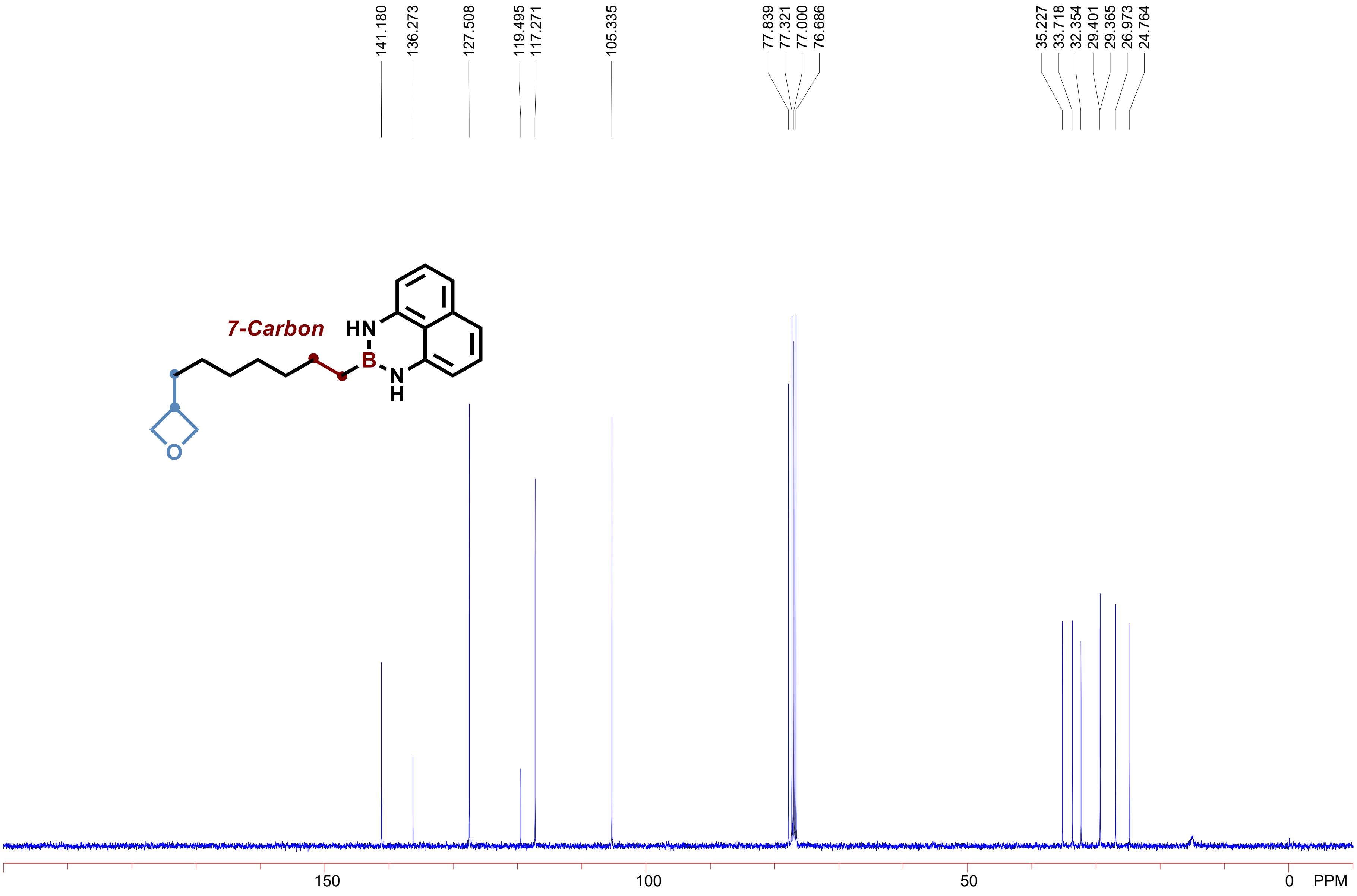

**$^{1}$H NMR-spectrum (400 MHz, CDCl₃) of 24**

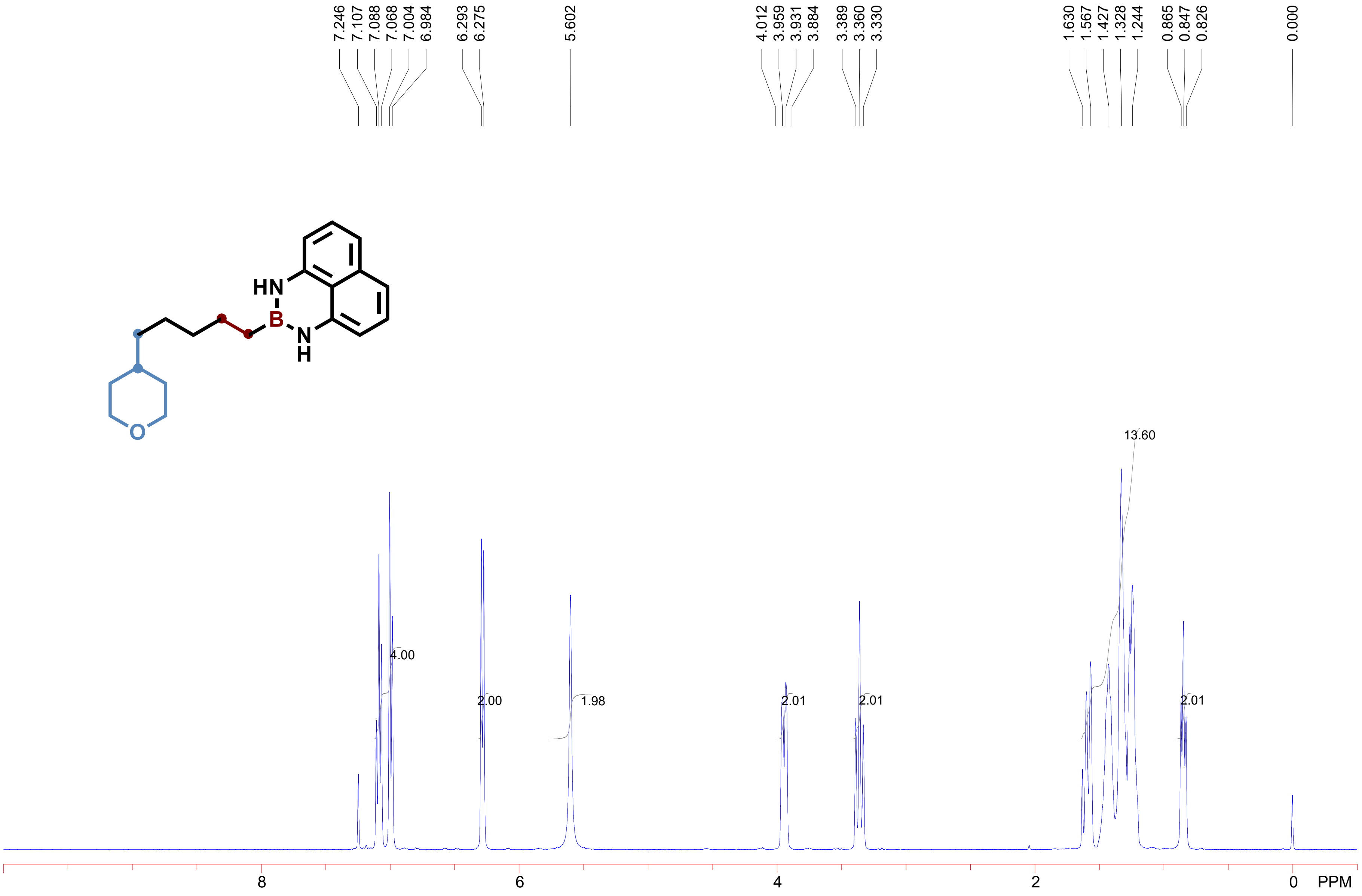

$^{13}$C NMR-spectrum (100 MHz, CDCl3) of 24

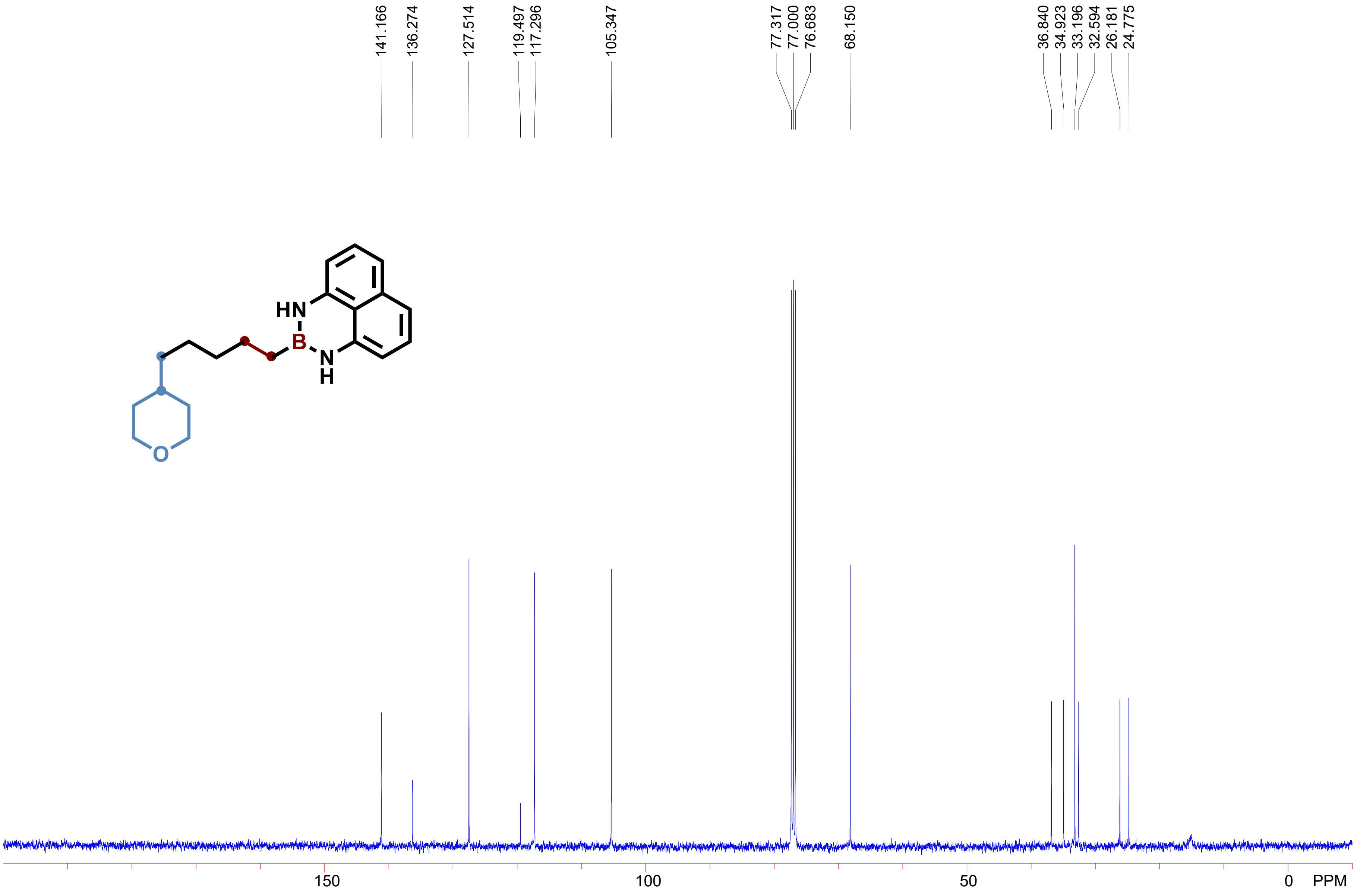

$^{1}$H NMR-spectrum (400 MHz, $CDCl_3$) of 25

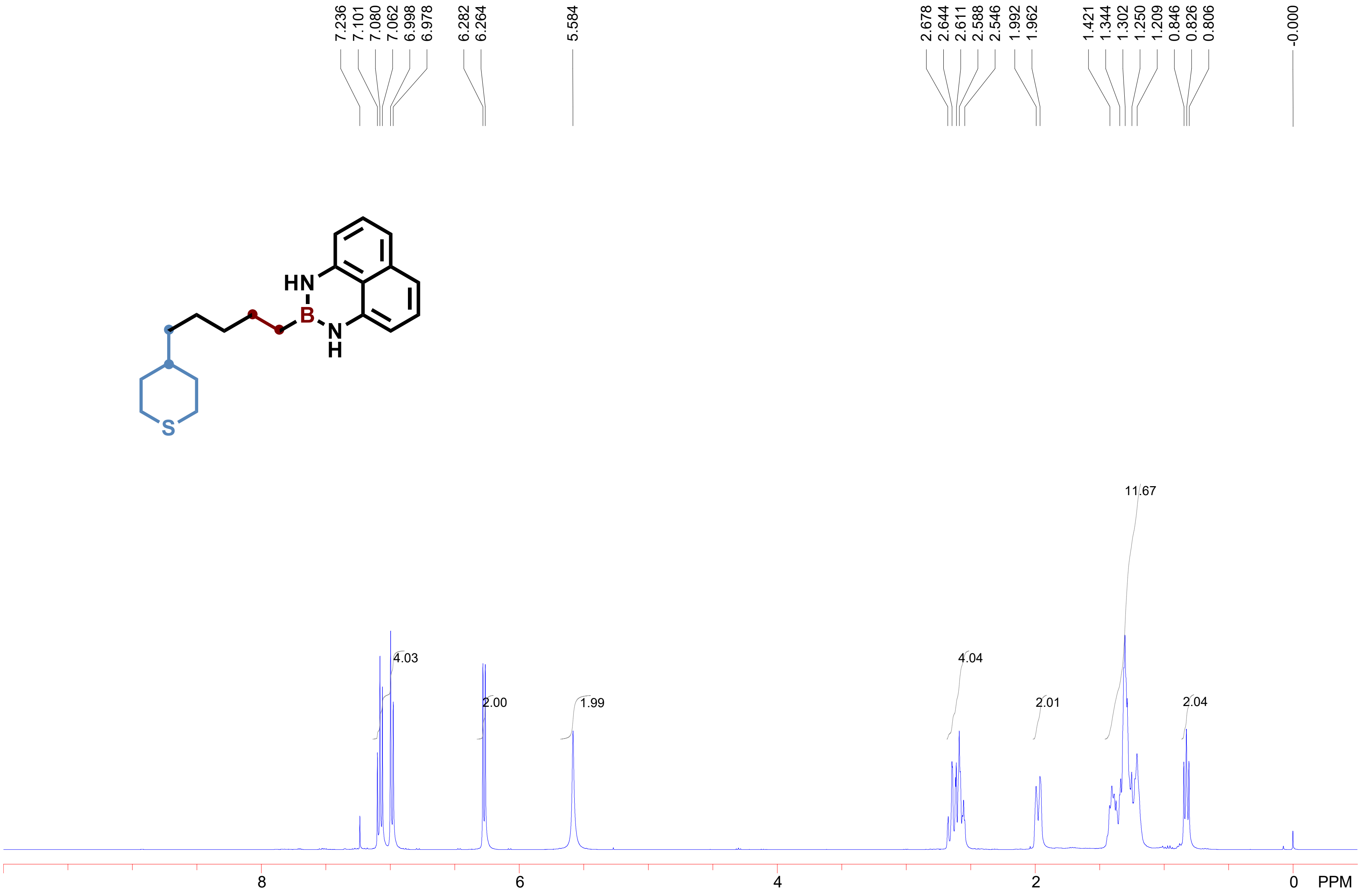

**$^{13}$C NMR-spectrum (100 MHz, $CDCl_3$) of 25**

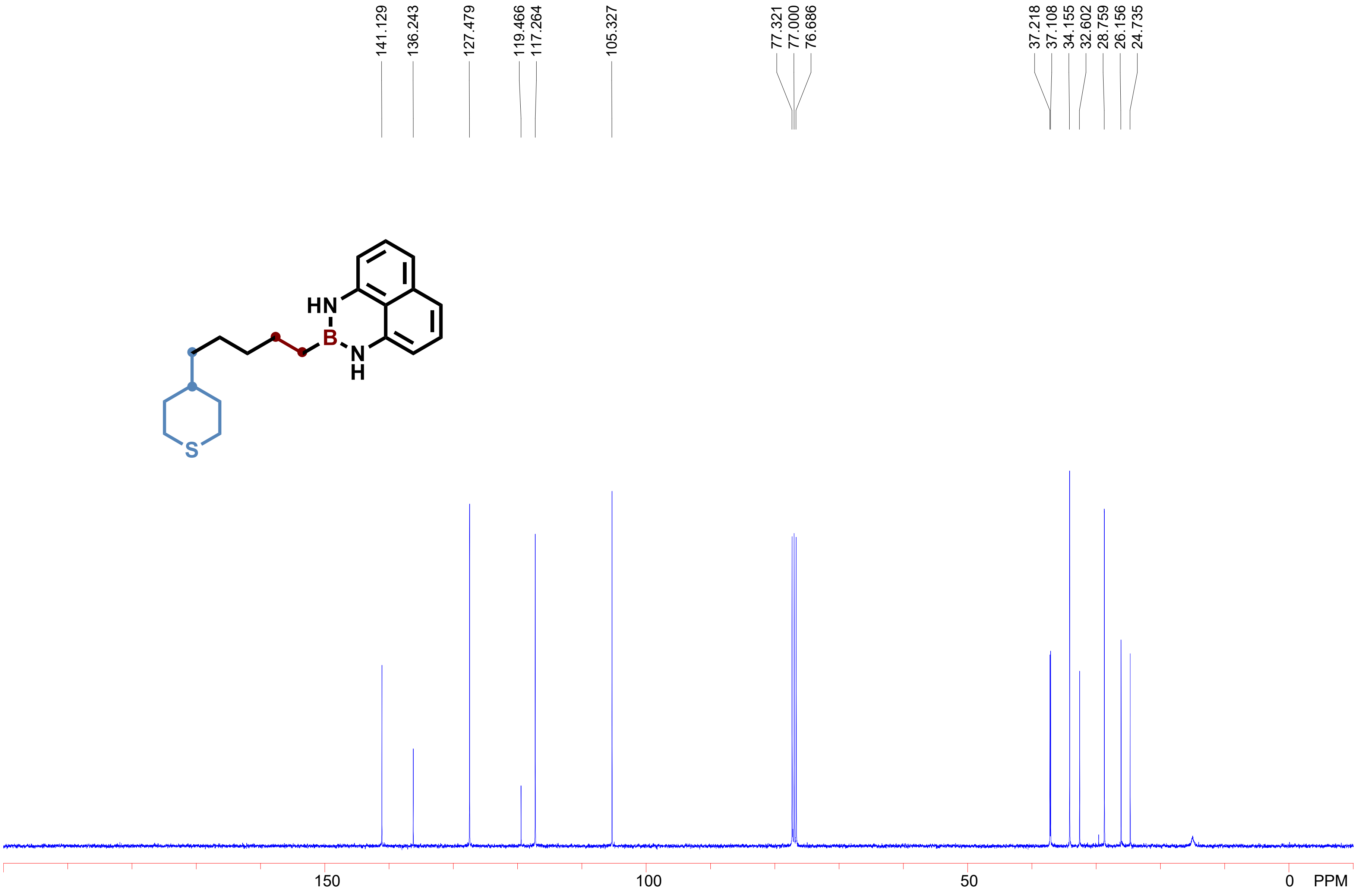

**$^{1}$H NMR-spectrum (400 MHz, CDCl3) of 26**

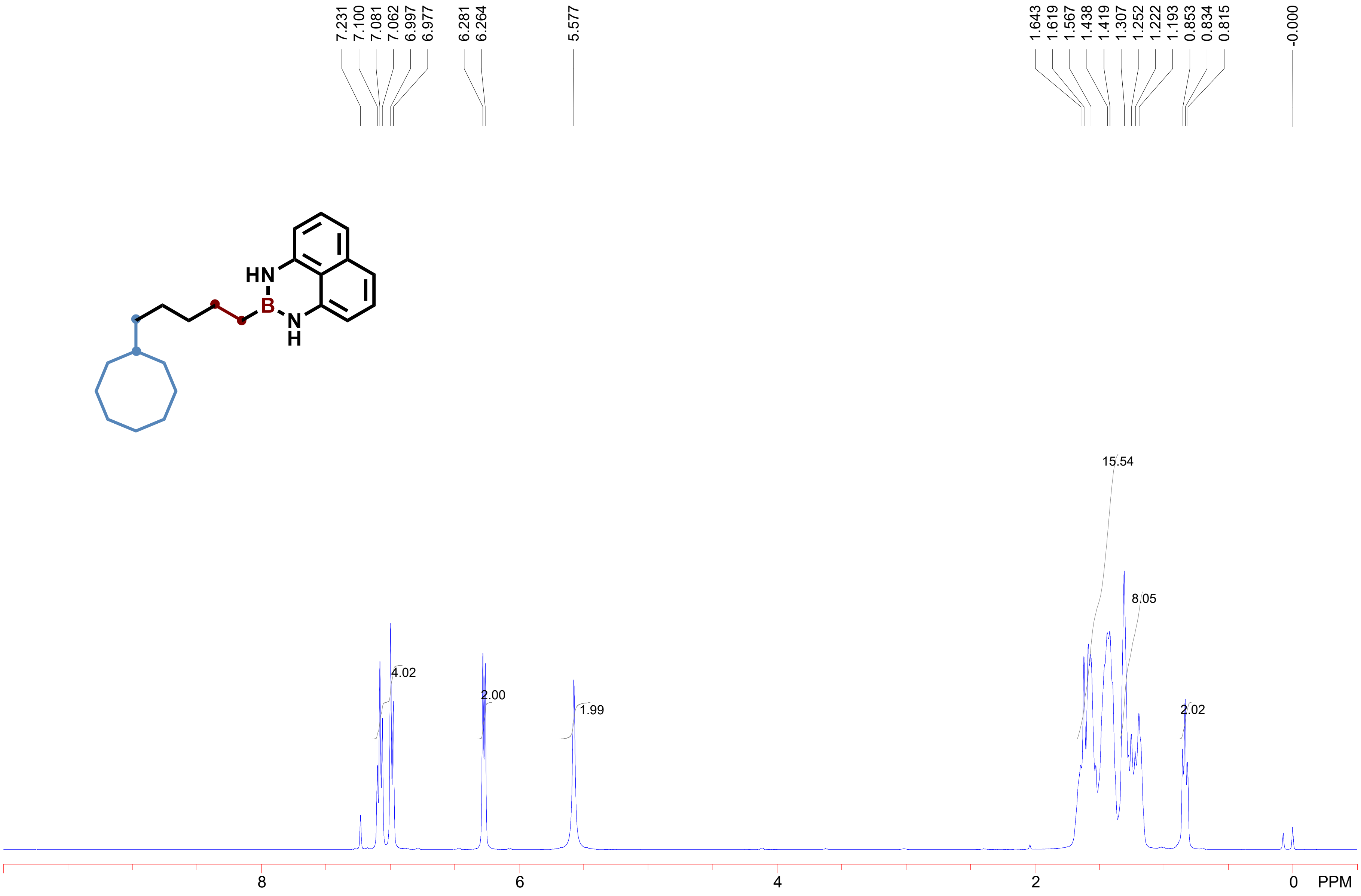

**$^{13}$C NMR-spectrum (100 MHz, CDCl₃) of 26**

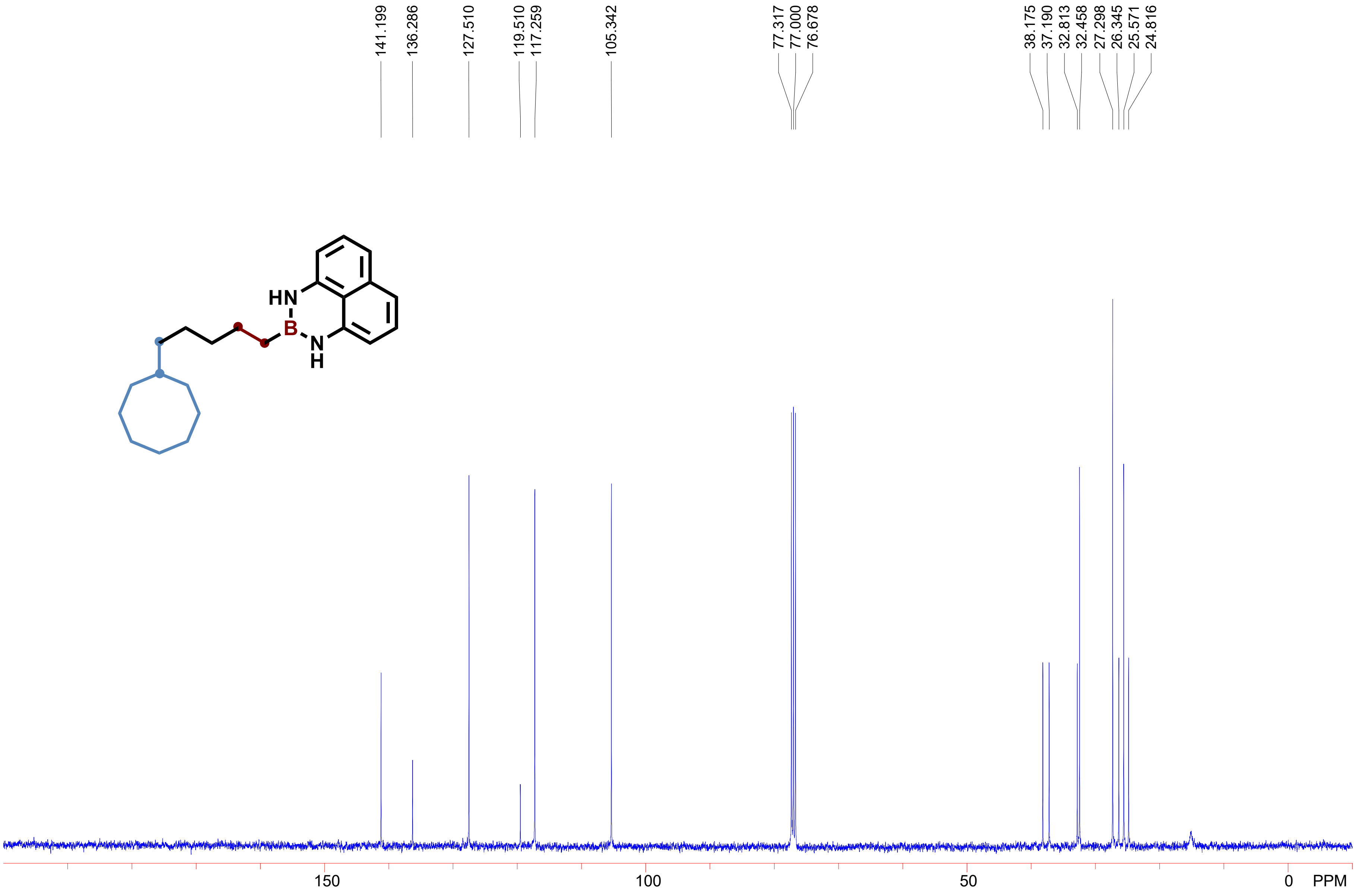

**$^{1}$H NMR-spectrum (400 MHz, CDCl3) of 27**

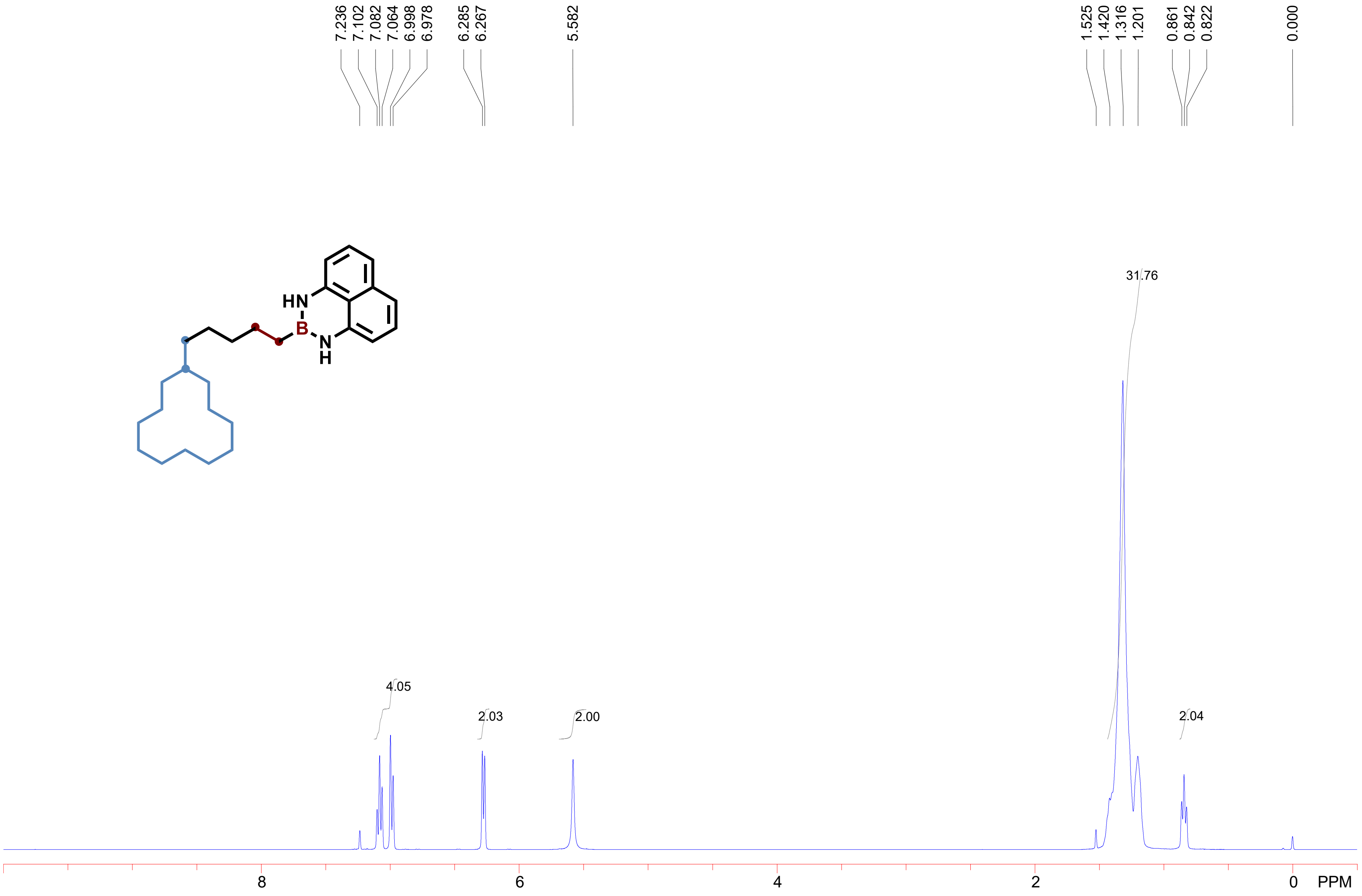

**$^{13}$C NMR-spectrum (100 MHz, $CDCl_3$) of 27**

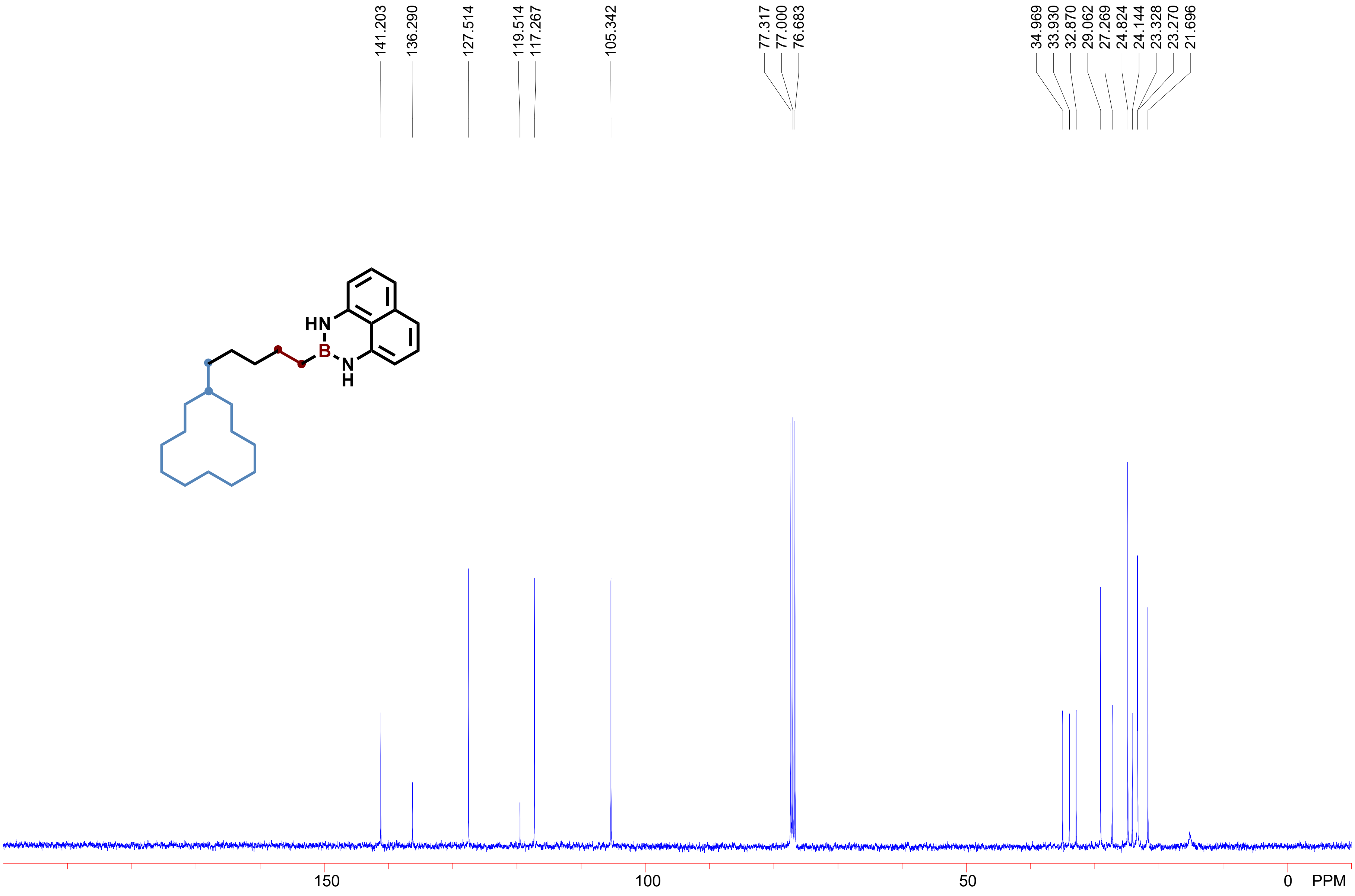

$^{1}$H NMR-spectrum (400 MHz, CDCl3) of 28

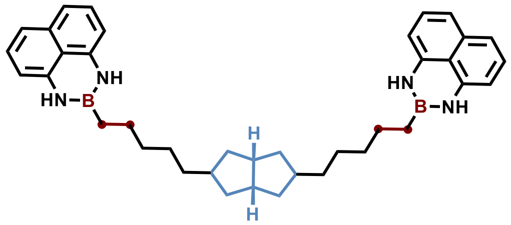


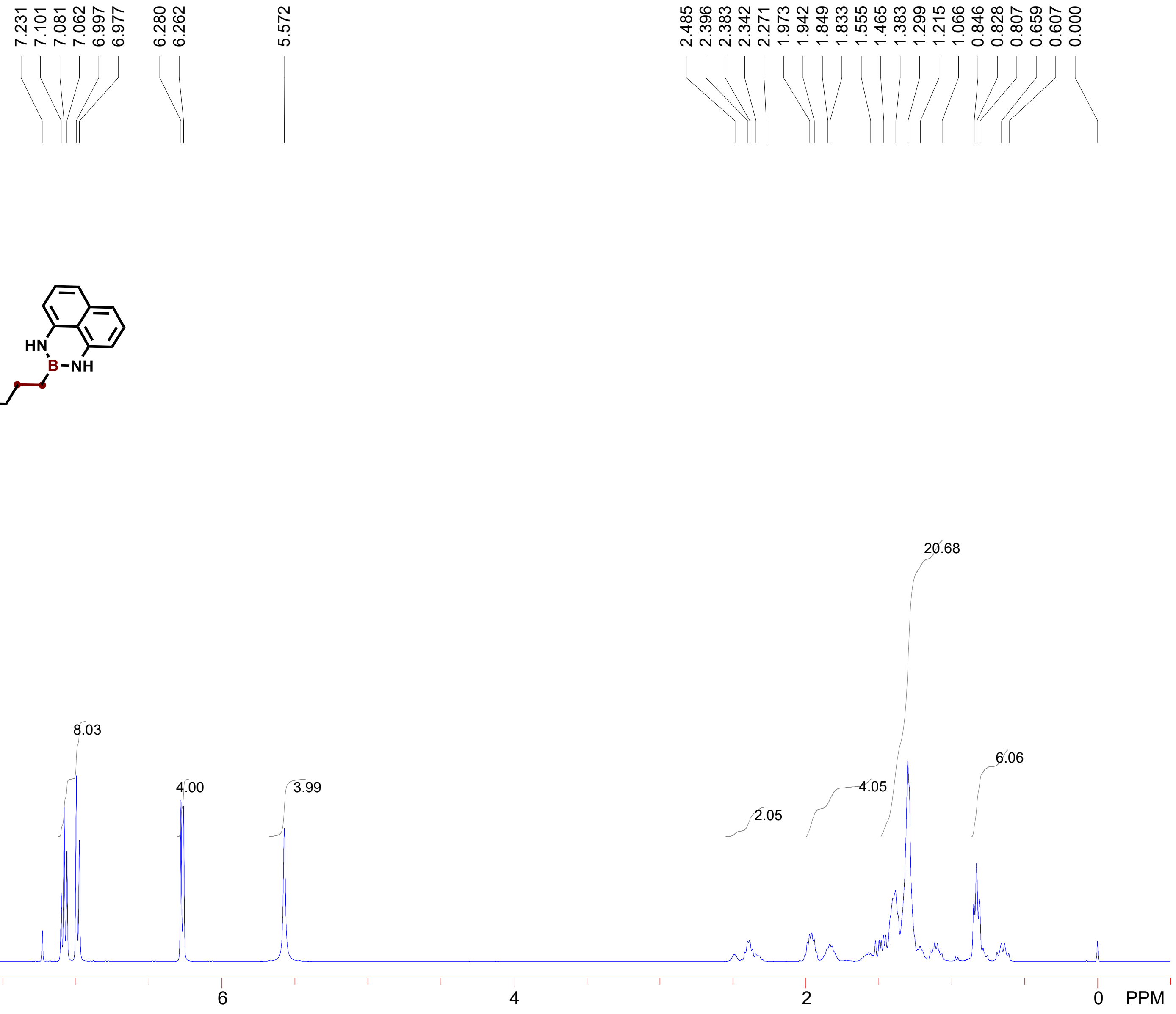

**$^{13}C$ NMR-spectrum (100 MHz, $CDCl_3$) of 28**

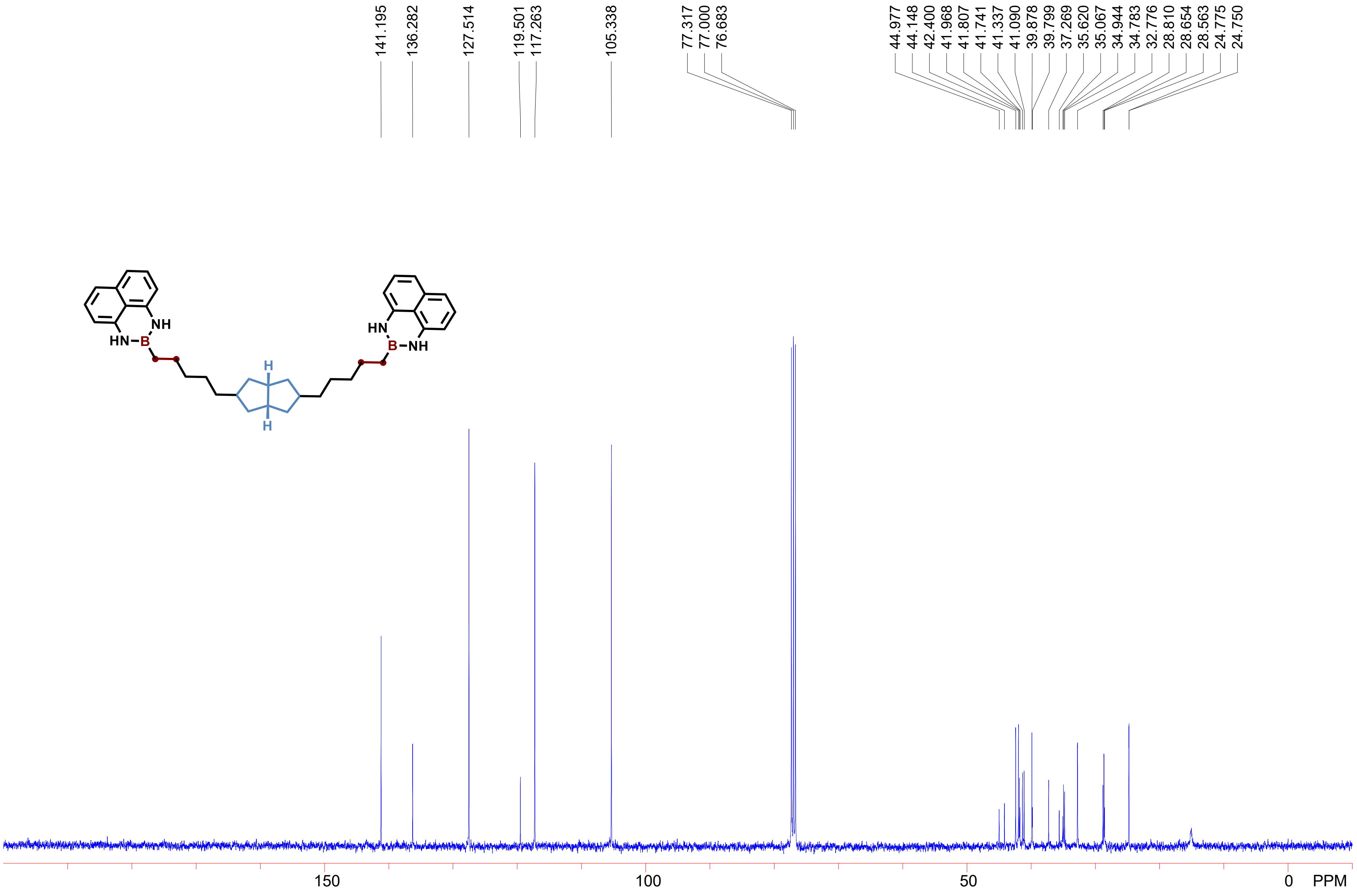

## $^{1}$H NMR-spectrum (400 MHz, $CDCl_3$) of 29

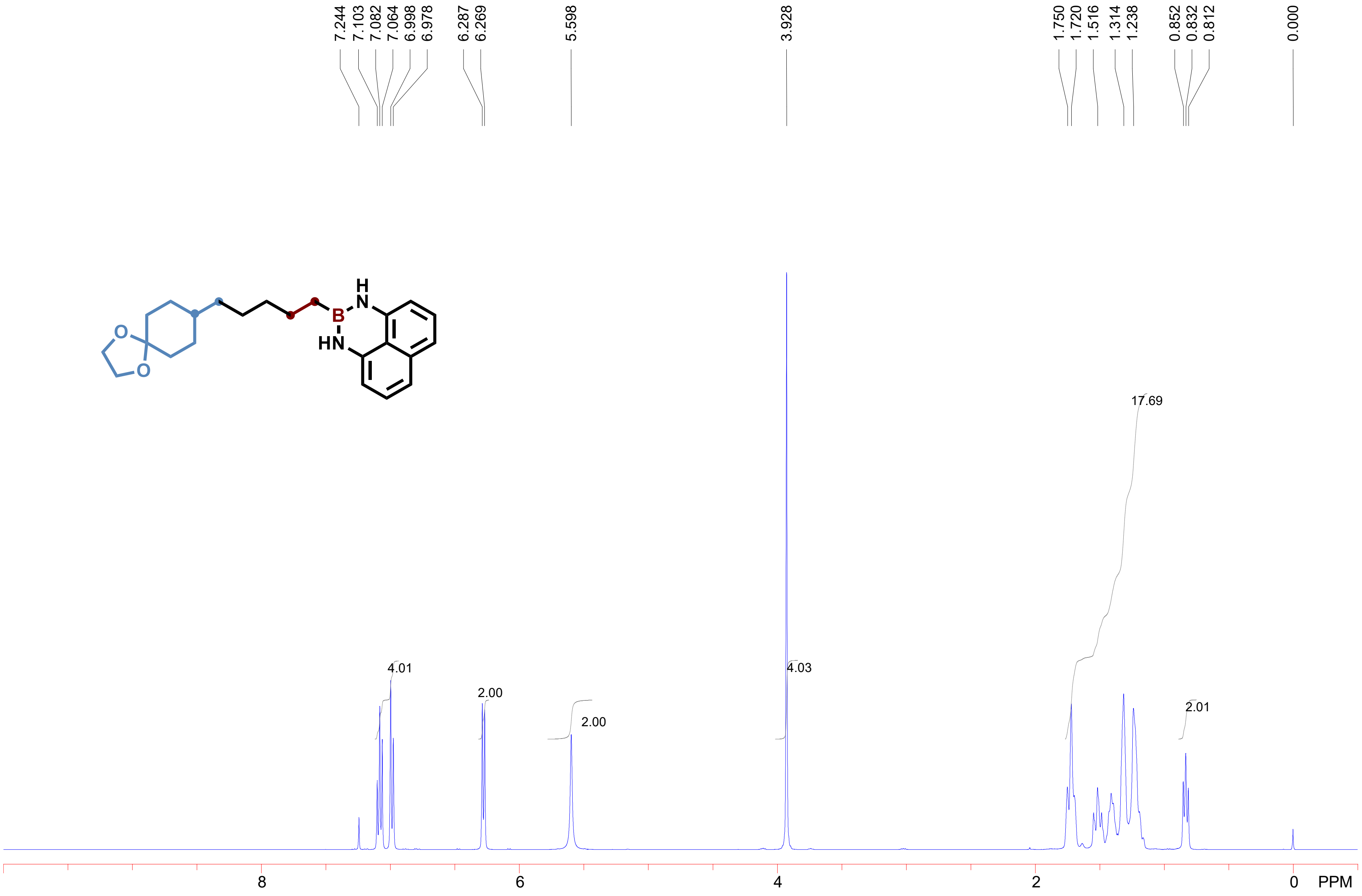

$^{13}C$ NMR-spectrum (100 MHz, $CDCl_3$) of 29

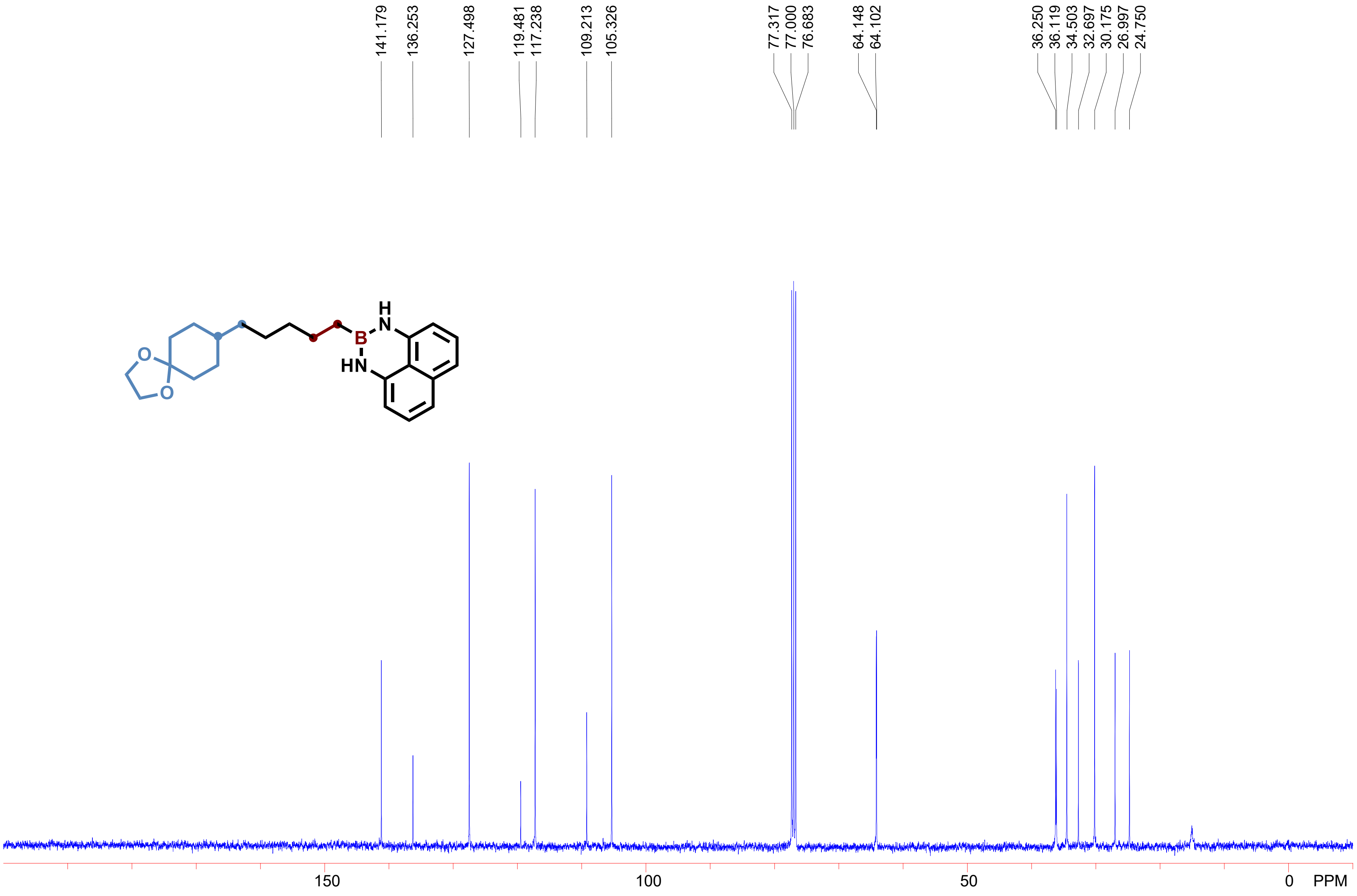

**$^{1}$H NMR-spectrum (400 MHz, CDCl$_3$) of 30**

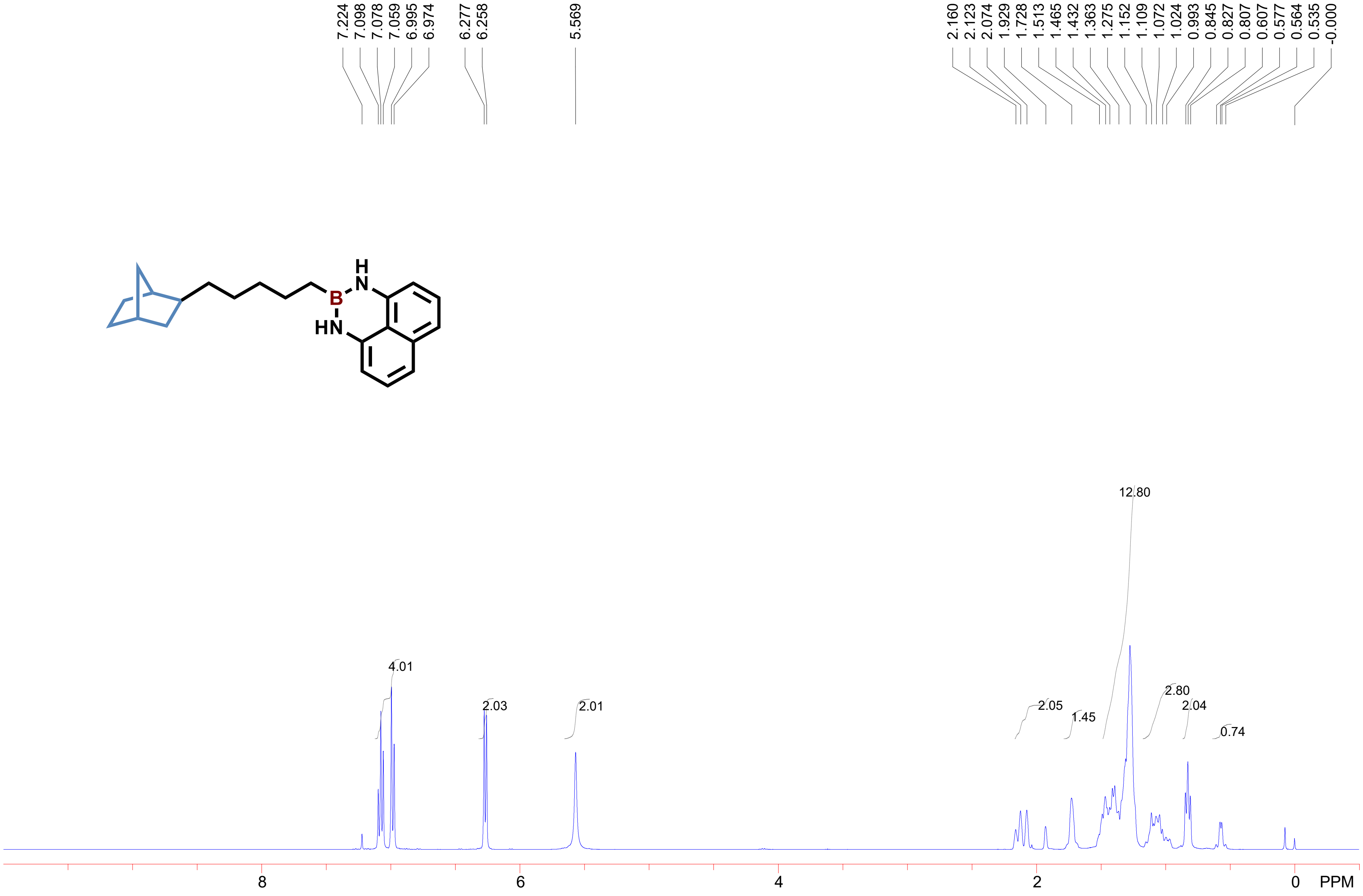

**$^{13}$C NMR-spectrum (100 MHz, $CDCl_3$) of 30**

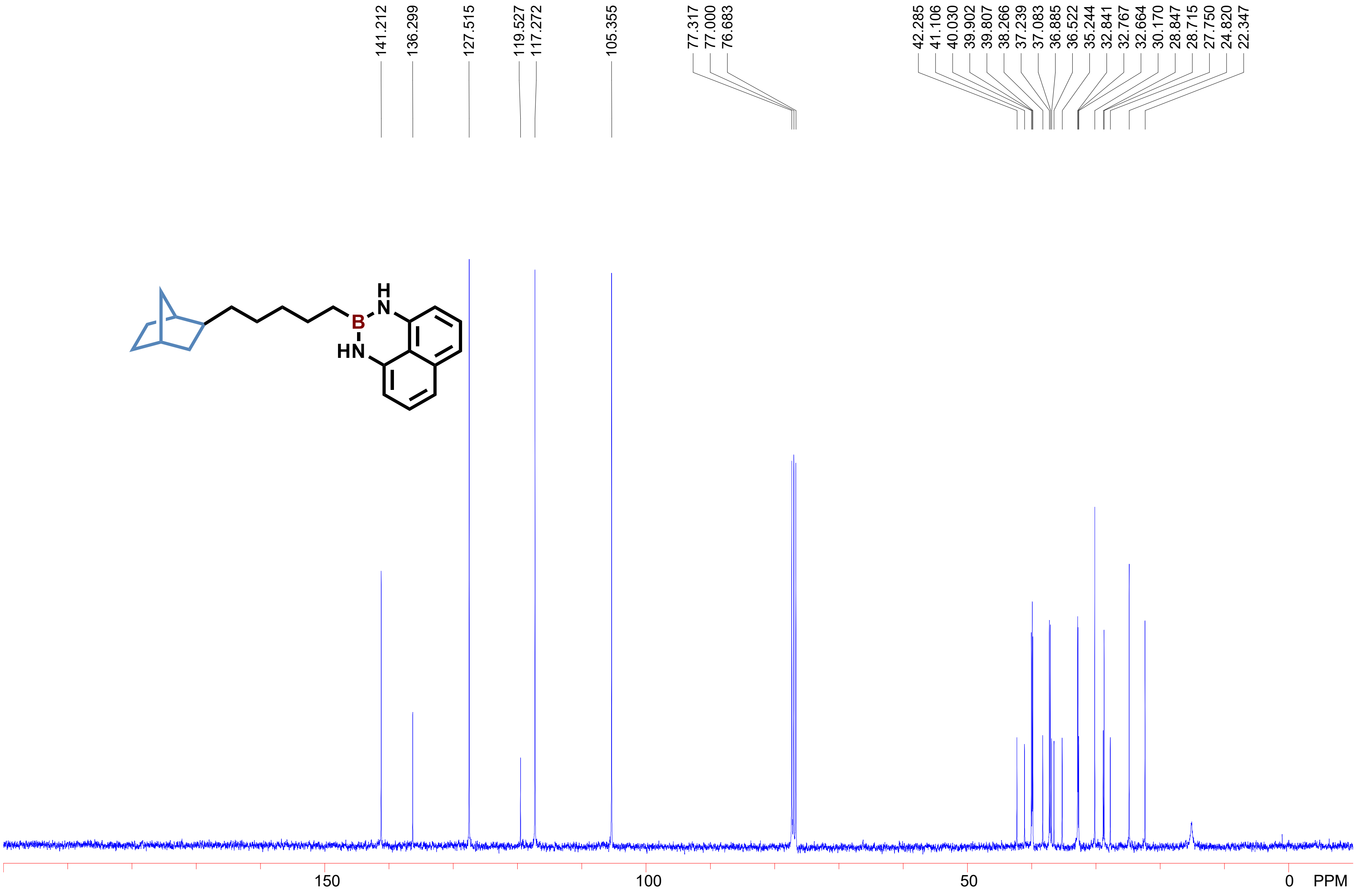

# $^{1}$H NMR-spectrum (400 MHz, CDCl3) of 31

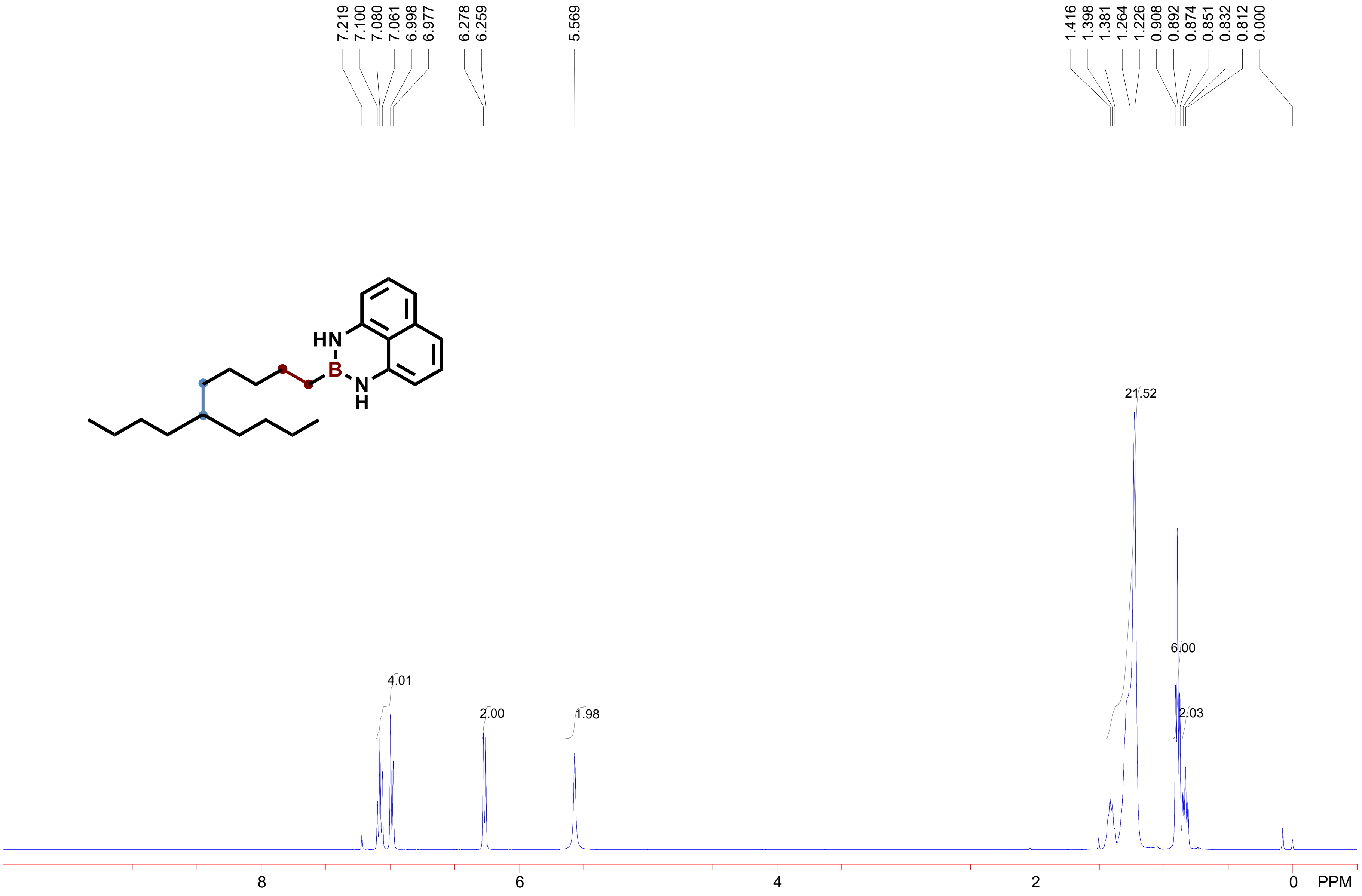

## $^{13}C$ NMR-spectrum (100 MHz, $CDCl_3$) of 31

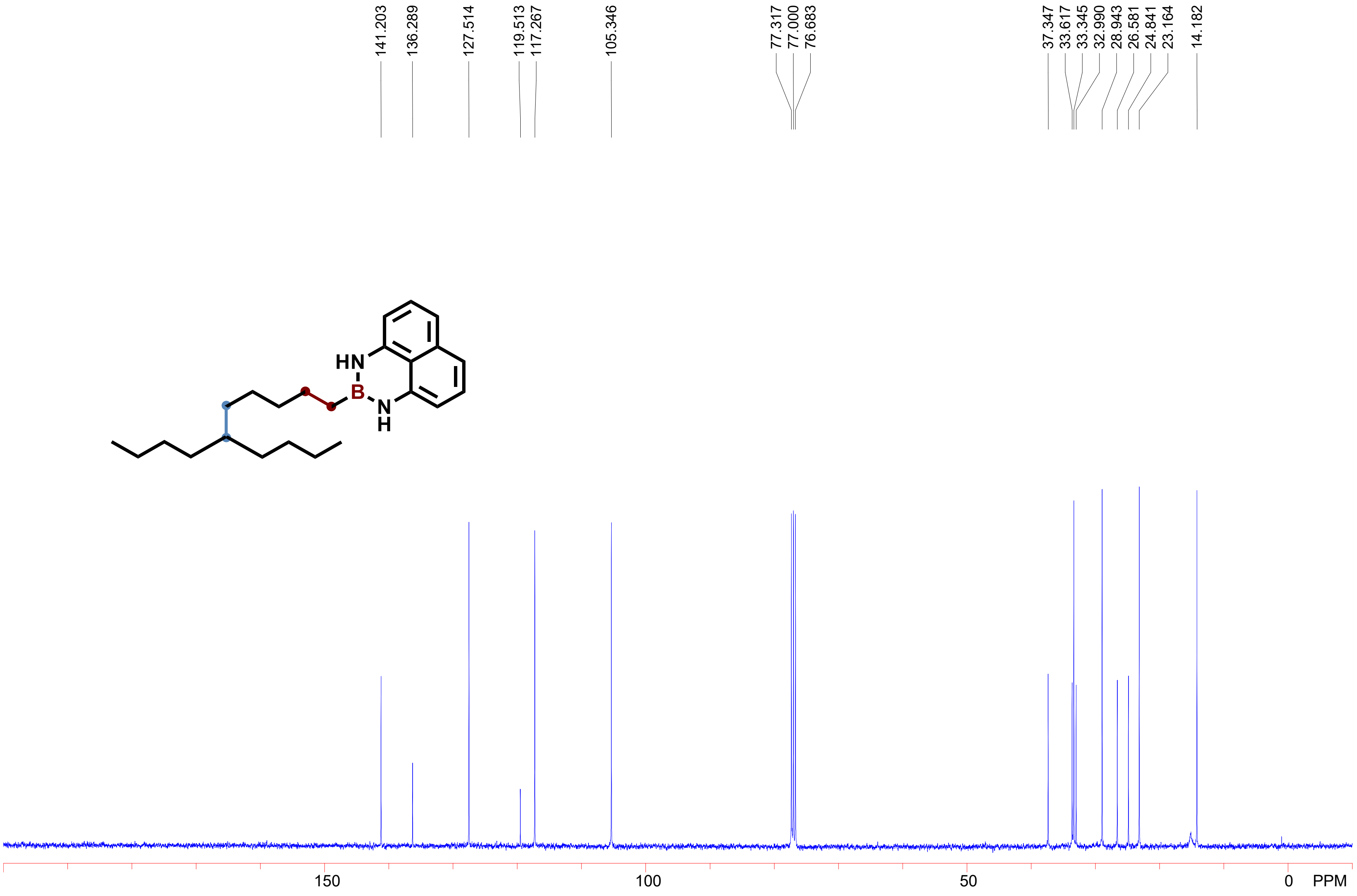

**$^{1}$H NMR-spectrum (400 MHz, $CDCl_3$) of 32**

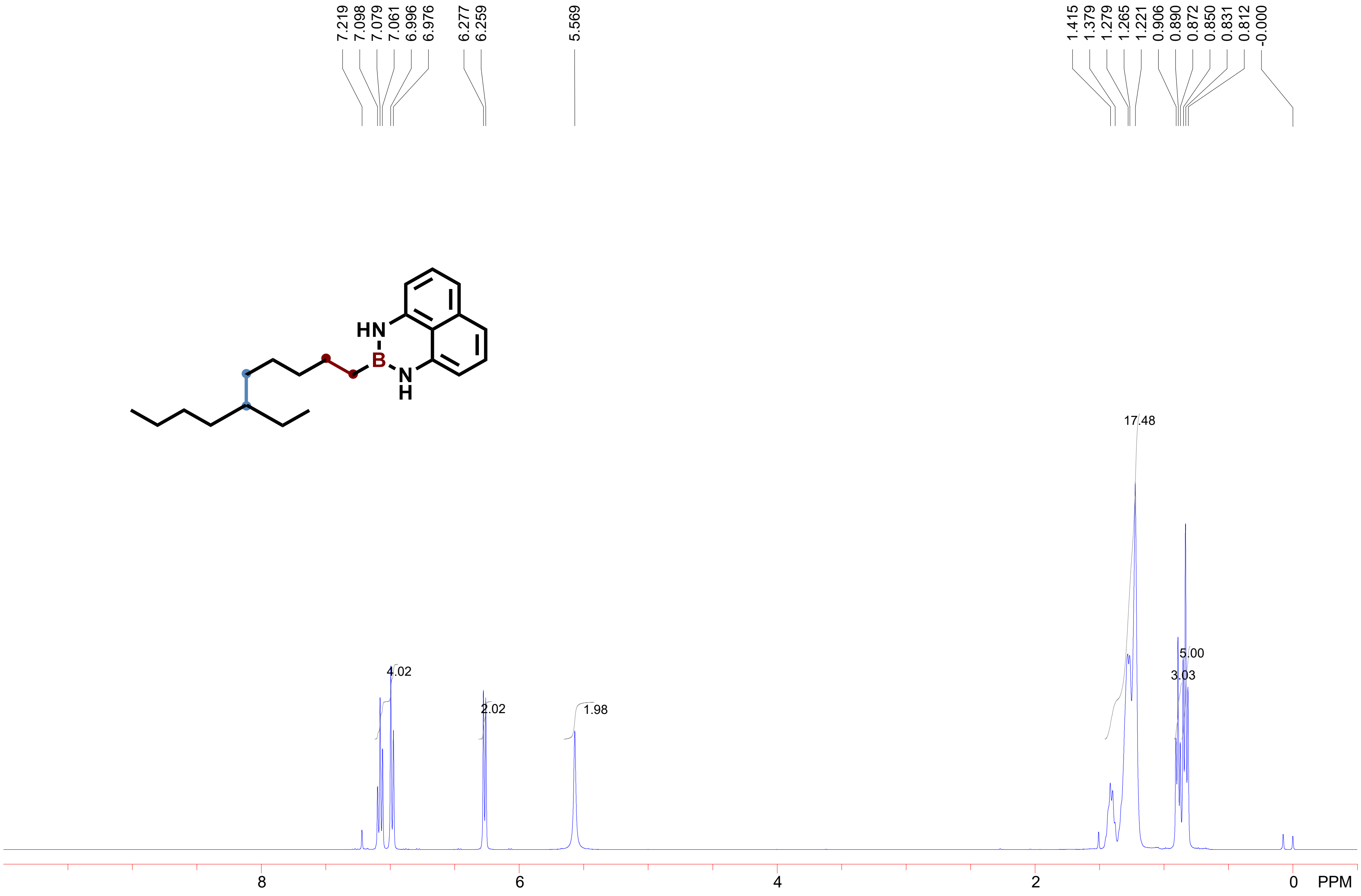

## $^{13}$C NMR-spectrum (100 MHz, $CDCl_3$) of 32

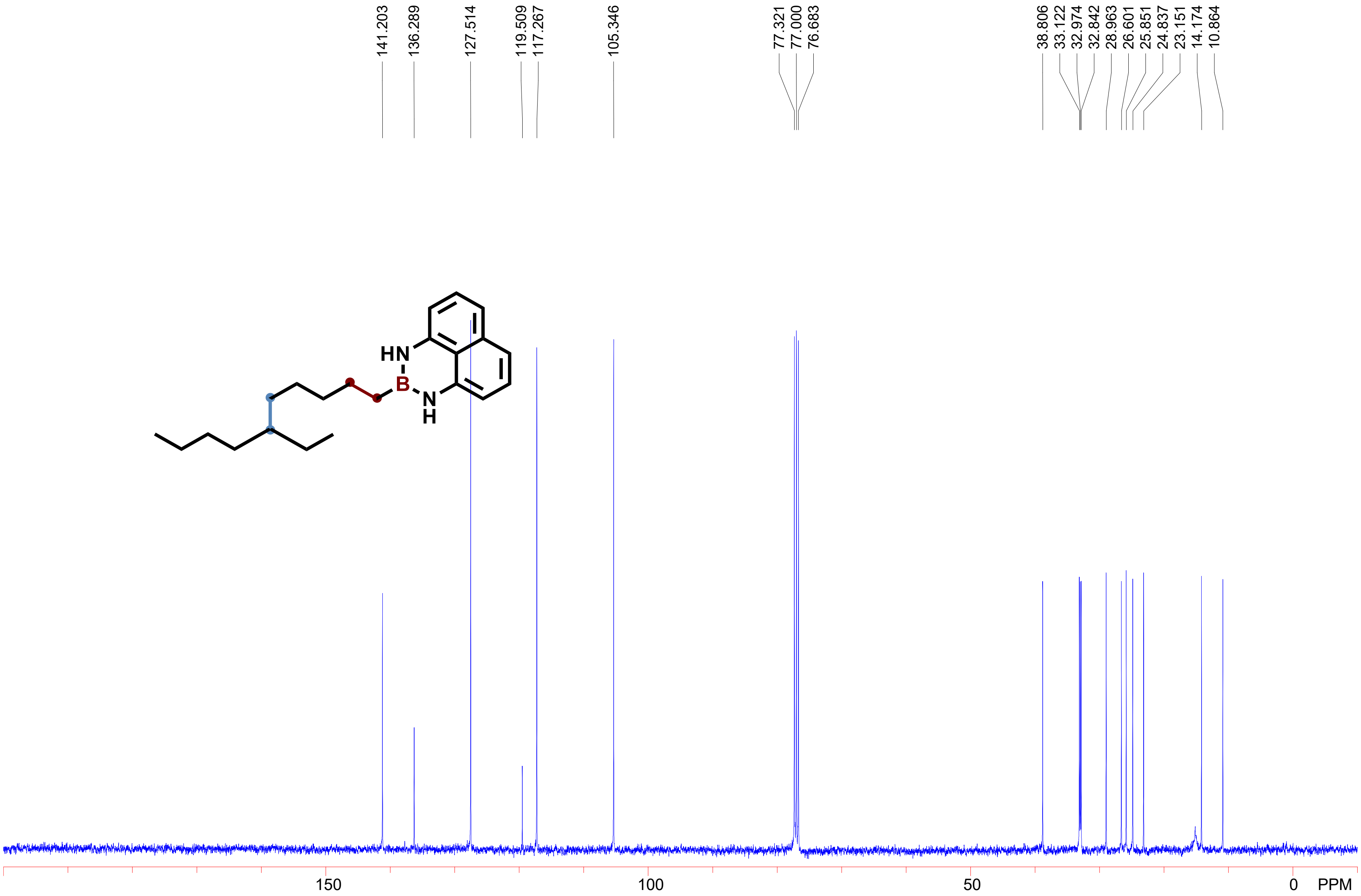

# $^1$H NMR-spectrum (400 MHz, $CDCl_3$) of 33

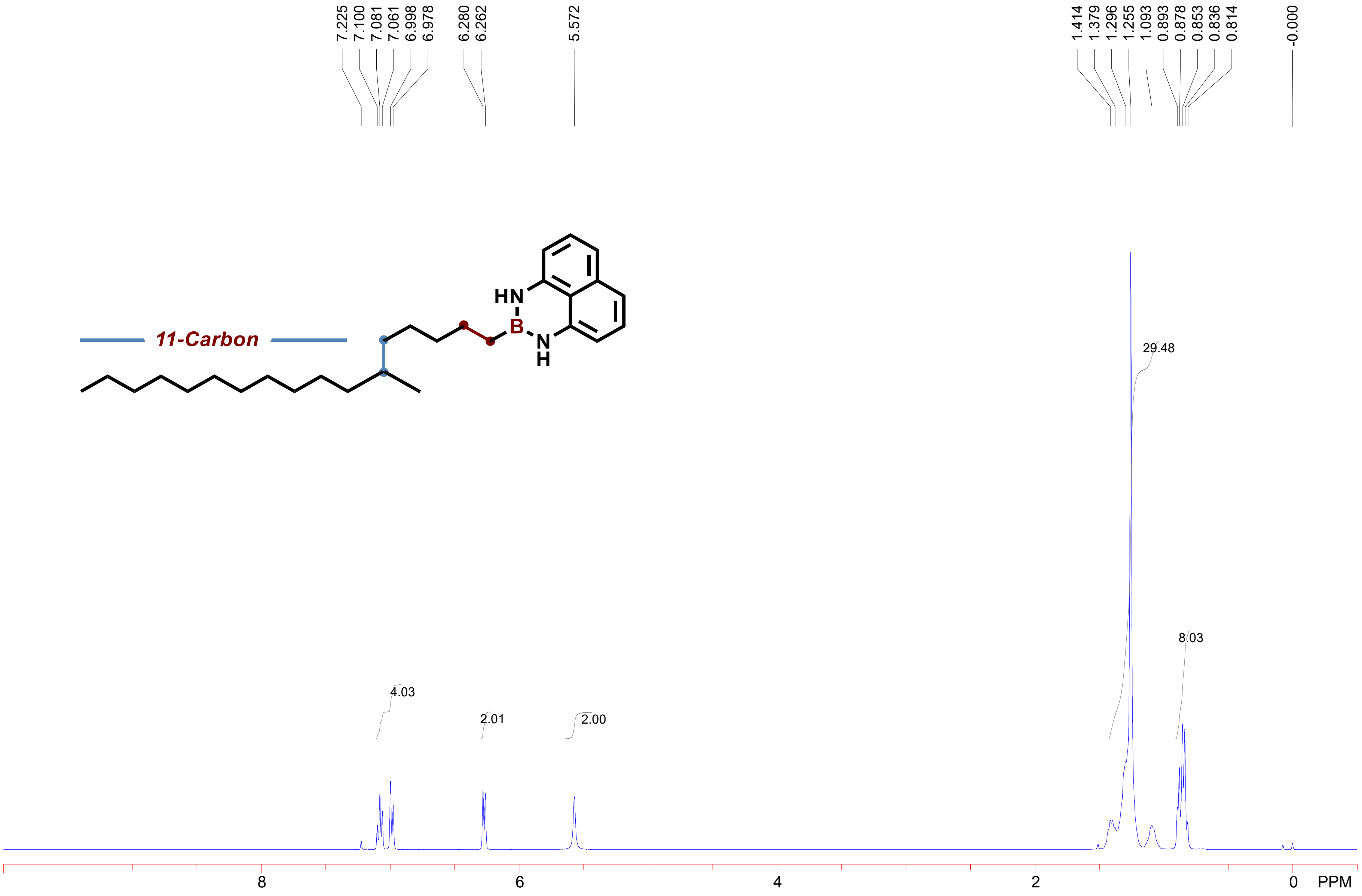

$^{13}$C NMR-spectrum (100 MHz, $CDCl_3$) of 33

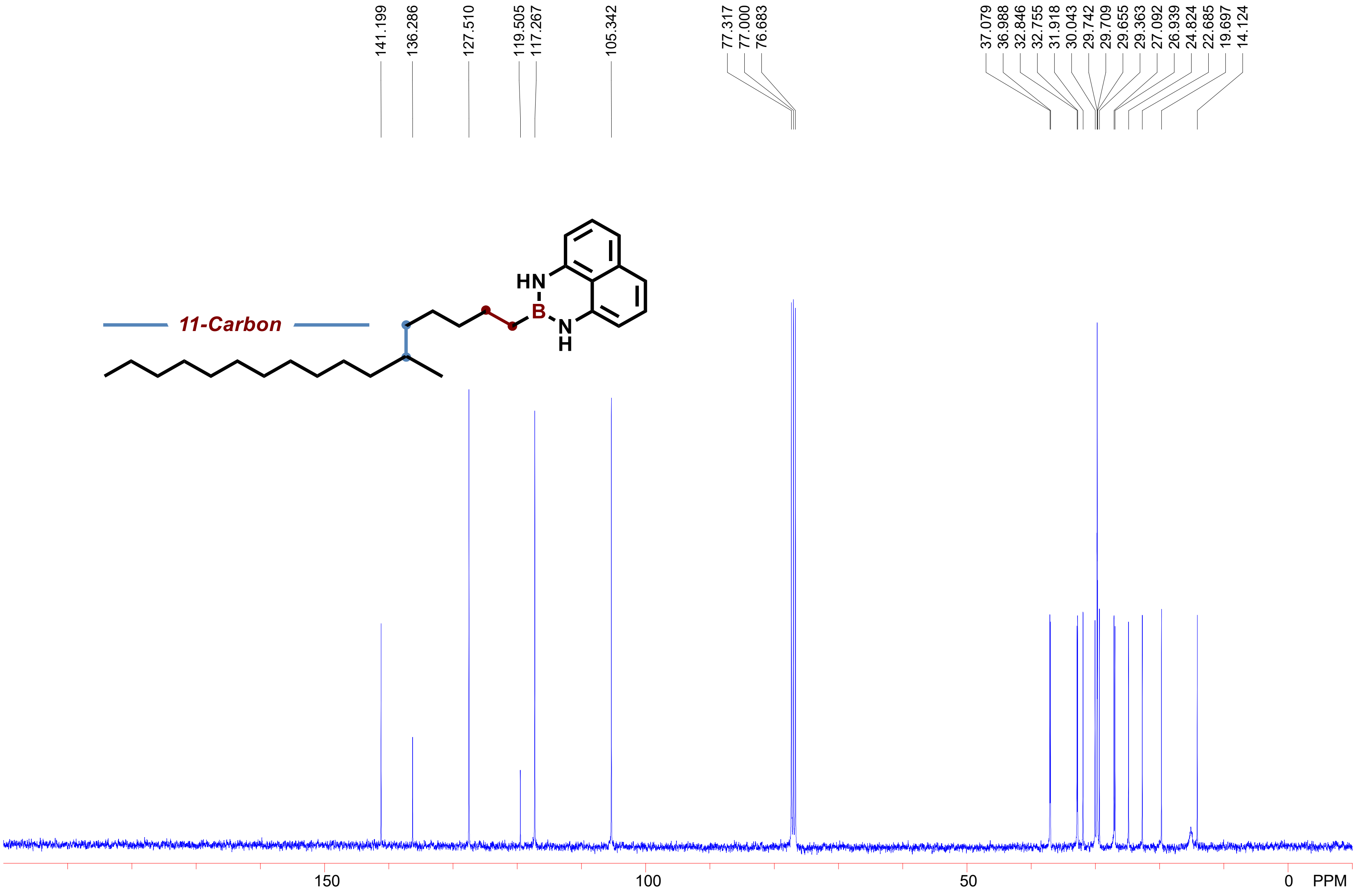

$^{1}$H NMR-spectrum (400 MHz, $CDCl_3$) of 34

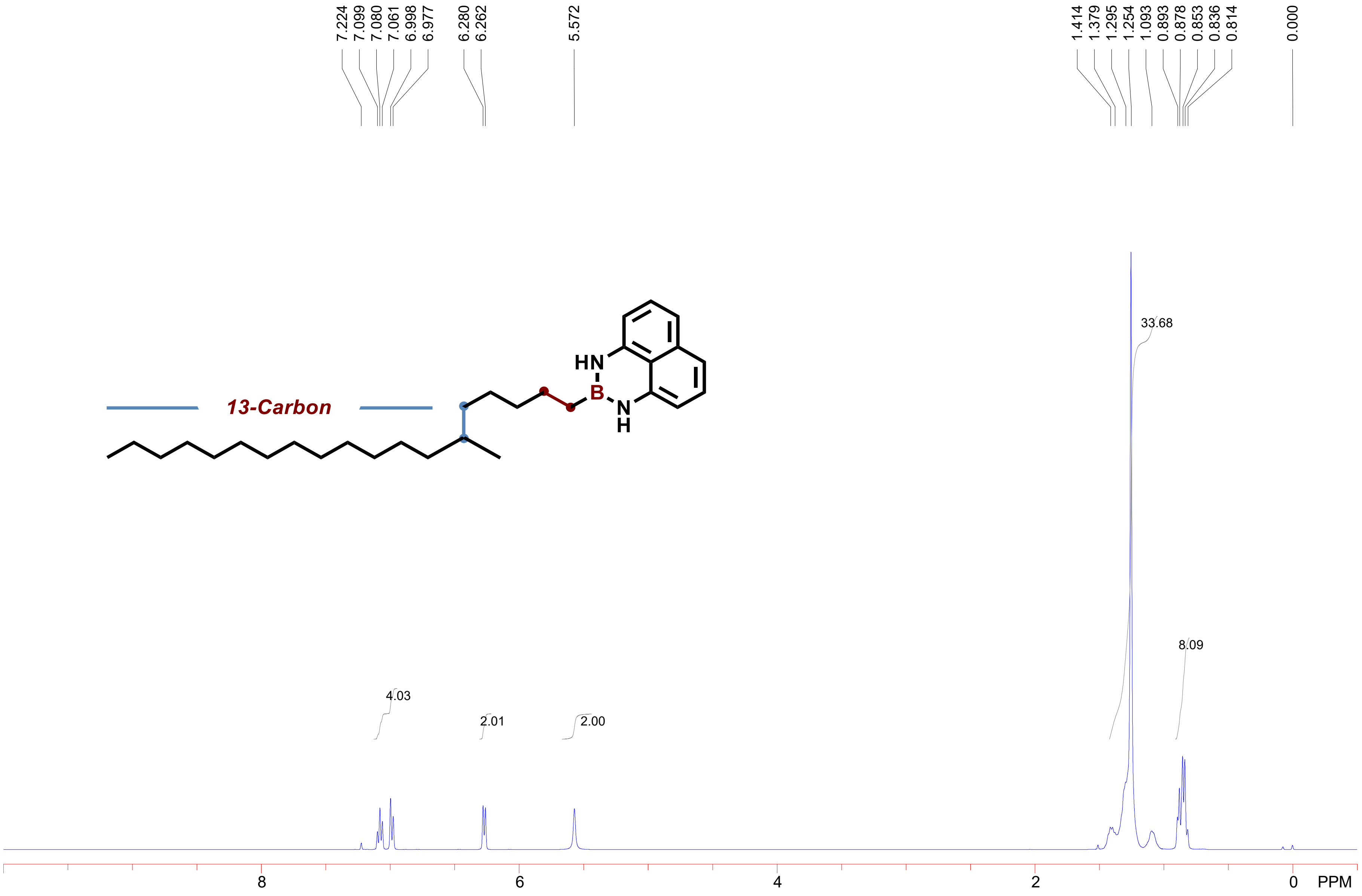

**$^{13}$C NMR-spectrum (100 MHz, CDCl₃) of 34**

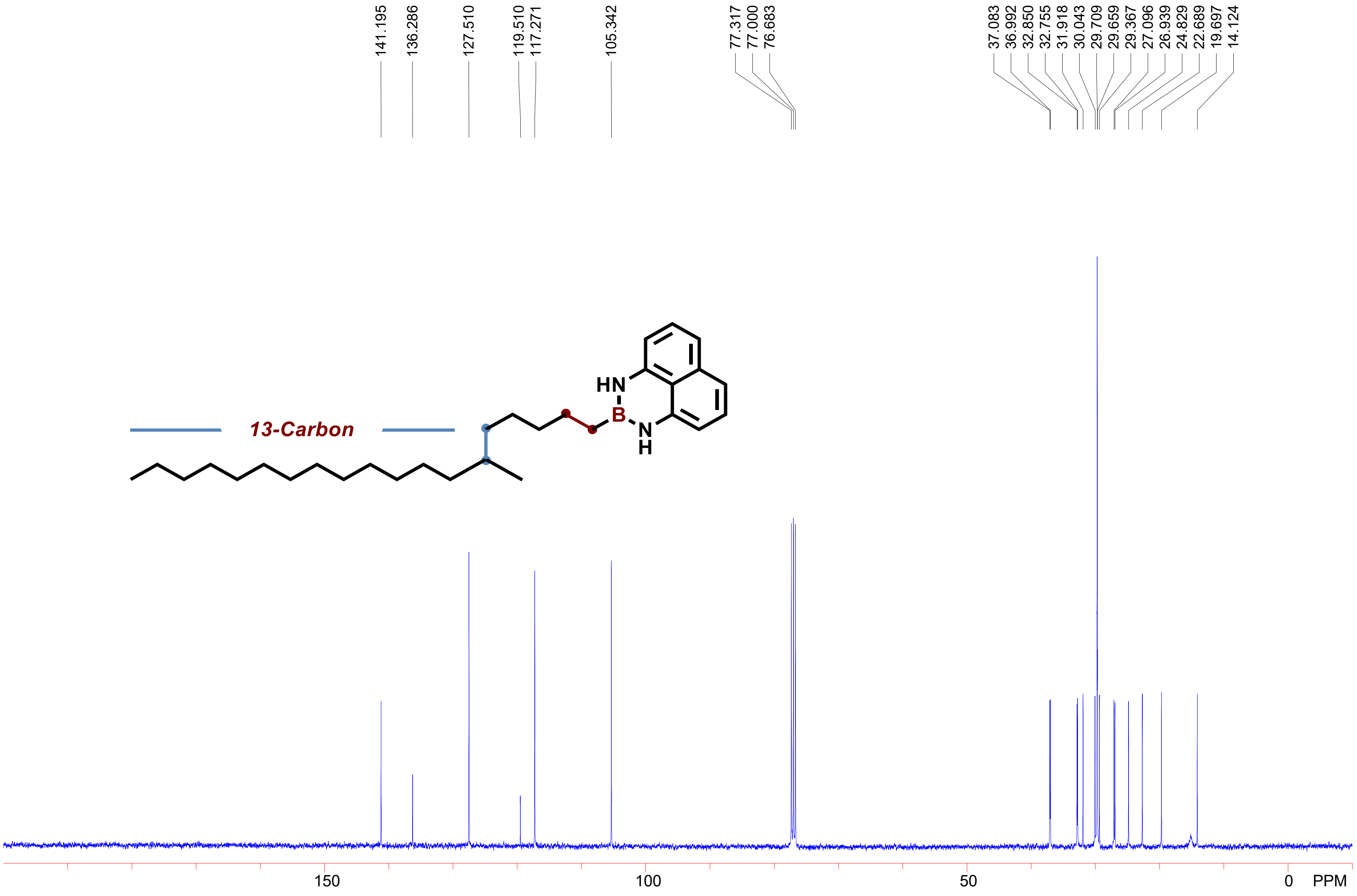

**$^{1}$H NMR-spectrum (400 MHz, CDCl$_3$) of 35**

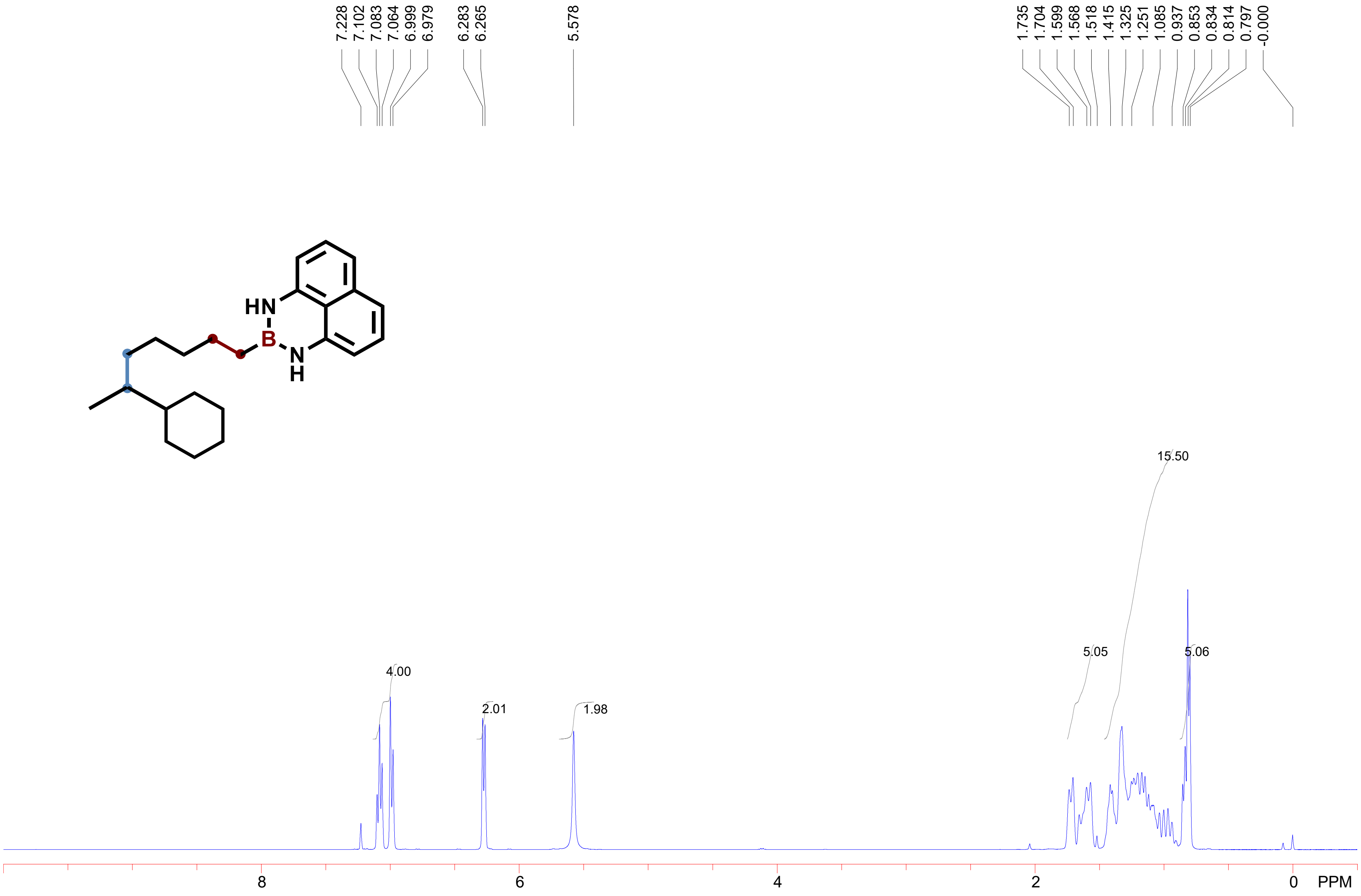

**$^{13}$C NMR-spectrum (100 MHz, CDCl$_3$) of 35**

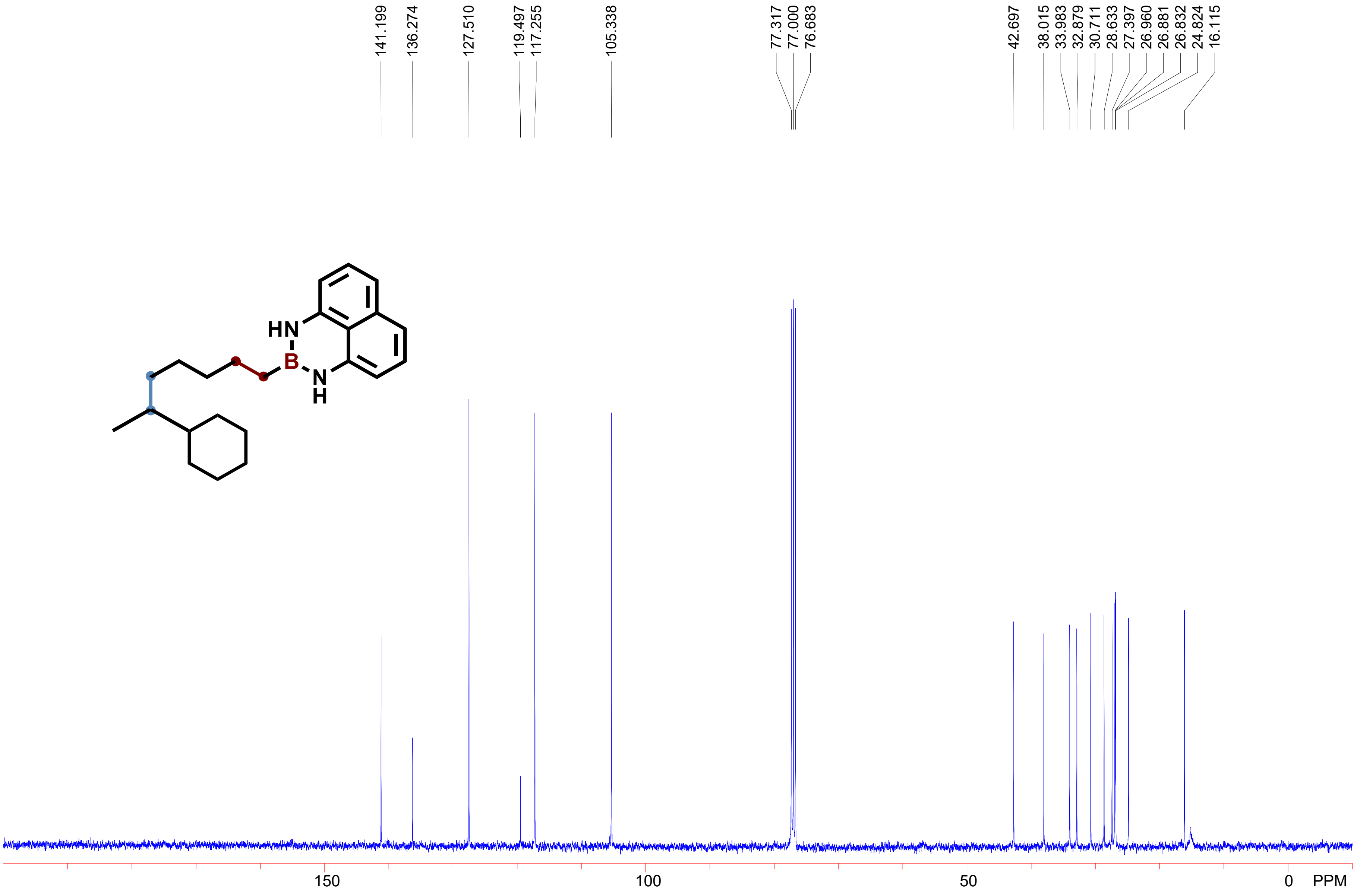

## $^{1}$H NMR-spectrum (400 MHz, $CDCl_3$) of 36

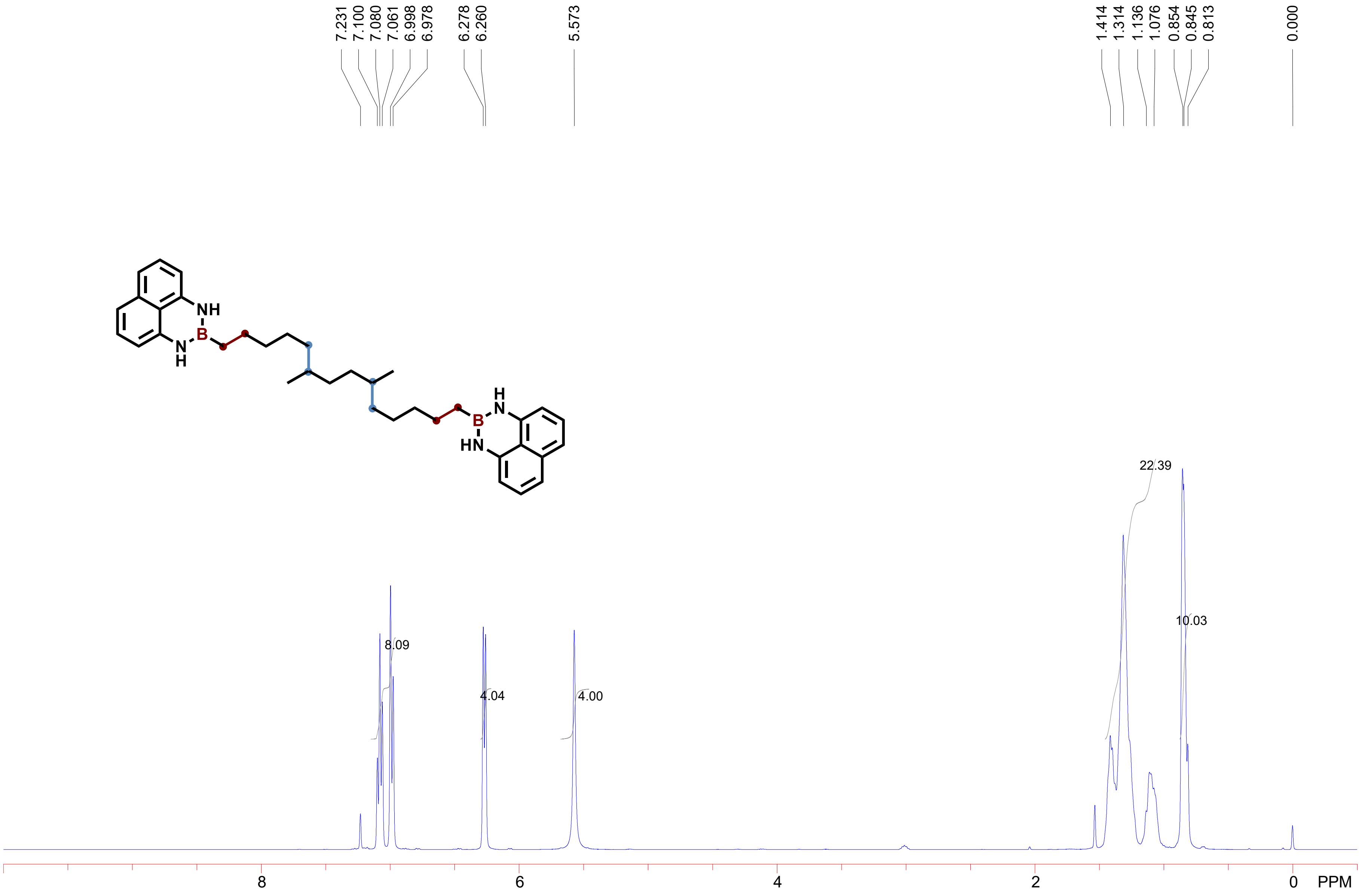

**$^{13}$C NMR-spectrum (100 MHz, CDCl3) of 36**

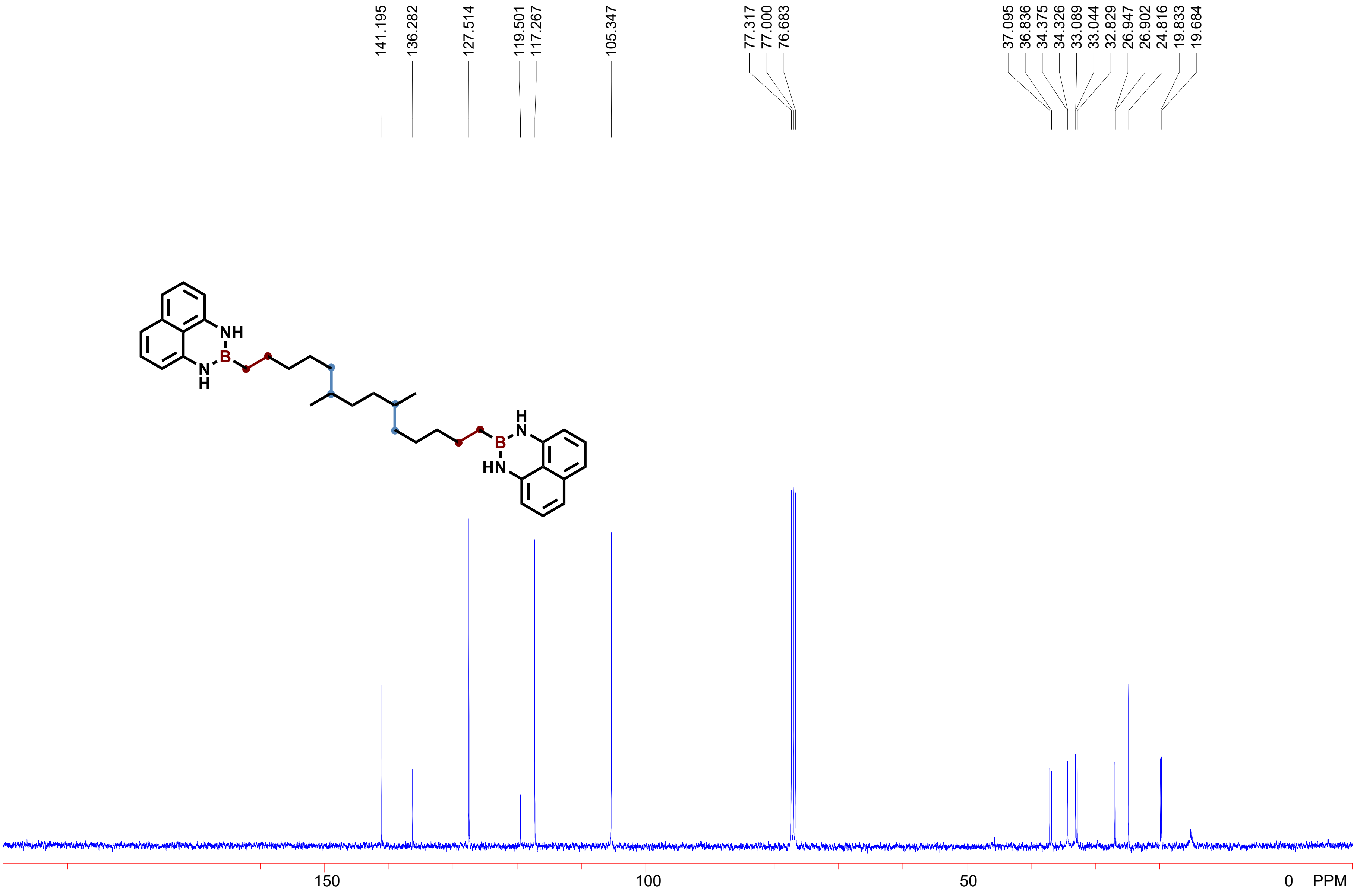

**$^{1}$H NMR-spectrum (400 MHz, CDCl3) of 37**

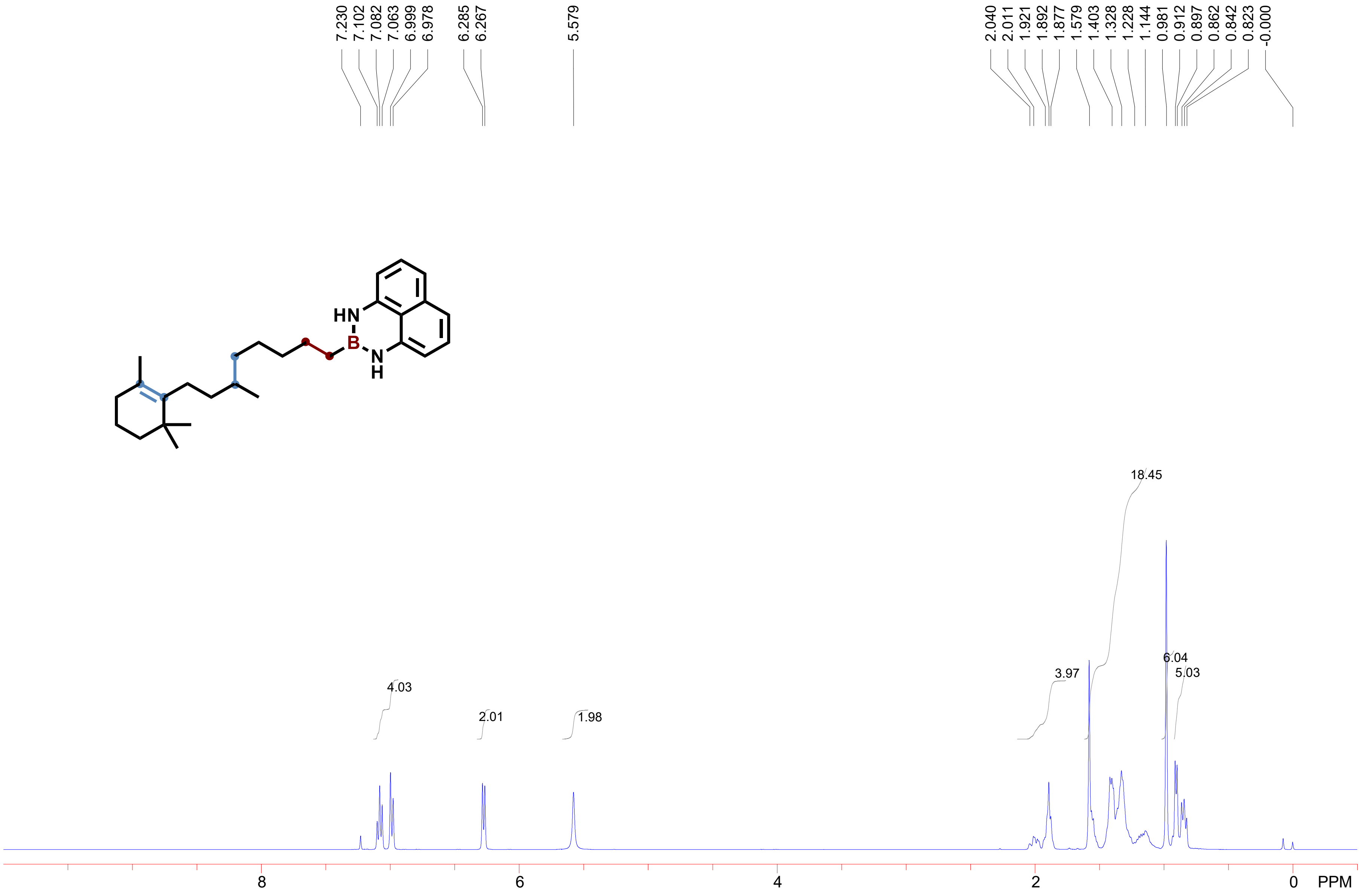

**$^{13}$C NMR-spectrum (100 MHz, CDCl3) of 37**

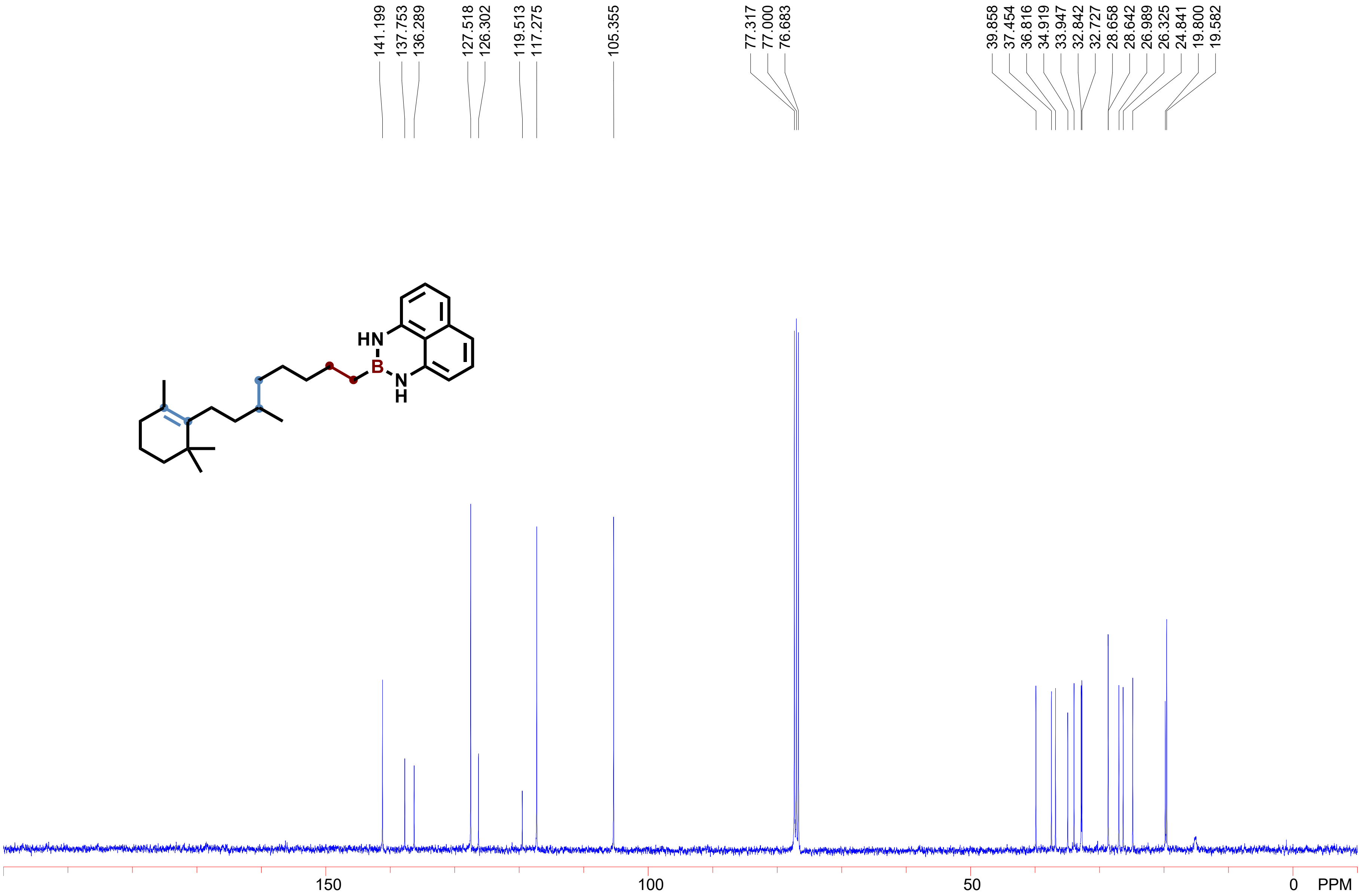

## $^{1}$H NMR-spectrum (400 MHz, $CDCl_3$) of 38

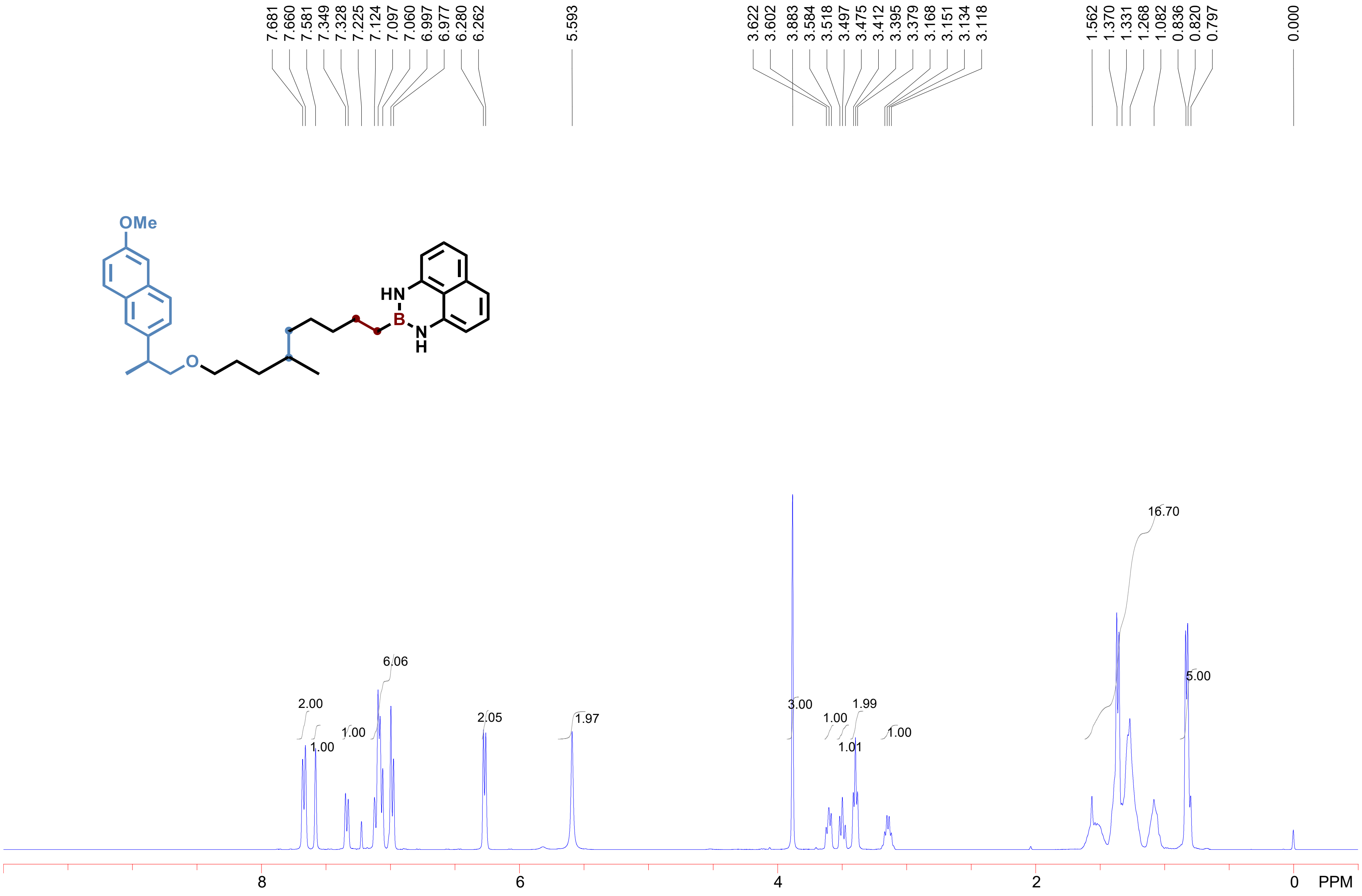

## $^{13}$C NMR-spectrum (100 MHz, $CDCl_3$) of 38

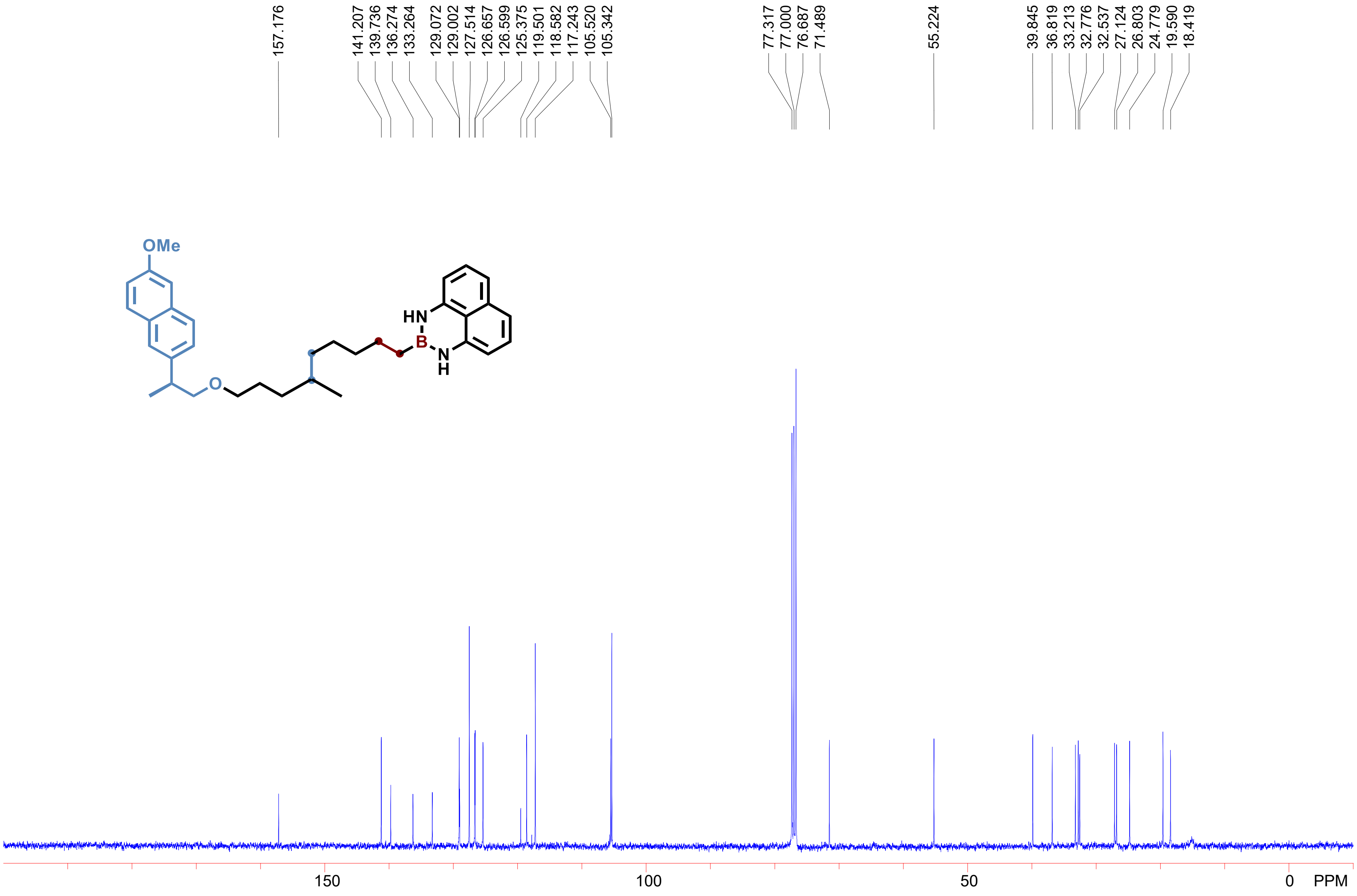

## $^{1}$H NMR-spectrum (400 MHz, $CDCl_3$) of 39

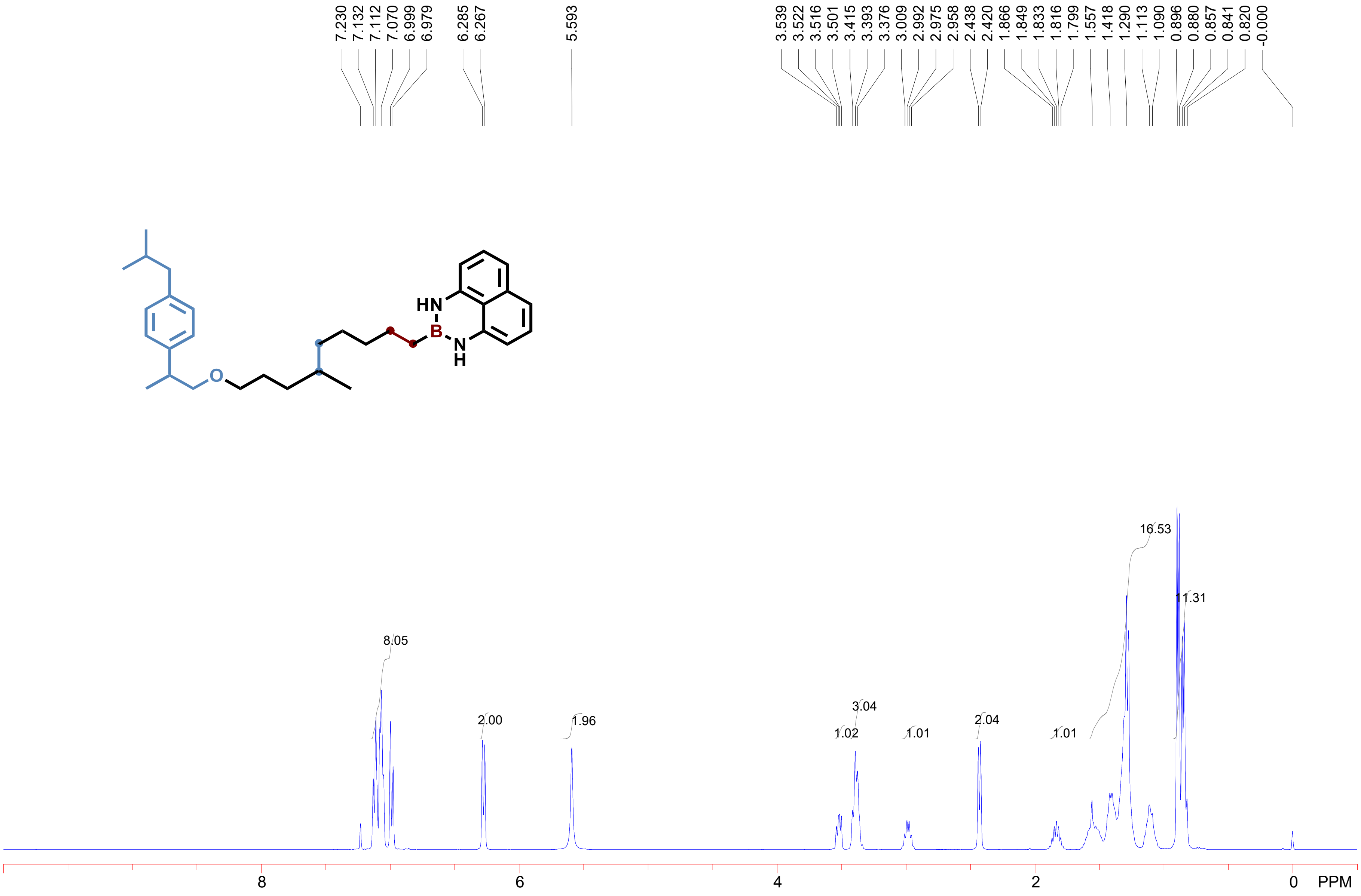

**$^{13}$C NMR-spectrum (100 MHz, $CDCl_3$) of 39**

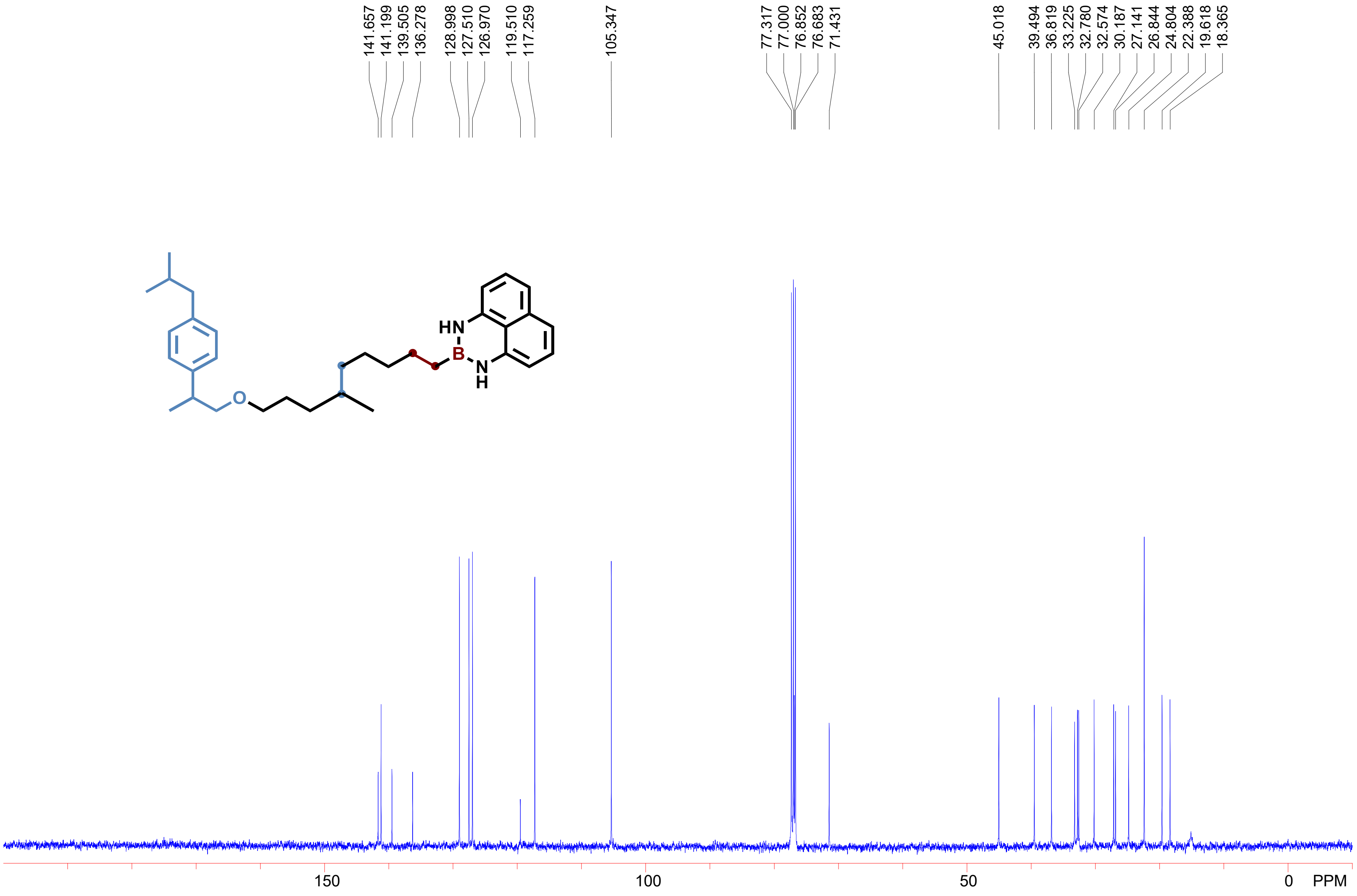

**$^{1}$H NMR-spectrum (400 MHz, $CDCl_3$) of 40**

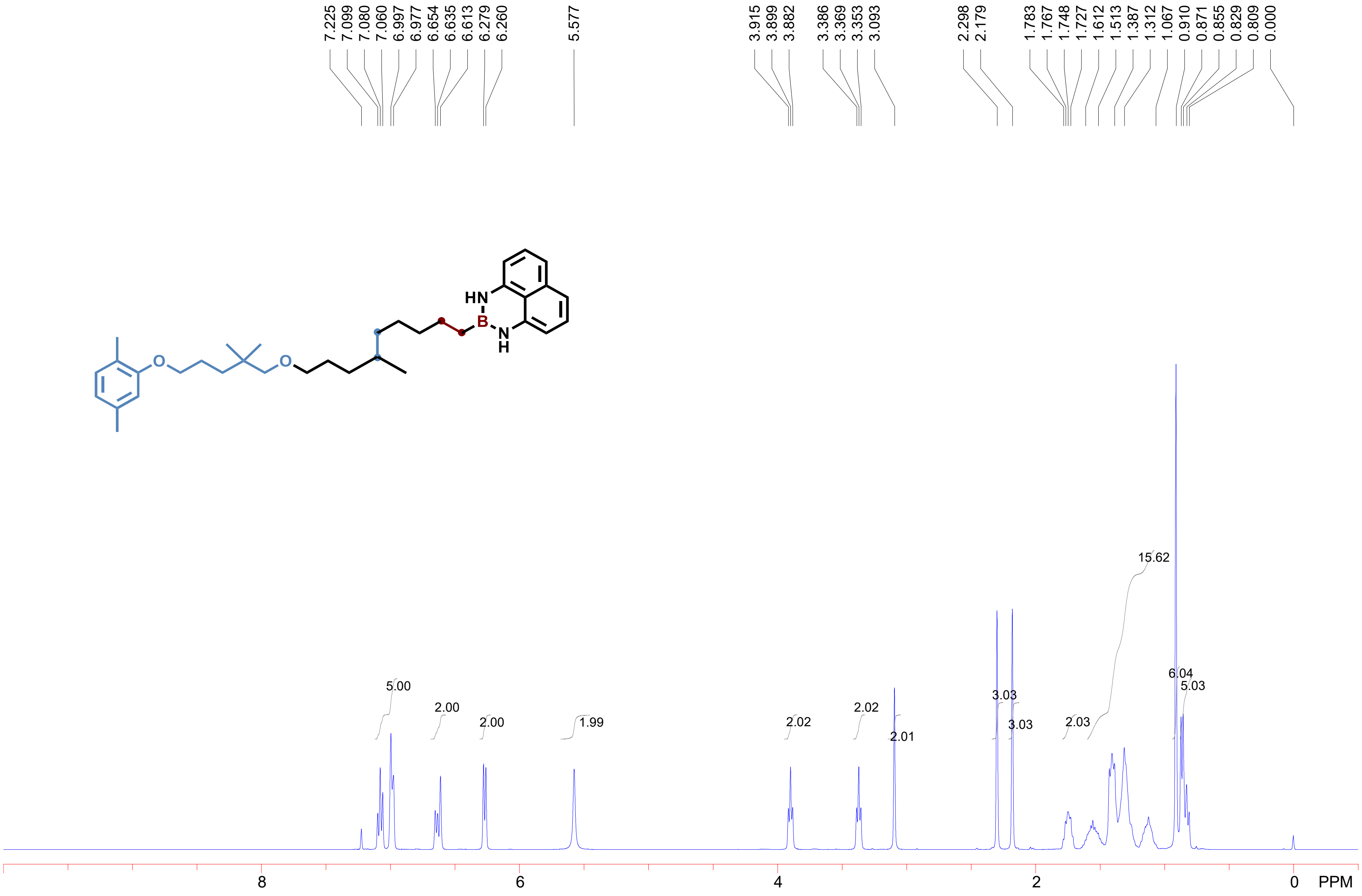

## $^{13}C$ NMR-spectrum (100 MHz, $CDCl_3$) of 40

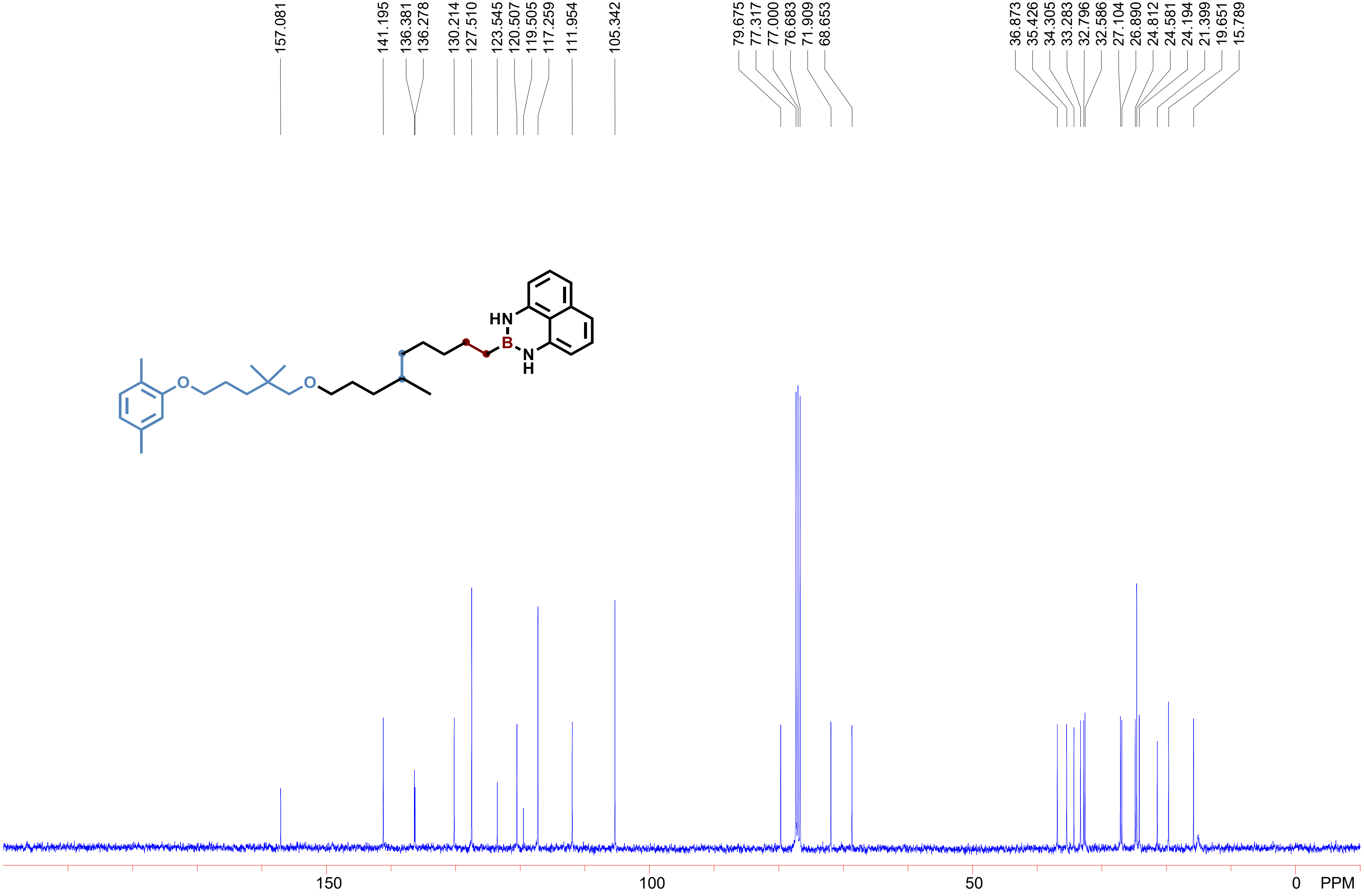

**$^{1}$H NMR-spectrum (400 MHz, CDCl3) of 41**

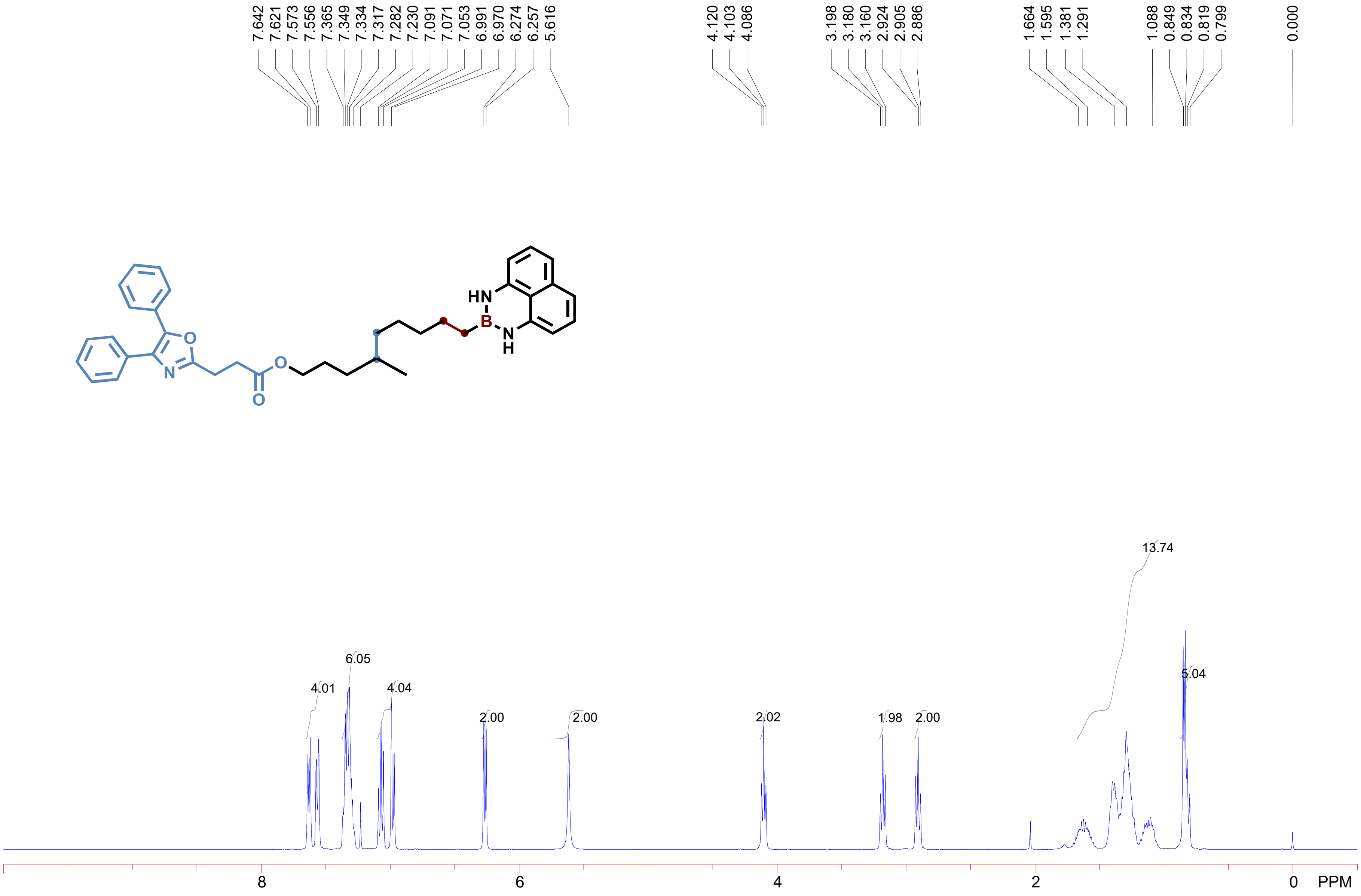

**$^{13}$C NMR-spectrum (100 MHz, CDCl3) of 41**

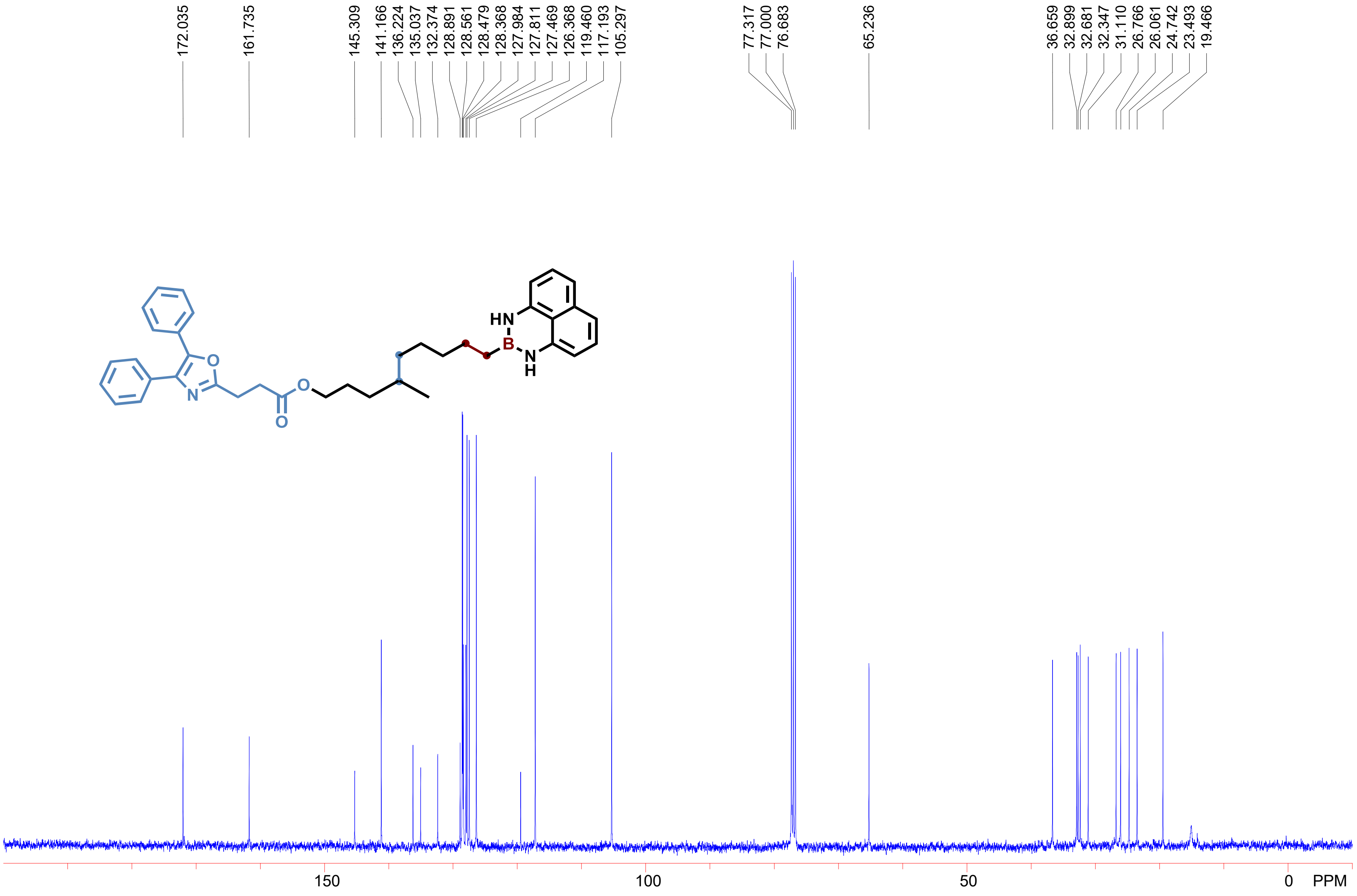

**$^{1}$H NMR-spectrum (400 MHz, CDCl3) of 42**

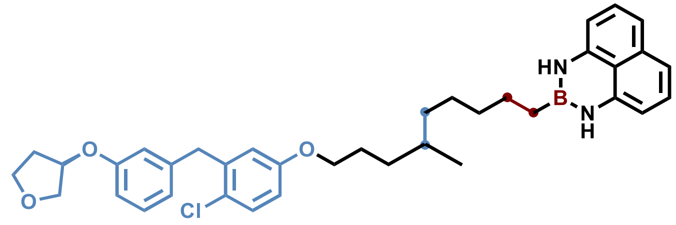


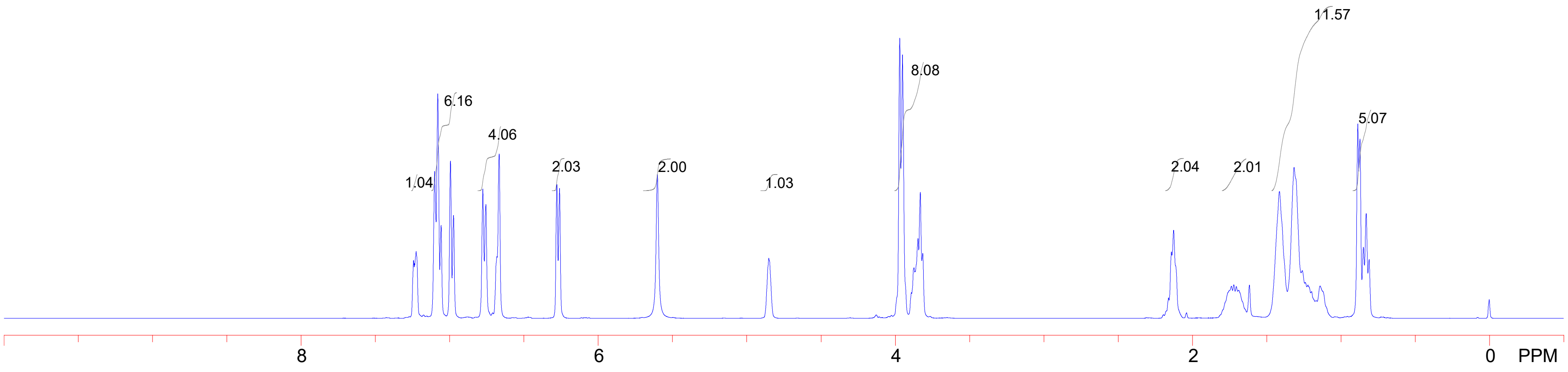

**$^{13}$C NMR-spectrum (100 MHz, $CDCl_3$) of 42**

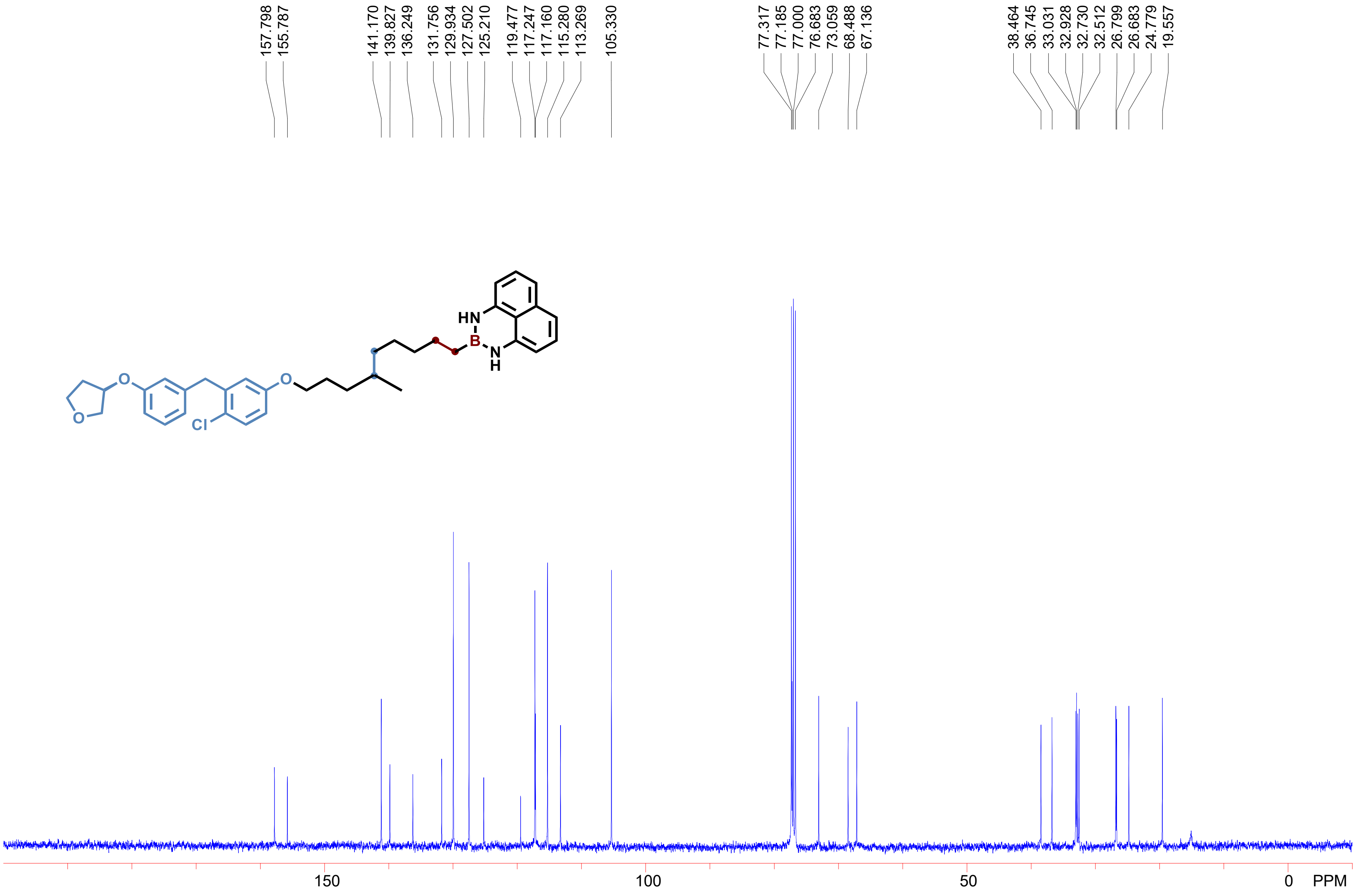

**$^{1}$H NMR-spectrum (400 MHz, CDCl3) of 43**

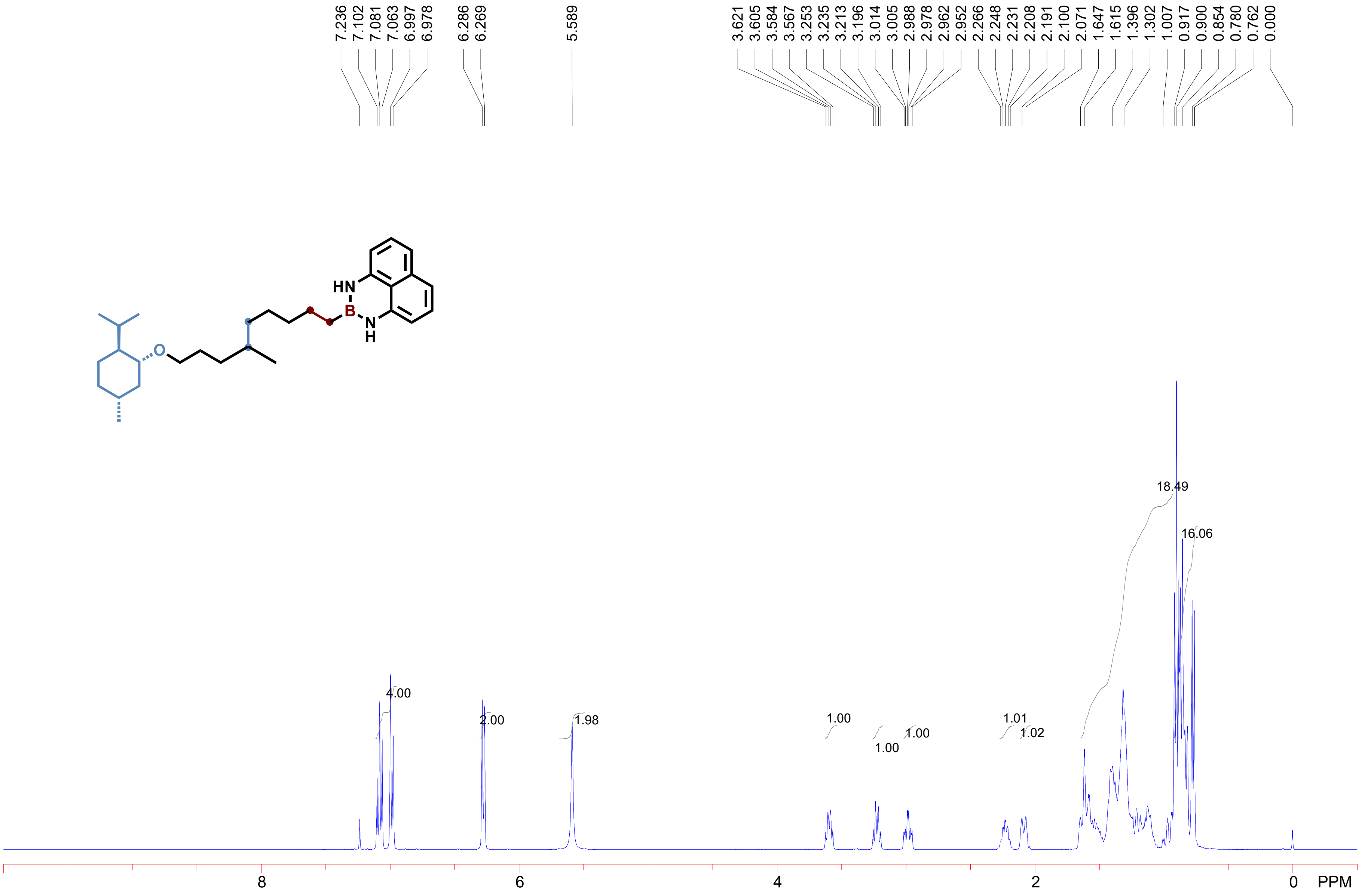

**$^{13}$C NMR-spectrum (100 MHz, $CDCl_3$) of 43**

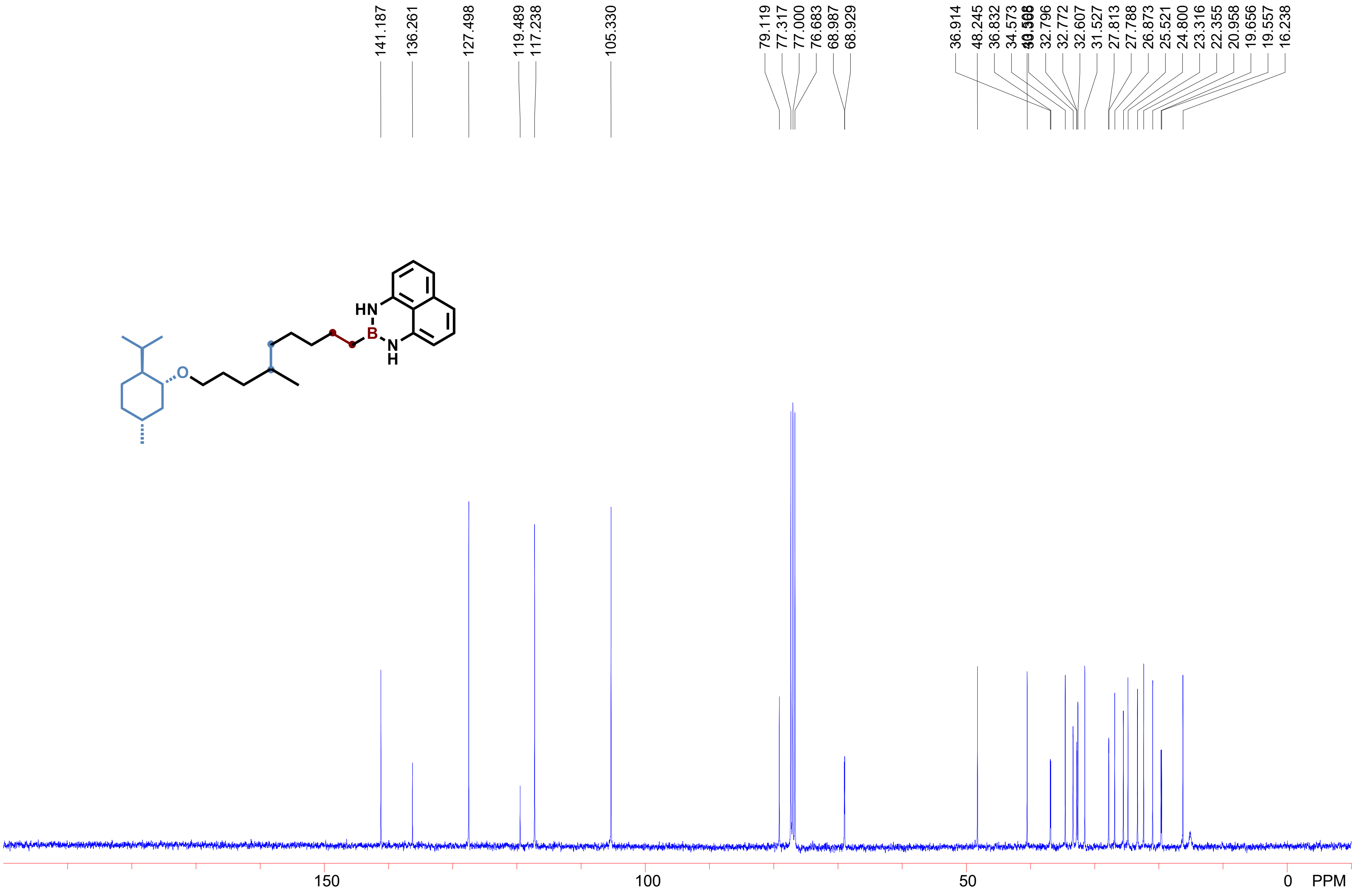

$^{1}$H NMR-spectrum (400 MHz, CDCl$_3$) of 44

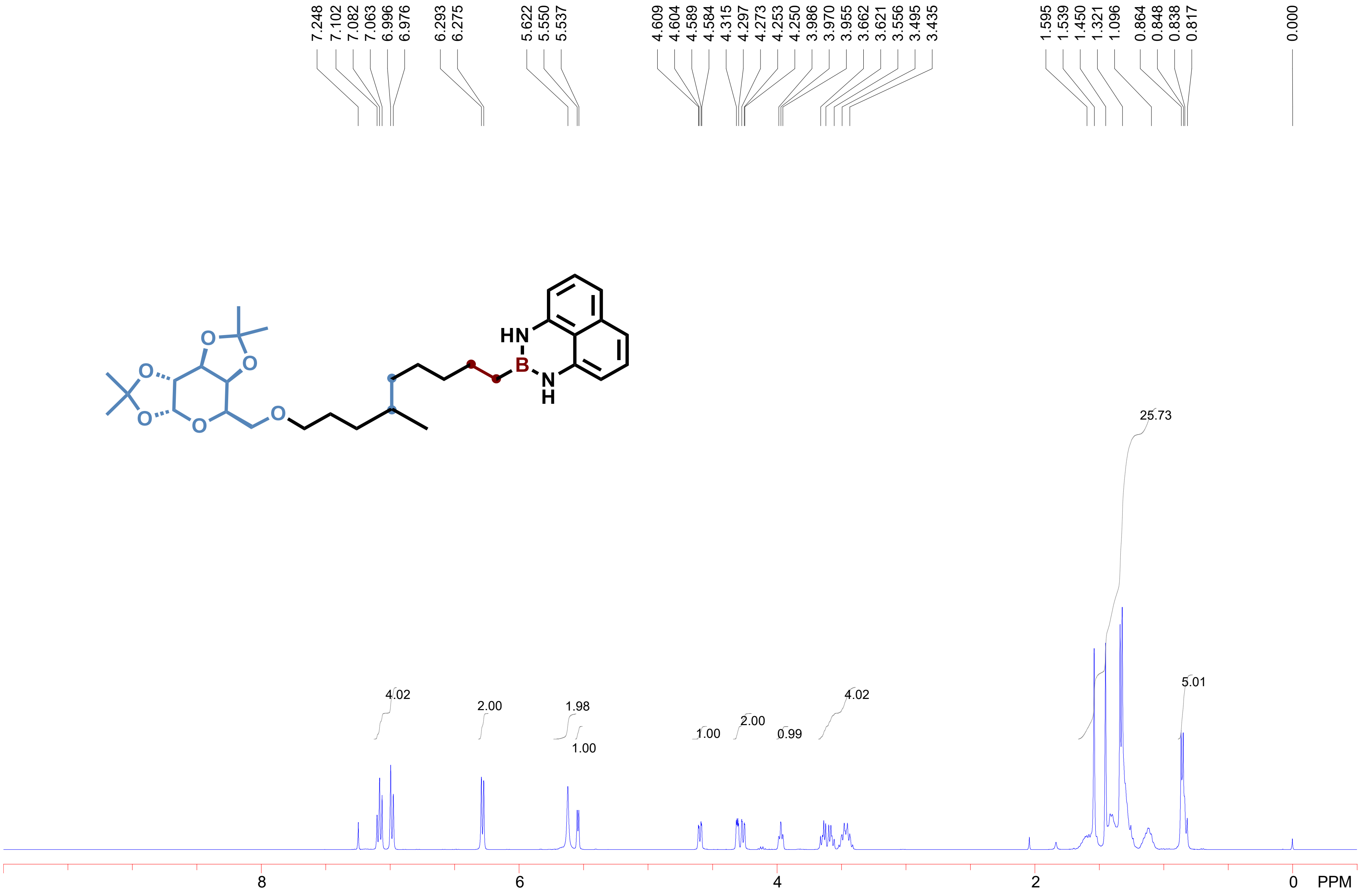

**$^{13}$C NMR-spectrum (100 MHz, CDCl3) of 44**

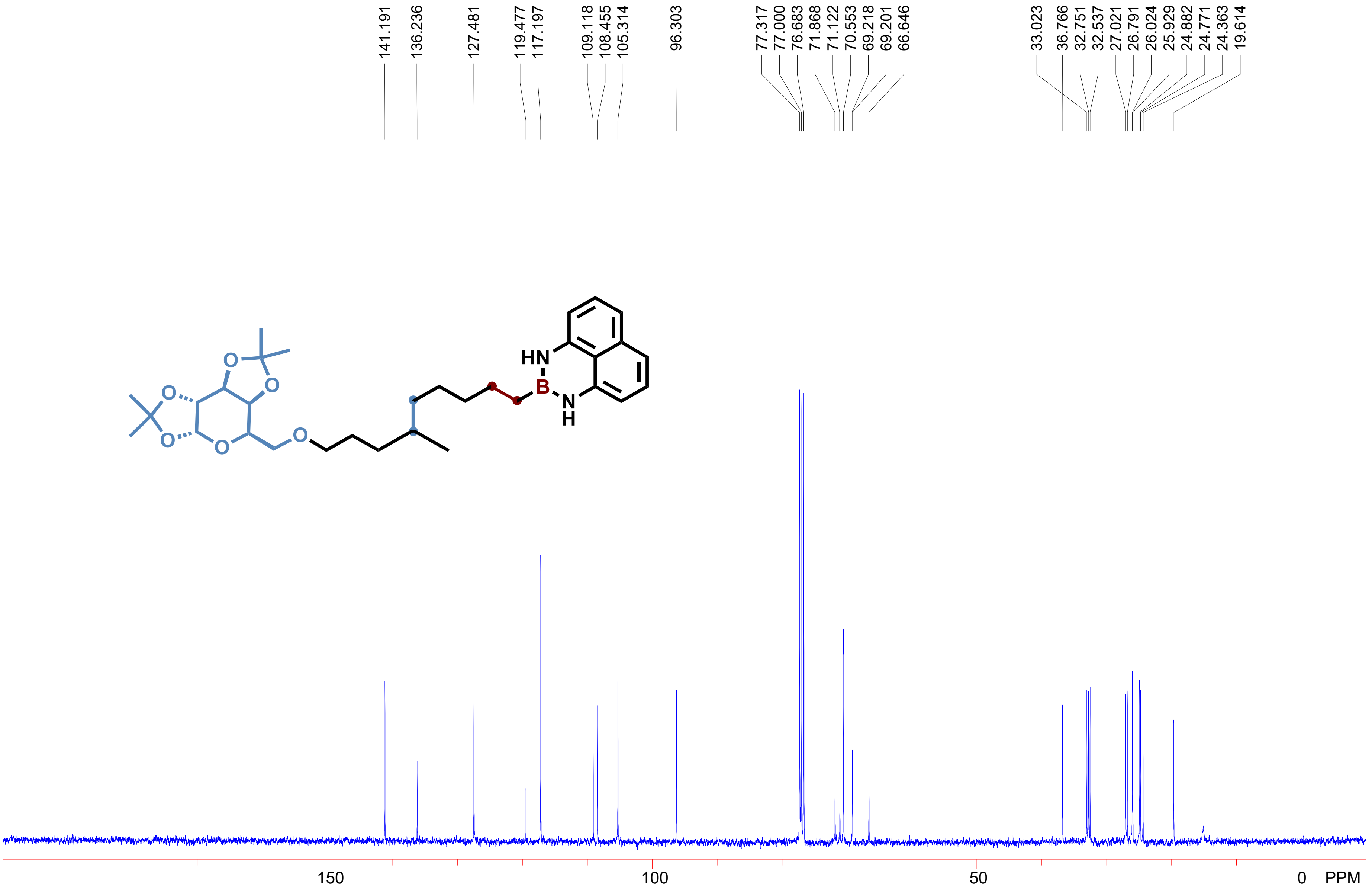

# $^1$H NMR-spectrum (400 MHz, $CDCl_3$) of 45

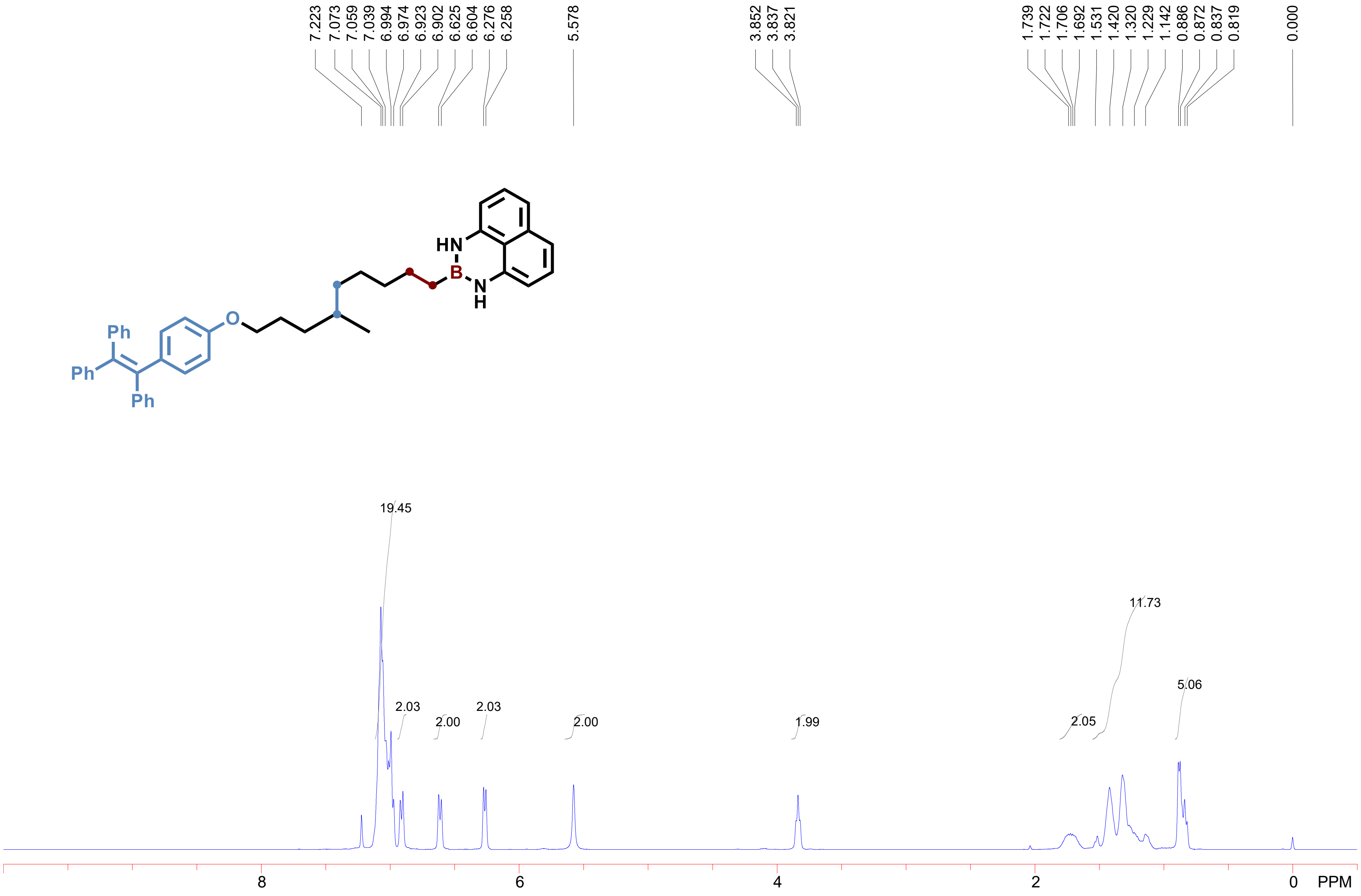

**$^{13}$C NMR-spectrum (100 MHz, CDCl$_3$) of 45**

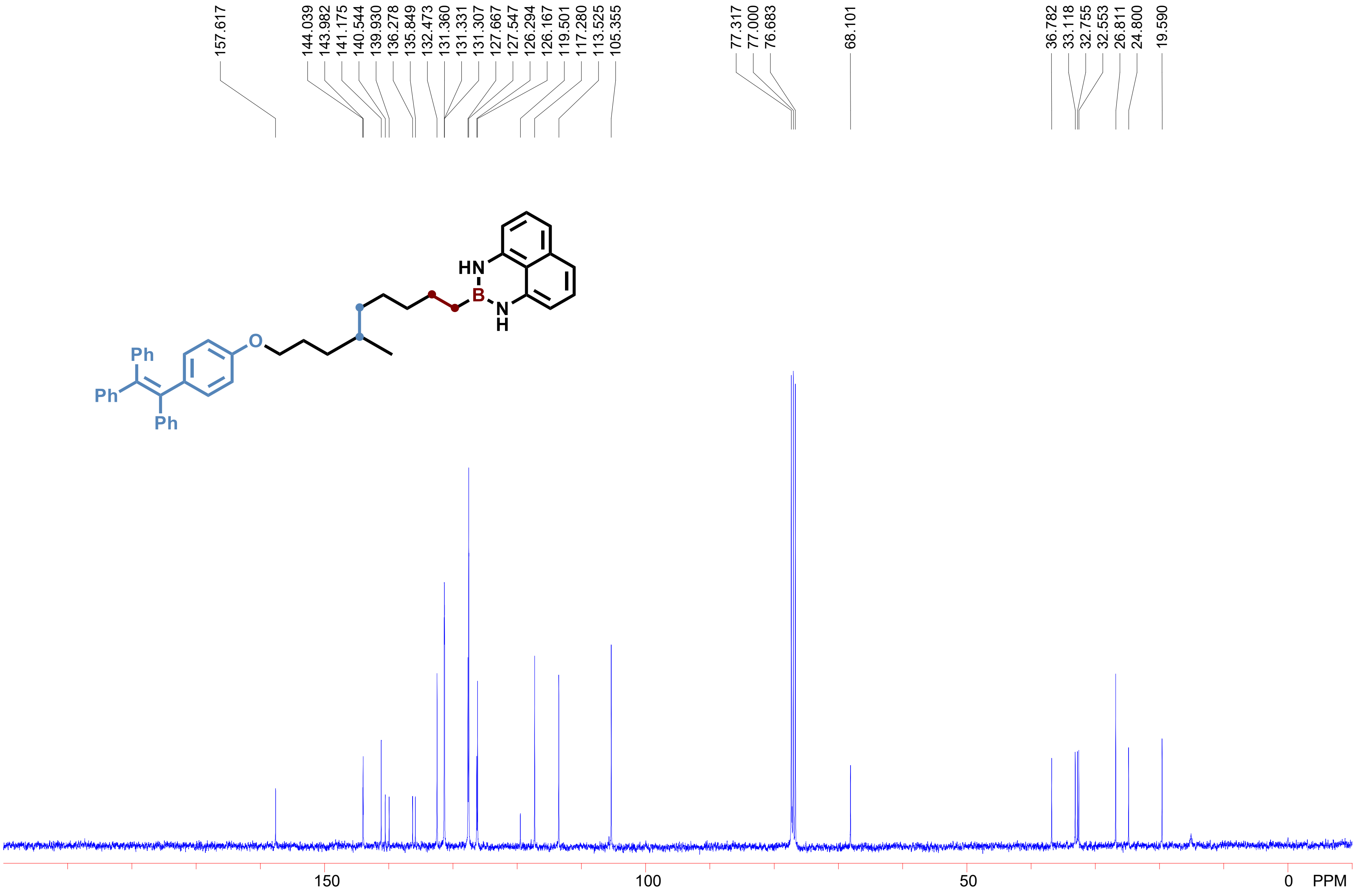

**$^{1}$H NMR-spectrum (400 MHz, CDCl3) of 46**

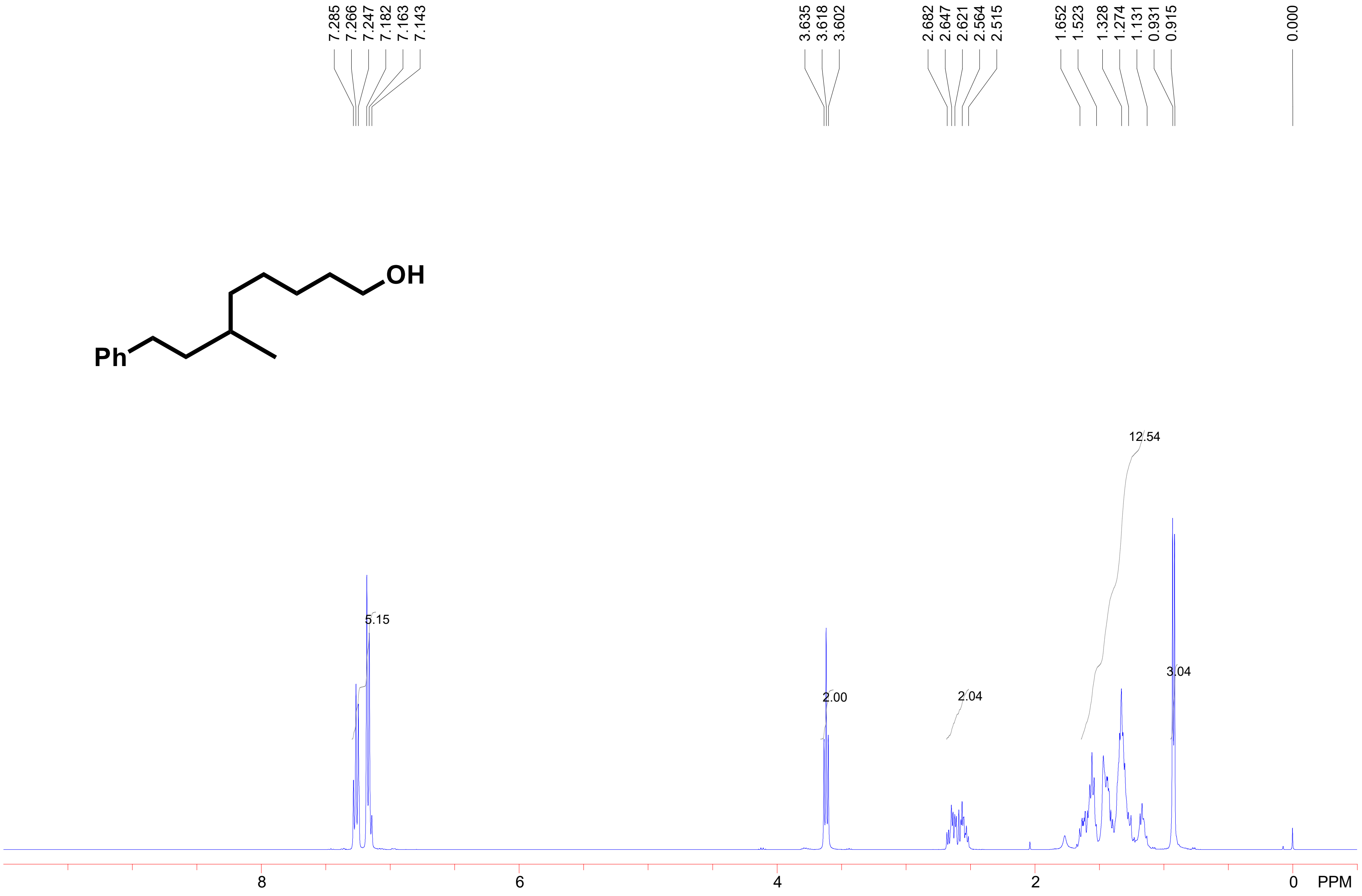

$^{13}$C NMR-spectrum (100 MHz, CDCl3) of 46

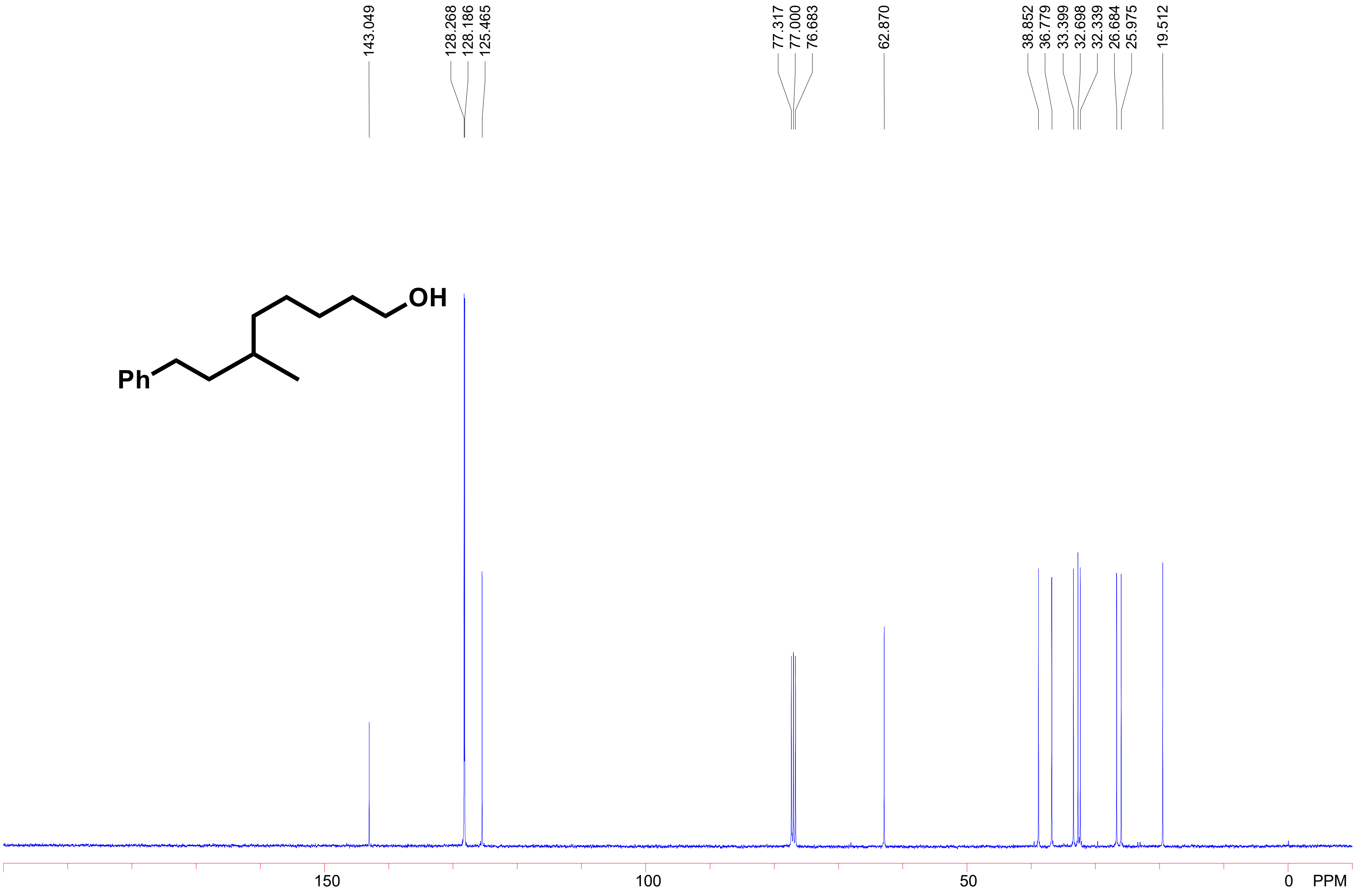

**$^{1}$H NMR-spectrum (400 MHz, CDCl3) of 47**

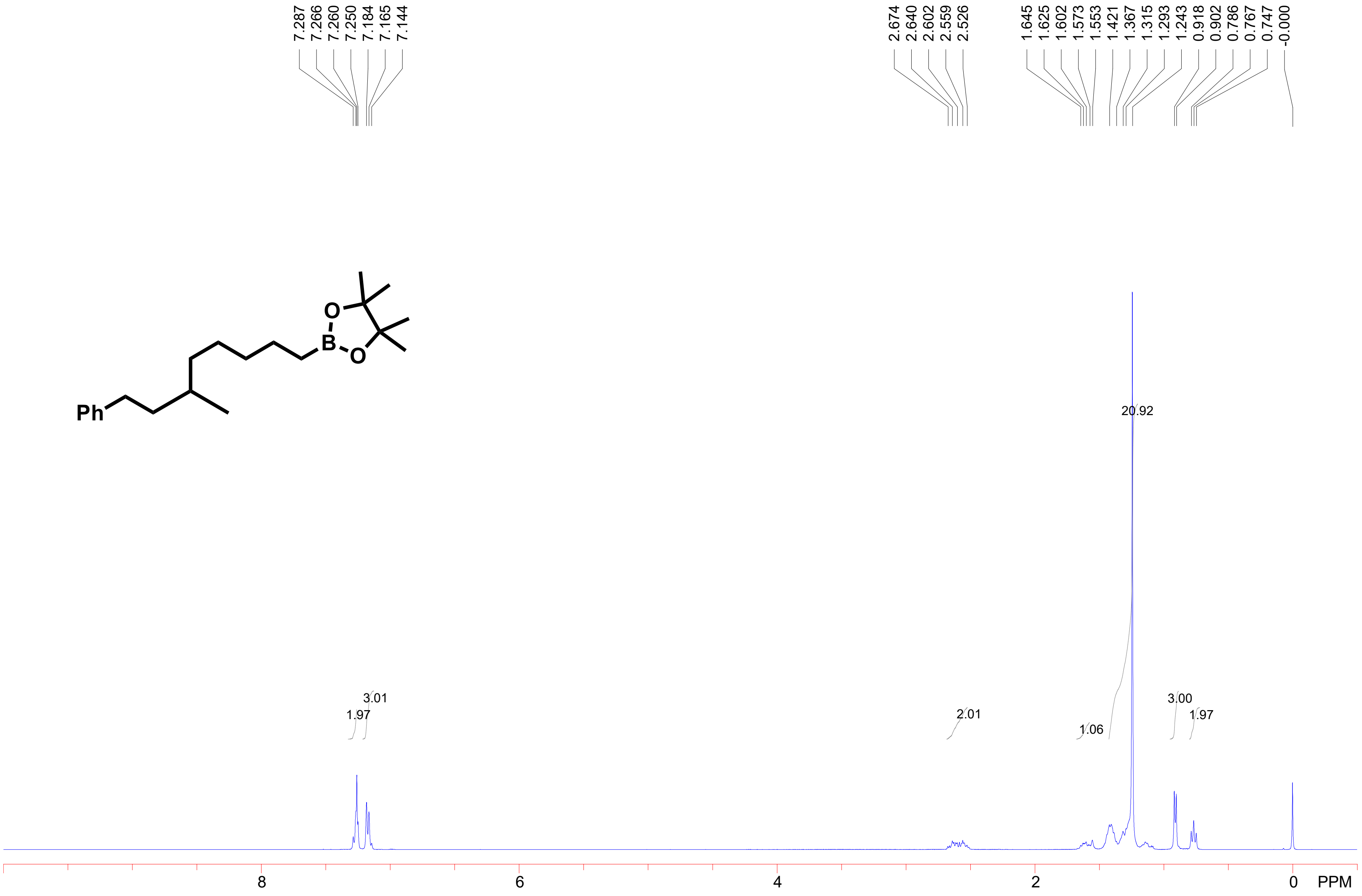

**$^{13}$C NMR-spectrum (100 MHz, CDCl3) of 47**

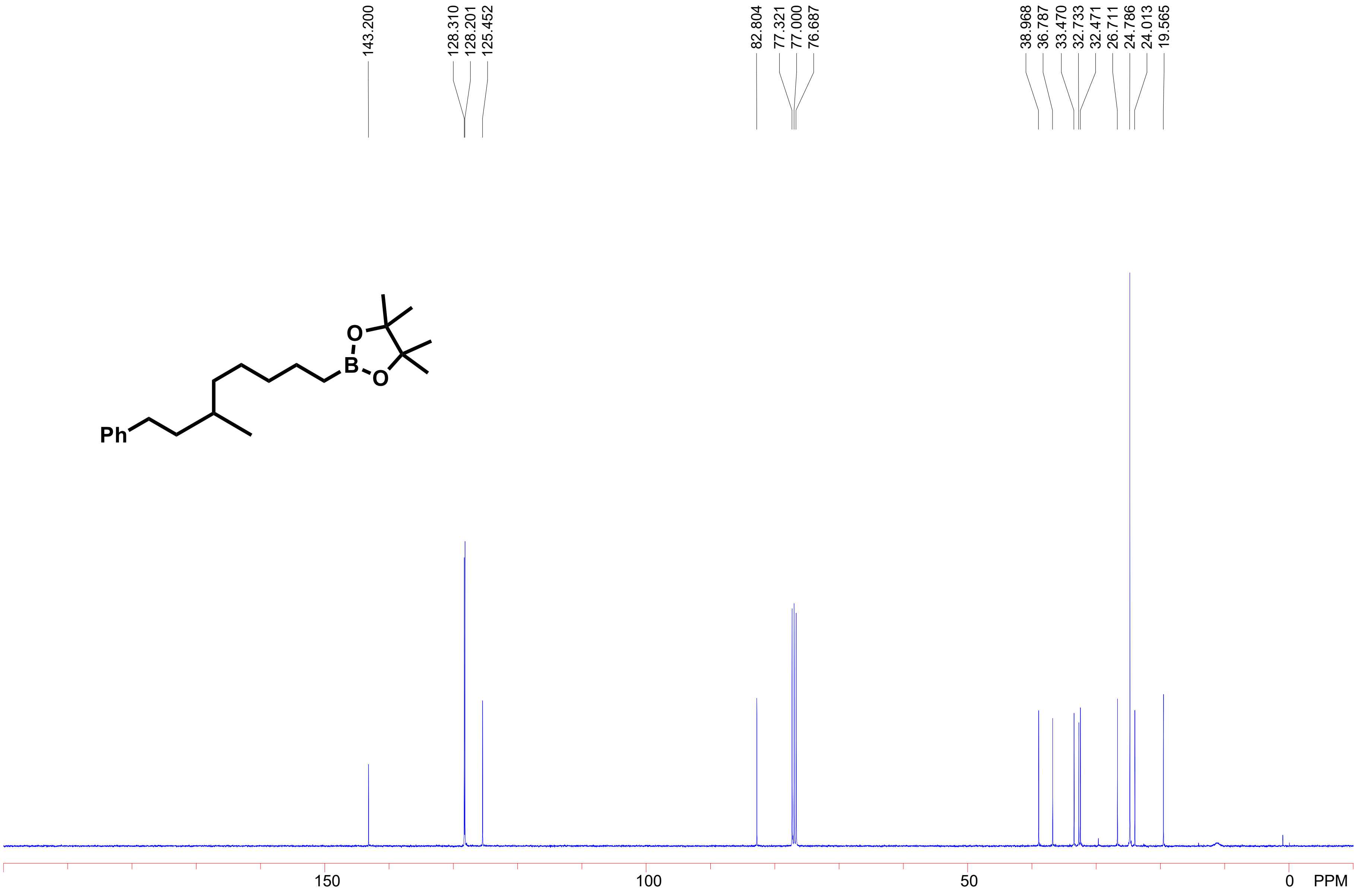

$^{1}$H NMR-spectrum (400 MHz, $CDCl_3$) of 48

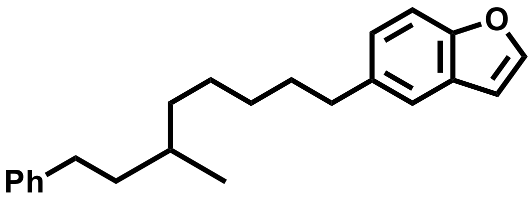

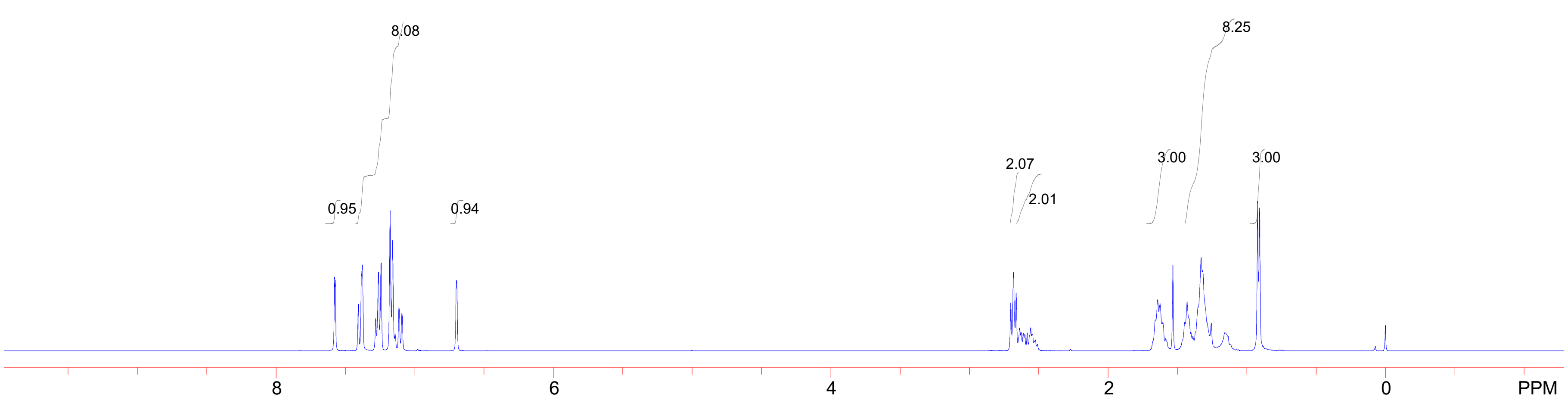

$^{13}$C NMR-spectrum (100 MHz, CDCl$_3$) of 48

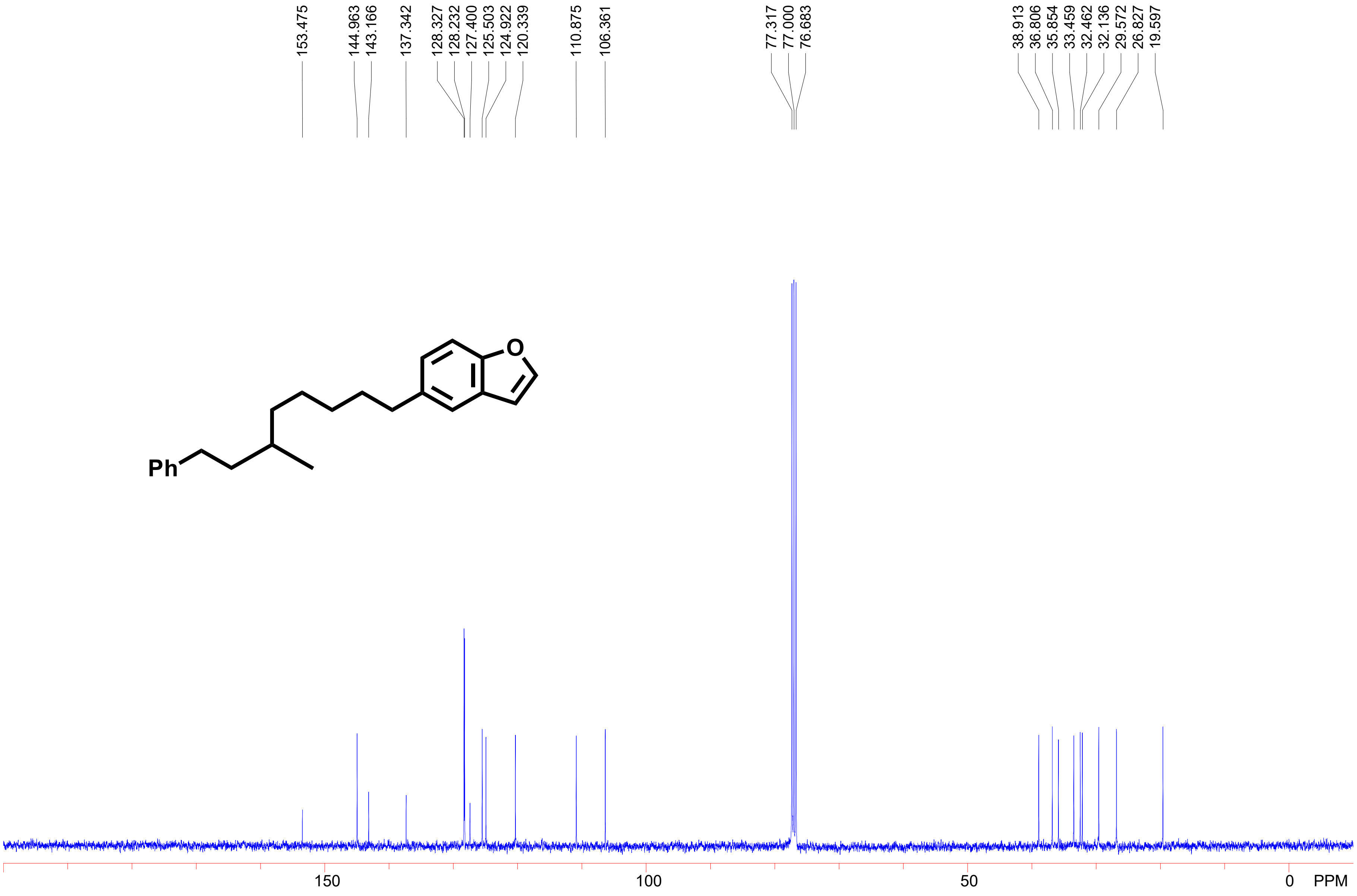

**$^{1}$H NMR-spectrum (400 MHz, CDCl3) of 49**

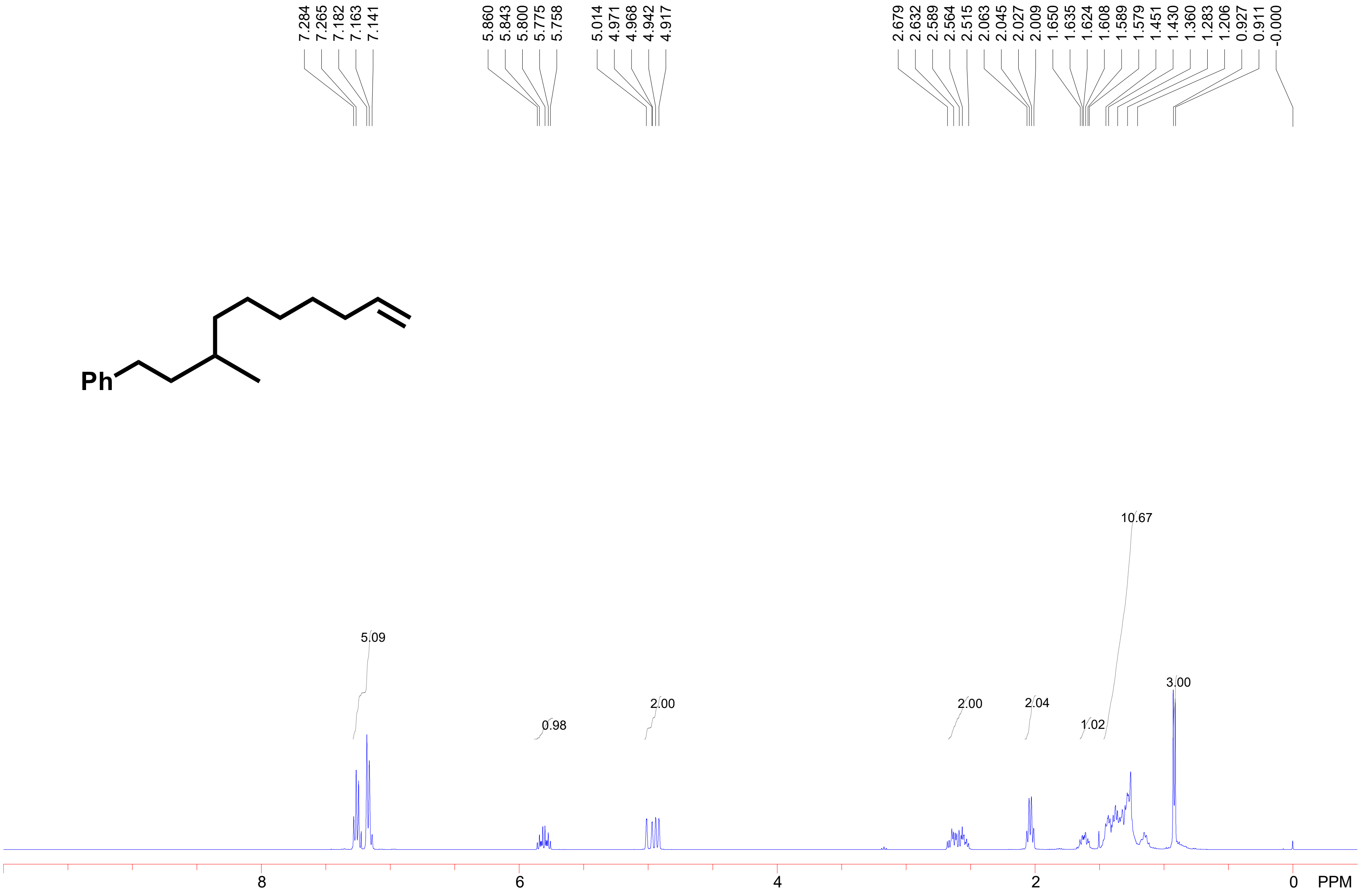

$^{13}C$ NMR-spectrum (100 MHz, $CDCl_3$) of 49

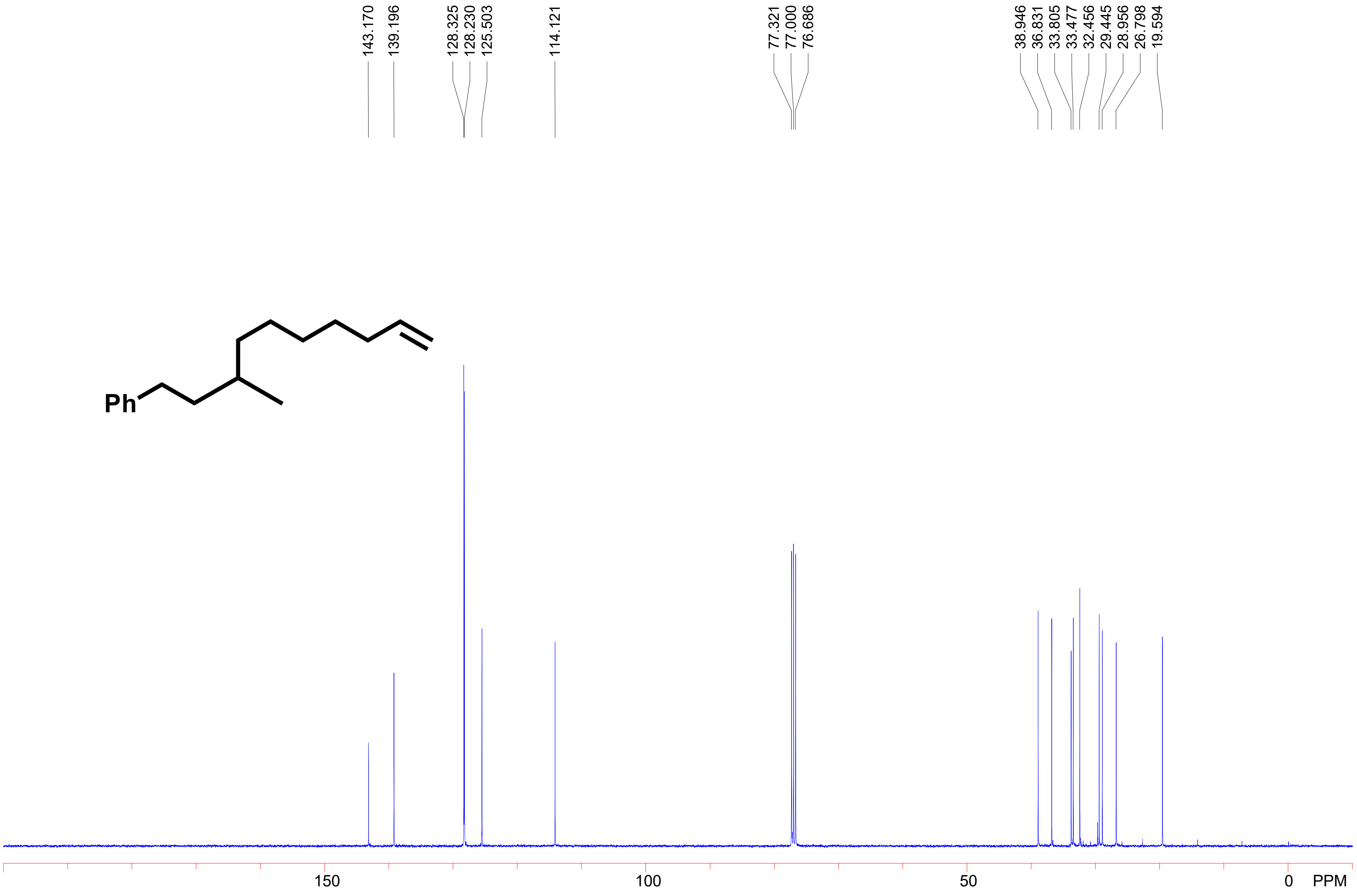

$^{1}$H NMR-spectrum (400 MHz, $CDCl_3$) of 50

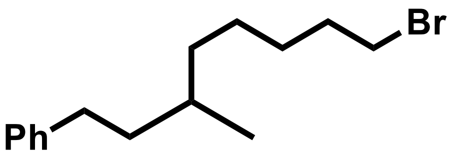

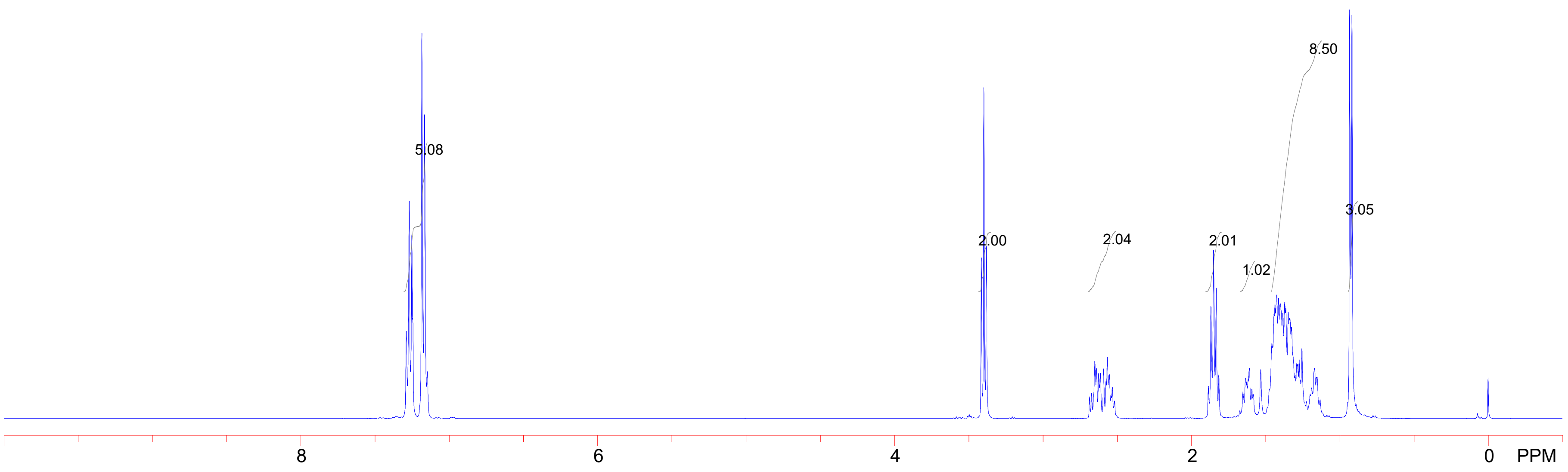

$^{13}$C NMR-spectrum (100 MHz, $CDCl_3$) of 50

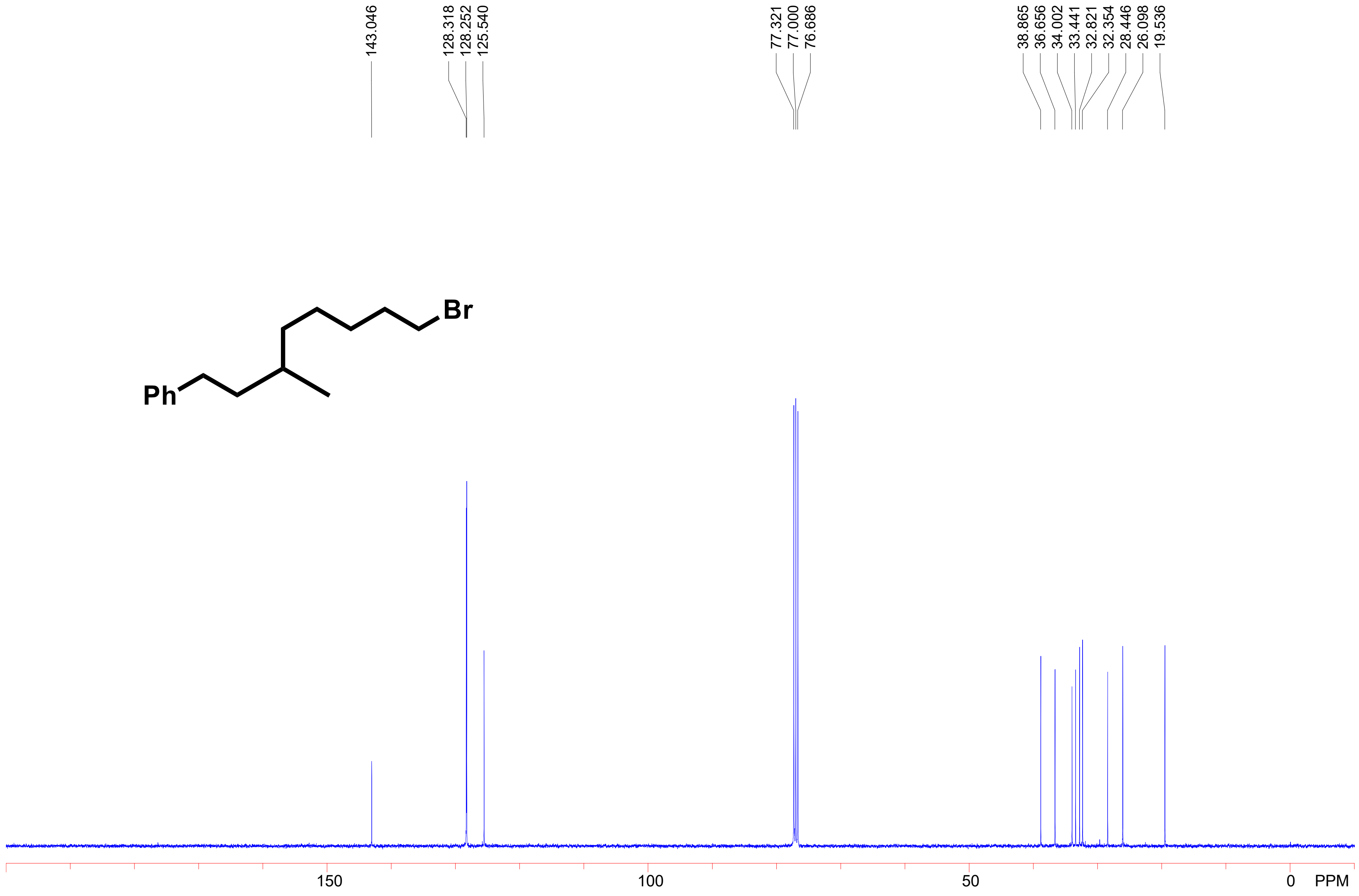

**$^{1}$H NMR-spectrum (400 MHz, $CDCl_3$) of 51**

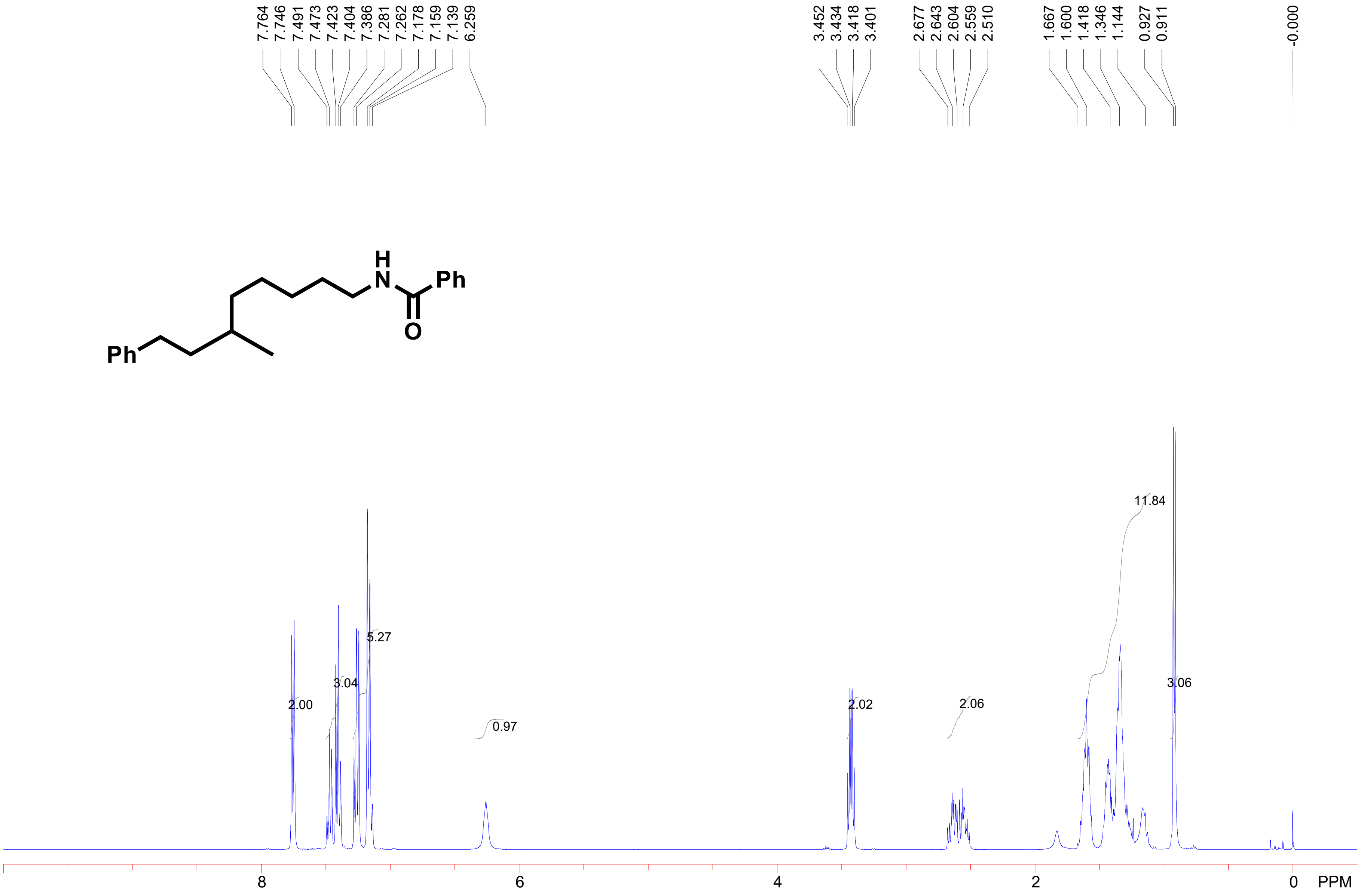

$^{13}$C NMR-spectrum (100 MHz, CDCl3) of 51

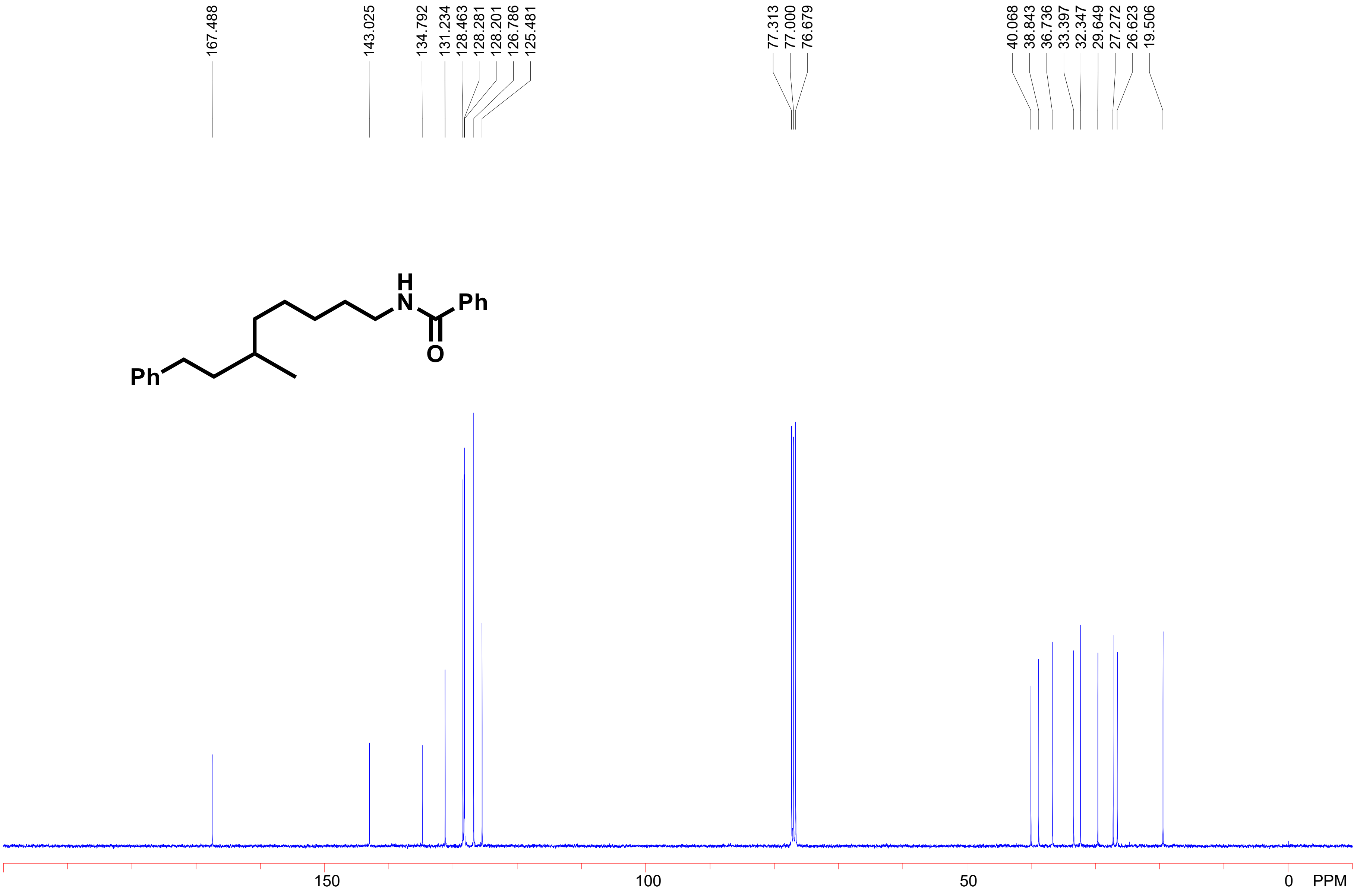

$^{1}H$ NMR-spectrum (400 MHz, $CDCl_3$) of 52

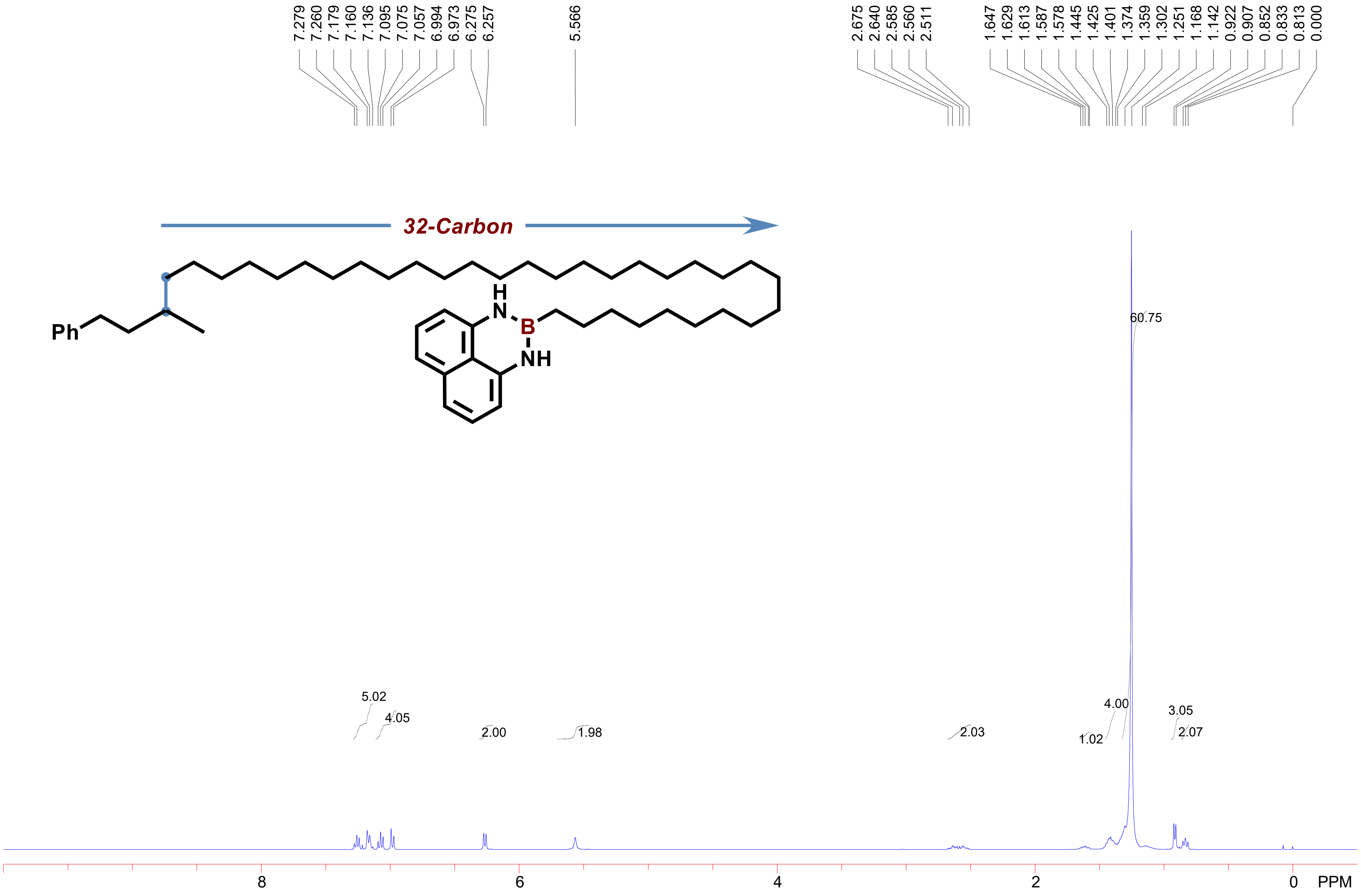

**$^{13}C$ NMR-spectrum (100 MHz, $CDCl_3$) of 52**

143.192, 141.202, 136.302, 128.318, 128.215, 127.508, 125.481, 119.531, 117.293, 105.357, 77.321, 77.000, 76.686, 38.960, 36.911, 33.484, 32.515, 32.493, 30.006, 29.715, 29.620, 29.547, 26.973, 24.800, 19.616

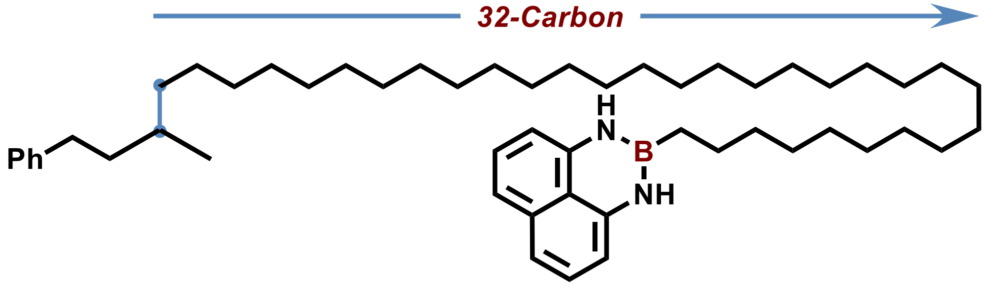


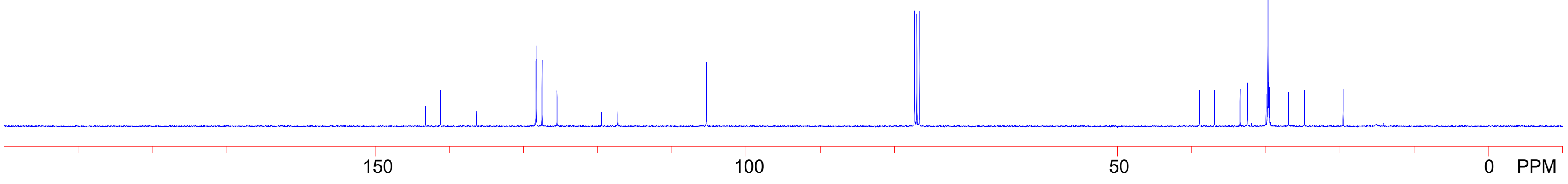

**$^{1}$H NMR-spectrum (400 MHz, $CDCl_3$) of 53**

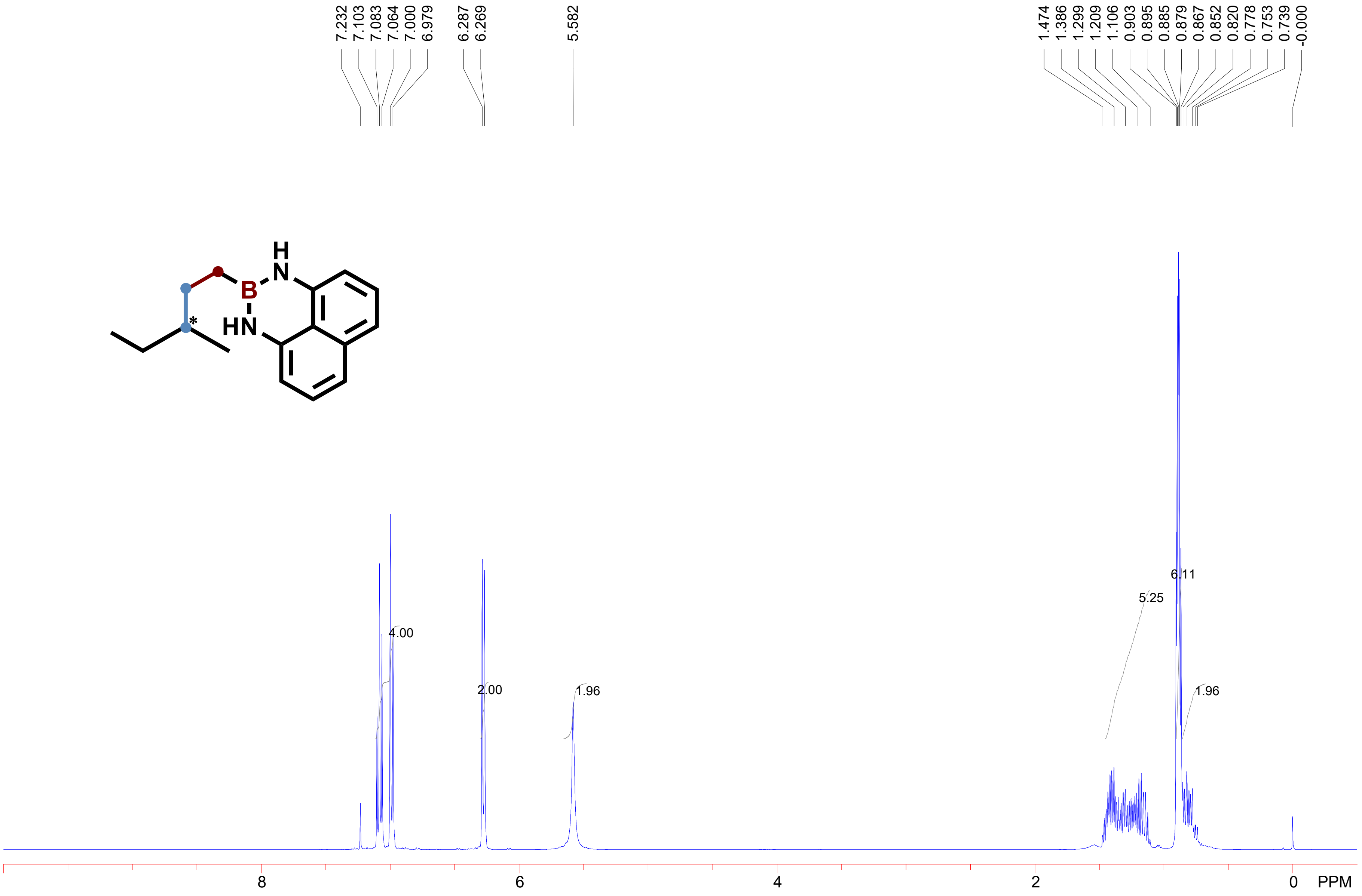

**$^{13}$C NMR-spectrum (100 MHz, $CDCl_3$) of 53**

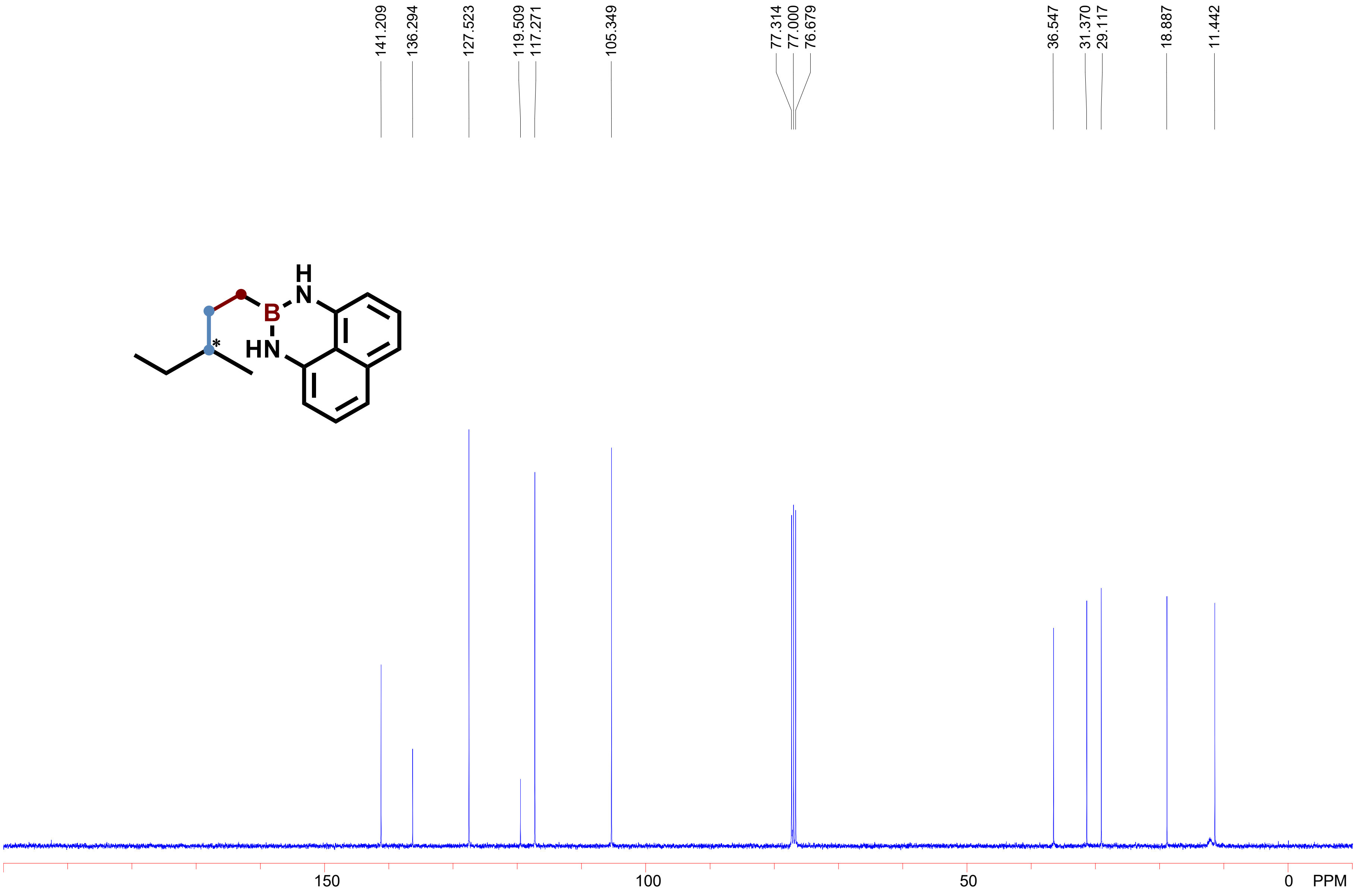

**$^{1}$H NMR-spectrum (400 MHz, CDCl₃) of 54**

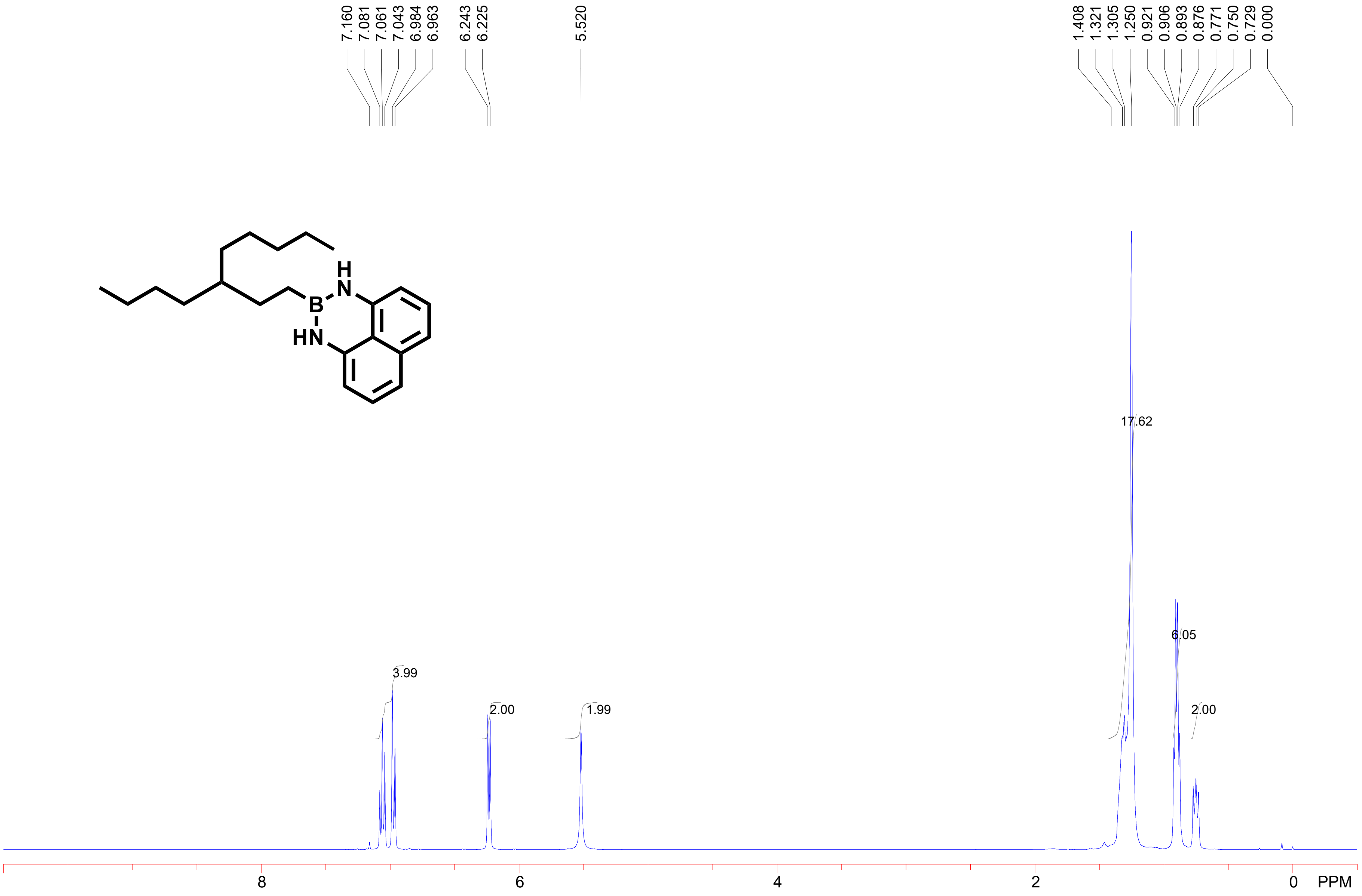

**$^{13}$C NMR-spectrum (100 MHz, $CDCl_3$) of 54**

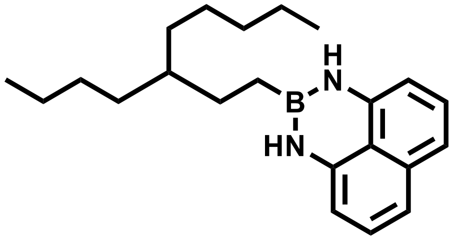


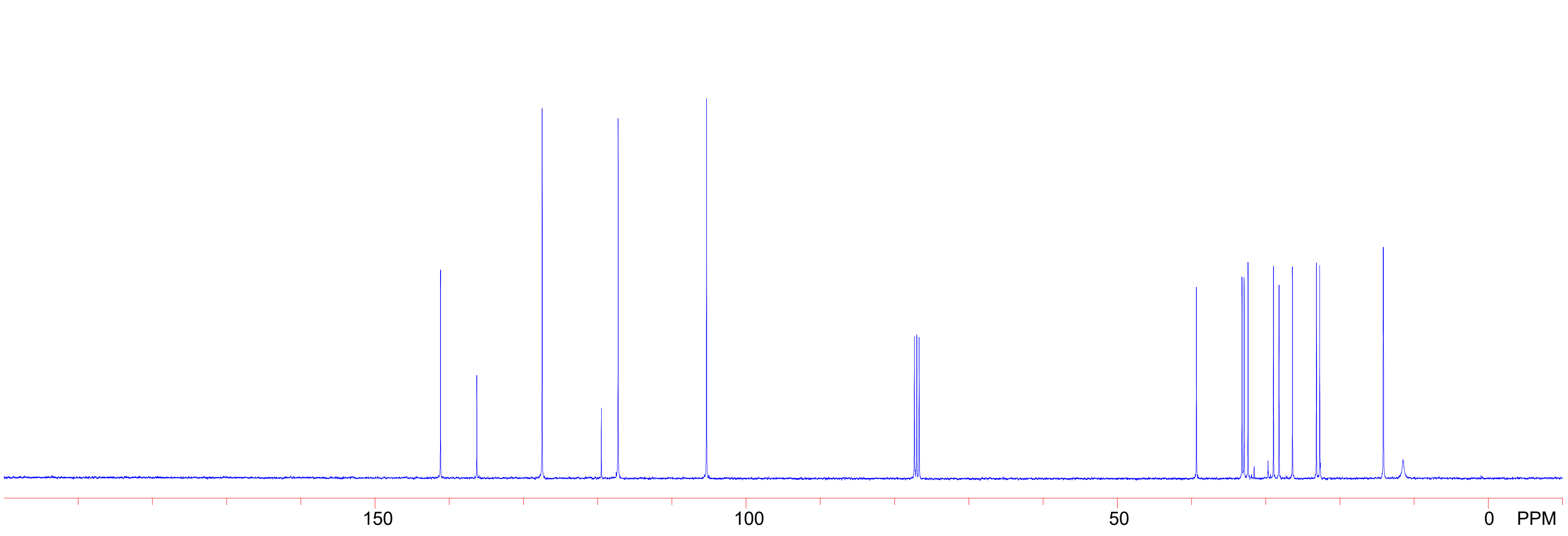

**$^{1}$H NMR-spectrum (400 MHz, CDCl3) of 55**

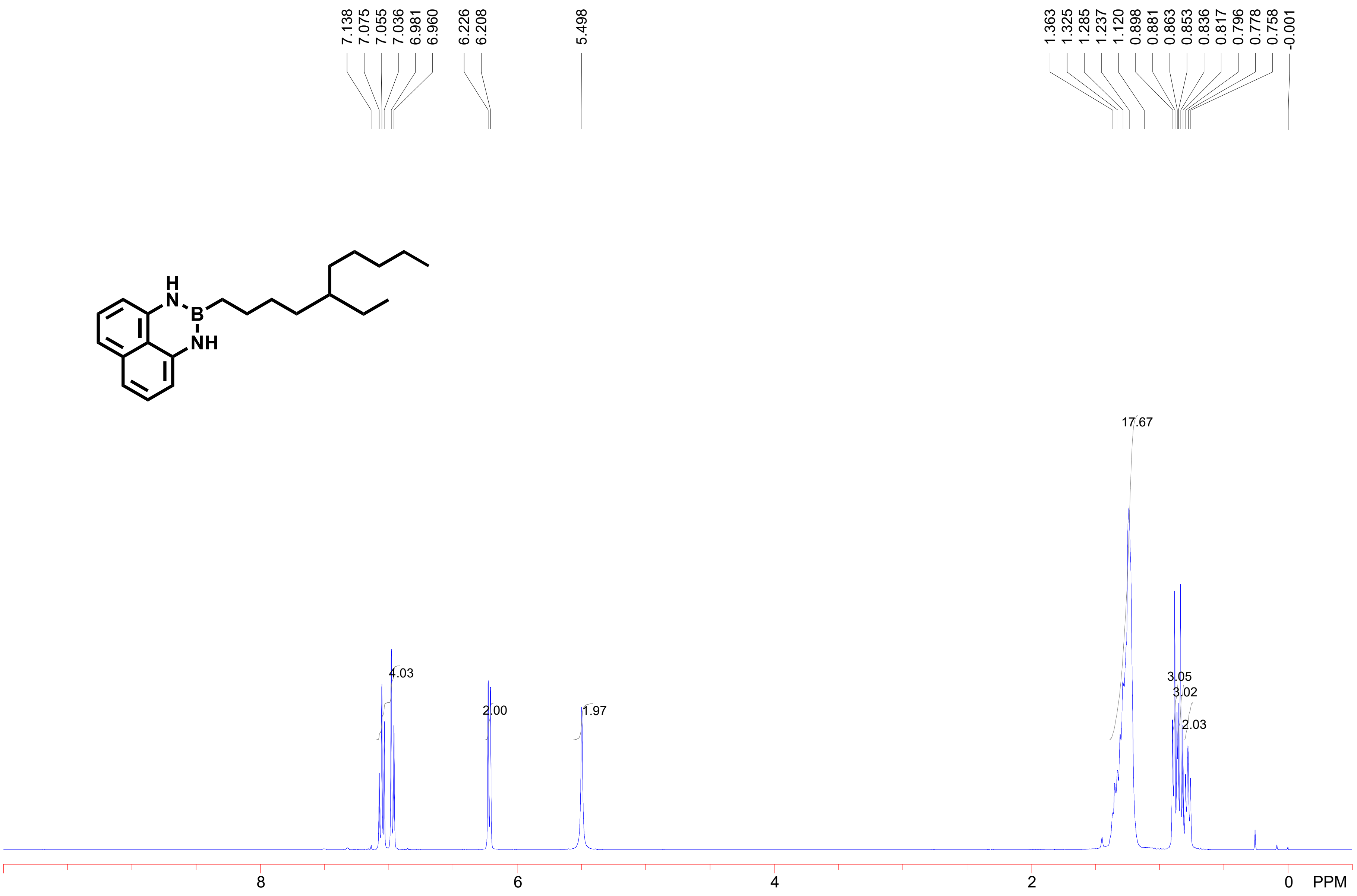

**$^{13}$C NMR-spectrum (100 MHz, $CDCl_3$) of 55**

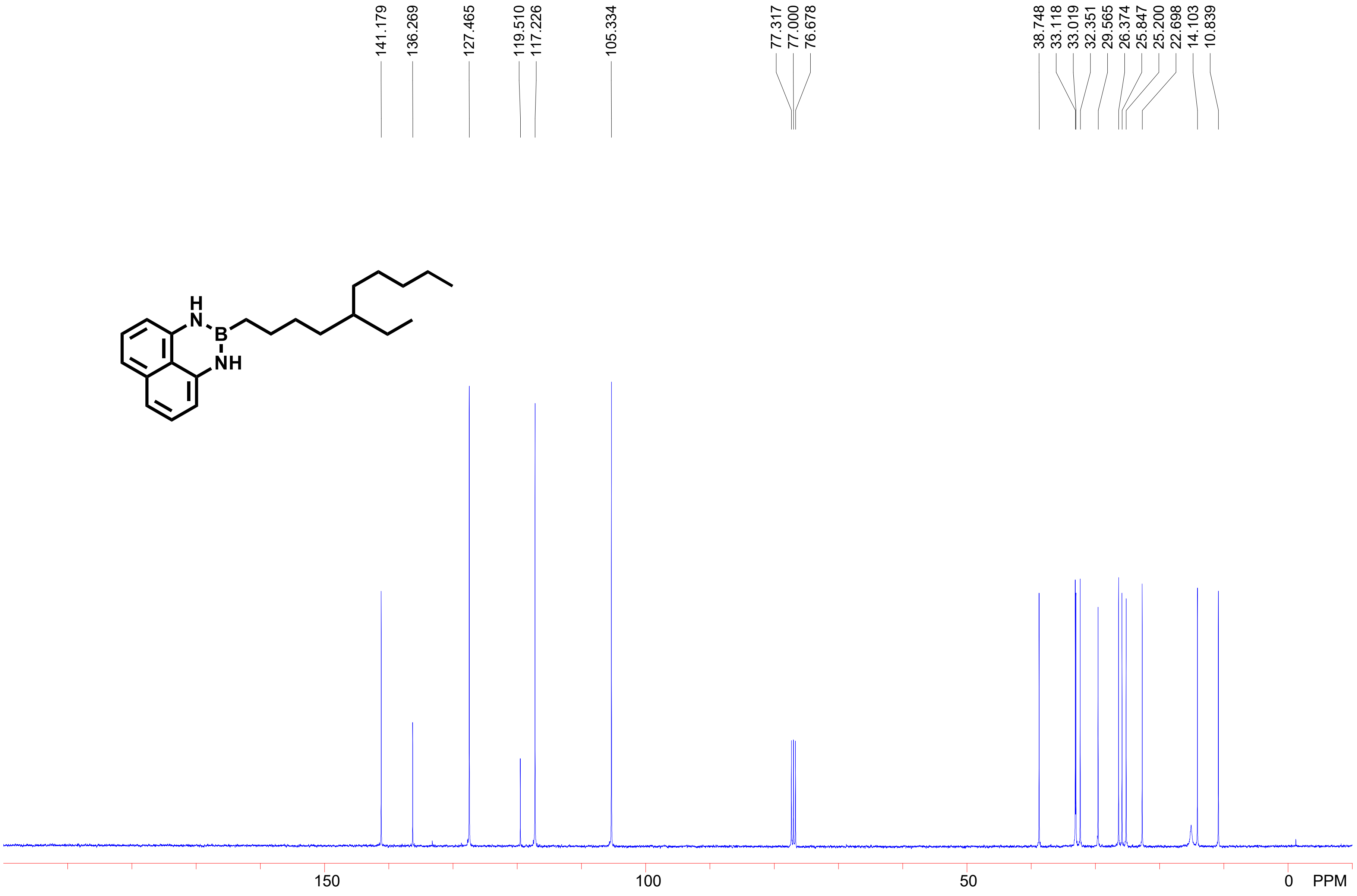

## X. HPLC Spectra for All Chiral Compounds

Comment : AD-H, n-hexane/iPrOH =99.5/0.5, 0.5 ml/min, 220 nm

HPLC Spectra

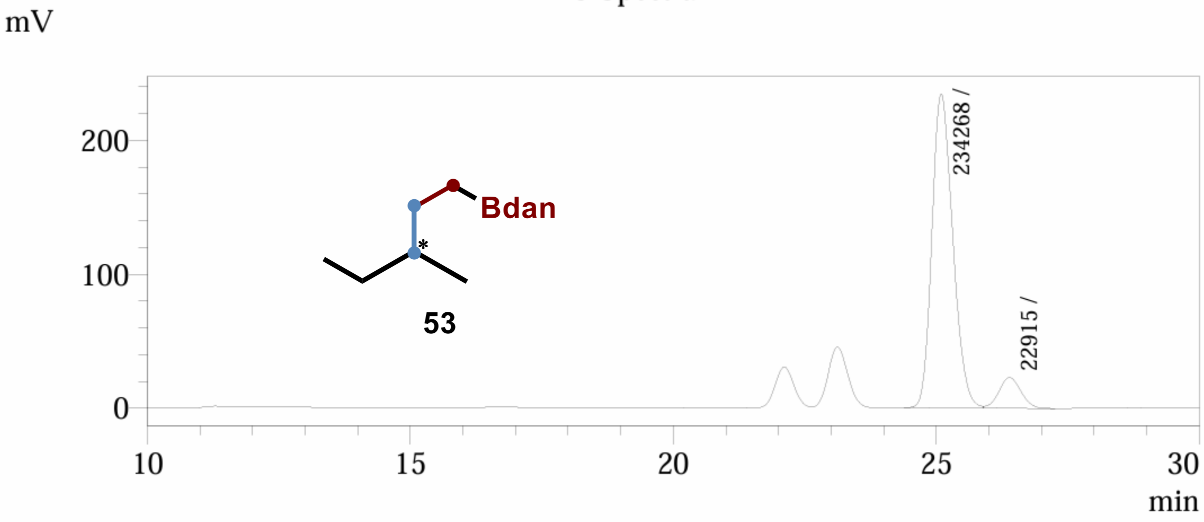


Peak Table

检测器A 220nm

| Peak Number | Retntion Time | Type | Area | Hight | Area% |
|---|---|---|---|---|---|
| 1 | 25.092 | | 6453520 | 234268 | 90.711 |
| 2 | 26.393 | V | 660848 | 22915 | 9.289 |
| 总计 | | | 7114368 | 257183 | 100.000 |

Comment : AD-H, n-hexane/iPrOH =99.5/0.5,0.5 ml/min,220 nm

HPLC Spectra

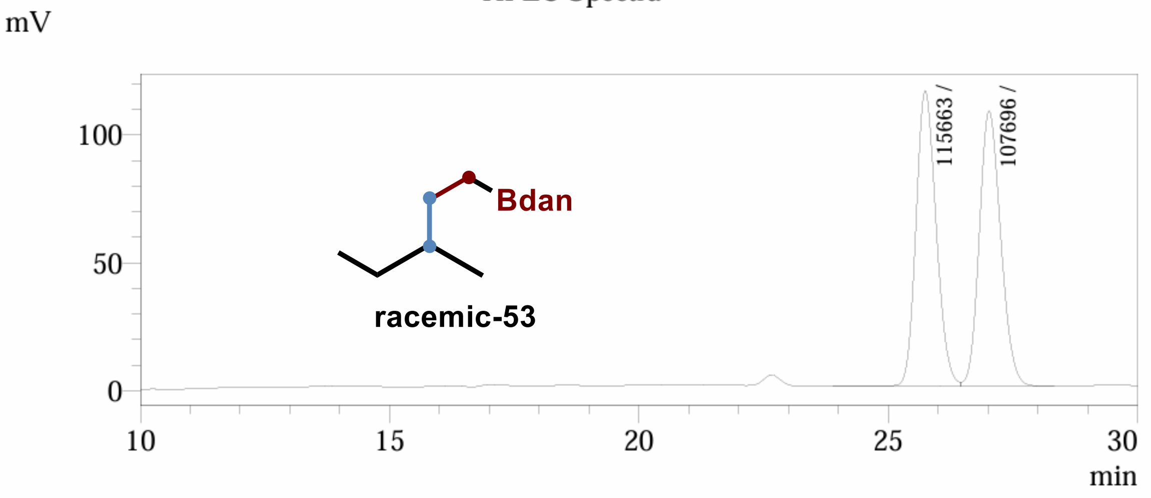


Peak Table

检测器A 220nm

| Peak Number | Retntion Time | Type | Area | Hight | Area% |
|---|---|---|---|---|---|
| 1 | 25.744 | | 3261391 | 115663 | 50.225 |
| 2 | 27.023 | SV | 3232221 | 107696 | 49.775 |
| 总计 | | | 6493612 | 223359 | 100.000 |

## REFERENCES


1. Z. Fan, X. Chen, K. Tanaka, H. S. Park, N. Y. S. Lam, J. J. Wong, K. N. Houk, J.-Q. Yu, Molecular editing of aza-arene C–H bonds by distance, geometry and chirality. *Nature* **610**, 87–93 (2022).

2. S. Das, C. D. Incarvito, R. H. Crabtree, G. W. Brudvig, Molecular recognition in the selective oxygenation of saturated C─H bonds by a dimanganese catalyst. *Science* **312**, 1941–1943 (2006).

3. J. Wencel-Delord, F. Glorius, C–H bond activation enables the rapid construction and late-stage diversification of functional molecules. *Nat. Chem.* **5**, 369–375 (2013).

4. B. Prabagar, Y. Yang, Z. Shi, Site-selective C–H functionalization to access the arene backbone of indoles and quinolines. *Chem. Soc. Rev.* **50**, 11249–11269 (2021).

5. H. Shi, Y. Lu, J. Weng, K. L. Bay, X. Chen, K. Tanaka, P. Verma, K. N. Houk, J.-Q. Yu, Differentiation and functionalization of remote C–H bonds in adjacent positions. *Nat. Chem.* **12**, 399–404 (2020).

6. B. M. Trost, Selectivity: A key to synthetic efficiency. *Science* **219**, 245–250 (1983).

7. Y.-J. Liu, H. Xu, W.-J. Kong, M. Shang, H.-X. Dai, J.-Q. Yu, Overcoming the limitations of directed C–H functionalizations of heterocycles. *Nature* **515**, 389–393 (2014).

8. D.-H. Wang, K. M. Engle, B.-F. Shi, J.-Q. Yu, Ligand-enabled reactivity and selectivity in a synthetically versatile aryl C–H olefination. *Science* **327**, 315–319 (2010).

9. D. Leow, G. Li, T.-S. Mei, J.-Q. Yu, Activation of remote meta-C–H bonds assisted by an end-on template. *Nature* **486**, 518–522 (2012).

10. R.-Y. Tang, G. Li, J.-Q. Yu, Conformation-induced remote meta-C–H activation of amines. *Nature* **507**, 215–220 (2014).

11. X.-C. Wang, W. Gong, L.-Z. Fang, R.-Y. Zhu, S. Li, K. M. Engle, J.-Q. Yu, Ligand-enabled meta-C–H activation using a transient mediator. *Nature* **519**, 334–338 (2015).

12. Z. Zhang, K. Tanaka, J.-Q. Yu, Remote site-selective C–H activation directed by a catalytic bifunctional template. *Nature* **543**, 538–542 (2017).

13. H. Chen, S. Schlecht, T. C. Semple, J. F. Hartwig, Thermal, catalytic, regiospecific functionalization of alkanes. *Science* **287**, 1995–1997 (2000).

14. K. Liao, Y.-F. Yang, Y. Li, J. N. Sanders, K. N. Houk, D. G. Musaev, H. M. L. Davies, Design of catalysts for site-selective and enantioselective functionalization of non-activated primary C–H bonds. *Nat. Chem.* **10**, 1048–1055 (2018).

15. R. Oeschger, B. Su, I. Yu, C. Ehinger, E. Romero, S. He, J. Hartwig, Diverse functionalization of strong alkyl C–H bonds by undirected borylation. *Science* **368**, 736–741 (2020).

16. C. Shu, A. Noble, V. K. Aggarwal, Metal-free photoinduced C($sp^3$)–H borylation of alkanes. *Nature* **586**, 714–719 (2020).

17. M. Wang, Y. Huang, P. Hu, Terminal C($sp^3$)–H borylation through intermolecular radical sampling. *Science* **383**, 537–544 (2024).

18. A. Vasseur, J. Bruffaerts, I. Marek, Remote functionalization through alkene isomerization. *Nat. Chem.* **8**, 209–219 (2016).

19. H. Sommer, F. Juliá-Hernández, R. Martin, I. Marek, Walking metals for remote functionalization. *ACS Cent. Sci.* **4**, 153–165 (2018).

20. C. Romano, R. Martin, Ni-catalysed remote C($sp^3$)–H functionalization using chain-walking strategies. *Nat. Rev. Chem.* **8**, 833–850 (2024).

21. J. Rodrigalvarez, F.-L. Haut, R. Martin, Regiodivergent $sp^3$ C–H functionalization via Ni-catalyzed chain-walking reactions. *JACS Au* **3**, 3270–3282 (2023).

22. D. Janssen-Müller, B. Sahoo, S.-Z. Sun, R. Martin, Tackling remote $sp^3$ C−H functionalization via Ni-catalyzed "chain-walking" reactions. *Isr. J. Chem.* **60**, 195–206 (2020).

23. Q. Wang, J. Kweon, D. Kim, S. Chang, Remote catalytic C($sp^3$)–H alkylation via relayed carbenoid transfer upon olefin chain walking. *J. Am. Chem. Soc.* **146**, 31114–31123 (2024).

24. C. Hou, Z. Liu, L. Gan, W. Fan, L. Huang, P. Chen, Z. Huang, G. Liu, Palladium-catalyzed remote hydrosulfonamidation of alkenes: Access to primary N-alkyl sulfamides by the SuFEx reaction. *J. Am. Chem. Soc.* **146**, 13536–13545 (2024).

25. W. N. Palmer, T. Diao, I. Pappas, P. J. Chirik, High-activity cobalt catalysts for alkene hydroboration with electronically responsive terpyridine and α-diimine ligands. *ACS Catal.* **5**, 622–626 (2015).

26. G. Wittig, U. Schöllkopf, Über triphenyl-phosphin-methylene als olefinbildende reagenzien (I. Mitteil). *Chem. Ber.* **87**, 1318–1330 (1954).

27. R. Zhang, T. Yu, G. Dong, Rhodium catalyzed tunable amide homologation through a hook-and-slide strategy. *Science* **382**, 951–957 (2023).

28. J. V. Obligacion, P. J. Chirik, Earth-abundant transition metal catalysts for alkene hydrosilylation and hydroboration. *Nat. Rev. Chem.* **2**, 15–34 (2018).

29. Y. Yang, The original design principles of the Y-series nonfullerene acceptors, from Y1 to Y6. *ACS Nano* **15**, 18679–18682 (2021).

30. G. Yu, J. Gao, J. C. Hummelen, F. Wudl, A. J. Heeger, Polymer photovoltaic cells: Enhanced efficiencies via a network of internal donor-acceptor heterojunctions. *Science* **270**, 1789–1791 (1995).

31. M. Granström, K. Petritsch, A. C. Arias, A. Lux, M. R. Andersson, R. H. Friend, Laminated fabrication of polymeric photovoltaic diodes. *Nature* **395**, 257–260 (1998).

32. H. Yan, Z. Chen, Y. Zheng, C. Newman, J. R. Quinn, F. Dötz, M. Kastler, A. Facchetti, A high-mobility electron-transporting polymer for printed transistors. *Nature* **457**, 679–686 (2009).

33. J. Y. Oh, S. Rondeau-Gagné, Y.-C. Chiu, A. Chortos, F. Lissel, G.-J. N. Wang, B. C. Schroeder, T. Kurosawa, J. Lopez, T. Katsumata, J. Xu, C. Zhu, X. Gu, W.-G. Bae, Y. Kim, L. Jin, J. W. Chung, J. B. H. Tok, Z. Bao, Intrinsically stretchable and healable semiconducting polymer for organic transistors. *Nature* **539**, 411–415 (2016).

34. Y.-Q. Zheng, Y. Liu, D. Zhong, S. Nikzad, S. Liu, Z. Yu, D. Liu, H.-C. Wu, C. Zhu, J. Li, H. Tran, J. B.-H. Tok, Z. Bao, Monolithic optical microlithography of high-density elastic circuits. *Science* **373**, 88–94 (2021).

35. S. Wang, T. Liu, Y. Huang, C. Du, D. Wang, X. Wang, Q. Lv, Z. He, Y. Zhai, B. Sun, J. Sun, The effect of lengths of branched-chain fatty alcohols on the efficacy and safety of docetaxel-prodrug nanoassemblies. *Acta Pharm. Sin. B* **14**, 1400–1411 (2024).

36. J. Guo, Z. Cheng, J. Chen, X. Chen, Z. Lu, Iron- and cobalt-catalyzed asymmetric hydrofunctionalization of alkenes and alkynes. *Acc. Chem. Res.* **54**, 2701–2716 (2021).

37. Y. Liu, Y. Li, Q. Yang, J.-D. Yang, L. Zhang, S. Luo, Prediction of bond dissociation energy for organic molecules based on a machine-learning approach. *Chin. J. Chem.* **42**, 1967–1974 (2024).

38. Y. Bao, C. Zheng, K. Xiong, C. Hu, P. Lu, Y. Wang, Z. Lu, Enantioconvergent hydroboration of E/Z-mixed trisubstituted alkenes. *J. Am. Chem. Soc.* **146**, 21089–21098 (2024).

39. J. V. Obligacion, P. J. Chirik, Bis(imino)pyridine cobalt-catalyzed alkene isomerization–hydroboration: A strategy for remote hydrofunctionalization with terminal selectivity. *J. Am. Chem. Soc.* **135**, 19107–19110 (2013).

40. J. Peng, J. H. Docherty, A. P. Dominey, S. P. Thomas, Cobalt-catalysed Markovnikov selective hydroboration of vinylarenes. *Chem. Commun.* **53**, 4726–4729 (2017).

41. C. Bianchini, D. Gatteschi, G. Giambastiani, I. Guerrero Rios, A. Ienco, F. Laschi, C. Mealli, A. Meli, L. Sorace, A. Toti, F. Vizza, Electronic influence of the thienyl sulfur atom on the oligomerization of ethylene by cobalt(II) 6-(Thienyl)-2-(imino)pyridine catalysis. *Organometallics* **26**, 726–739 (2007).

42. Y. Li, Y. Li, H. Shi, H. Wei, H. Li, I. Funes-Ardoiz, G. Yin, Modular access to substituted cyclohexanes with kinetic stereocontrol. *Science* **376**, 749–753 (2022).

43. X. Liu, W. Zhang, Y. Wang, Z.-X. Zhang, L. Jiao, Q. Liu, Cobalt-catalyzed regioselective olefin isomerization under kinetic control. *J. Am. Chem. Soc.* **140**, 6873–6882 (2018).

44. X. Liu, Q. Zhu, D. Chen, L. Wang, L. Jin, C. Liu, Aminoazanium of DABCO: An amination reagent for alkyl and aryl pinacol boronates. *Angew. Chem. Int. Ed.* **59**, 2745–2749 (2020).

45. L. Lin, C. Romano, C. Mazet, Palladium-catalyzed long-range deconjugative isomerization of highly substituted α,β-unsaturated carbonyl compounds. *J. Am. Chem. Soc.* **138**, 10344–10350 (2016).

46. M. Wang, S. Liu, H. Liu, Y. Wang, Y. Lan, Q. Liu, Asymmetric hydrogenation of ketimines with minimally different alkyl groups. *Nature* **631**, 556–562 (2024).

47. Y.-F. Zhang, B. Wang, Z. Chen, J.-R. Liu, N.-Y. Yang, J.-M. Xiang, J. Liu, Q.-S. Gu, X. Hong, X.-Y. Liu, Asymmetric amination of alkyl radicals with two minimally different alkyl substituents. *Science* **388**, 283–291 (2025).

48. Y. Wu, L. Shi, L. Xu, J. Ying, X. Miao, B. Hua, Z. Chen, J. L. Sessler, F. Huang, Supramolecular docking structure determination of alkyl-bearing molecules. *Nature* **640**, 676–682 (2025).

49. Y. Zhao, S. Ge, Synergistic hydrocobaltation and borylcobaltation enable regioselective migratory triborylation of unactivated alkenes. *Angew. Chem. Int. Ed.* **61**, e202116133 (2022).

50. T. T. Nguyen, M. J. Koh, T. J. Mann, R. R. Schrock, A. H. Hoveyda, Synthesis of E- and Z-trisubstituted alkenes by catalytic cross-metathesis. *Nature* **552**, 347–354 (2017).

51. H. Kim, S. Y. Choi, S. Shin, Asymmetric synthesis of dihydropyranones *via* gold(I)-catalyzed intermolecular [4+2] annulation of propiolates and alkenes. *Angew. Chem. Int. Ed.* **57**, 13130–13134 (2018).

52. H. Li, Z. Lai, M. Peng, L. Ning, Q. Dong, Y. Hou, J. An, One-pot sequential hydrogen isotope exchange/reductive deuteration for the preparation of α,β-deuterated alcohols using deuterium oxide. *Org. Lett.* **24**, 5319–5323 (2022).

53. D. Das, G. Sahoo, A. Biswas, R. Samanta, Rh(III)-catalyzed synthesis of highly substituted 2-pyridones using fluorinated diazomalonate. *Chem. Asian. J.* **15**, 360–364 (2020).

54. Z. Zheng, L. Chen, C. Qian, X. Zhu, Y. Yang, J. Liu, Y. Yang, Y. Liang, Copper-catalyzed synthesis of 2-acylbenzo[b]thiophenes from 3-(2-iodophenyl)-1-arylpropan-1-ones and potassium sulfide under aerobic conditions. *Org. Biomol. Chem.* **16**, 8020–8024 (2018).

55. Q. Xia, X. Bao, C. Sun, D. Wu, X. Rong, Z. Liu, Y. Gu, J. Zhou, G. Liang, Design, synthesis and biological evaluation of novel 2-sulfonylindoles as potential anti-inflammatory therapeutic agents for treatment of acute lung injury. *Eur. J. Med. Chem.* **160**, 120–132 (2018).

56. J.-P. Fontaine, V. Lapointe, M. Filliâtre, G. Bélanger, Synthesis of substituted indolines through photocatalyzed decarboxylative radical arylation. *J. Org. Chem.* **88**, 6557–6564 (2023).

57. R. Zhang, Z. Liu, Y. Liu, H. Yang, X. Wang, Z. Han, Z. Wang, K. Ding, Palladium-catalyzed regioselective asymmetric chemodivergent allylation of oxazolones with morita–baylis–hillman adducts. *CCS Chem.* **5**, 2790–2798 (2023).

58. B. Chen, L. Pagès, R. Dollet, C. Kouklovsky, S. Prévost, A. de la Torre, Regio- and chemoselective double allylic substitution of alkenyl vic-diols. *Org. Lett.* **26**, 2393–2397 (2024).

59. K. Kubota, E. Yamamoto, H. Ito, Copper(I)-catalyzed borylative exo-cyclization of alkenyl halides containing unactivated double bond. *J. Am. Chem. Soc.* **135**, 2635–2640 (2013).

60. S. Mayr, M. Marin-Luna, H. Zipse, Size-driven inversion of selectivity in esterification reactions: Secondary beat primary alcohols. *J. Org. Chem.* **86**, 3456–3489 (2021).

61. X. Chen, Z. Cheng, J. Guo, Z. Lu, Asymmetric remote C─H borylation of internal alkenes via alkene isomerization. *Nat. Commun.* **9**, 3939 (2018).

62. R. A. Altman, A. Shafir, A. Choi, P. A. Lichtor, S. L. Buchwald, An improved Cu-based catalyst system for the reactions of alcohols with aryl halides. *J. Org. Chem.* **73**, 284–286 (2008).

63. F.-H. Yang, B. Hao, X. Yue, P.-C. Ma, Fluorescence and stimuli-responsive performance of polymer composites filled with tetraphenylethene derivatives. *Polym. Chem.* **13**, 3126–3135 (2022).

64. L. Britton, M. Skrodzki, G. S. Nichol, A. P. Dominey, P. Pawluć, J. H. Docherty, S. P. Thomas, Manganese-catalyzed C($sp^2$)–H borylation of furan and thiophene derivatives. *ACS Catal.* **11**, 6857–6864 (2021).

65. C. Chen, H. Wang, T. Li, D. Lu, J. Li, X. Zhang, X. Hong, Z. Lu, Cobalt-catalyzed asymmetric sequential hydroboration/isomerization/hydroboration of 2-aryl vinylcyclopropanes. *Angew. Chem. Int. Ed.* **61**, e202205619 (2022).

66. L. Krause, R. Herbst-Irmer, G. M. Sheldrick, D. Stalke, Comparison of silver and molybdenum microfocus x-ray sources for single-crystal structure determination. *J. Appl. Cryst.* **48**, 3–10 (2015).

67. O. V. Dolomanov, L. J. Bourhis, R. J. Gildea, J. A. K. Howard, H. Puschmann, OLEX2: A complete structure solution, refinement and analysis program. *J. Appl. Cryst.* **42**, 339–341 (2009).

68. Q. Liu, X.-D. Su, B.-B. Zhang, H.-R. Kong, Y.-L. Tu, Z.-X. Wang, X.-Y. Chen, dn-Alkyl phosphonium iodide salts as radical dn-alkylating reagents and their applications in the photoinduced synthesis of dn-alkylated heterocycles. *CCS Chem.* **7**, 993–1004 (2025).

69. F. Hou, Y. Ning, L. Song, Z. Tan, J. Yang, Z. Liu, F.-E. Chen, Rhodium-catalyzed asymmetric hydroboration/cyclization of 1,6-enynes enabled by spirosiladiphosphine ligands: Constructing chiral five-membered rings with a boron handle. *Org. Lett.* **25**, 7810–7815 (2023).

70. Z.-Y. Xiao, Z.-L. Wang, Y.-H. Xu, Copper-catalyzed ring-opening hydrosilylation and hydroboration of arylidenecyclopropanes. *Chin. J. Chem.* **43**, 385–392 (2025).

71. Y. Liao, S. Liu, J. Lin, K. Chen, J. Tang, L. Zou, Q. Peng, X. Wang, Binuclear cobalt complex-catalyzed dehydrogenation of ammonia borane and transfer hydrogenation of alkenes, carbonyls and nitriles: High efficiency under mild conditions. *Chin. J. Chem.* **44**, 987–994 (2026).
